# Analysis of splice variants of the human protein disulfide isomerase (*P4HB*) gene

**DOI:** 10.1186/s12864-020-07164-y

**Published:** 2020-11-04

**Authors:** Daniela Kajihara, Chung-Chau Hon, Aimi Naim Abdullah, João Wosniak, Ana Iochabel S. Moretti, Joice F. Poloni, Diego Bonatto, Kosuke Hashimoto, Piero Carninci, Francisco R. M. Laurindo

**Affiliations:** 1 Vascular Biology Laboratory, LIM-64, Heart Institute (InCor), University of Sao Paulo School of Medicine, Av. Eneas Carvalho Aguiar, 44, Annex 2, 9th floor, Sao Paulo, CEP 05403-000 Brazil; 2 Laboratory for Transcriptome Technology, Division of Genomic Medicine, RIKEN Center for Integrative Medical Sciences, Yokohama, Japan; 3 Laboratory for Genome Information Analysis, Division of Genomic Medicine, RIKEN Center for Integrative Medical Sciences, Yokohama, Japan; 4 Department of Molecular Biology and Biotechnology, Biotechnology Center of the Federal University of Rio Grande do Sul, Federal University of Rio Grande do Sul (UFRGS), Porto Alegre, RS Brazil; 5 Laboratory of Computational Biology, Institute for Protein Research, Osaka University, Osaka, 565-0871 Japan

## Abstract

**Background:**

Protein Disulfide Isomerases are thiol oxidoreductase chaperones from thioredoxin superfamily with crucial roles in endoplasmic reticulum proteostasis, implicated in many diseases. The family prototype PDIA1 is also involved in vascular redox cell signaling. PDIA1 is coded by the *P4HB* gene. While forced changes in *P4HB* gene expression promote physiological effects, little is known about endogenous *P4HB* gene regulation and, in particular, gene modulation by alternative splicing. This study addressed the *P4HB* splice variant landscape.

**Results:**

Ten protein coding sequences (Ensembl) of the *P4HB* gene originating from alternative splicing were characterized. Structural features suggest that except for *P4HB-021*, other splice variants are unlikely to exert thiol isomerase activity at the endoplasmic reticulum. Extensive analyses using FANTOM5, ENCODE Consortium and GTEx project databases as RNA-seq data sources were performed. These indicated widespread expression but significant variability in the degree of isoform expression among distinct tissues and even among distinct locations of the same cell, e.g., vascular smooth muscle cells from different origins. *P4HB-*02, *P4HB-*027 and *P4HB-*021 were relatively more expressed across each database, the latter particularly in vascular smooth muscle. Expression of such variants was validated by qRT-PCR in some cell types. The most consistently expressed splice variant was *P4HB-*021 in human mammary artery vascular smooth muscle which, together with canonical *P4HB* gene, had its expression enhanced by serum starvation.

**Conclusions:**

Our study details the splice variant landscape of the *P4HB* gene, indicating their potential role to diversify the functional reach of this crucial gene. *P4HB*-021 splice variant deserves further investigation in vascular smooth muscle cells.

**Supplementary Information:**

The online version contains supplementary material available at 10.1186/s12864-020-07164-y.

## Background

**Fig. 1:**
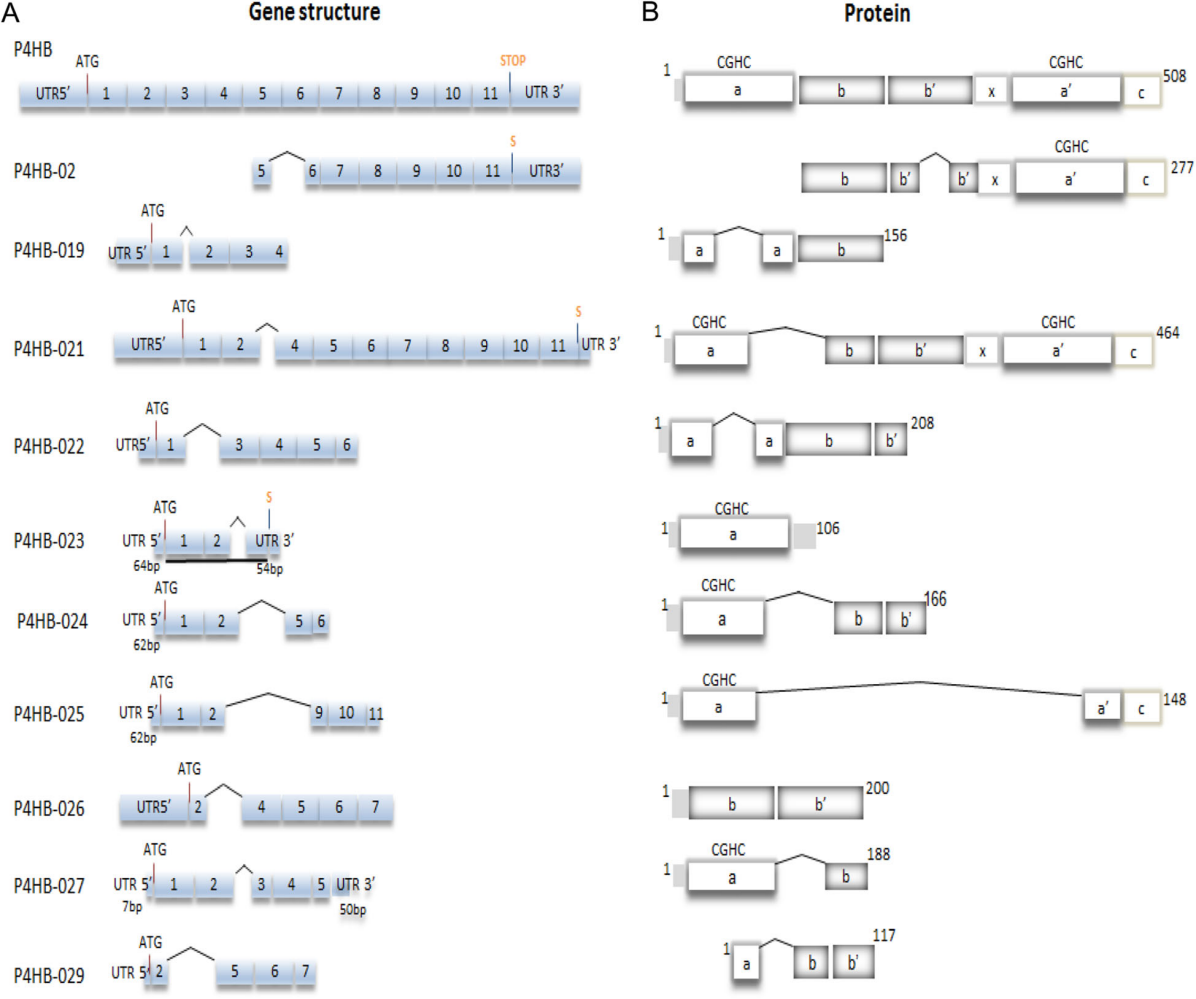
*P4HB* gene and protein organization. (**A**). Gene structure of human *P4HB* gene and predicted splice variants. Exons are represented by boxes; 5′ or 3′ untranslated regions (UTR) are also depicted. ATG indicates the start codon and STOP depicts the stop codon. The underline in *P4HB*-23 indicates the translated regions. The coding sequence of *P4HB*-027 includes the 3′ UTR, which does not contain a stop codon. (**B**). PDIA1 protein domain organization and predicted structure of alternatively processed isoforms. PDIA1 protein is composed of five domains: *a, b, b’, a’* and c. The *a* and *a* catalytic domains contain the thioredoxin redox-active CGHC motifs (white boxes), while the *b* and *b’* domains (gray boxes) are noncatalytic, structured as thioredoxin folds enriched in hydrophobic residues involved in substrate binding and chaperone activity. An unstructured *x-*linker stretch is located between *b’* and *a’* domains and confers flexibility to PDI. The C-terminal *c-* domain contains the KDEL sequence, an ER retrieval signal

Protein disulfide isomerases (PDIs) are a family of thiol oxidoreductase chaperones belonging to the thioredoxin superfamily, which also includes thioredoxins isoforms, glutaredoxins and peroxiredoxins [1, 2]. PDIs comprise at least 21 genes. The canonical activities of most PDIs are oxidation, reduction or isomerization of protein substrate cysteine thiols throughout their processing at the endoplasmic reticulum lumen [3]. In addition, many PDIs display a chaperone activity for which the thiol motifs are dispensable. The prototype of PDI family is PDIA1. In addition to its essential role in endoplasmic reticulum-associated proteostasis and redox balance, PDIA1 has been described by our and other groups to have additional important effects on thiol-related signaling of processes including Nox family NADPH oxidase activation [4, 5], vascular cell migration, smooth muscle cell cytoskeletal remodeling [6], thrombosis [7, 8], platelet activation [9] and vascular remodeling [10] Such effects appear related to locations of PDIA1 outside the endoplasmic reticulum, particularly the cell-surface or extracellular milieu and possibly the cytosol [5]. In fact, PDIA1 undergoes externalization through Golgi-dependent and independent routes in endothelial and vascular smooth muscle cells [11]. PDIA1 is coded by the *P4HB* gene, named after the well-known PDI role as the beta subunit heterodimer of prolyl-4-hydroxylase [12]. The human *P4HB* gene (Ensembl ID: ENSG00000185624) contains 11 exons (Fig. 1A and Table S1) and its genomic location is on chromosome 17 (17q25.3, reference GrCh37.p13 NC_000017.10).

However, little is known about *P4HB* gene regulation, as most studies tend to focus on its abundantly expressed protein product. Some PDIs exhibit an unfolded protein response-sensitive element in their promoter region, but this is not the case of *P4HB* [13]. We described recently a remarkably conserved pattern of gene clustering between the PDI and the RhoGDI (Rho guanine-dissociation inhibitor, a regulator of RhoGTPases affecting the cytoskeleton) gene families, with a microsyntenic arrangement dating to > 820 million-years, suggesting that functional convergence and protein association, indeed shown for PDIA1 and RhoGDIα, worked against gene separation throughout evolution [14]. These considerations indicate that at least in some instances the gene-level regulation of PDIA1 appears relevant. Moreover, the amount of forced PDIA1 expression or silencing appears to be associated with several physiological effects, despite the already high levels of PDI protein in general [5].

The *P4HB* product, PDIA1, is a 55 kDa U-shaped protein with four thioredoxin tandem domains composing a modular architecture, named *a, b, b’* and *a*’, plus a C-terminal domain named *c*. The *a* and *a*’ domains contain redox-active thioredoxin domains bearing Cys-X-X-Cys (CGHC) motifs. Domains *b* and *b’* comprise non-catalytic thioredoxin folds without the redox domains; they are enriched in hydrophobic residues responsible for substrate binding sites and for the bulk of PDI chaperone activity [15–17]. The unstructured *x-* linker, a 19-amino acid stretch between *b’* and *a’* domains, allows flexibility of this inter-domain region [16, 17] and confers considerable mobility to PDIA1, with an open configuration when oxidized and a closed one when reduced [18]. Domain *c* at the C-terminus depicts the KDEL sequence, responsible for ER retrieval of PDIA1 upon interaction with the KDEL receptor [19].

One important aspect of gene regulation is the occurrence of alternative splicing generated at transcriptional level, which can be a source of (patho) physiological protein diversity with the production of long or short variants. Some spliced isoforms can display premature transcription termination codons and others can acquire introns, generating products either truncated or with aberrant folding [20]. The most common types of alternative splicing relate to alternative transcription start sites or termination sites [21]. Splice variants can have important roles in a number of physiological regulatory processes and the use of different splice variants in adverse conditions, known as isoform switching [22], is connected to many diseases [23, 24].

However, there is essentially no information with respect to alternative splicing variants of *P4HB* gene. This question is important in perspective with the multiple, biologically relevant effects of PDIA1 discussed above, as well as with respect to its peculiar modular structure-function correlations, which can implicate in an array of potentially important functions for spliced isoforms. The aim of this study is to analyze the landscape of alternative spliced isoforms of PDIA1, with particular emphasis in the vascular smooth muscle cell.

## Results

### Alternative splice variants of *P4HB* gene

The *P4HB* gene has 24 transcripts in human genome (Ensembl, GRCh38.p10), comprising the canonical isoform plus 10 protein coding sequences, 1 nonsense mediated decay, 3 processed transcripts and 9 retained introns. The main variants described as protein-coding (Ensembl) are shown in Fig. 1 and available from http://www.ensembl.org [25]. All these 10 isoforms are supported by The Human Protein Atlas (http://www.proteinatlas.org) and annotated in UniProt. Table S1 summarizes the information about *P4HB* splice variants, including Transcript ID (Ensembl), UniProt identification, nucleotide and protein length, molecular mass and putative signal peptide.

The predicted organization of each protein coding isoform is depicted in Fig. 1B. Of note, *P4HB*-02 does not predictably display the classical ATG start codon, though it was possible to detect CAGE tags in that region. Moreover, except for *P4HB*-02 and *P4HB*-021, the splice variants are not predicted to have a classical stop codon, while *P4HB*-023 (detected) and *P4HB*-027 (possibly) have stop codons at the 3′-UTR regions. Except for isoform *P4HB*-021, which depicts all four thioredoxin domains (with *a* and *b* partially truncated - see below), the predicted *P4HB* isoform products lack one or more domains or depict incomplete forms of some domains, generating variable combinations with potential to display alternative functions, since the unique thiol isomerase activity of *PDIA1* requires all 4 (*a, a’, b, b’*) domains [26]. For example, *P4HB*-019 has a fragmented *a* domain lacking 36 amino acids which include the redox-active motif and *P4HB*-027 has one truncated *a* domain with a redox-active motif and one truncated *b* domain. *P4HB*-021 is predicted to have the signal peptide, the 2 active CGHC domains and exhibits only a lack of 44 amino acids between *a* and *b* domains. *P4HB*-02 and *P4HB*-021 are the only to display the intact C-terminus with the KDEL motif, indicating that eventual protein products generated from other isoforms may not be retrievable to the endoplasmic reticulum.

Taking advantage of CAGE tags to determine expression levels, we analyzed an upstream region of *P4HB*-02 and *P4HB* genes. For *P4HB*-02, we used 250 bp upstream of putative coding region to verify which samples presented higher normalized tags per million (TPM). For this, we selected a subset of data from FANTOM5 called FANTOM5 CAGE Phase1 CTSS human which displayed the highest TPM and was composed of samples from pancreas, Sertoli cells, smooth muscle cells (aortic), leiomyoma cell line and fibroblast (aortic adventitial) (Fig. S1). In addition, the information of ENCODE CAGE was also analyzed showing Hep G2, K562, HUVEC and Nhek cell lines (Fig. S1). We also checked for the presence of CAGE tags upstream of *P4HB* gene using the same data and the result was similar. The benefit of CAGE tags is the possibility of revealing a range of alternative transcription initiation events even in exonic coding sequences [27, 28]. These data were important to select samples from ENCODE RNA-seq (described below), filtering for samples in which CAGE tags were identified and more representative to such analysis.

### Expression profiling of *P4HB* splice variants in FANTOM5 database

We next addressed an overview of *P4HB* gene and spliced variant expression profiling in different cell lines and tissues, using a number of distinct databases: FANTOM5, ENCODE and GTEx.

**Fig. 2:**
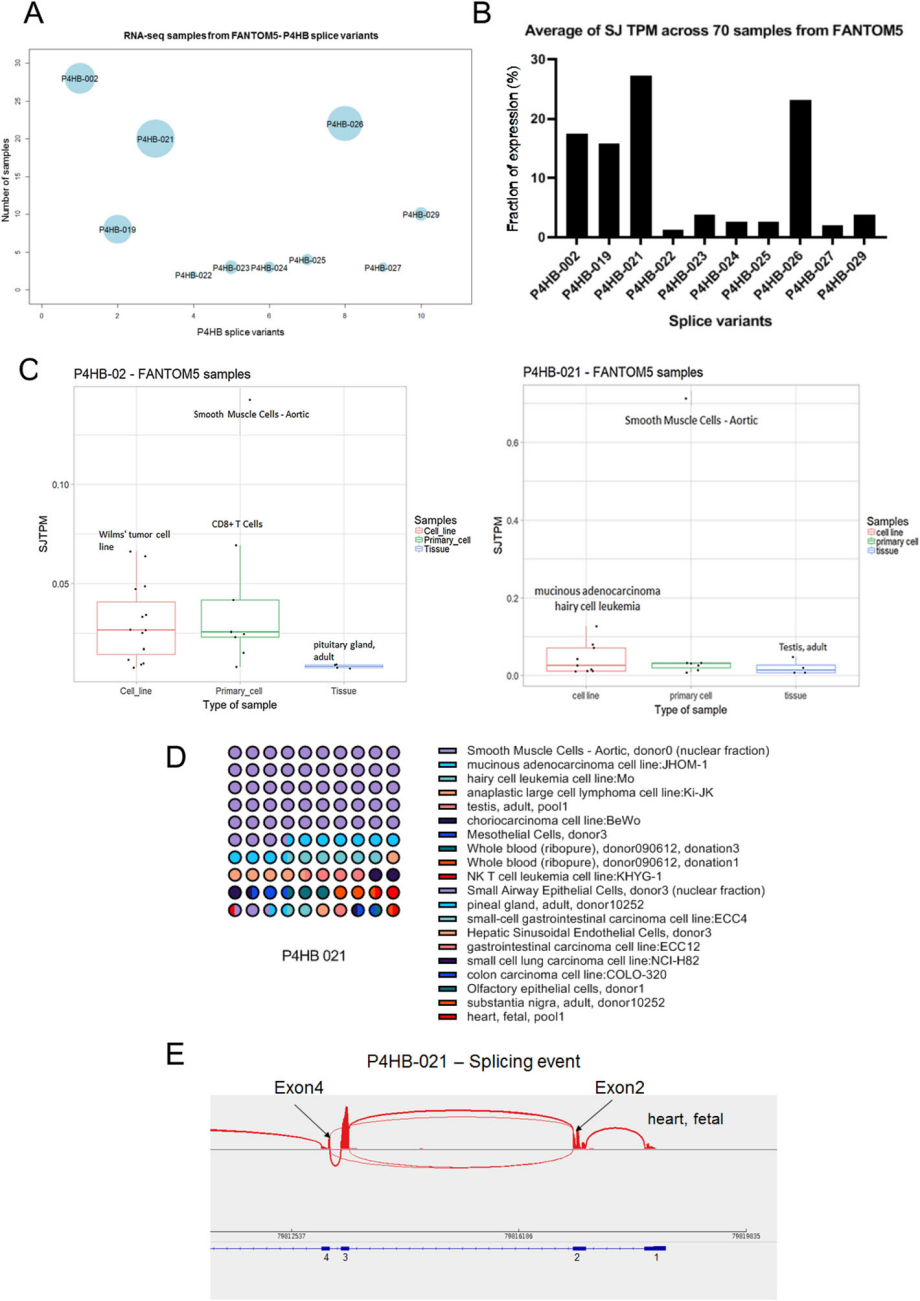
Distributions of *P4HB* splice variant expression in RNA-seq from FANTOM5. Splice Junction Tag per Million (SJ TPM) is a unit to count the number of a specific isoform junction normalized by the total number of *reads* for each RNA-seq dataset. **a** The graph shows the *P4HB* splice variant distribution. The circle radius indicates the average expression of all samples in SJ-TPM for each splice variant detected. **b** Fraction of expression of *P4HB* splice variants in FANTOM5. **c** The expression of *P4HB*-02 and *P4HB*-021 in three types of cell: primary cell, tissue and cell line. **d** Representative diagram of *P4HB*-021 expression in all analyzed samples. The amount of dots is proportional to the relative expression. **e** Visualization of splicing event of *P4HB*-021. The blue diagram at the bottom indicates a part of *P4HB* gene, in reverse direction from exon 1 to exon 4. The black arrows indicate the exon 2, with a splice junction to exon 4 associated to an absence of exon 3. The total number of RNA-seq data was 70 samples

First, the FANTOM5 project provides atlases of long noncoding RNAs and microRNAs and their promoters, with accompanying RNA-seq and short RNA transcriptome data [29]. We used information of all FANTOM5 RNA-seq libraries (70 samples) [30], in order to prospectively analyze *P4HB* splice junctions. These samples were composed of cell lines (*n* = 32), primary cells (*n* = 27) and tissues (*n* = 11). In some cases (*n* = 6), the average of triplicate data (whole blood samples, CD19 B cells and CD8 T cells) from the same donor was used. These 70 samples of FANTOM5 project were used to build Fig. 2a and b, which show profiles of expression for the 10 protein-coding isoforms from Fig. 1. Figure 2b represents the percentage of splice variant abundance in this set of samples from FANTOM5, showing that almost 30% of total isoform fraction is represented by *P4HB-021*. In Fig. 2c, two representative examples of the most expressed isoforms (*P4HB-02* and *P4HB- 021*) are shown for different cells and tissues.

These results showed, in brief, the following overview: three processed transcripts and ten protein coding isoforms. The variant *P4HB*-021 was significantly represented, particularly in aortic smooth muscle cell, followed in this cell type by *P4HB*-019, *P4HB-*023, *P4HB*-027 and *P4HB*-02. The samples with overall highest number of expressed *P4HB* isoforms were smooth muscle cells – aortic samples, followed by CD19+ B cells and mucinous adenocarcinoma cell line. The most frequently expressed splice variant across all the 70 samples was *P4HB*-02, present in 28 samples, while *P4HB*-021 and *P4HB*-027 depicted the highest splice junction TPMs. The isoform *P4HB*-021 had its highest level of expression in aortic smooth muscle cells (Fig. 2d) but due the relatively low number of samples, we focused this analysis into more abundant SMC data from ENCODE and a recently published study [31] (following sections).

To visualize the BAM file in IGV platform, we selected the splice variant *P4HB*-027 to check the splice junction in 4 different cell types. Fig. S2 illustrates the splicing event in the middle of *P4HB*-027 exon 3. Using this tool, it was possible to visualize the splice junction of different variants among multiple samples. Additionally, the *P4HB*-027 splice junction in exon 3 is not present in all the 4 samples analyzed, as indicated by the black arrow in Fig. S2. Also, in Fig. 2e there is a plot for *P4HB*-021 displaying the splicing event. Sashimi plots were generated in the IGV-Sashimi, which allows one to select a specific genomic region and to detect events of isoform usage [32].

### Expression profiling of *P4HB* splice variants in RNA-seq ENCODE database

**Fig. 3:**
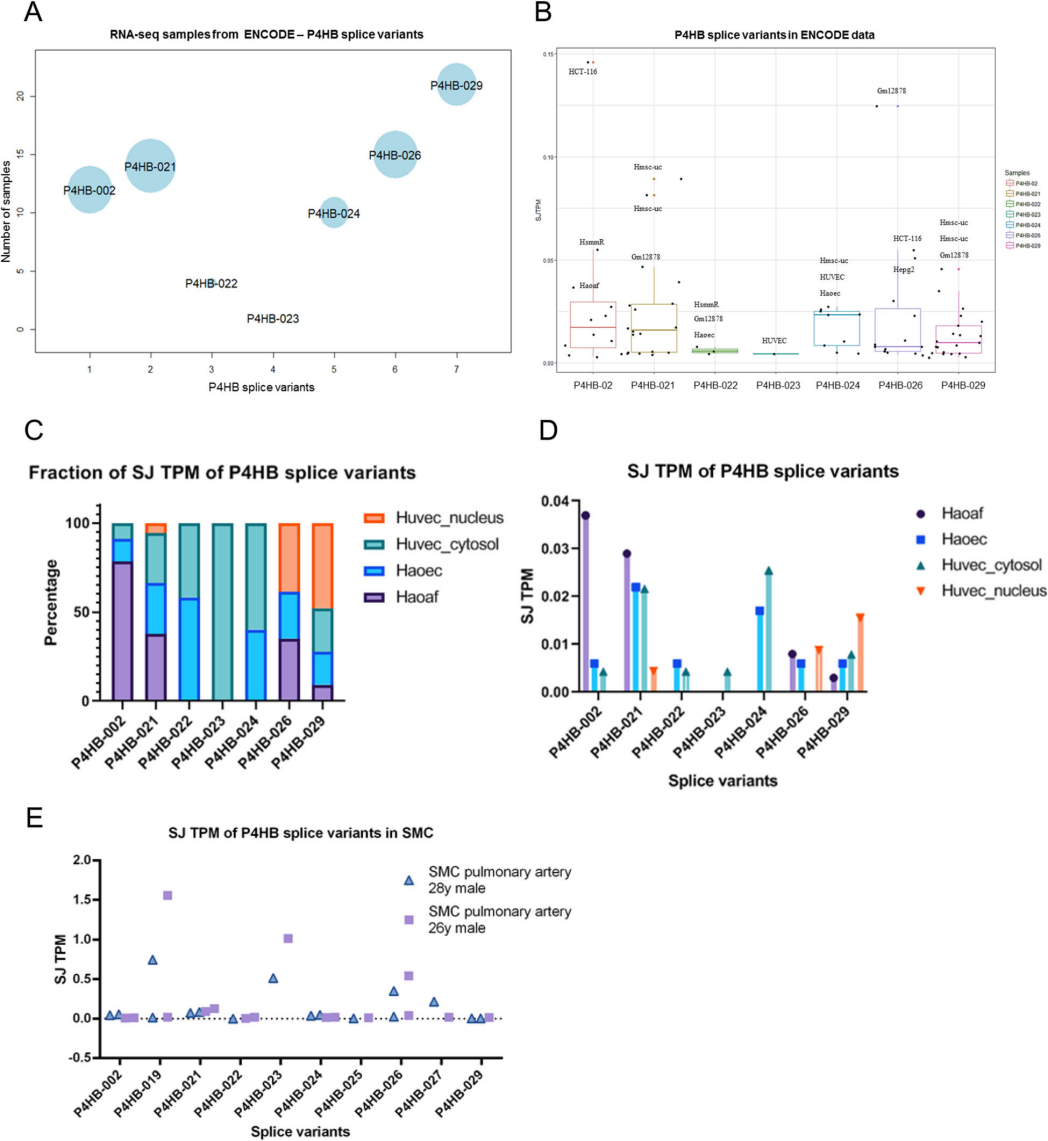
Distribution of splice variant expression in RNA-seq from ENCODE. Splice Junction Tag per Million (SJ TPM) is a unit to count the number of specific isoform junctions normalized by the total number of *reads* for each RNA-seq dataset. The circle radius indicates the average expression of all samples in SJ TPM for each splice variant detected. Samples from ENCODE (*n* = 27) were analyzed. **b** Distribution of *P4HB* splice variant expression, showing 7 detected splice variants. **c** Fractional distribution of *P4HB* splice variants expressed in distinct cell types (HUVEC, HAoEC, HaoAF). **d** SJ TPM of *P4HB* gene and splice variants in a set of 12 different cell types; **e** SJ TPM of P4HB gene and splice variants of SMC from pulmonary artery from 2 different donors

The Encyclopedia of DNA Elements (ENCODE) [33] has a set of different types of experiments such as Exon Arrays, Chip-Seq and RNA-seq analysis, available at http://www.encodeproject.org. Here we used the ENCODE Caltech RNA-seq data and CSHL/ENCODE RNA-seq data to analyze 27 RNA-seq datasets including 12 different cell lines, 5 of which cancer cell lines. Their choice was justified by the presence of CAGE peaks [34], which are tags for gene expression, as detailed in Methods. The distribution of splice variants counted by splice junction (tags per million) in the ENCODE datasets is shown in Fig. 3a. In this graph, the most representative (i.e., expressed in most samples) was *P4HB*-029, but the isoforms most expressed (in SJ TPM) were *P4HB*-02 and *P4HB*-021. HCT-116 (human colon cancer) cell line, Gm12878 (human lymphoblastoid cell line), Hmsc (Human mesenchymal stem cell line) and Hsmm (human skeletal muscle myoblast cell line) were the ones most represented in this set (Fig. 3b). In addition to this analysis, we performed a separate one focusing on endothelial cells (HUVEC and HAoEC) and aortic adventitial fibroblasts (HaoAF), shown in Fig. 3c-d. Isoform *P4HB*-02 is well expressed in aortic adventitial fibroblasts, *P4HB*-021 in fibroblasts and 2 types of endothelial cells and *P4HB*-024 in two other endothelial cell types.

We next applied the same pipeline above to identify and count the splice junction TPM (tag per million) to investigate *P4HB* gene expression in polyA RNA-seq ENCODE human datasets (https://www.encodeproject.org/) from donors (primary cell). We focused on data from pulmonary artery smooth muscle cells, which derive from two male individuals. *P4HB*-019, 023 and 026 were more expressed in these cells (Fig. 3e), representing around 0.7% (*P4HB*-019) and 0.5% (*P4HB*-023) of total *P4HB*. In all such cases, however, the expression of isoforms was relatively small vs. the canonical isoform (Fig. 3).

### Expression profiling of *P4HB* splice variants in GTEx database

**Fig. 4:**
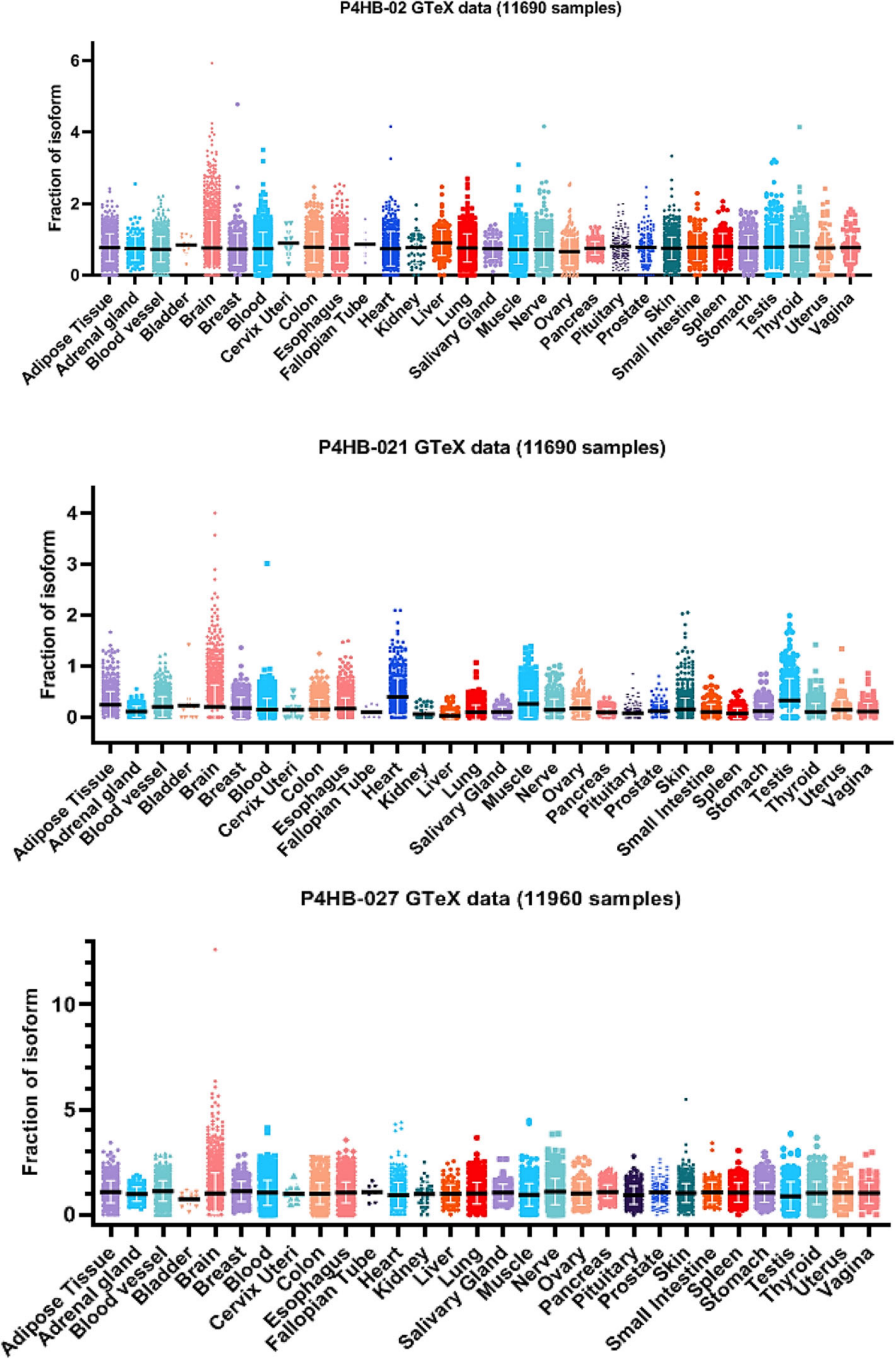
Quantification of *P4HB* splice variants (*P4HB*-02, *P4HB*-021 and *P4HB*-027) as splice junction tags per million (SJ TPM) relative to *P4HB* expression. The total number of samples was 11,960 within 30 different tissues. Black lines depict the mean and standard deviations are shown in white

The Genotype-Tissue Expression Project (GTEx) is one of many large cohort studies comprising a significant number of transcriptomic data, including RNA-seq from various tissues. Here, we used 11,690 RNA-seq data from different tissues and conditions listed in Table S3. These sets of data are highly enriched in whole blood (407 samples), blood vessel (913 samples) and heart (600 samples). Figure 4 shows the quantification of three *P4HB* splice variants (*P4HB*-02, *P4HB*-021 and *P4HB*-027) in 30 different tissues. Among these isoforms, *P4HB*-02 and *P4HB*-027 displayed slightly higher expression when compared to *P4HB*-021. The fractional expression of variant *P4HB*-021 in heart was higher compared to other tissues. For Fig. 4, we analyzed the 30 tissues by merging all sub-regions of each tissue. In Fig. S3, we separately analyzed isoform expression in different sub-regions of heart (atrial appendage and left ventricle), showing no difference in isoform expression. In this specific subset, *P4HB*-027, *P4HB*-021 and *P4HB*-02 were represented in arterial cells (aorta, coronary and tibial), with slightly higher prevalence of *P4HB*-027.

### *P4HB* splice variants are highly expressed in smooth muscle cell

**Fig. 5:**
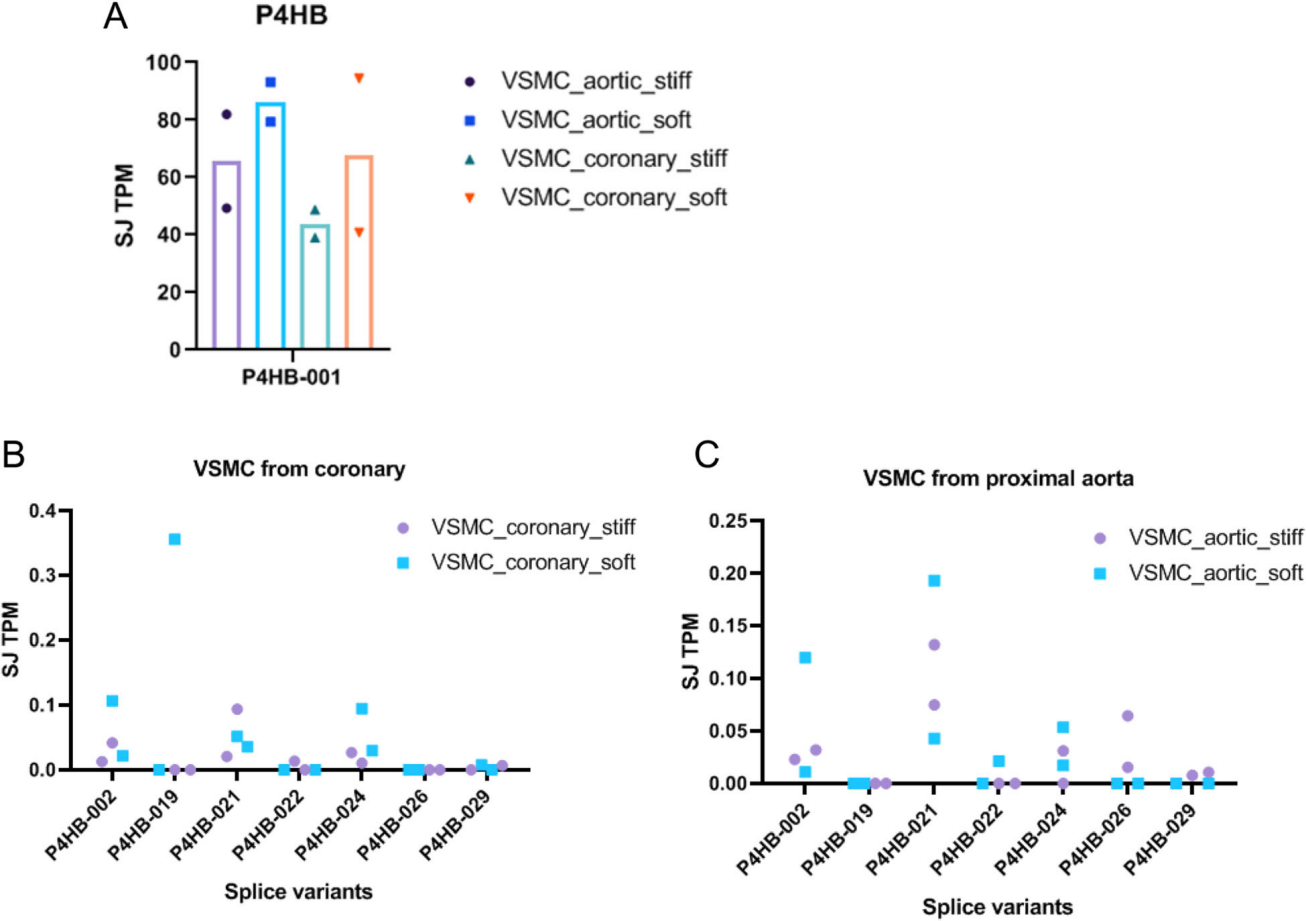
Analysis of RNA-seq data from a study [31] using VSMC (vascular smooth muscle cells) mimicking pathologic (stiff) and physiologic conditions (soft). **a** *P4HB* (*P4HB*-001) gene expression. Also shown is the expression of *P4HB* and its splice variants in (**b**) VSMC from coronary artery and (**c**) VSMC of proximal aorta

Given our focus on vascular cells and the above results from the FANTOM5 and ENCODE analysis, we further pursued the *P4HB* isoform analysis in these cell types. A recently published study [31] produced RNA-seq data from human aortic and coronary vascular smooth muscle cells (VSMC) aiming to investigate gene expression patterns during changes in extracellular matrix stiffness, since VSMC-extracellular matrix mechanobiological interactions are involved in disease pathogenesis. Figure 5 indicates that *P4HB* expression tended to be lower under pathologic, as compared with physiologic conditions. Concerning *P4HB* splice variants in coronary artery VSMC, the splice junction TPM tended to be higher in physiologic conditions. Similarly, in VSMC from proximal aorta isoforms *P4HB*-02 and *P4HB*-021 were more representative in physiological conditions. Taken together, these data indicate that the expression of specific isoforms was specific for each cell type, e.g., endothelial cells (Fig. 3) vs. VSMC and even between distinct VSMC locations (Fig. 4).

### Validation analysis of selected *P4HB* splice variants in cells

We next performed the validation of *P4HB* splice variant expression in distinct cell types using PCR. For that, we arbitrarily selected 3 isoforms on the basis of their expression levels and tissue specificity (above data), namely *P4HB*-02, *P4HB*-021 and *P4HB*-027.

**Fig. 6:**
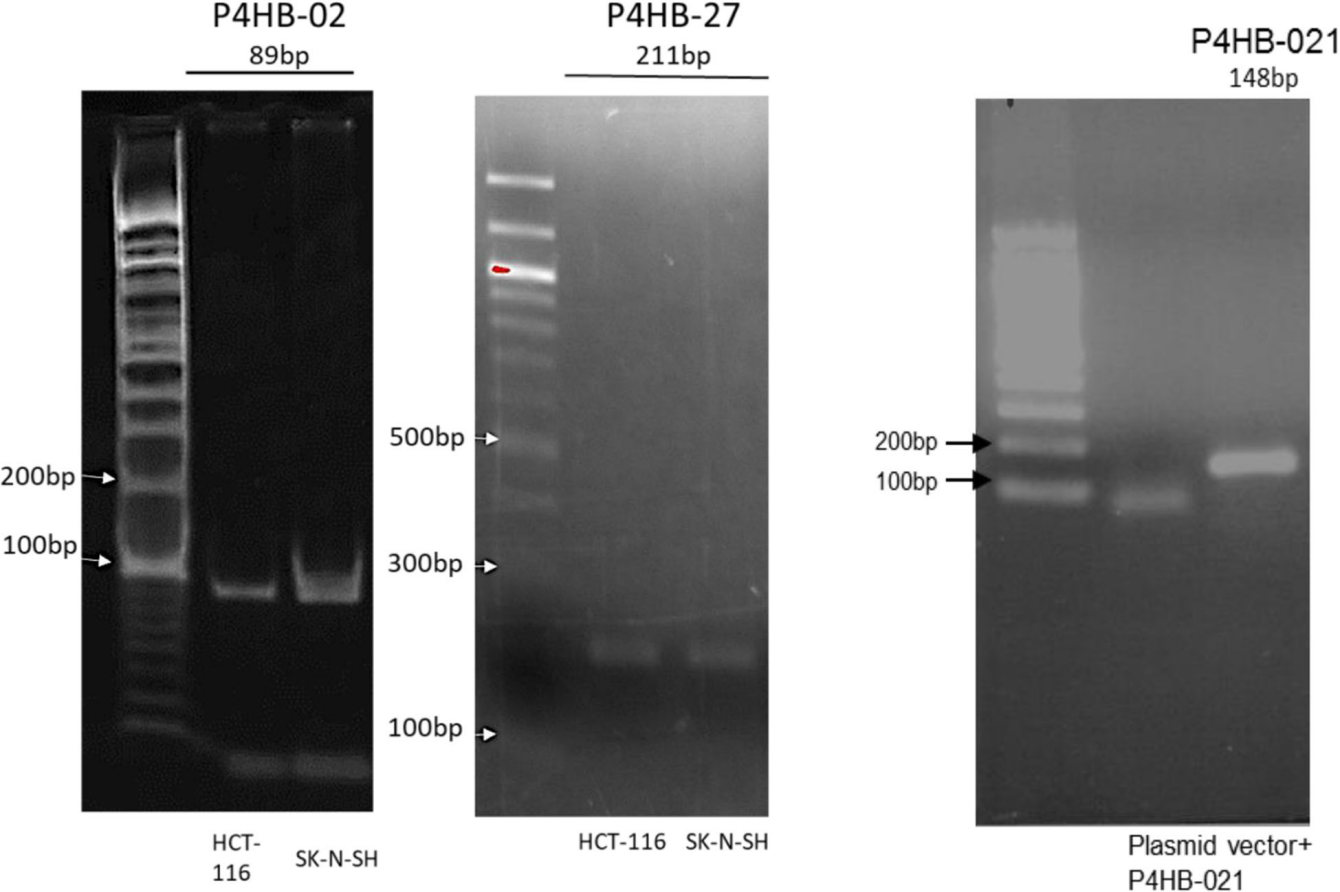
PCR amplification of the splice junction of splice variants *P4HB*-02, *P4HB*-021 and *P4HB*-027. The specific splice junction of *P4HB*-02 (89 bp) and 027 (211 bp) were amplified in HCT-116 cells and SK-N-SH cells. For the amplification of *P4HB*-021, the expected fragment was 148 bp. The amplification of this fragment was performed using a vector pUC57 with the *P4HB*-021 cloned as template to PCR reaction

The cells chosen for an initial overview were neuroblastoma (SK-N-SH) cell line and HCT-116 (human colon cancer) cell line, on the basis of a previous analysis using IGV to detect the splicing junction and the use of shell script to detect the splice junction in BAM files. After RNA extraction and cDNA synthesis, PCR assays using specific sets of primers for each splicing junction were conducted (Fig. 6), resulting in each case in one amplicon, which was purified and cloned in pGEM-T (Promega). The amplicons had 89 bp or 211 bp, respectively for *P4HB*-02 or *P4HB*-027. For *P4HB*-021, the amplicon was cloned in pUC57 vector containing the complete isoform sequence. The three amplicons were cloned, sequenced and the nucleotide sequence with the splice junction was confirmed.

**Fig. 7:**
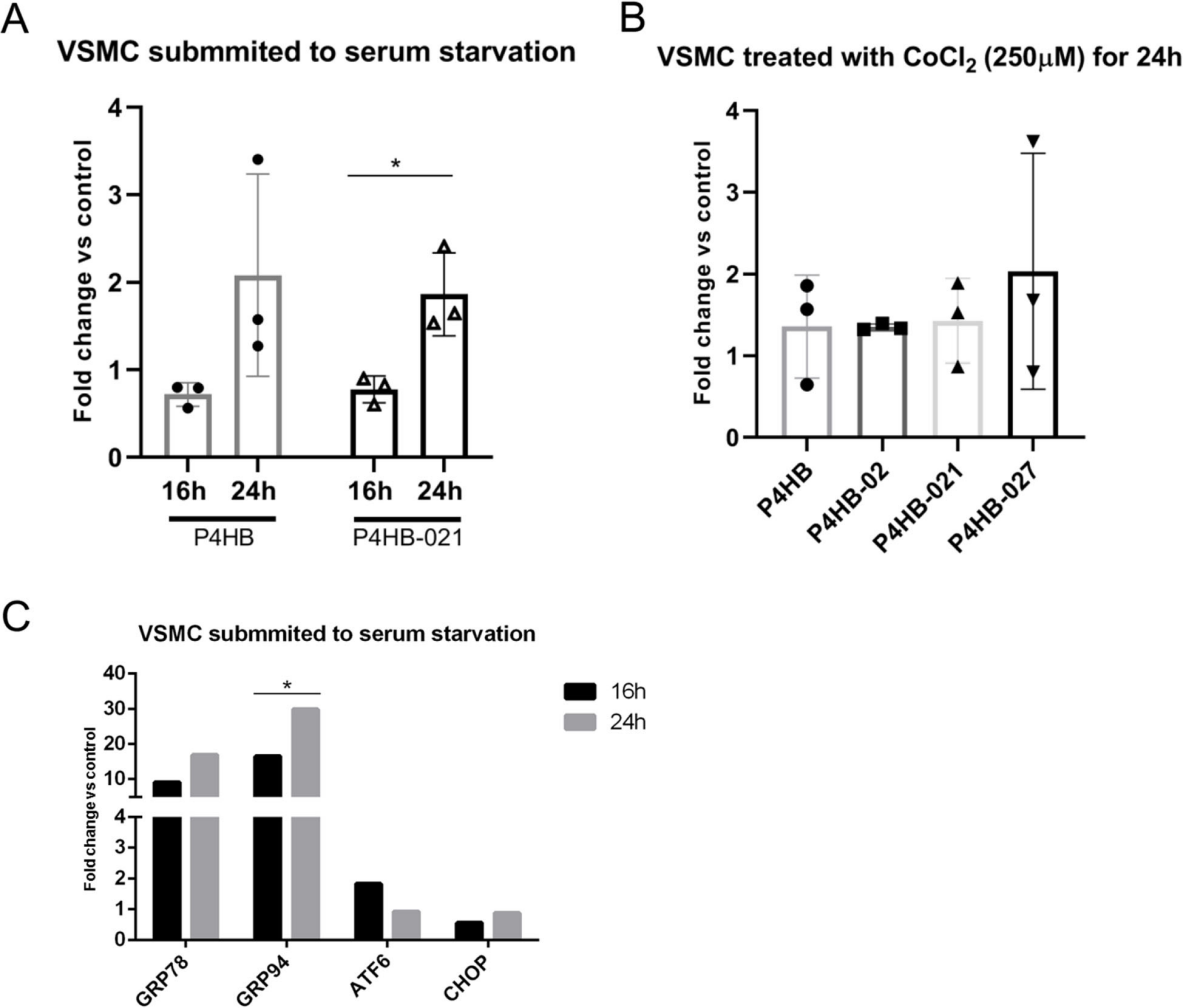
Real-time PCR expression. **a** Expression of *P4HB* gene and *P4HB*-021 in primary VSMC (from human mammary artery) submitted to serum starvation during 16 h and 24 h. **b** Expression of *P4HB* gene and three *P4HB* isoforms in VSMC treated with CoCl_2_. **c** Expression of four ER stress response genes in VSMC submitted to serum starvation during 16 h and 24 h. Results shown as fold change normalized by *β-Actin* and *HPRT* (hypoxanthine guanine phosphoribosyl transferase). Total RNA was used to perform RT-qPCR. * *p* < 0.05

We next pursued the validation of isoform detection using RT-qPCR, focusing on primary human VSMC from mammary artery and HEK-293 (human embryonic kidney) cell line. In both cases, cells were investigated in their basal state and following serum starvation (16 h or 24 h) or exposure to CoCl_2_ as a mimetic of hypoxia, since some PDIs are upregulated by hypoxia [35]. Important, in accordance with previous results from databanks, *P4HB*-021 was detected in VSMC at baseline and upregulated, together with the expression of *P4HB*, after 24 h serum starvation compared with 16 h (Fig. 7a). Exposure to CoCl_2_, however, did not significantly affect the expression of *P4HB* and its variants in the conditions of our experiments (Fig. 7b). Four genes related to ER stress response were also analyzed with serum starvation (Fig. 7c) and CoCl_2_ treatment.

We also assessed the effects of tunicamycin, a potent inhibitor of *N*-linked glycosylation, in HEK-293 cell line to investigate the influence of ensuing endoplasmic reticulum stress in the expression of *P4HB* gene and its splice variants. Cells were incubated for 16 h and 40 h with 3 tunicamycin concentrations and the expression of *P4HB* and its splicing variants analyzed (Fig. S4). Other genes involved in ER stress response, such as *ATF6*, *CHOP*, *GRP78* and *GRP94*, as well as *NOX1*, *NOX2* and *NOX4* were also analyzed (Fig. S5A). *GRP78* and *GRP94* gene expression, which are early and classical ER stress markers, were increased after 16 h and 40 h tunicamycin, with less robust increases of transcription factors *ATF6* and *CHOP*. While *P4HB* gene and its variants tended to increase vs. control (Fig. S4), expressions of *P4HB*-02 and *P4HB-027* (but not of *P4HB* or *P4HB-021*) decreased vs. those of reference gene (Fig. S5A). None of these differences, however, was statistically significant, confirming that *P4HB* gene and its associated splice variants are not per se directly unfolded protein response (UPR)- responsive genes [13]. Similarly to VSMC, exposure to CoCl_2_ for 24 h depicted a slight, but not statistically significant, difference in *P4HB* and *GRP94*.

## Discussion

Alternative splicing greatly expands the profile of proteins coded from a given gene subset, providing an enhanced diversity of protein isoforms and subtypes potentially involved in specific cellular functions. These include subcellular signaling involved in restricted organ functions and particularly in translocation to distinct subcellular locations such as nucleus [36], mitochondria [37] and Golgi apparatus [38]. These processes greatly enhance the potential for adaptability to distinct external conditions [39] but can also contribute to disease pathophysiology through isoform switching [22]. Alternative transcripts can also regulate canonical gene expression, as for example in the case of CTCF gene [40]. Thus, knowledge of the alternatively spliced isoforms of a given gene is crucial to understand the implications of its genetic regulation. Here we provide a comprehensive analysis of the alternative splicing landscape of the *P4HB* gene. Given the important and multiple functions of the *P4HB* gene, investigation of its alternative splicing landscape is particularly relevant. Moreover, the peculiar modular architecture of all PDIs is likely to yield profound differences in protein function with even minor modifications of its specific domains. In parallel, analysis of such spliced variants provides a relevant scenario to understand structure-function relations of PDIA1. In this regard, the array of functional possibilities evoked by the distinct *P4HB* isoform domain architectures include: 1) change in redox functions such as oxidase, reductase or isomerase, related mainly to *a-type* domains; 2) loss of the isomerase function of *P4HB*, which requires all 4 domains, so most PDIA1 alternative splicing isoforms are unlikely to display thiol isomerase activity, with possible exception of *P4HB*-021; 3) change in chaperone function, which is dependent mainly on the presence and integrity of *b-type* domains; 4) alterations in substrate specificity and binding affinity, also dependent mainly on *b-type* domains, but also on the overall protein conformation; 5) change in location, given by the N-terminus peptide signal and C-terminal KDEL sequences, in addition to other location signals. When all such aspects are considered together, most PDIA1 isoforms are unlikely to exert thiol isomerase activity at the ER, with the possible exception of *P4HB*-021. Likely, they may exert other types of activity at distinct subcellular locations, greatly expanding the functional reach of *P4HB* gene products. A recent computational study [41] highlighted differences in model structure and affinity to ligands between canonical *P4HB* and *P4HB*-02 (ENSP00000388117) protein products. *P4HB*-02 had a lower interaction energy with ribostamycin (inhibitor of chaperone-like PDI activity) compared with canonical *P4HB*. This finding supports evidence that *P4HB*-02 isoform displays different activities vs. the canonical protein and may potentially compete for specific targets.

Our results further corroborate that the expression pattern of *P4HB* isoforms is consistent with multiple specific functions, since the expression is distinct among the different cell types and tissues. While such expression levels are generally low, it must be considered that functional consequences could be relevant if the functional change occurs within a specific compartment. Indeed, while expression levels of PDIA1 are quite high at the endoplasmic reticulum, the levels of PDIA1 at the cell-surface or extracellular milieu are < 2% of total PDIA1 levels [11], yet this specific pool displays crucial functions related to thrombosis, viral infection and vascular remodeling, among others [5].

**Fig. 8:**
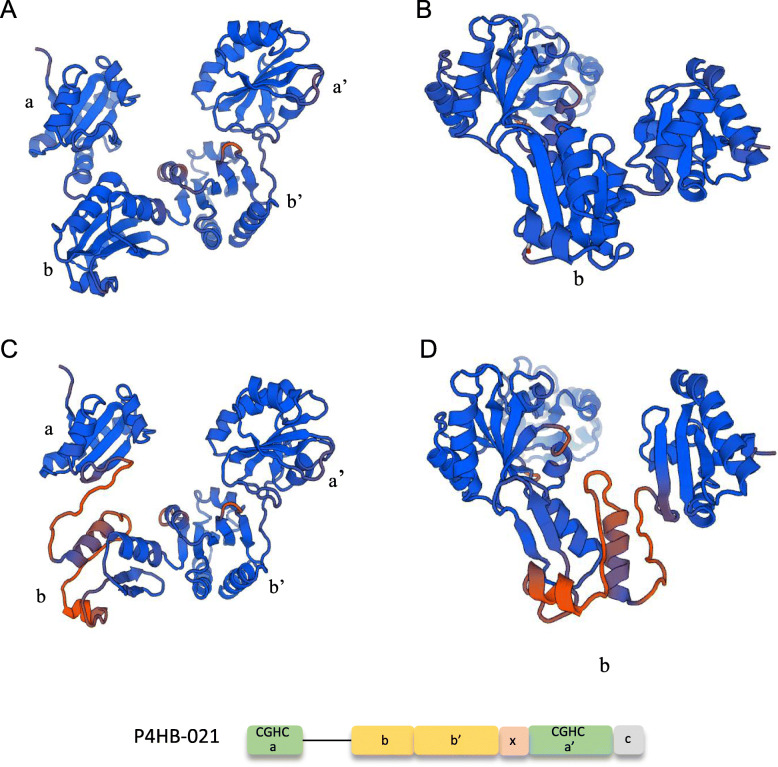
Predicted protein modeling of *P4HB*-021. (**A-B**) Protein domain organization of PDIA1 with indication of the four domains: *a, b, b’, a’*. (**C-D**) *P4HB*-021 splice variant shows a different conformation, particularly in the *b* domain, as compared to PDIA1. The model was predicted using the UniProt sequence and the SWISS Model (https://swissmodel.expasy.org). The absence of a 44-residue stretch at the transition between *a* and *b* domains associates with a predicted unstructured architecture, which might confer additional mobility in a way analogous to that provided by the *x*-linker between the *b’* and *a’* domains

In particular, *P4HB*-021 was significantly expressed in VSMC, however with a variable pattern across distinct VSMC locations. This different expression likely reflects factors such as diverse embryonic origins, distinct mechanobiological histories and variable exposure to paracrine mediators from endothelial cells or interactions with extracellular matrix. *P4HB*-021 depicts truncation of a 44-amino acid stretch at the transition between *a* and *b* domains. To get further insight into potential structure-function implications of this isoform, we modeled its structure using an automated protein homology server. Figure 8 depicts the domain structures of PDIA1 (A-B) and the predicted *P4HB*-021 protein product (C-D), drawn with SWISS-MODEL (https://swissmodel.expasy.org) using UniProt sequences. The absence of exon 3 at the *a* and *b* domain transition of *P4HB*-021 promotes the absence of one α-helix in *a* domain, two β-sheets and one α-helix in *b* domain and one α-helix in *b’* domain. These changes result in a partially unstructured stretch at the *a-b* domain transition, which might associate with significant increases in protein mobility, in a way reminiscent of the *x*-linker for the *b’* and *a’* transition. This could account for accommodation of large and/or complex types of substrates not usually accessible to canonical PDIA1. A particular stimulus that upregulated *P4HB*-021 expression was serum starvation, raising the possibility that this variant associates with metabolic-related signaling and cell differentiation.

## Conclusions

Our results indicate novel aspects to consider with respect to PDIA1-mediated effects on functions including NOX NADPH oxidase activation, cell migration, cell differentiation, mechanoregulation and RhoGTPase modulation. In all such processes, PDIA1-mediated effects have been addressed mainly at the protein expression level, given the high expression of PDI proteins in general. The present results provide a basis to further our understanding of how PDIA1-dependent functions may also be potentially modulated by genetic regulation. Future perspectives include the identification of specific functions of spliced variants.

## Methods

### Part I: Bioinformatic studies

#### Databases

The Functional Annotation of Mammalian Genomes 5 (FANTOM5) project covers more than 1000 human and mouse samples which were profiled by CAGE, with most samples derived from primary cells [42]. For this analysis, we used a subset of 70 human samples from FANTOM5 for which RNA-seq data were available [31]. More information about the FANTOM5 samples is available at this link: http://fantom.gsc.riken.jp/5/sstar/Browse_samples.

The RNA-seq libraries from ENCODE repository (https://www.encodeproject.org/) are publicly available. Table S2 details the accession number of each dataset. The first dataset group constitutes six samples from primary cells obtained from five donors. The second is composed by distinct cell lines, including specific cancer cell lines, accounting for 12 different cell types with 27 samples. This dataset was submitted to ENCODE Project by Caltech and Cold Spring Harbor Laboratory (CSHL). The accession number for VSMC RNA-seq data [31] is GSE100081.

For analysis of the Genotype Tissue Expression (GTEx) Project (https://gtexportal.org/home/datasets) database, we downloaded data from 30 different human tissues containing 11,690 samples.

#### Bioinformatic analysis

To determine the landscape of *P4HB* splice variant expression, we investigated RNA-seq data across FANTOM5, ENCODE Project and GTEX database. RNA-seq pipeline analyses to detect splice-junction of *P4HB* gene in FANTOM5, ENCODE and GTEx database were developed. The only difference was that for GTEx database we used the raw data obtained, meaning the splice junction value for each splice variant and normalized by the number of reads of *P4HB* gene. The computational pipeline which identifies the splice junction for one specific gene was made with stringent filters to maximize the specificity for splice-junction.

The development of new methods of transcriptome profiling, with longer read lengths, paired reads and mapping across splice junctions is also required for improvements of data analysis. For this study we develop a pipeline to count the splice junction using BAM files (Binary Alignment/Map format), which is a binary representation of the SAM files (Sequence Alignment/Map format) developed for the 1000 Genomes Project [43, 44] and detect the presence of splicing junctions for each *P4HB* splice variant. The pipeline for mapping the splice junction used in this work counts the specific splice junction for each *P4HB* splice variant described in Fig. 1, divided by the total number of reads multiplied by 1,000,000 and transforming in a unit called Splice Junction Tag per Million (SJ TPM), which is used as an expression level unit. The genome GRCh37 assembly was used as reference genome. The RStudio was used to visualize the expression in SJ TPM.

$$ \mathrm{SJ}\ \mathrm{TPM}=\frac{\mathrm{Number}\ \mathrm{of}\ \mathrm{splice}\mathrm{d}\ \mathrm{reads}\ \mathrm{for}\ \mathrm{splice}\ \mathrm{variants}}{\mathrm{Total}\ \mathrm{number}\ \mathrm{of}\ \mathrm{reads}\ \mathrm{for}\ \mathrm{each}\ \mathrm{dataset}}\ast 1,000,000 $$

#### RNA-seq libraries of human samples

RNA-seq data from FANTOM5, corresponding to 70 samples, includes a diverse set of human biological samples. This library is 100 bp single-end RNA-seq, sequenced at RIKEN GeNAS on an Illumina Hi-Seq2000 platform with a depth of ~ 200 million *reads* each [30].

RNA-seq data from ENCODE (https://genome.ucsc.edu) contains 12 different cell lines: CD20, GM12878, Haoaf, Haoec, Hep G2, HeLa, HSMM, HUVEC, HCT-116, H1hesc, Hmec, Nhek and SK-N-SH, with a total of 27 samples. The 12 samples were selected due the presence of CAGE peaks in the promoter region. These set of data contains *reads* around 75 ~ 200 bp and sequenced on Illumina Genome Analyzer or Illumina Hi-Seq 2000. The RNA-seq from [31], was sequenced in Illumina Hi-Seq 2500, 100 bp paired-end.

For GTEx, RNA-seq data was obtained from https://gtexportal.org/home/datasets. The RNA-seq was performed using the Illumina TruSeq library construction preparation and the sequencing produced 76 bp paired ended *reads*. More information is available at the link https://gtexportal.org/home/documentationPage .

#### CAGE tags

CAGE (Cap Analysis of Gene Expression) is a technology to map the majority of transcription starting sites and their promoters, thus deciphering the expression of the RNAs produced at each promoter [45] This technology allows the acquisition of gene expression profiling, the identification of promoter use and the specific transcriptional start site (TSS). Counting the numbers of CAGE tags for each promoter within a gene, it is possible to determine the expression level and the usage of different promoters. This tag has 27 nucleotides, facilitating the map of these tags in the genome. CAGE peaks were visualized and the data acquired through ZENBU [46].

#### Visualization of alternative splicing in *P4HB* isoforms

To visualize the RNA-seq data we use the Integrative Genomics Viewer (IGV) browser [47], using as input spliced alignments (in BAM files format) and gene model annotation in GFF format [48]. The BAM files are from FANTOM5 RNA-seq data.

### Part II: Cellular studies

#### Cell culture and treatment

HCT-116 and SK-H-SN cells were obtained from the Cell Bank at RIKEN. HCT-116 was maintained in DMEM (Sigma) medium supplemented with 10% FBS and SK-H-SK cells were maintained in MEM (Sigma) supplemented with 10% FBS, 1% penicillin/streptomycin and 1x L-Glutamine MEM medium. RNA extraction was performed by Maxwell® RSC simply RNA Tissue Kit (Promega) and cDNA synthesis by PrimeScript 1st cDNA Synthesis kit (Takara). HEK-293 cells were cultivated in DMEM with high glucose supplemented with 10% FBS and 1% penicillin/streptomycin. Primary vascular smooth muscle cells from human mammary artery were cultivated in DMEM with low glucose with 10% FBS and 1% penicillin/streptomycin. These cells were obtained from a pool of fragments obtained from donors undergoing coronary revascularization [49].

Exposure to 100 μM CoCl_2_ for 10 h and 24 h in HEK-293 and to VSMC 250 μM CoCl_2_ for 24 h was performed to mimic hypoxia. After removal of culture medium, cells were rinsed with phosphate-buffered saline (PBS; pH 7.4) and then collected to RNA extraction. For tunicamycin exposure, we used three different concentrations (0,5 μg/mL, 1 μg/mL and 2 μg/mL) for 16 h and 40 h.

#### PCR for isoforms splice sites

The primers were designed for the isoform *P4HB*-02 (exon5/6 fwd 5′-TCACCGAGCAGAGTGTGTCTG; exon 6/7 rvs 5′-GATGAACAGGATCTTGCCCTTG-3′), *P4HB*-021 (exon 2 fwd 5′ TATCCCACCATCAAGTTCTTCAG 3′; exon 2/4 rvs 5′ CGACTCCACGTCACCTGTATATT 3′) and *P4HB*-027 (exon 2/3 fwd 5′-GAATATACAGCTGCAGAGTCC-3′; exon 4/5 rvs 5′- CTTCATCAAACTTCTTAAAG-3′) in the specific splice junction for each splice variant. For the canonical *P4HB* gene, the primers were: exon 3 fwd 5′- GAGAGGCTGATGACATCGTG-3′; exon 3 rvs 5′-GACTCCACCAAGGACTCTGC-3′. The PCR products were loaded in 2.0% agarose gel to visualize the amplicons.

#### Experimental validation of alternative splicing junction

The fragments were amplified using primers specific for each splicing junction. A 2.0% agarose gel was used to detect the fragment followed by gel staining and photo documentation. PCR products were purified and cloned into pGEM-T vector (Promega). Miniprep was performed to extract the plasmidial DNA. The samples were sequenced through Sanger sequencing at Genetic Diagnosis Technology Unit, IMS at RIKEN. Sequencing primers T7 and SP6 were used.

#### Cloning of novel human *P4HB* splice variants

Two different isoforms and the *P4HB* gene were cloned (GeneScript) in the pUC57 vector. The variants selected were: *P4HB*-02 (825 bp) and *P4HB*-021 (1419 bp). The sequence of *P4HB*-021 as used as template for isoform validation.

#### Real time RT-PCR analysis

Next, we examined the transcript level information using HEK-293 cell line and primary culture of human mammary artery vascular smooth muscle cells (VSMC). Total RNA was isolated from HEK-293 cells and VSMC, using the illustra RNAspin Mini RNA isolation kit (Cat. No. 25–0500-72, GE Healthcare). For cDNA synthesis, we used the Superscript II reverse transcriptase kit (Cat.No.18064–014, Life Technologies). The qPCR reactions were performed with SYBR and the Platinum SYBR Green qPCR SuperMix-UDG kit (Cat. No.11733–033, Life Technologies) and ROX dye, as passive reference.

For quantitative analysis (qPCR) of splice variants [50], we designed a primer set for each of the three isoforms analyzed into the specific splice junction. To avoid co-amplification of other transcripts, the requirement was that the primer overlapped the splice junction at least 8 bases at 3′ end and 5′ end. Amplicon length analysis was performed to confirm the amplification.

Primers to detect the mRNA expression for real time PCR of ER stress and NOX family-related genes were as follows: *GRP78* (fwd 5′-CACAGTGGTGCCTACCAAGAAG-3′; rvs 5′-AGCAGGAGGAATTCCAGTCAGA-3′), *GRP94* (fwd 5′-GCTTCGGTCAGGGTATCTTT-3′; rvs 5′-GGCTCTTCTTCCACCTTTGC-3′), *CHOP* (fwd 5′-AAGGCACTGAGCGTATCATGT-3′; rvs 5′-TGAAGATACACTTCCTTCTTGAAC-3′), *ATF6* (fwd 5′-CCGTATTCTTCAGGGTGCTC-3′, rvs 5-CACTCCCTGAGTTCCTGCTG-3′), *NOX1* (fwd 5′-CTCTCTCCTGGAATGGCA-3′; rvs 5′-GACCATCCACTTCAATCC-3′), *NOX2* (fwd 5′-TGCCTTTGAGTGGTTTGCAGAT-3′; rvs 5′; rvs 5′-ATTGGCCTGAGACTCATCCCA-3′) *NOX4* (fwd 5′-TGTGCCGAACACTCTTGGC; rvs 5′-ACATGCACGCCTGAGAAAATA-3′) For reference genes, we used *β-actin* (fwd 5′-GATGACCCAGATCATGTTTGAGACC-3′; rvs 5′-CGGTGAGGATCTTCATGAGGTAGT-3′) and *HPRT* (fwd 5′-CGTCTTGCTCGAGATGTGATG-3′; rvs 5′-GCACACAGAGGGCTACAATGTG-3′).

#### Statistical analysis

The results are described as mean and standard deviation. Comparison between two groups was performed by Student t-test. GraphPad Prism 6.0 (GraphPad software, San Diego, CA, USA) was used for statistical analyses, adopting a 0.05 significance level.

## Supplementary Information

**Additional file 1: Table S1.** *P4HB* gene and splice variant information.

**Additional file 2: Table S2.** Accession numbers of ENCODE Files.

**Additional file 3: Table S3.** Table with information about GTEx RNA-seq data is presented as dataset.

**Additional file 4: Figure S1.** Top CAGE peaks in TPM (tags per million) for FANTOM CAGE samples (A) and ENCODE CAGE samples. These graphs represent the samples with highest TPM.

**Additional file 5: Figure S2.** Representative sashimi plots of 10 FANTOM5 samples showing 4 different cell lines for the region chr17: 79796651–79,822,949 obtained using sashimi-plot utility in IGV program. (A) The plot presents the entire *P4HB* gene with 11 exons in the bottom (blue). The lines indicate exon 3, with the specific junction for *P4HB*-027. The black arrow indicates the exon 3 and the splice junction of this isoform.

**Additional file 6: Figure S3.** Quantification of *P4HB* splice variants to detect the fraction of isoform abundance normalized by *P4HB* gene. (A) Fraction of *P4HB*-02, *P4HB*-021 and *P4HB*-027 in blood vessels of three subtypes: aorta (*n* = 299), coronary artery (*n* = 172) and tibial artery (*n* = 400) (B) Fraction of *P4HB* splice variants in heart with two sub-regions: atrial appendage (*n* = 300) and left ventricle (*n* = 300).

**Additional file 7: Figure S4.** Expression of *P4HB* gene and variants *P4HB-*02, *P4HB-*021 and *P4HB-*027. (A) *P4HB* and splice variant expression after exposure to tunicamycin for 16 h and (B) 40 h in HEK-293 cell line treated with tunicamycin (0.5 μg/mL, 1 μg/mL and 2 μg/mL). Results shown as fold change versus control sample (without tunicamycin treatment). Total RNA was used to perform RT-qPCR.

**Additional file 8: Figure S5.** Real-time PCR expression profiling of 8 genes and 3 *P4HB* splice variants in HEK-293 cells (A) exposed to tunicamycin (0.5 μg/mL, 1.0 μg/mL and 2.0 μg/mL) or (B) to CoCl_2_ for 10 h and 24 h. The heatmap was generated by a log transformation of the real-time PCR data presented as ∆Ct (C_T_ gene of interest – C_T_ reference gene).

## Data Availability

The Genotype-Tissue Expression (GTEx) Project was supported by the Common Fund of the Office of the Director of the National Institutes of Health, and by NCI, NHGRI, NHLBI, NIDA, NIMH, and NINDS. The data used for the analyses described in this manuscript were obtained from GTEx Portal on 09/05/2018. CAGE data and RNA-seq data sample information are available through the FANTOM5 resource browser at http://fantom.gsc.riken.jp/5/sstar/Browse_samples. We downloaded the datasets from ENCODE (https://www.encodeproject.org) the accession number were listed in Table S2 in supplementary data. All data generated or analysed during this study are included in this published article [and its supplementary information files].

## Abbreviations

BAM: Binary alignment map
CAGE: Cap analysis gene expression
ER: Endoplasmic reticulum
FANTOM5: Functional Annotation of the Mouse/Mammalian Genome
Gm12878: Human lymphoblastoid cell line
GTEx: Genotype Tissue Expression
HaoEC: Human aortic endothelial cells
HaoAF: Human aortic adventitial fibroblasts
HCT-116: Human colon cancer cell line
Hmsc: Human mesenchymal stem cell line
Hsmm: Human skeletal muscle myoblast cell line
HUVEC: Human umbilical vein endothelial cell
KDEL: Lys-Asp-Glu-Leu ER retrieval motif
PDI: Protein disulfide isomerase
RNA-seq: RNA sequencing
SK-N-SH: Neuroblastoma cell line
SAM: Sequence alignment map
TPM: Tags per million
VSMC: Vascular smooth muscle cell
UPR: Unfolded protein response
UTR: Untranslated region
