# Supplementary material for: Analysis of splice variants of the human protein disulfide isomerase (P4HB) gene
Source: BMC Genomics. 2020 Nov 4;21:766. doi: 10.1186/s12864-020-07164-y (PMC7640458; doi:10.1186/s12864-020-07164-y)
Supplement: Supplementary file 1 — Additional file 1: Table S1. P4HB gene and splice variant information. [file 12864_2020_7164_MOESM1_ESM.docx]

**SUPPLEMENTARY INFORMATION**

**Analysis of Splice variants of the Human Protein Disulfide Isomerase (*P4HB*) Gene**

Daniela Kajihara ^1,3^

Chung-Chau Hon ^2^

Aimi Naim Abdullah ^3^

João Wosniak Jr. ^1^

Ana Iochabel S. Moretti ^1^

Joice F. Poloni ^4^

Diego Bonatto ^4^

Kosuke Hashimoto ^3 *^

Piero Carninci ^3 *^

Francisco R. M. Laurindo ^1 *^

**SUPPLEMENTARY INFORMATION CONTAINS:**

**1) Tables S1 and S2**

**2) Figures S1 to S4**

**SUPPLEMENTARY TABLES**

**Table S1. - *P4HB* gene and splice variant information**

| **Table 1. Human *P4HB* splice variants** | | | | | | |  |
| --- | --- | --- | --- | --- | --- | --- | --- |
| **Gene/Splice variant** | **Transcipt ID** | **Length (bp)** | **UniProt** | **Length (aa)** | **Molecular mass (kDa)** | **Signal peptide (prediction)** | |
| ***P4HB*-001** | **ENST00000331483.4** | **2603bp** | **A0A024R8S5 / P07237** | **508** | **57.1** | **Yes** | |
| *P4HB*-002 | ENST00000415593.1 | 1669bp | H0Y3Z3 | 274 | 31.4 | No | |
| *P4HB*-019 | ENST00000574914.1 | 571bp | I3L3P5 | 156 | 16.9 | Yes | |
| *P4HB*-021 | ENST00000439918.2 | 1502bp | H7BZ94 | 464 | 52.5 | Yes | |
| *P4HB*-022 | ENST00000573778.2 | 688bp | I3L312 | 208 | 23 | Yes | |
| *P4HB*-023 | ENST00000576390.1 | 439bp | I3L514 | 106 | 11.6 | Yes | |
| *P4HB*-024 | ENST00000576052.1 | 562bp | I3NI03 | 166 | 18.5 | Yes | |
| *P4HB*-025 | ENST00000571617.1 | 505bp | I3L0S0 | 148 | 16.2 | Yes | |
| *P4HB*-026 | ENST00000576380.1 | 943bp | I3L398 | 200 | 23 | No | |
| *P4HB*-027 | ENST00000576541.1 | 571bp | I3L3U6 | 188 | 20.7 | Yes | |
| *P4HB*-029 | ENST00000570907.1 | 353bp | I3L4M2 | 117 | 13.5 | No | |
