# Supplementary material for: Analysis of splice variants of the human protein disulfide isomerase (P4HB) gene
Source: BMC Genomics. 2020 Nov 4;21:766. doi: 10.1186/s12864-020-07164-y (PMC7640458; doi:10.1186/s12864-020-07164-y)
Supplement: Supplementary file 2 — Additional file 2: Table S2. Accession numbers of ENCODE Files. [file 12864_2020_7164_MOESM2_ESM.docx]

**Table S2 – Accession numbers of ENCODE Files**

| **Table 2 - GEO accession number** |  |
| --- | --- |
| Source name | GEO ID |
| Homo sapiens smooth muscle of the pulmonary artery primary cell male adult (26 years) (2 biological replicates) | GSM2072330 |
| Homo sapiens smooth muscle of the pulmonary artery primary cell male adult (28 years) (2 biological replicates) | GSM2072331 |
| wgEncodeCaltechRnaSeqGm12878R2x75Il200AlignsRep2V2 | GSM958728 |
| wgEncodeCaltechRnaSeqHct116R2x75Il200AlignsRep1V2 | GSM958749 |
| wgEncodeCaltechRnaSeqHct116R2x75Il200AlignsRep2V2 | GSM958749 |
| wgEncodeCaltechRnaSeqHsmmR2x75Il200AlignsRep2V2 | GSM958744 |
| wgEncodeCshlLongRnaSeqHepg2CytosolPapAlnRep1 | GSM758576 |
| wgEncodeCshlLongRnaSeqHuvecCellPapAlnRep1 | GSM758563 |
| wgEncodeCshlLongRnaSeqHuvecCytosolPapAlnRep3 | GSM758569 |
| wgEncodeCshlLongRnaSeqHuvecCytosolPapAlnRep4 | GSM758569 |
| wgEncodeCshlLongRnaSeqHuvecNucleusLongnonpolyaAlnRep3 | GSM767857 |
| wgEncodeCshlLongRnaSeqHuvecNucleusPapAlnRep4 | GSM758565 |
| wgEncodeCshlLongRnaSeqCd20CellPapAlnRep1 | GSM981256 |
| wgEncodeCshlLongRnaSeqGm12878CellLongnonpolyaAlnRep2 | GSM758572 |
| wgEncodeCshlLongRnaSeqGm12878CytosolPapAlnRep1 | GSM758560 |
| wgEncodeCshlLongRnaSeqGm12878CytosolPapAlnRep2 | GSM758560 |
| wgEncodeCshlLongRnaSeqH1hescNucleusPapAlnRep2 | GSM758574 |
| wgEncodeCshlLongRnaSeqHaoaf609010111CellTotalAlnRep2 | GSM984614 |
| wgEncodeCshlLongRnaSeqHaoec70717061CellTotalAlnRep1 | GSM984618 |
| wgEncodeCshlLongRnaSeqHaoecCellTotalAlnRep2 | GSM984618 |
| wgEncodeCshlLongRnaSeqHepg2CellLongnonpolyaAlnRep1 | GSM758567 |
| wgEncodeCshlLongRnaSeqHepg2CellLongnonpolyaAlnRep2 | GSM758567 |
| wgEncodeCshlLongRnaSeqHepg2CytosolLongnonpolyaAlnRep2 | GSM767840 |
| wgEncodeCshlLongRnaSeqHepg2CytosolPapAlnRep1 | GSM758576 |
| wgEncodeCshlLongRnaSeqHmscuc00525017CellTotalAlnRep1 | GSM984607 |
| wgEncodeCshlLongRnaSeqHmscuc00811017CellTotalAlnRep2 | GSM984607 |
| wgEncodeCshlLongRnaSeqNhekCellLongnonpolyaAlnRep1 | GSM765398 |
| wgEncodeCshlLongRnaSeqSknshCellPapAlnRep3 | GSM981253 |
| wgEncodeCshlLongRnaSeqSknshCellPapAlnRep4 | GSM981253 |
