# Supplementary material for: Analysis of splice variants of the human protein disulfide isomerase (P4HB) gene
Source: BMC Genomics. 2020 Nov 4;21:766. doi: 10.1186/s12864-020-07164-y (PMC7640458; doi:10.1186/s12864-020-07164-y)
Supplement: Supplementary file 3 — Additional file 3: Table S3. Table with information about GTEx RNA-seq data is presented as dataset. [file 12864_2020_7164_MOESM3_ESM.pdf]

Sample description	Sample	Sample ID
Adipose - Subcutaneous	Adipose Tissue	GTEX-1117F-0226-SM-5GZZ7
Adipose - Subcutaneous	Adipose Tissue	GTEX-111CU-1826-SM-5GZYN
Adipose - Subcutaneous	Adipose Tissue	GTEX-111FC-0226-SM-5N9B8
Adipose - Subcutaneous	Adipose Tissue	GTEX-111VG-2326-SM-5N9BK
Adipose - Subcutaneous	Adipose Tissue	GTEX-111YS-2426-SM-5GZZQ
Adipose - Subcutaneous	Adipose Tissue	GTEX-1122O-2026-SM-5NQ91
Adipose - Subcutaneous	Adipose Tissue	GTEX-1128S-2126-SM-5H12U
Adipose - Subcutaneous	Adipose Tissue	GTEX-113IC-0226-SM-5HL5C
Adipose - Subcutaneous	Adipose Tissue	GTEX-117YX-2226-SM-5EGJJ
Adipose - Subcutaneous	Adipose Tissue	GTEX-11DXW-0326-SM-5H11W
Adipose - Subcutaneous	Adipose Tissue	GTEX-11DXX-2326-SM-5Q5A2
Adipose - Subcutaneous	Adipose Tissue	GTEX-11DZ1-0226-SM-5A5KF
Adipose - Subcutaneous	Adipose Tissue	GTEX-11EI6-0226-SM-5EQ64
Adipose - Subcutaneous	Adipose Tissue	GTEX-11EM3-2326-SM-5H12B
Adipose - Subcutaneous	Adipose Tissue	GTEX-11EMC-2826-SM-5PNY6
Adipose - Subcutaneous	Adipose Tissue	GTEX-11EQ8-0226-SM-5EQ5G
Adipose - Subcutaneous	Adipose Tissue	GTEX-11EQ9-2526-SM-5HL66
Adipose - Subcutaneous	Adipose Tissue	GTEX-11GS4-2626-SM-5A5LD
Adipose - Subcutaneous	Adipose Tissue	GTEX-11GSO-2326-SM-5A5LX
Adipose - Subcutaneous	Adipose Tissue	GTEX-11H98-0226-SM-5NQ89
Adipose - Subcutaneous	Adipose Tissue	GTEX-11I78-2626-SM-5Q5AI
Adipose - Subcutaneous	Adipose Tissue	GTEX-11LCK-1126-SM-5A5KC
Adipose - Subcutaneous	Adipose Tissue	GTEX-11O72-0226-SM-59869
Adipose - Subcutaneous	Adipose Tissue	GTEX-11OF3-2426-SM-5Q5AS
Adipose - Subcutaneous	Adipose Tissue	GTEX-11ONC-2526-SM-5986W
Adipose - Subcutaneous	Adipose Tissue	GTEX-11P7K-2026-SM-5GU74
Adipose - Subcutaneous	Adipose Tissue	GTEX-11P81-2426-SM-5GU65
Adipose - Subcutaneous	Adipose Tissue	GTEX-11P82-1726-SM-5Q5AT
Adipose - Subcutaneous	Adipose Tissue	GTEX-11PRG-0626-SM-5BC56
Adipose - Subcutaneous	Adipose Tissue	GTEX-11TT1-2426-SM-5EQMK
Adipose - Subcutaneous	Adipose Tissue	GTEX-11TTK-0226-SM-5N9EC
Adipose - Subcutaneous	Adipose Tissue	GTEX-11TUW-2626-SM-5EQKZ
Adipose - Subcutaneous	Adipose Tissue	GTEX-11UD1-0226-SM-5EQKL
Adipose - Subcutaneous	Adipose Tissue	GTEX-11UD2-0126-SM-5EQL2
Adipose - Subcutaneous	Adipose Tissue	GTEX-11WQC-2426-SM-5EQKQ
Adipose - Subcutaneous	Adipose Tissue	GTEX-11XUK-2126-SM-5EQLR
Adipose - Subcutaneous	Adipose Tissue	GTEX-11ZTS-0226-SM-5EQKD
Adipose - Subcutaneous	Adipose Tissue	GTEX-11ZTT-2526-SM-5EQM9
Adipose - Subcutaneous	Adipose Tissue	GTEX-11ZU8-2426-SM-5EQMV
Adipose - Subcutaneous	Adipose Tissue	GTEX-11ZUS-0726-SM-59886
Adipose - Subcutaneous	Adipose Tissue	GTEX-11ZVC-2626-SM-5FQTA
Adipose - Subcutaneous	Adipose Tissue	GTEX-1211K-2226-SM-5FQU6
Adipose - Subcutaneous	Adipose Tissue	GTEX-12126-0426-SM-5Q5AP
Adipose - Subcutaneous	Adipose Tissue	GTEX-1212Z-2426-SM-5EQ5D
Adipose - Subcutaneous	Adipose Tissue	GTEX-12584-0226-SM-5EQL5
Adipose - Subcutaneous	Adipose Tissue	GTEX-12696-2526-SM-5EQLN
Adipose - Subcutaneous	Adipose Tissue	GTEX-1269C-2726-SM-5EGJ4
Adipose - Subcutaneous	Adipose Tissue	GTEX-12C56-1626-SM-5FQUO
Adipose - Subcutaneous	Adipose Tissue	GTEX-12KS4-0426-SM-5EQMC
Adipose - Subcutaneous	Adipose Tissue	GTEX-12WSA-0326-SM-5BC6I
Adipose - Subcutaneous	Adipose Tissue	GTEX-12WSC-0226-SM-5EQ52
Adipose - Subcutaneous	Adipose Tissue	GTEX-12WSD-0226-SM-59HKO
Adipose - Subcutaneous	Adipose Tissue	GTEX-12WSL-2426-SM-5GCN7

Adipose - Subcutaneous	Adipose Tissue	GTEX-12WSM-0226-SM-5LZV6
Adipose - Subcutaneous	Adipose Tissue	GTEX-12ZZX-0226-SM-5DUXU
Adipose - Subcutaneous	Adipose Tissue	GTEX-12ZZY-0326-SM-5LZVQ
Adipose - Subcutaneous	Adipose Tissue	GTEX-12ZZZ-0526-SM-5DUX6
Adipose - Subcutaneous	Adipose Tissue	GTEX-13111-1726-SM-5EGJZ
Adipose - Subcutaneous	Adipose Tissue	GTEX-13112-2726-SM-5DUW5
Adipose - Subcutaneous	Adipose Tissue	GTEX-13113-1826-SM-5LZW4
Adipose - Subcutaneous	Adipose Tissue	GTEX-1313W-0226-SM-5LZV7
Adipose - Subcutaneous	Adipose Tissue	GTEX-1314G-1626-SM-5EQ67
Adipose - Subcutaneous	Adipose Tissue	GTEX-131XE-2426-SM-5EQ5V
Adipose - Subcutaneous	Adipose Tissue	GTEX-131XF-2226-SM-5EQKG
Adipose - Subcutaneous	Adipose Tissue	GTEX-131XW-0526-SM-5PNXZ
Adipose - Subcutaneous	Adipose Tissue	GTEX-131YS-0226-SM-5IJEH
Adipose - Subcutaneous	Adipose Tissue	GTEX-132AR-0226-SM-5IJBE
Adipose - Subcutaneous	Adipose Tissue	GTEX-132QS-2526-SM-62LFJ
Adipose - Subcutaneous	Adipose Tissue	GTEX-133LE-1926-SM-5N9FV
Adipose - Subcutaneous	Adipose Tissue	GTEX-1399R-2626-SM-5KLZ7
Adipose - Subcutaneous	Adipose Tissue	GTEX-1399T-2726-SM-5K7XK
Adipose - Subcutaneous	Adipose Tissue	GTEX-1399U-2426-SM-5K7XB
Adipose - Subcutaneous	Adipose Tissue	GTEX-139D8-0326-SM-5IJCJ
Adipose - Subcutaneous	Adipose Tissue	GTEX-139T4-0126-SM-5HL5G
Adipose - Subcutaneous	Adipose Tissue	GTEX-139T6-2126-SM-5KM37
Adipose - Subcutaneous	Adipose Tissue	GTEX-139T8-0226-SM-5L3EA
Adipose - Subcutaneous	Adipose Tissue	GTEX-139TT-0226-SM-5K7YH
Adipose - Subcutaneous	Adipose Tissue	GTEX-139TU-0226-SM-5J1NM
Adipose - Subcutaneous	Adipose Tissue	GTEX-139YR-2426-SM-5J1OB
Adipose - Subcutaneous	Adipose Tissue	GTEX-13CF2-2626-SM-5LZZA
Adipose - Subcutaneous	Adipose Tissue	GTEX-13CF3-2326-SM-5IFGL
Adipose - Subcutaneous	Adipose Tissue	GTEX-13D11-2326-SM-5IJCY
Adipose - Subcutaneous	Adipose Tissue	GTEX-13FH7-2226-SM-5IJD4
Adipose - Subcutaneous	Adipose Tissue	GTEX-13FHO-0326-SM-5IJBH
Adipose - Subcutaneous	Adipose Tissue	GTEX-13FHP-0226-SM-5K7WD
Adipose - Subcutaneous	Adipose Tissue	GTEX-13FTW-2526-SM-5IJC8
Adipose - Subcutaneous	Adipose Tissue	GTEX-13FTY-0626-SM-5L3F8
Adipose - Subcutaneous	Adipose Tissue	GTEX-13G51-2626-SM-5LZYW
Adipose - Subcutaneous	Adipose Tissue	GTEX-13IVO-0226-SM-5LZXU
Adipose - Subcutaneous	Adipose Tissue	GTEX-13JUV-2826-SM-5LZWU
Adipose - Subcutaneous	Adipose Tissue	GTEX-13JVG-0226-SM-5J1MW
Adipose - Subcutaneous	Adipose Tissue	GTEX-13N11-2626-SM-5K7UQ
Adipose - Subcutaneous	Adipose Tissue	GTEX-13N1W-0226-SM-5K7W6
Adipose - Subcutaneous	Adipose Tissue	GTEX-13N2G-2726-SM-5J1MG
Adipose - Subcutaneous	Adipose Tissue	GTEX-13NYS-0226-SM-5MR49
Adipose - Subcutaneous	Adipose Tissue	GTEX-13NZ8-0926-SM-5MR3U
Adipose - Subcutaneous	Adipose Tissue	GTEX-13NZ9-0526-SM-5J1OX
Adipose - Subcutaneous	Adipose Tissue	GTEX-13NZA-0126-SM-5K7UH
Adipose - Subcutaneous	Adipose Tissue	GTEX-13NZB-2426-SM-5K7UF
Adipose - Subcutaneous	Adipose Tissue	GTEX-13O1R-0226-SM-5K7U5
Adipose - Subcutaneous	Adipose Tissue	GTEX-13O21-1526-SM-5K7W4
Adipose - Subcutaneous	Adipose Tissue	GTEX-13O3P-0226-SM-5KM3Z
Adipose - Subcutaneous	Adipose Tissue	GTEX-13O3Q-2326-SM-5KM3G
Adipose - Subcutaneous	Adipose Tissue	GTEX-13O61-2426-SM-5J1NV
Adipose - Subcutaneous	Adipose Tissue	GTEX-13OVI-1326-SM-5IJBO
Adipose - Subcutaneous	Adipose Tissue	GTEX-13OVJ-0326-SM-5L3GL
Adipose - Subcutaneous	Adipose Tissue	GTEX-13OVK-2126-SM-6PAN1

Adipose - Subcutaneous	Adipose Tissue	GTEX-13OVL-0326-SM-5IJCS
Adipose - Subcutaneous	Adipose Tissue	GTEX-13OW6-0526-SM-5L3HY
Adipose - Subcutaneous	Adipose Tissue	GTEX-13OW7-0226-SM-5MR3N
Adipose - Subcutaneous	Adipose Tissue	GTEX-13OW8-1226-SM-5K7XD
Adipose - Subcutaneous	Adipose Tissue	GTEX-13PDP-0226-SM-5K7W9
Adipose - Subcutaneous	Adipose Tissue	GTEX-13PLJ-0126-SM-5L3HU
Adipose - Subcutaneous	Adipose Tissue	GTEX-13PVQ-0226-SM-5SIBG
Adipose - Subcutaneous	Adipose Tissue	GTEX-13PVR-2426-SM-5RQHN
Adipose - Subcutaneous	Adipose Tissue	GTEX-13QBU-2526-SM-5LU4X
Adipose - Subcutaneous	Adipose Tissue	GTEX-13QJ3-0426-SM-5RQHP
Adipose - Subcutaneous	Adipose Tissue	GTEX-13RTJ-2526-SM-5S2Q3
Adipose - Subcutaneous	Adipose Tissue	GTEX-13S7M-0226-SM-5S2UG
Adipose - Subcutaneous	Adipose Tissue	GTEX-13SLW-0226-SM-5S2NA
Adipose - Subcutaneous	Adipose Tissue	GTEX-13SLX-0426-SM-5QGRC
Adipose - Subcutaneous	Adipose Tissue	GTEX-13U4I-0226-SM-5SIB1
Adipose - Subcutaneous	Adipose Tissue	GTEX-13VXT-0226-SM-5LU9Q
Adipose - Subcutaneous	Adipose Tissue	GTEX-13VXU-0226-SM-5SI9R
Adipose - Subcutaneous	Adipose Tissue	GTEX-13W3W-2526-SM-5SI9P
Adipose - Subcutaneous	Adipose Tissue	GTEX-13X6J-0226-SM-7EPGC
Adipose - Subcutaneous	Adipose Tissue	GTEX-13X6K-0226-SM-5QGPB
Adipose - Subcutaneous	Adipose Tissue	GTEX-13YAN-0426-SM-5O9DR
Adipose - Subcutaneous	Adipose Tissue	GTEX-144FL-0226-SM-5Q5D4
Adipose - Subcutaneous	Adipose Tissue	GTEX-144GL-0226-SM-5QGOX
Adipose - Subcutaneous	Adipose Tissue	GTEX-144GM-1926-SM-5LUAN
Adipose - Subcutaneous	Adipose Tissue	GTEX-144GO-1626-SM-7EPG5
Adipose - Subcutaneous	Adipose Tissue	GTEX-145LS-0226-SM-5O9A5
Adipose - Subcutaneous	Adipose Tissue	GTEX-145LT-1826-SM-5QGP4
Adipose - Subcutaneous	Adipose Tissue	GTEX-145ME-1926-SM-5SI9S
Adipose - Subcutaneous	Adipose Tissue	GTEX-145MF-0526-SM-5LUA6
Adipose - Subcutaneous	Adipose Tissue	GTEX-145MG-0126-SM-5TDDA
Adipose - Subcutaneous	Adipose Tissue	GTEX-145MI-0426-SM-5O99V
Adipose - Subcutaneous	Adipose Tissue	GTEX-145MN-2626-SM-5NQAH
Adipose - Subcutaneous	Adipose Tissue	GTEX-145MO-0426-SM-5QGP6
Adipose - Subcutaneous	Adipose Tissue	GTEX-146FH-0226-SM-5QGPQ
Adipose - Subcutaneous	Adipose Tissue	GTEX-146FQ-0226-SM-5NQAL
Adipose - Subcutaneous	Adipose Tissue	GTEX-146FR-1826-SM-5QGPF
Adipose - Subcutaneous	Adipose Tissue	GTEX-14753-0326-SM-5QGQB
Adipose - Subcutaneous	Adipose Tissue	GTEX-147F3-0626-SM-5NQ9I
Adipose - Subcutaneous	Adipose Tissue	GTEX-147GR-0426-SM-5S2OL
Adipose - Subcutaneous	Adipose Tissue	GTEX-147JS-0426-SM-5TDD2
Adipose - Subcutaneous	Adipose Tissue	GTEX-148VI-1726-SM-5S2W9
Adipose - Subcutaneous	Adipose Tissue	GTEX-1497J-2326-SM-5NQBK
Adipose - Subcutaneous	Adipose Tissue	GTEX-14A5H-0526-SM-5TDCN
Adipose - Subcutaneous	Adipose Tissue	GTEX-14ABY-0326-SM-5TDDK
Adipose - Subcutaneous	Adipose Tissue	GTEX-14AS3-2026-SM-5TDD9
Adipose - Subcutaneous	Adipose Tissue	GTEX-14BIL-0226-SM-5SI9E
Adipose - Subcutaneous	Adipose Tissue	GTEX-14BIN-0726-SM-793DQ
Adipose - Subcutaneous	Adipose Tissue	GTEX-14BMU-2126-SM-5S2TS
Adipose - Subcutaneous	Adipose Tissue	GTEX-14BMV-0226-SM-5S2N2
Adipose - Subcutaneous	Adipose Tissue	GTEX-14C39-2526-SM-5S2UH
Adipose - Subcutaneous	Adipose Tissue	GTEX-14C5O-0226-SM-5SI6O
Adipose - Subcutaneous	Adipose Tissue	GTEX-14DAQ-0226-SM-664MQ
Adipose - Subcutaneous	Adipose Tissue	GTEX-14DAR-1926-SM-5S2NQ
Adipose - Subcutaneous	Adipose Tissue	GTEX-14E1K-2126-SM-793DU

Adipose - Subcutaneous	Adipose Tissue	GTEX-14E6C-0226-SM-5S2N3
Adipose - Subcutaneous	Adipose Tissue	GTEX-14E6D-0226-SM-62LDW
Adipose - Subcutaneous	Adipose Tissue	GTEX-14E6E-1926-SM-664N4
Adipose - Subcutaneous	Adipose Tissue	GTEX-14H4A-1026-SM-62LDY
Adipose - Subcutaneous	Adipose Tissue	GTEX-14ICK-0326-SM-6LLIZ
Adipose - Subcutaneous	Adipose Tissue	GTEX-14ICL-2026-SM-62LDN
Adipose - Subcutaneous	Adipose Tissue	GTEX-14JG1-0526-SM-6LLHW
Adipose - Subcutaneous	Adipose Tissue	GTEX-14JG6-2326-SM-6LLH3
Adipose - Subcutaneous	Adipose Tissue	GTEX-14JIY-0526-SM-62LFH
Adipose - Subcutaneous	Adipose Tissue	GTEX-14LLW-0226-SM-62LEL
Adipose - Subcutaneous	Adipose Tissue	GTEX-14PHX-2426-SM-62LEN
Adipose - Subcutaneous	Adipose Tissue	GTEX-14PII-0326-SM-6LLIQ
Adipose - Subcutaneous	Adipose Tissue	GTEX-14PJ2-0226-SM-6LLH4
Adipose - Subcutaneous	Adipose Tissue	GTEX-14PJ3-1926-SM-62LEY
Adipose - Subcutaneous	Adipose Tissue	GTEX-14PJ4-2226-SM-6LLIS
Adipose - Subcutaneous	Adipose Tissue	GTEX-14PJ6-2226-SM-6LLIV
Adipose - Subcutaneous	Adipose Tissue	GTEX-14PJM-0326-SM-62LF2
Adipose - Subcutaneous	Adipose Tissue	GTEX-14PK6-2026-SM-6LLHP
Adipose - Subcutaneous	Adipose Tissue	GTEX-14PKU-2126-SM-7LT8A
Adipose - Subcutaneous	Adipose Tissue	GTEX-14PN3-2526-SM-6EU15
Adipose - Subcutaneous	Adipose Tissue	GTEX-15CHQ-0226-SM-6EU2S
Adipose - Subcutaneous	Adipose Tissue	GTEX-15DCD-0226-SM-6LPKC
Adipose - Subcutaneous	Adipose Tissue	GTEX-15DDE-0226-SM-6LLJ6
Adipose - Subcutaneous	Adipose Tissue	GTEX-15ER7-0426-SM-6PAM3
Adipose - Subcutaneous	Adipose Tissue	GTEX-15ETS-0426-SM-6PANI
Adipose - Subcutaneous	Adipose Tissue	GTEX-15G19-0226-SM-6PAL2
Adipose - Subcutaneous	Adipose Tissue	GTEX-15RIE-2326-SM-7KFSC
Adipose - Subcutaneous	Adipose Tissue	GTEX-15SDE-2426-SM-7KFRC
Adipose - Subcutaneous	Adipose Tissue	GTEX-15UKP-0526-SM-7KFRO
Adipose - Subcutaneous	Adipose Tissue	GTEX-16AAH-2026-SM-7LG5F
Adipose - Subcutaneous	Adipose Tissue	GTEX-16BQI-0526-SM-6PAM8
Adipose - Subcutaneous	Adipose Tissue	GTEX-16GPK-0226-SM-7DUFI
Adipose - Subcutaneous	Adipose Tissue	GTEX-16MT8-0526-SM-6PAM6
Adipose - Subcutaneous	Adipose Tissue	GTEX-16MTA-2126-SM-6PALC
Adipose - Subcutaneous	Adipose Tissue	GTEX-16NGA-1826-SM-7LG5L
Adipose - Subcutaneous	Adipose Tissue	GTEX-16NPX-0326-SM-7EWDI
Adipose - Subcutaneous	Adipose Tissue	GTEX-16XZY-2526-SM-7DHL2
Adipose - Subcutaneous	Adipose Tissue	GTEX-16YQH-0226-SM-6PALD
Adipose - Subcutaneous	Adipose Tissue	GTEX-17EUY-0526-SM-7EWDE
Adipose - Subcutaneous	Adipose Tissue	GTEX-17EVP-0426-SM-79OKG
Adipose - Subcutaneous	Adipose Tissue	GTEX-17F96-1726-SM-7IGMB
Adipose - Subcutaneous	Adipose Tissue	GTEX-17F97-0426-SM-7LT9U
Adipose - Subcutaneous	Adipose Tissue	GTEX-17F98-1126-SM-79OJS
Adipose - Subcutaneous	Adipose Tissue	GTEX-17F9Y-0326-SM-7IGLS
Adipose - Subcutaneous	Adipose Tissue	GTEX-17HG3-2326-SM-79OKH
Adipose - Subcutaneous	Adipose Tissue	GTEX-17HGU-0326-SM-7DUER
Adipose - Subcutaneous	Adipose Tissue	GTEX-17HHE-1926-SM-7EPGW
Adipose - Subcutaneous	Adipose Tissue	GTEX-17HHY-0226-SM-793BQ
Adipose - Subcutaneous	Adipose Tissue	GTEX-17HII-0326-SM-7LG4X
Adipose - Subcutaneous	Adipose Tissue	GTEX-17KNJ-2226-SM-7LG6W
Adipose - Subcutaneous	Adipose Tissue	GTEX-17MF6-0226-SM-7LG4G
Adipose - Subcutaneous	Adipose Tissue	GTEX-17MFQ-1826-SM-7DHLS
Adipose - Subcutaneous	Adipose Tissue	GTEX-183FY-0326-SM-793BY
Adipose - Subcutaneous	Adipose Tissue	GTEX-183WM-0226-SM-7LT8T

Adipose - Subcutaneous	Adipose Tissue	GTEX-18465-0226-SM-7LG5M
Adipose - Subcutaneous	Adipose Tissue	GTEX-18A66-0326-SM-718BE
Adipose - Subcutaneous	Adipose Tissue	GTEX-18A67-0226-SM-7LG67
Adipose - Subcutaneous	Adipose Tissue	GTEX-18A6Q-0226-SM-718AS
Adipose - Subcutaneous	Adipose Tissue	GTEX-18A7A-0326-SM-731AT
Adipose - Subcutaneous	Adipose Tissue	GTEX-18D9A-2026-SM-718BL
Adipose - Subcutaneous	Adipose Tissue	GTEX-18D9U-0726-SM-7LG5J
Adipose - Subcutaneous	Adipose Tissue	GTEX-1A32A-0226-SM-718B2
Adipose - Subcutaneous	Adipose Tissue	GTEX-1A3MV-2126-SM-718BV
Adipose - Subcutaneous	Adipose Tissue	GTEX-1A3MW-0226-SM-7939O
Adipose - Subcutaneous	Adipose Tissue	GTEX-1A3MX-0226-SM-731C4
Adipose - Subcutaneous	Adipose Tissue	GTEX-1A8G7-0226-SM-731AS
Adipose - Subcutaneous	Adipose Tissue	GTEX-1AMEY-1426-SM-7939I
Adipose - Subcutaneous	Adipose Tissue	GTEX-1AMFI-2126-SM-731BT
Adipose - Subcutaneous	Adipose Tissue	GTEX-1AX9J-0326-SM-73KXY
Adipose - Subcutaneous	Adipose Tissue	GTEX-1AX9K-2426-SM-73KZ4
Adipose - Subcutaneous	Adipose Tissue	GTEX-1AYD5-2326-SM-73KXF
Adipose - Subcutaneous	Adipose Tissue	GTEX-1B8KE-2526-SM-73KYQ
Adipose - Subcutaneous	Adipose Tissue	GTEX-1B8KZ-1926-SM-7DUG8
Adipose - Subcutaneous	Adipose Tissue	GTEX-1B8SF-0526-SM-7939F
Adipose - Subcutaneous	Adipose Tissue	GTEX-1B8SG-0226-SM-7939Q
Adipose - Subcutaneous	Adipose Tissue	GTEX-1B933-0526-SM-7EPHM
Adipose - Subcutaneous	Adipose Tissue	GTEX-1B97I-1726-SM-7939V
Adipose - Subcutaneous	Adipose Tissue	GTEX-1BAJH-0426-SM-73KWY
Adipose - Subcutaneous	Adipose Tissue	GTEX-1C2JI-2226-SM-7IGPJ
Adipose - Subcutaneous	Adipose Tissue	GTEX-1C475-1926-SM-7DUFV
Adipose - Subcutaneous	Adipose Tissue	GTEX-1C4CL-0426-SM-7939N
Adipose - Subcutaneous	Adipose Tissue	GTEX-1C64O-0226-SM-73KXB
Adipose - Subcutaneous	Adipose Tissue	GTEX-1C6VS-0426-SM-79OJV
Adipose - Subcutaneous	Adipose Tissue	GTEX-1C6WA-0226-SM-7IGPO
Adipose - Subcutaneous	Adipose Tissue	GTEX-1CB4I-0426-SM-7IGPR
Adipose - Subcutaneous	Adipose Tissue	GTEX-1E1VI-0226-SM-793CZ
Adipose - Subcutaneous	Adipose Tissue	GTEX-1EWIQ-0226-SM-793D3
Adipose - Subcutaneous	Adipose Tissue	GTEX-N7MS-0326-SM-4E3K2
Adipose - Subcutaneous	Adipose Tissue	GTEX-NFK9-0326-SM-3MJGV
Adipose - Subcutaneous	Adipose Tissue	GTEX-NL3G-0226-SM-4RGLU
Adipose - Subcutaneous	Adipose Tissue	GTEX-NPJ8-0226-SM-48TBN
Adipose - Subcutaneous	Adipose Tissue	GTEX-O5YT-0226-SM-32PK5
Adipose - Subcutaneous	Adipose Tissue	GTEX-O5YV-0226-SM-48TBY
Adipose - Subcutaneous	Adipose Tissue	GTEX-OHPK-0226-SM-3MJH6
Adipose - Subcutaneous	Adipose Tissue	GTEX-OHPM-0226-SM-3LK61
Adipose - Subcutaneous	Adipose Tissue	GTEX-OHPN-0226-SM-48TBV
Adipose - Subcutaneous	Adipose Tissue	GTEX-OIZH-0226-SM-2YUMH
Adipose - Subcutaneous	Adipose Tissue	GTEX-OIZI-0226-SM-2XCEE
Adipose - Subcutaneous	Adipose Tissue	GTEX-OOBJ-0226-SM-2YUMM
Adipose - Subcutaneous	Adipose Tissue	GTEX-OOBK-0226-SM-2YUMF
Adipose - Subcutaneous	Adipose Tissue	GTEX-OXRK-0326-SM-3NB3R
Adipose - Subcutaneous	Adipose Tissue	GTEX-OXRL-0226-SM-3NB18
Adipose - Subcutaneous	Adipose Tissue	GTEX-OXRN-0226-SM-2I5EJ
Adipose - Subcutaneous	Adipose Tissue	GTEX-OXRO-0226-SM-3LK6F
Adipose - Subcutaneous	Adipose Tissue	GTEX-OXRP-0226-SM-3NB14
Adipose - Subcutaneous	Adipose Tissue	GTEX-P44H-0326-SM-2XCES
Adipose - Subcutaneous	Adipose Tissue	GTEX-P4PP-0226-SM-5S2VR
Adipose - Subcutaneous	Adipose Tissue	GTEX-P4PQ-0226-SM-2S1NK

Adipose - Subcutaneous	Adipose Tissue	GTEX-P4QS-0226-SM-3NB1U
Adipose - Subcutaneous	Adipose Tissue	GTEX-P4QT-0226-SM-3LK68
Adipose - Subcutaneous	Adipose Tissue	GTEX-P78B-0226-SM-3NB1Z
Adipose - Subcutaneous	Adipose Tissue	GTEX-PLZ4-0226-SM-5SI7M
Adipose - Subcutaneous	Adipose Tissue	GTEX-PLZ5-1826-SM-3NB22
Adipose - Subcutaneous	Adipose Tissue	GTEX-PLZ6-1326-SM-3NB24
Adipose - Subcutaneous	Adipose Tissue	GTEX-POMQ-2326-SM-5S2VS
Adipose - Subcutaneous	Adipose Tissue	GTEX-POYW-0726-SM-2XCEO
Adipose - Subcutaneous	Adipose Tissue	GTEX-PSDG-0326-SM-48TCP
Adipose - Subcutaneous	Adipose Tissue	GTEX-PWCY-1926-SM-3NB25
Adipose - Subcutaneous	Adipose Tissue	GTEX-PWN1-0226-SM-5SI7O
Adipose - Subcutaneous	Adipose Tissue	GTEX-PX3G-0226-SM-3NB2C
Adipose - Subcutaneous	Adipose Tissue	GTEX-Q2AG-0226-SM-2S1P4
Adipose - Subcutaneous	Adipose Tissue	GTEX-Q2AH-1726-SM-3NB2B
Adipose - Subcutaneous	Adipose Tissue	GTEX-Q2AI-1426-SM-2S1P5
Adipose - Subcutaneous	Adipose Tissue	GTEX-Q734-1826-SM-2I3EL
Adipose - Subcutaneous	Adipose Tissue	GTEX-QCQG-1826-SM-2S1P2
Adipose - Subcutaneous	Adipose Tissue	GTEX-QDT8-0226-SM-32PL4
Adipose - Subcutaneous	Adipose Tissue	GTEX-QDVJ-1826-SM-2S1P3
Adipose - Subcutaneous	Adipose Tissue	GTEX-QEG4-0326-SM-5S2UQ
Adipose - Subcutaneous	Adipose Tissue	GTEX-QEG5-0326-SM-2S1PB
Adipose - Subcutaneous	Adipose Tissue	GTEX-QEL4-0326-SM-3GAE5
Adipose - Subcutaneous	Adipose Tissue	GTEX-QESD-1526-SM-2S1QT
Adipose - Subcutaneous	Adipose Tissue	GTEX-QLQ7-1526-SM-2S1QA
Adipose - Subcutaneous	Adipose Tissue	GTEX-QLQW-1226-SM-2S1Q9
Adipose - Subcutaneous	Adipose Tissue	GTEX-QMRM-1726-SM-2S1QG
Adipose - Subcutaneous	Adipose Tissue	GTEX-QV31-1326-SM-2S1QE
Adipose - Subcutaneous	Adipose Tissue	GTEX-QV44-1825-SM-447CF
Adipose - Subcutaneous	Adipose Tissue	GTEX-R53T-1626-SM-3GAEW
Adipose - Subcutaneous	Adipose Tissue	GTEX-R55C-1626-SM-48FEG
Adipose - Subcutaneous	Adipose Tissue	GTEX-R55D-0326-SM-48FES
Adipose - Subcutaneous	Adipose Tissue	GTEX-R55G-2426-SM-2TC5I
Adipose - Subcutaneous	Adipose Tissue	GTEX-REY6-0326-SM-2TF5A
Adipose - Subcutaneous	Adipose Tissue	GTEX-RM2N-1726-SM-2TF55
Adipose - Subcutaneous	Adipose Tissue	GTEX-RTLS-0226-SM-2TF5E
Adipose - Subcutaneous	Adipose Tissue	GTEX-RU72-1026-SM-46MUG
Adipose - Subcutaneous	Adipose Tissue	GTEX-RWSA-0226-SM-2XCBA
Adipose - Subcutaneous	Adipose Tissue	GTEX-S32W-2226-SM-2XCAY
Adipose - Subcutaneous	Adipose Tissue	GTEX-S33H-1126-SM-2XCB6
Adipose - Subcutaneous	Adipose Tissue	GTEX-S7SE-0226-SM-2XCD4
Adipose - Subcutaneous	Adipose Tissue	GTEX-S95S-1326-SM-2XCDK
Adipose - Subcutaneous	Adipose Tissue	GTEX-SIU8-0226-SM-2XCDS
Adipose - Subcutaneous	Adipose Tissue	GTEX-SJXC-0226-SM-2XCDU
Adipose - Subcutaneous	Adipose Tissue	GTEX-SN8G-0226-SM-4DM6B
Adipose - Subcutaneous	Adipose Tissue	GTEX-SNMC-1326-SM-2XCFK
Adipose - Subcutaneous	Adipose Tissue	GTEX-SNOS-1426-SM-32PLY
Adipose - Subcutaneous	Adipose Tissue	GTEX-SSA3-0226-SM-32QPN
Adipose - Subcutaneous	Adipose Tissue	GTEX-T2IS-0226-SM-32QPH
Adipose - Subcutaneous	Adipose Tissue	GTEX-T5JC-0526-SM-32PM7
Adipose - Subcutaneous	Adipose Tissue	GTEX-T5JW-1726-SM-3GADN
Adipose - Subcutaneous	Adipose Tissue	GTEX-T6MN-0226-SM-32PMD
Adipose - Subcutaneous	Adipose Tissue	GTEX-T6MO-1726-SM-33HB8
Adipose - Subcutaneous	Adipose Tissue	GTEX-T8EM-1126-SM-3DB7D
Adipose - Subcutaneous	Adipose Tissue	GTEX-TKQ1-1126-SM-4GIAZ

Adipose - Subcutaneous	Adipose Tissue	GTEX-TML8-2026-SM-32QOP
Adipose - Subcutaneous	Adipose Tissue	GTEX-TMZS-0226-SM-3DB9N
Adipose - Subcutaneous	Adipose Tissue	GTEX-TSE9-0226-SM-3DB84
Adipose - Subcutaneous	Adipose Tissue	GTEX-U3ZN-2626-SM-3DB7T
Adipose - Subcutaneous	Adipose Tissue	GTEX-U412-0526-SM-3DB9I
Adipose - Subcutaneous	Adipose Tissue	GTEX-U4B1-1726-SM-3DB9F
Adipose - Subcutaneous	Adipose Tissue	GTEX-U8XE-0426-SM-3DB91
Adipose - Subcutaneous	Adipose Tissue	GTEX-UJHI-1626-SM-3DB9A
Adipose - Subcutaneous	Adipose Tissue	GTEX-UTHO-0426-SM-5SI7N
Adipose - Subcutaneous	Adipose Tissue	GTEX-VJYA-1326-SM-3GIJC
Adipose - Subcutaneous	Adipose Tissue	GTEX-VUSG-2426-SM-4KKZG
Adipose - Subcutaneous	Adipose Tissue	GTEX-W5WG-2526-SM-4SOJ2
Adipose - Subcutaneous	Adipose Tissue	GTEX-W5X1-2626-SM-4LMI8
Adipose - Subcutaneous	Adipose Tissue	GTEX-WEY5-1926-SM-3GIL8
Adipose - Subcutaneous	Adipose Tissue	GTEX-WFG8-2326-SM-5S2UE
Adipose - Subcutaneous	Adipose Tissue	GTEX-WFJO-1926-SM-3GILA
Adipose - Subcutaneous	Adipose Tissue	GTEX-WFON-2226-SM-3TW8W
Adipose - Subcutaneous	Adipose Tissue	GTEX-WH7G-2226-SM-3NMBN
Adipose - Subcutaneous	Adipose Tissue	GTEX-WHPG-2126-SM-4M1ZM
Adipose - Subcutaneous	Adipose Tissue	GTEX-WI4N-1126-SM-3LK7Q
Adipose - Subcutaneous	Adipose Tissue	GTEX-WL46-0326-SM-3LK6Y
Adipose - Subcutaneous	Adipose Tissue	GTEX-WVJS-0226-SM-4MVOU
Adipose - Subcutaneous	Adipose Tissue	GTEX-WVLH-0226-SM-3MJG6
Adipose - Subcutaneous	Adipose Tissue	GTEX-WY7C-2426-SM-3NB2V
Adipose - Subcutaneous	Adipose Tissue	GTEX-X15G-2326-SM-4PQZS
Adipose - Subcutaneous	Adipose Tissue	GTEX-X261-0226-SM-3NMD2
Adipose - Subcutaneous	Adipose Tissue	GTEX-X4EO-0426-SM-4QASA
Adipose - Subcutaneous	Adipose Tissue	GTEX-X4LF-1726-SM-3NMBZ
Adipose - Subcutaneous	Adipose Tissue	GTEX-X4XY-0326-SM-46MVZ
Adipose - Subcutaneous	Adipose Tissue	GTEX-X5EB-2426-SM-4E3HX
Adipose - Subcutaneous	Adipose Tissue	GTEX-X62O-0226-SM-4E3JB
Adipose - Subcutaneous	Adipose Tissue	GTEX-X638-0226-SM-47JZ9
Adipose - Subcutaneous	Adipose Tissue	GTEX-X88G-0226-SM-4GIE4
Adipose - Subcutaneous	Adipose Tissue	GTEX-X8HC-0226-SM-4E3K1
Adipose - Subcutaneous	Adipose Tissue	GTEX-XAJ8-0926-SM-47JXZ
Adipose - Subcutaneous	Adipose Tissue	GTEX-XBEC-0326-SM-4AT4M
Adipose - Subcutaneous	Adipose Tissue	GTEX-XBED-2326-SM-47JYR
Adipose - Subcutaneous	Adipose Tissue	GTEX-XBEW-0726-SM-4QARX
Adipose - Subcutaneous	Adipose Tissue	GTEX-XGQ4-2226-SM-4AT4Y
Adipose - Subcutaneous	Adipose Tissue	GTEX-XK95-0426-SM-4AT4R
Adipose - Subcutaneous	Adipose Tissue	GTEX-XLM4-0226-SM-4AT4N
Adipose - Subcutaneous	Adipose Tissue	GTEX-XMD2-0226-SM-4WWEE
Adipose - Subcutaneous	Adipose Tissue	GTEX-XMK1-2226-SM-4B673
Adipose - Subcutaneous	Adipose Tissue	GTEX-XPVG-2726-SM-4B66W
Adipose - Subcutaneous	Adipose Tissue	GTEX-XQ3S-1626-SM-4WAYN
Adipose - Subcutaneous	Adipose Tissue	GTEX-XQ8I-0526-SM-4BOPS
Adipose - Subcutaneous	Adipose Tissue	GTEX-XUW1-0526-SM-4BOP3
Adipose - Subcutaneous	Adipose Tissue	GTEX-XUYS-0226-SM-47JX1
Adipose - Subcutaneous	Adipose Tissue	GTEX-XUZC-1826-SM-4BRVO
Adipose - Subcutaneous	Adipose Tissue	GTEX-XV7Q-2626-SM-4BRVA
Adipose - Subcutaneous	Adipose Tissue	GTEX-XXEK-2426-SM-4BRUS
Adipose - Subcutaneous	Adipose Tissue	GTEX-XYKS-2726-SM-4E3IC
Adipose - Subcutaneous	Adipose Tissue	GTEX-Y111-0226-SM-4SOIW
Adipose - Subcutaneous	Adipose Tissue	GTEX-Y114-2326-SM-4TT7X

Adipose - Subcutaneous	Adipose Tissue	GTEX-Y3I4-2226-SM-4TT7O
Adipose - Subcutaneous	Adipose Tissue	GTEX-Y3IK-2526-SM-4WWDM
Adipose - Subcutaneous	Adipose Tissue	GTEX-Y5V5-2426-SM-5IFJA
Adipose - Subcutaneous	Adipose Tissue	GTEX-Y5V6-2526-SM-5IFJV
Adipose - Subcutaneous	Adipose Tissue	GTEX-Y8E4-0726-SM-4WWFN
Adipose - Subcutaneous	Adipose Tissue	GTEX-Y8E5-0226-SM-57WCM
Adipose - Subcutaneous	Adipose Tissue	GTEX-Y8LW-1926-SM-4WWFZ
Adipose - Subcutaneous	Adipose Tissue	GTEX-Y9LG-2026-SM-62LFS
Adipose - Subcutaneous	Adipose Tissue	GTEX-YB5E-2126-SM-5IFI7
Adipose - Subcutaneous	Adipose Tissue	GTEX-YB5K-2226-SM-4WWG1
Adipose - Subcutaneous	Adipose Tissue	GTEX-YEC3-1226-SM-5IFI8
Adipose - Subcutaneous	Adipose Tissue	GTEX-YEC4-2426-SM-57WFO
Adipose - Subcutaneous	Adipose Tissue	GTEX-YECK-0226-SM-4W215
Adipose - Subcutaneous	Adipose Tissue	GTEX-YF7O-2426-SM-5IFJL
Adipose - Subcutaneous	Adipose Tissue	GTEX-YFC4-0226-SM-57WD2
Adipose - Subcutaneous	Adipose Tissue	GTEX-YFCO-2026-SM-4W21Q
Adipose - Subcutaneous	Adipose Tissue	GTEX-YJ89-0226-SM-4TT3Y
Adipose - Subcutaneous	Adipose Tissue	GTEX-YJ8A-0526-SM-5IFHT
Adipose - Subcutaneous	Adipose Tissue	GTEX-YJ8O-2426-SM-5HL7S
Adipose - Subcutaneous	Adipose Tissue	GTEX-Z93S-0226-SM-5HL7R
Adipose - Subcutaneous	Adipose Tissue	GTEX-Z93T-0226-SM-5HL5Z
Adipose - Subcutaneous	Adipose Tissue	GTEX-Z9EW-1826-SM-5CVMA
Adipose - Subcutaneous	Adipose Tissue	GTEX-ZAJG-0226-SM-5HL81
Adipose - Subcutaneous	Adipose Tissue	GTEX-ZAK1-0226-SM-5CVMX
Adipose - Subcutaneous	Adipose Tissue	GTEX-ZC5H-0126-SM-4WAYL
Adipose - Subcutaneous	Adipose Tissue	GTEX-ZDTS-0226-SM-5HL7Q
Adipose - Subcutaneous	Adipose Tissue	GTEX-ZDTT-2626-SM-5S2OP
Adipose - Subcutaneous	Adipose Tissue	GTEX-ZDXO-2426-SM-5S2NJ
Adipose - Subcutaneous	Adipose Tissue	GTEX-ZDYS-2126-SM-5S2OD
Adipose - Subcutaneous	Adipose Tissue	GTEX-ZE7O-0226-SM-5S2N1
Adipose - Subcutaneous	Adipose Tissue	GTEX-ZE9C-2826-SM-5S2MO
Adipose - Subcutaneous	Adipose Tissue	GTEX-ZEX8-2326-SM-5S2MN
Adipose - Subcutaneous	Adipose Tissue	GTEX-ZF28-0226-SM-5S2OE
Adipose - Subcutaneous	Adipose Tissue	GTEX-ZF29-2226-SM-4WWB9
Adipose - Subcutaneous	Adipose Tissue	GTEX-ZF3C-0226-SM-4WWB3
Adipose - Subcutaneous	Adipose Tissue	GTEX-ZLV1-1826-SM-5FQU5
Adipose - Subcutaneous	Adipose Tissue	GTEX-ZLWG-2226-SM-5DUWO
Adipose - Subcutaneous	Adipose Tissue	GTEX-ZP4G-2326-SM-57WEM
Adipose - Subcutaneous	Adipose Tissue	GTEX-ZPCL-2126-SM-57WFZ
Adipose - Subcutaneous	Adipose Tissue	GTEX-ZPIC-1826-SM-57WFE
Adipose - Subcutaneous	Adipose Tissue	GTEX-ZPU1-2426-SM-4WWFM
Adipose - Subcutaneous	Adipose Tissue	GTEX-ZQG8-1526-SM-5HL65
Adipose - Subcutaneous	Adipose Tissue	GTEX-ZT9X-1926-SM-57WE7
Adipose - Subcutaneous	Adipose Tissue	GTEX-ZTPG-0226-SM-5O99I
Adipose - Subcutaneous	Adipose Tissue	GTEX-ZTSS-2026-SM-5987K
Adipose - Subcutaneous	Adipose Tissue	GTEX-ZTX8-1526-SM-5N9GI
Adipose - Subcutaneous	Adipose Tissue	GTEX-ZUA1-0226-SM-5NQ9Q
Adipose - Subcutaneous	Adipose Tissue	GTEX-ZV68-0226-SM-59HJF
Adipose - Subcutaneous	Adipose Tissue	GTEX-ZVE2-0326-SM-57WFC
Adipose - Subcutaneous	Adipose Tissue	GTEX-ZVP2-1826-SM-5GU62
Adipose - Subcutaneous	Adipose Tissue	GTEX-ZVT2-2226-SM-5NQ6P
Adipose - Subcutaneous	Adipose Tissue	GTEX-ZVT4-0226-SM-51MSD
Adipose - Subcutaneous	Adipose Tissue	GTEX-ZVTK-0526-SM-5GZWT
Adipose - Subcutaneous	Adipose Tissue	GTEX-ZVZO-0226-SM-5A5LA

Adipose - Subcutaneous   Adipose Tissue   GTEX-ZVZP-2326-SM-59HL4
Adipose - Subcutaneous   Adipose Tissue   GTEX-ZXES-2026-SM-5NQ6R
Adipose - Subcutaneous   Adipose Tissue   GTEX-ZXG5-0226-SM-59HJI
Adipose - Subcutaneous   Adipose Tissue   GTEX-ZYFC-0326-SM-5NQ7H
Adipose - Subcutaneous   Adipose Tissue   GTEX-ZYFD-0226-SM-5NQ86
Adipose - Subcutaneous   Adipose Tissue   GTEX-ZYT6-0326-SM-7LG5R
Adipose - Subcutaneous   Adipose Tissue   GTEX-ZYVF-0226-SM-5GIEG
Adipose - Subcutaneous   Adipose Tissue   GTEX-ZYW4-0226-SM-5E44M
Adipose - Subcutaneous   Adipose Tissue   GTEX-ZYY3-0226-SM-5E45M
Adipose - Subcutaneous   Adipose Tissue   GTEX-ZZ64-1626-SM-5E43W
Adipose - Subcutaneous   Adipose Tissue   GTEX-ZZPU-2726-SM-5NQ8O
Adipose - Visceral (Omentum)   Adipose Tissue   GTEX-1117F-1326-SM-5EGHH
Adipose - Visceral (Omentum)   Adipose Tissue   GTEX-111CU-1026-SM-5EGIL
Adipose - Visceral (Omentum)   Adipose Tissue   GTEX-111YS-1326-SM-5EGGK
Adipose - Visceral (Omentum)   Adipose Tissue   GTEX-1122O-0926-SM-5N9C9
Adipose - Visceral (Omentum)   Adipose Tissue   GTEX-1128S-0926-SM-5GZZU
Adipose - Visceral (Omentum)   Adipose Tissue   GTEX-113JC-0726-SM-5GZZR
Adipose - Visceral (Omentum)   Adipose Tissue   GTEX-117YW-0826-SM-5H11O
Adipose - Visceral (Omentum)   Adipose Tissue   GTEX-117YX-0726-SM-5GIET
Adipose - Visceral (Omentum)   Adipose Tissue   GTEX-1192X-1526-SM-5H11I
Adipose - Visceral (Omentum)   Adipose Tissue   GTEX-11DXX-2026-SM-5EGJL
Adipose - Visceral (Omentum)   Adipose Tissue   GTEX-11DXY-0326-SM-5H12F
Adipose - Visceral (Omentum)   Adipose Tissue   GTEX-11DXZ-1426-SM-5H12A
Adipose - Visceral (Omentum)   Adipose Tissue   GTEX-11DYG-2026-SM-5EGGX
Adipose - Visceral (Omentum)   Adipose Tissue   GTEX-11EM3-1126-SM-5A5KM
Adipose - Visceral (Omentum)   Adipose Tissue   GTEX-11EQ9-1126-SM-5987I
Adipose - Visceral (Omentum)   Adipose Tissue   GTEX-11GS4-1026-SM-5N9DE
Adipose - Visceral (Omentum)   Adipose Tissue   GTEX-11GSO-0226-SM-5A5LV
Adipose - Visceral (Omentum)   Adipose Tissue   GTEX-11I78-0226-SM-5HL6R
Adipose - Visceral (Omentum)   Adipose Tissue   GTEX-11LCK-0326-SM-5A5M7
Adipose - Visceral (Omentum)   Adipose Tissue   GTEX-11NUK-2026-SM-5PNVZ
Adipose - Visceral (Omentum)   Adipose Tissue   GTEX-11NV4-1426-SM-5BC4W
Adipose - Visceral (Omentum)   Adipose Tissue   GTEX-11OF3-0826-

SM-5BC51
Adipose - Visceral (Omentum)   Adipose Tissue   GTEX-11P7K-0826-
SM-5BC5F
Adipose - Visceral (Omentum)   Adipose Tissue   GTEX-11P81-0726-
SM-5PNYH
Adipose - Visceral (Omentum)   Adipose Tissue   GTEX-11P82-0326-
SM-5HL51
Adipose - Visceral (Omentum)   Adipose Tissue   GTEX-11PRG-1526-
SM-5HL6Z
Adipose - Visceral (Omentum)   Adipose Tissue   GTEX-11TT1-1926-
SM-5PNYN
Adipose - Visceral (Omentum)   Adipose Tissue   GTEX-11VI4-1226-
SM-5EQMP
Adipose - Visceral (Omentum)   Adipose Tissue   GTEX-11WQK-1526-
SM-5EGKN
Adipose - Visceral (Omentum)   Adipose Tissue   GTEX-11XUK-1726-
SM-5GU61
Adipose - Visceral (Omentum)   Adipose Tissue   GTEX-11ZTS-1326-
SM-5EQMA
Adipose - Visceral (Omentum)   Adipose Tissue   GTEX-11ZUS-1826-
SM-5FQTR
Adipose - Visceral (Omentum)   Adipose Tissue   GTEX-1211K-1226-
SM-5EQ4N
Adipose - Visceral (Omentum)   Adipose Tissue   GTEX-1212Z-0526-
SM-5FQSQ
Adipose - Visceral (Omentum)   Adipose Tissue   GTEX-12584-2126-
SM-5FQUM
Adipose - Visceral (Omentum)   Adipose Tissue   GTEX-12696-1826-
SM-5EGJS
Adipose - Visceral (Omentum)   Adipose Tissue   GTEX-12BJ1-0826-
SM-5EQ5P
Adipose - Visceral (Omentum)   Adipose Tissue   GTEX-12C56-0826-
SM-5EGJ5
Adipose - Visceral (Omentum)   Adipose Tissue   GTEX-12KS4-1526-
SM-5EQ6E
Adipose - Visceral (Omentum)   Adipose Tissue   GTEX-12WSA-1626-
SM-5EGJV
Adipose - Visceral (Omentum)   Adipose Tissue   GTEX-12WSC-1726-
SM-5GCN3
Adipose - Visceral (Omentum)   Adipose Tissue   GTEX-12WSF-1526-
SM-6PAN6
Adipose - Visceral (Omentum)   Adipose Tissue   GTEX-12WSG-0426-
SM-5FQSE
Adipose - Visceral (Omentum)   Adipose Tissue   GTEX-12WSI-1526-
SM-5GCNM
Adipose - Visceral (Omentum)   Adipose Tissue   GTEX-12WSK-1026-
SM-5CVNR
Adipose - Visceral (Omentum)   Adipose Tissue   GTEX-12WSL-1126-
SM-5CVNK
Adipose - Visceral (Omentum)   Adipose Tissue   GTEX-12WSM-0626-
SM-5GCOK
Adipose - Visceral (Omentum)   Adipose Tissue   GTEX-12ZZW-2326-
SM-5DUW2
Adipose - Visceral (Omentum)   Adipose Tissue   GTEX-12ZZX-1726-

SM-5BC63
Adipose - Visceral (Omentum)	Adipose Tissue	GTEX-13111-0626-
SM-5LZVR
Adipose - Visceral (Omentum)	Adipose Tissue	GTEX-13112-1126-
SM-5GCMY
Adipose - Visceral (Omentum)	Adipose Tissue	GTEX-13113-1126-
SM-5LZU9
Adipose - Visceral (Omentum)	Adipose Tissue	GTEX-1313W-1626-
SM-5J1OE
Adipose - Visceral (Omentum)	Adipose Tissue	GTEX-131XE-2026-
SM-5LZW1
Adipose - Visceral (Omentum)	Adipose Tissue	GTEX-131XF-0726-
SM-5GIE5
Adipose - Visceral (Omentum)	Adipose Tissue	GTEX-131XG-0126-
SM-5K7UJ
Adipose - Visceral (Omentum)	Adipose Tissue	GTEX-131XH-0826-
SM-5GCN8
Adipose - Visceral (Omentum)	Adipose Tissue	GTEX-131YS-1726-
SM-5HL7G
Adipose - Visceral (Omentum)	Adipose Tissue	GTEX-132AR-0526-
SM-5J2MJ
Adipose - Visceral (Omentum)	Adipose Tissue	GTEX-132NY-1126-
SM-5PNVE
Adipose - Visceral (Omentum)	Adipose Tissue	GTEX-132Q8-1726-
SM-5EGK8
Adipose - Visceral (Omentum)	Adipose Tissue	GTEX-132QS-0826-
SM-5K7WV
Adipose - Visceral (Omentum)	Adipose Tissue	GTEX-1339X-0726-
SM-5L3FF
Adipose - Visceral (Omentum)	Adipose Tissue	GTEX-133LE-0926-
SM-5P9J1
Adipose - Visceral (Omentum)	Adipose Tissue	GTEX-1399S-1426-
SM-5PNYQ
Adipose - Visceral (Omentum)	Adipose Tissue	GTEX-1399T-1326-
SM-5PNVH
Adipose - Visceral (Omentum)	Adipose Tissue	GTEX-139D8-1626-
SM-5IJFZ
Adipose - Visceral (Omentum)	Adipose Tissue	GTEX-139T6-0526-
SM-5IJC9
Adipose - Visceral (Omentum)	Adipose Tissue	GTEX-139TU-1426-
SM-5LZY9
Adipose - Visceral (Omentum)	Adipose Tissue	GTEX-139YR-0326-
SM-5J1MC
Adipose - Visceral (Omentum)	Adipose Tissue	GTEX-13CF3-1126-
SM-5LZXB
Adipose - Visceral (Omentum)	Adipose Tissue	GTEX-13D11-0526-
SM-5LZYM
Adipose - Visceral (Omentum)	Adipose Tissue	GTEX-13FH7-1926-
SM-5J2OL
Adipose - Visceral (Omentum)	Adipose Tissue	GTEX-13FHO-2026-
SM-5J1OA
Adipose - Visceral (Omentum)	Adipose Tissue	GTEX-13FTW-0426-
SM-5K7X2
Adipose - Visceral (Omentum)	Adipose Tissue	GTEX-13FTX-0626-

SM-5J2NS
Adipose - Visceral (Omentum)   Adipose Tissue   GTEX-13FXS-1326-
SM-62LFR
Adipose - Visceral (Omentum)   Adipose Tissue   GTEX-13G51-0526-
SM-5IJCQ
Adipose - Visceral (Omentum)   Adipose Tissue   GTEX-13IVO-2426-
SM-5K7V9
Adipose - Visceral (Omentum)   Adipose Tissue   GTEX-13JUV-0626-
SM-5J1MQ
Adipose - Visceral (Omentum)   Adipose Tissue   GTEX-13N11-1826-
SM-5IJDS
Adipose - Visceral (Omentum)   Adipose Tissue   GTEX-13N1W-1226-
SM-5K7UM
Adipose - Visceral (Omentum)   Adipose Tissue   GTEX-13N2G-1926-
SM-5J1O3
Adipose - Visceral (Omentum)   Adipose Tissue   GTEX-13NYS-0726-
SM-5J1OR
Adipose - Visceral (Omentum)   Adipose Tissue   GTEX-13NZ9-2026-
SM-5KM1U
Adipose - Visceral (Omentum)   Adipose Tissue   GTEX-13NZB-0826-
SM-5K7X5
Adipose - Visceral (Omentum)   Adipose Tissue   GTEX-13O21-3126-
SM-5J1OI
Adipose - Visceral (Omentum)   Adipose Tissue   GTEX-13O3P-0926-
SM-5IFFI
Adipose - Visceral (Omentum)   Adipose Tissue   GTEX-13O3Q-1126-
SM-5K7YK
Adipose - Visceral (Omentum)   Adipose Tissue   GTEX-13O61-1926-
SM-5KM1R
Adipose - Visceral (Omentum)   Adipose Tissue   GTEX-13OVG-1326-
SM-5K7Y3
Adipose - Visceral (Omentum)   Adipose Tissue   GTEX-13OVH-1126-
SM-5K7YP
Adipose - Visceral (Omentum)   Adipose Tissue   GTEX-13OVJ-0926-
SM-5K7U1
Adipose - Visceral (Omentum)   Adipose Tissue   GTEX-13OVK-0326-
SM-7KUEJ
Adipose - Visceral (Omentum)   Adipose Tissue   GTEX-13OVL-0826-
SM-5L3GV
Adipose - Visceral (Omentum)   Adipose Tissue   GTEX-13OW6-2426-
SM-5K7WO
Adipose - Visceral (Omentum)   Adipose Tissue   GTEX-13OW7-1126-
SM-5K7V6
Adipose - Visceral (Omentum)   Adipose Tissue   GTEX-13OW8-0326-
SM-5L3FB
Adipose - Visceral (Omentum)   Adipose Tissue   GTEX-13PDP-1226-
SM-5K7TW
Adipose - Visceral (Omentum)   Adipose Tissue   GTEX-13PL6-0926-
SM-5KLYY
Adipose - Visceral (Omentum)   Adipose Tissue   GTEX-13PL7-1226-
SM-5SIAR
Adipose - Visceral (Omentum)   Adipose Tissue   GTEX-13PVQ-2226-
SM-5L3G9
Adipose - Visceral (Omentum)   Adipose Tissue   GTEX-13PVR-0526-

SM-5S2OB
Adipose - Visceral (Omentum)	Adipose Tissue	GTEX-13QBU-1326-
SM-5LU4K
Adipose - Visceral (Omentum)	Adipose Tissue	GTEX-13RTK-0726-
SM-5Q5EN
Adipose - Visceral (Omentum)	Adipose Tissue	GTEX-13S7M-0826-
SM-5QGQX
Adipose - Visceral (Omentum)	Adipose Tissue	GTEX-13S86-0926-
SM-5SI6I
Adipose - Visceral (Omentum)	Adipose Tissue	GTEX-13U4I-1726-
SM-5J1MF
Adipose - Visceral (Omentum)	Adipose Tissue	GTEX-13VXT-0926-
SM-5LU5K
Adipose - Visceral (Omentum)	Adipose Tissue	GTEX-13VXU-1026-
SM-5KLZE
Adipose - Visceral (Omentum)	Adipose Tissue	GTEX-13W3W-1126-
SM-5L3DO
Adipose - Visceral (Omentum)	Adipose Tissue	GTEX-13W46-1226-
SM-5SIAS
Adipose - Visceral (Omentum)	Adipose Tissue	GTEX-13X6H-1726-
SM-5Q5EH
Adipose - Visceral (Omentum)	Adipose Tissue	GTEX-13X6I-1526-
SM-5SIAC
Adipose - Visceral (Omentum)	Adipose Tissue	GTEX-13X6J-1926-
SM-5TDCU
Adipose - Visceral (Omentum)	Adipose Tissue	GTEX-13X6K-0726-
SM-5LU2Z
Adipose - Visceral (Omentum)	Adipose Tissue	GTEX-13YAN-1126-
SM-5TDC7
Adipose - Visceral (Omentum)	Adipose Tissue	GTEX-1445S-2426-
SM-79ON2
Adipose - Visceral (Omentum)	Adipose Tissue	GTEX-144GM-0626-
SM-79OKY
Adipose - Visceral (Omentum)	Adipose Tissue	GTEX-144GN-0726-
SM-5O98O
Adipose - Visceral (Omentum)	Adipose Tissue	GTEX-145LT-1526-
SM-5O98P
Adipose - Visceral (Omentum)	Adipose Tissue	GTEX-145LU-1226-
SM-5LU9B
Adipose - Visceral (Omentum)	Adipose Tissue	GTEX-145LV-1026-
SM-5O9AE
Adipose - Visceral (Omentum)	Adipose Tissue	GTEX-145ME-0726-
SM-5O9A3
Adipose - Visceral (Omentum)	Adipose Tissue	GTEX-145MF-1026-
SM-5Q5EQ
Adipose - Visceral (Omentum)	Adipose Tissue	GTEX-145MI-0826-
SM-5SI95
Adipose - Visceral (Omentum)	Adipose Tissue	GTEX-146FH-2026-
SM-5SI9I
Adipose - Visceral (Omentum)	Adipose Tissue	GTEX-146FR-0226-
SM-5Q5F6
Adipose - Visceral (Omentum)	Adipose Tissue	GTEX-14753-2526-
SM-5LU97
Adipose - Visceral (Omentum)	Adipose Tissue	GTEX-147JS-1026-

SM-5S2MP
Adipose - Visceral (Omentum)	Adipose Tissue	GTEX-14A6H-1026-
SM-5SIAZ
Adipose - Visceral (Omentum)	Adipose Tissue	GTEX-14ABY-1926-
SM-62LDJ
Adipose - Visceral (Omentum)	Adipose Tissue	GTEX-14AS3-0426-
SM-5QGQK
Adipose - Visceral (Omentum)	Adipose Tissue	GTEX-14B4R-0526-
SM-5QGQT
Adipose - Visceral (Omentum)	Adipose Tissue	GTEX-14BIL-2426-
SM-5QGQR
Adipose - Visceral (Omentum)	Adipose Tissue	GTEX-14BIM-1626-
SM-7EWD2
Adipose - Visceral (Omentum)	Adipose Tissue	GTEX-14BIN-1226-
SM-5S2OM
Adipose - Visceral (Omentum)	Adipose Tissue	GTEX-14BMU-0626-
SM-73KZ6
Adipose - Visceral (Omentum)	Adipose Tissue	GTEX-14BMV-1626-
SM-73KVQ
Adipose - Visceral (Omentum)	Adipose Tissue	GTEX-14C38-1026-
SM-73KY7
Adipose - Visceral (Omentum)	Adipose Tissue	GTEX-14C39-0826-
SM-5TDDU
Adipose - Visceral (Omentum)	Adipose Tissue	GTEX-14C5O-0926-
SM-793DM
Adipose - Visceral (Omentum)	Adipose Tissue	GTEX-14DAQ-2526-
SM-5RQIO
Adipose - Visceral (Omentum)	Adipose Tissue	GTEX-14DAR-0426-
SM-664MW
Adipose - Visceral (Omentum)	Adipose Tissue	GTEX-14E1K-0426-
SM-5S2PF
Adipose - Visceral (Omentum)	Adipose Tissue	GTEX-14E6C-1226-
SM-5ZZUN
Adipose - Visceral (Omentum)	Adipose Tissue	GTEX-14E6D-1326-
SM-62LES
Adipose - Visceral (Omentum)	Adipose Tissue	GTEX-14E6E-0226-
SM-7DUEF
Adipose - Visceral (Omentum)	Adipose Tissue	GTEX-14E7W-2326-
SM-69LQC
Adipose - Visceral (Omentum)	Adipose Tissue	GTEX-14ICK-1726-
SM-6AJA5
Adipose - Visceral (Omentum)	Adipose Tissue	GTEX-14ICL-0926-
SM-5S2TU
Adipose - Visceral (Omentum)	Adipose Tissue	GTEX-14JG6-0526-
SM-6LLIH
Adipose - Visceral (Omentum)	Adipose Tissue	GTEX-14JIY-1426-
SM-6EU29
Adipose - Visceral (Omentum)	Adipose Tissue	GTEX-14PHW-1626-
SM-6EU2H
Adipose - Visceral (Omentum)	Adipose Tissue	GTEX-14PHX-1626-
SM-69LQB
Adipose - Visceral (Omentum)	Adipose Tissue	GTEX-14PHY-1026-
SM-62LEV
Adipose - Visceral (Omentum)	Adipose Tissue	GTEX-14PJ2-1426-

SM-62LFF
Adipose - Visceral (Omentum)	Adipose Tissue	GTEX-14PJ3-0726-
SM-69LQE
Adipose - Visceral (Omentum)	Adipose Tissue	GTEX-14PJ4-0126-
SM-62LEZ
Adipose - Visceral (Omentum)	Adipose Tissue	GTEX-14PJ5-0426-
SM-5ZZW2
Adipose - Visceral (Omentum)	Adipose Tissue	GTEX-14PJM-1226-
SM-686Z7
Adipose - Visceral (Omentum)	Adipose Tissue	GTEX-14PJN-0626-
SM-6EU13
Adipose - Visceral (Omentum)	Adipose Tissue	GTEX-14PJO-1626-
SM-69LPQ
Adipose - Visceral (Omentum)	Adipose Tissue	GTEX-14PK6-0926-
SM-62LF4
Adipose - Visceral (Omentum)	Adipose Tissue	GTEX-14PKU-0226-
SM-6EU16
Adipose - Visceral (Omentum)	Adipose Tissue	GTEX-14PN3-0326-
SM-6EU1X
Adipose - Visceral (Omentum)	Adipose Tissue	GTEX-14PN4-1426-
SM-6LLJ4
Adipose - Visceral (Omentum)	Adipose Tissue	GTEX-15CHC-0826-
SM-686YW
Adipose - Visceral (Omentum)	Adipose Tissue	GTEX-15CHQ-1126-
SM-686YY
Adipose - Visceral (Omentum)	Adipose Tissue	GTEX-15D1Q-1326-
SM-69LOA
Adipose - Visceral (Omentum)	Adipose Tissue	GTEX-15DCZ-1726-
SM-69LPX
Adipose - Visceral (Omentum)	Adipose Tissue	GTEX-15DYW-1526-
SM-7KUEP
Adipose - Visceral (Omentum)	Adipose Tissue	GTEX-15DZA-0626-
SM-6PANC
Adipose - Visceral (Omentum)	Adipose Tissue	GTEX-15EOM-2326-
SM-7KUMV
Adipose - Visceral (Omentum)	Adipose Tissue	GTEX-15ER7-1026-
SM-7KUMD
Adipose - Visceral (Omentum)	Adipose Tissue	GTEX-15EU6-1726-
SM-6M48H
Adipose - Visceral (Omentum)	Adipose Tissue	GTEX-15G19-1126-
SM-7KUEL
Adipose - Visceral (Omentum)	Adipose Tissue	GTEX-15RIE-0126-
SM-7KUF8
Adipose - Visceral (Omentum)	Adipose Tissue	GTEX-15SHU-2326-
SM-7KUMC
Adipose - Visceral (Omentum)	Adipose Tissue	GTEX-15SHW-1726-
SM-6LPJ2
Adipose - Visceral (Omentum)	Adipose Tissue	GTEX-15SKB-1626-
SM-6PAL6
Adipose - Visceral (Omentum)	Adipose Tissue	GTEX-15UKP-1426-
SM-7KUDX
Adipose - Visceral (Omentum)	Adipose Tissue	GTEX-169BO-0426-
SM-7EPI1
Adipose - Visceral (Omentum)	Adipose Tissue	GTEX-16AAH-1526-

SM-7LT9W
Adipose - Visceral (Omentum)	Adipose Tissue	GTEX-16BQI-1126-SM-7KULF
Adipose - Visceral (Omentum)	Adipose Tissue	GTEX-16GPK-1326-SM-7KUE4
Adipose - Visceral (Omentum)	Adipose Tissue	GTEX-16MTA-1326-SM-6PALX
Adipose - Visceral (Omentum)	Adipose Tissue	GTEX-16NGA-0526-SM-731AJ
Adipose - Visceral (Omentum)	Adipose Tissue	GTEX-16NPV-0626-SM-7IGOR
Adipose - Visceral (Omentum)	Adipose Tissue	GTEX-16NPX-1026-SM-6PAM7
Adipose - Visceral (Omentum)	Adipose Tissue	GTEX-16XZZ-1626-SM-7KUEC
Adipose - Visceral (Omentum)	Adipose Tissue	GTEX-178AV-0626-SM-6PAM1
Adipose - Visceral (Omentum)	Adipose Tissue	GTEX-17EVP-1426-SM-79OMN
Adipose - Visceral (Omentum)	Adipose Tissue	GTEX-17EVQ-0626-SM-7KFTB
Adipose - Visceral (Omentum)	Adipose Tissue	GTEX-17F96-0726-SM-793CB
Adipose - Visceral (Omentum)	Adipose Tissue	GTEX-17F97-1226-SM-79OJT
Adipose - Visceral (Omentum)	Adipose Tissue	GTEX-17F98-0326-SM-7DHLH
Adipose - Visceral (Omentum)	Adipose Tissue	GTEX-17F9E-1326-SM-7DHL4
Adipose - Visceral (Omentum)	Adipose Tissue	GTEX-17F9Y-0726-SM-7LT9G
Adipose - Visceral (Omentum)	Adipose Tissue	GTEX-17GQL-0826-SM-731AM
Adipose - Visceral (Omentum)	Adipose Tissue	GTEX-17HGU-1726-SM-79OKM
Adipose - Visceral (Omentum)	Adipose Tissue	GTEX-17HHE-1526-SM-793AS
Adipose - Visceral (Omentum)	Adipose Tissue	GTEX-17HHY-1026-SM-7LG5E
Adipose - Visceral (Omentum)	Adipose Tissue	GTEX-17HII-0826-SM-7DUFH
Adipose - Visceral (Omentum)	Adipose Tissue	GTEX-17JCI-1626-SM-7EPGZ
Adipose - Visceral (Omentum)	Adipose Tissue	GTEX-17KNJ-0426-SM-718AK
Adipose - Visceral (Omentum)	Adipose Tissue	GTEX-17MF6-1326-SM-79ONE
Adipose - Visceral (Omentum)	Adipose Tissue	GTEX-17MFQ-1126-SM-7LT9O
Adipose - Visceral (Omentum)	Adipose Tissue	GTEX-183FY-1226-SM-7DHLK
Adipose - Visceral (Omentum)	Adipose Tissue	GTEX-18465-1826-SM-731BB
Adipose - Visceral (Omentum)	Adipose Tissue	GTEX-18A66-1926-

SM-7LTAG
Adipose - Visceral (Omentum)	Adipose Tissue	GTEX-18A67-1326-
SM-7LT8V
Adipose - Visceral (Omentum)	Adipose Tissue	GTEX-18A6Q-1026-
SM-7LT9S
Adipose - Visceral (Omentum)	Adipose Tissue	GTEX-18A7A-0926-
SM-7LT8H
Adipose - Visceral (Omentum)	Adipose Tissue	GTEX-18A7B-1226-
SM-718AU
Adipose - Visceral (Omentum)	Adipose Tissue	GTEX-18D9A-0726-
SM-7LTA7
Adipose - Visceral (Omentum)	Adipose Tissue	GTEX-18D9B-1726-
SM-7LTAP
Adipose - Visceral (Omentum)	Adipose Tissue	GTEX-18D9U-0426-
SM-731AN
Adipose - Visceral (Omentum)	Adipose Tissue	GTEX-18QFQ-1326-
SM-731CF
Adipose - Visceral (Omentum)	Adipose Tissue	GTEX-1A32A-0926-
SM-718AB
Adipose - Visceral (Omentum)	Adipose Tissue	GTEX-1A3MV-0626-
SM-731CH
Adipose - Visceral (Omentum)	Adipose Tissue	GTEX-1A3MX-0826-
SM-72D64
Adipose - Visceral (Omentum)	Adipose Tissue	GTEX-1A8G6-2026-
SM-73KYC
Adipose - Visceral (Omentum)	Adipose Tissue	GTEX-1A8G7-1226-
SM-73KYP
Adipose - Visceral (Omentum)	Adipose Tissue	GTEX-1AMEY-0526-
SM-72D62
Adipose - Visceral (Omentum)	Adipose Tissue	GTEX-1AMFI-1026-
SM-73KYM
Adipose - Visceral (Omentum)	Adipose Tissue	GTEX-1AX8Z-1626-
SM-73KWD
Adipose - Visceral (Omentum)	Adipose Tissue	GTEX-1AX9I-1726-
SM-731BX
Adipose - Visceral (Omentum)	Adipose Tissue	GTEX-1AX9J-2226-
SM-731CW
Adipose - Visceral (Omentum)	Adipose Tissue	GTEX-1AX9K-0926-
SM-7939G
Adipose - Visceral (Omentum)	Adipose Tissue	GTEX-1AYCT-1126-
SM-79OO8
Adipose - Visceral (Omentum)	Adipose Tissue	GTEX-1AYD5-0126-
SM-7DUG5
Adipose - Visceral (Omentum)	Adipose Tissue	GTEX-1B8KE-0826-
SM-72D6S
Adipose - Visceral (Omentum)	Adipose Tissue	GTEX-1B8KZ-1026-
SM-73KYN
Adipose - Visceral (Omentum)	Adipose Tissue	GTEX-1B8SF-1726-
SM-73KZ5
Adipose - Visceral (Omentum)	Adipose Tissue	GTEX-1B8SG-1426-
SM-7EWEM
Adipose - Visceral (Omentum)	Adipose Tissue	GTEX-1B932-1726-
SM-793A5
Adipose - Visceral (Omentum)	Adipose Tissue	GTEX-1B933-1326-

SM-7939U
Adipose - Visceral (Omentum)	Adipose Tissue	GTEX-1B97I-0526-
SM-73KXE
Adipose - Visceral (Omentum)	Adipose Tissue	GTEX-1B996-1226-
SM-73KWZ
Adipose - Visceral (Omentum)	Adipose Tissue	GTEX-1BAJH-2126-
SM-7EWFB
Adipose - Visceral (Omentum)	Adipose Tissue	GTEX-1C2JI-0826-
SM-7EPHB
Adipose - Visceral (Omentum)	Adipose Tissue	GTEX-1C64O-1526-
SM-7EWES
Adipose - Visceral (Omentum)	Adipose Tissue	GTEX-1C6VQ-1526-
SM-73KU9
Adipose - Visceral (Omentum)	Adipose Tissue	GTEX-1C6VR-1126-
SM-7EPHD
Adipose - Visceral (Omentum)	Adipose Tissue	GTEX-1C6VS-1826-
SM-73KXN
Adipose - Visceral (Omentum)	Adipose Tissue	GTEX-1CAMR-0326-
SM-793AH
Adipose - Visceral (Omentum)	Adipose Tissue	GTEX-1CAV2-0226-
SM-7IGPQ
Adipose - Visceral (Omentum)	Adipose Tissue	GTEX-1CB4E-0826-
SM-793CS
Adipose - Visceral (Omentum)	Adipose Tissue	GTEX-1CB4F-1126-
SM-79OLG
Adipose - Visceral (Omentum)	Adipose Tissue	GTEX-1CB4G-2126-
SM-7IGQ8
Adipose - Visceral (Omentum)	Adipose Tissue	GTEX-1EH9U-1326-
SM-79OM3
Adipose - Visceral (Omentum)	Adipose Tissue	GTEX-1EN7A-1626-
SM-79OLV
Adipose - Visceral (Omentum)	Adipose Tissue	GTEX-1EU9M-1126-
SM-793DE
Adipose - Visceral (Omentum)	Adipose Tissue	GTEX-1EWIQ-2526-
SM-7EPIJ
Adipose - Visceral (Omentum)	Adipose Tissue	GTEX-U8XE-1926-
SM-3DB98
Adipose - Visceral (Omentum)	Adipose Tissue	GTEX-UPK5-1826-
SM-3GAEB
Adipose - Visceral (Omentum)	Adipose Tissue	GTEX-UTHO-1826-
SM-3GAFE
Adipose - Visceral (Omentum)	Adipose Tissue	GTEX-VUSG-1426-
SM-3GIJN
Adipose - Visceral (Omentum)	Adipose Tissue	GTEX-W5X1-1426-
SM-3GIKH
Adipose - Visceral (Omentum)	Adipose Tissue	GTEX-WEY5-1326-
SM-3GILS
Adipose - Visceral (Omentum)	Adipose Tissue	GTEX-WFJO-1026-
SM-3GIKL
Adipose - Visceral (Omentum)	Adipose Tissue	GTEX-WFON-1226-
SM-3TW8F
Adipose - Visceral (Omentum)	Adipose Tissue	GTEX-WH7G-1126-
SM-3NMBK
Adipose - Visceral (Omentum)	Adipose Tissue	GTEX-WHPG-0626-

SM-3NMBD
Adipose - Visceral (Omentum)   Adipose Tissue   GTEX-WHWD-0826-
SM-3LK6R
Adipose - Visceral (Omentum)   Adipose Tissue   GTEX-WK11-2426-
SM-3NMAA
Adipose - Visceral (Omentum)   Adipose Tissue   GTEX-WL46-2026-
SM-3LK7U
Adipose - Visceral (Omentum)   Adipose Tissue   GTEX-WOFM-0726-
SM-3MJF8
Adipose - Visceral (Omentum)   Adipose Tissue   GTEX-WRHK-0826-
SM-3MJFG
Adipose - Visceral (Omentum)   Adipose Tissue   GTEX-WWTW-1326-
SM-4MVNT
Adipose - Visceral (Omentum)   Adipose Tissue   GTEX-WXYG-0926-
SM-3NB2O
Adipose - Visceral (Omentum)   Adipose Tissue   GTEX-WY7C-0926-
SM-3NB34
Adipose - Visceral (Omentum)   Adipose Tissue   GTEX-WYBS-1026-
SM-4ONCY
Adipose - Visceral (Omentum)   Adipose Tissue   GTEX-WYJK-1426-
SM-3NM8V
Adipose - Visceral (Omentum)   Adipose Tissue   GTEX-WYVS-0626-
SM-4SOJY
Adipose - Visceral (Omentum)   Adipose Tissue   GTEX-X3Y1-0926-
SM-3P5YT
Adipose - Visceral (Omentum)   Adipose Tissue   GTEX-X585-2526-
SM-4QAS1
Adipose - Visceral (Omentum)   Adipose Tissue   GTEX-X8HC-2326-
SM-4QAS4
Adipose - Visceral (Omentum)   Adipose Tissue   GTEX-XAJ8-0226-
SM-4GIB2
Adipose - Visceral (Omentum)   Adipose Tissue   GTEX-XBEC-1826-
SM-4QASN
Adipose - Visceral (Omentum)   Adipose Tissue   GTEX-XBED-1326-
SM-4AT4F
Adipose - Visceral (Omentum)   Adipose Tissue   GTEX-XBEW-1726-
SM-4RGMA
Adipose - Visceral (Omentum)   Adipose Tissue   GTEX-XGQ4-1026-
SM-4AT4L
Adipose - Visceral (Omentum)   Adipose Tissue   GTEX-XLM4-1026-
SM-4AT51
Adipose - Visceral (Omentum)   Adipose Tissue   GTEX-XMK1-0926-
SM-4B66X
Adipose - Visceral (Omentum)   Adipose Tissue   GTEX-XOT4-0826-
SM-4B66Y
Adipose - Visceral (Omentum)   Adipose Tissue   GTEX-XQ3S-2426-
SM-4WAYB
Adipose - Visceral (Omentum)   Adipose Tissue   GTEX-XQ8I-1426-
SM-4BOPW
Adipose - Visceral (Omentum)   Adipose Tissue   GTEX-XUZC-1126-
SM-4BOPZ
Adipose - Visceral (Omentum)   Adipose Tissue   GTEX-XV7Q-1726-
SM-4BRUU
Adipose - Visceral (Omentum)   Adipose Tissue   GTEX-XXEK-1426-

SM-4BRW1
Adipose - Visceral (Omentum)	Adipose Tissue	GTEX-XYKS-1826-
SM-4E3JV
Adipose - Visceral (Omentum)	Adipose Tissue	GTEX-Y111-0926-
SM-4SOIU
Adipose - Visceral (Omentum)	Adipose Tissue	GTEX-Y114-1326-
SM-4TT9A
Adipose - Visceral (Omentum)	Adipose Tissue	GTEX-Y3I4-0726-
SM-4TT7M
Adipose - Visceral (Omentum)	Adipose Tissue	GTEX-Y5LM-0826-
SM-4VDSW
Adipose - Visceral (Omentum)	Adipose Tissue	GTEX-Y5V5-0226-
SM-5S2N8
Adipose - Visceral (Omentum)	Adipose Tissue	GTEX-Y5V6-0626-
SM-4WWFY
Adipose - Visceral (Omentum)	Adipose Tissue	GTEX-Y8E4-1226-
SM-5IFIM
Adipose - Visceral (Omentum)	Adipose Tissue	GTEX-Y8LW-0926-
SM-4VBQ8
Adipose - Visceral (Omentum)	Adipose Tissue	GTEX-Y9LG-1226-
SM-4VBQA
Adipose - Visceral (Omentum)	Adipose Tissue	GTEX-YEC3-0926-
SM-5Q5AC
Adipose - Visceral (Omentum)	Adipose Tissue	GTEX-YEC4-0926-
SM-5PNXY
Adipose - Visceral (Omentum)	Adipose Tissue	GTEX-YECK-1426-
SM-5IFHE
Adipose - Visceral (Omentum)	Adipose Tissue	GTEX-YFCO-1126-
SM-5S2N9
Adipose - Visceral (Omentum)	Adipose Tissue	GTEX-YJ89-1926-
SM-5IFJ6
Adipose - Visceral (Omentum)	Adipose Tissue	GTEX-Z9EW-0826-
SM-5HL9T
Adipose - Visceral (Omentum)	Adipose Tissue	GTEX-ZA64-0926-
SM-5HL7H
Adipose - Visceral (Omentum)	Adipose Tissue	GTEX-ZAB4-1426-
SM-5CVN6
Adipose - Visceral (Omentum)	Adipose Tissue	GTEX-ZAB5-1126-
SM-5Q5AD
Adipose - Visceral (Omentum)	Adipose Tissue	GTEX-ZAJG-1626-
SM-5HL7I
Adipose - Visceral (Omentum)	Adipose Tissue	GTEX-ZC5H-1626-
SM-5HL7L
Adipose - Visceral (Omentum)	Adipose Tissue	GTEX-ZDTS-1826-
SM-5S2N6
Adipose - Visceral (Omentum)	Adipose Tissue	GTEX-ZDTT-0526-
SM-5HL4M
Adipose - Visceral (Omentum)	Adipose Tissue	GTEX-ZDXO-2326-
SM-5LU9W
Adipose - Visceral (Omentum)	Adipose Tissue	GTEX-ZDYS-2226-
SM-5S2MI
Adipose - Visceral (Omentum)	Adipose Tissue	GTEX-ZE7O-1526-
SM-5J1NH
Adipose - Visceral (Omentum)	Adipose Tissue	GTEX-ZE9C-1326-

SM-5S2OW
Adipose - Visceral (Omentum)	Adipose Tissue	GTEX-ZEX8-1226-
SM-5S2OK
Adipose - Visceral (Omentum)	Adipose Tissue	GTEX-ZF28-1426-
SM-5S2OG
Adipose - Visceral (Omentum)	Adipose Tissue	GTEX-ZF29-0826-
SM-5S2NP
Adipose - Visceral (Omentum)	Adipose Tissue	GTEX-ZF2S-1526-
SM-5S2OO
Adipose - Visceral (Omentum)	Adipose Tissue	GTEX-ZF3C-1026-
SM-5S2OC
Adipose - Visceral (Omentum)	Adipose Tissue	GTEX-ZG7Y-1626-
SM-5S2NO
Adipose - Visceral (Omentum)	Adipose Tissue	GTEX-ZGAY-1326-
SM-5S2NS
Adipose - Visceral (Omentum)	Adipose Tissue	GTEX-ZLV1-0926-
SM-5S2MR
Adipose - Visceral (Omentum)	Adipose Tissue	GTEX-ZLWG-0726-
SM-5S2NG
Adipose - Visceral (Omentum)	Adipose Tissue	GTEX-ZP4G-1326-
SM-5EGIW
Adipose - Visceral (Omentum)	Adipose Tissue	GTEX-ZPCL-1026-
SM-5GCOX
Adipose - Visceral (Omentum)	Adipose Tissue	GTEX-ZPIC-0726-
SM-57WFM
Adipose - Visceral (Omentum)	Adipose Tissue	GTEX-ZPU1-1226-
SM-5HL71
Adipose - Visceral (Omentum)	Adipose Tissue	GTEX-ZQG8-1026-
SM-51MR9
Adipose - Visceral (Omentum)	Adipose Tissue	GTEX-ZQUD-0526-
SM-57WG4
Adipose - Visceral (Omentum)	Adipose Tissue	GTEX-ZT9W-1026-
SM-57WG5
Adipose - Visceral (Omentum)	Adipose Tissue	GTEX-ZT9X-0926-
SM-5O9C4
Adipose - Visceral (Omentum)	Adipose Tissue	GTEX-ZTPG-2326-
SM-57WFL
Adipose - Visceral (Omentum)	Adipose Tissue	GTEX-ZTSS-1026-
SM-5985N
Adipose - Visceral (Omentum)	Adipose Tissue	GTEX-ZTX8-0726-
SM-59HL9
Adipose - Visceral (Omentum)	Adipose Tissue	GTEX-ZUA1-2126-
SM-57YRB
Adipose - Visceral (Omentum)	Adipose Tissue	GTEX-ZV6S-1626-
SM-5NQ81
Adipose - Visceral (Omentum)	Adipose Tissue	GTEX-ZV7C-0826-
SM-59HK8
Adipose - Visceral (Omentum)	Adipose Tissue	GTEX-ZVE2-1826-
SM-51MRZ
Adipose - Visceral (Omentum)	Adipose Tissue	GTEX-ZVP2-0226-
SM-57WBD
Adipose - Visceral (Omentum)	Adipose Tissue	GTEX-ZVT2-0226-
SM-5GIE3
Adipose - Visceral (Omentum)	Adipose Tissue	GTEX-ZVZP-1126-

SM-5GICU
Adipose - Visceral (Omentum)	Adipose Tissue	GTEX-ZXES-0926-SM-5E43O
Adipose - Visceral (Omentum)	Adipose Tissue	GTEX-ZXG5-1626-SM-59HJK
Adipose - Visceral (Omentum)	Adipose Tissue	GTEX-ZYFC-1826-SM-5GZZA
Adipose - Visceral (Omentum)	Adipose Tissue	GTEX-ZYFG-1426-SM-5GICS
Adipose - Visceral (Omentum)	Adipose Tissue	GTEX-ZYT6-2126-SM-5GZZ4
Adipose - Visceral (Omentum)	Adipose Tissue	GTEX-ZYVF-1026-SM-5E44V
Adipose - Visceral (Omentum)	Adipose Tissue	GTEX-ZYW4-2226-SM-5GZYG
Adipose - Visceral (Omentum)	Adipose Tissue	GTEX-ZZ64-0626-SM-5GZWR
Adipose - Visceral (Omentum)	Adipose Tissue	GTEX-ZZPU-0326-SM-5N9BJ
Adrenal Gland	Adrenal Gland	GTEX-111CU-0126-SM-5GZWZ
Adrenal Gland	Adrenal Gland	GTEX-111YS-0126-SM-5987T
Adrenal Gland	Adrenal Gland	GTEX-1122O-0326-SM-5H124
Adrenal Gland	Adrenal Gland	GTEX-117YX-0126-SM-5EGH5
Adrenal Gland	Adrenal Gland	GTEX-11DXX-0126-SM-5EGH7
Adrenal Gland	Adrenal Gland	GTEX-11DXY-1626-SM-5H12L
Adrenal Gland	Adrenal Gland	GTEX-11DXZ-0226-SM-5EGGZ
Adrenal Gland	Adrenal Gland	GTEX-11EM3-0326-SM-5A5KJ
Adrenal Gland	Adrenal Gland	GTEX-11EMC-0526-SM-5EGJN
Adrenal Gland	Adrenal Gland	GTEX-11EQ9-0126-SM-5986I
Adrenal Gland	Adrenal Gland	GTEX-11GSP-0326-SM-5A5KW
Adrenal Gland	Adrenal Gland	GTEX-11I78-1826-SM-5A5M4
Adrenal Gland	Adrenal Gland	GTEX-11NSD-0226-SM-5A5LR
Adrenal Gland	Adrenal Gland	GTEX-11P7K-0126-SM-5986E
Adrenal Gland	Adrenal Gland	GTEX-11TT1-0126-SM-5LUAA
Adrenal Gland	Adrenal Gland	GTEX-11XUK-0126-SM-5CVLK
Adrenal Gland	Adrenal Gland	GTEX-1211K-0126-SM-59HJE
Adrenal Gland	Adrenal Gland	GTEX-12696-0626-SM-5EGGD
Adrenal Gland	Adrenal Gland	GTEX-12BJ1-0526-SM-5FQUD
Adrenal Gland	Adrenal Gland	GTEX-12WSD-2126-SM-5LZWK
Adrenal Gland	Adrenal Gland	GTEX-12WSG-0726-SM-5EGIG
Adrenal Gland	Adrenal Gland	GTEX-12WSJ-0126-SM-5GCOM
Adrenal Gland	Adrenal Gland	GTEX-12WSK-0326-SM-5GCOJ
Adrenal Gland	Adrenal Gland	GTEX-12WSL-0326-SM-5CVMK
Adrenal Gland	Adrenal Gland	GTEX-12WSN-0926-SM-5GCN1
Adrenal Gland	Adrenal Gland	GTEX-12ZZZ-0926-SM-5N9EQ
Adrenal Gland	Adrenal Gland	GTEX-13111-0326-SM-5DUXF
Adrenal Gland	Adrenal Gland	GTEX-131XF-0126-SM-5DUVF
Adrenal Gland	Adrenal Gland	GTEX-131XG-1326-SM-5DUX4
Adrenal Gland	Adrenal Gland	GTEX-131YS-2026-SM-5P9J8
Adrenal Gland	Adrenal Gland	GTEX-132AR-1726-SM-5EGHQ
Adrenal Gland	Adrenal Gland	GTEX-132NY-2226-SM-5J2LZ
Adrenal Gland	Adrenal Gland	GTEX-132QS-0126-SM-5IFH9
Adrenal Gland	Adrenal Gland	GTEX-1339X-0826-SM-5J2O6
Adrenal Gland	Adrenal Gland	GTEX-1399R-0326-SM-5KM1X

Adrenal Gland	Adrenal Gland	GTEX-1399S-0426-SM-5IFG5
Adrenal Gland	Adrenal Gland	GTEX-1399U-0226-SM-5P9J2
Adrenal Gland	Adrenal Gland	GTEX-139YR-0126-SM-5IJEY
Adrenal Gland	Adrenal Gland	GTEX-13CF3-0126-SM-5IFFM
Adrenal Gland	Adrenal Gland	GTEX-13D11-0426-SM-5LZYA
Adrenal Gland	Adrenal Gland	GTEX-13FH7-0226-SM-5IFGG
Adrenal Gland	Adrenal Gland	GTEX-13FTW-0126-SM-5IJED
Adrenal Gland	Adrenal Gland	GTEX-13N11-0826-SM-5IJFP
Adrenal Gland	Adrenal Gland	GTEX-13O21-0126-SM-5IJE8
Adrenal Gland	Adrenal Gland	GTEX-13O3O-1626-SM-5KM1O
Adrenal Gland	Adrenal Gland	GTEX-13O61-0126-SM-5KM4P
Adrenal Gland	Adrenal Gland	GTEX-13OVI-1226-SM-5J2ME
Adrenal Gland	Adrenal Gland	GTEX-13OW6-1726-SM-5IJGI
Adrenal Gland	Adrenal Gland	GTEX-13OW7-2726-SM-5L3HN
Adrenal Gland	Adrenal Gland	GTEX-13PL7-1026-SM-5MR5C
Adrenal Gland	Adrenal Gland	GTEX-13PVR-0226-SM-5RQJI
Adrenal Gland	Adrenal Gland	GTEX-13QBU-0526-SM-5IJFE
Adrenal Gland	Adrenal Gland	GTEX-13W3W-1326-SM-5LU4T
Adrenal Gland	Adrenal Gland	GTEX-144GL-1826-SM-79OMB
Adrenal Gland	Adrenal Gland	GTEX-145LT-0126-SM-5S2QJ
Adrenal Gland	Adrenal Gland	GTEX-145MN-0226-SM-5QGPY
Adrenal Gland	Adrenal Gland	GTEX-146FR-1526-SM-5SIB8
Adrenal Gland	Adrenal Gland	GTEX-14753-1726-SM-5NQ9X
Adrenal Gland	Adrenal Gland	GTEX-147F4-2526-SM-5NQBB
Adrenal Gland	Adrenal Gland	GTEX-14A5I-2126-SM-5NQA8
Adrenal Gland	Adrenal Gland	GTEX-14BMU-0126-SM-5S2Q9
Adrenal Gland	Adrenal Gland	GTEX-14C38-1726-SM-5RQJC
Adrenal Gland	Adrenal Gland	GTEX-14C39-2026-SM-664OG
Adrenal Gland	Adrenal Gland	GTEX-14DAQ-1826-SM-5S2VV
Adrenal Gland	Adrenal Gland	GTEX-14DAR-1726-SM-664MZ
Adrenal Gland	Adrenal Gland	GTEX-14E1K-0626-SM-5S2PG
Adrenal Gland	Adrenal Gland	GTEX-14E6E-0126-SM-73KXH
Adrenal Gland	Adrenal Gland	GTEX-14H4A-0226-SM-5SI6Q
Adrenal Gland	Adrenal Gland	GTEX-14JG6-0126-SM-68726
Adrenal Gland	Adrenal Gland	GTEX-14PJ4-0426-SM-6871F
Adrenal Gland	Adrenal Gland	GTEX-14PJ6-0126-SM-686Z5
Adrenal Gland	Adrenal Gland	GTEX-14PK6-0126-SM-664O1
Adrenal Gland	Adrenal Gland	GTEX-14PKV-0726-SM-686ZF
Adrenal Gland	Adrenal Gland	GTEX-14PN3-1426-SM-686Z8
Adrenal Gland	Adrenal Gland	GTEX-14XAO-0326-SM-6ETZN
Adrenal Gland	Adrenal Gland	GTEX-15CHR-0626-SM-7938V
Adrenal Gland	Adrenal Gland	GTEX-15DYW-2126-SM-6M47B
Adrenal Gland	Adrenal Gland	GTEX-15ER7-1126-SM-6M47F
Adrenal Gland	Adrenal Gland	GTEX-15EU6-0326-SM-6M48I
Adrenal Gland	Adrenal Gland	GTEX-15SHU-1926-SM-7KUMO
Adrenal Gland	Adrenal Gland	GTEX-16AAH-0226-SM-793A8
Adrenal Gland	Adrenal Gland	GTEX-16MT8-1626-SM-7EWDW
Adrenal Gland	Adrenal Gland	GTEX-16NGA-0126-SM-72D6U
Adrenal Gland	Adrenal Gland	GTEX-17HGU-0126-SM-7DHLV
Adrenal Gland	Adrenal Gland	GTEX-17HHE-0226-SM-79399
Adrenal Gland	Adrenal Gland	GTEX-17HHY-2226-SM-7KFS4
Adrenal Gland	Adrenal Gland	GTEX-17KNJ-0326-SM-7KFS5
Adrenal Gland	Adrenal Gland	GTEX-18A6Q-1826-SM-72D6I
Adrenal Gland	Adrenal Gland	GTEX-18A7A-1826-SM-7KFTU

Adrenal Gland	Adrenal Gland	GTEX-18D9B-1926-SM-7KFSV
Adrenal Gland	Adrenal Gland	GTEX-1A3MV-0226-SM-731FS
Adrenal Gland	Adrenal Gland	GTEX-1A3MW-2126-SM-731DO
Adrenal Gland	Adrenal Gland	GTEX-1AMFI-0626-SM-731DR
Adrenal Gland	Adrenal Gland	GTEX-1AX8Z-2426-SM-72D7D
Adrenal Gland	Adrenal Gland	GTEX-1AX9J-0626-SM-72D7L
Adrenal Gland	Adrenal Gland	GTEX-1B8KE-0926-SM-7189I
Adrenal Gland	Adrenal Gland	GTEX-1B8KZ-0126-SM-7DHM5
Adrenal Gland	Adrenal Gland	GTEX-1B8L1-1326-SM-7MKG6
Adrenal Gland	Adrenal Gland	GTEX-1BAJH-2626-SM-7EPG6
Adrenal Gland	Adrenal Gland	GTEX-1C475-0126-SM-731DC
Adrenal Gland	Adrenal Gland	GTEX-1CAMR-0126-SM-79391
Adrenal Gland	Adrenal Gland	GTEX-1CAMS-0226-SM-79397
Adrenal Gland	Adrenal Gland	GTEX-1EKGG-2126-SM-7MKFT
Adrenal Gland	Adrenal Gland	GTEX-1F5PL-2326-SM-7MKFC
Adrenal Gland	Adrenal Gland	GTEX-NFK9-1726-SM-3TW8P
Adrenal Gland	Adrenal Gland	GTEX-O5YT-1326-SM-3MJGR
Adrenal Gland	Adrenal Gland	GTEX-O5YV-1126-SM-3LK73
Adrenal Gland	Adrenal Gland	GTEX-OHPK-1326-SM-3MJGN
Adrenal Gland	Adrenal Gland	GTEX-OHPL-1326-SM-3MJGG
Adrenal Gland	Adrenal Gland	GTEX-OIZH-1326-SM-3NB1H
Adrenal Gland	Adrenal Gland	GTEX-OOBK-3125-SM-3LK5R
Adrenal Gland	Adrenal Gland	GTEX-OXRK-0126-SM-3NB1E
Adrenal Gland	Adrenal Gland	GTEX-P4QS-1326-SM-3NMCD
Adrenal Gland	Adrenal Gland	GTEX-P4QT-1326-SM-3NMD3
Adrenal Gland	Adrenal Gland	GTEX-PLZ5-0326-SM-3P614
Adrenal Gland	Adrenal Gland	GTEX-PLZ6-0226-SM-3P61I
Adrenal Gland	Adrenal Gland	GTEX-PW2O-0226-SM-48TC7
Adrenal Gland	Adrenal Gland	GTEX-PWCY-0226-SM-48TD8
Adrenal Gland	Adrenal Gland	GTEX-PX3G-1326-SM-48U18
Adrenal Gland	Adrenal Gland	GTEX-Q2AH-0126-SM-48U2B
Adrenal Gland	Adrenal Gland	GTEX-Q2AI-0226-SM-48U1D
Adrenal Gland	Adrenal Gland	GTEX-Q734-0126-SM-48U1E
Adrenal Gland	Adrenal Gland	GTEX-QCQG-0126-SM-48U27
Adrenal Gland	Adrenal Gland	GTEX-QDVJ-1126-SM-48U1U
Adrenal Gland	Adrenal Gland	GTEX-QDVN-0426-SM-48TZ6
Adrenal Gland	Adrenal Gland	GTEX-QEG5-1826-SM-4R1JP
Adrenal Gland	Adrenal Gland	GTEX-QLQ7-0126-SM-4R1JW
Adrenal Gland	Adrenal Gland	GTEX-QLQW-0226-SM-447BJ
Adrenal Gland	Adrenal Gland	GTEX-QMRM-0126-SM-4R1K9
Adrenal Gland	Adrenal Gland	GTEX-R53T-0226-SM-48FEH
Adrenal Gland	Adrenal Gland	GTEX-R55C-0226-SM-48FEO
Adrenal Gland	Adrenal Gland	GTEX-R55G-0126-SM-48FDS
Adrenal Gland	Adrenal Gland	GTEX-REY6-1726-SM-48FDL
Adrenal Gland	Adrenal Gland	GTEX-RM2N-0126-SM-48FDD
Adrenal Gland	Adrenal Gland	GTEX-RUSQ-0226-SM-47JWT
Adrenal Gland	Adrenal Gland	GTEX-S32W-0126-SM-4AD61
Adrenal Gland	Adrenal Gland	GTEX-S33H-1326-SM-4AD6A
Adrenal Gland	Adrenal Gland	GTEX-S341-0126-SM-4AD64
Adrenal Gland	Adrenal Gland	GTEX-S3XE-0126-SM-4AD4R
Adrenal Gland	Adrenal Gland	GTEX-S4Q7-0226-SM-4AD5O
Adrenal Gland	Adrenal Gland	GTEX-S4Z8-0126-SM-4GICC
Adrenal Gland	Adrenal Gland	GTEX-T6MO-0126-SM-4DM6X
Adrenal Gland	Adrenal Gland	GTEX-TKQ2-0226-SM-4DM6V

Adrenal Gland	Adrenal Gland	GTEX-TMMY-0126-SM-4DXTP
Adrenal Gland	Adrenal Gland	GTEX-U3ZM-0226-SM-4DXTA
Adrenal Gland	Adrenal Gland	GTEX-U3ZN-0826-SM-4DXSZ
Adrenal Gland	Adrenal Gland	GTEX-U4B1-0126-SM-4DXSN
Adrenal Gland	Adrenal Gland	GTEX-UJMC-0226-SM-4IHLH
Adrenal Gland	Adrenal Gland	GTEX-V1D1-0226-SM-4JBHG
Adrenal Gland	Adrenal Gland	GTEX-V955-0126-SM-4JBH5
Adrenal Gland	Adrenal Gland	GTEX-VJYA-0526-SM-4KL1R
Adrenal Gland	Adrenal Gland	GTEX-VUSG-1526-SM-4KKZH
Adrenal Gland	Adrenal Gland	GTEX-W5WG-1126-SM-4LMK4
Adrenal Gland	Adrenal Gland	GTEX-WFG8-0126-SM-4LVMH
Adrenal Gland	Adrenal Gland	GTEX-WHPG-1526-SM-4M1ZK
Adrenal Gland	Adrenal Gland	GTEX-WQUQ-0126-SM-4OOSS
Adrenal Gland	Adrenal Gland	GTEX-WY7C-0326-SM-4OND5
Adrenal Gland	Adrenal Gland	GTEX-WYVS-0226-SM-4ONDK
Adrenal Gland	Adrenal Gland	GTEX-X4LF-0126-SM-4QAS7
Adrenal Gland	Adrenal Gland	GTEX-XMK1-0226-SM-4B65D
Adrenal Gland	Adrenal Gland	GTEX-XQ3S-1726-SM-4BOOD
Adrenal Gland	Adrenal Gland	GTEX-XUZC-0226-SM-4BOO7
Adrenal Gland	Adrenal Gland	GTEX-XV7Q-0226-SM-4BRVL
Adrenal Gland	Adrenal Gland	GTEX-XYKS-0926-SM-4BRVG
Adrenal Gland	Adrenal Gland	GTEX-Y114-0526-SM-4TT8V
Adrenal Gland	Adrenal Gland	GTEX-Y3IK-0226-SM-51MRJ
Adrenal Gland	Adrenal Gland	GTEX-Y5LM-0126-SM-4VBRL
Adrenal Gland	Adrenal Gland	GTEX-Y5V5-1326-SM-4V6G9
Adrenal Gland	Adrenal Gland	GTEX-Y5V6-1926-SM-5IFIL
Adrenal Gland	Adrenal Gland	GTEX-Y9LG-0126-SM-5Q59W
Adrenal Gland	Adrenal Gland	GTEX-YB5E-0226-SM-5IFHI
Adrenal Gland	Adrenal Gland	GTEX-YF7O-0126-SM-5IFIR
Adrenal Gland	Adrenal Gland	GTEX-YFCO-0126-SM-4W1YT
Adrenal Gland	Adrenal Gland	GTEX-ZA64-0226-SM-5HL9C
Adrenal Gland	Adrenal Gland	GTEX-ZDTT-0126-SM-4WKHA
Adrenal Gland	Adrenal Gland	GTEX-ZF2S-0126-SM-4WKFO
Adrenal Gland	Adrenal Gland	GTEX-ZLFU-0826-SM-4WWBP
Adrenal Gland	Adrenal Gland	GTEX-ZLV1-0226-SM-4WWC1
Adrenal Gland	Adrenal Gland	GTEX-ZLWG-0126-SM-4WWC5
Adrenal Gland	Adrenal Gland	GTEX-ZP4G-0126-SM-4YCE3
Adrenal Gland	Adrenal Gland	GTEX-ZT9W-0126-SM-4YCFD
Adrenal Gland	Adrenal Gland	GTEX-ZT9X-0126-SM-4YCFC
Adrenal Gland	Adrenal Gland	GTEX-ZTSS-0326-SM-5987M
Adrenal Gland	Adrenal Gland	GTEX-ZUA1-1926-SM-5E45E
Adrenal Gland	Adrenal Gland	GTEX-ZVP2-0126-SM-5NQ7D
Adrenal Gland	Adrenal Gland	GTEX-ZVT2-0826-SM-5GIEO
Adrenal Gland	Adrenal Gland	GTEX-ZY6K-0126-SM-5SIAM
Adrenal Gland	Adrenal Gland	GTEX-ZYFG-0926-SM-5BC5U
Adrenal Gland	Adrenal Gland	GTEX-ZYVF-1626-SM-5N9EH
Adrenal Gland	Adrenal Gland	GTEX-ZZPU-1226-SM-5N9CK
Artery - Aorta	Blood Vessel	GTEX-111YS-0526-SM-5GZXJ
Artery - Aorta	Blood Vessel	GTEX-1122O-1126-SM-5NQ8X
Artery - Aorta	Blood Vessel	GTEX-1128S-0326-SM-5GZZF
Artery - Aorta	Blood Vessel	GTEX-117XS-0426-SM-5GZZN
Artery - Aorta	Blood Vessel	GTEX-117YW-0226-SM-5N9CM
Artery - Aorta	Blood Vessel	GTEX-11DXX-0426-SM-5EQ5F
Artery - Aorta	Blood Vessel	GTEX-11DXZ-0426-SM-5987Y

Artery - Aorta	Blood Vessel	GTEX-11DYG-1226-SM-5N9DC
Artery - Aorta	Blood Vessel	GTEX-11EM3-0226-SM-5985Y
Artery - Aorta	Blood Vessel	GTEX-11EMC-0926-SM-59863
Artery - Aorta	Blood Vessel	GTEX-11GS4-0326-SM-5N9F7
Artery - Aorta	Blood Vessel	GTEX-11GSP-1126-SM-5A5LM
Artery - Aorta	Blood Vessel	GTEX-11I78-0726-SM-5A5M1
Artery - Aorta	Blood Vessel	GTEX-11LCK-0726-SM-5PNYC
Artery - Aorta	Blood Vessel	GTEX-11NUK-0726-SM-5A5ME
Artery - Aorta	Blood Vessel	GTEX-11ONC-0926-SM-5BC5E
Artery - Aorta	Blood Vessel	GTEX-11P81-0326-SM-5HL6B
Artery - Aorta	Blood Vessel	GTEX-11P82-0126-SM-5HL72
Artery - Aorta	Blood Vessel	GTEX-11TT1-1226-SM-5Q5AV
Artery - Aorta	Blood Vessel	GTEX-11TUW-0926-SM-5EQMW
Artery - Aorta	Blood Vessel	GTEX-11UD2-0826-SM-5EQKS
Artery - Aorta	Blood Vessel	GTEX-11WQK-0326-SM-5EQL1
Artery - Aorta	Blood Vessel	GTEX-11ZTS-0826-SM-5EQ49
Artery - Aorta	Blood Vessel	GTEX-11ZVC-0326-SM-5CVLC
Artery - Aorta	Blood Vessel	GTEX-1211K-0326-SM-5FQT1
Artery - Aorta	Blood Vessel	GTEX-1212Z-0826-SM-5EQ51
Artery - Aorta	Blood Vessel	GTEX-12696-0426-SM-5EGL5
Artery - Aorta	Blood Vessel	GTEX-12C56-0226-SM-5N9FB
Artery - Aorta	Blood Vessel	GTEX-12KS4-0626-SM-5PNY4
Artery - Aorta	Blood Vessel	GTEX-12WSD-1326-SM-5GCNU
Artery - Aorta	Blood Vessel	GTEX-12WSK-0426-SM-5GCNS
Artery - Aorta	Blood Vessel	GTEX-12WSN-0326-SM-5GCP6
Artery - Aorta	Blood Vessel	GTEX-12ZZX-1026-SM-5LZUW
Artery - Aorta	Blood Vessel	GTEX-12ZZY-1326-SM-5GCNW
Artery - Aorta	Blood Vessel	GTEX-13111-0526-SM-5LZVF
Artery - Aorta	Blood Vessel	GTEX-13113-0526-SM-5GCN2
Artery - Aorta	Blood Vessel	GTEX-1313W-1026-SM-5EQ5I
Artery - Aorta	Blood Vessel	GTEX-131XF-0626-SM-5GIDS
Artery - Aorta	Blood Vessel	GTEX-131XW-2226-SM-5PNY1
Artery - Aorta	Blood Vessel	GTEX-131YS-1126-SM-5N9FQ
Artery - Aorta	Blood Vessel	GTEX-132AR-2126-SM-5K7XG
Artery - Aorta	Blood Vessel	GTEX-132QS-0626-SM-62LFM
Artery - Aorta	Blood Vessel	GTEX-1339X-0526-SM-5KLYW
Artery - Aorta	Blood Vessel	GTEX-133LE-0226-SM-5PNY3
Artery - Aorta	Blood Vessel	GTEX-1399R-0626-SM-5K7UZ
Artery - Aorta	Blood Vessel	GTEX-1399T-0526-SM-5J1NC
Artery - Aorta	Blood Vessel	GTEX-1399U-0426-SM-5K7UU
Artery - Aorta	Blood Vessel	GTEX-139D8-1226-SM-5L3ED
Artery - Aorta	Blood Vessel	GTEX-139TS-0226-SM-5K7W5
Artery - Aorta	Blood Vessel	GTEX-139TU-1126-SM-5J1NY
Artery - Aorta	Blood Vessel	GTEX-139UW-0426-SM-5K7V4
Artery - Aorta	Blood Vessel	GTEX-139YR-0626-SM-5IJBT
Artery - Aorta	Blood Vessel	GTEX-13CF3-0526-SM-5IJBV
Artery - Aorta	Blood Vessel	GTEX-13D11-1426-SM-5K7Y9
Artery - Aorta	Blood Vessel	GTEX-13FH7-0326-SM-5J1MR
Artery - Aorta	Blood Vessel	GTEX-13FHO-1626-SM-5IJGB
Artery - Aorta	Blood Vessel	GTEX-13FLV-1426-SM-5J1N4
Artery - Aorta	Blood Vessel	GTEX-13FTX-0226-SM-5K7TX
Artery - Aorta	Blood Vessel	GTEX-13FTZ-0126-SM-5K7V3
Artery - Aorta	Blood Vessel	GTEX-13N2G-0526-SM-5MR5L
Artery - Aorta	Blood Vessel	GTEX-13NYB-0426-SM-5IJDD

Artery - Aorta	Blood Vessel	GTEX-13NZ9-1226-SM-5MR3J
Artery - Aorta	Blood Vessel	GTEX-13O1R-1026-SM-5KM2L
Artery - Aorta	Blood Vessel	GTEX-13O21-0426-SM-5K7VR
Artery - Aorta	Blood Vessel	GTEX-13O3O-1126-SM-5KM4Q
Artery - Aorta	Blood Vessel	GTEX-13O3P-1126-SM-5L3FG
Artery - Aorta	Blood Vessel	GTEX-13O61-0326-SM-5KM2O
Artery - Aorta	Blood Vessel	GTEX-13OVG-0826-SM-5L3D8
Artery - Aorta	Blood Vessel	GTEX-13OW5-0826-SM-5J1NE
Artery - Aorta	Blood Vessel	GTEX-13OW6-1526-SM-5L3HX
Artery - Aorta	Blood Vessel	GTEX-13OW8-1826-SM-5L3H1
Artery - Aorta	Blood Vessel	GTEX-13PVQ-1426-SM-5J1O4
Artery - Aorta	Blood Vessel	GTEX-13PVR-0826-SM-5S2PV
Artery - Aorta	Blood Vessel	GTEX-13QBU-0326-SM-5LU3F
Artery - Aorta	Blood Vessel	GTEX-13RTK-0126-SM-5RQHQ
Artery - Aorta	Blood Vessel	GTEX-13U4I-1026-SM-5LU3G
Artery - Aorta	Blood Vessel	GTEX-13VXT-0826-SM-5SIAP
Artery - Aorta	Blood Vessel	GTEX-13W3W-0226-SM-731ET
Artery - Aorta	Blood Vessel	GTEX-13W46-1826-SM-5K7VA
Artery - Aorta	Blood Vessel	GTEX-13X6I-0926-SM-5LUAB
Artery - Aorta	Blood Vessel	GTEX-13X6K-1926-SM-5LU4O
Artery - Aorta	Blood Vessel	GTEX-13YAN-1526-SM-5LU3E
Artery - Aorta	Blood Vessel	GTEX-144GN-0326-SM-5O9AD
Artery - Aorta	Blood Vessel	GTEX-145MH-1026-SM-5Q5CI
Artery - Aorta	Blood Vessel	GTEX-145MO-1226-SM-5QGPC
Artery - Aorta	Blood Vessel	GTEX-146FH-1326-SM-5NQBI
Artery - Aorta	Blood Vessel	GTEX-146FQ-0826-SM-5LUAJ
Artery - Aorta	Blood Vessel	GTEX-146FR-1026-SM-5NQ9T
Artery - Aorta	Blood Vessel	GTEX-147F4-1026-SM-5Q5F9
Artery - Aorta	Blood Vessel	GTEX-1497J-1026-SM-5NQAV
Artery - Aorta	Blood Vessel	GTEX-14A5I-1326-SM-5SIAQ
Artery - Aorta	Blood Vessel	GTEX-14AS3-0526-SM-5QGQQ
Artery - Aorta	Blood Vessel	GTEX-14ASI-1126-SM-5QGPN
Artery - Aorta	Blood Vessel	GTEX-14B4R-0226-SM-5TDDO
Artery - Aorta	Blood Vessel	GTEX-14BIM-2626-SM-5S2OF
Artery - Aorta	Blood Vessel	GTEX-14BMU-0326-SM-793DO
Artery - Aorta	Blood Vessel	GTEX-14BMV-1226-SM-5TDDY
Artery - Aorta	Blood Vessel	GTEX-14C39-0126-SM-6128R
Artery - Aorta	Blood Vessel	GTEX-14C5O-1526-SM-7DHKT
Artery - Aorta	Blood Vessel	GTEX-14DAQ-1326-SM-5RQIM
Artery - Aorta	Blood Vessel	GTEX-14E1K-0726-SM-62LDU
Artery - Aorta	Blood Vessel	GTEX-14E6C-0626-SM-664NA
Artery - Aorta	Blood Vessel	GTEX-14E7W-1426-SM-6LLJ2
Artery - Aorta	Blood Vessel	GTEX-14ICL-0326-SM-5S2OS
Artery - Aorta	Blood Vessel	GTEX-14PJ2-1226-SM-62LFE
Artery - Aorta	Blood Vessel	GTEX-14PJ3-0226-SM-6EU37
Artery - Aorta	Blood Vessel	GTEX-14PJ4-0826-SM-6LLH5
Artery - Aorta	Blood Vessel	GTEX-14PJ6-0526-SM-6LLIU
Artery - Aorta	Blood Vessel	GTEX-14PJM-1426-SM-62LF3
Artery - Aorta	Blood Vessel	GTEX-14PK6-0626-SM-6AJ9P
Artery - Aorta	Blood Vessel	GTEX-15CHC-0626-SM-686YV
Artery - Aorta	Blood Vessel	GTEX-15DDE-1326-SM-6LLJM
Artery - Aorta	Blood Vessel	GTEX-15DYW-1226-SM-6PALV
Artery - Aorta	Blood Vessel	GTEX-15DZA-1226-SM-7KFS1
Artery - Aorta	Blood Vessel	GTEX-15ER7-2026-SM-793B8

Artery - Aorta	Blood Vessel	GTEX-15ETS-0726-SM-6LPKL
Artery - Aorta	Blood Vessel	GTEX-15F5U-0226-SM-6PANK
Artery - Aorta	Blood Vessel	GTEX-15FZZ-0426-SM-6PANM
Artery - Aorta	Blood Vessel	GTEX-15G19-1026-SM-6PALM
Artery - Aorta	Blood Vessel	GTEX-15RIF-0126-SM-7KUG3
Artery - Aorta	Blood Vessel	GTEX-15RJE-2426-SM-6PAM9
Artery - Aorta	Blood Vessel	GTEX-15SHU-1226-SM-718BY
Artery - Aorta	Blood Vessel	GTEX-15SHV-0826-SM-7KUFA
Artery - Aorta	Blood Vessel	GTEX-15UF6-0226-SM-7KUF6
Artery - Aorta	Blood Vessel	GTEX-16XZZ-1426-SM-7KUE3
Artery - Aorta	Blood Vessel	GTEX-17EVP-0926-SM-7IGOY
Artery - Aorta	Blood Vessel	GTEX-17EVQ-1626-SM-79ONH
Artery - Aorta	Blood Vessel	GTEX-17F96-1126-SM-7EWDF
Artery - Aorta	Blood Vessel	GTEX-17F98-0726-SM-793BJ
Artery - Aorta	Blood Vessel	GTEX-17GQL-2326-SM-731EJ
Artery - Aorta	Blood Vessel	GTEX-17HII-1826-SM-7DUFQ
Artery - Aorta	Blood Vessel	GTEX-17KNJ-0526-SM-7LTA8
Artery - Aorta	Blood Vessel	GTEX-17MF6-1026-SM-7EWE1
Artery - Aorta	Blood Vessel	GTEX-18A66-1326-SM-7LG4M
Artery - Aorta	Blood Vessel	GTEX-18A67-0926-SM-7LG5Y
Artery - Aorta	Blood Vessel	GTEX-18A6Q-1426-SM-7LG63
Artery - Aorta	Blood Vessel	GTEX-18A7A-1426-SM-731AP
Artery - Aorta	Blood Vessel	GTEX-18D9U-1326-SM-731BI
Artery - Aorta	Blood Vessel	GTEX-1A3MV-0426-SM-73KXL
Artery - Aorta	Blood Vessel	GTEX-1AMEY-0726-SM-72D5X
Artery - Aorta	Blood Vessel	GTEX-1AMFI-0426-SM-731C7
Artery - Aorta	Blood Vessel	GTEX-1AX8Z-1226-SM-7DHM7
Artery - Aorta	Blood Vessel	GTEX-1AX9I-1226-SM-731CJ
Artery - Aorta	Blood Vessel	GTEX-1AYCT-0326-SM-73KVN
Artery - Aorta	Blood Vessel	GTEX-1B8KE-0226-SM-7EWEK
Artery - Aorta	Blood Vessel	GTEX-1B8SG-0626-SM-79OL3
Artery - Aorta	Blood Vessel	GTEX-1B933-1126-SM-73KX2
Artery - Aorta	Blood Vessel	GTEX-1C2JI-0426-SM-79OOF
Artery - Aorta	Blood Vessel	GTEX-1C4CL-1226-SM-7DUFT
Artery - Aorta	Blood Vessel	GTEX-1C6VQ-1326-SM-7939X
Artery - Aorta	Blood Vessel	GTEX-1CB4I-1826-SM-793CX
Artery - Aorta	Blood Vessel	GTEX-1EKGG-0826-SM-79OLL
Artery - Aorta	Blood Vessel	GTEX-1EMGI-0926-SM-7IGPV
Artery - Aorta	Blood Vessel	GTEX-1EN7A-1126-SM-7IGQ1
Artery - Aorta	Blood Vessel	GTEX-1EU9M-1726-SM-7EWF3
Artery - Aorta	Blood Vessel	GTEX-N7MT-1426-SM-3LK5M
Artery - Aorta	Blood Vessel	GTEX-NFK9-1326-SM-3LK5I
Artery - Aorta	Blood Vessel	GTEX-NPJ8-0526-SM-3MJHN
Artery - Aorta	Blood Vessel	GTEX-O5YT-0426-SM-3MJHD
Artery - Aorta	Blood Vessel	GTEX-O5YV-0626-SM-3LK64
Artery - Aorta	Blood Vessel	GTEX-O5YW-0426-SM-3MJHJ
Artery - Aorta	Blood Vessel	GTEX-OHPK-0426-SM-3MJH3
Artery - Aorta	Blood Vessel	GTEX-OHPL-0426-SM-3TW8X
Artery - Aorta	Blood Vessel	GTEX-OHPM-0426-SM-3TW8V
Artery - Aorta	Blood Vessel	GTEX-OIZG-0426-SM-3LK5W
Artery - Aorta	Blood Vessel	GTEX-OIZI-1126-SM-3NB1F
Artery - Aorta	Blood Vessel	GTEX-OOBJ-0426-SM-3NB1S
Artery - Aorta	Blood Vessel	GTEX-OOBK-0425-SM-3LK5O
Artery - Aorta	Blood Vessel	GTEX-OXRL-0426-SM-3NM97

Artery - Aorta	Blood Vessel	GTEX-P44H-1026-SM-3NM96
Artery - Aorta	Blood Vessel	GTEX-P4PP-0426-SM-3NM9H
Artery - Aorta	Blood Vessel	GTEX-P4PQ-0426-SM-3NMCI
Artery - Aorta	Blood Vessel	GTEX-P4QS-0426-SM-3NMCQ
Artery - Aorta	Blood Vessel	GTEX-P78B-0826-SM-3NMCA
Artery - Aorta	Blood Vessel	GTEX-PLZ5-0426-SM-3P612
Artery - Aorta	Blood Vessel	GTEX-PLZ6-0326-SM-3P61J
Artery - Aorta	Blood Vessel	GTEX-POMQ-0426-SM-3P61G
Artery - Aorta	Blood Vessel	GTEX-POYW-1126-SM-48TCI
Artery - Aorta	Blood Vessel	GTEX-PSDG-1026-SM-48TCV
Artery - Aorta	Blood Vessel	GTEX-PW2O-0426-SM-48TCC
Artery - Aorta	Blood Vessel	GTEX-PWCY-0326-SM-43IRU
Artery - Aorta	Blood Vessel	GTEX-PX3G-0426-SM-48U1C
Artery - Aorta	Blood Vessel	GTEX-Q2AG-0926-SM-48U1Q
Artery - Aorta	Blood Vessel	GTEX-Q2AH-0326-SM-48U1K
Artery - Aorta	Blood Vessel	GTEX-QCQG-1526-SM-48U25
Artery - Aorta	Blood Vessel	GTEX-QDT8-1026-SM-43V6X
Artery - Aorta	Blood Vessel	GTEX-QDVJ-0626-SM-48U1T
Artery - Aorta	Blood Vessel	GTEX-QDVN-0226-SM-48TZ9
Artery - Aorta	Blood Vessel	GTEX-QEG4-0926-SM-4R1JO
Artery - Aorta	Blood Vessel	GTEX-QEG5-1226-SM-447AR
Artery - Aorta	Blood Vessel	GTEX-QEL4-1126-SM-4R1JJ
Artery - Aorta	Blood Vessel	GTEX-QESD-0426-SM-4R1JZ
Artery - Aorta	Blood Vessel	GTEX-QLQ7-0426-SM-4R1JU
Artery - Aorta	Blood Vessel	GTEX-QMRM-0626-SM-447BQ
Artery - Aorta	Blood Vessel	GTEX-QV31-0326-SM-447BM
Artery - Aorta	Blood Vessel	GTEX-QV44-0626-SM-4R1KJ
Artery - Aorta	Blood Vessel	GTEX-QVJO-0526-SM-447CE
Artery - Aorta	Blood Vessel	GTEX-QVUS-0426-SM-48FE3
Artery - Aorta	Blood Vessel	GTEX-QXCU-0926-SM-48FEP
Artery - Aorta	Blood Vessel	GTEX-R53T-0826-SM-48FCP
Artery - Aorta	Blood Vessel	GTEX-R55D-1026-SM-3GAEQ
Artery - Aorta	Blood Vessel	GTEX-R55E-1126-SM-48FDZ
Artery - Aorta	Blood Vessel	GTEX-R55G-0626-SM-48FDB
Artery - Aorta	Blood Vessel	GTEX-REY6-1126-SM-48FDU
Artery - Aorta	Blood Vessel	GTEX-RUSQ-0326-SM-47JWS
Artery - Aorta	Blood Vessel	GTEX-RWS6-0526-SM-4GIAJ
Artery - Aorta	Blood Vessel	GTEX-RWSA-0926-SM-47JXW
Artery - Aorta	Blood Vessel	GTEX-S32W-0426-SM-4AD6H
Artery - Aorta	Blood Vessel	GTEX-S33H-0726-SM-4AD6M
Artery - Aorta	Blood Vessel	GTEX-S3XE-0326-SM-4AD6L
Artery - Aorta	Blood Vessel	GTEX-S4P3-0326-SM-4AD6P
Artery - Aorta	Blood Vessel	GTEX-S4Z8-0626-SM-4AD6J
Artery - Aorta	Blood Vessel	GTEX-S95S-0226-SM-4B656
Artery - Aorta	Blood Vessel	GTEX-SJXC-1126-SM-4DM6Y
Artery - Aorta	Blood Vessel	GTEX-SNOS-0326-SM-4DM6C
Artery - Aorta	Blood Vessel	GTEX-T5JC-0326-SM-4DM5C
Artery - Aorta	Blood Vessel	GTEX-T6MN-1126-SM-4DM71
Artery - Aorta	Blood Vessel	GTEX-T8EM-0426-SM-4DM7E
Artery - Aorta	Blood Vessel	GTEX-TMMY-1026-SM-4DXTI
Artery - Aorta	Blood Vessel	GTEX-U3ZH-0626-SM-4DXT3
Artery - Aorta	Blood Vessel	GTEX-U3ZM-0326-SM-4DXUJ
Artery - Aorta	Blood Vessel	GTEX-U3ZN-0526-SM-4DXTH
Artery - Aorta	Blood Vessel	GTEX-U8XE-1226-SM-4E3HN

Artery - Aorta	Blood Vessel	GTEX-UJHI-0226-SM-4IHJL
Artery - Aorta	Blood Vessel	GTEX-UJMC-0626-SM-4IHJQ
Artery - Aorta	Blood Vessel	GTEX-UPJH-0926-SM-4IHKA
Artery - Aorta	Blood Vessel	GTEX-UPK5-1526-SM-4JBJA
Artery - Aorta	Blood Vessel	GTEX-V1D1-0326-SM-4JBIY
Artery - Aorta	Blood Vessel	GTEX-V955-0926-SM-4JBJ8
Artery - Aorta	Blood Vessel	GTEX-VJYA-0226-SM-4KL1Q
Artery - Aorta	Blood Vessel	GTEX-VUSG-0526-SM-4KL22
Artery - Aorta	Blood Vessel	GTEX-WFG7-0926-SM-4LMK7
Artery - Aorta	Blood Vessel	GTEX-WFG8-0826-SM-4LVN5
Artery - Aorta	Blood Vessel	GTEX-WFON-0526-SM-4LVLY
Artery - Aorta	Blood Vessel	GTEX-WH7G-0626-SM-4LVMO
Artery - Aorta	Blood Vessel	GTEX-WHSB-0226-SM-4M1XE
Artery - Aorta	Blood Vessel	GTEX-WHWD-0526-SM-4OORW
Artery - Aorta	Blood Vessel	GTEX-WQUQ-1526-SM-4OOSM
Artery - Aorta	Blood Vessel	GTEX-WRHU-1326-SM-4E3K7
Artery - Aorta	Blood Vessel	GTEX-WY7C-0626-SM-4ONCT
Artery - Aorta	Blood Vessel	GTEX-WYJK-1226-SM-4ONCP
Artery - Aorta	Blood Vessel	GTEX-WYVS-0426-SM-4ONDL
Artery - Aorta	Blood Vessel	GTEX-WZTO-1626-SM-4PQYR
Artery - Aorta	Blood Vessel	GTEX-X3Y1-0326-SM-4PQZ7
Artery - Aorta	Blood Vessel	GTEX-X4EO-1026-SM-4QARP
Artery - Aorta	Blood Vessel	GTEX-X4EP-1426-SM-4PQZP
Artery - Aorta	Blood Vessel	GTEX-X4XY-1526-SM-4QASE
Artery - Aorta	Blood Vessel	GTEX-XBED-0726-SM-4GIAR
Artery - Aorta	Blood Vessel	GTEX-XBEW-0526-SM-4QASO
Artery - Aorta	Blood Vessel	GTEX-XPT6-0326-SM-4B66V
Artery - Aorta	Blood Vessel	GTEX-XQ3S-0126-SM-4BOO9
Artery - Aorta	Blood Vessel	GTEX-XQ8I-1326-SM-4BOPV
Artery - Aorta	Blood Vessel	GTEX-XUW1-1126-SM-4BONZ
Artery - Aorta	Blood Vessel	GTEX-XUZC-0626-SM-4BOPG
Artery - Aorta	Blood Vessel	GTEX-XV7Q-0526-SM-4BRWR
Artery - Aorta	Blood Vessel	GTEX-XXEK-0726-SM-4BRWF
Artery - Aorta	Blood Vessel	GTEX-XYKS-0426-SM-4BRW4
Artery - Aorta	Blood Vessel	GTEX-Y114-0426-SM-4TT6V
Artery - Aorta	Blood Vessel	GTEX-Y3I4-0326-SM-4TT28
Artery - Aorta	Blood Vessel	GTEX-Y3IK-0726-SM-4WWE5
Artery - Aorta	Blood Vessel	GTEX-Y5LM-0226-SM-4VBRM
Artery - Aorta	Blood Vessel	GTEX-Y5V5-0426-SM-4VBPU
Artery - Aorta	Blood Vessel	GTEX-Y5V6-0426-SM-4VBRZ
Artery - Aorta	Blood Vessel	GTEX-Y8E4-1126-SM-5IFIA
Artery - Aorta	Blood Vessel	GTEX-Y8LW-0426-SM-4VBQ6
Artery - Aorta	Blood Vessel	GTEX-Y9LG-0226-SM-4VBS4
Artery - Aorta	Blood Vessel	GTEX-YB5K-0326-SM-5IFJQ
Artery - Aorta	Blood Vessel	GTEX-YEC3-0526-SM-5IFHN
Artery - Aorta	Blood Vessel	GTEX-YEC4-0726-SM-5CVLV
Artery - Aorta	Blood Vessel	GTEX-YECK-1326-SM-4W1ZF
Artery - Aorta	Blood Vessel	GTEX-YFC4-0326-SM-4TT3U
Artery - Aorta	Blood Vessel	GTEX-YJ8O-0226-SM-5S2NL
Artery - Aorta	Blood Vessel	GTEX-Z9EW-0126-SM-5CVM6
Artery - Aorta	Blood Vessel	GTEX-ZA64-0126-SM-5HL8Z
Artery - Aorta	Blood Vessel	GTEX-ZAB4-0526-SM-5HL7Z
Artery - Aorta	Blood Vessel	GTEX-ZAJG-1226-SM-5S2MH
Artery - Aorta	Blood Vessel	GTEX-ZAK1-1226-SM-5Q5AA

Artery - Aorta	Blood Vessel	GTEX-ZC5H-1026-SM-5CVN1
Artery - Aorta	Blood Vessel	GTEX-ZDTS-1426-SM-5K7WC
Artery - Aorta	Blood Vessel	GTEX-ZDTT-0226-SM-4WKHM
Artery - Aorta	Blood Vessel	GTEX-ZDXO-2726-SM-4WKFA
Artery - Aorta	Blood Vessel	GTEX-ZDYS-0526-SM-5L3EE
Artery - Aorta	Blood Vessel	GTEX-ZE9C-0726-SM-4WKGA
Artery - Aorta	Blood Vessel	GTEX-ZF28-1026-SM-4WKGV
Artery - Aorta	Blood Vessel	GTEX-ZF29-0326-SM-4WKFB
Artery - Aorta	Blood Vessel	GTEX-ZF2S-0926-SM-4WWBJ
Artery - Aorta	Blood Vessel	GTEX-ZF3C-1426-SM-4WWCD
Artery - Aorta	Blood Vessel	GTEX-ZG7Y-0126-SM-4WWEX
Artery - Aorta	Blood Vessel	GTEX-ZP4G-0526-SM-4YCED
Artery - Aorta	Blood Vessel	GTEX-ZPIC-1026-SM-59HKZ
Artery - Aorta	Blood Vessel	GTEX-ZQUD-0226-SM-4YCEB
Artery - Aorta	Blood Vessel	GTEX-ZVT2-0726-SM-5GIER
Artery - Aorta	Blood Vessel	GTEX-ZVZP-0126-SM-5NQ6Q
Artery - Aorta	Blood Vessel	GTEX-ZYFD-2126-SM-5E43D
Artery - Aorta	Blood Vessel	GTEX-ZYFG-0326-SM-5E45Z
Artery - Aorta	Blood Vessel	GTEX-ZYT6-1026-SM-7LG6X
Artery - Aorta	Blood Vessel	GTEX-ZYVF-2126-SM-5E44N
Artery - Aorta	Blood Vessel	GTEX-ZYW4-1226-SM-5E45B
Artery - Aorta	Blood Vessel	GTEX-ZYY3-1226-SM-5EQKM
Artery - Coronary	Blood Vessel	GTEX-1117F-0626-SM-5N9CS
Artery - Coronary	Blood Vessel	GTEX-1122O-0426-SM-5H12G
Artery - Coronary	Blood Vessel	GTEX-117YX-1726-SM-5GZZS
Artery - Coronary	Blood Vessel	GTEX-11DXX-0826-SM-5GZZP
Artery - Coronary	Blood Vessel	GTEX-11DXY-1126-SM-5987W
Artery - Coronary	Blood Vessel	GTEX-11DXZ-0526-SM-5GU7J
Artery - Coronary	Blood Vessel	GTEX-11DYG-0926-SM-5EGGW
Artery - Coronary	Blood Vessel	GTEX-11EM3-0526-SM-5H12N
Artery - Coronary	Blood Vessel	GTEX-11GS4-0626-SM-5A5LC
Artery - Coronary	Blood Vessel	GTEX-11I78-1026-SM-5HL74
Artery - Coronary	Blood Vessel	GTEX-11ONC-1126-SM-5GU6G
Artery - Coronary	Blood Vessel	GTEX-11TT1-1526-SM-5EQKU
Artery - Coronary	Blood Vessel	GTEX-11UD2-1126-SM-5EQM6
Artery - Coronary	Blood Vessel	GTEX-11ZTS-1026-SM-5LU8O
Artery - Coronary	Blood Vessel	GTEX-11ZTT-0426-SM-5EQLK
Artery - Coronary	Blood Vessel	GTEX-11ZUS-0526-SM-59884
Artery - Coronary	Blood Vessel	GTEX-12696-0526-SM-5EQ3Z
Artery - Coronary	Blood Vessel	GTEX-12WSC-0926-SM-5EQ63
Artery - Coronary	Blood Vessel	GTEX-12WSD-1026-SM-5EQ42
Artery - Coronary	Blood Vessel	GTEX-12WSE-1126-SM-7DUEH
Artery - Coronary	Blood Vessel	GTEX-12WSK-0726-SM-5LZUV
Artery - Coronary	Blood Vessel	GTEX-12WSL-0926-SM-5N9EK
Artery - Coronary	Blood Vessel	GTEX-12ZZX-0626-SM-5EQ46
Artery - Coronary	Blood Vessel	GTEX-131XF-0526-SM-5HL87
Artery - Coronary	Blood Vessel	GTEX-131XG-0826-SM-5LZVS
Artery - Coronary	Blood Vessel	GTEX-131YS-1226-SM-5K7VE
Artery - Coronary	Blood Vessel	GTEX-132AR-1326-SM-5K7YC
Artery - Coronary	Blood Vessel	GTEX-1399R-1826-SM-5J2M9
Artery - Coronary	Blood Vessel	GTEX-1399S-0926-SM-5PNVQ
Artery - Coronary	Blood Vessel	GTEX-1399U-0626-SM-5J1NZ
Artery - Coronary	Blood Vessel	GTEX-139YR-0726-SM-62LFN
Artery - Coronary	Blood Vessel	GTEX-13CF3-0626-SM-5J1NO

Artery - Coronary	Blood Vessel	GTEX-13FH7-0426-SM-5KM4M
Artery - Coronary	Blood Vessel	GTEX-13FHP-1126-SM-5IJDO
Artery - Coronary	Blood Vessel	GTEX-13FTW-0926-SM-5J2NJ
Artery - Coronary	Blood Vessel	GTEX-13FXS-1126-SM-5IJBB
Artery - Coronary	Blood Vessel	GTEX-13N11-0626-SM-5J1OL
Artery - Coronary	Blood Vessel	GTEX-13NZA-1126-SM-5MR4K
Artery - Coronary	Blood Vessel	GTEX-13O21-2926-SM-5J1OU
Artery - Coronary	Blood Vessel	GTEX-13O61-0626-SM-5L3D1
Artery - Coronary	Blood Vessel	GTEX-13OVG-0726-SM-5IJBY
Artery - Coronary	Blood Vessel	GTEX-13PL7-1826-SM-5IJDV
Artery - Coronary	Blood Vessel	GTEX-13PVR-1126-SM-5S2NX
Artery - Coronary	Blood Vessel	GTEX-13QBU-0126-SM-5LU3V
Artery - Coronary	Blood Vessel	GTEX-13QJC-0626-SM-5S2O9
Artery - Coronary	Blood Vessel	GTEX-13RTJ-0626-SM-5S2NB
Artery - Coronary	Blood Vessel	GTEX-13S7M-2226-SM-5S2WB
Artery - Coronary	Blood Vessel	GTEX-13S86-0526-SM-5QGPZ
Artery - Coronary	Blood Vessel	GTEX-13U4I-0926-SM-5LU5A
Artery - Coronary	Blood Vessel	GTEX-13VXU-1926-SM-5LU5J
Artery - Coronary	Blood Vessel	GTEX-13W3W-0626-SM-5LU45
Artery - Coronary	Blood Vessel	GTEX-13X6J-1326-SM-5QGOV
Artery - Coronary	Blood Vessel	GTEX-1445S-1126-SM-5QGOW
Artery - Coronary	Blood Vessel	GTEX-146FH-0926-SM-5SI96
Artery - Coronary	Blood Vessel	GTEX-147F4-1326-SM-5LU9V
Artery - Coronary	Blood Vessel	GTEX-14BMV-0926-SM-7DHKM
Artery - Coronary	Blood Vessel	GTEX-14C38-1226-SM-5S2UK
Artery - Coronary	Blood Vessel	GTEX-14C5O-1426-SM-5RQI8
Artery - Coronary	Blood Vessel	GTEX-14E1K-0926-SM-5S2U4
Artery - Coronary	Blood Vessel	GTEX-14E7W-1026-SM-62LEK
Artery - Coronary	Blood Vessel	GTEX-14H4A-0526-SM-62LEU
Artery - Coronary	Blood Vessel	GTEX-14PJ4-1026-SM-6AJ97
Artery - Coronary	Blood Vessel	GTEX-14PJ6-1226-SM-6LLHJ
Artery - Coronary	Blood Vessel	GTEX-14PJM-1026-SM-6ETZT
Artery - Coronary	Blood Vessel	GTEX-14PN3-0426-SM-6LLIY
Artery - Coronary	Blood Vessel	GTEX-14XAO-1026-SM-6LLJ7
Artery - Coronary	Blood Vessel	GTEX-15CHC-0526-SM-6EU23
Artery - Coronary	Blood Vessel	GTEX-15CHQ-1626-SM-6LLJR
Artery - Coronary	Blood Vessel	GTEX-15CHR-0526-SM-79OLJ
Artery - Coronary	Blood Vessel	GTEX-15DDE-1226-SM-6PAN8
Artery - Coronary	Blood Vessel	GTEX-15ER7-1426-SM-6PAMA
Artery - Coronary	Blood Vessel	GTEX-15RJ7-0526-SM-6PANP
Artery - Coronary	Blood Vessel	GTEX-15SHU-1026-SM-6PAL7
Artery - Coronary	Blood Vessel	GTEX-15SHV-0726-SM-6PAMO
Artery - Coronary	Blood Vessel	GTEX-16YQH-0826-SM-7938J
Artery - Coronary	Blood Vessel	GTEX-17F9E-1226-SM-7EWDG
Artery - Coronary	Blood Vessel	GTEX-17F9Y-1426-SM-7IGOO
Artery - Coronary	Blood Vessel	GTEX-17GQL-0426-SM-7LG5V
Artery - Coronary	Blood Vessel	GTEX-17HII-1126-SM-7IGOP
Artery - Coronary	Blood Vessel	GTEX-18A66-1026-SM-731B8
Artery - Coronary	Blood Vessel	GTEX-18A67-1426-SM-718BC
Artery - Coronary	Blood Vessel	GTEX-18A7A-1126-SM-7LT8O
Artery - Coronary	Blood Vessel	GTEX-18A7B-0926-SM-7189F
Artery - Coronary	Blood Vessel	GTEX-1A32A-1726-SM-731BW
Artery - Coronary	Blood Vessel	GTEX-1A8G6-1726-SM-73KXZ
Artery - Coronary	Blood Vessel	GTEX-1AX8Z-1426-SM-7939T

Artery - Coronary	Blood Vessel	GTEX-1AX9I-0926-SM-73KX8
Artery - Coronary	Blood Vessel	GTEX-1AX9K-0326-SM-7DHM9
Artery - Coronary	Blood Vessel	GTEX-1AYCT-0626-SM-7EPI5
Artery - Coronary	Blood Vessel	GTEX-1B8KE-0426-SM-7189G
Artery - Coronary	Blood Vessel	GTEX-1B8SG-0926-SM-73KV6
Artery - Coronary	Blood Vessel	GTEX-1C64O-1126-SM-7IGMY
Artery - Coronary	Blood Vessel	GTEX-1C6WA-1126-SM-7IGP6
Artery - Coronary	Blood Vessel	GTEX-1CAMS-1226-SM-79393
Artery - Coronary	Blood Vessel	GTEX-1CB4E-0726-SM-7EWEY
Artery - Coronary	Blood Vessel	GTEX-NPJ7-1326-SM-3MJHO
Artery - Coronary	Blood Vessel	GTEX-NPJ8-1326-SM-3LK6B
Artery - Coronary	Blood Vessel	GTEX-O5YV-0426-SM-3LK66
Artery - Coronary	Blood Vessel	GTEX-O5YW-3026-SM-3MJHI
Artery - Coronary	Blood Vessel	GTEX-OHPL-3026-SM-3MJGS
Artery - Coronary	Blood Vessel	GTEX-OIZH-3026-SM-3NB1G
Artery - Coronary	Blood Vessel	GTEX-OOBJ-3026-SM-3NB1D
Artery - Coronary	Blood Vessel	GTEX-P4PP-3026-SM-3P61O
Artery - Coronary	Blood Vessel	GTEX-PSDG-0826-SM-48TCZ
Artery - Coronary	Blood Vessel	GTEX-PWCY-0426-SM-48TCW
Artery - Coronary	Blood Vessel	GTEX-PX3G-3026-SM-48TZR
Artery - Coronary	Blood Vessel	GTEX-Q2AH-0626-SM-48TZJ
Artery - Coronary	Blood Vessel	GTEX-QDVN-0126-SM-4GIC9
Artery - Coronary	Blood Vessel	GTEX-QEG5-1026-SM-4R1KT
Artery - Coronary	Blood Vessel	GTEX-QEL4-1026-SM-4R1JK
Artery - Coronary	Blood Vessel	GTEX-QESD-0326-SM-47J4F
Artery - Coronary	Blood Vessel	GTEX-QLQ7-0326-SM-4R1JV
Artery - Coronary	Blood Vessel	GTEX-QMRM-0426-SM-4R1K2
Artery - Coronary	Blood Vessel	GTEX-QV44-0726-SM-4R1KP
Artery - Coronary	Blood Vessel	GTEX-R53T-0726-SM-48FCS
Artery - Coronary	Blood Vessel	GTEX-R55G-0426-SM-48FDH
Artery - Coronary	Blood Vessel	GTEX-REY6-0926-SM-48FDA
Artery - Coronary	Blood Vessel	GTEX-RN64-0726-SM-48FCV
Artery - Coronary	Blood Vessel	GTEX-RTLS-0726-SM-46MV4
Artery - Coronary	Blood Vessel	GTEX-RU72-0226-SM-46MUD
Artery - Coronary	Blood Vessel	GTEX-RUSQ-0426-SM-47JWR
Artery - Coronary	Blood Vessel	GTEX-RWS6-0426-SM-47JXH
Artery - Coronary	Blood Vessel	GTEX-S3XE-0226-SM-4AD6I
Artery - Coronary	Blood Vessel	GTEX-S4Z8-0926-SM-4AD6Q
Artery - Coronary	Blood Vessel	GTEX-SE5C-0426-SM-4BRUI
Artery - Coronary	Blood Vessel	GTEX-SNMC-0226-SM-4DM6F
Artery - Coronary	Blood Vessel	GTEX-U3ZH-0426-SM-4DXSE
Artery - Coronary	Blood Vessel	GTEX-U3ZN-0426-SM-4DXSH
Artery - Coronary	Blood Vessel	GTEX-U4B1-0226-SM-4DXU8
Artery - Coronary	Blood Vessel	GTEX-U8XE-1026-SM-4E3HM
Artery - Coronary	Blood Vessel	GTEX-UJHI-0326-SM-4IHJE
Artery - Coronary	Blood Vessel	GTEX-UJMC-0426-SM-4IHJF
Artery - Coronary	Blood Vessel	GTEX-UPK5-0126-SM-3GADM
Artery - Coronary	Blood Vessel	GTEX-VUSG-0626-SM-4KL1Z
Artery - Coronary	Blood Vessel	GTEX-WEY5-0326-SM-4LMI7
Artery - Coronary	Blood Vessel	GTEX-WFG7-0626-SM-4LMK6
Artery - Coronary	Blood Vessel	GTEX-WHPG-0726-SM-4M1XX
Artery - Coronary	Blood Vessel	GTEX-WHWD-0226-SM-4OORT
Artery - Coronary	Blood Vessel	GTEX-WRHU-1126-SM-4E3I1
Artery - Coronary	Blood Vessel	GTEX-WY7C-1526-SM-4OND2

Artery - Coronary	Blood Vessel	GTEX-WYJK-0926-SM-4ONCF
Artery - Coronary	Blood Vessel	GTEX-X5EB-0326-SM-4QASF
Artery - Coronary	Blood Vessel	GTEX-XGQ4-0626-SM-4AT56
Artery - Coronary	Blood Vessel	GTEX-XPT6-0426-SM-4B672
Artery - Coronary	Blood Vessel	GTEX-XPVG-0926-SM-4B651
Artery - Coronary	Blood Vessel	GTEX-XQ8I-0326-SM-4BOPN
Artery - Coronary	Blood Vessel	GTEX-XV7Q-0626-SM-4BRV5
Artery - Coronary	Blood Vessel	GTEX-Y114-0326-SM-4TT99
Artery - Coronary	Blood Vessel	GTEX-Y3IK-0926-SM-51MQU
Artery - Coronary	Blood Vessel	GTEX-Y5V6-0326-SM-4VBRW
Artery - Coronary	Blood Vessel	GTEX-Y8E4-0226-SM-4VBQ3
Artery - Coronary	Blood Vessel	GTEX-YB5K-0426-SM-5LUA4
Artery - Coronary	Blood Vessel	GTEX-YEC3-0326-SM-5IFJJ
Artery - Coronary	Blood Vessel	GTEX-YEC4-0426-SM-4W1ZH
Artery - Coronary	Blood Vessel	GTEX-YF7O-0226-SM-5IFJ4
Artery - Coronary	Blood Vessel	GTEX-YFC4-0726-SM-62LF6
Artery - Coronary	Blood Vessel	GTEX-YJ8O-0126-SM-5QGRG
Artery - Coronary	Blood Vessel	GTEX-ZAB4-0726-SM-5HL8I
Artery - Coronary	Blood Vessel	GTEX-ZDYS-0126-SM-5HL6X
Artery - Coronary	Blood Vessel	GTEX-ZF29-0126-SM-4WKGZ
Artery - Coronary	Blood Vessel	GTEX-ZF3C-1326-SM-5A5MF
Artery - Coronary	Blood Vessel	GTEX-ZGAY-0426-SM-4WWAM
Artery - Coronary	Blood Vessel	GTEX-ZLFU-0126-SM-5EQMN
Artery - Coronary	Blood Vessel	GTEX-ZPCL-0326-SM-5FQSG
Artery - Coronary	Blood Vessel	GTEX-ZPU1-0526-SM-4YCDA
Artery - Coronary	Blood Vessel	GTEX-ZQG8-0626-SM-57WF3
Artery - Coronary	Blood Vessel	GTEX-ZT9W-0426-SM-57WF4
Artery - Coronary	Blood Vessel	GTEX-ZUA1-0426-SM-5NQAF
Artery - Coronary	Blood Vessel	GTEX-ZV7C-0426-SM-51MQY
Artery - Coronary	Blood Vessel	GTEX-ZVT2-0526-SM-5E455
Artery - Coronary	Blood Vessel	GTEX-ZYFC-1226-SM-5GIE4
Artery - Coronary	Blood Vessel	GTEX-ZYFG-0126-SM-5GIDH
Artery - Coronary	Blood Vessel	GTEX-ZYT6-0826-SM-5E43I
Artery - Tibial	Blood Vessel	GTEX-1117F-0526-SM-5EGHJ
Artery - Tibial	Blood Vessel	GTEX-111FC-0426-SM-5N9CV
Artery - Tibial	Blood Vessel	GTEX-111YS-2226-SM-5987P
Artery - Tibial	Blood Vessel	GTEX-1128S-2526-SM-5H11N
Artery - Tibial	Blood Vessel	GTEX-113IC-0426-SM-5HL5O
Artery - Tibial	Blood Vessel	GTEX-117YW-2726-SM-5GZZT
Artery - Tibial	Blood Vessel	GTEX-117YX-2626-SM-5EQ53
Artery - Tibial	Blood Vessel	GTEX-11DXW-0526-SM-5H127
Artery - Tibial	Blood Vessel	GTEX-11DXX-2626-SM-5Q5A3
Artery - Tibial	Blood Vessel	GTEX-11DXZ-2626-SM-59882
Artery - Tibial	Blood Vessel	GTEX-11EI6-0526-SM-5985S
Artery - Tibial	Blood Vessel	GTEX-11EM3-2226-SM-5H11Y
Artery - Tibial	Blood Vessel	GTEX-11EMC-3026-SM-5EQ4R
Artery - Tibial	Blood Vessel	GTEX-11EQ8-0426-SM-5N9DP
Artery - Tibial	Blood Vessel	GTEX-11EQ9-2226-SM-5987H
Artery - Tibial	Blood Vessel	GTEX-11GS4-2926-SM-5A5LF
Artery - Tibial	Blood Vessel	GTEX-11GSP-2926-SM-5N9C2
Artery - Tibial	Blood Vessel	GTEX-11I78-2326-SM-5Q5AH
Artery - Tibial	Blood Vessel	GTEX-11LCK-1426-SM-5HL5R
Artery - Tibial	Blood Vessel	GTEX-11NUK-0526-SM-5A5L5
Artery - Tibial	Blood Vessel	GTEX-11NV4-0526-SM-5N9BF

Artery - Tibial	Blood Vessel	GTEX-11O72-0426-SM-5BC5B
Artery - Tibial	Blood Vessel	GTEX-11OC5-0426-SM-5Q5AR
Artery - Tibial	Blood Vessel	GTEX-11ONC-2726-SM-5N9DZ
Artery - Tibial	Blood Vessel	GTEX-11P7K-2326-SM-5EGKZ
Artery - Tibial	Blood Vessel	GTEX-11P81-2726-SM-59876
Artery - Tibial	Blood Vessel	GTEX-11TT1-2726-SM-5LU8L
Artery - Tibial	Blood Vessel	GTEX-11TUW-2926-SM-5BC59
Artery - Tibial	Blood Vessel	GTEX-11UD2-0526-SM-5GU69
Artery - Tibial	Blood Vessel	GTEX-11VI4-2026-SM-5CVLJ
Artery - Tibial	Blood Vessel	GTEX-11WQC-2726-SM-5CVLG
Artery - Tibial	Blood Vessel	GTEX-11XUK-2426-SM-5GU72
Artery - Tibial	Blood Vessel	GTEX-11ZTS-0326-SM-5EQKP
Artery - Tibial	Blood Vessel	GTEX-11ZUS-1326-SM-5FQUC
Artery - Tibial	Blood Vessel	GTEX-11ZVC-2926-SM-5986H
Artery - Tibial	Blood Vessel	GTEX-1211K-2426-SM-59HL8
Artery - Tibial	Blood Vessel	GTEX-12126-0726-SM-5FQTX
Artery - Tibial	Blood Vessel	GTEX-1212Z-2826-SM-59HJ5
Artery - Tibial	Blood Vessel	GTEX-12584-0426-SM-5FQTT
Artery - Tibial	Blood Vessel	GTEX-12696-2726-SM-5FQUL
Artery - Tibial	Blood Vessel	GTEX-1269C-2826-SM-5EQ5O
Artery - Tibial	Blood Vessel	GTEX-12BJ1-2726-SM-5HL9M
Artery - Tibial	Blood Vessel	GTEX-12C56-2026-SM-5FQSI
Artery - Tibial	Blood Vessel	GTEX-12WSC-0526-SM-5BC5X
Artery - Tibial	Blood Vessel	GTEX-12WSH-2826-SM-5GCP5
Artery - Tibial	Blood Vessel	GTEX-12WSK-2726-SM-5CVND
Artery - Tibial	Blood Vessel	GTEX-12WSL-2626-SM-5GCNV
Artery - Tibial	Blood Vessel	GTEX-12WSM-0526-SM-5GCO8
Artery - Tibial	Blood Vessel	GTEX-12WSN-2726-SM-5LZUK
Artery - Tibial	Blood Vessel	GTEX-12ZZW-0426-SM-5GCO9
Artery - Tibial	Blood Vessel	GTEX-12ZZX-0526-SM-5DUW6
Artery - Tibial	Blood Vessel	GTEX-12ZZY-0526-SM-5EQ5H
Artery - Tibial	Blood Vessel	GTEX-13111-2126-SM-5LZU2
Artery - Tibial	Blood Vessel	GTEX-13113-2126-SM-5EQ4U
Artery - Tibial	Blood Vessel	GTEX-1314G-1926-SM-5BC6F
Artery - Tibial	Blood Vessel	GTEX-131XE-2626-SM-5PNYY
Artery - Tibial	Blood Vessel	GTEX-131XF-2526-SM-5EQ68
Artery - Tibial	Blood Vessel	GTEX-131XG-2226-SM-5DUXS
Artery - Tibial	Blood Vessel	GTEX-131XH-2426-SM-5PNZ6
Artery - Tibial	Blood Vessel	GTEX-131XW-0226-SM-5IJCA
Artery - Tibial	Blood Vessel	GTEX-131YS-0426-SM-5EQ4J
Artery - Tibial	Blood Vessel	GTEX-132AR-0726-SM-5EQ6J
Artery - Tibial	Blood Vessel	GTEX-132NY-0626-SM-5L3D9
Artery - Tibial	Blood Vessel	GTEX-132Q8-0526-SM-5IJBQ
Artery - Tibial	Blood Vessel	GTEX-1339X-2626-SM-5K7YX
Artery - Tibial	Blood Vessel	GTEX-133LE-2226-SM-5IFGU
Artery - Tibial	Blood Vessel	GTEX-1399R-2326-SM-5PNYT
Artery - Tibial	Blood Vessel	GTEX-1399S-2626-SM-5L3DU
Artery - Tibial	Blood Vessel	GTEX-1399T-2626-SM-5L3E4
Artery - Tibial	Blood Vessel	GTEX-139T6-1826-SM-5KM2E
Artery - Tibial	Blood Vessel	GTEX-139T8-0426-SM-5K7VC
Artery - Tibial	Blood Vessel	GTEX-139TT-0526-SM-5J1NN
Artery - Tibial	Blood Vessel	GTEX-139UW-2526-SM-62LFO
Artery - Tibial	Blood Vessel	GTEX-139YR-2726-SM-5K7VV
Artery - Tibial	Blood Vessel	GTEX-13CF3-2226-SM-5J2MX

Artery - Tibial	Blood Vessel	GTEX-13D11-2726-SM-5K7V7
Artery - Tibial	Blood Vessel	GTEX-13FH7-2326-SM-5KM1E
Artery - Tibial	Blood Vessel	GTEX-13FHP-0426-SM-5J1O5
Artery - Tibial	Blood Vessel	GTEX-13FTW-2226-SM-5J1OH
Artery - Tibial	Blood Vessel	GTEX-13FTX-2226-SM-5J1OP
Artery - Tibial	Blood Vessel	GTEX-13FTY-0426-SM-5IJC2
Artery - Tibial	Blood Vessel	GTEX-13G51-2926-SM-5IJDL
Artery - Tibial	Blood Vessel	GTEX-13JUV-2426-SM-5J1NS
Artery - Tibial	Blood Vessel	GTEX-13JVG-0426-SM-5MR59
Artery - Tibial	Blood Vessel	GTEX-13N11-2926-SM-5K7VD
Artery - Tibial	Blood Vessel	GTEX-13N1W-0326-SM-5IJDJ
Artery - Tibial	Blood Vessel	GTEX-13NYB-2926-SM-5K7VH
Artery - Tibial	Blood Vessel	GTEX-13NYC-0826-SM-5N9FP
Artery - Tibial	Blood Vessel	GTEX-13NYS-0526-SM-5J1OF
Artery - Tibial	Blood Vessel	GTEX-13NZ8-1626-SM-5IJBC
Artery - Tibial	Blood Vessel	GTEX-13NZ9-0826-SM-5J1NI
Artery - Tibial	Blood Vessel	GTEX-13NZB-2226-SM-5MR5B
Artery - Tibial	Blood Vessel	GTEX-13O21-2526-SM-5J1OZ
Artery - Tibial	Blood Vessel	GTEX-13O3O-0426-SM-5KM2Y
Artery - Tibial	Blood Vessel	GTEX-13O3P-0526-SM-5K7UT
Artery - Tibial	Blood Vessel	GTEX-13O3Q-2526-SM-5IJC4
Artery - Tibial	Blood Vessel	GTEX-13O61-2526-SM-5J1O8
Artery - Tibial	Blood Vessel	GTEX-13OVG-2326-SM-5J1OV
Artery - Tibial	Blood Vessel	GTEX-13OVH-0426-SM-5K7UC
Artery - Tibial	Blood Vessel	GTEX-13OVK-1726-SM-7KUE9
Artery - Tibial	Blood Vessel	GTEX-13OVL-1326-SM-5IJCZ
Artery - Tibial	Blood Vessel	GTEX-13OW6-0326-SM-5L3H9
Artery - Tibial	Blood Vessel	GTEX-13OW7-0626-SM-5K7UD
Artery - Tibial	Blood Vessel	GTEX-13OW8-1626-SM-5L3HW
Artery - Tibial	Blood Vessel	GTEX-13PL6-0526-SM-5L3G6
Artery - Tibial	Blood Vessel	GTEX-13PL7-2126-SM-5L3HZ
Artery - Tibial	Blood Vessel	GTEX-13PLJ-0526-SM-5K7VW
Artery - Tibial	Blood Vessel	GTEX-13PVQ-0526-SM-5LU8Q
Artery - Tibial	Blood Vessel	GTEX-13PVR-2726-SM-5SI6H
Artery - Tibial	Blood Vessel	GTEX-13QBU-2226-SM-5K7WI
Artery - Tibial	Blood Vessel	GTEX-13QIC-1626-SM-5K7TZ
Artery - Tibial	Blood Vessel	GTEX-13QJ3-0626-SM-5SI6G
Artery - Tibial	Blood Vessel	GTEX-13QJC-2526-SM-7DHKJ
Artery - Tibial	Blood Vessel	GTEX-13RTJ-2626-SM-7DHKR
Artery - Tibial	Blood Vessel	GTEX-13RTK-1826-SM-5S2P3
Artery - Tibial	Blood Vessel	GTEX-13RTL-0426-SM-7LG66
Artery - Tibial	Blood Vessel	GTEX-13S86-2526-SM-5S2V5
Artery - Tibial	Blood Vessel	GTEX-13U4I-0426-SM-5LU4W
Artery - Tibial	Blood Vessel	GTEX-13VXT-0526-SM-5SI9O
Artery - Tibial	Blood Vessel	GTEX-13VXU-0526-SM-5K7V1
Artery - Tibial	Blood Vessel	GTEX-13W3W-2826-SM-731E5
Artery - Tibial	Blood Vessel	GTEX-13W46-0626-SM-5LU3N
Artery - Tibial	Blood Vessel	GTEX-13X6H-2526-SM-5QGOT
Artery - Tibial	Blood Vessel	GTEX-13X6K-0526-SM-5LU51
Artery - Tibial	Blood Vessel	GTEX-144GL-0526-SM-5LU41
Artery - Tibial	Blood Vessel	GTEX-144GM-2226-SM-5O9B5
Artery - Tibial	Blood Vessel	GTEX-145LS-0426-SM-5LU8M
Artery - Tibial	Blood Vessel	GTEX-145LU-0826-SM-79OJJ
Artery - Tibial	Blood Vessel	GTEX-145ME-2226-SM-5S2VY

Artery - Tibial	Blood Vessel	GTEX-145MF-0426-SM-5LU9T
Artery - Tibial	Blood Vessel	GTEX-145MH-1526-SM-5LUAU
Artery - Tibial	Blood Vessel	GTEX-145MN-2326-SM-5SIB7
Artery - Tibial	Blood Vessel	GTEX-145MO-0626-SM-5NQAW
Artery - Tibial	Blood Vessel	GTEX-146FH-0426-SM-5QGPS
Artery - Tibial	Blood Vessel	GTEX-146FQ-0526-SM-5LU9I
Artery - Tibial	Blood Vessel	GTEX-146FR-1626-SM-5Q5DS
Artery - Tibial	Blood Vessel	GTEX-14753-0226-SM-5Q5CH
Artery - Tibial	Blood Vessel	GTEX-147F3-0426-SM-5SI8V
Artery - Tibial	Blood Vessel	GTEX-147F4-0126-SM-5QGPV
Artery - Tibial	Blood Vessel	GTEX-147JS-0726-SM-5S2UL
Artery - Tibial	Blood Vessel	GTEX-148VJ-0426-SM-5S2V8
Artery - Tibial	Blood Vessel	GTEX-14A5H-0226-SM-5S2VK
Artery - Tibial	Blood Vessel	GTEX-14A6H-2726-SM-5QGPL
Artery - Tibial	Blood Vessel	GTEX-14AS3-2326-SM-5S2NZ
Artery - Tibial	Blood Vessel	GTEX-14B4R-2026-SM-5QGQL
Artery - Tibial	Blood Vessel	GTEX-14BIN-0926-SM-5S2OZ
Artery - Tibial	Blood Vessel	GTEX-14BMU-2426-SM-5RQIJ
Artery - Tibial	Blood Vessel	GTEX-14BMV-0526-SM-5S2NE
Artery - Tibial	Blood Vessel	GTEX-14C39-2626-SM-5S2QD
Artery - Tibial	Blood Vessel	GTEX-14C5O-0526-SM-62LEI
Artery - Tibial	Blood Vessel	GTEX-14DAQ-0526-SM-793AY
Artery - Tibial	Blood Vessel	GTEX-14DAR-2126-SM-5RQID
Artery - Tibial	Blood Vessel	GTEX-14E1K-2526-SM-5ZZWA
Artery - Tibial	Blood Vessel	GTEX-14E6C-1126-SM-62LDM
Artery - Tibial	Blood Vessel	GTEX-14E6E-2126-SM-664N6
Artery - Tibial	Blood Vessel	GTEX-14E7W-0526-SM-5S2W8
Artery - Tibial	Blood Vessel	GTEX-14ICL-1826-SM-5RQIY
Artery - Tibial	Blood Vessel	GTEX-14JFF-0526-SM-62LFL
Artery - Tibial	Blood Vessel	GTEX-14JG1-0326-SM-62LDO
Artery - Tibial	Blood Vessel	GTEX-14JG6-2026-SM-6EU2E
Artery - Tibial	Blood Vessel	GTEX-14PHY-2726-SM-62LE2
Artery - Tibial	Blood Vessel	GTEX-14PII-0526-SM-6EU2A
Artery - Tibial	Blood Vessel	GTEX-14PJ3-2126-SM-5ZZVL
Artery - Tibial	Blood Vessel	GTEX-14PJ5-1926-SM-6LLIM
Artery - Tibial	Blood Vessel	GTEX-14PJM-0626-SM-686ZN
Artery - Tibial	Blood Vessel	GTEX-14PJN-2226-SM-6872I
Artery - Tibial	Blood Vessel	GTEX-14PK6-2326-SM-69LOG
Artery - Tibial	Blood Vessel	GTEX-14PKU-2226-SM-7LG5T
Artery - Tibial	Blood Vessel	GTEX-14PN4-0426-SM-6AJ9N
Artery - Tibial	Blood Vessel	GTEX-15CHQ-0626-SM-6AJ9E
Artery - Tibial	Blood Vessel	GTEX-15D1Q-1226-SM-686Z1
Artery - Tibial	Blood Vessel	GTEX-15D79-0426-SM-6AJAW
Artery - Tibial	Blood Vessel	GTEX-15DDE-0426-SM-6M48M
Artery - Tibial	Blood Vessel	GTEX-15DZA-2226-SM-7KUMY
Artery - Tibial	Blood Vessel	GTEX-15EOM-0526-SM-7KUFY
Artery - Tibial	Blood Vessel	GTEX-15ETS-2426-SM-6PANJ
Artery - Tibial	Blood Vessel	GTEX-15EU6-0726-SM-7KUFU
Artery - Tibial	Blood Vessel	GTEX-15G1A-2326-SM-6PAMJ
Artery - Tibial	Blood Vessel	GTEX-15RJE-0526-SM-6PAMN
Artery - Tibial	Blood Vessel	GTEX-15SDE-2526-SM-6PAL5
Artery - Tibial	Blood Vessel	GTEX-15SHW-0626-SM-6PALH
Artery - Tibial	Blood Vessel	GTEX-15SKB-0526-SM-6PAMP
Artery - Tibial	Blood Vessel	GTEX-15UF6-0726-SM-6PALI

Artery - Tibial	Blood Vessel	GTEX-15UF7-2326-SM-7KUFO
Artery - Tibial	Blood Vessel	GTEX-16AAH-1926-SM-7LG4C
Artery - Tibial	Blood Vessel	GTEX-16GPK-0426-SM-6PAMW
Artery - Tibial	Blood Vessel	GTEX-16MT8-0126-SM-7DHLD
Artery - Tibial	Blood Vessel	GTEX-16MTA-1926-SM-7KUG1
Artery - Tibial	Blood Vessel	GTEX-16NPV-0526-SM-79OKI
Artery - Tibial	Blood Vessel	GTEX-16XZY-2426-SM-7EPGQ
Artery - Tibial	Blood Vessel	GTEX-16XZZ-0426-SM-793BW
Artery - Tibial	Blood Vessel	GTEX-16YQH-0626-SM-7938I
Artery - Tibial	Blood Vessel	GTEX-178AV-1926-SM-7KUL9
Artery - Tibial	Blood Vessel	GTEX-17EUY-0326-SM-7DUEY
Artery - Tibial	Blood Vessel	GTEX-17EVP-1226-SM-793BF
Artery - Tibial	Blood Vessel	GTEX-17EVQ-1126-SM-7LG58
Artery - Tibial	Blood Vessel	GTEX-17F97-0226-SM-7LG6Q
Artery - Tibial	Blood Vessel	GTEX-17F98-0926-SM-7DHLT
Artery - Tibial	Blood Vessel	GTEX-17F9E-0526-SM-7DUFD
Artery - Tibial	Blood Vessel	GTEX-17F9Y-0826-SM-7LG52
Artery - Tibial	Blood Vessel	GTEX-17GQL-2226-SM-718BB
Artery - Tibial	Blood Vessel	GTEX-17HGU-0526-SM-793BX
Artery - Tibial	Blood Vessel	GTEX-17HHE-2326-SM-793C5
Artery - Tibial	Blood Vessel	GTEX-17HHY-0526-SM-793BZ
Artery - Tibial	Blood Vessel	GTEX-17HII-0626-SM-7LT9H
Artery - Tibial	Blood Vessel	GTEX-17KNJ-2526-SM-7DUFR
Artery - Tibial	Blood Vessel	GTEX-17MF6-0526-SM-7LG4H
Artery - Tibial	Blood Vessel	GTEX-17MFQ-2126-SM-7LG5W
Artery - Tibial	Blood Vessel	GTEX-183FY-0526-SM-7939Z
Artery - Tibial	Blood Vessel	GTEX-18465-0426-SM-7LTAE
Artery - Tibial	Blood Vessel	GTEX-18A66-0526-SM-7LG5O
Artery - Tibial	Blood Vessel	GTEX-18A67-0526-SM-7LT9X
Artery - Tibial	Blood Vessel	GTEX-18A6Q-0526-SM-7LG4O
Artery - Tibial	Blood Vessel	GTEX-18A7A-0526-SM-718BI
Artery - Tibial	Blood Vessel	GTEX-18A7B-0526-SM-7LG68
Artery - Tibial	Blood Vessel	GTEX-18D9A-1626-SM-7LG5Q
Artery - Tibial	Blood Vessel	GTEX-18D9B-0326-SM-731B2
Artery - Tibial	Blood Vessel	GTEX-18D9U-0926-SM-731AZ
Artery - Tibial	Blood Vessel	GTEX-18QFQ-0526-SM-718AV
Artery - Tibial	Blood Vessel	GTEX-1A32A-0426-SM-7939P
Artery - Tibial	Blood Vessel	GTEX-1A3MV-2226-SM-731CP
Artery - Tibial	Blood Vessel	GTEX-1A3MW-0426-SM-731AI
Artery - Tibial	Blood Vessel	GTEX-1A3MX-0426-SM-72D5W
Artery - Tibial	Blood Vessel	GTEX-1A8G6-0226-SM-79ONQ
Artery - Tibial	Blood Vessel	GTEX-1A8G7-0426-SM-731B5
Artery - Tibial	Blood Vessel	GTEX-1AMFI-2426-SM-73KWO
Artery - Tibial	Blood Vessel	GTEX-1AX8Z-0526-SM-7939M
Artery - Tibial	Blood Vessel	GTEX-1AX9K-2626-SM-793CO
Artery - Tibial	Blood Vessel	GTEX-1AYD5-2126-SM-73KWQ
Artery - Tibial	Blood Vessel	GTEX-1B8KE-2326-SM-7939L
Artery - Tibial	Blood Vessel	GTEX-1B8KZ-2226-SM-73KYS
Artery - Tibial	Blood Vessel	GTEX-1B8L1-2826-SM-793D2
Artery - Tibial	Blood Vessel	GTEX-1B8SF-0726-SM-73KYZ
Artery - Tibial	Blood Vessel	GTEX-1B8SG-0526-SM-73KWP
Artery - Tibial	Blood Vessel	GTEX-1B932-0426-SM-73KX4
Artery - Tibial	Blood Vessel	GTEX-1B97I-1926-SM-7DUGC
Artery - Tibial	Blood Vessel	GTEX-1B996-0626-SM-79OJP

Artery - Tibial	Blood Vessel	GTEX-1BAJH-0226-SM-7EPHQ
Artery - Tibial	Blood Vessel	GTEX-1C2JI-1926-SM-73KXO
Artery - Tibial	Blood Vessel	GTEX-1C4CL-0126-SM-7IGN5
Artery - Tibial	Blood Vessel	GTEX-1C64O-0526-SM-793D6
Artery - Tibial	Blood Vessel	GTEX-1C6VQ-0726-SM-7EPI6
Artery - Tibial	Blood Vessel	GTEX-1C6VR-2826-SM-7EWEE
Artery - Tibial	Blood Vessel	GTEX-1CAMQ-0426-SM-7IGPL
Artery - Tibial	Blood Vessel	GTEX-1CB4F-0726-SM-793AO
Artery - Tibial	Blood Vessel	GTEX-N7MS-0626-SM-2YUN7
Artery - Tibial	Blood Vessel	GTEX-NFK9-0526-SM-2YUNL
Artery - Tibial	Blood Vessel	GTEX-NPJ8-1826-SM-2YUNC
Artery - Tibial	Blood Vessel	GTEX-O5YT-1826-SM-32PK9
Artery - Tibial	Blood Vessel	GTEX-O5YV-1626-SM-2YUNJ
Artery - Tibial	Blood Vessel	GTEX-O5YW-1826-SM-2YUN2
Artery - Tibial	Blood Vessel	GTEX-OHPK-1826-SM-2YUMR
Artery - Tibial	Blood Vessel	GTEX-OHPL-1826-SM-2YUN8
Artery - Tibial	Blood Vessel	GTEX-OHPM-1826-SM-2YUNF
Artery - Tibial	Blood Vessel	GTEX-OHPN-2826-SM-3LK67
Artery - Tibial	Blood Vessel	GTEX-OIZH-1826-SM-2YUNP
Artery - Tibial	Blood Vessel	GTEX-OIZI-0526-SM-2XCEG
Artery - Tibial	Blood Vessel	GTEX-OOBJ-1826-SM-3NB1C
Artery - Tibial	Blood Vessel	GTEX-OXRK-0526-SM-3NB2F
Artery - Tibial	Blood Vessel	GTEX-OXRL-1826-SM-2YUMV
Artery - Tibial	Blood Vessel	GTEX-OXRN-1226-SM-2I5EL
Artery - Tibial	Blood Vessel	GTEX-OXRO-1926-SM-2S1O3
Artery - Tibial	Blood Vessel	GTEX-OXRP-2426-SM-2S1NR
Artery - Tibial	Blood Vessel	GTEX-P44H-0626-SM-2XCF2
Artery - Tibial	Blood Vessel	GTEX-P4PP-1826-SM-2S1NT
Artery - Tibial	Blood Vessel	GTEX-P4QS-1826-SM-2S1NI
Artery - Tibial	Blood Vessel	GTEX-P4QT-1826-SM-2S1NJ
Artery - Tibial	Blood Vessel	GTEX-P78B-0726-SM-2S1O2
Artery - Tibial	Blood Vessel	GTEX-PLZ4-1326-SM-2S1O7
Artery - Tibial	Blood Vessel	GTEX-PLZ6-1626-SM-3NB23
Artery - Tibial	Blood Vessel	GTEX-POMQ-2126-SM-2S1OJ
Artery - Tibial	Blood Vessel	GTEX-PSDG-0526-SM-2S1OH
Artery - Tibial	Blood Vessel	GTEX-PVOW-0726-SM-2XCF5
Artery - Tibial	Blood Vessel	GTEX-PW2O-1926-SM-2S1OB
Artery - Tibial	Blood Vessel	GTEX-PWCY-2226-SM-2S1OP
Artery - Tibial	Blood Vessel	GTEX-PWN1-1826-SM-2S1PE
Artery - Tibial	Blood Vessel	GTEX-PX3G-1826-SM-2S1PK
Artery - Tibial	Blood Vessel	GTEX-Q2AG-0526-SM-2S1PW
Artery - Tibial	Blood Vessel	GTEX-Q2AH-2026-SM-2S1PX
Artery - Tibial	Blood Vessel	GTEX-Q2AI-1726-SM-2S1PZ
Artery - Tibial	Blood Vessel	GTEX-Q734-2226-SM-3GAD9
Artery - Tibial	Blood Vessel	GTEX-QCQG-1926-SM-5SI7Q
Artery - Tibial	Blood Vessel	GTEX-QDT8-0426-SM-32PKZ
Artery - Tibial	Blood Vessel	GTEX-QDVJ-2126-SM-5S2U2
Artery - Tibial	Blood Vessel	GTEX-QDVN-2326-SM-2S1PF
Artery - Tibial	Blood Vessel	GTEX-QEG4-1226-SM-2S1P6
Artery - Tibial	Blood Vessel	GTEX-QEG5-0626-SM-2S1PP
Artery - Tibial	Blood Vessel	GTEX-QEL4-0526-SM-3GIJ4
Artery - Tibial	Blood Vessel	GTEX-QLQ7-1926-SM-2S1R6
Artery - Tibial	Blood Vessel	GTEX-QLQW-1526-SM-2S1QV
Artery - Tibial	Blood Vessel	GTEX-QV31-1626-SM-2S1QC

Artery - Tibial	Blood Vessel	GTEX-QV44-2226-SM-447A3
Artery - Tibial	Blood Vessel	GTEX-QXCU-0226-SM-2TC5W
Artery - Tibial	Blood Vessel	GTEX-R53T-2026-SM-3GIJF
Artery - Tibial	Blood Vessel	GTEX-R55C-1826-SM-3GADI
Artery - Tibial	Blood Vessel	GTEX-R55E-0326-SM-48FD2
Artery - Tibial	Blood Vessel	GTEX-RM2N-1826-SM-2TF5B
Artery - Tibial	Blood Vessel	GTEX-RN64-0526-SM-2TC5P
Artery - Tibial	Blood Vessel	GTEX-RNOR-0426-SM-2TF4U
Artery - Tibial	Blood Vessel	GTEX-RTLS-0426-SM-2TF5K
Artery - Tibial	Blood Vessel	GTEX-RU1J-1926-SM-2TF6S
Artery - Tibial	Blood Vessel	GTEX-RU72-1226-SM-2TF6N
Artery - Tibial	Blood Vessel	GTEX-RUSQ-1926-SM-2TF6K
Artery - Tibial	Blood Vessel	GTEX-RVPV-0226-SM-2TF6W
Artery - Tibial	Blood Vessel	GTEX-RWS6-2326-SM-2XCB4
Artery - Tibial	Blood Vessel	GTEX-RWSA-0526-SM-2XCBC
Artery - Tibial	Blood Vessel	GTEX-S32W-2426-SM-2XCAT
Artery - Tibial	Blood Vessel	GTEX-S33H-2426-SM-2XCB2
Artery - Tibial	Blood Vessel	GTEX-S341-2026-SM-2XCAA
Artery - Tibial	Blood Vessel	GTEX-S4Q7-1326-SM-4AD74
Artery - Tibial	Blood Vessel	GTEX-S7PM-0426-SM-3NM91
Artery - Tibial	Blood Vessel	GTEX-S7SE-0526-SM-2XCD8
Artery - Tibial	Blood Vessel	GTEX-S95S-1626-SM-2XCDJ
Artery - Tibial	Blood Vessel	GTEX-SIU8-0426-SM-4BRUE
Artery - Tibial	Blood Vessel	GTEX-SJXC-0426-SM-2XCFH
Artery - Tibial	Blood Vessel	GTEX-SN8G-0526-SM-32PLE
Artery - Tibial	Blood Vessel	GTEX-SNMC-1526-SM-2XCFN
Artery - Tibial	Blood Vessel	GTEX-SNOS-1726-SM-32PLN
Artery - Tibial	Blood Vessel	GTEX-SSA3-0426-SM-32QPI
Artery - Tibial	Blood Vessel	GTEX-T2IS-1026-SM-32QP1
Artery - Tibial	Blood Vessel	GTEX-T5JC-0226-SM-32PMA
Artery - Tibial	Blood Vessel	GTEX-T6MN-0426-SM-32PMF
Artery - Tibial	Blood Vessel	GTEX-T6MO-1926-SM-32QOJ
Artery - Tibial	Blood Vessel	GTEX-T8EM-1626-SM-3DB7K
Artery - Tibial	Blood Vessel	GTEX-TKQ1-1226-SM-4GICJ
Artery - Tibial	Blood Vessel	GTEX-TKQ2-1026-SM-33HB7
Artery - Tibial	Blood Vessel	GTEX-TMMY-0626-SM-33HBD
Artery - Tibial	Blood Vessel	GTEX-TSE9-0426-SM-3DB81
Artery - Tibial	Blood Vessel	GTEX-U3ZH-1726-SM-3DB79
Artery - Tibial	Blood Vessel	GTEX-U3ZN-2526-SM-3DB7V
Artery - Tibial	Blood Vessel	GTEX-U412-0226-SM-3NMC8
Artery - Tibial	Blood Vessel	GTEX-U4B1-1926-SM-3DB9E
Artery - Tibial	Blood Vessel	GTEX-U8T8-0326-SM-3DB93
Artery - Tibial	Blood Vessel	GTEX-U8XE-0626-SM-3DB8U
Artery - Tibial	Blood Vessel	GTEX-UJHI-1926-SM-3DB8Z
Artery - Tibial	Blood Vessel	GTEX-UJMC-2026-SM-3GADR
Artery - Tibial	Blood Vessel	GTEX-UPJH-0726-SM-4IHJW
Artery - Tibial	Blood Vessel	GTEX-V1D1-2626-SM-4JBJH
Artery - Tibial	Blood Vessel	GTEX-V955-2626-SM-3NM9F
Artery - Tibial	Blood Vessel	GTEX-VJWN-0626-SM-3NMAN
Artery - Tibial	Blood Vessel	GTEX-VJYA-1826-SM-4KL1W
Artery - Tibial	Blood Vessel	GTEX-VUSG-2826-SM-4KKZM
Artery - Tibial	Blood Vessel	GTEX-W5WG-2226-SM-4LMI3
Artery - Tibial	Blood Vessel	GTEX-WCDI-0426-SM-4GIAL
Artery - Tibial	Blood Vessel	GTEX-WEY5-2126-SM-3GILK

Artery - Tibial	Blood Vessel	GTEX-WFG7-2326-SM-3GIKV
Artery - Tibial	Blood Vessel	GTEX-WFON-2526-SM-3LK7P
Artery - Tibial	Blood Vessel	GTEX-WHPG-2326-SM-3NMBP
Artery - Tibial	Blood Vessel	GTEX-WHSE-1126-SM-3NMBU
Artery - Tibial	Blood Vessel	GTEX-WHWD-2426-SM-3LK6S
Artery - Tibial	Blood Vessel	GTEX-WL46-0526-SM-3LK7W
Artery - Tibial	Blood Vessel	GTEX-WOFM-1726-SM-3MJFA
Artery - Tibial	Blood Vessel	GTEX-WRHK-1726-SM-3MJFK
Artery - Tibial	Blood Vessel	GTEX-WRHU-0726-SM-3MJFL
Artery - Tibial	Blood Vessel	GTEX-WWYW-0426-SM-3NB31
Artery - Tibial	Blood Vessel	GTEX-WY7C-2826-SM-3NB3Q
Artery - Tibial	Blood Vessel	GTEX-WYBS-0426-SM-3NM9M
Artery - Tibial	Blood Vessel	GTEX-WYJK-0426-SM-3NM9G
Artery - Tibial	Blood Vessel	GTEX-WYVS-2526-SM-3NMAT
Artery - Tibial	Blood Vessel	GTEX-WZTO-1126-SM-3NM93
Artery - Tibial	Blood Vessel	GTEX-X261-0526-SM-4PQYS
Artery - Tibial	Blood Vessel	GTEX-X4EP-0826-SM-3P5YK
Artery - Tibial	Blood Vessel	GTEX-X4XY-0526-SM-46MW1
Artery - Tibial	Blood Vessel	GTEX-X5EB-2626-SM-4E3HZ
Artery - Tibial	Blood Vessel	GTEX-X62O-1626-SM-46MW9
Artery - Tibial	Blood Vessel	GTEX-X88G-0426-SM-47JZ5
Artery - Tibial	Blood Vessel	GTEX-XAJ8-1226-SM-47JYS
Artery - Tibial	Blood Vessel	GTEX-XBEC-0526-SM-4QARU
Artery - Tibial	Blood Vessel	GTEX-XBED-2426-SM-4AT4O
Artery - Tibial	Blood Vessel	GTEX-XBEW-0826-SM-4QASQ
Artery - Tibial	Blood Vessel	GTEX-XGQ4-2426-SM-4AT55
Artery - Tibial	Blood Vessel	GTEX-XLM4-0426-SM-4AT54
Artery - Tibial	Blood Vessel	GTEX-XMK1-2526-SM-4B666
Artery - Tibial	Blood Vessel	GTEX-XOT4-0426-SM-4B66T
Artery - Tibial	Blood Vessel	GTEX-XPT6-2226-SM-4B66R
Artery - Tibial	Blood Vessel	GTEX-XPVG-2926-SM-4B66G
Artery - Tibial	Blood Vessel	GTEX-XQ8I-0826-SM-4BOOE
Artery - Tibial	Blood Vessel	GTEX-XUW1-0726-SM-4BOP5
Artery - Tibial	Blood Vessel	GTEX-XUYS-0426-SM-47JX3
Artery - Tibial	Blood Vessel	GTEX-XUZC-2026-SM-4BRW9
Artery - Tibial	Blood Vessel	GTEX-XYKS-2626-SM-4BRUT
Artery - Tibial	Blood Vessel	GTEX-Y111-0426-SM-4TT3K
Artery - Tibial	Blood Vessel	GTEX-Y114-2726-SM-4TT9B
Artery - Tibial	Blood Vessel	GTEX-Y3IK-2826-SM-4WWDW
Artery - Tibial	Blood Vessel	GTEX-Y5LM-2326-SM-5S2NK
Artery - Tibial	Blood Vessel	GTEX-Y5V5-2726-SM-4VDS2
Artery - Tibial	Blood Vessel	GTEX-Y5V6-2826-SM-4VDSF
Artery - Tibial	Blood Vessel	GTEX-Y8DK-0526-SM-4TT3R
Artery - Tibial	Blood Vessel	GTEX-Y9LG-2326-SM-4VDS1
Artery - Tibial	Blood Vessel	GTEX-YB5E-2426-SM-5IFIV
Artery - Tibial	Blood Vessel	GTEX-YB5K-2526-SM-62LEW
Artery - Tibial	Blood Vessel	GTEX-YBZK-0226-SM-59HLO
Artery - Tibial	Blood Vessel	GTEX-YEC3-1126-SM-5IFIH
Artery - Tibial	Blood Vessel	GTEX-YEC4-2326-SM-5IFHV
Artery - Tibial	Blood Vessel	GTEX-YECK-0526-SM-4W1ZD
Artery - Tibial	Blood Vessel	GTEX-YFC4-0926-SM-5S2U7
Artery - Tibial	Blood Vessel	GTEX-YJ8A-0826-SM-5Q5A7
Artery - Tibial	Blood Vessel	GTEX-YJ8O-2726-SM-5IFJ7
Artery - Tibial	Blood Vessel	GTEX-Z93S-0926-SM-5HL8B

Artery - Tibial Blood Vessel   GTEX-Z93T-0426-SM-5HL6G
Artery - Tibial Blood Vessel   GTEX-Z9EW-1926-SM-5CVMP
Artery - Tibial Blood Vessel   GTEX-ZA64-2226-SM-5Q5A9
Artery - Tibial Blood Vessel   GTEX-ZAB4-1926-SM-57WD8
Artery - Tibial Blood Vessel   GTEX-ZAB5-2026-SM-5L3EQ
Artery - Tibial Blood Vessel   GTEX-ZAK1-0426-SM-5J1MS
Artery - Tibial Blood Vessel   GTEX-ZAKK-0426-SM-5EGHB
Artery - Tibial Blood Vessel   GTEX-ZC5H-0426-SM-5HL9R
Artery - Tibial Blood Vessel   GTEX-ZDTS-0426-SM-4WAYH
Artery - Tibial Blood Vessel   GTEX-ZDTT-2426-SM-4WKFX
Artery - Tibial Blood Vessel   GTEX-ZDYS-1626-SM-4WKGB
Artery - Tibial Blood Vessel   GTEX-ZF2S-2426-SM-5DUXV
Artery - Tibial Blood Vessel   GTEX-ZF3C-0526-SM-4WWBG
Artery - Tibial Blood Vessel   GTEX-ZGAY-0926-SM-4WWBQ
Artery - Tibial Blood Vessel   GTEX-ZLV1-1926-SM-5FQSF
Artery - Tibial Blood Vessel   GTEX-ZLWG-2426-SM-57WG6
Artery - Tibial Blood Vessel   GTEX-ZP4G-2126-SM-57WEA
Artery - Tibial Blood Vessel   GTEX-ZPIC-2426-SM-5DUW1
Artery - Tibial Blood Vessel   GTEX-ZPU1-2726-SM-4WWFT
Artery - Tibial Blood Vessel   GTEX-ZQUD-1326-SM-57WEF
Artery - Tibial Blood Vessel   GTEX-ZT9W-2726-SM-57WG8
Artery - Tibial Blood Vessel   GTEX-ZT9X-2026-SM-57WEJ
Artery - Tibial Blood Vessel   GTEX-ZTPG-0426-SM-5O98K
Artery - Tibial Blood Vessel   GTEX-ZTX8-1826-SM-5N9E5
Artery - Tibial Blood Vessel   GTEX-ZUA1-0626-SM-4YCCW
Artery - Tibial Blood Vessel   GTEX-ZV6S-2026-SM-5NQ8P
Artery - Tibial Blood Vessel   GTEX-ZVE2-0526-SM-5GU7E
Artery - Tibial Blood Vessel   GTEX-ZVP2-2226-SM-5GU6Q
Artery - Tibial Blood Vessel   GTEX-ZVT2-2626-SM-51MTD
Artery - Tibial Blood Vessel   GTEX-ZVT3-0426-SM-5GIDW
Artery - Tibial Blood Vessel   GTEX-ZVTK-0726-SM-5GZX6
Artery - Tibial Blood Vessel   GTEX-ZVZO-0426-SM-5HL4Z
Artery - Tibial Blood Vessel   GTEX-ZVZP-2726-SM-5GZXZ
Artery - Tibial Blood Vessel   GTEX-ZXG5-0526-SM-5NQ7S
Artery - Tibial Blood Vessel   GTEX-ZY6K-1926-SM-5GZXW
Artery - Tibial Blood Vessel   GTEX-ZYFC-0726-SM-5E448
Artery - Tibial Blood Vessel   GTEX-ZYFD-0526-SM-5E442
Artery - Tibial Blood Vessel   GTEX-ZYT6-1526-SM-5E45F
Artery - Tibial Blood Vessel   GTEX-ZYVF-0826-SM-5E447
Artery - Tibial Blood Vessel   GTEX-ZYW4-0426-SM-5GZYS
Artery - Tibial Blood Vessel   GTEX-ZZ64-1326-SM-5E444
Artery - Tibial Blood Vessel   GTEX-ZZPU-2426-SM-5E44I
Bladder Bladder GTEX-S32W-1126-SM-4AD5V
Bladder Bladder GTEX-S3XE-1226-SM-4AD4L
Bladder Bladder GTEX-S4Q7-0926-SM-4AD5D
Bladder Bladder GTEX-S4UY-0926-SM-4AD6O
Bladder Bladder GTEX-SE5C-1026-SM-4BRUG
Bladder Bladder GTEX-SNMC-0826-SM-4DM66
Bladder Bladder GTEX-SNOS-0526-SM-4DM54
Bladder Bladder GTEX-TMMY-1526-SM-4DXST
Bladder Bladder GTEX-U3ZM-0826-SM-4DXU6
Bladder Bladder GTEX-U3ZN-1226-SM-4DXUD
Bladder Bladder GTEX-U4B1-1226-SM-4DXT7
Brain - Amygdala Brain   GTEX-11ZU8-0011-R4a-SM-5BC6Y

Brain - Amygdala	Brain	GTEX-11ZVC-0011-R4a-SM-5BC6Z
Brain - Amygdala	Brain	GTEX-12WSA-0011-R4a-SM-57WB7
Brain - Amygdala	Brain	GTEX-12WSD-0011-R4b-SM-5LZUA
Brain - Amygdala	Brain	GTEX-12WSF-0011-R4b-SM-5HL88
Brain - Amygdala	Brain	GTEX-12WSH-0011-R4a-SM-5GU6K
Brain - Amygdala	Brain	GTEX-12ZZW-0011-R4a-SM-5DUX9
Brain - Amygdala	Brain	GTEX-12ZZZ-0011-R4b-SM-5DUV7
Brain - Amygdala	Brain	GTEX-13112-0011-R4b-SM-5DUXL
Brain - Amygdala	Brain	GTEX-1313W-0011-R4b-SM-5KLZV
Brain - Amygdala	Brain	GTEX-131XH-0011-R4b-SM-5DUWB
Brain - Amygdala	Brain	GTEX-131YS-0011-R4a-SM-5DUVL
Brain - Amygdala	Brain	GTEX-1399T-0011-R4b-SM-5DUVX
Brain - Amygdala	Brain	GTEX-139T8-0011-R4a-SM-5HL54
Brain - Amygdala	Brain	GTEX-13CZV-0011-R4b-SM-5J2NY
Brain - Amygdala	Brain	GTEX-13FLV-0011-R4a-SM-5LZYU
Brain - Amygdala	Brain	GTEX-13JVG-0011-R4a-SM-5MR4C
Brain - Amygdala	Brain	GTEX-13N1W-0011-R4a-SM-5MR52
Brain - Amygdala	Brain	GTEX-13N2G-0011-R4b-SM-5MR54
Brain - Amygdala	Brain	GTEX-13NYS-0011-R4b-SM-5MR3O
Brain - Amygdala	Brain	GTEX-13O3O-0011-R4b-SM-5KM3F
Brain - Amygdala	Brain	GTEX-13O3Q-0011-R4a-SM-5P9H2
Brain - Amygdala	Brain	GTEX-13OVH-0011-R4a-SM-5KM3W
Brain - Amygdala	Brain	GTEX-13OVJ-0011-R4b-SM-5P9H5
Brain - Amygdala	Brain	GTEX-13OVL-0011-R4b-SM-5L3HV
Brain - Amygdala	Brain	GTEX-13OW5-0011-R4b-SM-5L3HS
Brain - Amygdala	Brain	GTEX-13OW6-0011-R4a-SM-5O9DX
Brain - Amygdala	Brain	GTEX-13OW7-0011-R4b-SM-5O9CX
Brain - Amygdala	Brain	GTEX-13PVQ-0011-R4a-SM-5O9CT
Brain - Amygdala	Brain	GTEX-13QIC-0011-R4a-SM-5PNX8
Brain - Amygdala	Brain	GTEX-13RTJ-0011-R4b-SM-5PNX1
Brain - Amygdala	Brain	GTEX-13SLW-0011-R4b-SM-5S2W2
Brain - Amygdala	Brain	GTEX-13SLX-0011-R4a-SM-5O9BJ
Brain - Amygdala	Brain	GTEX-13VXU-0011-R4b-SM-5O9CI
Brain - Amygdala	Brain	GTEX-13X6I-0011-R4b-SM-5PNU9
Brain - Amygdala	Brain	GTEX-13X6J-0011-R4b-SM-5P9K4
Brain - Amygdala	Brain	GTEX-13X6K-0011-R4b-SM-5P9HO
Brain - Amygdala	Brain	GTEX-1445S-0011-R4a-SM-5PNWN
Brain - Amygdala	Brain	GTEX-144GL-0011-R4a-SM-5PNUI
Brain - Amygdala	Brain	GTEX-145LS-0011-R4b-SM-5S2UT
Brain - Amygdala	Brain	GTEX-145MI-0011-R4b-SM-5PNZH
Brain - Amygdala	Brain	GTEX-147GR-0011-R4a-SM-5S2V2
Brain - Amygdala	Brain	GTEX-14ABY-0011-R4b-SM-5S2VN
Brain - Amygdala	Brain	GTEX-14ASI-0011-R4a-SM-69LQ4
Brain - Amygdala	Brain	GTEX-14BIM-0011-R4b-SM-5S2RK
Brain - Amygdala	Brain	GTEX-14DAQ-0011-R4a-SM-5YYAI
Brain - Amygdala	Brain	GTEX-14E7W-0011-R4b-SM-62LEA
Brain - Amygdala	Brain	GTEX-14JG1-0011-R4b-SM-62LEB
Brain - Amygdala	Brain	GTEX-14JIY-0011-R4b-SM-5ZZV7
Brain - Amygdala	Brain	GTEX-14PJM-0011-R4a-SM-5YYAS
Brain - Amygdala	Brain	GTEX-14PN4-0011-R4a-SM-686ZT
Brain - Amygdala	Brain	GTEX-14PQA-0011-R4b-SM-6AJBU
Brain - Amygdala	Brain	GTEX-15CHQ-0011-R4a-SM-686ZX
Brain - Amygdala	Brain	GTEX-15DCD-0011-R4a-SM-69LPB
Brain - Amygdala	Brain	GTEX-15DDE-0011-R4b-SM-7KULI

Brain - Amygdala Brain   GTEX-15DYW-0011-R4a-SM-6M46V
Brain - Amygdala Brain   GTEX-15EO6-0011-R4a-SM-7KULH
Brain - Amygdala Brain   GTEX-15ER7-0011-R4a-SM-6M46S
Brain - Amygdala Brain   GTEX-15SHU-0011-R4b-SM-7IGLC
Brain - Amygdala Brain   GTEX-16YQH-0011-R4a-SM-7EPGI
Brain - Amygdala Brain   GTEX-17EVP-0011-R4a-SM-79OML
Brain - Amygdala Brain   GTEX-17EVQ-0011-R4b-SM-7LT94
Brain - Amygdala Brain   GTEX-17F97-0011-R4b-SM-7KFS2
Brain - Amygdala Brain   GTEX-17HG3-0011-R4a-SM-7IGLL
Brain - Amygdala Brain   GTEX-183WM-0011-R4a-SM-7LG6S
Brain - Amygdala Brain   GTEX-18464-0011-R4a-SM-7LG6T
Brain - Amygdala Brain   GTEX-1A3MX-0011-R4b-SM-7EWFK
Brain - Amygdala Brain   GTEX-1AX9I-0011-R4b-SM-7EPIB
Brain - Amygdala Brain   GTEX-1B8L1-0011-R4a-SM-7EWFJ
Brain - Amygdala Brain   GTEX-N7MS-0011-R4a-SM-2HMKW
Brain - Amygdala Brain   GTEX-N7MT-0011-R4a-SM-2I3G9
Brain - Amygdala Brain   GTEX-NL3H-0011-R4a-SM-2I3GK
Brain - Amygdala Brain   GTEX-NL4W-0011-R4a-SM-2I5GZ
Brain - Amygdala Brain   GTEX-NPJ7-0011-R4a-SM-2I3GJ
Brain - Amygdala Brain   GTEX-NPJ8-0011-R4a-SM-2HML3
Brain - Amygdala Brain   GTEX-OHPN-0011-R4A-SM-2I5FD
Brain - Amygdala Brain   GTEX-P44H-0011-R4A-SM-2XCEW
Brain - Amygdala Brain   GTEX-Q2AG-0011-R4A-SM-2HMKA
Brain - Amygdala Brain   GTEX-QDT8-0011-R4A-SM-32PKM
Brain - Amygdala Brain   GTEX-QMR6-0011-R4A-SM-32PKU
Brain - Amygdala Brain   GTEX-QVJO-0011-R4A-SM-2S1QL
Brain - Amygdala Brain   GTEX-QVUS-0011-R4A-SM-3GAE7
Brain - Amygdala Brain   GTEX-R55E-0011-R4A-SM-2TC5H
Brain - Amygdala Brain   GTEX-RNOR-0011-R4A-SM-3GAD3
Brain - Amygdala Brain   GTEX-S7SE-0011-R4A-SM-2XCDB
Brain - Amygdala Brain   GTEX-T5JC-0011-R4A-SM-32PLT
Brain - Amygdala Brain   GTEX-T6MN-0011-R4A-SM-32QPG
Brain - Amygdala Brain   GTEX-TSE9-0011-R4A-SM-3DB7H
Brain - Amygdala Brain   GTEX-UTHO-0011-R4A-SM-3GIJP
Brain - Amygdala Brain   GTEX-WHSE-0011-R4A-SM-3P5ZN
Brain - Amygdala Brain   GTEX-WVLH-0011-R4A-SM-3MJFS
Brain - Amygdala Brain   GTEX-WZTO-0011-R4A-SM-3NMC7
Brain - Amygdala Brain   GTEX-X4XX-0011-R4B-SM-46MWL
Brain - Amygdala Brain   GTEX-X585-0011-R4B-SM-46MVH
Brain - Amygdala Brain   GTEX-XLM4-0011-R4B-SM-4AT5C
Brain - Amygdala Brain   GTEX-YFC4-0011-R4a-SM-4RGLQ
Brain - Amygdala Brain   GTEX-ZAB4-0011-R4a-SM-4SOKB
Brain - Amygdala Brain   GTEX-ZAK1-0011-R4a-SM-6AJBK
Brain - Amygdala Brain   GTEX-ZE9C-0011-R4a-SM-4WKH6
Brain - Amygdala Brain   GTEX-ZVZQ-0011-R4b-SM-57WCZ
Brain - Anterior cingulate cortex (BA24)   Brain   GTEX-11GSO-0011-R3b-SM-57WB2
Brain - Anterior cingulate cortex (BA24)   Brain   GTEX-11UD1-0011-R3a-SM-5BC6S
Brain - Anterior cingulate cortex (BA24)   Brain   GTEX-11ZTS-0011-R3a-SM-5BC78
Brain - Anterior cingulate cortex (BA24)   Brain   GTEX-11ZU8-0011-R3a-SM-5BC6U
Brain - Anterior cingulate cortex (BA24)   Brain   GTEX-11ZVC-0011-

R3a-SM-5BC6V
Brain - Anterior cingulate cortex (BA24) Brain GTEX-12WSC-0011-R3a-SM-5P9F1
Brain - Anterior cingulate cortex (BA24) Brain GTEX-12WSD-0011-R3b-SM-5LZWN
Brain - Anterior cingulate cortex (BA24) Brain GTEX-12WSF-0011-R3a-SM-5DUW7
Brain - Anterior cingulate cortex (BA24) Brain GTEX-12WSM-0011-R3b-SM-5LZWI
Brain - Anterior cingulate cortex (BA24) Brain GTEX-12ZZW-0011-R3a-SM-5DUWW
Brain - Anterior cingulate cortex (BA24) Brain GTEX-12ZZX-0011-R3b-SM-5EGLH
Brain - Anterior cingulate cortex (BA24) Brain GTEX-12ZZZ-0011-R3b-SM-5DUXK
Brain - Anterior cingulate cortex (BA24) Brain GTEX-13112-0011-R3b-SM-5HL9L
Brain - Anterior cingulate cortex (BA24) Brain GTEX-1313W-0011-R3a-SM-5EGLI
Brain - Anterior cingulate cortex (BA24) Brain GTEX-131XH-0011-R3b-SM-5DUWM
Brain - Anterior cingulate cortex (BA24) Brain GTEX-131YS-0011-R3b-SM-5DUXB
Brain - Anterior cingulate cortex (BA24) Brain GTEX-139T8-0011-R3b-SM-5HL4R
Brain - Anterior cingulate cortex (BA24) Brain GTEX-139TT-0011-R3b-SM-5K7VL
Brain - Anterior cingulate cortex (BA24) Brain GTEX-13CF2-0011-R3b-SM-5LZZ6
Brain - Anterior cingulate cortex (BA24) Brain GTEX-13CIG-0011-R3b-SM-5K7XF
Brain - Anterior cingulate cortex (BA24) Brain GTEX-13CZV-0011-R3a-SM-5KM27
Brain - Anterior cingulate cortex (BA24) Brain GTEX-13FHO-0011-R3b-SM-5K7YA
Brain - Anterior cingulate cortex (BA24) Brain GTEX-13FLV-0011-R3b-SM-5LZYP
Brain - Anterior cingulate cortex (BA24) Brain GTEX-13IVO-0011-R3b-SM-5IJBJ
Brain - Anterior cingulate cortex (BA24) Brain GTEX-13JVG-0011-R3b-SM-5LZXF
Brain - Anterior cingulate cortex (BA24) Brain GTEX-13N1W-0011-R3a-SM-5MR5E
Brain - Anterior cingulate cortex (BA24) Brain GTEX-13N2G-0011-R3b-SM-5MR5G
Brain - Anterior cingulate cortex (BA24) Brain GTEX-13NYS-0011-R3b-SM-5MR41
Brain - Anterior cingulate cortex (BA24) Brain GTEX-13O3O-0011-R3b-SM-5KM3R
Brain - Anterior cingulate cortex (BA24) Brain GTEX-13O3Q-0011-R3a-SM-5P9H3
Brain - Anterior cingulate cortex (BA24) Brain GTEX-13OVH-0011-R3a-SM-5KM49
Brain - Anterior cingulate cortex (BA24) Brain GTEX-13OVJ-0011-

R3b-SM-5P9H6
Brain - Anterior cingulate cortex (BA24) Brain   GTEX-13OVL-0011-R3b-SM-5L3I8
Brain - Anterior cingulate cortex (BA24) Brain   GTEX-13OW5-0011-R3a-SM-5L3I5
Brain - Anterior cingulate cortex (BA24) Brain   GTEX-13OW7-0011-R3a-SM-5O9DA
Brain - Anterior cingulate cortex (BA24) Brain   GTEX-13OW8-0011-R3a-SM-5L3I4
Brain - Anterior cingulate cortex (BA24) Brain   GTEX-13PLJ-0011-R3a-SM-5O9DT
Brain - Anterior cingulate cortex (BA24) Brain   GTEX-13PVQ-0011-R3b-SM-5O9D6
Brain - Anterior cingulate cortex (BA24) Brain   GTEX-13QIC-0011-R3a-SM-5P9HT
Brain - Anterior cingulate cortex (BA24) Brain   GTEX-13RTJ-0011-R3a-SM-5PNX7
Brain - Anterior cingulate cortex (BA24) Brain   GTEX-13RTL-0011-R3a-SM-5P9JP
Brain - Anterior cingulate cortex (BA24) Brain   GTEX-13S7M-0011-R3a-SM-5PNX2
Brain - Anterior cingulate cortex (BA24) Brain   GTEX-13SLW-0011-R3b-SM-5SI6Y
Brain - Anterior cingulate cortex (BA24) Brain   GTEX-13VXU-0011-R3b-SM-5O9CU
Brain - Anterior cingulate cortex (BA24) Brain   GTEX-13X6I-0011-R3b-SM-5P9HM
Brain - Anterior cingulate cortex (BA24) Brain   GTEX-13X6J-0011-R3b-SM-5P9K5
Brain - Anterior cingulate cortex (BA24) Brain   GTEX-13X6K-0011-R3b-SM-5PNUD
Brain - Anterior cingulate cortex (BA24) Brain   GTEX-1445S-0011-R3a-SM-5P9HY
Brain - Anterior cingulate cortex (BA24) Brain   GTEX-144GL-0011-R3a-SM-5P9HP
Brain - Anterior cingulate cortex (BA24) Brain   GTEX-145LS-0011-R3a-SM-5S2VJ
Brain - Anterior cingulate cortex (BA24) Brain   GTEX-145LU-0011-R3a-SM-5P9K2
Brain - Anterior cingulate cortex (BA24) Brain   GTEX-145MG-0011-R3b-SM-5SI67
Brain - Anterior cingulate cortex (BA24) Brain   GTEX-145MH-0011-R3b-SM-5P9JQ
Brain - Anterior cingulate cortex (BA24) Brain   GTEX-145MI-0011-R3b-SM-5PNWK
Brain - Anterior cingulate cortex (BA24) Brain   GTEX-1477Z-0011-R3b-SM-5PNX9
Brain - Anterior cingulate cortex (BA24) Brain   GTEX-148VJ-0011-R3b-SM-69LQJ
Brain - Anterior cingulate cortex (BA24) Brain   GTEX-14BIM-0011-R3b-SM-5S2RL
Brain - Anterior cingulate cortex (BA24) Brain   GTEX-14BIN-0011-R3b-SM-6AJBL
Brain - Anterior cingulate cortex (BA24) Brain   GTEX-14BMV-0011-

R3a-SM-5ZZU7
Brain - Anterior cingulate cortex (BA24) Brain   GTEX-14C39-0011-R3b-SM-5YYA3
Brain - Anterior cingulate cortex (BA24) Brain   GTEX-14DAQ-0011-R3b-SM-5YYAJ
Brain - Anterior cingulate cortex (BA24) Brain   GTEX-14E7W-0011-R3b-SM-5YYB5
Brain - Anterior cingulate cortex (BA24) Brain   GTEX-14JG1-0011-R3a-SM-62LEC
Brain - Anterior cingulate cortex (BA24) Brain   GTEX-14JIY-0011-R3a-SM-5YYAQ
Brain - Anterior cingulate cortex (BA24) Brain   GTEX-14LLW-0011-R3b-SM-62LFW
Brain - Anterior cingulate cortex (BA24) Brain   GTEX-14LZ3-0011-R3a-SM-664NQ
Brain - Anterior cingulate cortex (BA24) Brain   GTEX-14PJM-0011-R3b-SM-5ZZV9
Brain - Anterior cingulate cortex (BA24) Brain   GTEX-14PJO-0011-R3a-SM-6AJBY
Brain - Anterior cingulate cortex (BA24) Brain   GTEX-14PN4-0011-R3b-SM-686ZU
Brain - Anterior cingulate cortex (BA24) Brain   GTEX-14PQA-0011-R3b-SM-69LQO
Brain - Anterior cingulate cortex (BA24) Brain   GTEX-15CHQ-0011-R3b-SM-686ZY
Brain - Anterior cingulate cortex (BA24) Brain   GTEX-15DCD-0011-R3b-SM-6EU1I
Brain - Anterior cingulate cortex (BA24) Brain   GTEX-15DYW-0011-R3b-SM-6LPIM
Brain - Anterior cingulate cortex (BA24) Brain   GTEX-15EO6-0011-R3b-SM-7KUDY
Brain - Anterior cingulate cortex (BA24) Brain   GTEX-15ER7-0011-R3a-SM-6LPIK
Brain - Anterior cingulate cortex (BA24) Brain   GTEX-15G19-0011-R3a-SM-6LPIG
Brain - Anterior cingulate cortex (BA24) Brain   GTEX-15SHU-0011-R3a-SM-7IGPS
Brain - Anterior cingulate cortex (BA24) Brain   GTEX-16XZZ-0011-R3a-SM-7IGLH
Brain - Anterior cingulate cortex (BA24) Brain   GTEX-16YQH-0011-R3a-SM-7EPGM
Brain - Anterior cingulate cortex (BA24) Brain   GTEX-17EVP-0011-R3a-SM-7EPI9
Brain - Anterior cingulate cortex (BA24) Brain   GTEX-17EVQ-0011-R3b-SM-7LT9B
Brain - Anterior cingulate cortex (BA24) Brain   GTEX-17F97-0011-R3b-SM-7LT9E
Brain - Anterior cingulate cortex (BA24) Brain   GTEX-17HHY-0011-R3a-SM-7EWDK
Brain - Anterior cingulate cortex (BA24) Brain   GTEX-17HII-0011-R3a-SM-7LT9D
Brain - Anterior cingulate cortex (BA24) Brain   GTEX-18A6Q-0011-R3a-SM-718A7
Brain - Anterior cingulate cortex (BA24) Brain   GTEX-1A3MX-0011-

R3b-SM-79OOW
Brain - Anterior cingulate cortex (BA24) Brain   GTEX-1B8L1-0011-R3a-SM-7EPIO
Brain - Anterior cingulate cortex (BA24) Brain   GTEX-1BAJH-0011-R3a-SM-7IGNU
Brain - Anterior cingulate cortex (BA24) Brain   GTEX-N7MS-0011-R3a-SM-33HC6
Brain - Anterior cingulate cortex (BA24) Brain   GTEX-N7MT-0011-R3a-SM-2I3GC
Brain - Anterior cingulate cortex (BA24) Brain   GTEX-NL3H-0011-R3a-SM-2I3GL
Brain - Anterior cingulate cortex (BA24) Brain   GTEX-NPJ8-0011-R3a-SM-2HMIW
Brain - Anterior cingulate cortex (BA24) Brain   GTEX-OHPN-0011-R3A-SM-2I5FC
Brain - Anterior cingulate cortex (BA24) Brain   GTEX-P44H-0011-R3A-SM-5SI8A
Brain - Anterior cingulate cortex (BA24) Brain   GTEX-PVOW-0011-R3A-SM-32PKX
Brain - Anterior cingulate cortex (BA24) Brain   GTEX-PWO3-0011-R3A-SM-2I5EX
Brain - Anterior cingulate cortex (BA24) Brain   GTEX-Q2AG-0011-R3A-SM-2HMJ9
Brain - Anterior cingulate cortex (BA24) Brain   GTEX-QDT8-0011-R3A-SM-32PKR
Brain - Anterior cingulate cortex (BA24) Brain   GTEX-QVUS-0011-R3A-SM-3GAFD
Brain - Anterior cingulate cortex (BA24) Brain   GTEX-RNOR-0011-R3A-SM-5SI8E
Brain - Anterior cingulate cortex (BA24) Brain   GTEX-RVPV-0011-R3A-SM-2TF63
Brain - Anterior cingulate cortex (BA24) Brain   GTEX-T2IS-0011-R3A-SM-32QPB
Brain - Anterior cingulate cortex (BA24) Brain   GTEX-UTHO-0011-R3A-SM-3GIK8
Brain - Anterior cingulate cortex (BA24) Brain   GTEX-WHSE-0011-R3A-SM-3P5ZM
Brain - Anterior cingulate cortex (BA24) Brain   GTEX-WL46-0011-R3A-SM-3TW8E
Brain - Anterior cingulate cortex (BA24) Brain   GTEX-WZTO-0011-R3B-SM-3NMC6
Brain - Anterior cingulate cortex (BA24) Brain   GTEX-X4XX-0011-R3B-SM-46MWK
Brain - Anterior cingulate cortex (BA24) Brain   GTEX-X585-0011-R3B-SM-46MVG
Brain - Anterior cingulate cortex (BA24) Brain   GTEX-XLM4-0011-R3B-SM-4AT6E
Brain - Anterior cingulate cortex (BA24) Brain   GTEX-XMD1-0011-R3B-SM-4AT5R
Brain - Anterior cingulate cortex (BA24) Brain   GTEX-Y8DK-0011-R3A-SM-4RTW5
Brain - Anterior cingulate cortex (BA24) Brain   GTEX-YFC4-0011-R3a-SM-4RGLP
Brain - Anterior cingulate cortex (BA24) Brain   GTEX-YJ89-0011-

R3b-SM-4V6EF
Brain - Anterior cingulate cortex (BA24) Brain   GTEX-Z93S-0011-R3b-SM-4RGNH
Brain - Anterior cingulate cortex (BA24) Brain   GTEX-ZAB4-0011-R3a-SM-4RGNQ
Brain - Anterior cingulate cortex (BA24) Brain   GTEX-ZDXO-0011-R3a-SM-4WKFS
Brain - Anterior cingulate cortex (BA24) Brain   GTEX-ZE9C-0011-R3a-SM-4WKGT
Brain - Anterior cingulate cortex (BA24) Brain   GTEX-ZF28-0011-R3a-SM-4WWDA
Brain - Anterior cingulate cortex (BA24) Brain   GTEX-ZUA1-0011-R3a-SM-4YCEE
Brain - Anterior cingulate cortex (BA24) Brain   GTEX-ZVT3-0011-R3b-SM-51MTJ
Brain - Anterior cingulate cortex (BA24) Brain   GTEX-ZVZQ-0011-R3a-SM-57WC7
Brain - Caudate (basal ganglia)   Brain   GTEX-11GSP-0011-R5a-SM-57WBE
Brain - Caudate (basal ganglia)   Brain   GTEX-11H98-0011-R5b-SM-57WCF
Brain - Caudate (basal ganglia)   Brain   GTEX-11NV4-0011-R5a-SM-5NQ88
Brain - Caudate (basal ganglia)   Brain   GTEX-11OF3-0011-R5a-SM-57WB4
Brain - Caudate (basal ganglia)   Brain   GTEX-11TUW-0011-R5b-SM-5BC6Q
Brain - Caudate (basal ganglia)   Brain   GTEX-11UD1-0011-R5b-SM-5P9FP
Brain - Caudate (basal ganglia)   Brain   GTEX-11WQC-0011-R5a-SM-5BC74
Brain - Caudate (basal ganglia)   Brain   GTEX-12WSA-0011-R5b-SM-5GU5I
Brain - Caudate (basal ganglia)   Brain   GTEX-12WSC-0011-R5b-SM-5CVNS
Brain - Caudate (basal ganglia)   Brain   GTEX-12WSD-0011-R5b-SM-5CVNG
Brain - Caudate (basal ganglia)   Brain   GTEX-12WSF-0011-R5a-SM-5HL8K
Brain - Caudate (basal ganglia)   Brain   GTEX-12WSH-0011-R5b-SM-5P9F3
Brain - Caudate (basal ganglia)   Brain   GTEX-12WSM-0011-R5a-SM-5EQ4Y
Brain - Caudate (basal ganglia)   Brain   GTEX-12ZZW-0011-R5a-SM-5DUVN
Brain - Caudate (basal ganglia)   Brain   GTEX-12ZZX-0011-R5a-SM-5HL89
Brain - Caudate (basal ganglia)   Brain   GTEX-12ZZY-0011-R5a-SM-5EGL9
Brain - Caudate (basal ganglia)   Brain   GTEX-12ZZZ-0011-R5a-SM-5EQ4M
Brain - Caudate (basal ganglia)   Brain   GTEX-13112-0011-R5b-SM-5DUV8
Brain - Caudate (basal ganglia)   Brain   GTEX-1313W-0011-R5b-

SM-5L3EP
Brain - Caudate (basal ganglia)   Brain   GTEX-131YS-0011-R5b-SM-5K7YB
Brain - Caudate (basal ganglia)   Brain   GTEX-132Q8-0011-R5a-SM-5IJDM
Brain - Caudate (basal ganglia)   Brain   GTEX-1399T-0011-R5b-SM-5IJDK
Brain - Caudate (basal ganglia)   Brain   GTEX-139T8-0011-R5b-SM-5J1MO
Brain - Caudate (basal ganglia)   Brain   GTEX-139TS-0011-R5b-SM-5J2MZ
Brain - Caudate (basal ganglia)   Brain   GTEX-139TT-0011-R5b-SM-5K7W8
Brain - Caudate (basal ganglia)   Brain   GTEX-139TU-0011-R5b-SM-5L3E1
Brain - Caudate (basal ganglia)   Brain   GTEX-13CF2-0011-R5a-SM-5LZWS
Brain - Caudate (basal ganglia)   Brain   GTEX-13CIG-0011-R5b-SM-5L3F2
Brain - Caudate (basal ganglia)   Brain   GTEX-13CZV-0011-R5a-SM-5K7XA
Brain - Caudate (basal ganglia)   Brain   GTEX-13FHO-0011-R5b-SM-5LZZ3
Brain - Caudate (basal ganglia)   Brain   GTEX-13FHP-0011-R5b-SM-5LZY2
Brain - Caudate (basal ganglia)   Brain   GTEX-13FLV-0011-R5a-SM-5LZWT
Brain - Caudate (basal ganglia)   Brain   GTEX-13FLW-0011-R5a-SM-5LZX6
Brain - Caudate (basal ganglia)   Brain   GTEX-13FXS-0011-R5b-SM-5LZYE
Brain - Caudate (basal ganglia)   Brain   GTEX-13G51-0011-R5a-SM-5L3EB
Brain - Caudate (basal ganglia)   Brain   GTEX-13IVO-0011-R5a-SM-5L3CY
Brain - Caudate (basal ganglia)   Brain   GTEX-13JUV-0011-R5a-SM-5LZY5
Brain - Caudate (basal ganglia)   Brain   GTEX-13JVG-0011-R5a-SM-5MR4O
Brain - Caudate (basal ganglia)   Brain   GTEX-13N1W-0011-R5b-SM-5MR5Q
Brain - Caudate (basal ganglia)   Brain   GTEX-13N2G-0011-R5a-SM-5MR33
Brain - Caudate (basal ganglia)   Brain   GTEX-13NYB-0011-R5a-SM-5MR45
Brain - Caudate (basal ganglia)   Brain   GTEX-13NYS-0011-R5a-SM-5MR4D
Brain - Caudate (basal ganglia)   Brain   GTEX-13NZA-0011-R5b-SM-5KM4K
Brain - Caudate (basal ganglia)   Brain   GTEX-13O3O-0011-R5b-SM-5KM44
Brain - Caudate (basal ganglia)   Brain   GTEX-13O3Q-0011-R5b-SM-5P9H4
Brain - Caudate (basal ganglia)   Brain   GTEX-13OVH-0011-R5b-

SM-5KM4L
Brain - Caudate (basal ganglia)   Brain   GTEX-13OVJ-0011-R5a-SM-5P9H7
Brain - Caudate (basal ganglia)   Brain   GTEX-13OVL-0011-R5b-SM-5L3FU
Brain - Caudate (basal ganglia)   Brain   GTEX-13OW5-0011-R5b-SM-5L3FR
Brain - Caudate (basal ganglia)   Brain   GTEX-13OW6-0011-R5a-SM-5O9BK
Brain - Caudate (basal ganglia)   Brain   GTEX-13OW7-0011-R5b-SM-5O9DM
Brain - Caudate (basal ganglia)   Brain   GTEX-13OW8-0011-R5b-SM-5L3FQ
Brain - Caudate (basal ganglia)   Brain   GTEX-13PL6-0011-R5b-SM-5O9BI
Brain - Caudate (basal ganglia)   Brain   GTEX-13PLJ-0011-R5a-SM-5O9BG
Brain - Caudate (basal ganglia)   Brain   GTEX-13PVQ-0011-R5b-SM-5O9DI
Brain - Caudate (basal ganglia)   Brain   GTEX-13RTJ-0011-R5a-SM-5P9HR
Brain - Caudate (basal ganglia)   Brain   GTEX-13S7M-0011-R5b-SM-5P9HS
Brain - Caudate (basal ganglia)   Brain   GTEX-13SLW-0011-R5b-SM-5SI6Z
Brain - Caudate (basal ganglia)   Brain   GTEX-13SLX-0011-R5a-SM-5O9BV
Brain - Caudate (basal ganglia)   Brain   GTEX-13VXU-0011-R5a-SM-5O9D7
Brain - Caudate (basal ganglia)   Brain   GTEX-13X6I-0011-R5a-SM-5PNWW
Brain - Caudate (basal ganglia)   Brain   GTEX-13X6J-0011-R5b-SM-5PNWT
Brain - Caudate (basal ganglia)   Brain   GTEX-13X6K-0011-R5b-SM-5PNUE
Brain - Caudate (basal ganglia)   Brain   GTEX-1445S-0011-R5a-SM-5PNUS
Brain - Caudate (basal ganglia)   Brain   GTEX-144GL-0011-R5b-SM-5PNUJ
Brain - Caudate (basal ganglia)   Brain   GTEX-144GO-0011-R5a-SM-5PNUR
Brain - Caudate (basal ganglia)   Brain   GTEX-145LS-0011-R5a-SM-5SI65
Brain - Caudate (basal ganglia)   Brain   GTEX-145MG-0011-R5b-SM-5S2VU
Brain - Caudate (basal ganglia)   Brain   GTEX-145MH-0011-R5a-SM-5P9JT
Brain - Caudate (basal ganglia)   Brain   GTEX-14753-0011-R5a-SM-5S2VW
Brain - Caudate (basal ganglia)   Brain   GTEX-1477Z-0011-R5b-SM-5PNUO
Brain - Caudate (basal ganglia)   Brain   GTEX-14ABY-0011-R5b-SM-5SI7B
Brain - Caudate (basal ganglia)   Brain   GTEX-14ASI-0011-R5b-

SM-6EU2M
Brain - Caudate (basal ganglia)   Brain   GTEX-14BIM-0011-R5b-SM-5S2RM
Brain - Caudate (basal ganglia)   Brain   GTEX-14BIN-0011-R5b-SM-5SI78
Brain - Caudate (basal ganglia)   Brain   GTEX-14BMV-0011-R5b-SM-62LFT
Brain - Caudate (basal ganglia)   Brain   GTEX-14C39-0011-R5b-SM-5ZZV1
Brain - Caudate (basal ganglia)   Brain   GTEX-14DAQ-0011-R5b-SM-5YYAK
Brain - Caudate (basal ganglia)   Brain   GTEX-14E6D-0011-R5b-SM-69LQR
Brain - Caudate (basal ganglia)   Brain   GTEX-14JG1-0011-R5a-SM-62LED
Brain - Caudate (basal ganglia)   Brain   GTEX-14JIY-0011-R5b-SM-62LE7
Brain - Caudate (basal ganglia)   Brain   GTEX-14LLW-0011-R5b-SM-5ZZU6
Brain - Caudate (basal ganglia)   Brain   GTEX-14LZ3-0011-R5b-SM-5ZZV8
Brain - Caudate (basal ganglia)   Brain   GTEX-14PJO-0011-R5a-SM-6AJBZ
Brain - Caudate (basal ganglia)   Brain   GTEX-14PN4-0011-R5a-SM-686ZV
Brain - Caudate (basal ganglia)   Brain   GTEX-14PQA-0011-R5b-SM-6AJC2
Brain - Caudate (basal ganglia)   Brain   GTEX-15CHQ-0011-R5b-SM-6AJAN
Brain - Caudate (basal ganglia)   Brain   GTEX-15DCD-0011-R5a-SM-69LPC
Brain - Caudate (basal ganglia)   Brain   GTEX-15DDE-0011-R5b-SM-7KULJ
Brain - Caudate (basal ganglia)   Brain   GTEX-15DYW-0011-R5a-SM-6M46W
Brain - Caudate (basal ganglia)   Brain   GTEX-15EO6-0011-R5b-SM-7KUDZ
Brain - Caudate (basal ganglia)   Brain   GTEX-15ER7-0011-R5b-SM-6M46T
Brain - Caudate (basal ganglia)   Brain   GTEX-15G19-0011-R5b-SM-6M46P
Brain - Caudate (basal ganglia)   Brain   GTEX-15SHU-0011-R5a-SM-7938Q
Brain - Caudate (basal ganglia)   Brain   GTEX-16NPV-0011-R5a-SM-7EPGK
Brain - Caudate (basal ganglia)   Brain   GTEX-16YQH-0011-R5b-SM-7938U
Brain - Caudate (basal ganglia)   Brain   GTEX-16Z82-0011-R5a-SM-7EPGB
Brain - Caudate (basal ganglia)   Brain   GTEX-17EVP-0011-R5b-SM-7DHKW
Brain - Caudate (basal ganglia)   Brain   GTEX-17EVQ-0011-R5a-SM-7LT92
Brain - Caudate (basal ganglia)   Brain   GTEX-17F97-0011-R5a-

SM-7LG4Z
Brain - Caudate (basal ganglia)   Brain   GTEX-17HG3-0011-R5a-SM-7DUEW
Brain - Caudate (basal ganglia)   Brain   GTEX-17HHY-0011-R5b-SM-79OMZ
Brain - Caudate (basal ganglia)   Brain   GTEX-17HII-0011-R5a-SM-7LG51
Brain - Caudate (basal ganglia)   Brain   GTEX-17MF6-0011-R5a-SM-731BA
Brain - Caudate (basal ganglia)   Brain   GTEX-183WM-0011-R5a-SM-718A4
Brain - Caudate (basal ganglia)   Brain   GTEX-18464-0011-R5b-SM-718A6
Brain - Caudate (basal ganglia)   Brain   GTEX-18465-0011-R5a-SM-72D5J
Brain - Caudate (basal ganglia)   Brain   GTEX-1A3MX-0011-R5a-SM-79OM5
Brain - Caudate (basal ganglia)   Brain   GTEX-1A8G6-0011-R5b-SM-7IGQG
Brain - Caudate (basal ganglia)   Brain   GTEX-1AX9I-0011-R5b-SM-793DI
Brain - Caudate (basal ganglia)   Brain   GTEX-1B8L1-0011-R5a-SM-7DHN5
Brain - Caudate (basal ganglia)   Brain   GTEX-1B8SF-0011-R5a-SM-79OOO
Brain - Caudate (basal ganglia)   Brain   GTEX-N7MS-0011-R5a-SM-2HMK8
Brain - Caudate (basal ganglia)   Brain   GTEX-N7MT-0011-R5a-SM-2I3G6
Brain - Caudate (basal ganglia)   Brain   GTEX-NL3H-0011-R5a-SM-2I3GB
Brain - Caudate (basal ganglia)   Brain   GTEX-NL4W-0011-R5a-SM-2I3GD
Brain - Caudate (basal ganglia)   Brain   GTEX-NPJ7-0011-R5a-SM-33HBK
Brain - Caudate (basal ganglia)   Brain   GTEX-NPJ8-0011-R5a-SM-2HMJY
Brain - Caudate (basal ganglia)   Brain   GTEX-OXRN-0011-R5A-SM-2I5EF
Brain - Caudate (basal ganglia)   Brain   GTEX-OXRO-0011-R5A-SM-2I5EG
Brain - Caudate (basal ganglia)   Brain   GTEX-P44H-0011-R5A-SM-2XCEX
Brain - Caudate (basal ganglia)   Brain   GTEX-PVOW-0011-R5A-SM-32PL7
Brain - Caudate (basal ganglia)   Brain   GTEX-PWO3-0011-R5A-SM-2I5EZ
Brain - Caudate (basal ganglia)   Brain   GTEX-Q2AG-0011-R5A-SM-2HMJH
Brain - Caudate (basal ganglia)   Brain   GTEX-QDT8-0011-R5A-SM-32PKN
Brain - Caudate (basal ganglia)   Brain   GTEX-QMR6-0011-R5A-SM-32PKT
Brain - Caudate (basal ganglia)   Brain   GTEX-QVJO-0011-R5A-

SM-2S1QM
Brain - Caudate (basal ganglia)   Brain   GTEX-R55E-0011-R5A-
SM-2TC5N
Brain - Caudate (basal ganglia)   Brain   GTEX-RU72-0011-R5A-
SM-2TF6U
Brain - Caudate (basal ganglia)   Brain   GTEX-RVPV-0011-R5A-
SM-2TF69
Brain - Caudate (basal ganglia)   Brain   GTEX-S7PM-0011-R5A-
SM-3NM8G
Brain - Caudate (basal ganglia)   Brain   GTEX-S7SE-0011-R5A-
SM-2XCDA
Brain - Caudate (basal ganglia)   Brain   GTEX-T2IS-0011-R5A-
SM-32QP4
Brain - Caudate (basal ganglia)   Brain   GTEX-T5JC-0011-R5A-
SM-32PLK
Brain - Caudate (basal ganglia)   Brain   GTEX-T6MN-0011-R5A-
SM-32QPD
Brain - Caudate (basal ganglia)   Brain   GTEX-TSE9-0011-R5A-
SM-3DB7J
Brain - Caudate (basal ganglia)   Brain   GTEX-UTHO-0011-R5A-
SM-3GIJD
Brain - Caudate (basal ganglia)   Brain   GTEX-WHSE-0011-R5A-
SM-3P5ZO
Brain - Caudate (basal ganglia)   Brain   GTEX-WL46-0011-R5A-
SM-3LK6V
Brain - Caudate (basal ganglia)   Brain   GTEX-WVLH-0011-R5A-
SM-3MJFW
Brain - Caudate (basal ganglia)   Brain   GTEX-WWYW-0011-R5A-
SM-3NB3E
Brain - Caudate (basal ganglia)   Brain   GTEX-WZTO-0011-R5B-
SM-3NMC5
Brain - Caudate (basal ganglia)   Brain   GTEX-X261-0011-R5A-
SM-3NMB4
Brain - Caudate (basal ganglia)   Brain   GTEX-X4XX-0011-R5A-
SM-46MWN
Brain - Caudate (basal ganglia)   Brain   GTEX-X585-0011-R5A-
SM-46MVI
Brain - Caudate (basal ganglia)   Brain   GTEX-XMD1-0011-R5A-
SM-4AT47
Brain - Caudate (basal ganglia)   Brain   GTEX-Y8DK-0011-R5B-
SM-4RTW6
Brain - Caudate (basal ganglia)   Brain   GTEX-YFC4-0011-R5a-
SM-4RGLR
Brain - Caudate (basal ganglia)   Brain   GTEX-YJ89-0011-R5a-
SM-4V6GM
Brain - Caudate (basal ganglia)   Brain   GTEX-Z93S-0011-R5b-
SM-4RGNI
Brain - Caudate (basal ganglia)   Brain   GTEX-ZAB4-0011-R5a-
SM-4SOKC
Brain - Caudate (basal ganglia)   Brain   GTEX-ZDXO-0011-R5a-
SM-4WKG5
Brain - Caudate (basal ganglia)   Brain   GTEX-ZE7O-0011-R5a-
SM-57WBO
Brain - Caudate (basal ganglia)   Brain   GTEX-ZE9C-0011-R5a-

SM-5EGLO
Brain - Caudate (basal ganglia)   Brain   GTEX-ZF28-0011-R5a-
SM-4WWCZ
Brain - Caudate (basal ganglia)   Brain   GTEX-ZUA1-0011-R5b-
SM-51MTG
Brain - Caudate (basal ganglia)   Brain   GTEX-ZV68-0011-R5a-
SM-4YCDW
Brain - Caudate (basal ganglia)   Brain   GTEX-ZVT3-0011-R5a-
SM-51MSI
Brain - Caudate (basal ganglia)   Brain   GTEX-ZVZQ-0011-R5b-
SM-57WDC
Brain - Caudate (basal ganglia)   Brain   GTEX-ZXG5-0011-R5b-
SM-57WBN
Brain - Cerebellar Hemisphere   Brain   GTEX-11GSP-0011-R11b-
SM-5NQ7V
Brain - Cerebellar Hemisphere   Brain   GTEX-11H98-0011-R11b-
SM-5NQ6U
Brain - Cerebellar Hemisphere   Brain   GTEX-11OF3-0011-R11a-
SM-57WCR
Brain - Cerebellar Hemisphere   Brain   GTEX-11WQC-0011-R11b-
SM-57WD6
Brain - Cerebellar Hemisphere   Brain   GTEX-11ZUS-0011-R11b-
SM-5BC7B
Brain - Cerebellar Hemisphere   Brain   GTEX-12126-0011-R11a-
SM-5BC6X
Brain - Cerebellar Hemisphere   Brain   GTEX-12WSC-0011-R11a-
SM-5GU5J
Brain - Cerebellar Hemisphere   Brain   GTEX-12WSE-0011-R11b-
SM-5P9JW
Brain - Cerebellar Hemisphere   Brain   GTEX-12WSF-0011-R11a-
SM-5LZVT
Brain - Cerebellar Hemisphere   Brain   GTEX-12WSH-0011-R11a-
SM-5LZVM
Brain - Cerebellar Hemisphere   Brain   GTEX-12WSI-0011-R11b-
SM-5P9EZ
Brain - Cerebellar Hemisphere   Brain   GTEX-12WSM-0011-R11a-
SM-5DUX8
Brain - Cerebellar Hemisphere   Brain   GTEX-12ZZX-0011-R11a-
SM-5DUVJ
Brain - Cerebellar Hemisphere   Brain   GTEX-12ZZZ-0011-R11a-
SM-5EGLC
Brain - Cerebellar Hemisphere   Brain   GTEX-1313W-0011-R11a-
SM-5DUXN
Brain - Cerebellar Hemisphere   Brain   GTEX-131XH-0011-R11a-
SM-5EGLN
Brain - Cerebellar Hemisphere   Brain   GTEX-131XW-0011-R11a-
SM-5DUV9
Brain - Cerebellar Hemisphere   Brain   GTEX-131YS-0011-R11b-
SM-5EGLM
Brain - Cerebellar Hemisphere   Brain   GTEX-132Q8-0011-R11b-
SM-5DUW9
Brain - Cerebellar Hemisphere   Brain   GTEX-1399T-0011-R11a-
SM-5J2O1
Brain - Cerebellar Hemisphere   Brain   GTEX-139TT-0011-R11b-

SM-5LZV1
Brain - Cerebellar Hemisphere Brain GTEX-139UW-0011-R11a-
SM-5IJGN
Brain - Cerebellar Hemisphere Brain GTEX-13CF2-0011-R11b-
SM-5LZXO
Brain - Cerebellar Hemisphere Brain GTEX-13CZV-0011-R11b-
SM-5N9FN
Brain - Cerebellar Hemisphere Brain GTEX-13FHO-0011-R11b-
SM-5LZYQ
Brain - Cerebellar Hemisphere Brain GTEX-13FHP-0011-R11b-
SM-5LZXP
Brain - Cerebellar Hemisphere Brain GTEX-13FLV-0011-R11a-
SM-5LZZ7
Brain - Cerebellar Hemisphere Brain GTEX-13FTY-0011-R11a-
SM-5IJEA
Brain - Cerebellar Hemisphere Brain GTEX-13FXS-0011-R11a-
SM-5L3DM
Brain - Cerebellar Hemisphere Brain GTEX-13G51-0011-R11b-
SM-5J2NE
Brain - Cerebellar Hemisphere Brain GTEX-13JUV-0011-R11a-
SM-5LZYH
Brain - Cerebellar Hemisphere Brain GTEX-13JVG-0011-R11a-
SM-5KM53
Brain - Cerebellar Hemisphere Brain GTEX-13N2G-0011-R11a-
SM-5MR3F
Brain - Cerebellar Hemisphere Brain GTEX-13NYB-0011-R11a-
SM-5KM3Q
Brain - Cerebellar Hemisphere Brain GTEX-13NYS-0011-R11b-
SM-5MR4P
Brain - Cerebellar Hemisphere Brain GTEX-13NZA-0011-R11b-
SM-5KM4W
Brain - Cerebellar Hemisphere Brain GTEX-13O3O-0011-R11b-
SM-5KM4G
Brain - Cerebellar Hemisphere Brain GTEX-13O3Q-0011-R11b-
SM-5KM2W
Brain - Cerebellar Hemisphere Brain GTEX-13OVH-0011-R11b-
SM-5KM4X
Brain - Cerebellar Hemisphere Brain GTEX-13OVJ-0011-R11a-
SM-5P9H8
Brain - Cerebellar Hemisphere Brain GTEX-13OVL-0011-R11b-
SM-5L3G7
Brain - Cerebellar Hemisphere Brain GTEX-13OW5-0011-R11b-
SM-5L3GG
Brain - Cerebellar Hemisphere Brain GTEX-13OW6-0011-R11a-
SM-5L3H2
Brain - Cerebellar Hemisphere Brain GTEX-13OW7-0011-R11a-
SM-5O9DY
Brain - Cerebellar Hemisphere Brain GTEX-13OW8-0011-R11a-
SM-5L3GT
Brain - Cerebellar Hemisphere Brain GTEX-13PLJ-0011-R11b-
SM-5O9BS
Brain - Cerebellar Hemisphere Brain GTEX-13PVQ-0011-R11a-
SM-5O9DU
Brain - Cerebellar Hemisphere Brain GTEX-13QIC-0011-R11b-

SM-5O9BU
Brain - Cerebellar Hemisphere   Brain   GTEX-13RTJ-0011-R11a-
SM-5O9CK
Brain - Cerebellar Hemisphere   Brain   GTEX-13S7M-0011-R11b-
SM-5P9HU
Brain - Cerebellar Hemisphere   Brain   GTEX-13SLW-0011-R11a-
SM-5SI8Q
Brain - Cerebellar Hemisphere   Brain   GTEX-13SLX-0011-R11b-
SM-5O9C8
Brain - Cerebellar Hemisphere   Brain   GTEX-13VXU-0011-R11b-
SM-5O9DJ
Brain - Cerebellar Hemisphere   Brain   GTEX-13X6I-0011-R11a-
SM-5P9HN
Brain - Cerebellar Hemisphere   Brain   GTEX-13X6J-0011-R11a-
SM-5P9HE
Brain - Cerebellar Hemisphere   Brain   GTEX-13X6K-0011-R11a-
SM-5P9F5
Brain - Cerebellar Hemisphere   Brain   GTEX-144GL-0011-R11b-
SM-5P9F6
Brain - Cerebellar Hemisphere   Brain   GTEX-145LU-0011-R11b-
SM-5P9JX
Brain - Cerebellar Hemisphere   Brain   GTEX-145MH-0011-R11a-
SM-5P9HQ
Brain - Cerebellar Hemisphere   Brain   GTEX-145MI-0011-R11a-
SM-5P9JZ
Brain - Cerebellar Hemisphere   Brain   GTEX-14ABY-0011-R11a-
SM-5SI7C
Brain - Cerebellar Hemisphere   Brain   GTEX-14ASI-0011-R11b-
SM-6AJBJ
Brain - Cerebellar Hemisphere   Brain   GTEX-14BIM-0011-R11b-
SM-5S2RN
Brain - Cerebellar Hemisphere   Brain   GTEX-14BIN-0011-R11a-
SM-5SI79
Brain - Cerebellar Hemisphere   Brain   GTEX-14C39-0011-R11b-
SM-5YYAF
Brain - Cerebellar Hemisphere   Brain   GTEX-14C5O-0011-R11b-
SM-6872H
Brain - Cerebellar Hemisphere   Brain   GTEX-14DAQ-0011-R11a-
SM-664NO
Brain - Cerebellar Hemisphere   Brain   GTEX-14E6D-0011-R11b-
SM-664NP
Brain - Cerebellar Hemisphere   Brain   GTEX-14E7W-0011-R11a-
SM-5ZZVA
Brain - Cerebellar Hemisphere   Brain   GTEX-14JG1-0011-R11a-
SM-62LEE
Brain - Cerebellar Hemisphere   Brain   GTEX-14JIY-0011-R11b-
SM-62LE8
Brain - Cerebellar Hemisphere   Brain   GTEX-14PN4-0011-R11a-
SM-686ZW
Brain - Cerebellar Hemisphere   Brain   GTEX-15CHQ-0011-R11b-
SM-686ZZ
Brain - Cerebellar Hemisphere   Brain   GTEX-15DCD-0011-R11a-
SM-6AJAF
Brain - Cerebellar Hemisphere   Brain   GTEX-15DYW-0011-R11a-

SM-6LPIT
Brain - Cerebellar Hemisphere Brain GTEX-15EO6-0011-R11b-SM-6M47M
Brain - Cerebellar Hemisphere Brain GTEX-15ER7-0011-R11a-SM-6LPIL
Brain - Cerebellar Hemisphere Brain GTEX-15G19-0011-R11b-SM-6LPIH
Brain - Cerebellar Hemisphere Brain GTEX-16GPK-0011-R11b-SM-7MKFF
Brain - Cerebellar Hemisphere Brain GTEX-16XZZ-0011-R11a-SM-7EPI3
Brain - Cerebellar Hemisphere Brain GTEX-16YQH-0011-R11b-SM-7LT98
Brain - Cerebellar Hemisphere Brain GTEX-16Z82-0011-R11a-SM-79OKJ
Brain - Cerebellar Hemisphere Brain GTEX-17EVP-0011-R11a-SM-7EWDL
Brain - Cerebellar Hemisphere Brain GTEX-17EVQ-0011-R11a-SM-7LT9C
Brain - Cerebellar Hemisphere Brain GTEX-17F97-0011-R11b-SM-7KFRQ
Brain - Cerebellar Hemisphere Brain GTEX-17HG3-0011-R11b-SM-7KFT4
Brain - Cerebellar Hemisphere Brain GTEX-17HII-0011-R11b-SM-7KFSX
Brain - Cerebellar Hemisphere Brain GTEX-17JCI-0011-R11a-SM-7189Y
Brain - Cerebellar Hemisphere Brain GTEX-183WM-0011-R11a-SM-718A5
Brain - Cerebellar Hemisphere Brain GTEX-18464-0011-R11a-SM-72D76
Brain - Cerebellar Hemisphere Brain GTEX-18465-0011-R11a-SM-72D78
Brain - Cerebellar Hemisphere Brain GTEX-18A6Q-0011-R11a-SM-72D6H
Brain - Cerebellar Hemisphere Brain GTEX-1A3MX-0011-R11b-SM-7DUGP
Brain - Cerebellar Hemisphere Brain GTEX-1AX9I-0011-R11b-SM-7DHN4
Brain - Cerebellar Hemisphere Brain GTEX-1B8L1-0011-R11b-SM-793DJ
Brain - Cerebellar Hemisphere Brain GTEX-1B8SF-0011-R11a-SM-79OOP
Brain - Cerebellar Hemisphere Brain GTEX-1BAJH-0011-R11b-SM-7IGLM
Brain - Cerebellar Hemisphere Brain GTEX-N7MS-0011-R11A-SM-2HMJS
Brain - Cerebellar Hemisphere Brain GTEX-NL4W-0011-R11A-SM-2I3DW
Brain - Cerebellar Hemisphere Brain GTEX-NPJ7-0011-R11A-SM-2I3E8
Brain - Cerebellar Hemisphere Brain GTEX-OHPN-0011-R11A-SM-2I5FM
Brain - Cerebellar Hemisphere Brain GTEX-P44H-0011-R11A-

SM−2XCER
Brain − Cerebellar Hemisphere   Brain   GTEX−Q2AG−0011−R11A−
SM−2HMKZ
Brain − Cerebellar Hemisphere   Brain   GTEX−QDT8−0011−R11A−
SM−32PKD
Brain − Cerebellar Hemisphere   Brain   GTEX−QMR6−0011−R11A−
SM−32PKK
Brain − Cerebellar Hemisphere   Brain   GTEX−R55E−0011−R11A−
SM−2TC6I
Brain − Cerebellar Hemisphere   Brain   GTEX−RU72−0011−R11A−
SM−5SI8G
Brain − Cerebellar Hemisphere   Brain   GTEX−RVPV−0011−R11A−
SM−2TF6F
Brain − Cerebellar Hemisphere   Brain   GTEX−S7SE−0011−R11A−
SM−2XCDD
Brain − Cerebellar Hemisphere   Brain   GTEX−T2IS−0011−R11A−
SM−32QPC
Brain − Cerebellar Hemisphere   Brain   GTEX−T5JC−0011−R11A−
SM−32PMB
Brain − Cerebellar Hemisphere   Brain   GTEX−T6MN−0011−R11A−
SM−32QOX
Brain − Cerebellar Hemisphere   Brain   GTEX−TSE9−0011−R11A−
SM−3DB7N
Brain − Cerebellar Hemisphere   Brain   GTEX−UTHO−0011−R11A−
SM−3GIJE
Brain − Cerebellar Hemisphere   Brain   GTEX−WHSE−0011−R11A−
SM−3P5YY
Brain − Cerebellar Hemisphere   Brain   GTEX−WL46−0011−R11A−
SM−3MJFT
Brain − Cerebellar Hemisphere   Brain   GTEX−WVLH−0011−R11A−
SM−3MJFO
Brain − Cerebellar Hemisphere   Brain   GTEX−WWYW−0011−R11A−
SM−3NB38
Brain − Cerebellar Hemisphere   Brain   GTEX−WZTO−0011−R11A−
SM−4E3K9
Brain − Cerebellar Hemisphere   Brain   GTEX−X261−0011−R11A−
SM−4E3JY
Brain − Cerebellar Hemisphere   Brain   GTEX−X4EP−0011−R11B−
SM−4QASK
Brain − Cerebellar Hemisphere   Brain   GTEX−X4XX−0011−R11A−
SM−46MWQ
Brain − Cerebellar Hemisphere   Brain   GTEX−X585−0011−R11B−
SM−46MUZ
Brain − Cerebellar Hemisphere   Brain   GTEX−XMD1−0011−R11A−
SM−4AT5J
Brain − Cerebellar Hemisphere   Brain   GTEX−Y8DK−0011−R11A−
SM−4SOK2
Brain − Cerebellar Hemisphere   Brain   GTEX−YFC4−0011−R11a−
SM−4SOK6
Brain − Cerebellar Hemisphere   Brain   GTEX−YJ89−0011−R11a−
SM−4SOKA
Brain − Cerebellar Hemisphere   Brain   GTEX−Z93S−0011−R11a−
SM−4RGNN
Brain − Cerebellar Hemisphere   Brain   GTEX−ZAB4−0011−R11a−

SM-4SOKI
Brain - Cerebellar Hemisphere	Brain	GTEX-ZDXO-0011-R11a-SM-4WWD9
Brain - Cerebellar Hemisphere	Brain	GTEX-ZE7O-0011-R11a-SM-57WBC
Brain - Cerebellar Hemisphere	Brain	GTEX-ZE9C-0011-R11a-SM-4WKGG
Brain - Cerebellar Hemisphere	Brain	GTEX-ZF28-0011-R11a-SM-4WWEI
Brain - Cerebellar Hemisphere	Brain	GTEX-ZUA1-0011-R11b-SM-51MTI
Brain - Cerebellar Hemisphere	Brain	GTEX-ZVT3-0011-R11b-SM-57WBI
Brain - Cerebellar Hemisphere	Brain	GTEX-ZVZQ-0011-R11a-SM-51MS6
Brain - Cerebellum	Brain	GTEX-111FC-3326-SM-5GZYV
Brain - Cerebellum	Brain	GTEX-1128S-2826-SM-5N9DI
Brain - Cerebellum	Brain	GTEX-117XS-3126-SM-5GIDP
Brain - Cerebellum	Brain	GTEX-1192X-3226-SM-5987D
Brain - Cerebellum	Brain	GTEX-11DXW-1026-SM-5H11K
Brain - Cerebellum	Brain	GTEX-11DXY-3126-SM-5N9BT
Brain - Cerebellum	Brain	GTEX-11DYG-2926-SM-5H132
Brain - Cerebellum	Brain	GTEX-11DZ1-2926-SM-5A5KI
Brain - Cerebellum	Brain	GTEX-11EI6-2926-SM-5985U
Brain - Cerebellum	Brain	GTEX-11EMC-3326-SM-5P9JH
Brain - Cerebellum	Brain	GTEX-11GSO-3026-SM-5Q5AL
Brain - Cerebellum	Brain	GTEX-11GSP-3126-SM-5A5LL
Brain - Cerebellum	Brain	GTEX-11NUK-3026-SM-5986K
Brain - Cerebellum	Brain	GTEX-11NV4-2226-SM-5BC4T
Brain - Cerebellum	Brain	GTEX-11O72-2826-SM-5HL6W
Brain - Cerebellum	Brain	GTEX-11OC5-0626-SM-5HL6M
Brain - Cerebellum	Brain	GTEX-11ONC-3026-SM-5985W
Brain - Cerebellum	Brain	GTEX-11PRG-2826-SM-5BC54
Brain - Cerebellum	Brain	GTEX-11TTK-2826-SM-5GU5K
Brain - Cerebellum	Brain	GTEX-11ZTS-3226-SM-5EGID
Brain - Cerebellum	Brain	GTEX-11ZUS-2826-SM-5EQKW
Brain - Cerebellum	Brain	GTEX-11ZVC-3026-SM-59HJM
Brain - Cerebellum	Brain	GTEX-12126-0926-SM-5FQTW
Brain - Cerebellum	Brain	GTEX-12696-3026-SM-5FQTU
Brain - Cerebellum	Brain	GTEX-1269C-3126-SM-5EGI6
Brain - Cerebellum	Brain	GTEX-12WSC-2926-SM-5BC5Z
Brain - Cerebellum	Brain	GTEX-12WSD-3026-SM-5LZU7
Brain - Cerebellum	Brain	GTEX-12WSE-2926-SM-5RQJ9
Brain - Cerebellum	Brain	GTEX-12WSH-3126-SM-5LZW8
Brain - Cerebellum	Brain	GTEX-12WSM-2126-SM-5GCOC
Brain - Cerebellum	Brain	GTEX-12ZZX-2826-SM-5BC6K
Brain - Cerebellum	Brain	GTEX-12ZZY-2926-SM-5DUXO
Brain - Cerebellum	Brain	GTEX-12ZZZ-3126-SM-5LZU6
Brain - Cerebellum	Brain	GTEX-13112-2926-SM-5DUWT
Brain - Cerebellum	Brain	GTEX-1313W-3026-SM-5LZUZ
Brain - Cerebellum	Brain	GTEX-131XH-2926-SM-5LZU4
Brain - Cerebellum	Brain	GTEX-131YS-3026-SM-5EGHY
Brain - Cerebellum	Brain	GTEX-132Q8-2926-SM-5IFFP
Brain - Cerebellum	Brain	GTEX-1399T-3126-SM-5IJFS

Brain - Cerebellum	Brain	GTEX-139TS-3126-SM-5LZWX
Brain - Cerebellum	Brain	GTEX-139TT-2626-SM-5LZUB
Brain - Cerebellum	Brain	GTEX-13CF2-3026-SM-5IFFX
Brain - Cerebellum	Brain	GTEX-13FHO-2926-SM-5L3ES
Brain - Cerebellum	Brain	GTEX-13FHP-2926-SM-5IJG9
Brain - Cerebellum	Brain	GTEX-13FXS-3026-SM-5LZYO
Brain - Cerebellum	Brain	GTEX-13G51-3126-SM-5IJG7
Brain - Cerebellum	Brain	GTEX-13JUV-3026-SM-5K7WX
Brain - Cerebellum	Brain	GTEX-13JVG-3226-SM-5IJGJ
Brain - Cerebellum	Brain	GTEX-13N2G-3126-SM-5IJGL
Brain - Cerebellum	Brain	GTEX-13NYB-3226-SM-5J2ND
Brain - Cerebellum	Brain	GTEX-13O3O-3026-SM-5KM42
Brain - Cerebellum	Brain	GTEX-13O3Q-3026-SM-5IJGD
Brain - Cerebellum	Brain	GTEX-13OVH-2926-SM-5IJFH
Brain - Cerebellum	Brain	GTEX-13OVJ-2726-SM-5IJG6
Brain - Cerebellum	Brain	GTEX-13OVL-3026-SM-5IJF1
Brain - Cerebellum	Brain	GTEX-13OW5-3126-SM-5MR3Y
Brain - Cerebellum	Brain	GTEX-13OW6-2926-SM-5KM29
Brain - Cerebellum	Brain	GTEX-13OW7-2926-SM-5MR3Z
Brain - Cerebellum	Brain	GTEX-13OW8-2926-SM-5L3FV
Brain - Cerebellum	Brain	GTEX-13PVQ-2926-SM-5L3HJ
Brain - Cerebellum	Brain	GTEX-13QIC-3026-SM-5LU58
Brain - Cerebellum	Brain	GTEX-13RTJ-3126-SM-5S2Q4
Brain - Cerebellum	Brain	GTEX-13S7M-3026-SM-5S2QQ
Brain - Cerebellum	Brain	GTEX-13SLX-3226-SM-5YYA6
Brain - Cerebellum	Brain	GTEX-13VXU-3026-SM-5LU3K
Brain - Cerebellum	Brain	GTEX-13X6I-2926-SM-5Q5C3
Brain - Cerebellum	Brain	GTEX-13X6J-2926-SM-7EPGP
Brain - Cerebellum	Brain	GTEX-13X6K-2826-SM-5O9DS
Brain - Cerebellum	Brain	GTEX-144GL-2926-SM-5O99F
Brain - Cerebellum	Brain	GTEX-145LS-3026-SM-5O99S
Brain - Cerebellum	Brain	GTEX-145LU-2626-SM-5LU9Z
Brain - Cerebellum	Brain	GTEX-145MF-2826-SM-7EPGU
Brain - Cerebellum	Brain	GTEX-145MG-3126-SM-5Q5D3
Brain - Cerebellum	Brain	GTEX-145MH-2926-SM-5Q5D2
Brain - Cerebellum	Brain	GTEX-145MI-3126-SM-5Q5CF
Brain - Cerebellum	Brain	GTEX-147F4-3026-SM-5Q5BJ
Brain - Cerebellum	Brain	GTEX-147GR-3126-SM-5S2PM
Brain - Cerebellum	Brain	GTEX-14A5I-2826-SM-5SIBF
Brain - Cerebellum	Brain	GTEX-14ABY-3026-SM-664NE
Brain - Cerebellum	Brain	GTEX-14ASI-2926-SM-5Q5DG
Brain - Cerebellum	Brain	GTEX-14BIM-3126-SM-5Q5C7
Brain - Cerebellum	Brain	GTEX-14BIN-3326-SM-5Q5E6
Brain - Cerebellum	Brain	GTEX-14BMV-2926-SM-5TDEE
Brain - Cerebellum	Brain	GTEX-14C39-3026-SM-5S2QE
Brain - Cerebellum	Brain	GTEX-14C5O-2926-SM-5RQI1
Brain - Cerebellum	Brain	GTEX-14DAQ-3026-SM-5S2QZ
Brain - Cerebellum	Brain	GTEX-14E6D-2926-SM-664OP
Brain - Cerebellum	Brain	GTEX-14E7W-2926-SM-5S2R8
Brain - Cerebellum	Brain	GTEX-14JFF-2826-SM-6871Q
Brain - Cerebellum	Brain	GTEX-14JG1-2926-SM-686ZK
Brain - Cerebellum	Brain	GTEX-14JIY-2326-SM-6AJAJ
Brain - Cerebellum	Brain	GTEX-14PJM-3026-SM-6LLHM
Brain - Cerebellum	Brain	GTEX-14PJO-2926-SM-686ZI

Brain - Cerebellum	Brain	GTEX-14PN4-3026-SM-664O4
Brain - Cerebellum	Brain	GTEX-15DYW-3026-SM-7KFRM
Brain - Cerebellum	Brain	GTEX-15EO6-2926-SM-6M48D
Brain - Cerebellum	Brain	GTEX-15ER7-3026-SM-6M47W
Brain - Cerebellum	Brain	GTEX-15G19-2826-SM-6M48K
Brain - Cerebellum	Brain	GTEX-15SHU-3026-SM-6LPIB
Brain - Cerebellum	Brain	GTEX-16XZZ-2926-SM-6LPK4
Brain - Cerebellum	Brain	GTEX-16YQH-2926-SM-6LPJE
Brain - Cerebellum	Brain	GTEX-16Z82-2926-SM-6LPJJ
Brain - Cerebellum	Brain	GTEX-17EVP-2926-SM-793BU
Brain - Cerebellum	Brain	GTEX-17EVQ-2726-SM-7EWDT
Brain - Cerebellum	Brain	GTEX-17F97-2926-SM-7DUEQ
Brain - Cerebellum	Brain	GTEX-17HG3-2826-SM-7EPGO
Brain - Cerebellum	Brain	GTEX-17HHY-2926-SM-79OJW
Brain - Cerebellum	Brain	GTEX-17HII-2926-SM-79OMK
Brain - Cerebellum	Brain	GTEX-17JCI-3026-SM-7EWDR
Brain - Cerebellum	Brain	GTEX-183WM-2726-SM-7KFRF
Brain - Cerebellum	Brain	GTEX-18465-2926-SM-7KFSE
Brain - Cerebellum	Brain	GTEX-18A6Q-2826-SM-7LTAO
Brain - Cerebellum	Brain	GTEX-1A3MX-2926-SM-718B7
Brain - Cerebellum	Brain	GTEX-1A8G6-3126-SM-731D8
Brain - Cerebellum	Brain	GTEX-1A8G7-3026-SM-73KTT
Brain - Cerebellum	Brain	GTEX-1B8SF-3026-SM-731DX
Brain - Cerebellum	Brain	GTEX-1B8SG-1926-SM-73KUB
Brain - Cerebellum	Brain	GTEX-1B933-3026-SM-731DB
Brain - Cerebellum	Brain	GTEX-1C64N-3126-SM-79OO3
Brain - Cerebellum	Brain	GTEX-1C6VR-3026-SM-7IGLW
Brain - Cerebellum	Brain	GTEX-1C6WA-3326-SM-7MKGN
Brain - Cerebellum	Brain	GTEX-1CB4G-2926-SM-7MKFQ
Brain - Cerebellum	Brain	GTEX-1CB4H-3026-SM-79OLX
Brain - Cerebellum	Brain	GTEX-1E1VI-2926-SM-7IGLT
Brain - Cerebellum	Brain	GTEX-1EU9M-2926-SM-793DF
Brain - Cerebellum	Brain	GTEX-1EWIQ-3026-SM-7IGOJ
Brain - Cerebellum	Brain	GTEX-N7MS-2526-SM-2D7W3
Brain - Cerebellum	Brain	GTEX-N7MT-1226-SM-2D7W4
Brain - Cerebellum	Brain	GTEX-NPJ7-2826-SM-2I3FU
Brain - Cerebellum	Brain	GTEX-NPJ8-2626-SM-2D7W2
Brain - Cerebellum	Brain	GTEX-OHPN-2626-SM-2I5H1
Brain - Cerebellum	Brain	GTEX-P44H-2426-SM-2XCEJ
Brain - Cerebellum	Brain	GTEX-PVOW-2626-SM-32PL8
Brain - Cerebellum	Brain	GTEX-PWO3-1026-SM-2I5F2
Brain - Cerebellum	Brain	GTEX-Q2AG-2826-SM-2HMJQ
Brain - Cerebellum	Brain	GTEX-QDT8-3026-SM-32PKB
Brain - Cerebellum	Brain	GTEX-QMR6-1326-SM-32PLB
Brain - Cerebellum	Brain	GTEX-QVJO-1325-SM-2S1QX
Brain - Cerebellum	Brain	GTEX-QVUS-2926-SM-3GIJB
Brain - Cerebellum	Brain	GTEX-R55E-2526-SM-2TC6H
Brain - Cerebellum	Brain	GTEX-RNOR-2226-SM-2TF5O
Brain - Cerebellum	Brain	GTEX-RU72-2926-SM-2TF66
Brain - Cerebellum	Brain	GTEX-S7SE-2526-SM-2XCDL
Brain - Cerebellum	Brain	GTEX-T2IS-2926-SM-32QPO
Brain - Cerebellum	Brain	GTEX-T5JC-2326-SM-32PMR
Brain - Cerebellum	Brain	GTEX-T6MN-2526-SM-32PMN
Brain - Cerebellum	Brain	GTEX-TSE9-2926-SM-3DB77

Brain - Cerebellum	Brain	GTEX-UTHO-2926-SM-3P5Z9
Brain - Cerebellum	Brain	GTEX-WHSE-2926-SM-3NMBG
Brain - Cerebellum	Brain	GTEX-WL46-2826-SM-3LK81
Brain - Cerebellum	Brain	GTEX-WVLH-2926-SM-3MJG5
Brain - Cerebellum	Brain	GTEX-WWYW-3026-SM-3NB36
Brain - Cerebellum	Brain	GTEX-WZTO-2826-SM-3NM8P
Brain - Cerebellum	Brain	GTEX-X261-3126-SM-4PQZC
Brain - Cerebellum	Brain	GTEX-X4EP-3026-SM-3P5YP
Brain - Cerebellum	Brain	GTEX-X4XX-2926-SM-3NMB1
Brain - Cerebellum	Brain	GTEX-XLM4-2926-SM-4AT59
Brain - Cerebellum	Brain	GTEX-XMD1-2826-SM-4AT5F
Brain - Cerebellum	Brain	GTEX-Y111-2826-SM-4TT3O
Brain - Cerebellum	Brain	GTEX-Y8DK-0726-SM-4TT3S
Brain - Cerebellum	Brain	GTEX-YFC4-3026-SM-5IFJK
Brain - Cerebellum	Brain	GTEX-YJ89-2926-SM-5P9IT
Brain - Cerebellum	Brain	GTEX-Z93S-2826-SM-57WBL
Brain - Cerebellum	Brain	GTEX-ZAB4-2926-SM-57WCV
Brain - Cerebellum	Brain	GTEX-ZAJG-3026-SM-5HL92
Brain - Cerebellum	Brain	GTEX-ZAK1-2926-SM-5HL9S
Brain - Cerebellum	Brain	GTEX-ZDXO-2926-SM-4WKFM
Brain - Cerebellum	Brain	GTEX-ZF28-2926-SM-4WKG1
Brain - Cerebellum	Brain	GTEX-ZUA1-2926-SM-59HL3
Brain - Cerebellum	Brain	GTEX-ZVT3-2926-SM-5GU6M
Brain - Cerebellum	Brain	GTEX-ZYFD-2926-SM-5GID9
Brain - Cerebellum	Brain	GTEX-ZYY3-3026-SM-5GIEJ
Brain - Cerebellum	Brain	GTEX-ZZPT-2926-SM-5EQ5S
Brain - Cortex	Brain	GTEX-1117F-3226-SM-5N9CT
Brain - Cortex	Brain	GTEX-111FC-3126-SM-5GZZ2
Brain - Cortex	Brain	GTEX-1128S-2726-SM-5H12C
Brain - Cortex	Brain	GTEX-117XS-3026-SM-5N9CA
Brain - Cortex	Brain	GTEX-1192X-3126-SM-5N9BY
Brain - Cortex	Brain	GTEX-11DXW-1126-SM-5H12Q
Brain - Cortex	Brain	GTEX-11DXY-3226-SM-5GIDE
Brain - Cortex	Brain	GTEX-11EI6-3026-SM-5GZZO
Brain - Cortex	Brain	GTEX-11EMC-3226-SM-5EGKW
Brain - Cortex	Brain	GTEX-11GS4-3126-SM-5A5LH
Brain - Cortex	Brain	GTEX-11GSO-2926-SM-5HL73
Brain - Cortex	Brain	GTEX-11GSP-3226-SM-5986O
Brain - Cortex	Brain	GTEX-11NUK-2926-SM-5A5MD
Brain - Cortex	Brain	GTEX-11NV4-2126-SM-5N9DS
Brain - Cortex	Brain	GTEX-11O72-2926-SM-5BC4V
Brain - Cortex	Brain	GTEX-11OC5-0726-SM-5P9JK
Brain - Cortex	Brain	GTEX-11ONC-2926-SM-5P9JM
Brain - Cortex	Brain	GTEX-11PRG-2926-SM-5987A
Brain - Cortex	Brain	GTEX-11TTK-2926-SM-5PNYP
Brain - Cortex	Brain	GTEX-11WQK-3026-SM-5EQL6
Brain - Cortex	Brain	GTEX-11ZUS-2926-SM-5FQSL
Brain - Cortex	Brain	GTEX-12126-1026-SM-5P9JJ
Brain - Cortex	Brain	GTEX-12WSA-2926-SM-5EQ4D
Brain - Cortex	Brain	GTEX-12WSC-3026-SM-5GCNF
Brain - Cortex	Brain	GTEX-12WSD-3126-SM-5HL7P
Brain - Cortex	Brain	GTEX-12WSF-3126-SM-6M478
Brain - Cortex	Brain	GTEX-12WSH-3026-SM-5CVNI
Brain - Cortex	Brain	GTEX-12ZZW-2926-SM-5LZUP

Brain - Cortex	Brain	GTEX-12ZZX-2926-SM-5GCOQ
Brain - Cortex	Brain	GTEX-12ZZY-3026-SM-5GCOU
Brain - Cortex	Brain	GTEX-12ZZZ-3026-SM-5BC67
Brain - Cortex	Brain	GTEX-1313W-3126-SM-5LZUI
Brain - Cortex	Brain	GTEX-131XW-3126-SM-5LZUC
Brain - Cortex	Brain	GTEX-131YS-3126-SM-5KLYT
Brain - Cortex	Brain	GTEX-132Q8-3026-SM-5PNVG
Brain - Cortex	Brain	GTEX-1399T-3026-SM-5KLZC
Brain - Cortex	Brain	GTEX-139T8-1026-SM-5J2MC
Brain - Cortex	Brain	GTEX-13FHO-3026-SM-5J1O9
Brain - Cortex	Brain	GTEX-13FHP-3026-SM-5IJBS
Brain - Cortex	Brain	GTEX-13FLW-1426-SM-5K7YE
Brain - Cortex	Brain	GTEX-13FXS-3126-SM-5J2NN
Brain - Cortex	Brain	GTEX-13IVO-2926-SM-5L3CZ
Brain - Cortex	Brain	GTEX-13JUV-2926-SM-5LZX7
Brain - Cortex	Brain	GTEX-13JVG-3126-SM-5L3FH
Brain - Cortex	Brain	GTEX-13NYB-3026-SM-5IJD7
Brain - Cortex	Brain	GTEX-13NYC-2826-SM-5K7WR
Brain - Cortex	Brain	GTEX-13NYS-3126-SM-5KLYV
Brain - Cortex	Brain	GTEX-13O3O-3126-SM-5KM3H
Brain - Cortex	Brain	GTEX-13O3Q-2926-SM-5KM45
Brain - Cortex	Brain	GTEX-13OVH-3026-SM-5MR4N
Brain - Cortex	Brain	GTEX-13OVJ-2826-SM-5L3GW
Brain - Cortex	Brain	GTEX-13OW6-3026-SM-5J2MI
Brain - Cortex	Brain	GTEX-13OW7-3026-SM-5L3GY
Brain - Cortex	Brain	GTEX-13OW8-2826-SM-5L3GC
Brain - Cortex	Brain	GTEX-13PL6-3126-SM-5LUAR
Brain - Cortex	Brain	GTEX-13PVQ-3026-SM-5SI93
Brain - Cortex	Brain	GTEX-13QIC-2926-SM-5J2NF
Brain - Cortex	Brain	GTEX-13S7M-3126-SM-5RQJQ
Brain - Cortex	Brain	GTEX-13SLX-3126-SM-5S2Q5
Brain - Cortex	Brain	GTEX-13VXU-2926-SM-5LU5C
Brain - Cortex	Brain	GTEX-13X6J-3026-SM-5Q5CU
Brain - Cortex	Brain	GTEX-13X6K-2926-SM-5Q5D5
Brain - Cortex	Brain	GTEX-1445S-3026-SM-5O9BR
Brain - Cortex	Brain	GTEX-144FL-3026-SM-5O99C
Brain - Cortex	Brain	GTEX-144GL-3026-SM-5Q5CW
Brain - Cortex	Brain	GTEX-145LS-3126-SM-5Q5BY
Brain - Cortex	Brain	GTEX-145MF-2726-SM-5O995
Brain - Cortex	Brain	GTEX-145MG-3026-SM-5RQJA
Brain - Cortex	Brain	GTEX-145MH-3026-SM-5Q5DZ
Brain - Cortex	Brain	GTEX-147GR-3026-SM-5S2ML
Brain - Cortex	Brain	GTEX-14A5I-2926-SM-5Q5CQ
Brain - Cortex	Brain	GTEX-14ASI-3026-SM-5S2PN
Brain - Cortex	Brain	GTEX-14BIL-3026-SM-7EWD4
Brain - Cortex	Brain	GTEX-14BIM-3026-SM-7EWCY
Brain - Cortex	Brain	GTEX-14BIN-3226-SM-62LDR
Brain - Cortex	Brain	GTEX-14BMV-3026-SM-5S2PQ
Brain - Cortex	Brain	GTEX-14C39-3126-SM-5ZZW6
Brain - Cortex	Brain	GTEX-14C5O-3026-SM-5YYB2
Brain - Cortex	Brain	GTEX-14DAQ-3126-SM-62LDS
Brain - Cortex	Brain	GTEX-14JG1-3026-SM-5YYAA
Brain - Cortex	Brain	GTEX-14JIY-2926-SM-69LPT
Brain - Cortex	Brain	GTEX-14LZ3-3026-SM-5ZZVU

Brain - Cortex	Brain	GTEX-14PJM-3126-SM-6EU2R
Brain - Cortex	Brain	GTEX-15EO6-3026-SM-7KUMA
Brain - Cortex	Brain	GTEX-15ER7-3126-SM-7KUGH
Brain - Cortex	Brain	GTEX-15G19-2926-SM-7KUFL
Brain - Cortex	Brain	GTEX-15RJE-3026-SM-7KUM1
Brain - Cortex	Brain	GTEX-15SHU-3126-SM-6M46H
Brain - Cortex	Brain	GTEX-15UF6-3026-SM-7KUKL
Brain - Cortex	Brain	GTEX-16GPK-3026-SM-79ON9
Brain - Cortex	Brain	GTEX-16XZZ-3026-SM-79OMJ
Brain - Cortex	Brain	GTEX-16YQH-3026-SM-79OMM
Brain - Cortex	Brain	GTEX-16Z82-3026-SM-79ON3
Brain - Cortex	Brain	GTEX-17EVP-3026-SM-7IGQL
Brain - Cortex	Brain	GTEX-17EVQ-2826-SM-7IGQK
Brain - Cortex	Brain	GTEX-17F97-3026-SM-79OKC
Brain - Cortex	Brain	GTEX-17HG3-2926-SM-79ON8
Brain - Cortex	Brain	GTEX-17HHY-3026-SM-79OKP
Brain - Cortex	Brain	GTEX-17HII-3026-SM-7EWFM
Brain - Cortex	Brain	GTEX-17JCI-3126-SM-7LTA2
Brain - Cortex	Brain	GTEX-17MF6-2926-SM-7LTAW
Brain - Cortex	Brain	GTEX-183WM-2826-SM-731C2
Brain - Cortex	Brain	GTEX-18465-3026-SM-7LG6Y
Brain - Cortex	Brain	GTEX-18A6Q-2926-SM-718AR
Brain - Cortex	Brain	GTEX-1A3MX-3026-SM-731BF
Brain - Cortex	Brain	GTEX-1A8G6-2926-SM-731CK
Brain - Cortex	Brain	GTEX-1A8G7-3126-SM-7IGP7
Brain - Cortex	Brain	GTEX-1AX9I-3026-SM-73KUH
Brain - Cortex	Brain	GTEX-1B8L1-3026-SM-7EPHK
Brain - Cortex	Brain	GTEX-1B8SF-3126-SM-73KU1
Brain - Cortex	Brain	GTEX-1B8SG-1726-SM-73KXK
Brain - Cortex	Brain	GTEX-1C6VQ-3026-SM-7IGPU
Brain - Cortex	Brain	GTEX-1C6VS-3026-SM-79OLA
Brain - Cortex	Brain	GTEX-1CAV2-2726-SM-7IGNN
Brain - Cortex	Brain	GTEX-1CB4H-3126-SM-7IGN4
Brain - Cortex	Brain	GTEX-1E2YA-3026-SM-7IGO7
Brain - Cortex	Brain	GTEX-1EKGG-2926-SM-7EPIH
Brain - Cortex	Brain	GTEX-NPJ7-2726-SM-2I3FT
Brain - Cortex	Brain	GTEX-NPJ8-1526-SM-2D7VU
Brain - Cortex	Brain	GTEX-OXRN-2426-SM-2I5EQ
Brain - Cortex	Brain	GTEX-PVOW-2526-SM-2XCF7
Brain - Cortex	Brain	GTEX-PWO3-0926-SM-2I5EY
Brain - Cortex	Brain	GTEX-Q2AG-2926-SM-2HMJ3
Brain - Cortex	Brain	GTEX-QDT8-2926-SM-32PKC
Brain - Cortex	Brain	GTEX-QMR6-1426-SM-32PLA
Brain - Cortex	Brain	GTEX-QVJO-1426-SM-2S1QY
Brain - Cortex	Brain	GTEX-QVUS-2826-SM-3GADB
Brain - Cortex	Brain	GTEX-RNOR-2326-SM-2TF4I
Brain - Cortex	Brain	GTEX-RU72-3026-SM-5SI7Y
Brain - Cortex	Brain	GTEX-T2IS-3026-SM-32QPM
Brain - Cortex	Brain	GTEX-T5JC-2426-SM-3NMDB
Brain - Cortex	Brain	GTEX-T6MN-2626-SM-32PMQ
Brain - Cortex	Brain	GTEX-TSE9-3026-SM-3DB76
Brain - Cortex	Brain	GTEX-UTHO-3026-SM-3GAFB
Brain - Cortex	Brain	GTEX-WHSE-3026-SM-3P5ZH
Brain - Cortex	Brain	GTEX-WL46-2926-SM-3LK82

Brain - Cortex	Brain	GTEX-WVLH-3026-SM-3MJG9
Brain - Cortex	Brain	GTEX-WWYW-3126-SM-3NB39
Brain - Cortex	Brain	GTEX-WZTO-2926-SM-3NM9I
Brain - Cortex	Brain	GTEX-X4XX-3026-SM-3NMB2
Brain - Cortex	Brain	GTEX-X585-3026-SM-46MWF
Brain - Cortex	Brain	GTEX-XLM4-3026-SM-4AT6L
Brain - Cortex	Brain	GTEX-Y111-2726-SM-4TT3N
Brain - Cortex	Brain	GTEX-Y8DK-0826-SM-4TT3T
Brain - Cortex	Brain	GTEX-YFC4-3126-SM-5PNV6
Brain - Cortex	Brain	GTEX-YJ89-3026-SM-5IFJI
Brain - Cortex	Brain	GTEX-Z93S-2926-SM-57WB9
Brain - Cortex	Brain	GTEX-ZAJG-3126-SM-5HL9J
Brain - Cortex	Brain	GTEX-ZAK1-3026-SM-5S2MJ
Brain - Cortex	Brain	GTEX-ZDXO-2126-SM-4WKFI
Brain - Cortex	Brain	GTEX-ZE7O-3126-SM-5HL5X
Brain - Cortex	Brain	GTEX-ZE9C-3026-SM-4WKHB
Brain - Cortex	Brain	GTEX-ZF28-3026-SM-4WKHP
Brain - Cortex	Brain	GTEX-ZUA1-3026-SM-59HJC
Brain - Cortex	Brain	GTEX-ZVT3-3026-SM-5E43N
Brain - Cortex	Brain	GTEX-ZYFD-3026-SM-5E44C
Brain - Cortex	Brain	GTEX-ZYY3-3126-SM-5SI9L
Brain - Cortex	Brain	GTEX-ZZPT-3026-SM-5GZXH
Brain - Frontal Cortex (BA9)	Brain	GTEX-11GSP-0011-R10a-SM-5NQ7J
Brain - Frontal Cortex (BA9)	Brain	GTEX-11H98-0011-R10b-SM-5NQ98
Brain - Frontal Cortex (BA9)	Brain	GTEX-11WQC-0011-R10a-SM-57WCT
Brain - Frontal Cortex (BA9)	Brain	GTEX-11WQK-0011-R10a-SM-5BC6R
Brain - Frontal Cortex (BA9)	Brain	GTEX-11ZUS-0011-R10b-SM-5BC79
Brain - Frontal Cortex (BA9)	Brain	GTEX-12126-0011-R10b-SM-5BC6T
Brain - Frontal Cortex (BA9)	Brain	GTEX-12WSA-0011-R10b-SM-5P9ET
Brain - Frontal Cortex (BA9)	Brain	GTEX-12WSC-0011-R10a-SM-5GU57
Brain - Frontal Cortex (BA9)	Brain	GTEX-12WSD-0011-R10b-SM-5GU79
Brain - Frontal Cortex (BA9)	Brain	GTEX-12WSE-0011-R10b-SM-5P9JV
Brain - Frontal Cortex (BA9)	Brain	GTEX-12WSF-0011-R10a-SM-5LZVH
Brain - Frontal Cortex (BA9)	Brain	GTEX-12WSH-0011-R10a-SM-5LZUS
Brain - Frontal Cortex (BA9)	Brain	GTEX-12WSM-0011-R10a-SM-5DUWV
Brain - Frontal Cortex (BA9)	Brain	GTEX-12ZZW-0011-R10b-SM-5HL9X
Brain - Frontal Cortex (BA9)	Brain	GTEX-12ZZX-0011-R10b-SM-5DUWK
Brain - Frontal Cortex (BA9)	Brain	GTEX-12ZZY-0011-R10b-SM-5HL7W

Brain - Frontal Cortex (BA9)	Brain	GTEX-12ZZZ-0011-R10a-SM-5P9HC
Brain - Frontal Cortex (BA9)	Brain	GTEX-1313W-0011-R10b-SM-5DUXA
Brain - Frontal Cortex (BA9)	Brain	GTEX-131XH-0011-R10a-SM-5EGLJ
Brain - Frontal Cortex (BA9)	Brain	GTEX-131XW-0011-R10a-SM-5DUVA
Brain - Frontal Cortex (BA9)	Brain	GTEX-131YS-0011-R10b-SM-5EQ5N
Brain - Frontal Cortex (BA9)	Brain	GTEX-132Q8-0011-R10b-SM-5DUWZ
Brain - Frontal Cortex (BA9)	Brain	GTEX-139TS-0011-R10a-SM-5K7TU
Brain - Frontal Cortex (BA9)	Brain	GTEX-13CZV-0011-R10b-SM-5LZYD
Brain - Frontal Cortex (BA9)	Brain	GTEX-13FHO-0011-R10b-SM-5J2MM
Brain - Frontal Cortex (BA9)	Brain	GTEX-13FLV-0011-R10b-SM-5LZZ2
Brain - Frontal Cortex (BA9)	Brain	GTEX-13FLW-0011-R10b-SM-5LZZF
Brain - Frontal Cortex (BA9)	Brain	GTEX-13FTZ-0011-R10b-SM-5KLZS
Brain - Frontal Cortex (BA9)	Brain	GTEX-13FXS-0011-R10a-SM-5J2MA
Brain - Frontal Cortex (BA9)	Brain	GTEX-13G51-0011-R10b-SM-5LZYF
Brain - Frontal Cortex (BA9)	Brain	GTEX-13IVO-0011-R10a-SM-5LZY3
Brain - Frontal Cortex (BA9)	Brain	GTEX-13JUV-0011-R10b-SM-5LZXR
Brain - Frontal Cortex (BA9)	Brain	GTEX-13JVG-0011-R10b-SM-5KM2P
Brain - Frontal Cortex (BA9)	Brain	GTEX-13N1W-0011-R10b-SM-5MR4H
Brain - Frontal Cortex (BA9)	Brain	GTEX-13N2G-0011-R10a-SM-5MR34
Brain - Frontal Cortex (BA9)	Brain	GTEX-13NYB-0011-R10a-SM-5KM43
Brain - Frontal Cortex (BA9)	Brain	GTEX-13NYS-0011-R10a-SM-5MR4S
Brain - Frontal Cortex (BA9)	Brain	GTEX-13NZA-0011-R10b-SM-5KM54
Brain - Frontal Cortex (BA9)	Brain	GTEX-13O3O-0011-R10a-SM-5LUA9
Brain - Frontal Cortex (BA9)	Brain	GTEX-13O3Q-0011-R10b-SM-5KM39
Brain - Frontal Cortex (BA9)	Brain	GTEX-13OVJ-0011-R10b-SM-5L3HT
Brain - Frontal Cortex (BA9)	Brain	GTEX-13OVL-0011-R10a-SM-5L3GS
Brain - Frontal Cortex (BA9)	Brain	GTEX-13OW6-0011-R10a-SM-5L3HE

Brain - Frontal Cortex (BA9)	Brain	GTEX-13OW7-0011-R10a-SM-5L3H3
Brain - Frontal Cortex (BA9)	Brain	GTEX-13OW8-0011-R10a-SM-5L3H6
Brain - Frontal Cortex (BA9)	Brain	GTEX-13QIC-0011-R10a-SM-5O9C7
Brain - Frontal Cortex (BA9)	Brain	GTEX-13QJC-0011-R10a-SM-5SI63
Brain - Frontal Cortex (BA9)	Brain	GTEX-13RTJ-0011-R10b-SM-5O9CW
Brain - Frontal Cortex (BA9)	Brain	GTEX-13S7M-0011-R10b-SM-5PNZB
Brain - Frontal Cortex (BA9)	Brain	GTEX-13SLW-0011-R10a-SM-5S2UI
Brain - Frontal Cortex (BA9)	Brain	GTEX-13SLX-0011-R10a-SM-5P9HV
Brain - Frontal Cortex (BA9)	Brain	GTEX-13X6I-0011-R10a-SM-5PNWI
Brain - Frontal Cortex (BA9)	Brain	GTEX-13X6J-0011-R10b-SM-5PNWA
Brain - Frontal Cortex (BA9)	Brain	GTEX-13X6K-0011-R10a-SM-5P9JR
Brain - Frontal Cortex (BA9)	Brain	GTEX-1445S-0011-R10a-SM-5PNWB
Brain - Frontal Cortex (BA9)	Brain	GTEX-145LS-0011-R10a-SM-5PNUQ
Brain - Frontal Cortex (BA9)	Brain	GTEX-145LU-0011-R10a-SM-5PNWJ
Brain - Frontal Cortex (BA9)	Brain	GTEX-145MG-0011-R10a-SM-5PNWG
Brain - Frontal Cortex (BA9)	Brain	GTEX-145MH-0011-R10b-SM-5PNUK
Brain - Frontal Cortex (BA9)	Brain	GTEX-14753-0011-R10b-SM-5S2WE
Brain - Frontal Cortex (BA9)	Brain	GTEX-1477Z-0011-R10b-SM-5S2RF
Brain - Frontal Cortex (BA9)	Brain	GTEX-147F4-0011-R10b-SM-5S2WC
Brain - Frontal Cortex (BA9)	Brain	GTEX-147GR-0011-R10b-SM-5S2UM
Brain - Frontal Cortex (BA9)	Brain	GTEX-14ASI-0011-R10b-SM-5S2VZ
Brain - Frontal Cortex (BA9)	Brain	GTEX-14BIL-0011-R10a-SM-5SI75
Brain - Frontal Cortex (BA9)	Brain	GTEX-14BIN-0011-R10a-SM-5S2UA
Brain - Frontal Cortex (BA9)	Brain	GTEX-14C39-0011-R10b-SM-5YYBE
Brain - Frontal Cortex (BA9)	Brain	GTEX-14C5O-0011-R10a-SM-6872F
Brain - Frontal Cortex (BA9)	Brain	GTEX-14E6D-0011-R10a-SM-5YY9C
Brain - Frontal Cortex (BA9)	Brain	GTEX-14E7W-0011-R10a-SM-5YYAX

Brain - Frontal Cortex (BA9)	Brain	GTEX-14JIY-0011-R10a-SM-6AJAO
Brain - Frontal Cortex (BA9)	Brain	GTEX-14LZ3-0011-R10b-SM-6AJA9
Brain - Frontal Cortex (BA9)	Brain	GTEX-14PJM-0011-R10a-SM-686ZP
Brain - Frontal Cortex (BA9)	Brain	GTEX-14PJO-0011-R10a-SM-686ZS
Brain - Frontal Cortex (BA9)	Brain	GTEX-14PN4-0011-R10b-SM-69LP2
Brain - Frontal Cortex (BA9)	Brain	GTEX-14PQA-0011-R10a-SM-6AJAA
Brain - Frontal Cortex (BA9)	Brain	GTEX-15CHQ-0011-R10b-SM-6AJBV
Brain - Frontal Cortex (BA9)	Brain	GTEX-15DCD-0011-R10b-SM-6LPII
Brain - Frontal Cortex (BA9)	Brain	GTEX-15DDE-0011-R10a-SM-7KULA
Brain - Frontal Cortex (BA9)	Brain	GTEX-15G19-0011-R10b-SM-7KUG4
Brain - Frontal Cortex (BA9)	Brain	GTEX-16GPK-0011-R10b-SM-7MKFG
Brain - Frontal Cortex (BA9)	Brain	GTEX-16XZZ-0011-R10b-SM-7LT91
Brain - Frontal Cortex (BA9)	Brain	GTEX-16YQH-0011-R10b-SM-7LT8U
Brain - Frontal Cortex (BA9)	Brain	GTEX-16Z82-0011-R10a-SM-79OJX
Brain - Frontal Cortex (BA9)	Brain	GTEX-17EVP-0011-R10a-SM-7IGLP
Brain - Frontal Cortex (BA9)	Brain	GTEX-17F97-0011-R10b-SM-793BB
Brain - Frontal Cortex (BA9)	Brain	GTEX-17HG3-0011-R10b-SM-7LT97
Brain - Frontal Cortex (BA9)	Brain	GTEX-17HII-0011-R10a-SM-79OMY
Brain - Frontal Cortex (BA9)	Brain	GTEX-17JCI-0011-R10b-SM-718A2
Brain - Frontal Cortex (BA9)	Brain	GTEX-18464-0011-R10a-SM-72D6X
Brain - Frontal Cortex (BA9)	Brain	GTEX-18A6Q-0011-R10b-SM-72D7A
Brain - Frontal Cortex (BA9)	Brain	GTEX-N7MS-0011-R10A-SM-2HMJK
Brain - Frontal Cortex (BA9)	Brain	GTEX-N7MT-0011-R10A-SM-2I3E1
Brain - Frontal Cortex (BA9)	Brain	GTEX-NL3H-0011-R10A-SM-2I3E9
Brain - Frontal Cortex (BA9)	Brain	GTEX-NL4W-0011-R10A-SM-2I3DY
Brain - Frontal Cortex (BA9)	Brain	GTEX-NPJ7-0011-R10A-SM-2I3E5
Brain - Frontal Cortex (BA9)	Brain	GTEX-NPJ8-0011-R10A-SM-2YUMO

Brain - Frontal Cortex (BA9)	Brain	GTEX-OXRN-0011-R10A-SM-2I5GC
Brain - Frontal Cortex (BA9)	Brain	GTEX-OXRO-0011-R10A-SM-2I5EH
Brain - Frontal Cortex (BA9)	Brain	GTEX-P44H-0011-R10A-SM-2XCEK
Brain - Frontal Cortex (BA9)	Brain	GTEX-Q2AG-0011-R10A-SM-2HMLA
Brain - Frontal Cortex (BA9)	Brain	GTEX-QDT8-0011-R10A-SM-32PKG
Brain - Frontal Cortex (BA9)	Brain	GTEX-QMR6-0011-R10A-SM-32PKO
Brain - Frontal Cortex (BA9)	Brain	GTEX-QVJO-0011-R10A-SM-2S1QJ
Brain - Frontal Cortex (BA9)	Brain	GTEX-QVUS-0011-R10A-SM-3GIK3
Brain - Frontal Cortex (BA9)	Brain	GTEX-S7SE-0011-R10A-SM-2XCDF
Brain - Frontal Cortex (BA9)	Brain	GTEX-T5JC-0011-R10A-SM-32PM2
Brain - Frontal Cortex (BA9)	Brain	GTEX-T6MN-0011-R10A-SM-32QP7
Brain - Frontal Cortex (BA9)	Brain	GTEX-TSE9-0011-R10A-SM-3DB7O
Brain - Frontal Cortex (BA9)	Brain	GTEX-WL46-0011-R10A-SM-3MJFQ
Brain - Frontal Cortex (BA9)	Brain	GTEX-WVLH-0011-R10A-SM-3MJFM
Brain - Frontal Cortex (BA9)	Brain	GTEX-WWYW-0011-R10A-SM-3NB35
Brain - Frontal Cortex (BA9)	Brain	GTEX-X261-0011-R10B-SM-4E3JT
Brain - Frontal Cortex (BA9)	Brain	GTEX-X4EP-0011-R10B-SM-4QASJ
Brain - Frontal Cortex (BA9)	Brain	GTEX-X4XX-0011-R10B-SM-46MWO
Brain - Frontal Cortex (BA9)	Brain	GTEX-X585-0011-R10A-SM-46MUY
Brain - Frontal Cortex (BA9)	Brain	GTEX-XLM4-0011-R10A-SM-4AT5P
Brain - Frontal Cortex (BA9)	Brain	GTEX-Y8DK-0011-R10A-SM-4SOK1
Brain - Frontal Cortex (BA9)	Brain	GTEX-YFC4-0011-R10a-SM-4SOK5
Brain - Frontal Cortex (BA9)	Brain	GTEX-YJ89-0011-R10a-SM-4SOK9
Brain - Frontal Cortex (BA9)	Brain	GTEX-Z93S-0011-R10a-SM-4RGNM
Brain - Frontal Cortex (BA9)	Brain	GTEX-ZAB4-0011-R10a-SM-4SOKH
Brain - Frontal Cortex (BA9)	Brain	GTEX-ZDXO-0011-R10a-SM-4WWD8
Brain - Frontal Cortex (BA9)	Brain	GTEX-ZF28-0011-R10a-SM-4WWEH

Brain - Frontal Cortex (BA9)	Brain	GTEX-ZUA1-0011-R10a-
SM-51MT6
Brain - Frontal Cortex (BA9)	Brain	GTEX-ZV68-0011-R10a-
SM-51MT7
Brain - Frontal Cortex (BA9)	Brain	GTEX-ZVT3-0011-R10b-
SM-57WB6
Brain - Frontal Cortex (BA9)	Brain	GTEX-ZVZQ-0011-R10b-
SM-51MRT
Brain - Frontal Cortex (BA9)	Brain	GTEX-ZXG5-0011-R10a-
SM-57WDD
Brain - Hippocampus	Brain	GTEX-11GSO-0011-R1b-SM-57WD3
Brain - Hippocampus	Brain	GTEX-11ONC-0011-R1a-SM-57WD4
Brain - Hippocampus	Brain	GTEX-11UD1-0011-R1b-SM-5BC7D
Brain - Hippocampus	Brain	GTEX-11ZTS-0011-R1a-SM-5BC71
Brain - Hippocampus	Brain	GTEX-11ZVC-0011-R1b-SM-5BC6M
Brain - Hippocampus	Brain	GTEX-1212Z-0011-R1b-SM-5BC72
Brain - Hippocampus	Brain	GTEX-12WSD-0011-R1b-SM-5LZVY
Brain - Hippocampus	Brain	GTEX-12WSE-0011-R1b-SM-5GU4V
Brain - Hippocampus	Brain	GTEX-12WSF-0011-R1a-SM-5DUVI
Brain - Hippocampus	Brain	GTEX-12WSH-0011-R1a-SM-5GU5V
Brain - Hippocampus	Brain	GTEX-12WSM-0011-R1a-SM-5LZW6
Brain - Hippocampus	Brain	GTEX-12ZZX-0011-R1a-SM-5EQ6B
Brain - Hippocampus	Brain	GTEX-13112-0011-R1b-SM-5HL7K
Brain - Hippocampus	Brain	GTEX-1313W-0011-R1b-SM-5EQ4A
Brain - Hippocampus	Brain	GTEX-131XH-0011-R1a-SM-5DUWA
Brain - Hippocampus	Brain	GTEX-131YS-0011-R1a-SM-5DUXC
Brain - Hippocampus	Brain	GTEX-132Q8-0011-R1a-SM-5EGLK
Brain - Hippocampus	Brain	GTEX-1399T-0011-R1b-SM-5DUWN
Brain - Hippocampus	Brain	GTEX-139T8-0011-R1a-SM-5HL75
Brain - Hippocampus	Brain	GTEX-139TT-0011-R1a-SM-5LZVD
Brain - Hippocampus	Brain	GTEX-13CF2-0011-R1a-SM-5LZY1
Brain - Hippocampus	Brain	GTEX-13FHO-0011-R1b-SM-5KLZG
Brain - Hippocampus	Brain	GTEX-13FHP-0011-R1b-SM-5K7XL
Brain - Hippocampus	Brain	GTEX-13FLW-0011-R1b-SM-5LZX2
Brain - Hippocampus	Brain	GTEX-13FTY-0011-R1a-SM-5LZXE
Brain - Hippocampus	Brain	GTEX-13FXS-0011-R1a-SM-5K7U6
Brain - Hippocampus	Brain	GTEX-13G51-0011-R1a-SM-5LZYR
Brain - Hippocampus	Brain	GTEX-13JUV-0011-R1a-SM-5LZY4
Brain - Hippocampus	Brain	GTEX-13JVG-0011-R1a-SM-5KM32
Brain - Hippocampus	Brain	GTEX-13N1W-0011-R1b-SM-5MR4T
Brain - Hippocampus	Brain	GTEX-13N2G-0011-R1b-SM-5MR3G
Brain - Hippocampus	Brain	GTEX-13NYB-0011-R1b-SM-5KM4F
Brain - Hippocampus	Brain	GTEX-13NYS-0011-R1b-SM-5MR55
Brain - Hippocampus	Brain	GTEX-13NZ8-0011-R1b-SM-5KM3V
Brain - Hippocampus	Brain	GTEX-13NZA-0011-R1b-SM-5LUAF
Brain - Hippocampus	Brain	GTEX-13O3O-0011-R1b-SM-5KM2K
Brain - Hippocampus	Brain	GTEX-13O3Q-0011-R1b-SM-5KM3L
Brain - Hippocampus	Brain	GTEX-13OVJ-0011-R1b-SM-5L3I6
Brain - Hippocampus	Brain	GTEX-13OVL-0011-R1a-SM-5L3H5
Brain - Hippocampus	Brain	GTEX-13OW5-0011-R1b-SM-5L3GE
Brain - Hippocampus	Brain	GTEX-13OW6-0011-R1a-SM-5L3HQ
Brain - Hippocampus	Brain	GTEX-13OW7-0011-R1b-SM-5L3HF
Brain - Hippocampus	Brain	GTEX-13OW8-0011-R1a-SM-5L3HI
Brain - Hippocampus	Brain	GTEX-13PDP-0011-R1a-SM-5PNX5

Brain - Hippocampus	Brain	GTEX-13QIC-0011-R1a-SM-5O9CJ
Brain - Hippocampus	Brain	GTEX-13QJC-0011-R1b-SM-5S2UU
Brain - Hippocampus	Brain	GTEX-13RTJ-0011-R1a-SM-5O9D9
Brain - Hippocampus	Brain	GTEX-13RTL-0011-R1a-SM-5PNZ8
Brain - Hippocampus	Brain	GTEX-13S7M-0011-R1b-SM-5PNZE
Brain - Hippocampus	Brain	GTEX-13SLW-0011-R1a-SM-5S2W7
Brain - Hippocampus	Brain	GTEX-13X6I-0011-R1b-SM-5PNZC
Brain - Hippocampus	Brain	GTEX-144GL-0011-R1a-SM-5PNZ5
Brain - Hippocampus	Brain	GTEX-144GO-0011-R1a-SM-5PNWO
Brain - Hippocampus	Brain	GTEX-145LS-0011-R1b-SM-5PNUP
Brain - Hippocampus	Brain	GTEX-145LU-0011-R1a-SM-5Q5CE
Brain - Hippocampus	Brain	GTEX-145MH-0011-R1b-SM-5PNWY
Brain - Hippocampus	Brain	GTEX-14ABY-0011-R1a-SM-6EU2W
Brain - Hippocampus	Brain	GTEX-14ASI-0011-R1b-SM-5S2TY
Brain - Hippocampus	Brain	GTEX-14BIM-0011-R1a-SM-69LQI
Brain - Hippocampus	Brain	GTEX-14C5O-0011-R1b-SM-6AJBW
Brain - Hippocampus	Brain	GTEX-14E6D-0011-R1b-SM-5YYAW
Brain - Hippocampus	Brain	GTEX-14E7W-0011-R1b-SM-5YYAT
Brain - Hippocampus	Brain	GTEX-14JIY-0011-R1a-SM-68713
Brain - Hippocampus	Brain	GTEX-14LLW-0011-R1a-SM-5ZZVD
Brain - Hippocampus	Brain	GTEX-14LZ3-0011-R1a-SM-69LP5
Brain - Hippocampus	Brain	GTEX-14PJM-0011-R1a-SM-6EU2Z
Brain - Hippocampus	Brain	GTEX-14PJO-0011-R1b-SM-6AJAK
Brain - Hippocampus	Brain	GTEX-14PN4-0011-R1b-SM-69LP8
Brain - Hippocampus	Brain	GTEX-14PQA-0011-R1b-SM-6EU1G
Brain - Hippocampus	Brain	GTEX-15CHQ-0011-R1a-SM-6EU31
Brain - Hippocampus	Brain	GTEX-15DCD-0011-R1b-SM-6LPIJ
Brain - Hippocampus	Brain	GTEX-15ER7-0011-R1b-SM-7KUE1
Brain - Hippocampus	Brain	GTEX-16GPK-0011-R1b-SM-7EWDH
Brain - Hippocampus	Brain	GTEX-16XZZ-0011-R1a-SM-7LTAH
Brain - Hippocampus	Brain	GTEX-16YQH-0011-R1a-SM-7LT8R
Brain - Hippocampus	Brain	GTEX-16Z82-0011-R1b-SM-7EPHZ
Brain - Hippocampus	Brain	GTEX-17EVP-0011-R1a-SM-7DHLA
Brain - Hippocampus	Brain	GTEX-17EVQ-0011-R1a-SM-7189S
Brain - Hippocampus	Brain	GTEX-17F97-0011-R1b-SM-7IGM9
Brain - Hippocampus	Brain	GTEX-17HG3-0011-R1a-SM-7LG6H
Brain - Hippocampus	Brain	GTEX-17HHY-0011-R1a-SM-7189V
Brain - Hippocampus	Brain	GTEX-17JCI-0011-R1b-SM-718A3
Brain - Hippocampus	Brain	GTEX-17MF6-0011-R1a-SM-72D6O
Brain - Hippocampus	Brain	GTEX-183WM-0011-R1a-SM-718A8
Brain - Hippocampus	Brain	GTEX-18A6Q-0011-R1b-SM-731DI
Brain - Hippocampus	Brain	GTEX-N7MS-0011-R1a-SM-2HMJG
Brain - Hippocampus	Brain	GTEX-N7MT-0011-R1a-SM-5SI7S
Brain - Hippocampus	Brain	GTEX-NL3H-0011-R1a-SM-48TDJ
Brain - Hippocampus	Brain	GTEX-NPJ7-0011-R1a-SM-3GACT
Brain - Hippocampus	Brain	GTEX-NPJ8-0011-R1a-SM-33HCB
Brain - Hippocampus	Brain	GTEX-OHPN-0011-R1A-SM-2I5GB
Brain - Hippocampus	Brain	GTEX-P44H-0011-R1A-SM-3NM8J
Brain - Hippocampus	Brain	GTEX-PVOW-0011-R1A-SM-32PL6
Brain - Hippocampus	Brain	GTEX-PWO3-0011-R1A-SM-2I5EW
Brain - Hippocampus	Brain	GTEX-Q2AG-0011-R1A-SM-2HMJI
Brain - Hippocampus	Brain	GTEX-QDT8-0011-R1A-SM-32PKS
Brain - Hippocampus	Brain	GTEX-QMR6-0011-R1A-SM-32PKW
Brain - Hippocampus	Brain	GTEX-QVJO-0011-R1A-SM-2S1QI

Brain - Hippocampus	Brain	GTEX-QVUS-0011-R1A-SM-3GAD2
Brain - Hippocampus	Brain	GTEX-R55E-0011-R1A-SM-2TC6N
Brain - Hippocampus	Brain	GTEX-RNOR-0011-R1A-SM-5SI8F
Brain - Hippocampus	Brain	GTEX-S7SE-0011-R1A-SM-2XCDE
Brain - Hippocampus	Brain	GTEX-T5JC-0011-R1A-SM-32PM6
Brain - Hippocampus	Brain	GTEX-T6MN-0011-R1A-SM-32QOY
Brain - Hippocampus	Brain	GTEX-TSE9-0011-R1A-SM-3DB7E
Brain - Hippocampus	Brain	GTEX-UTHO-0011-R1A-SM-3GIJO
Brain - Hippocampus	Brain	GTEX-WHSE-0011-R1A-SM-3P5ZK
Brain - Hippocampus	Brain	GTEX-WL46-0011-R1A-SM-3LK6M
Brain - Hippocampus	Brain	GTEX-WVLH-0011-R1A-SM-4MVOK
Brain - Hippocampus	Brain	GTEX-WWYW-0011-R1A-SM-3TW8G
Brain - Hippocampus	Brain	GTEX-WZTO-0011-R1B-SM-3NMAR
Brain - Hippocampus	Brain	GTEX-X4XX-0011-R1B-SM-3P622
Brain - Hippocampus	Brain	GTEX-X585-0011-R1B-SM-46MVE
Brain - Hippocampus	Brain	GTEX-XMD1-0011-R1A-SM-4AT4C
Brain - Hippocampus	Brain	GTEX-Y8DK-0011-R1A-SM-4RGLO
Brain - Hippocampus	Brain	GTEX-YFC4-0011-R1a-SM-4V6EH
Brain - Hippocampus	Brain	GTEX-YJ89-0011-R1a-SM-4RGLS
Brain - Hippocampus	Brain	GTEX-ZAB4-0011-R1a-SM-4RGNO
Brain - Hippocampus	Brain	GTEX-ZDXO-0011-R1a-SM-4WKF4
Brain - Hippocampus	Brain	GTEX-ZE7O-0011-R1a-SM-57WDM
Brain - Hippocampus	Brain	GTEX-ZF28-0011-R1a-SM-4WWD1
Brain - Hippocampus	Brain	GTEX-ZUA1-0011-R1a-SM-4YCE2
Brain - Hippocampus	Brain	GTEX-ZVZQ-0011-R1a-SM-57WBU
Brain - Hypothalamus	Brain	GTEX-11GSP-0011-R8b-SM-5NQ79
Brain - Hypothalamus	Brain	GTEX-11H98-0011-R8a-SM-5NQ8V
Brain - Hypothalamus	Brain	GTEX-11OF3-0011-R8b-SM-57WCS
Brain - Hypothalamus	Brain	GTEX-11ONC-0011-R8b-SM-5NQ87
Brain - Hypothalamus	Brain	GTEX-11WQC-0011-R8b-SM-57WCH
Brain - Hypothalamus	Brain	GTEX-11WQK-0011-R8a-SM-5BC7C
Brain - Hypothalamus	Brain	GTEX-11ZTS-0011-R8a-SM-57WDI
Brain - Hypothalamus	Brain	GTEX-11ZUS-0011-R8a-SM-5BC73
Brain - Hypothalamus	Brain	GTEX-12WSA-0011-R8a-SM-5P9EX
Brain - Hypothalamus	Brain	GTEX-12WSM-0011-R8a-SM-5DUWJ
Brain - Hypothalamus	Brain	GTEX-12ZZX-0011-R8a-SM-5DUW8
Brain - Hypothalamus	Brain	GTEX-13112-0011-R8b-SM-664OV
Brain - Hypothalamus	Brain	GTEX-1313W-0011-R8a-SM-5DUVM
Brain - Hypothalamus	Brain	GTEX-131XH-0011-R8a-SM-5EGLL
Brain - Hypothalamus	Brain	GTEX-131XW-0011-R8a-SM-5DUWY
Brain - Hypothalamus	Brain	GTEX-132Q8-0011-R8b-SM-5DUWX
Brain - Hypothalamus	Brain	GTEX-1399T-0011-R8b-SM-5IJD8
Brain - Hypothalamus	Brain	GTEX-13CF2-0011-R8b-SM-5IJCX
Brain - Hypothalamus	Brain	GTEX-13CIG-0011-R8b-SM-5LZX5
Brain - Hypothalamus	Brain	GTEX-13FLV-0011-R8a-SM-5LZZE
Brain - Hypothalamus	Brain	GTEX-13FLW-0011-R8b-SM-5J1MZ
Brain - Hypothalamus	Brain	GTEX-13FXS-0011-R8a-SM-5J1MN
Brain - Hypothalamus	Brain	GTEX-13G51-0011-R8b-SM-5LZZ4
Brain - Hypothalamus	Brain	GTEX-13IVO-0011-R8a-SM-5L3DY
Brain - Hypothalamus	Brain	GTEX-13JVG-0011-R8a-SM-5KM3E
Brain - Hypothalamus	Brain	GTEX-13N1W-0011-R8a-SM-5MR56
Brain - Hypothalamus	Brain	GTEX-13NYB-0011-R8a-SM-5KM4R
Brain - Hypothalamus	Brain	GTEX-13NYS-0011-R8a-SM-5MR5H
Brain - Hypothalamus	Brain	GTEX-13NZ8-0011-R8b-SM-5KM48

Brain - Hypothalamus	Brain	GTEX-13NZA-0011-R8b-SM-5KM2Q
Brain - Hypothalamus	Brain	GTEX-13O3O-0011-R8b-SM-5LUAL
Brain - Hypothalamus	Brain	GTEX-13O3Q-0011-R8b-SM-5KM2J
Brain - Hypothalamus	Brain	GTEX-13OVH-0011-R8b-SM-5MR35
Brain - Hypothalamus	Brain	GTEX-13OVJ-0011-R8b-SM-5L3FS
Brain - Hypothalamus	Brain	GTEX-13OVL-0011-R8a-SM-5L3HH
Brain - Hypothalamus	Brain	GTEX-13OW5-0011-R8a-SM-5L3GQ
Brain - Hypothalamus	Brain	GTEX-13OW6-0011-R8a-SM-5L3GF
Brain - Hypothalamus	Brain	GTEX-13OW7-0011-R8b-SM-5L3HR
Brain - Hypothalamus	Brain	GTEX-13OW8-0011-R8a-SM-5L3G5
Brain - Hypothalamus	Brain	GTEX-13PDP-0011-R8b-SM-5O9CS
Brain - Hypothalamus	Brain	GTEX-13QIC-0011-R8b-SM-5O9CV
Brain - Hypothalamus	Brain	GTEX-13QJC-0011-R8b-SM-5S2U6
Brain - Hypothalamus	Brain	GTEX-13RTJ-0011-R8b-SM-5O9DL
Brain - Hypothalamus	Brain	GTEX-13SLW-0011-R8b-SM-5SI64
Brain - Hypothalamus	Brain	GTEX-13SLX-0011-R8b-SM-5PNWZ
Brain - Hypothalamus	Brain	GTEX-13X6I-0011-R8a-SM-5PNZF
Brain - Hypothalamus	Brain	GTEX-13X6J-0011-R8b-SM-5PNUA
Brain - Hypothalamus	Brain	GTEX-13X6K-0011-R8b-SM-5PNZ4
Brain - Hypothalamus	Brain	GTEX-1445S-0011-R8a-SM-5PNWX
Brain - Hypothalamus	Brain	GTEX-145LS-0011-R8b-SM-5PNXA
Brain - Hypothalamus	Brain	GTEX-145LU-0011-R8b-SM-5PNZG
Brain - Hypothalamus	Brain	GTEX-145MH-0011-R8a-SM-5PNUL
Brain - Hypothalamus	Brain	GTEX-1477Z-0011-R8b-SM-5S2RG
Brain - Hypothalamus	Brain	GTEX-147F4-0011-R8b-SM-5S2UN
Brain - Hypothalamus	Brain	GTEX-14ASI-0011-R8b-SM-5S2UB
Brain - Hypothalamus	Brain	GTEX-14BIL-0011-R8b-SM-69LQK
Brain - Hypothalamus	Brain	GTEX-14C5O-0011-R8a-SM-5YYAG
Brain - Hypothalamus	Brain	GTEX-14JIY-0011-R8a-SM-6AJAP
Brain - Hypothalamus	Brain	GTEX-14LZ3-0011-R8b-SM-69LP6
Brain - Hypothalamus	Brain	GTEX-14PJM-0011-R8b-SM-69LPD
Brain - Hypothalamus	Brain	GTEX-14PJO-0011-R8a-SM-6AJAL
Brain - Hypothalamus	Brain	GTEX-14PN4-0011-R8a-SM-6EU1H
Brain - Hypothalamus	Brain	GTEX-14PQA-0011-R8b-SM-6AJAB
Brain - Hypothalamus	Brain	GTEX-15CHQ-0011-R8b-SM-6EU32
Brain - Hypothalamus	Brain	GTEX-15DCD-0011-R8b-SM-7KUEF
Brain - Hypothalamus	Brain	GTEX-15ER7-0011-R8a-SM-7KUL2
Brain - Hypothalamus	Brain	GTEX-15G19-0011-R8b-SM-7KUL1
Brain - Hypothalamus	Brain	GTEX-16GPK-0011-R8a-SM-79OMQ
Brain - Hypothalamus	Brain	GTEX-16NPV-0011-R8a-SM-7EPH8
Brain - Hypothalamus	Brain	GTEX-16XZZ-0011-R8b-SM-7LT8S
Brain - Hypothalamus	Brain	GTEX-16YQH-0011-R8a-SM-7DUFB
Brain - Hypothalamus	Brain	GTEX-16Z82-0011-R8a-SM-7DUEX
Brain - Hypothalamus	Brain	GTEX-17EVP-0011-R8b-SM-7DUF9
Brain - Hypothalamus	Brain	GTEX-17EVQ-0011-R8a-SM-7189T
Brain - Hypothalamus	Brain	GTEX-17F97-0011-R8a-SM-7DUET
Brain - Hypothalamus	Brain	GTEX-17HG3-0011-R8a-SM-79OK7
Brain - Hypothalamus	Brain	GTEX-17HII-0011-R8a-SM-79OK6
Brain - Hypothalamus	Brain	GTEX-183WM-0011-R8a-SM-7189W
Brain - Hypothalamus	Brain	GTEX-18464-0011-R8a-SM-72D4Z
Brain - Hypothalamus	Brain	GTEX-18465-0011-R8b-SM-7LG6O
Brain - Hypothalamus	Brain	GTEX-1B8SG-0011-R8b-SM-7IGNW
Brain - Hypothalamus	Brain	GTEX-1B996-0011-R8b-SM-79OOS
Brain - Hypothalamus	Brain	GTEX-N7MS-0011-R8a-SM-2YUMK

Brain - Hypothalamus	Brain	GTEX-N7MT-0011-R8a-SM-2I5GU
Brain - Hypothalamus	Brain	GTEX-NL3H-0011-R8a-SM-5S2VF
Brain - Hypothalamus	Brain	GTEX-NL4W-0011-R8a-SM-2I3G4
Brain - Hypothalamus	Brain	GTEX-NPJ7-0011-R8a-SM-2I3G2
Brain - Hypothalamus	Brain	GTEX-NPJ8-0011-R8a-SM-2HMLG
Brain - Hypothalamus	Brain	GTEX-P44H-0011-R8A-SM-2XCEL
Brain - Hypothalamus	Brain	GTEX-PWO3-0011-R8A-SM-2I5GD
Brain - Hypothalamus	Brain	GTEX-Q2AG-0011-R8A-SM-2HMK5
Brain - Hypothalamus	Brain	GTEX-QDT8-0011-R8A-SM-32PKE
Brain - Hypothalamus	Brain	GTEX-QMR6-0011-R8A-SM-32PKJ
Brain - Hypothalamus	Brain	GTEX-QVJO-0011-R8A-SM-447C7
Brain - Hypothalamus	Brain	GTEX-QVUS-0011-R8A-SM-3GAD7
Brain - Hypothalamus	Brain	GTEX-R55E-0011-R8A-SM-2TC66
Brain - Hypothalamus	Brain	GTEX-RU72-0011-R8A-SM-2TF61
Brain - Hypothalamus	Brain	GTEX-S7SE-0011-R8A-SM-2XCDG
Brain - Hypothalamus	Brain	GTEX-T5JC-0011-R8A-SM-32PLM
Brain - Hypothalamus	Brain	GTEX-T6MN-0011-R8A-SM-32QP3
Brain - Hypothalamus	Brain	GTEX-TSE9-0011-R8A-SM-3DB7R
Brain - Hypothalamus	Brain	GTEX-WHSE-0011-R8A-SM-3P5Z1
Brain - Hypothalamus	Brain	GTEX-WVLH-0011-R8A-SM-3MJFC
Brain - Hypothalamus	Brain	GTEX-WWYW-0011-R8A-SM-3NB3S
Brain - Hypothalamus	Brain	GTEX-WZTO-0011-R8A-SM-4E3II
Brain - Hypothalamus	Brain	GTEX-X261-0011-R8A-SM-4E3I5
Brain - Hypothalamus	Brain	GTEX-X4XX-0011-R8B-SM-46MWM
Brain - Hypothalamus	Brain	GTEX-X4XY-0011-R8A-SM-46MVC
Brain - Hypothalamus	Brain	GTEX-X585-0011-R8A-SM-46MUX
Brain - Hypothalamus	Brain	GTEX-XLM4-0011-R8A-SM-4AT44
Brain - Hypothalamus	Brain	GTEX-XMD1-0011-R8A-SM-4AT48
Brain - Hypothalamus	Brain	GTEX-YFC4-0011-R8a-SM-4SOK3
Brain - Hypothalamus	Brain	GTEX-YJ89-0011-R8b-SM-4SOK8
Brain - Hypothalamus	Brain	GTEX-Z93S-0011-R8a-SM-4RGNL
Brain - Hypothalamus	Brain	GTEX-ZAB4-0011-R8b-SM-4SOKF
Brain - Hypothalamus	Brain	GTEX-ZDXO-0011-R8a-SM-4WWD7
Brain - Hypothalamus	Brain	GTEX-ZF28-0011-R8a-SM-4WWFW
Brain - Hypothalamus	Brain	GTEX-ZUA1-0011-R8b-SM-51MST
Brain - Hypothalamus	Brain	GTEX-ZVT3-0011-R8b-SM-57WDJ
Brain - Hypothalamus	Brain	GTEX-ZVZQ-0011-R8a-SM-51MR5
Brain - Hypothalamus	Brain	GTEX-ZXG5-0011-R8a-SM-57WD1
Brain - Nucleus accumbens (basal ganglia)	Brain	GTEX-11GSP-0011-R6b-SM-57WBQ
Brain - Nucleus accumbens (basal ganglia)	Brain	GTEX-11NV4-0011-R6a-SM-57WD5
Brain - Nucleus accumbens (basal ganglia)	Brain	GTEX-11OF3-0011-R6b-SM-57WBG
Brain - Nucleus accumbens (basal ganglia)	Brain	GTEX-11WQC-0011-R6a-SM-5BC77
Brain - Nucleus accumbens (basal ganglia)	Brain	GTEX-12584-0011-R6a-SM-5NQ7C
Brain - Nucleus accumbens (basal ganglia)	Brain	GTEX-12WSC-0011-R6b-SM-5LU99
Brain - Nucleus accumbens (basal ganglia)	Brain	GTEX-12WSD-0011-R6b-SM-5P9JU
Brain - Nucleus accumbens (basal ganglia)	Brain	GTEX-12WSE-0011-R6b-SM-5LZV5

Brain - Nucleus accumbens (basal ganglia) Brain GTEX-12WSF-0011-R6b-SM-5HL8W
Brain - Nucleus accumbens (basal ganglia) Brain GTEX-12WSH-0011-R6b-SM-5P9F4
Brain - Nucleus accumbens (basal ganglia) Brain GTEX-12WSM-0011-R6b-SM-5EQ6N
Brain - Nucleus accumbens (basal ganglia) Brain GTEX-12ZZW-0011-R6a-SM-5DUVZ
Brain - Nucleus accumbens (basal ganglia) Brain GTEX-12ZZX-0011-R6a-SM-5HL8L
Brain - Nucleus accumbens (basal ganglia) Brain GTEX-12ZZY-0011-R6b-SM-5EGLA
Brain - Nucleus accumbens (basal ganglia) Brain GTEX-12ZZZ-0011-R6b-SM-5EQ5B
Brain - Nucleus accumbens (basal ganglia) Brain GTEX-13112-0011-R6b-SM-5DUVK
Brain - Nucleus accumbens (basal ganglia) Brain GTEX-1313W-0011-R6a-SM-5K7XO
Brain - Nucleus accumbens (basal ganglia) Brain GTEX-131XH-0011-R6a-SM-5KM2G
Brain - Nucleus accumbens (basal ganglia) Brain GTEX-131XW-0011-R6b-SM-5K7XY
Brain - Nucleus accumbens (basal ganglia) Brain GTEX-132Q8-0011-R6a-SM-5K7YN
Brain - Nucleus accumbens (basal ganglia) Brain GTEX-1399T-0011-R6b-SM-5K7YL
Brain - Nucleus accumbens (basal ganglia) Brain GTEX-139TS-0011-R6a-SM-5J2OB
Brain - Nucleus accumbens (basal ganglia) Brain GTEX-139TT-0011-R6b-SM-5J2ON
Brain - Nucleus accumbens (basal ganglia) Brain GTEX-13FHO-0011-R6b-SM-5LZX1
Brain - Nucleus accumbens (basal ganglia) Brain GTEX-13FHP-0011-R6b-SM-5LZY6
Brain - Nucleus accumbens (basal ganglia) Brain GTEX-13FLV-0011-R6a-SM-5KLZ4
Brain - Nucleus accumbens (basal ganglia) Brain GTEX-13FLW-0011-R6b-SM-5L3EN
Brain - Nucleus accumbens (basal ganglia) Brain GTEX-13FXS-0011-R6b-SM-5L3F7
Brain - Nucleus accumbens (basal ganglia) Brain GTEX-13G51-0011-R6b-SM-5LZX4
Brain - Nucleus accumbens (basal ganglia) Brain GTEX-13IVO-0011-R6b-SM-5K7X9
Brain - Nucleus accumbens (basal ganglia) Brain GTEX-13JUV-0011-R6b-SM-5LZYG
Brain - Nucleus accumbens (basal ganglia) Brain GTEX-13JVG-0011-R6a-SM-5MR3E
Brain - Nucleus accumbens (basal ganglia) Brain GTEX-13N1W-0011-R6a-SM-5MR43
Brain - Nucleus accumbens (basal ganglia) Brain GTEX-13N2G-0011-R6b-SM-5MR3P
Brain - Nucleus accumbens (basal ganglia) Brain GTEX-13NYB-0011-R6b-SM-5MR51

Brain - Nucleus accumbens (basal ganglia) Brain GTEX-13NYS-0011-R6b-SM-5MR3R
Brain - Nucleus accumbens (basal ganglia) Brain GTEX-13O3O-0011-R6b-SM-5P9GY
Brain - Nucleus accumbens (basal ganglia) Brain GTEX-13O3Q-0011-R6a-SM-5KM2V
Brain - Nucleus accumbens (basal ganglia) Brain GTEX-13OVH-0011-R6b-SM-5LUAX
Brain - Nucleus accumbens (basal ganglia) Brain GTEX-13OVJ-0011-R6b-SM-5L3GH
Brain - Nucleus accumbens (basal ganglia) Brain GTEX-13OVL-0011-R6a-SM-5L3G4
Brain - Nucleus accumbens (basal ganglia) Brain GTEX-13OW5-0011-R6b-SM-5O9BW
Brain - Nucleus accumbens (basal ganglia) Brain GTEX-13OW6-0011-R6b-SM-5L3GR
Brain - Nucleus accumbens (basal ganglia) Brain GTEX-13OW8-0011-R6b-SM-5L3I3
Brain - Nucleus accumbens (basal ganglia) Brain GTEX-13PDP-0011-R6b-SM-5O9D5
Brain - Nucleus accumbens (basal ganglia) Brain GTEX-13PL6-0011-R6a-SM-5O9C5
Brain - Nucleus accumbens (basal ganglia) Brain GTEX-13QJC-0011-R6a-SM-5S2VI
Brain - Nucleus accumbens (basal ganglia) Brain GTEX-13RTJ-0011-R6a-SM-5SI74
Brain - Nucleus accumbens (basal ganglia) Brain GTEX-13S7M-0011-R6b-SM-5O9D8
Brain - Nucleus accumbens (basal ganglia) Brain GTEX-13SLW-0011-R6a-SM-5O9BH
Brain - Nucleus accumbens (basal ganglia) Brain GTEX-13SLX-0011-R6a-SM-5PNX4
Brain - Nucleus accumbens (basal ganglia) Brain GTEX-13VXU-0011-R6a-SM-5SI71
Brain - Nucleus accumbens (basal ganglia) Brain GTEX-13X6J-0011-R6a-SM-5PNUB
Brain - Nucleus accumbens (basal ganglia) Brain GTEX-13X6K-0011-R6a-SM-5P9K6
Brain - Nucleus accumbens (basal ganglia) Brain GTEX-1445S-0011-R6b-SM-5PNUF
Brain - Nucleus accumbens (basal ganglia) Brain GTEX-144GL-0011-R6a-SM-5P9HW
Brain - Nucleus accumbens (basal ganglia) Brain GTEX-144GO-0011-R6b-SM-5S2V7
Brain - Nucleus accumbens (basal ganglia) Brain GTEX-145LS-0011-R6b-SM-5PNWL
Brain - Nucleus accumbens (basal ganglia) Brain GTEX-145LU-0011-R6a-SM-5S2V6
Brain - Nucleus accumbens (basal ganglia) Brain GTEX-145MH-0011-R6a-SM-5PNZ7
Brain - Nucleus accumbens (basal ganglia) Brain GTEX-145MI-0011-R6a-SM-5PNZA
Brain - Nucleus accumbens (basal ganglia) Brain GTEX-147GR-0011-R6b-SM-5S2RO

Brain - Nucleus accumbens (basal ganglia) Brain GTEX-14ABY-0011-R6a-SM-5PNWR
Brain - Nucleus accumbens (basal ganglia) Brain GTEX-14ASI-0011-R6a-SM-5S2UZ
Brain - Nucleus accumbens (basal ganglia) Brain GTEX-14BIM-0011-R6b-SM-5S2VB
Brain - Nucleus accumbens (basal ganglia) Brain GTEX-14BIN-0011-R6a-SM-5S2RH
Brain - Nucleus accumbens (basal ganglia) Brain GTEX-14BMV-0011-R6b-SM-5YY9I
Brain - Nucleus accumbens (basal ganglia) Brain GTEX-14C39-0011-R6b-SM-5YYAZ
Brain - Nucleus accumbens (basal ganglia) Brain GTEX-14C5O-0011-R6a-SM-5YYB6
Brain - Nucleus accumbens (basal ganglia) Brain GTEX-14E7W-0011-R6b-SM-5ZZV3
Brain - Nucleus accumbens (basal ganglia) Brain GTEX-14JG1-0011-R6b-SM-5YYAU
Brain - Nucleus accumbens (basal ganglia) Brain GTEX-14JIY-0011-R6b-SM-68714
Brain - Nucleus accumbens (basal ganglia) Brain GTEX-14LZ3-0011-R6a-SM-686ZO
Brain - Nucleus accumbens (basal ganglia) Brain GTEX-14PJM-0011-R6a-SM-6872G
Brain - Nucleus accumbens (basal ganglia) Brain GTEX-14PN4-0011-R6b-SM-69LQU
Brain - Nucleus accumbens (basal ganglia) Brain GTEX-14PQA-0011-R6b-SM-6AJAM
Brain - Nucleus accumbens (basal ganglia) Brain GTEX-15CHQ-0011-R6b-SM-69LOX
Brain - Nucleus accumbens (basal ganglia) Brain GTEX-15DCD-0011-R6b-SM-6M46L
Brain - Nucleus accumbens (basal ganglia) Brain GTEX-15DYW-0011-R6b-SM-6PALA
Brain - Nucleus accumbens (basal ganglia) Brain GTEX-15EO6-0011-R6a-SM-7KULK
Brain - Nucleus accumbens (basal ganglia) Brain GTEX-15ER7-0011-R6a-SM-6M46M
Brain - Nucleus accumbens (basal ganglia) Brain GTEX-15G19-0011-R6b-SM-7KUM3
Brain - Nucleus accumbens (basal ganglia) Brain GTEX-15RJE-0011-R6a-SM-7DHL8
Brain - Nucleus accumbens (basal ganglia) Brain GTEX-16XZZ-0011-R6a-SM-7IGNE
Brain - Nucleus accumbens (basal ganglia) Brain GTEX-16YQH-0011-R6b-SM-7938T
Brain - Nucleus accumbens (basal ganglia) Brain GTEX-16Z82-0011-R6a-SM-7IGLV
Brain - Nucleus accumbens (basal ganglia) Brain GTEX-17EVP-0011-R6b-SM-7LT99
Brain - Nucleus accumbens (basal ganglia) Brain GTEX-17EVQ-0011-R6a-SM-7189U
Brain - Nucleus accumbens (basal ganglia) Brain GTEX-17F97-0011-R6a-SM-7EPGS

Brain - Nucleus accumbens (basal ganglia) Brain GTEX-17HG3-0011-R6b-SM-79OK8
Brain - Nucleus accumbens (basal ganglia) Brain GTEX-17HHY-0011-R6b-SM-7189Z
Brain - Nucleus accumbens (basal ganglia) Brain GTEX-17JCI-0011-R6a-SM-72D6D
Brain - Nucleus accumbens (basal ganglia) Brain GTEX-17MF6-0011-R6b-SM-7LG6G
Brain - Nucleus accumbens (basal ganglia) Brain GTEX-183WM-0011-R6b-SM-72D66
Brain - Nucleus accumbens (basal ganglia) Brain GTEX-18464-0011-R6a-SM-731CA
Brain - Nucleus accumbens (basal ganglia) Brain GTEX-18465-0011-R6a-SM-7LG6M
Brain - Nucleus accumbens (basal ganglia) Brain GTEX-18A6Q-0011-R6a-SM-7LG6R
Brain - Nucleus accumbens (basal ganglia) Brain GTEX-1B933-0011-R6b-SM-79OOQ
Brain - Nucleus accumbens (basal ganglia) Brain GTEX-1B996-0011-R6b-SM-793AQ
Brain - Nucleus accumbens (basal ganglia) Brain GTEX-1C64N-0011-R6a-SM-7IGO9
Brain - Nucleus accumbens (basal ganglia) Brain GTEX-N7MS-0011-R6a-SM-2HMJ4
Brain - Nucleus accumbens (basal ganglia) Brain GTEX-N7MT-0011-R6a-SM-2I3G3
Brain - Nucleus accumbens (basal ganglia) Brain GTEX-NL3H-0011-R6a-SM-2I3G8
Brain - Nucleus accumbens (basal ganglia) Brain GTEX-NL4W-0011-R6a-SM-2I3GA
Brain - Nucleus accumbens (basal ganglia) Brain GTEX-NPJ7-0011-R6a-SM-2I3G7
Brain - Nucleus accumbens (basal ganglia) Brain GTEX-NPJ8-0011-R6a-SM-2HMKB
Brain - Nucleus accumbens (basal ganglia) Brain GTEX-OHPN-0011-R6A-SM-2I5FH
Brain - Nucleus accumbens (basal ganglia) Brain GTEX-PWO3-0011-R6A-SM-2I5F3
Brain - Nucleus accumbens (basal ganglia) Brain GTEX-Q2AG-0011-R6A-SM-2HML7
Brain - Nucleus accumbens (basal ganglia) Brain GTEX-QDT8-0011-R6A-SM-32PKI
Brain - Nucleus accumbens (basal ganglia) Brain GTEX-QMR6-0011-R6A-SM-32PKP
Brain - Nucleus accumbens (basal ganglia) Brain GTEX-QVJO-0011-R6A-SM-2S1QN
Brain - Nucleus accumbens (basal ganglia) Brain GTEX-QVUS-0011-R6A-SM-3GACX
Brain - Nucleus accumbens (basal ganglia) Brain GTEX-R55E-0011-R6A-SM-2TC5T
Brain - Nucleus accumbens (basal ganglia) Brain GTEX-RU72-0011-R6A-SM-2TF71
Brain - Nucleus accumbens (basal ganglia) Brain GTEX-S7PM-0011-R6A-SM-3NM8F

Brain - Nucleus accumbens (basal ganglia) Brain GTEX-S7SE-0011-R6A-SM-2XCD9
Brain - Nucleus accumbens (basal ganglia) Brain GTEX-T2IS-0011-R6A-SM-32QP2
Brain - Nucleus accumbens (basal ganglia) Brain GTEX-T5JC-0011-R6A-SM-5SI89
Brain - Nucleus accumbens (basal ganglia) Brain GTEX-T6MN-0011-R6A-SM-32QP8
Brain - Nucleus accumbens (basal ganglia) Brain GTEX-TSE9-0011-R6A-SM-3DB7L
Brain - Nucleus accumbens (basal ganglia) Brain GTEX-UTHO-0011-R6A-SM-3GIJW
Brain - Nucleus accumbens (basal ganglia) Brain GTEX-WHSE-0011-R6A-SM-3P5ZP
Brain - Nucleus accumbens (basal ganglia) Brain GTEX-WL46-0011-R6A-SM-3LK6X
Brain - Nucleus accumbens (basal ganglia) Brain GTEX-WVLH-0011-R6A-SM-3MJFZ
Brain - Nucleus accumbens (basal ganglia) Brain GTEX-WWYW-0011-R6A-SM-3NB3G
Brain - Nucleus accumbens (basal ganglia) Brain GTEX-WZTO-0011-R6B-SM-4E3J6
Brain - Nucleus accumbens (basal ganglia) Brain GTEX-X261-0011-R6B-SM-4E3J8
Brain - Nucleus accumbens (basal ganglia) Brain GTEX-X4XX-0011-R6B-SM-46MWP
Brain - Nucleus accumbens (basal ganglia) Brain GTEX-X4XY-0011-R6B-SM-46MVB
Brain - Nucleus accumbens (basal ganglia) Brain GTEX-X585-0011-R6A-SM-46MVJ
Brain - Nucleus accumbens (basal ganglia) Brain GTEX-XLM4-0011-R6A-SM-4AT4B
Brain - Nucleus accumbens (basal ganglia) Brain GTEX-XMD1-0011-R6A-SM-4AT5K
Brain - Nucleus accumbens (basal ganglia) Brain GTEX-Y8DK-0011-R6A-SM-4V6EG
Brain - Nucleus accumbens (basal ganglia) Brain GTEX-YFC4-0011-R6b-SM-4V6E1
Brain - Nucleus accumbens (basal ganglia) Brain GTEX-YJ89-0011-R6a-SM-4V6GN
Brain - Nucleus accumbens (basal ganglia) Brain GTEX-Z93S-0011-R6b-SM-4RGNJ
Brain - Nucleus accumbens (basal ganglia) Brain GTEX-ZAB4-0011-R6a-SM-4SOKD
Brain - Nucleus accumbens (basal ganglia) Brain GTEX-ZDXO-0011-R6a-SM-4WWCT
Brain - Nucleus accumbens (basal ganglia) Brain GTEX-ZE7O-0011-R6a-SM-57WCI
Brain - Nucleus accumbens (basal ganglia) Brain GTEX-ZE9C-0011-R6a-SM-4WWCW
Brain - Nucleus accumbens (basal ganglia) Brain GTEX-ZF28-0011-R6a-SM-4WKHI
Brain - Nucleus accumbens (basal ganglia) Brain GTEX-ZUA1-0011-R6b-SM-4YCDD

Brain - Nucleus accumbens (basal ganglia) Brain GTEX-ZV68-0011-R6a-SM-51MSR
Brain - Nucleus accumbens (basal ganglia) Brain GTEX-ZVT3-0011-R6a-SM-51MSU
Brain - Nucleus accumbens (basal ganglia) Brain GTEX-ZVZQ-0011-R6b-SM-57WAY
Brain - Nucleus accumbens (basal ganglia) Brain GTEX-ZXG5-0011-R6a-SM-57WBZ
Brain - Putamen (basal ganglia) Brain GTEX-11GSP-0011-R7b-SM-57WC3
Brain - Putamen (basal ganglia) Brain GTEX-11NV4-0011-R7a-SM-57WDH
Brain - Putamen (basal ganglia) Brain GTEX-11OF3-0011-R7a-SM-57WCG
Brain - Putamen (basal ganglia) Brain GTEX-11WQC-0011-R7b-SM-5BC7A
Brain - Putamen (basal ganglia) Brain GTEX-12WSA-0011-R7b-SM-5GU5U
Brain - Putamen (basal ganglia) Brain GTEX-12WSC-0011-R7b-SM-5LU9L
Brain - Putamen (basal ganglia) Brain GTEX-12WSD-0011-R7b-SM-5PNWE
Brain - Putamen (basal ganglia) Brain GTEX-12WSE-0011-R7a-SM-5PNWF
Brain - Putamen (basal ganglia) Brain GTEX-12WSF-0011-R7b-SM-5HL99
Brain - Putamen (basal ganglia) Brain GTEX-12WSI-0011-R7b-SM-5GU78
Brain - Putamen (basal ganglia) Brain GTEX-12WSM-0011-R7b-SM-5EGLD
Brain - Putamen (basal ganglia) Brain GTEX-12ZZW-0011-R7a-SM-5DUWC
Brain - Putamen (basal ganglia) Brain GTEX-12ZZX-0011-R7b-SM-5DUVV
Brain - Putamen (basal ganglia) Brain GTEX-12ZZY-0011-R7a-SM-5EGLB
Brain - Putamen (basal ganglia) Brain GTEX-12ZZZ-0011-R7b-SM-5EGLE
Brain - Putamen (basal ganglia) Brain GTEX-13112-0011-R7b-SM-5DUVW
Brain - Putamen (basal ganglia) Brain GTEX-1313W-0011-R7b-SM-5DUWL
Brain - Putamen (basal ganglia) Brain GTEX-131YS-0011-R7a-SM-5IJDA
Brain - Putamen (basal ganglia) Brain GTEX-132Q8-0011-R7b-SM-5N9F1
Brain - Putamen (basal ganglia) Brain GTEX-139TS-0011-R7a-SM-5K7X7
Brain - Putamen (basal ganglia) Brain GTEX-139TT-0011-R7b-SM-5J2MN
Brain - Putamen (basal ganglia) Brain GTEX-13CF2-0011-R7b-SM-5K7UV
Brain - Putamen (basal ganglia) Brain GTEX-13CZV-0011-R7b-SM-5LZXH

Brain - Putamen (basal ganglia)   Brain   GTEX-13FHO-0011-R7b-SM-5LZXD
Brain - Putamen (basal ganglia)   Brain   GTEX-13FHP-0011-R7b-SM-5LZYI
Brain - Putamen (basal ganglia)   Brain   GTEX-13FLV-0011-R7b-SM-5K7WW
Brain - Putamen (basal ganglia)   Brain   GTEX-13FLW-0011-R7b-SM-5L3EZ
Brain - Putamen (basal ganglia)   Brain   GTEX-13FXS-0011-R7a-SM-5L3FC
Brain - Putamen (basal ganglia)   Brain   GTEX-13G51-0011-R7b-SM-5LZXG
Brain - Putamen (basal ganglia)   Brain   GTEX-13JUV-0011-R7b-SM-5LZZG
Brain - Putamen (basal ganglia)   Brain   GTEX-13JVG-0011-R7b-SM-5MR3Q
Brain - Putamen (basal ganglia)   Brain   GTEX-13N1W-0011-R7b-SM-5MR4F
Brain - Putamen (basal ganglia)   Brain   GTEX-13N2G-0011-R7a-SM-5MR42
Brain - Putamen (basal ganglia)   Brain   GTEX-13NYB-0011-R7a-SM-5MR5D
Brain - Putamen (basal ganglia)   Brain   GTEX-13O3O-0011-R7b-SM-5P9GZ
Brain - Putamen (basal ganglia)   Brain   GTEX-13O3Q-0011-R7a-SM-5KM38
Brain - Putamen (basal ganglia)   Brain   GTEX-13OVJ-0011-R7a-SM-5L3G1
Brain - Putamen (basal ganglia)   Brain   GTEX-13OVL-0011-R7a-SM-5P9GW
Brain - Putamen (basal ganglia)   Brain   GTEX-13OW5-0011-R7a-SM-5O9C9
Brain - Putamen (basal ganglia)   Brain   GTEX-13OW6-0011-R7b-SM-5L3H4
Brain - Putamen (basal ganglia)   Brain   GTEX-13OW8-0011-R7a-SM-5L3FP
Brain - Putamen (basal ganglia)   Brain   GTEX-13PVQ-0011-R7a-SM-5L3G3
Brain - Putamen (basal ganglia)   Brain   GTEX-13QJC-0011-R7b-SM-5PNUM
Brain - Putamen (basal ganglia)   Brain   GTEX-13RTJ-0011-R7b-SM-5P9JS
Brain - Putamen (basal ganglia)   Brain   GTEX-13S7M-0011-R7a-SM-5O9DK
Brain - Putamen (basal ganglia)   Brain   GTEX-13VXU-0011-R7b-SM-5SI72
Brain - Putamen (basal ganglia)   Brain   GTEX-13X6I-0011-R7b-SM-5PNWP
Brain - Putamen (basal ganglia)   Brain   GTEX-13X6J-0011-R7b-SM-5PNUC
Brain - Putamen (basal ganglia)   Brain   GTEX-13X6K-0011-R7b-SM-5P9K7
Brain - Putamen (basal ganglia)   Brain   GTEX-1445S-0011-R7b-SM-5PNUG

Brain - Putamen (basal ganglia)   Brain   GTEX-144GL-0011-R7b-SM-5P9HX
Brain - Putamen (basal ganglia)   Brain   GTEX-144GO-0011-R7b-SM-5S2TT
Brain - Putamen (basal ganglia)   Brain   GTEX-145LS-0011-R7a-SM-5PNWM
Brain - Putamen (basal ganglia)   Brain   GTEX-145MG-0011-R7b-SM-5P9JY
Brain - Putamen (basal ganglia)   Brain   GTEX-145MH-0011-R7a-SM-5PNWH
Brain - Putamen (basal ganglia)   Brain   GTEX-147GR-0011-R7a-SM-5S2RP
Brain - Putamen (basal ganglia)   Brain   GTEX-14ABY-0011-R7b-SM-5PNWS
Brain - Putamen (basal ganglia)   Brain   GTEX-14ASI-0011-R7b-SM-5YY9G
Brain - Putamen (basal ganglia)   Brain   GTEX-14BIN-0011-R7b-SM-5S2RI
Brain - Putamen (basal ganglia)   Brain   GTEX-14BMV-0011-R7b-SM-5YY9J
Brain - Putamen (basal ganglia)   Brain   GTEX-14C5O-0011-R7b-SM-5YYAH
Brain - Putamen (basal ganglia)   Brain   GTEX-14E7W-0011-R7b-SM-5ZZV4
Brain - Putamen (basal ganglia)   Brain   GTEX-14JG1-0011-R7b-SM-5YYAV
Brain - Putamen (basal ganglia)   Brain   GTEX-14LZ3-0011-R7a-SM-69LQN
Brain - Putamen (basal ganglia)   Brain   GTEX-14PJM-0011-R7a-SM-6AJBX
Brain - Putamen (basal ganglia)   Brain   GTEX-14PJO-0011-R7a-SM-664NR
Brain - Putamen (basal ganglia)   Brain   GTEX-14PN4-0011-R7b-SM-69LQQ
Brain - Putamen (basal ganglia)   Brain   GTEX-15CHQ-0011-R7b-SM-69LP1
Brain - Putamen (basal ganglia)   Brain   GTEX-15DCD-0011-R7b-SM-6LPID
Brain - Putamen (basal ganglia)   Brain   GTEX-15DYW-0011-R7a-SM-7KULG
Brain - Putamen (basal ganglia)   Brain   GTEX-15EO6-0011-R7a-SM-6M46Q
Brain - Putamen (basal ganglia)   Brain   GTEX-15ER7-0011-R7a-SM-6M46N
Brain - Putamen (basal ganglia)   Brain   GTEX-15G19-0011-R7b-SM-6M479
Brain - Putamen (basal ganglia)   Brain   GTEX-15RJE-0011-R7a-SM-7DHL9
Brain - Putamen (basal ganglia)   Brain   GTEX-16Z82-0011-R7a-SM-7MKGJ
Brain - Putamen (basal ganglia)   Brain   GTEX-17EVP-0011-R7a-SM-7LTAN
Brain - Putamen (basal ganglia)   Brain   GTEX-17EVQ-0011-R7a-SM-7EWDA

Brain - Putamen (basal ganglia)   Brain   GTEX-17HG3-0011-R7b-SM-7EPGR
Brain - Putamen (basal ganglia)   Brain   GTEX-17HHY-0011-R7a-SM-718A1
Brain - Putamen (basal ganglia)   Brain   GTEX-17HII-0011-R7a-SM-79OMS
Brain - Putamen (basal ganglia)   Brain   GTEX-17MF6-0011-R7b-SM-7LG6J
Brain - Putamen (basal ganglia)   Brain   GTEX-183WM-0011-R7b-SM-731AX
Brain - Putamen (basal ganglia)   Brain   GTEX-18464-0011-R7b-SM-731CM
Brain - Putamen (basal ganglia)   Brain   GTEX-18465-0011-R7b-SM-7LG6V
Brain - Putamen (basal ganglia)   Brain   GTEX-1A3MX-0011-R7a-SM-7DUEM
Brain - Putamen (basal ganglia)   Brain   GTEX-1B933-0011-R7a-SM-7DUGX
Brain - Putamen (basal ganglia)   Brain   GTEX-1B996-0011-R7b-SM-7IGNT
Brain - Putamen (basal ganglia)   Brain   GTEX-1C64N-0011-R7a-SM-7IGOI
Brain - Putamen (basal ganglia)   Brain   GTEX-N7MS-0011-R7a-SM-2HMKN
Brain - Putamen (basal ganglia)   Brain   GTEX-N7MT-0011-R7a-SM-2I3FZ
Brain - Putamen (basal ganglia)   Brain   GTEX-NL3H-0011-R7a-SM-2I3G5
Brain - Putamen (basal ganglia)   Brain   GTEX-NPJ8-0011-R7a-SM-2HMJV
Brain - Putamen (basal ganglia)   Brain   GTEX-OHPN-0011-R7A-SM-2I5FI
Brain - Putamen (basal ganglia)   Brain   GTEX-Q2AG-0011-R7A-SM-2HMJP
Brain - Putamen (basal ganglia)   Brain   GTEX-QDT8-0011-R7A-SM-32PKF
Brain - Putamen (basal ganglia)   Brain   GTEX-QMR6-0011-R7A-SM-32PKL
Brain - Putamen (basal ganglia)   Brain   GTEX-R55E-0011-R7A-SM-2TC5Z
Brain - Putamen (basal ganglia)   Brain   GTEX-RNOR-0011-R7A-SM-2TF4V
Brain - Putamen (basal ganglia)   Brain   GTEX-RU72-0011-R7A-SM-2TF5U
Brain - Putamen (basal ganglia)   Brain   GTEX-S7SE-0011-R7A-SM-2XCDI
Brain - Putamen (basal ganglia)   Brain   GTEX-T5JC-0011-R7A-SM-32PME
Brain - Putamen (basal ganglia)   Brain   GTEX-T6MN-0011-R7A-SM-32QP5
Brain - Putamen (basal ganglia)   Brain   GTEX-TSE9-0011-R7A-SM-3DB7P
Brain - Putamen (basal ganglia)   Brain   GTEX-WHSE-0011-R7A-SM-3P5YZ

Brain - Putamen (basal ganglia)   Brain   GTEX-WL46-0011-R7A-SM-3LK7X
Brain - Putamen (basal ganglia)   Brain   GTEX-WVLH-0011-R7A-SM-3MJFB
Brain - Putamen (basal ganglia)   Brain   GTEX-WWYW-0011-R7A-SM-3NB3H
Brain - Putamen (basal ganglia)   Brain   GTEX-WZTO-0011-R7B-SM-4E3IS
Brain - Putamen (basal ganglia)   Brain   GTEX-X261-0011-R7A-SM-4E3JJ
Brain - Putamen (basal ganglia)   Brain   GTEX-XLM4-0011-R7A-SM-4AT5L
Brain - Putamen (basal ganglia)   Brain   GTEX-Y8DK-0011-R7A-SM-4SOJZ
Brain - Putamen (basal ganglia)   Brain   GTEX-YFC4-0011-R7b-SM-4V6E2
Brain - Putamen (basal ganglia)   Brain   GTEX-YJ89-0011-R7a-SM-4V6GO
Brain - Putamen (basal ganglia)   Brain   GTEX-Z93S-0011-R7b-SM-4RGNK
Brain - Putamen (basal ganglia)   Brain   GTEX-ZAB4-0011-R7a-SM-4SOKE
Brain - Putamen (basal ganglia)   Brain   GTEX-ZDXO-0011-R7a-SM-4WWCU
Brain - Putamen (basal ganglia)   Brain   GTEX-ZE7O-0011-R7a-SM-57WCU
Brain - Putamen (basal ganglia)   Brain   GTEX-ZE9C-0011-R7a-SM-4WWCX
Brain - Putamen (basal ganglia)   Brain   GTEX-ZF28-0011-R7a-SM-4WKF5
Brain - Putamen (basal ganglia)   Brain   GTEX-ZUA1-0011-R7b-SM-4YCDP
Brain - Putamen (basal ganglia)   Brain   GTEX-ZV68-0011-R7a-SM-51MT4
Brain - Putamen (basal ganglia)   Brain   GTEX-ZVT3-0011-R7a-SM-57WCO
Brain - Putamen (basal ganglia)   Brain   GTEX-ZVZQ-0011-R7b-SM-57WBB
Brain - Putamen (basal ganglia)   Brain   GTEX-ZXG5-0011-R7b-SM-57WCC
Brain - Spinal cord (cervical c-1)   Brain   GTEX-11GSP-0011-R9b-SM-5NQ77
Brain - Spinal cord (cervical c-1)   Brain   GTEX-11ONC-0011-R9b-SM-5NQ8J
Brain - Spinal cord (cervical c-1)   Brain   GTEX-11WQK-0011-R9b-SM-5BC6N
Brain - Spinal cord (cervical c-1)   Brain   GTEX-11ZUS-0011-R9b-SM-5BC76
Brain - Spinal cord (cervical c-1)   Brain   GTEX-11ZVC-0011-R9a-SM-57WC5
Brain - Spinal cord (cervical c-1)   Brain   GTEX-12126-0011-R9b-SM-5BC6P
Brain - Spinal cord (cervical c-1)   Brain   GTEX-12WSC-0011-R9a-SM-5GU4U

Brain - Spinal cord (cervical c-1)	Brain	GTEX-12WSD-0011-R9a-SM-5GU6W
Brain - Spinal cord (cervical c-1)	Brain	GTEX-12WSH-0011-R9b-SM-5LZUG
Brain - Spinal cord (cervical c-1)	Brain	GTEX-131XW-0011-R9a-SM-5N9EO
Brain - Spinal cord (cervical c-1)	Brain	GTEX-131YS-0011-R9b-SM-5EQLY
Brain - Spinal cord (cervical c-1)	Brain	GTEX-139T4-0011-R9b-SM-5HL6S
Brain - Spinal cord (cervical c-1)	Brain	GTEX-139UW-0011-R9b-SM-5LZYT
Brain - Spinal cord (cervical c-1)	Brain	GTEX-13CIG-0011-R9a-SM-5K7VU
Brain - Spinal cord (cervical c-1)	Brain	GTEX-13CZV-0011-R9a-SM-5LZXT
Brain - Spinal cord (cervical c-1)	Brain	GTEX-13FLV-0011-R9b-SM-5L3DA
Brain - Spinal cord (cervical c-1)	Brain	GTEX-13FTY-0011-R9b-SM-5KM2U
Brain - Spinal cord (cervical c-1)	Brain	GTEX-13FXS-0011-R9a-SM-5K7UI
Brain - Spinal cord (cervical c-1)	Brain	GTEX-13JUV-0011-R9a-SM-5LZX3
Brain - Spinal cord (cervical c-1)	Brain	GTEX-13N1W-0011-R9b-SM-5MR4R
Brain - Spinal cord (cervical c-1)	Brain	GTEX-13N2G-0011-R9b-SM-5MR4E
Brain - Spinal cord (cervical c-1)	Brain	GTEX-13NYB-0011-R9b-SM-5MR5P
Brain - Spinal cord (cervical c-1)	Brain	GTEX-13NYS-0011-R9b-SM-5MR44
Brain - Spinal cord (cervical c-1)	Brain	GTEX-13NZA-0011-R9b-SM-5MR5I
Brain - Spinal cord (cervical c-1)	Brain	GTEX-13O1R-0011-R9b-SM-5KM33
Brain - Spinal cord (cervical c-1)	Brain	GTEX-13OVJ-0011-R9b-SM-5L3GD
Brain - Spinal cord (cervical c-1)	Brain	GTEX-13OVL-0011-R9a-SM-5P9GX
Brain - Spinal cord (cervical c-1)	Brain	GTEX-13OW5-0011-R9b-SM-5O9CL
Brain - Spinal cord (cervical c-1)	Brain	GTEX-13PL6-0011-R9b-SM-5O9CH
Brain - Spinal cord (cervical c-1)	Brain	GTEX-13PLJ-0011-R9b-SM-5O9DV
Brain - Spinal cord (cervical c-1)	Brain	GTEX-13PVQ-0011-R9b-SM-5LU3B
Brain - Spinal cord (cervical c-1)	Brain	GTEX-13QJC-0011-R9b-SM-5PNX3
Brain - Spinal cord (cervical c-1)	Brain	GTEX-13RTJ-0011-R9a-SM-5Q5AY
Brain - Spinal cord (cervical c-1)	Brain	GTEX-13S7M-0011-R9a-SM-5O9DW

Brain - Spinal cord (cervical c-1)	Brain	GTEX-13SLW-0011-
R9a-SM-5O9BT
Brain - Spinal cord (cervical c-1)	Brain	GTEX-13SLX-0011-
R9b-SM-5SI6X
Brain - Spinal cord (cervical c-1)	Brain	GTEX-13VXU-0011-
R9b-SM-5SI73
Brain - Spinal cord (cervical c-1)	Brain	GTEX-13X6I-0011-
R9b-SM-5PNZJ
Brain - Spinal cord (cervical c-1)	Brain	GTEX-145LU-0011-
R9b-SM-5SI66
Brain - Spinal cord (cervical c-1)	Brain	GTEX-147GR-0011-
R9a-SM-5S2UY
Brain - Spinal cord (cervical c-1)	Brain	GTEX-148VJ-0011-
R9b-SM-5SI77
Brain - Spinal cord (cervical c-1)	Brain	GTEX-14ABY-0011-
R9a-SM-5PNZK
Brain - Spinal cord (cervical c-1)	Brain	GTEX-14BIL-0011-
R9a-SM-5SI7A
Brain - Spinal cord (cervical c-1)	Brain	GTEX-14BIM-0011-
R9a-SM-6LLJL
Brain - Spinal cord (cervical c-1)	Brain	GTEX-14BIN-0011-
R9a-SM-5SI76
Brain - Spinal cord (cervical c-1)	Brain	GTEX-14BMV-0011-
R9b-SM-5ZZUA
Brain - Spinal cord (cervical c-1)	Brain	GTEX-14E6C-0011-
R9a-SM-6EU33
Brain - Spinal cord (cervical c-1)	Brain	GTEX-14E7W-0011-
R9a-SM-62LE6
Brain - Spinal cord (cervical c-1)	Brain	GTEX-14JIY-0011-
R9a-SM-6EU34
Brain - Spinal cord (cervical c-1)	Brain	GTEX-14PJM-0011-
R9b-SM-6EU35
Brain - Spinal cord (cervical c-1)	Brain	GTEX-14PJO-0011-
R9b-SM-62LE9
Brain - Spinal cord (cervical c-1)	Brain	GTEX-14PN4-0011-
R9a-SM-6LLH9
Brain - Spinal cord (cervical c-1)	Brain	GTEX-15CHQ-0011-
R9a-SM-6LLHX
Brain - Spinal cord (cervical c-1)	Brain	GTEX-15DCD-0011-
R9b-SM-7KUED
Brain - Spinal cord (cervical c-1)	Brain	GTEX-15DYW-0011-
R9b-SM-6PALB
Brain - Spinal cord (cervical c-1)	Brain	GTEX-15EO6-0011-
R9a-SM-6PALG
Brain - Spinal cord (cervical c-1)	Brain	GTEX-15ER7-0011-
R9b-SM-6PALF
Brain - Spinal cord (cervical c-1)	Brain	GTEX-15G19-0011-
R9b-SM-7KUEE
Brain - Spinal cord (cervical c-1)	Brain	GTEX-16NPV-0011-
R9b-SM-79ONF
Brain - Spinal cord (cervical c-1)	Brain	GTEX-16XZZ-0011-
R9b-SM-7DHKX
Brain - Spinal cord (cervical c-1)	Brain	GTEX-16Z82-0011-
R9a-SM-7DUFA

Brain - Spinal cord (cervical c-1)	Brain	GTEX-17EVQ-0011-R9b-SM-79OJY
Brain - Spinal cord (cervical c-1)	Brain	GTEX-18464-0011-R9a-SM-731BK
Brain - Spinal cord (cervical c-1)	Brain	GTEX-1A8G7-0011-R9b-SM-7IGQH
Brain - Spinal cord (cervical c-1)	Brain	GTEX-1B933-0011-R9a-SM-79OOR
Brain - Spinal cord (cervical c-1)	Brain	GTEX-1BAJH-0011-R9b-SM-7EPIQ
Brain - Spinal cord (cervical c-1)	Brain	GTEX-NL4W-0011-R9a-SM-2I3G1
Brain - Spinal cord (cervical c-1)	Brain	GTEX-NPJ7-0011-R9a-SM-2TC5R
Brain - Spinal cord (cervical c-1)	Brain	GTEX-NPJ8-0011-R9a-SM-2YUN5
Brain - Spinal cord (cervical c-1)	Brain	GTEX-OHPN-0011-R9A-SM-5TDEJ
Brain - Spinal cord (cervical c-1)	Brain	GTEX-OXRO-0011-R9A-SM-3NB1X
Brain - Spinal cord (cervical c-1)	Brain	GTEX-Q2AG-0011-R9A-SM-2HMJ6
Brain - Spinal cord (cervical c-1)	Brain	GTEX-QDT8-0011-R9A-SM-5SI8J
Brain - Spinal cord (cervical c-1)	Brain	GTEX-QVJO-0011-R9A-SM-2S1QH
Brain - Spinal cord (cervical c-1)	Brain	GTEX-QVUS-0011-R9A-SM-3GIJA
Brain - Spinal cord (cervical c-1)	Brain	GTEX-R55E-0011-R9A-SM-2TC6C
Brain - Spinal cord (cervical c-1)	Brain	GTEX-RNOR-0011-R9A-SM-2TF52
Brain - Spinal cord (cervical c-1)	Brain	GTEX-RU72-0011-R9A-SM-2TF67
Brain - Spinal cord (cervical c-1)	Brain	GTEX-T5JC-0011-R9A-SM-32PLV
Brain - Spinal cord (cervical c-1)	Brain	GTEX-T6MN-0011-R9A-SM-32QOZ
Brain - Spinal cord (cervical c-1)	Brain	GTEX-TSE9-0011-R9A-SM-3DB7Q
Brain - Spinal cord (cervical c-1)	Brain	GTEX-WL46-0011-R9A-SM-3MJFP
Brain - Spinal cord (cervical c-1)	Brain	GTEX-X4EP-0011-R9B-SM-4QASI
Brain - Spinal cord (cervical c-1)	Brain	GTEX-XLM4-0011-R9A-SM-4AT45
Brain - Spinal cord (cervical c-1)	Brain	GTEX-XMD1-0011-R9A-SM-4AT49
Brain - Spinal cord (cervical c-1)	Brain	GTEX-YFC4-0011-R9a-SM-4SOK4
Brain - Spinal cord (cervical c-1)	Brain	GTEX-YJ89-0011-R9a-SM-4SOK7
Brain - Spinal cord (cervical c-1)	Brain	GTEX-ZAB4-0011-R9a-SM-4SOKG

Brain - Spinal cord (cervical c-1)	Brain	GTEX-ZE9C-0011-R9a-SM-4WWCY
Brain - Spinal cord (cervical c-1)	Brain	GTEX-ZF28-0011-R9a-SM-4WWDY
Brain - Spinal cord (cervical c-1)	Brain	GTEX-ZVZQ-0011-R9a-SM-51MRH
Brain - Substantia nigra	Brain	GTEX-11GSO-0011-R2a-SM-57WDF
Brain - Substantia nigra	Brain	GTEX-11PRG-0011-R2a-SM-69LQ5
Brain - Substantia nigra	Brain	GTEX-11UD1-0011-R2b-SM-5BC6O
Brain - Substantia nigra	Brain	GTEX-11ZTS-0011-R2b-SM-5BC75
Brain - Substantia nigra	Brain	GTEX-12WSA-0011-R2a-SM-57WDK
Brain - Substantia nigra	Brain	GTEX-12WSD-0011-R2a-SM-5LZWB
Brain - Substantia nigra	Brain	GTEX-12WSF-0011-R2a-SM-5DUVU
Brain - Substantia nigra	Brain	GTEX-12WSH-0011-R2a-SM-5GU68
Brain - Substantia nigra	Brain	GTEX-12ZZX-0011-R2a-SM-5EGLG
Brain - Substantia nigra	Brain	GTEX-1313W-0011-R2a-SM-5EGLF
Brain - Substantia nigra	Brain	GTEX-131XH-0011-R2b-SM-5DUVY
Brain - Substantia nigra	Brain	GTEX-132Q8-0011-R2b-SM-5EQ5Z
Brain - Substantia nigra	Brain	GTEX-1399T-0011-R2b-SM-5DUXM
Brain - Substantia nigra	Brain	GTEX-139TS-0011-R2b-SM-5J1MX
Brain - Substantia nigra	Brain	GTEX-13CF2-0011-R2a-SM-5L3DC
Brain - Substantia nigra	Brain	GTEX-13FXS-0011-R2b-SM-5K7XX
Brain - Substantia nigra	Brain	GTEX-13G51-0011-R2b-SM-5LZXS
Brain - Substantia nigra	Brain	GTEX-13N2G-0011-R2a-SM-5MR4Q
Brain - Substantia nigra	Brain	GTEX-13NYB-0011-R2a-SM-5MR3C
Brain - Substantia nigra	Brain	GTEX-13NYS-0011-R2b-SM-5MR4G
Brain - Substantia nigra	Brain	GTEX-13O3O-0011-R2b-SM-5P9H1
Brain - Substantia nigra	Brain	GTEX-13O3Q-0011-R2b-SM-5KM3K
Brain - Substantia nigra	Brain	GTEX-13OVJ-0011-R2b-SM-5L3GP
Brain - Substantia nigra	Brain	GTEX-13OW6-0011-R2a-SM-5L3HG
Brain - Substantia nigra	Brain	GTEX-13OW8-0011-R2a-SM-5L3G2
Brain - Substantia nigra	Brain	GTEX-13PDP-0011-R2b-SM-5O9DH
Brain - Substantia nigra	Brain	GTEX-13QJC-0011-R2b-SM-5PNUN
Brain - Substantia nigra	Brain	GTEX-13RTJ-0011-R2a-SM-5PNW9
Brain - Substantia nigra	Brain	GTEX-13SLW-0011-R2b-SM-5O9C6
Brain - Substantia nigra	Brain	GTEX-13SLX-0011-R2b-SM-5S2VP
Brain - Substantia nigra	Brain	GTEX-13X6I-0011-R2b-SM-5PNWQ
Brain - Substantia nigra	Brain	GTEX-13X6K-0011-R2b-SM-5P9K3
Brain - Substantia nigra	Brain	GTEX-1445S-0011-R2b-SM-5PNUH
Brain - Substantia nigra	Brain	GTEX-145LS-0011-R2a-SM-5PNZI
Brain - Substantia nigra	Brain	GTEX-147GR-0011-R2b-SM-5S2RQ
Brain - Substantia nigra	Brain	GTEX-14ASI-0011-R2b-SM-5YY9H
Brain - Substantia nigra	Brain	GTEX-14BIM-0011-R2b-SM-69LQ6
Brain - Substantia nigra	Brain	GTEX-14BIN-0011-R2a-SM-5S2RJ
Brain - Substantia nigra	Brain	GTEX-14C5O-0011-R2a-SM-5ZZV2
Brain - Substantia nigra	Brain	GTEX-14JG1-0011-R2a-SM-5ZZVE
Brain - Substantia nigra	Brain	GTEX-14PJO-0011-R2a-SM-5ZZVF
Brain - Substantia nigra	Brain	GTEX-14PN4-0011-R2a-SM-6EU2Y
Brain - Substantia nigra	Brain	GTEX-15CHQ-0011-R2b-SM-6AJAD
Brain - Substantia nigra	Brain	GTEX-15DCD-0011-R2b-SM-6LPIE
Brain - Substantia nigra	Brain	GTEX-15DYW-0011-R2b-SM-7KUKY
Brain - Substantia nigra	Brain	GTEX-15EO6-0011-R2a-SM-6M46R
Brain - Substantia nigra	Brain	GTEX-15ER7-0011-R2a-SM-6M46O
Brain - Substantia nigra	Brain	GTEX-15RJE-0011-R2b-SM-79OMP

Brain - Substantia nigra	Brain	GTEX-16Z82-0011-R2b-SM-79OK9
Brain - Substantia nigra	Brain	GTEX-17EVP-0011-R2a-SM-7LG4W
Brain - Substantia nigra	Brain	GTEX-18464-0011-R2a-SM-731AL
Brain - Substantia nigra	Brain	GTEX-1A3MX-0011-R2b-SM-7EWFL
Brain - Substantia nigra	Brain	GTEX-1A8G7-0011-R2a-SM-7IGQI
Brain - Substantia nigra	Brain	GTEX-1B8SG-0011-R2b-SM-79OOT
Brain - Substantia nigra	Brain	GTEX-1B933-0011-R2b-SM-7IGNM
Brain - Substantia nigra	Brain	GTEX-1B996-0011-R2a-SM-7IGON
Brain - Substantia nigra	Brain	GTEX-N7MS-0011-R2a-SM-2HML6
Brain - Substantia nigra	Brain	GTEX-N7MT-0011-R2a-SM-2I3GI
Brain - Substantia nigra	Brain	GTEX-NL4W-0011-R2a-SM-2I5GV
Brain - Substantia nigra	Brain	GTEX-NPJ7-0011-R2a-SM-2I3GF
Brain - Substantia nigra	Brain	GTEX-NPJ8-0011-R2a-SM-2TC6M
Brain - Substantia nigra	Brain	GTEX-OHPN-0011-R2A-SM-2I5FB
Brain - Substantia nigra	Brain	GTEX-OXRO-0011-R2A-SM-3NB1W
Brain - Substantia nigra	Brain	GTEX-PWO3-0011-R2A-SM-2S1OX
Brain - Substantia nigra	Brain	GTEX-Q2AG-0011-R2A-SM-2HMIT
Brain - Substantia nigra	Brain	GTEX-QDT8-0011-R2A-SM-32PKQ
Brain - Substantia nigra	Brain	GTEX-QMR6-0011-R2A-SM-32PKV
Brain - Substantia nigra	Brain	GTEX-QVJO-0011-R2A-SM-2S1QK
Brain - Substantia nigra	Brain	GTEX-RU72-0011-R2A-SM-2TF6O
Brain - Substantia nigra	Brain	GTEX-S7SE-0011-R2A-SM-2XCDC
Brain - Substantia nigra	Brain	GTEX-T2IS-0011-R2A-SM-32QPF
Brain - Substantia nigra	Brain	GTEX-T5JC-0011-R2A-SM-32PLZ
Brain - Substantia nigra	Brain	GTEX-T6MN-0011-R2A-SM-32QOW
Brain - Substantia nigra	Brain	GTEX-UTHO-0011-R2A-SM-3GIKC
Brain - Substantia nigra	Brain	GTEX-WHSE-0011-R2A-SM-3P5ZL
Brain - Substantia nigra	Brain	GTEX-WL46-0011-R2A-SM-3LK6O
Brain - Substantia nigra	Brain	GTEX-WVLH-0011-R2A-SM-3MJFJ
Brain - Substantia nigra	Brain	GTEX-X4XX-0011-R2A-SM-3P623
Brain - Substantia nigra	Brain	GTEX-X585-0011-R2B-SM-46MVF
Brain - Substantia nigra	Brain	GTEX-XLM4-0011-R2B-SM-4AT5Z
Brain - Substantia nigra	Brain	GTEX-XMD1-0011-R2B-SM-4AT5N
Brain - Substantia nigra	Brain	GTEX-YFC4-0011-R2a-SM-4V6DZ
Brain - Substantia nigra	Brain	GTEX-YJ89-0011-R2b-SM-4RGLT
Brain - Substantia nigra	Brain	GTEX-Z93S-0011-R2a-SM-4RGNG
Brain - Substantia nigra	Brain	GTEX-ZAB4-0011-R2a-SM-4RGNP
Brain - Substantia nigra	Brain	GTEX-ZDXO-0011-R2b-SM-4WKFG
Brain - Substantia nigra	Brain	GTEX-ZE9C-0011-R2a-SM-4WKGH
Brain - Substantia nigra	Brain	GTEX-ZV68-0011-R2a-SM-4YCDK
Breast - Mammary Tissue	Breast	GTEX-1117F-2826-SM-5GZXL
Breast - Mammary Tissue	Breast	GTEX-111YS-1926-SM-5GICC
Breast - Mammary Tissue	Breast	GTEX-1122O-1226-SM-5H113
Breast - Mammary Tissue	Breast	GTEX-117XS-1926-SM-5GICO
Breast - Mammary Tissue	Breast	GTEX-117YX-1426-SM-5H12H
Breast - Mammary Tissue	Breast	GTEX-1192X-2326-SM-5987X
Breast - Mammary Tissue	Breast	GTEX-11DXW-0626-SM-5N9ER
Breast - Mammary Tissue	Breast	GTEX-11DXY-2326-SM-5GICW
Breast - Mammary Tissue	Breast	GTEX-11DXZ-1926-SM-5GZZL
Breast - Mammary Tissue	Breast	GTEX-11DZ1-0326-SM-5N9BN
Breast - Mammary Tissue	Breast	GTEX-11EI6-0626-SM-5985T
Breast - Mammary Tissue	Breast	GTEX-11EM3-1326-SM-5N9C6
Breast - Mammary Tissue	Breast	GTEX-11EMC-2026-SM-5A5JV
Breast - Mammary Tissue	Breast	GTEX-11EQ9-1826-SM-5Q5AJ

Breast - Mammary Tissue	Breast	GTEX-11GS4-2126-SM-5A5KR
Breast - Mammary Tissue	Breast	GTEX-11GSO-1926-SM-5A5K3
Breast - Mammary Tissue	Breast	GTEX-11I78-2226-SM-5PNYA
Breast - Mammary Tissue	Breast	GTEX-11LCK-2426-SM-5HL5F
Breast - Mammary Tissue	Breast	GTEX-11NSD-0926-SM-5N9DR
Breast - Mammary Tissue	Breast	GTEX-11NV4-2026-SM-5N9DG
Breast - Mammary Tissue	Breast	GTEX-11O72-2126-SM-5N9FO
Breast - Mammary Tissue	Breast	GTEX-11OF3-1926-SM-59889
Breast - Mammary Tissue	Breast	GTEX-11ONC-2126-SM-5HL6E
Breast - Mammary Tissue	Breast	GTEX-11P7K-0726-SM-5EGKX
Breast - Mammary Tissue	Breast	GTEX-11P81-1926-SM-5BC53
Breast - Mammary Tissue	Breast	GTEX-11P82-1326-SM-5HL62
Breast - Mammary Tissue	Breast	GTEX-11PRG-0826-SM-5EQ6A
Breast - Mammary Tissue	Breast	GTEX-11TT1-2126-SM-5GU5Y
Breast - Mammary Tissue	Breast	GTEX-11TUW-1826-SM-5BC5D
Breast - Mammary Tissue	Breast	GTEX-11WQC-1726-SM-5GU4W
Breast - Mammary Tissue	Breast	GTEX-11WQK-2426-SM-5GU5C
Breast - Mammary Tissue	Breast	GTEX-11ZTT-2326-SM-5EQLG
Breast - Mammary Tissue	Breast	GTEX-11ZUS-0826-SM-5FQUY
Breast - Mammary Tissue	Breast	GTEX-1211K-1926-SM-5EQLB
Breast - Mammary Tissue	Breast	GTEX-1269C-2426-SM-5FQSN
Breast - Mammary Tissue	Breast	GTEX-12BJ1-1826-SM-5HL9N
Breast - Mammary Tissue	Breast	GTEX-12KS4-0126-SM-5Q5A5
Breast - Mammary Tissue	Breast	GTEX-12WSK-2226-SM-5GCO5
Breast - Mammary Tissue	Breast	GTEX-12WSM-1726-SM-5BC6J
Breast - Mammary Tissue	Breast	GTEX-12WSN-1326-SM-5GCNT
Breast - Mammary Tissue	Breast	GTEX-12ZZX-1126-SM-5EGKB
Breast - Mammary Tissue	Breast	GTEX-13113-1726-SM-5GCOO
Breast - Mammary Tissue	Breast	GTEX-1313W-0826-SM-5EQ4T
Breast - Mammary Tissue	Breast	GTEX-1314G-1226-SM-5BC6D
Breast - Mammary Tissue	Breast	GTEX-131XW-0726-SM-5EGK3
Breast - Mammary Tissue	Breast	GTEX-131YS-0626-SM-5EGKL
Breast - Mammary Tissue	Breast	GTEX-132AR-0826-SM-5EGK6
Breast - Mammary Tissue	Breast	GTEX-132NY-0826-SM-5K7Y7
Breast - Mammary Tissue	Breast	GTEX-133LE-1726-SM-5K7VQ
Breast - Mammary Tissue	Breast	GTEX-1399U-1826-SM-5PNZ1
Breast - Mammary Tissue	Breast	GTEX-139T6-1626-SM-5PNYZ
Breast - Mammary Tissue	Breast	GTEX-139T8-0826-SM-5L3DE
Breast - Mammary Tissue	Breast	GTEX-139TU-0626-SM-5KM3X
Breast - Mammary Tissue	Breast	GTEX-13CF2-2026-SM-5K7VI
Breast - Mammary Tissue	Breast	GTEX-13CF3-2126-SM-5IFJP
Breast - Mammary Tissue	Breast	GTEX-13D11-1026-SM-5IJFB
Breast - Mammary Tissue	Breast	GTEX-13FHO-0826-SM-5L3E8
Breast - Mammary Tissue	Breast	GTEX-13FTW-1426-SM-5LZWZ
Breast - Mammary Tissue	Breast	GTEX-13FTX-1126-SM-5N9EN
Breast - Mammary Tissue	Breast	GTEX-13FTY-2226-SM-5J1ND
Breast - Mammary Tissue	Breast	GTEX-13N11-1726-SM-5J1OJ
Breast - Mammary Tissue	Breast	GTEX-13N1W-0626-SM-5MR4U
Breast - Mammary Tissue	Breast	GTEX-13NZ8-0126-SM-5IJCT
Breast - Mammary Tissue	Breast	GTEX-13NZ9-1026-SM-5MR5K
Breast - Mammary Tissue	Breast	GTEX-13NZB-2126-SM-5MR4Y
Breast - Mammary Tissue	Breast	GTEX-13O3O-0826-SM-5K7WE
Breast - Mammary Tissue	Breast	GTEX-13O3P-0826-SM-5L3DH
Breast - Mammary Tissue	Breast	GTEX-13O3Q-2226-SM-5KM4O

Breast - Mammary Tissue	Breast	GTEX-13O61-1826-SM-5KM4I
Breast - Mammary Tissue	Breast	GTEX-13OW5-2226-SM-5L3HC
Breast - Mammary Tissue	Breast	GTEX-13OW8-0226-SM-5K7UP
Breast - Mammary Tissue	Breast	GTEX-13PL6-2926-SM-5L3I2
Breast - Mammary Tissue	Breast	GTEX-13PVQ-1026-SM-5KM3M
Breast - Mammary Tissue	Breast	GTEX-13PVR-2226-SM-7DHKP
Breast - Mammary Tissue	Breast	GTEX-13QIC-2326-SM-5LU5N
Breast - Mammary Tissue	Breast	GTEX-13QJ3-0826-SM-7DHKK
Breast - Mammary Tissue	Breast	GTEX-13SLW-2526-SM-62LDQ
Breast - Mammary Tissue	Breast	GTEX-13SLX-2326-SM-5ZZWE
Breast - Mammary Tissue	Breast	GTEX-13VXU-2826-SM-664MA
Breast - Mammary Tissue	Breast	GTEX-13W3W-1226-SM-5LU4H
Breast - Mammary Tissue	Breast	GTEX-13W46-0826-SM-5LU3H
Breast - Mammary Tissue	Breast	GTEX-144GL-2026-SM-5LU3O
Breast - Mammary Tissue	Breast	GTEX-144GM-0926-SM-5O994
Breast - Mammary Tissue	Breast	GTEX-145LT-0726-SM-5S2VM
Breast - Mammary Tissue	Breast	GTEX-145ME-1526-SM-5Q5F2
Breast - Mammary Tissue	Breast	GTEX-145MF-2226-SM-7EPIR
Breast - Mammary Tissue	Breast	GTEX-145MN-1926-SM-5SIAI
Breast - Mammary Tissue	Breast	GTEX-145MO-0826-SM-5NQBL
Breast - Mammary Tissue	Breast	GTEX-146FH-0826-SM-5SI8T
Breast - Mammary Tissue	Breast	GTEX-14753-2426-SM-5LU8U
Breast - Mammary Tissue	Breast	GTEX-147F4-2826-SM-5NQBN
Breast - Mammary Tissue	Breast	GTEX-14A5I-0726-SM-5TDEB
Breast - Mammary Tissue	Breast	GTEX-14AS3-1626-SM-5S2OY
Breast - Mammary Tissue	Breast	GTEX-14B4R-1226-SM-5TDDT
Breast - Mammary Tissue	Breast	GTEX-14BMU-1626-SM-5TDE7
Breast - Mammary Tissue	Breast	GTEX-14BMV-0626-SM-793AU
Breast - Mammary Tissue	Breast	GTEX-14DAR-1326-SM-7DUEG
Breast - Mammary Tissue	Breast	GTEX-14E6C-1326-SM-62LEQ
Breast - Mammary Tissue	Breast	GTEX-14E6E-1326-SM-5S2NR
Breast - Mammary Tissue	Breast	GTEX-14E7W-0826-SM-62LEJ
Breast - Mammary Tissue	Breast	GTEX-14H4A-2526-SM-5YYAY
Breast - Mammary Tissue	Breast	GTEX-14ICK-2426-SM-6EU27
Breast - Mammary Tissue	Breast	GTEX-14LLW-0626-SM-62LFC
Breast - Mammary Tissue	Breast	GTEX-14PHY-1926-SM-5YY95
Breast - Mammary Tissue	Breast	GTEX-14PJ4-2126-SM-6ETZJ
Breast - Mammary Tissue	Breast	GTEX-14PJO-0726-SM-69LO8
Breast - Mammary Tissue	Breast	GTEX-14PKU-0426-SM-6EU1P
Breast - Mammary Tissue	Breast	GTEX-14PN4-0626-SM-62LFP
Breast - Mammary Tissue	Breast	GTEX-15DCZ-0726-SM-69LOV
Breast - Mammary Tissue	Breast	GTEX-15EOM-5019-SM-793DK
Breast - Mammary Tissue	Breast	GTEX-15ER7-1626-SM-6PAMZ
Breast - Mammary Tissue	Breast	GTEX-15ETS-0626-SM-7KUMX
Breast - Mammary Tissue	Breast	GTEX-15FZZ-0726-SM-7KUFZ
Breast - Mammary Tissue	Breast	GTEX-15G19-2126-SM-6M48J
Breast - Mammary Tissue	Breast	GTEX-15RJE-2626-SM-7KFT1
Breast - Mammary Tissue	Breast	GTEX-15SHW-1326-SM-6PAL8
Breast - Mammary Tissue	Breast	GTEX-15UF6-0126-SM-6PAMB
Breast - Mammary Tissue	Breast	GTEX-15UF7-0726-SM-6M46D
Breast - Mammary Tissue	Breast	GTEX-16BQI-1026-SM-7KUEA
Breast - Mammary Tissue	Breast	GTEX-16NGA-0826-SM-718AF
Breast - Mammary Tissue	Breast	GTEX-16YQH-2826-SM-6PAMY
Breast - Mammary Tissue	Breast	GTEX-17EUY-1926-SM-7DUF6

Breast - Mammary Tissue	Breast	GTEX-17EVP-0226-SM-79OND
Breast - Mammary Tissue	Breast	GTEX-17EVQ-0426-SM-7LG57
Breast - Mammary Tissue	Breast	GTEX-17F96-2426-SM-7IGLN
Breast - Mammary Tissue	Breast	GTEX-17F97-2526-SM-7EWDV
Breast - Mammary Tissue	Breast	GTEX-17F98-0526-SM-79OK5
Breast - Mammary Tissue	Breast	GTEX-17GQL-0326-SM-7LG5U
Breast - Mammary Tissue	Breast	GTEX-17HG3-0126-SM-7IGNH
Breast - Mammary Tissue	Breast	GTEX-17HGU-1326-SM-79OKB
Breast - Mammary Tissue	Breast	GTEX-17HHE-1426-SM-7EPH4
Breast - Mammary Tissue	Breast	GTEX-17HHY-0926-SM-793C1
Breast - Mammary Tissue	Breast	GTEX-17JCI-0726-SM-7EPH1
Breast - Mammary Tissue	Breast	GTEX-17KNJ-2026-SM-7LG53
Breast - Mammary Tissue	Breast	GTEX-17MF6-0326-SM-7EPH5
Breast - Mammary Tissue	Breast	GTEX-17MFQ-0926-SM-7LG4S
Breast - Mammary Tissue	Breast	GTEX-183FY-1126-SM-7DHLJ
Breast - Mammary Tissue	Breast	GTEX-183WM-0726-SM-7LTAA
Breast - Mammary Tissue	Breast	GTEX-18465-2026-SM-718AP
Breast - Mammary Tissue	Breast	GTEX-18A6Q-0926-SM-7LG4N
Breast - Mammary Tissue	Breast	GTEX-18A7A-0726-SM-7LTAI
Breast - Mammary Tissue	Breast	GTEX-18A7B-2626-SM-7LG55
Breast - Mammary Tissue	Breast	GTEX-18D9A-1526-SM-7LG4J
Breast - Mammary Tissue	Breast	GTEX-18QFQ-0826-SM-718AX
Breast - Mammary Tissue	Breast	GTEX-1A3MV-1626-SM-731C1
Breast - Mammary Tissue	Breast	GTEX-1A3MX-2726-SM-718B6
Breast - Mammary Tissue	Breast	GTEX-1A8G7-2426-SM-731AK
Breast - Mammary Tissue	Breast	GTEX-1AMEY-1026-SM-718AA
Breast - Mammary Tissue	Breast	GTEX-1AX8Z-0926-SM-731AW
Breast - Mammary Tissue	Breast	GTEX-1AX9I-0726-SM-73KWV
Breast - Mammary Tissue	Breast	GTEX-1AX9J-1126-SM-731B7
Breast - Mammary Tissue	Breast	GTEX-1B8KE-1226-SM-73KWK
Breast - Mammary Tissue	Breast	GTEX-1B8KZ-1526-SM-7DUG7
Breast - Mammary Tissue	Breast	GTEX-1B932-0826-SM-73KXG
Breast - Mammary Tissue	Breast	GTEX-1B933-2526-SM-7IGO5
Breast - Mammary Tissue	Breast	GTEX-1B97I-0426-SM-79OL7
Breast - Mammary Tissue	Breast	GTEX-1B97J-0426-SM-79OLQ
Breast - Mammary Tissue	Breast	GTEX-1BAJH-0826-SM-7EWEF
Breast - Mammary Tissue	Breast	GTEX-1C64O-0726-SM-7DUFU
Breast - Mammary Tissue	Breast	GTEX-1C6VQ-0426-SM-79OOX
Breast - Mammary Tissue	Breast	GTEX-1C6VS-0726-SM-7EPHF
Breast - Mammary Tissue	Breast	GTEX-1CAMR-1426-SM-793BO
Breast - Mammary Tissue	Breast	GTEX-1CAMS-1426-SM-7IGPM
Breast - Mammary Tissue	Breast	GTEX-1CB4E-0226-SM-79OLW
Breast - Mammary Tissue	Breast	GTEX-1CB4G-2326-SM-79OOI
Breast - Mammary Tissue	Breast	GTEX-1CB4J-1826-SM-7EWF9
Breast - Mammary Tissue	Breast	GTEX-1E2YA-2726-SM-7IGPW
Breast - Mammary Tissue	Breast	GTEX-1EKGG-2626-SM-7IGPY
Breast - Mammary Tissue	Breast	GTEX-1EU9M-2826-SM-7EWFH
Breast - Mammary Tissue	Breast	GTEX-PSDG-1626-SM-48TCQ
Breast - Mammary Tissue	Breast	GTEX-Q2AG-0326-SM-48U1O
Breast - Mammary Tissue	Breast	GTEX-QDT8-0626-SM-48TYW
Breast - Mammary Tissue	Breast	GTEX-QEG5-0726-SM-4R1JQ
Breast - Mammary Tissue	Breast	GTEX-QEL4-2126-SM-447AE
Breast - Mammary Tissue	Breast	GTEX-QMRM-1626-SM-4R1KV
Breast - Mammary Tissue	Breast	GTEX-QVJO-1826-SM-447C9

Breast - Mammary Tissue	Breast	GTEX-R3RS-0626-SM-48FE1
Breast - Mammary Tissue	Breast	GTEX-R53T-1526-SM-48FEK
Breast - Mammary Tissue	Breast	GTEX-R55D-0826-SM-48FEA
Breast - Mammary Tissue	Breast	GTEX-REY6-2426-SM-48FF5
Breast - Mammary Tissue	Breast	GTEX-RU1J-0626-SM-4WAWY
Breast - Mammary Tissue	Breast	GTEX-RU72-0626-SM-46MUI
Breast - Mammary Tissue	Breast	GTEX-RUSQ-2026-SM-4GIAK
Breast - Mammary Tissue	Breast	GTEX-RWS6-1926-SM-47JXY
Breast - Mammary Tissue	Breast	GTEX-S32W-2026-SM-4AD6E
Breast - Mammary Tissue	Breast	GTEX-S33H-0326-SM-4AD6N
Breast - Mammary Tissue	Breast	GTEX-S341-1526-SM-4AD6K
Breast - Mammary Tissue	Breast	GTEX-S4P3-1326-SM-4AD6V
Breast - Mammary Tissue	Breast	GTEX-S4Q7-1126-SM-4AD6R
Breast - Mammary Tissue	Breast	GTEX-S4UY-0726-SM-4AD6X
Breast - Mammary Tissue	Breast	GTEX-S7SE-0826-SM-4AT4D
Breast - Mammary Tissue	Breast	GTEX-SE5C-2126-SM-4BRUJ
Breast - Mammary Tissue	Breast	GTEX-T2IS-1526-SM-32QPR
Breast - Mammary Tissue	Breast	GTEX-T2YK-2226-SM-32QPT
Breast - Mammary Tissue	Breast	GTEX-T5JC-2126-SM-32PMO
Breast - Mammary Tissue	Breast	GTEX-T5JW-2026-SM-4DM63
Breast - Mammary Tissue	Breast	GTEX-T6MN-0726-SM-32PML
Breast - Mammary Tissue	Breast	GTEX-T6MO-0326-SM-32QOK
Breast - Mammary Tissue	Breast	GTEX-TKQ1-0226-SM-33HB5
Breast - Mammary Tissue	Breast	GTEX-TKQ2-1826-SM-33HB2
Breast - Mammary Tissue	Breast	GTEX-TML8-1226-SM-32QON
Breast - Mammary Tissue	Breast	GTEX-TMMY-0726-SM-33HBE
Breast - Mammary Tissue	Breast	GTEX-U3ZH-1426-SM-4DXSR
Breast - Mammary Tissue	Breast	GTEX-U3ZN-1926-SM-4DXSG
Breast - Mammary Tissue	Breast	GTEX-U412-1826-SM-4DXTJ
Breast - Mammary Tissue	Breast	GTEX-U8XE-0826-SM-4E3J1
Breast - Mammary Tissue	Breast	GTEX-UJHI-1426-SM-3DB9C
Breast - Mammary Tissue	Breast	GTEX-UPK5-2326-SM-3P5Z8
Breast - Mammary Tissue	Breast	GTEX-UTHO-1026-SM-3GAF7
Breast - Mammary Tissue	Breast	GTEX-V955-2026-SM-3GAFA
Breast - Mammary Tissue	Breast	GTEX-VJWN-0726-SM-3GIJ8
Breast - Mammary Tissue	Breast	GTEX-VUSG-2226-SM-4KKZO
Breast - Mammary Tissue	Breast	GTEX-W5X1-2326-SM-3GIL6
Breast - Mammary Tissue	Breast	GTEX-WFON-1826-SM-3GILG
Breast - Mammary Tissue	Breast	GTEX-WI4N-1426-SM-3LK7H
Breast - Mammary Tissue	Breast	GTEX-WOFL-0826-SM-3MJG1
Breast - Mammary Tissue	Breast	GTEX-WRHU-0326-SM-3MJFY
Breast - Mammary Tissue	Breast	GTEX-WXYG-2226-SM-4E3IM
Breast - Mammary Tissue	Breast	GTEX-WY7C-2726-SM-3NB3P
Breast - Mammary Tissue	Breast	GTEX-WYBS-0926-SM-3NM94
Breast - Mammary Tissue	Breast	GTEX-WYJK-1326-SM-3NB2T
Breast - Mammary Tissue	Breast	GTEX-WYVS-1726-SM-3NMAY
Breast - Mammary Tissue	Breast	GTEX-X15G-1626-SM-3NMB3
Breast - Mammary Tissue	Breast	GTEX-X261-0626-SM-3NMD9
Breast - Mammary Tissue	Breast	GTEX-X4EP-2926-SM-3P5YQ
Breast - Mammary Tissue	Breast	GTEX-X4XY-0926-SM-4E3JD
Breast - Mammary Tissue	Breast	GTEX-XBED-1626-SM-47JYN
Breast - Mammary Tissue	Breast	GTEX-XGQ4-0926-SM-4AT4U
Breast - Mammary Tissue	Breast	GTEX-XMD1-0826-SM-4AT52
Breast - Mammary Tissue	Breast	GTEX-XMD2-0926-SM-4WWEF

Breast - Mammary Tissue	Breast	GTEX-XMK1-1126-SM-4IHJ8
Breast - Mammary Tissue	Breast	GTEX-XOT4-0726-SM-4GIAW
Breast - Mammary Tissue	Breast	GTEX-XQ3S-1326-SM-4BOPQ
Breast - Mammary Tissue	Breast	GTEX-XQ8I-2426-SM-4WAXY
Breast - Mammary Tissue	Breast	GTEX-XUW1-2326-SM-4BOO5
Breast - Mammary Tissue	Breast	GTEX-XUZC-1626-SM-4BRVP
Breast - Mammary Tissue	Breast	GTEX-XV7Q-2326-SM-4BRVZ
Breast - Mammary Tissue	Breast	GTEX-XYKS-1326-SM-4BRUN
Breast - Mammary Tissue	Breast	GTEX-Y111-2026-SM-4SOJA
Breast - Mammary Tissue	Breast	GTEX-Y114-2026-SM-4TT7L
Breast - Mammary Tissue	Breast	GTEX-Y3I4-1526-SM-4TT7K
Breast - Mammary Tissue	Breast	GTEX-Y3IK-2326-SM-4WWDT
Breast - Mammary Tissue	Breast	GTEX-Y5LM-1726-SM-4VDSX
Breast - Mammary Tissue	Breast	GTEX-Y5V5-2126-SM-4WWFO
Breast - Mammary Tissue	Breast	GTEX-Y5V6-2126-SM-4WWFX
Breast - Mammary Tissue	Breast	GTEX-Y8E4-1626-SM-5S2MW
Breast - Mammary Tissue	Breast	GTEX-Y8LW-1626-SM-5IFHX
Breast - Mammary Tissue	Breast	GTEX-Y9LG-1426-SM-5IFJZ
Breast - Mammary Tissue	Breast	GTEX-YB5E-1726-SM-5IFJ3
Breast - Mammary Tissue	Breast	GTEX-YB5K-1626-SM-5IFIN
Breast - Mammary Tissue	Breast	GTEX-YEC3-1026-SM-5IFI5
Breast - Mammary Tissue	Breast	GTEX-YFC4-1426-SM-5IFJG
Breast - Mammary Tissue	Breast	GTEX-YFCO-1826-SM-4W1YH
Breast - Mammary Tissue	Breast	GTEX-YJ8O-2226-SM-5IFHW
Breast - Mammary Tissue	Breast	GTEX-ZA64-1526-SM-5CVMD
Breast - Mammary Tissue	Breast	GTEX-ZAB4-2526-SM-5HL8M
Breast - Mammary Tissue	Breast	GTEX-ZAJG-0626-SM-5HL8X
Breast - Mammary Tissue	Breast	GTEX-ZC5H-2626-SM-5J2MG
Breast - Mammary Tissue	Breast	GTEX-ZDTT-2126-SM-5S2OJ
Breast - Mammary Tissue	Breast	GTEX-ZDXO-0126-SM-5S2ND
Breast - Mammary Tissue	Breast	GTEX-ZDYS-1126-SM-5K7UB
Breast - Mammary Tissue	Breast	GTEX-ZEX8-2226-SM-57WC6
Breast - Mammary Tissue	Breast	GTEX-ZF29-1926-SM-5S2P1
Breast - Mammary Tissue	Breast	GTEX-ZF2S-2026-SM-5E461
Breast - Mammary Tissue	Breast	GTEX-ZF3C-2326-SM-5S2MZ
Breast - Mammary Tissue	Breast	GTEX-ZLFU-2126-SM-4WWEV
Breast - Mammary Tissue	Breast	GTEX-ZLV1-1426-SM-4WWES
Breast - Mammary Tissue	Breast	GTEX-ZPIC-1126-SM-5BC7F
Breast - Mammary Tissue	Breast	GTEX-ZQG8-0726-SM-5P9H9
Breast - Mammary Tissue	Breast	GTEX-ZQUD-1926-SM-51MSA
Breast - Mammary Tissue	Breast	GTEX-ZT9W-2026-SM-51MRA
Breast - Mammary Tissue	Breast	GTEX-ZTTD-1026-SM-51MRD
Breast - Mammary Tissue	Breast	GTEX-ZTX8-1226-SM-4YCE9
Breast - Mammary Tissue	Breast	GTEX-ZU9S-1926-SM-5NQBP
Breast - Mammary Tissue	Breast	GTEX-ZUA1-1526-SM-59HLS
Breast - Mammary Tissue	Breast	GTEX-ZV6S-1826-SM-5NQ8D
Breast - Mammary Tissue	Breast	GTEX-ZV7C-1826-SM-5NQ83
Breast - Mammary Tissue	Breast	GTEX-ZVE2-1226-SM-5NQ8R
Breast - Mammary Tissue	Breast	GTEX-ZVT2-1826-SM-5NQ8W
Breast - Mammary Tissue	Breast	GTEX-ZVT4-1026-SM-57WC4
Breast - Mammary Tissue	Breast	GTEX-ZVTK-0326-SM-51MRR
Breast - Mammary Tissue	Breast	GTEX-ZVZQ-0826-SM-51MRF
Breast - Mammary Tissue	Breast	GTEX-ZWKS-2826-SM-5NQ74
Breast - Mammary Tissue	Breast	GTEX-ZXES-0826-SM-5E43C

Breast - Mammary Tissue Breast GTEX-ZY6K-1626-SM-5GZWV
Breast - Mammary Tissue Breast GTEX-ZYFC-0826-SM-5E44K
Breast - Mammary Tissue Breast GTEX-ZYT6-0126-SM-5E45J
Breast - Mammary Tissue Breast GTEX-ZYW4-0826-SM-5GIDG
Breast - Mammary Tissue Breast GTEX-ZZ64-1226-SM-5E43R
Breast - Mammary Tissue Breast GTEX-ZZPU-0626-SM-5E43T
Cells - EBV-transformed lymphocytes Blood GTEX-1122O-0003-SM-5Q5DL
Cells - EBV-transformed lymphocytes Blood GTEX-11EM3-0001-SM-5Q5BD
Cells - EBV-transformed lymphocytes Blood GTEX-11EMC-0002-SM-5Q5DO
Cells - EBV-transformed lymphocytes Blood GTEX-11EQ9-0002-SM-5QGQ1
Cells - EBV-transformed lymphocytes Blood GTEX-11I78-0001-SM-5Q5BE
Cells - EBV-transformed lymphocytes Blood GTEX-11OC5-0004-SM-5S2O6
Cells - EBV-transformed lymphocytes Blood GTEX-11P7K-0003-SM-5S2OU
Cells - EBV-transformed lymphocytes Blood GTEX-11TT1-0004-SM-5S2NT
Cells - EBV-transformed lymphocytes Blood GTEX-11VI4-0001-SM-5S2OI
Cells - EBV-transformed lymphocytes Blood GTEX-11ZTT-0002-SM-5S2PA
Cells - EBV-transformed lymphocytes Blood GTEX-1211K-0001-SM-5S2P9
Cells - EBV-transformed lymphocytes Blood GTEX-1212Z-0002-SM-5SI6W
Cells - EBV-transformed lymphocytes Blood GTEX-12696-0002-SM-5SI6U
Cells - EBV-transformed lymphocytes Blood GTEX-1269C-0003-SM-5S2PB
Cells - EBV-transformed lymphocytes Blood GTEX-12BJ1-0003-SM-5SI6V
Cells - EBV-transformed lymphocytes Blood GTEX-12C56-0002-SM-5S2PC
Cells - EBV-transformed lymphocytes Blood GTEX-12WSJ-0002-SM-5TDBV
Cells - EBV-transformed lymphocytes Blood GTEX-12WSL-0002-SM-664MF
Cells - EBV-transformed lymphocytes Blood GTEX-12WSN-0001-SM-664MG
Cells - EBV-transformed lymphocytes Blood GTEX-13111-0002-SM-5TDBU
Cells - EBV-transformed lymphocytes Blood GTEX-132QS-0001-SM-62LDA
Cells - EBV-transformed lymphocytes Blood GTEX-1399R-0002-SM-62LD9
Cells - EBV-transformed lymphocytes Blood GTEX-RWS6-0001-SM-3NMAL
Cells - EBV-transformed lymphocytes Blood GTEX-S4Q7-0003-SM-3NM8M

Cells - EBV-transformed lymphocytes Blood GTEX-S95S-0002-
SM-3NM8K
Cells - EBV-transformed lymphocytes Blood GTEX-SN8G-0001-
SM-3NM8L
Cells - EBV-transformed lymphocytes Blood GTEX-SNOS-0003-
SM-3NMAO
Cells - EBV-transformed lymphocytes Blood GTEX-SSA3-0002-
SM-3P61R
Cells - EBV-transformed lymphocytes Blood GTEX-T5JC-0001-
SM-3NMAK
Cells - EBV-transformed lymphocytes Blood GTEX-T5JW-0003-
SM-3NMAD
Cells - EBV-transformed lymphocytes Blood GTEX-T6MN-0002-
SM-3NMAH
Cells - EBV-transformed lymphocytes Blood GTEX-T6MO-0003-
SM-3NMAG
Cells - EBV-transformed lymphocytes Blood GTEX-TKQ1-0003-
SM-3NMAE
Cells - EBV-transformed lymphocytes Blood GTEX-TKQ2-0004-
SM-3NMAC
Cells - EBV-transformed lymphocytes Blood GTEX-TML8-0001-
SM-3NMAF
Cells - EBV-transformed lymphocytes Blood GTEX-TMZS-0001-
SM-3P61Q
Cells - EBV-transformed lymphocytes Blood GTEX-U3ZG-0001-
SM-47JYF
Cells - EBV-transformed lymphocytes Blood GTEX-U3ZH-0002-
SM-3NMDD
Cells - EBV-transformed lymphocytes Blood GTEX-U3ZM-0002-
SM-3NMDM
Cells - EBV-transformed lymphocytes Blood GTEX-U3ZN-0002-
SM-3NMDF
Cells - EBV-transformed lymphocytes Blood GTEX-UPJH-0001-
SM-3NMDE
Cells - EBV-transformed lymphocytes Blood GTEX-UPK5-0003-
SM-3NMDI
Cells - EBV-transformed lymphocytes Blood GTEX-V1D1-0003-
SM-3NMDP
Cells - EBV-transformed lymphocytes Blood GTEX-V955-0004-
SM-3NMDH
Cells - EBV-transformed lymphocytes Blood GTEX-VJYA-0001-
SM-3NMDJ
Cells - EBV-transformed lymphocytes Blood GTEX-VUSG-0003-
SM-3NMDK
Cells - EBV-transformed lymphocytes Blood GTEX-VUSH-0004-
SM-3P61T
Cells - EBV-transformed lymphocytes Blood GTEX-W5WG-0002-
SM-3NMDN
Cells - EBV-transformed lymphocytes Blood GTEX-W5X1-0001-
SM-3P61V
Cells - EBV-transformed lymphocytes Blood GTEX-WCDI-0002-
SM-3P61U
Cells - EBV-transformed lymphocytes Blood GTEX-WEY5-0001-
SM-3P61Z

Cells - EBV-transformed lymphocytes	Blood	GTEX-WFG7-0001-SM-3P61S
Cells - EBV-transformed lymphocytes	Blood	GTEX-WFG8-0001-SM-4LVN8
Cells - EBV-transformed lymphocytes	Blood	GTEX-WFJO-0002-SM-3P61X
Cells - EBV-transformed lymphocytes	Blood	GTEX-WFON-0001-SM-3P61W
Cells - EBV-transformed lymphocytes	Blood	GTEX-WH7G-0002-SM-4LVN9
Cells - EBV-transformed lymphocytes	Blood	GTEX-WHPG-0004-SM-3NMDO
Cells - EBV-transformed lymphocytes	Blood	GTEX-WHSB-0002-SM-4M1ZR
Cells - EBV-transformed lymphocytes	Blood	GTEX-WHWD-0003-SM-4M1ZS
Cells - EBV-transformed lymphocytes	Blood	GTEX-WOFM-0001-SM-4OOT2
Cells - EBV-transformed lymphocytes	Blood	GTEX-WRHK-0001-SM-4WWDD
Cells - EBV-transformed lymphocytes	Blood	GTEX-WWTW-0002-SM-4MVNH
Cells - EBV-transformed lymphocytes	Blood	GTEX-WXYG-0004-SM-4MVOS
Cells - EBV-transformed lymphocytes	Blood	GTEX-WY7C-0004-SM-4ONDS
Cells - EBV-transformed lymphocytes	Blood	GTEX-WYVS-0004-SM-4ONDT
Cells - EBV-transformed lymphocytes	Blood	GTEX-WZTO-0001-SM-4PQZY
Cells - EBV-transformed lymphocytes	Blood	GTEX-X15G-0001-SM-4PQZV
Cells - EBV-transformed lymphocytes	Blood	GTEX-X3Y1-0001-SM-4PQYP
Cells - EBV-transformed lymphocytes	Blood	GTEX-X4EO-0004-SM-4QARM
Cells - EBV-transformed lymphocytes	Blood	GTEX-X4EP-0004-SM-4PQZX
Cells - EBV-transformed lymphocytes	Blood	GTEX-X4LF-0002-SM-4QASG
Cells - EBV-transformed lymphocytes	Blood	GTEX-X585-0002-SM-46MVA
Cells - EBV-transformed lymphocytes	Blood	GTEX-X5EB-0004-SM-46MWA
Cells - EBV-transformed lymphocytes	Blood	GTEX-X638-0003-SM-47JZ1
Cells - EBV-transformed lymphocytes	Blood	GTEX-X88G-0004-SM-47JZ6
Cells - EBV-transformed lymphocytes	Blood	GTEX-XBED-0003-SM-47JWP
Cells - EBV-transformed lymphocytes	Blood	GTEX-XBEW-0002-SM-4AT5O
Cells - EBV-transformed lymphocytes	Blood	GTEX-XGQ4-0004-SM-4AT5S

Cells - EBV-transformed lymphocytes Blood GTEX-XLM4-0004-SM-4AT5I
Cells - EBV-transformed lymphocytes Blood GTEX-XMK1-0001-SM-4B64F
Cells - EBV-transformed lymphocytes Blood GTEX-XPT6-0001-SM-4B64G
Cells - EBV-transformed lymphocytes Blood GTEX-XQ3S-0001-SM-4B64K
Cells - EBV-transformed lymphocytes Blood GTEX-XUYS-0002-SM-47JXL
Cells - EBV-transformed lymphocytes Blood GTEX-XXEK-0004-SM-4BRWO
Cells - EBV-transformed lymphocytes Blood GTEX-XYKS-0002-SM-4BRWN
Cells - EBV-transformed lymphocytes Blood GTEX-Y114-0002-SM-4TT78
Cells - EBV-transformed lymphocytes Blood GTEX-Y3IK-0001-SM-4WWE1
Cells - EBV-transformed lymphocytes Blood GTEX-Y5LM-0003-SM-4V6G1
Cells - EBV-transformed lymphocytes Blood GTEX-Y5V5-0001-SM-4V6FZ
Cells - EBV-transformed lymphocytes Blood GTEX-Y5V6-0003-SM-4V6FX
Cells - EBV-transformed lymphocytes Blood GTEX-Y8DK-0004-SM-4RGM7
Cells - EBV-transformed lymphocytes Blood GTEX-Y8E4-0003-SM-4V6FY
Cells - EBV-transformed lymphocytes Blood GTEX-Y8E5-0002-SM-57WD9
Cells - EBV-transformed lymphocytes Blood GTEX-Y8LW-0002-SM-4V6G2
Cells - EBV-transformed lymphocytes Blood GTEX-Y9LG-0001-SM-4VBRQ
Cells - EBV-transformed lymphocytes Blood GTEX-YB5E-0001-SM-4VDSV
Cells - EBV-transformed lymphocytes Blood GTEX-YB5K-0003-SM-4VDSN
Cells - EBV-transformed lymphocytes Blood GTEX-YEC3-0002-SM-4W1YI
Cells - EBV-transformed lymphocytes Blood GTEX-YEC4-0002-SM-4W1Z6
Cells - EBV-transformed lymphocytes Blood GTEX-YF7O-0004-SM-4W1ZT
Cells - EBV-transformed lymphocytes Blood GTEX-YFCO-0003-SM-4W21I
Cells - EBV-transformed lymphocytes Blood GTEX-ZC5H-0004-SM-4WAXK
Cells - EBV-transformed lymphocytes Blood GTEX-ZDTS-0001-SM-4WAXW
Cells - EBV-transformed lymphocytes Blood GTEX-ZDTT-0004-SM-4WKG3
Cells - EBV-transformed lymphocytes Blood GTEX-ZEX8-0004-SM-4WKFQ

Cells - EBV-transformed lymphocytes Blood GTEX-ZF29-0002-
SM-4WKF2
Cells - EBV-transformed lymphocytes Blood GTEX-ZF2S-0004-
SM-4WKFE
Cells - EBV-transformed lymphocytes Blood GTEX-ZF3C-0001-
SM-4WWAW
Cells - EBV-transformed lymphocytes Blood GTEX-ZG7Y-0003-
SM-4WWEJ
Cells - EBV-transformed lymphocytes Blood GTEX-ZLV1-0001-
SM-4WWAO
Cells - EBV-transformed lymphocytes Blood GTEX-ZLWG-0004-
SM-4WWD5
Cells - EBV-transformed lymphocytes Blood GTEX-ZP4G-0003-
SM-4WWED
Cells - EBV-transformed lymphocytes Blood GTEX-ZPCL-0004-
SM-4WWD6
Cells - EBV-transformed lymphocytes Blood GTEX-ZPIC-0002-
SM-4WWEC
Cells - EBV-transformed lymphocytes Blood GTEX-ZPU1-0004-
SM-4WWAV
Cells - EBV-transformed lymphocytes Blood GTEX-ZQG8-0001-
SM-4YCDH
Cells - EBV-transformed lymphocytes Blood GTEX-ZQUD-0003-
SM-4YCD3
Cells - EBV-transformed lymphocytes Blood GTEX-ZT9W-0003-
SM-4YCE6
Cells - EBV-transformed lymphocytes Blood GTEX-ZT9X-0004-
SM-4YCDT
Cells - EBV-transformed lymphocytes Blood GTEX-ZTPG-0002-
SM-4YCEI
Cells - EBV-transformed lymphocytes Blood GTEX-ZTSS-0004-
SM-4YCCS
Cells - EBV-transformed lymphocytes Blood GTEX-ZTTD-0001-
SM-4YCD5
Cells - EBV-transformed lymphocytes Blood GTEX-ZTX8-0002-
SM-4YCEU
Cells - EBV-transformed lymphocytes Blood GTEX-ZUA1-0002-
SM-4YCF7
Cells - EBV-transformed lymphocytes Blood GTEX-ZV6S-0003-
SM-4YCCT
Cells - EBV-transformed lymphocytes Blood GTEX-ZV7C-0003-
SM-4YCF6
Cells - EBV-transformed lymphocytes Blood GTEX-ZVT2-0001-
SM-57WCK
Cells - EBV-transformed lymphocytes Blood GTEX-ZVTK-0003-
SM-51MRV
Cells - EBV-transformed lymphocytes Blood GTEX-ZVZP-0004-
SM-51MS8
Cells - EBV-transformed lymphocytes Blood GTEX-ZXES-0004-
SM-57WCW
Cells - Transformed fibroblasts Skin GTEX-111VG-0008-SM-5Q5BG
Cells - Transformed fibroblasts Skin GTEX-111YS-0008-SM-5Q5BH
Cells - Transformed fibroblasts Skin GTEX-1122O-0008-SM-5QGR2
Cells - Transformed fibroblasts Skin GTEX-1128S-0008-SM-5Q5DP

Cells - Transformed fibroblasts	Skin	GTEX-113IC-0008-SM-5QGRF
Cells - Transformed fibroblasts	Skin	GTEX-113JC-0008-SM-5QGR6
Cells - Transformed fibroblasts	Skin	GTEX-117XS-0008-SM-5Q5DQ
Cells - Transformed fibroblasts	Skin	GTEX-117YW-0008-SM-5QGRD
Cells - Transformed fibroblasts	Skin	GTEX-1192W-0008-SM-5QGRE
Cells - Transformed fibroblasts	Skin	GTEX-1192X-0008-SM-5Q5B7
Cells - Transformed fibroblasts	Skin	GTEX-11DXW-0008-SM-5Q59V
Cells - Transformed fibroblasts	Skin	GTEX-11DXX-0008-SM-5Q5B8
Cells - Transformed fibroblasts	Skin	GTEX-11DXY-0008-SM-5QGR4
Cells - Transformed fibroblasts	Skin	GTEX-11DYG-0008-SM-5QGR5
Cells - Transformed fibroblasts	Skin	GTEX-11DZ1-0008-SM-5QGR3
Cells - Transformed fibroblasts	Skin	GTEX-11EI6-0008-SM-5QGR7
Cells - Transformed fibroblasts	Skin	GTEX-11EMC-0008-SM-5Q5DR
Cells - Transformed fibroblasts	Skin	GTEX-11EQ8-0008-SM-5Q5DJ
Cells - Transformed fibroblasts	Skin	GTEX-11GS4-0008-SM-5QGRA
Cells - Transformed fibroblasts	Skin	GTEX-11GSP-0008-SM-5Q5DM
Cells - Transformed fibroblasts	Skin	GTEX-11H98-0008-SM-5QGR8
Cells - Transformed fibroblasts	Skin	GTEX-11I78-0008-SM-5Q5DI
Cells - Transformed fibroblasts	Skin	GTEX-11LCK-0008-SM-5Q5BB
Cells - Transformed fibroblasts	Skin	GTEX-11NSD-0008-SM-5Q5BC
Cells - Transformed fibroblasts	Skin	GTEX-11NUK-0008-SM-5Q5B9
Cells - Transformed fibroblasts	Skin	GTEX-11NV4-0008-SM-5Q5BA
Cells - Transformed fibroblasts	Skin	GTEX-11O72-0008-SM-5Q5DN
Cells - Transformed fibroblasts	Skin	GTEX-11OC5-0008-SM-5S2OH
Cells - Transformed fibroblasts	Skin	GTEX-11OF3-0008-SM-5S2NH
Cells - Transformed fibroblasts	Skin	GTEX-11ONC-0008-SM-5S2MG
Cells - Transformed fibroblasts	Skin	GTEX-11P7K-0008-SM-5S2O5
Cells - Transformed fibroblasts	Skin	GTEX-11P81-0008-SM-5S2OT
Cells - Transformed fibroblasts	Skin	GTEX-11P82-0008-SM-5S2MS
Cells - Transformed fibroblasts	Skin	GTEX-11PRG-0008-SM-5S2N5
Cells - Transformed fibroblasts	Skin	GTEX-11TT1-0008-SM-5S2P8
Cells - Transformed fibroblasts	Skin	GTEX-11TTK-0008-SM-5S2RU
Cells - Transformed fibroblasts	Skin	GTEX-11TUW-0008-SM-5SI6S
Cells - Transformed fibroblasts	Skin	GTEX-11UD1-0008-SM-5S2P6
Cells - Transformed fibroblasts	Skin	GTEX-11UD2-0008-SM-5S2P7
Cells - Transformed fibroblasts	Skin	GTEX-11WQC-0008-SM-5SI6R
Cells - Transformed fibroblasts	Skin	GTEX-11WQK-0008-SM-5SI6T
Cells - Transformed fibroblasts	Skin	GTEX-11XUK-0008-SM-5S2WD
Cells - Transformed fibroblasts	Skin	GTEX-11ZTS-0008-SM-5S2VC
Cells - Transformed fibroblasts	Skin	GTEX-11ZTT-0008-SM-5S2TZ
Cells - Transformed fibroblasts	Skin	GTEX-11ZU8-0008-SM-5S2VO
Cells - Transformed fibroblasts	Skin	GTEX-11ZUS-0008-SM-5S2UO
Cells - Transformed fibroblasts	Skin	GTEX-1211K-0008-SM-5S2W1
Cells - Transformed fibroblasts	Skin	GTEX-12126-0008-SM-5S2UC
Cells - Transformed fibroblasts	Skin	GTEX-1212Z-0008-SM-5TDBW
Cells - Transformed fibroblasts	Skin	GTEX-12584-0008-SM-664MB
Cells - Transformed fibroblasts	Skin	GTEX-12696-0008-SM-5TDBX
Cells - Transformed fibroblasts	Skin	GTEX-1269C-0008-SM-5TDBY
Cells - Transformed fibroblasts	Skin	GTEX-12BJ1-0008-SM-5TDBZ
Cells - Transformed fibroblasts	Skin	GTEX-12C56-0008-SM-5TDC1
Cells - Transformed fibroblasts	Skin	GTEX-12KS4-0008-SM-664MC
Cells - Transformed fibroblasts	Skin	GTEX-12WSA-0008-SM-5TDC3
Cells - Transformed fibroblasts	Skin	GTEX-12WSC-0008-SM-664MK
Cells - Transformed fibroblasts	Skin	GTEX-12WSD-0008-SM-664ME

Cells - Transformed fibroblasts	Skin	GTEX-12WSE-0008-SM-5YY9P
Cells - Transformed fibroblasts	Skin	GTEX-12WSF-0008-SM-5YY8S
Cells - Transformed fibroblasts	Skin	GTEX-12WSG-0008-SM-5TDC4
Cells - Transformed fibroblasts	Skin	GTEX-12WSH-0008-SM-5S2V1
Cells - Transformed fibroblasts	Skin	GTEX-12WSI-0008-SM-664MD
Cells - Transformed fibroblasts	Skin	GTEX-12WSJ-0008-SM-664ML
Cells - Transformed fibroblasts	Skin	GTEX-12WSK-0008-SM-5YY9R
Cells - Transformed fibroblasts	Skin	GTEX-12WSL-0008-SM-664MM
Cells - Transformed fibroblasts	Skin	GTEX-12WSM-0008-SM-5S2VD
Cells - Transformed fibroblasts	Skin	GTEX-12WSN-0008-SM-664MI
Cells - Transformed fibroblasts	Skin	GTEX-12ZZW-0008-SM-5YY9L
Cells - Transformed fibroblasts	Skin	GTEX-12ZZX-0008-SM-5YY9N
Cells - Transformed fibroblasts	Skin	GTEX-12ZZY-0008-SM-5YY9Q
Cells - Transformed fibroblasts	Skin	GTEX-12ZZZ-0008-SM-5YY9S
Cells - Transformed fibroblasts	Skin	GTEX-13111-0008-SM-5YY8R
Cells - Transformed fibroblasts	Skin	GTEX-13112-0008-SM-5YY9M
Cells - Transformed fibroblasts	Skin	GTEX-13113-0008-SM-5YY9T
Cells - Transformed fibroblasts	Skin	GTEX-1313W-0008-SM-5YYAP
Cells - Transformed fibroblasts	Skin	GTEX-1314G-0008-SM-5YY9K
Cells - Transformed fibroblasts	Skin	GTEX-131XF-0008-SM-664MH
Cells - Transformed fibroblasts	Skin	GTEX-131XG-0008-SM-5YY9O
Cells - Transformed fibroblasts	Skin	GTEX-131XH-0008-SM-5YYAC
Cells - Transformed fibroblasts	Skin	GTEX-131XW-0008-SM-664MJ
Cells - Transformed fibroblasts	Skin	GTEX-131YS-0008-SM-5YY9Z
Cells - Transformed fibroblasts	Skin	GTEX-132AR-0008-SM-62LDH
Cells - Transformed fibroblasts	Skin	GTEX-132NY-0008-SM-62LFX
Cells - Transformed fibroblasts	Skin	GTEX-132Q8-0008-SM-5ZZUC
Cells - Transformed fibroblasts	Skin	GTEX-132QS-0008-SM-5ZZUB
Cells - Transformed fibroblasts	Skin	GTEX-1339X-0008-SM-5ZZUL
Cells - Transformed fibroblasts	Skin	GTEX-133LE-0008-SM-62LDD
Cells - Transformed fibroblasts	Skin	GTEX-1399Q-0008-SM-62LDE
Cells - Transformed fibroblasts	Skin	GTEX-1399R-0008-SM-5ZZUF
Cells - Transformed fibroblasts	Skin	GTEX-1399S-0008-SM-62LDF
Cells - Transformed fibroblasts	Skin	GTEX-1399T-0008-SM-62LDB
Cells - Transformed fibroblasts	Skin	GTEX-1399U-0008-SM-5S2VE
Cells - Transformed fibroblasts	Skin	GTEX-139D8-0008-SM-5ZZUH
Cells - Transformed fibroblasts	Skin	GTEX-139T4-0008-SM-5S2WF
Cells - Transformed fibroblasts	Skin	GTEX-139T8-0008-SM-5ZZUD
Cells - Transformed fibroblasts	Skin	GTEX-139TS-0008-SM-62LDG
Cells - Transformed fibroblasts	Skin	GTEX-139TU-0008-SM-5ZZUK
Cells - Transformed fibroblasts	Skin	GTEX-139YR-0008-SM-5ZZUM
Cells - Transformed fibroblasts	Skin	GTEX-13CF2-0008-SM-62LDC
Cells - Transformed fibroblasts	Skin	GTEX-13CZU-0008-SM-5ZZUI
Cells - Transformed fibroblasts	Skin	GTEX-13CZV-0008-SM-5ZZUE
Cells - Transformed fibroblasts	Skin	GTEX-13FH7-0008-SM-5ZZUJ
Cells - Transformed fibroblasts	Skin	GTEX-N7MS-0008-SM-4E3JI
Cells - Transformed fibroblasts	Skin	GTEX-NFK9-0008-SM-4E3JE
Cells - Transformed fibroblasts	Skin	GTEX-NL3G-0008-SM-4E3JX
Cells - Transformed fibroblasts	Skin	GTEX-NL3H-0008-SM-4E3HU
Cells - Transformed fibroblasts	Skin	GTEX-NL4W-0008-SM-4E3I2
Cells - Transformed fibroblasts	Skin	GTEX-NPJ7-0008-SM-4E3JS
Cells - Transformed fibroblasts	Skin	GTEX-O5YT-0008-SM-4E3IQ
Cells - Transformed fibroblasts	Skin	GTEX-O5YU-0008-SM-4E3I7
Cells - Transformed fibroblasts	Skin	GTEX-O5YV-0008-SM-4E3HP

Cells - Transformed fibroblasts	Skin	GTEX-O5YW-0008-SM-4E3IE
Cells - Transformed fibroblasts	Skin	GTEX-OHPK-0008-SM-4E3JL
Cells - Transformed fibroblasts	Skin	GTEX-OHPL-0008-SM-4E3I9
Cells - Transformed fibroblasts	Skin	GTEX-OHPM-0008-SM-4E3IP
Cells - Transformed fibroblasts	Skin	GTEX-OHPN-0008-SM-4E3HW
Cells - Transformed fibroblasts	Skin	GTEX-OIZG-0008-SM-4E3J2
Cells - Transformed fibroblasts	Skin	GTEX-OIZI-0008-SM-2XCFD
Cells - Transformed fibroblasts	Skin	GTEX-OOBJ-0008-SM-3NB26
Cells - Transformed fibroblasts	Skin	GTEX-OOBK-0008-SM-3NB27
Cells - Transformed fibroblasts	Skin	GTEX-OXRK-0008-SM-3NB28
Cells - Transformed fibroblasts	Skin	GTEX-OXRL-0008-SM-3NB29
Cells - Transformed fibroblasts	Skin	GTEX-P44H-0008-SM-48TDW
Cells - Transformed fibroblasts	Skin	GTEX-P4PP-0008-SM-48TDV
Cells - Transformed fibroblasts	Skin	GTEX-P4PQ-0008-SM-48TDX
Cells - Transformed fibroblasts	Skin	GTEX-P4QS-0008-SM-48TDY
Cells - Transformed fibroblasts	Skin	GTEX-P4QT-0008-SM-48TDZ
Cells - Transformed fibroblasts	Skin	GTEX-P78B-0008-SM-48TE1
Cells - Transformed fibroblasts	Skin	GTEX-PLZ4-0008-SM-48TE6
Cells - Transformed fibroblasts	Skin	GTEX-PLZ6-0008-SM-48TD5
Cells - Transformed fibroblasts	Skin	GTEX-POMQ-0008-SM-48TE7
Cells - Transformed fibroblasts	Skin	GTEX-POYW-0008-SM-48TE4
Cells - Transformed fibroblasts	Skin	GTEX-PSDG-0008-SM-48TE5
Cells - Transformed fibroblasts	Skin	GTEX-PVOW-0008-SM-48TE8
Cells - Transformed fibroblasts	Skin	GTEX-PW2O-0008-SM-48TEB
Cells - Transformed fibroblasts	Skin	GTEX-PWCY-0008-SM-48TE9
Cells - Transformed fibroblasts	Skin	GTEX-PWN1-0008-SM-48TEA
Cells - Transformed fibroblasts	Skin	GTEX-PX3G-0008-SM-48U2L
Cells - Transformed fibroblasts	Skin	GTEX-Q2AH-0008-SM-48U2J
Cells - Transformed fibroblasts	Skin	GTEX-Q2AI-0008-SM-48U2H
Cells - Transformed fibroblasts	Skin	GTEX-Q734-0008-SM-48U2I
Cells - Transformed fibroblasts	Skin	GTEX-QCQG-0008-SM-48U2G
Cells - Transformed fibroblasts	Skin	GTEX-QDT8-0008-SM-48U2F
Cells - Transformed fibroblasts	Skin	GTEX-QDVJ-0008-SM-48U2E
Cells - Transformed fibroblasts	Skin	GTEX-QDVN-0008-SM-48U2D
Cells - Transformed fibroblasts	Skin	GTEX-QEG4-0008-SM-48TYZ
Cells - Transformed fibroblasts	Skin	GTEX-QEG5-0008-SM-4R1KL
Cells - Transformed fibroblasts	Skin	GTEX-QEL4-0008-SM-447AZ
Cells - Transformed fibroblasts	Skin	GTEX-QESD-0008-SM-447B2
Cells - Transformed fibroblasts	Skin	GTEX-QLQ7-0008-SM-447AW
Cells - Transformed fibroblasts	Skin	GTEX-QMR6-0008-SM-447AV
Cells - Transformed fibroblasts	Skin	GTEX-QMRM-0008-SM-447B1
Cells - Transformed fibroblasts	Skin	GTEX-QV31-0008-SM-447AT
Cells - Transformed fibroblasts	Skin	GTEX-QV44-0008-SM-447AX
Cells - Transformed fibroblasts	Skin	GTEX-QVJO-0008-SM-447AU
Cells - Transformed fibroblasts	Skin	GTEX-QVUS-0008-SM-447AY
Cells - Transformed fibroblasts	Skin	GTEX-QXCU-0008-SM-48FCH
Cells - Transformed fibroblasts	Skin	GTEX-R3RS-0008-SM-48FF1
Cells - Transformed fibroblasts	Skin	GTEX-R45C-0008-SM-48FF2
Cells - Transformed fibroblasts	Skin	GTEX-R53T-0008-SM-48FEW
Cells - Transformed fibroblasts	Skin	GTEX-R55C-0008-SM-48FCF
Cells - Transformed fibroblasts	Skin	GTEX-R55D-0008-SM-48FEV
Cells - Transformed fibroblasts	Skin	GTEX-R55E-0008-SM-48FCG
Cells - Transformed fibroblasts	Skin	GTEX-R55G-0008-SM-48FEX
Cells - Transformed fibroblasts	Skin	GTEX-RM2N-0008-SM-48FF3

Cells - Transformed fibroblasts	Skin	GTEX-RN64-0008-SM-48FEZ
Cells - Transformed fibroblasts	Skin	GTEX-RNOR-0008-SM-48FEY
Cells - Transformed fibroblasts	Skin	GTEX-RTLS-0008-SM-48FET
Cells - Transformed fibroblasts	Skin	GTEX-RU1J-0008-SM-46MV9
Cells - Transformed fibroblasts	Skin	GTEX-RU72-0008-SM-46MV8
Cells - Transformed fibroblasts	Skin	GTEX-RVPV-0008-SM-47JYW
Cells - Transformed fibroblasts	Skin	GTEX-RWS6-0008-SM-47JYV
Cells - Transformed fibroblasts	Skin	GTEX-RWSA-0008-SM-47JYX
Cells - Transformed fibroblasts	Skin	GTEX-S33H-0008-SM-4AD6C
Cells - Transformed fibroblasts	Skin	GTEX-S341-0008-SM-4AD6D
Cells - Transformed fibroblasts	Skin	GTEX-S3XE-0008-SM-3NM8O
Cells - Transformed fibroblasts	Skin	GTEX-S4P3-0008-SM-3NM8R
Cells - Transformed fibroblasts	Skin	GTEX-S4Q7-0008-SM-3NM8A
Cells - Transformed fibroblasts	Skin	GTEX-S4UY-0008-SM-3NM8H
Cells - Transformed fibroblasts	Skin	GTEX-S4Z8-0008-SM-33HAZ
Cells - Transformed fibroblasts	Skin	GTEX-S7PM-0008-SM-3NM9Q
Cells - Transformed fibroblasts	Skin	GTEX-S7SE-0008-SM-33HB1
Cells - Transformed fibroblasts	Skin	GTEX-S7SF-0008-SM-3NM8T
Cells - Transformed fibroblasts	Skin	GTEX-S95S-0008-SM-4AT5M
Cells - Transformed fibroblasts	Skin	GTEX-SE5C-0008-SM-4B64J
Cells - Transformed fibroblasts	Skin	GTEX-SIU8-0008-SM-4BRUC
Cells - Transformed fibroblasts	Skin	GTEX-SJXC-0008-SM-4DM7G
Cells - Transformed fibroblasts	Skin	GTEX-SN8G-0008-SM-4DM4X
Cells - Transformed fibroblasts	Skin	GTEX-SNMC-0008-SM-4DM5A
Cells - Transformed fibroblasts	Skin	GTEX-SNOS-0008-SM-4DM6I
Cells - Transformed fibroblasts	Skin	GTEX-SSA3-0008-SM-47JWJ
Cells - Transformed fibroblasts	Skin	GTEX-T2IS-0008-SM-4DM75
Cells - Transformed fibroblasts	Skin	GTEX-T2YK-0008-SM-4DM6M
Cells - Transformed fibroblasts	Skin	GTEX-T5JC-0008-SM-4DM6A
Cells - Transformed fibroblasts	Skin	GTEX-T5JW-0008-SM-4DM5X
Cells - Transformed fibroblasts	Skin	GTEX-T6MN-0008-SM-4DM7H
Cells - Transformed fibroblasts	Skin	GTEX-T6MO-0008-SM-4DM6T
Cells - Transformed fibroblasts	Skin	GTEX-TKQ1-0008-SM-4DXSO
Cells - Transformed fibroblasts	Skin	GTEX-TKQ2-0008-SM-4DM5L
Cells - Transformed fibroblasts	Skin	GTEX-TML8-0008-SM-4DXUI
Cells - Transformed fibroblasts	Skin	GTEX-TMMY-0008-SM-4DXU3
Cells - Transformed fibroblasts	Skin	GTEX-TMZS-0008-SM-47JWM
Cells - Transformed fibroblasts	Skin	GTEX-TSE9-0008-SM-4DXT8
Cells - Transformed fibroblasts	Skin	GTEX-U3ZG-0008-SM-47JWL
Cells - Transformed fibroblasts	Skin	GTEX-U3ZH-0008-SM-4DXT2
Cells - Transformed fibroblasts	Skin	GTEX-U3ZM-0008-SM-4DXTQ
Cells - Transformed fibroblasts	Skin	GTEX-U3ZN-0008-SM-4DXTL
Cells - Transformed fibroblasts	Skin	GTEX-U412-0008-SM-4DXTE
Cells - Transformed fibroblasts	Skin	GTEX-U4B1-0008-SM-4DXUW
Cells - Transformed fibroblasts	Skin	GTEX-U8T8-0008-SM-4DXSP
Cells - Transformed fibroblasts	Skin	GTEX-U8XE-0008-SM-4E3K4
Cells - Transformed fibroblasts	Skin	GTEX-UJHI-0008-SM-4IHL1
Cells - Transformed fibroblasts	Skin	GTEX-UJMC-0008-SM-4IHKK
Cells - Transformed fibroblasts	Skin	GTEX-UPK5-0008-SM-4IHJD
Cells - Transformed fibroblasts	Skin	GTEX-UTHO-0008-SM-4JBID
Cells - Transformed fibroblasts	Skin	GTEX-V1D1-0008-SM-4JBIJ
Cells - Transformed fibroblasts	Skin	GTEX-VUSG-0008-SM-4KL24
Cells - Transformed fibroblasts	Skin	GTEX-VUSH-0008-SM-47JWK
Cells - Transformed fibroblasts	Skin	GTEX-W5WG-0008-SM-4KL25

Cells - Transformed fibroblasts	Skin	GTEX-W5X1-0008-SM-4LMKA
Cells - Transformed fibroblasts	Skin	GTEX-WCDI-0008-SM-47JYE
Cells - Transformed fibroblasts	Skin	GTEX-WEY5-0008-SM-4LMKC
Cells - Transformed fibroblasts	Skin	GTEX-WFG7-0008-SM-4LMKB
Cells - Transformed fibroblasts	Skin	GTEX-WFG8-0008-SM-4LVN6
Cells - Transformed fibroblasts	Skin	GTEX-WFJO-0008-SM-4LVN7
Cells - Transformed fibroblasts	Skin	GTEX-WH7G-0008-SM-4LVNM
Cells - Transformed fibroblasts	Skin	GTEX-WHPG-0008-SM-4M1ZQ
Cells - Transformed fibroblasts	Skin	GTEX-WHSB-0008-SM-4M1ZP
Cells - Transformed fibroblasts	Skin	GTEX-WHSE-0008-SM-4M1ZO
Cells - Transformed fibroblasts	Skin	GTEX-WHWD-0008-SM-4OOSU
Cells - Transformed fibroblasts	Skin	GTEX-WI4N-0008-SM-4OOSV
Cells - Transformed fibroblasts	Skin	GTEX-WK11-0008-SM-4OOSX
Cells - Transformed fibroblasts	Skin	GTEX-WL46-0008-SM-4OOSW
Cells - Transformed fibroblasts	Skin	GTEX-WOFL-0008-SM-4OOSZ
Cells - Transformed fibroblasts	Skin	GTEX-WOFM-0008-SM-4OOSY
Cells - Transformed fibroblasts	Skin	GTEX-WQUQ-0008-SM-4OOT1
Cells - Transformed fibroblasts	Skin	GTEX-WRHK-0008-SM-4MVPA
Cells - Transformed fibroblasts	Skin	GTEX-WRHU-0008-SM-4MVPB
Cells - Transformed fibroblasts	Skin	GTEX-WVJS-0008-SM-4MVPC
Cells - Transformed fibroblasts	Skin	GTEX-WVLH-0008-SM-4MVPD
Cells - Transformed fibroblasts	Skin	GTEX-WWTW-0008-SM-4MVPE
Cells - Transformed fibroblasts	Skin	GTEX-WWYW-0008-SM-4MVPF
Cells - Transformed fibroblasts	Skin	GTEX-WXYG-0008-SM-4ONDU
Cells - Transformed fibroblasts	Skin	GTEX-WY7C-0008-SM-4ONDW
Cells - Transformed fibroblasts	Skin	GTEX-WYBS-0008-SM-4ONDX
Cells - Transformed fibroblasts	Skin	GTEX-WYJK-0008-SM-4ONDV
Cells - Transformed fibroblasts	Skin	GTEX-WYVS-0008-SM-4ONDY
Cells - Transformed fibroblasts	Skin	GTEX-WZTO-0008-SM-4PQZZ
Cells - Transformed fibroblasts	Skin	GTEX-X15G-0008-SM-4PR2D
Cells - Transformed fibroblasts	Skin	GTEX-X261-0008-SM-4PR11
Cells - Transformed fibroblasts	Skin	GTEX-X3Y1-0008-SM-4PR12
Cells - Transformed fibroblasts	Skin	GTEX-X4EO-0008-SM-4QASS
Cells - Transformed fibroblasts	Skin	GTEX-X4EP-0008-SM-4PQYO
Cells - Transformed fibroblasts	Skin	GTEX-X4LF-0008-SM-4QAST
Cells - Transformed fibroblasts	Skin	GTEX-X4XX-0008-SM-46MVK
Cells - Transformed fibroblasts	Skin	GTEX-X4XY-0008-SM-46MVL
Cells - Transformed fibroblasts	Skin	GTEX-X585-0008-SM-46MU4
Cells - Transformed fibroblasts	Skin	GTEX-X5EB-0008-SM-46MU3
Cells - Transformed fibroblasts	Skin	GTEX-X62O-0008-SM-46MU5
Cells - Transformed fibroblasts	Skin	GTEX-X638-0008-SM-47JZ7
Cells - Transformed fibroblasts	Skin	GTEX-X88G-0008-SM-47JWN
Cells - Transformed fibroblasts	Skin	GTEX-X8HC-0008-SM-46MU6
Cells - Transformed fibroblasts	Skin	GTEX-XBEC-0008-SM-4AT3X
Cells - Transformed fibroblasts	Skin	GTEX-XBED-0008-SM-47JWO
Cells - Transformed fibroblasts	Skin	GTEX-XBEW-0008-SM-4AT3Y
Cells - Transformed fibroblasts	Skin	GTEX-XGQ4-0008-SM-4AT3Z
Cells - Transformed fibroblasts	Skin	GTEX-XK95-0008-SM-4AT5G
Cells - Transformed fibroblasts	Skin	GTEX-XLM4-0008-SM-4AT4W
Cells - Transformed fibroblasts	Skin	GTEX-XMD1-0008-SM-4AT41
Cells - Transformed fibroblasts	Skin	GTEX-XMD2-0008-SM-4WWE7
Cells - Transformed fibroblasts	Skin	GTEX-XMD3-0008-SM-4AT4V
Cells - Transformed fibroblasts	Skin	GTEX-XMK1-0008-SM-4GICF
Cells - Transformed fibroblasts	Skin	GTEX-XOT4-0008-SM-4B664

Cells - Transformed fibroblasts	Skin	GTEX-XPT6-0008-SM-4B64Q
Cells - Transformed fibroblasts	Skin	GTEX-XPVG-0008-SM-4GICH
Cells - Transformed fibroblasts	Skin	GTEX-XQ3S-0008-SM-4GIDZ
Cells - Transformed fibroblasts	Skin	GTEX-XQ8I-0008-SM-4WAWM
Cells - Transformed fibroblasts	Skin	GTEX-XUW1-0008-SM-4BOQH
Cells - Transformed fibroblasts	Skin	GTEX-XUYS-0008-SM-47JYZ
Cells - Transformed fibroblasts	Skin	GTEX-XUZC-0008-SM-4BOQG
Cells - Transformed fibroblasts	Skin	GTEX-XV7Q-0008-SM-4BRWL
Cells - Transformed fibroblasts	Skin	GTEX-XXEK-0008-SM-4BRW7
Cells - Transformed fibroblasts	Skin	GTEX-XYKS-0008-SM-4BRW6
Cells - Transformed fibroblasts	Skin	GTEX-Y111-0008-SM-4SOJ3
Cells - Transformed fibroblasts	Skin	GTEX-Y114-0008-SM-4TT8X
Cells - Transformed fibroblasts	Skin	GTEX-Y3I4-0008-SM-4TT21
Cells - Transformed fibroblasts	Skin	GTEX-Y3IK-0008-SM-4WWDZ
Cells - Transformed fibroblasts	Skin	GTEX-Y5V6-0008-SM-4V6FV
Cells - Transformed fibroblasts	Skin	GTEX-Y8DK-0008-SM-4RGM2
Cells - Transformed fibroblasts	Skin	GTEX-Y8E4-0008-SM-4V6FW
Cells - Transformed fibroblasts	Skin	GTEX-Y8E5-0008-SM-57WBA
Cells - Transformed fibroblasts	Skin	GTEX-Y8LW-0008-SM-4V6G5
Cells - Transformed fibroblasts	Skin	GTEX-Y9LG-0008-SM-4VBRJ
Cells - Transformed fibroblasts	Skin	GTEX-YB5E-0008-SM-4VDT7
Cells - Transformed fibroblasts	Skin	GTEX-YB5K-0008-SM-4VDT8
Cells - Transformed fibroblasts	Skin	GTEX-YBZK-0008-SM-59HL6
Cells - Transformed fibroblasts	Skin	GTEX-YEC4-0008-SM-4W1YR
Cells - Transformed fibroblasts	Skin	GTEX-YECK-0008-SM-4W1ZG
Cells - Transformed fibroblasts	Skin	GTEX-YF7O-0008-SM-4W1ZS
Cells - Transformed fibroblasts	Skin	GTEX-YFC4-0008-SM-4RGM3
Cells - Transformed fibroblasts	Skin	GTEX-YJ89-0008-SM-4RGM4
Cells - Transformed fibroblasts	Skin	GTEX-Z93S-0008-SM-4RGM5
Cells - Transformed fibroblasts	Skin	GTEX-ZAB4-0008-SM-4RGM6
Cells - Transformed fibroblasts	Skin	GTEX-ZC5H-0008-SM-4WAX8
Cells - Transformed fibroblasts	Skin	GTEX-ZDTS-0008-SM-4E3I8
Cells - Transformed fibroblasts	Skin	GTEX-ZDTT-0008-SM-4E3K5
Cells - Transformed fibroblasts	Skin	GTEX-ZDXO-0008-SM-4E3HR
Cells - Transformed fibroblasts	Skin	GTEX-ZDYS-0008-SM-4E3IX
Cells - Transformed fibroblasts	Skin	GTEX-ZE7O-0008-SM-4E3JQ
Cells - Transformed fibroblasts	Skin	GTEX-ZE9C-0008-SM-4E3K6
Cells - Transformed fibroblasts	Skin	GTEX-ZEX8-0008-SM-4E3JU
Cells - Transformed fibroblasts	Skin	GTEX-ZF28-0008-SM-4E3K3
Cells - Transformed fibroblasts	Skin	GTEX-ZF2S-0008-SM-4E3IK
Cells - Transformed fibroblasts	Skin	GTEX-ZF3C-0008-SM-4E3IL
Cells - Transformed fibroblasts	Skin	GTEX-ZLWG-0008-SM-4E3J4
Cells - Transformed fibroblasts	Skin	GTEX-ZP4G-0008-SM-4E3I4
Cells - Transformed fibroblasts	Skin	GTEX-ZPCL-0008-SM-4WWAU
Cells - Transformed fibroblasts	Skin	GTEX-ZPIC-0008-SM-4E3JF
Cells - Transformed fibroblasts	Skin	GTEX-ZPU1-0008-SM-4E3IR
Cells - Transformed fibroblasts	Skin	GTEX-ZQG8-0008-SM-4E3J9
Cells - Transformed fibroblasts	Skin	GTEX-ZQUD-0008-SM-4YCCU
Cells - Transformed fibroblasts	Skin	GTEX-ZT9W-0008-SM-4YCDJ
Cells - Transformed fibroblasts	Skin	GTEX-ZT9X-0008-SM-4YCD7
Cells - Transformed fibroblasts	Skin	GTEX-ZTPG-0008-SM-4YCEK
Cells - Transformed fibroblasts	Skin	GTEX-ZTSS-0008-SM-4YCE8
Cells - Transformed fibroblasts	Skin	GTEX-ZTTD-0008-SM-51MQV
Cells - Transformed fibroblasts	Skin	GTEX-ZTX8-0008-SM-4YCDV

Cells - Transformed fibroblasts	Skin	GTEX-ZUA1-0008-SM-4YCEW
Cells - Transformed fibroblasts	Skin	GTEX-ZV68-0008-SM-4YCCV
Cells - Transformed fibroblasts	Skin	GTEX-ZV6S-0008-SM-4YCF9
Cells - Transformed fibroblasts	Skin	GTEX-ZV7C-0008-SM-57WCL
Cells - Transformed fibroblasts	Skin	GTEX-ZVE1-0008-SM-51MS9
Cells - Transformed fibroblasts	Skin	GTEX-ZVE2-0008-SM-51MRU
Cells - Transformed fibroblasts	Skin	GTEX-ZVP2-0008-SM-51MSL
Cells - Transformed fibroblasts	Skin	GTEX-ZVT2-0008-SM-57WC9
Cells - Transformed fibroblasts	Skin	GTEX-ZVT3-0008-SM-51MRI
Cells - Transformed fibroblasts	Skin	GTEX-ZVT4-0008-SM-57WCA
Cells - Transformed fibroblasts	Skin	GTEX-ZVTK-0008-SM-57WDA
Cells - Transformed fibroblasts	Skin	GTEX-ZVZP-0008-SM-51MSX
Cells - Transformed fibroblasts	Skin	GTEX-ZVZQ-0008-SM-51MSK
Cells - Transformed fibroblasts	Skin	GTEX-ZXES-0008-SM-57WCX
Cells - Transformed fibroblasts	Skin	GTEX-ZXG5-0008-SM-57WDB
Cervix - Ectocervix	Cervix Uteri	GTEX-S32W-1526-SM-4AD6Z
Cervix - Ectocervix	Cervix Uteri	GTEX-S341-1126-SM-4AD6T
Cervix - Ectocervix	Cervix Uteri	GTEX-S4UY-1426-SM-4AD6Y
Cervix - Ectocervix	Cervix Uteri	GTEX-T5JW-0726-SM-4DM6D
Cervix - Ectocervix	Cervix Uteri	GTEX-TSE9-2826-SM-4DXTF
Cervix - Ectocervix	Cervix Uteri	GTEX-U3ZN-1626-SM-4DXTZ
Cervix - Endocervix	Cervix Uteri	GTEX-S32W-1626-SM-4AD6G
Cervix - Endocervix	Cervix Uteri	GTEX-S341-1326-SM-4AD72
Cervix - Endocervix	Cervix Uteri	GTEX-T6MO-1426-SM-4DM73
Cervix - Endocervix	Cervix Uteri	GTEX-TML8-0726-SM-4DXTT
Cervix - Endocervix	Cervix Uteri	GTEX-TSE9-2726-SM-4DXSQ
Colon - Sigmoid	Colon	GTEX-111CU-1226-SM-5EGIN
Colon - Sigmoid	Colon	GTEX-111YS-1826-SM-5GIEP
Colon - Sigmoid	Colon	GTEX-1122O-1426-SM-5H11R
Colon - Sigmoid	Colon	GTEX-117XS-2226-SM-5GIE1
Colon - Sigmoid	Colon	GTEX-117YX-0526-SM-5EGJH
Colon - Sigmoid	Colon	GTEX-11DXX-1926-SM-5EGJK
Colon - Sigmoid	Colon	GTEX-11DXY-2026-SM-5N9CZ
Colon - Sigmoid	Colon	GTEX-11DXZ-1626-SM-5GIDI
Colon - Sigmoid	Colon	GTEX-11EI6-2626-SM-5PNVT
Colon - Sigmoid	Colon	GTEX-11EM3-1626-SM-5N9CO
Colon - Sigmoid	Colon	GTEX-11EMC-1426-SM-5N9E4
Colon - Sigmoid	Colon	GTEX-11EQ9-1526-SM-5PNY7
Colon - Sigmoid	Colon	GTEX-11GS4-1826-SM-5HL4T
Colon - Sigmoid	Colon	GTEX-11I78-1326-SM-5PNY9
Colon - Sigmoid	Colon	GTEX-11ONC-1926-SM-5GU75
Colon - Sigmoid	Colon	GTEX-11P7K-1826-SM-5HL5A
Colon - Sigmoid	Colon	GTEX-11P81-1426-SM-5P9JN
Colon - Sigmoid	Colon	GTEX-11PRG-2026-SM-5GU5F
Colon - Sigmoid	Colon	GTEX-11TT1-0926-SM-5GU5M
Colon - Sigmoid	Colon	GTEX-11TUW-2326-SM-5EQMO
Colon - Sigmoid	Colon	GTEX-11VI4-1426-SM-5GU6O
Colon - Sigmoid	Colon	GTEX-11WQK-2526-SM-5CVL8
Colon - Sigmoid	Colon	GTEX-11ZTS-1526-SM-5N9G7
Colon - Sigmoid	Colon	GTEX-11ZTT-1626-SM-5EQKR
Colon - Sigmoid	Colon	GTEX-11ZU8-1126-SM-5EQ5K
Colon - Sigmoid	Colon	GTEX-1211K-1826-SM-5EGJ2
Colon - Sigmoid	Colon	GTEX-1212Z-1826-SM-5FQSH
Colon - Sigmoid	Colon	GTEX-12696-1926-SM-5EGJT

Colon - Sigmoid	Colon	GTEX-12BJ1-2026-SM-5FQUH
Colon - Sigmoid	Colon	GTEX-12KS4-2326-SM-5EGJB
Colon - Sigmoid	Colon	GTEX-12WSD-2626-SM-5EGJE
Colon - Sigmoid	Colon	GTEX-12WSG-2426-SM-5EQLZ
Colon - Sigmoid	Colon	GTEX-12WSJ-1426-SM-5GCON
Colon - Sigmoid	Colon	GTEX-12WSL-1826-SM-5LZW9
Colon - Sigmoid	Colon	GTEX-12WSM-1426-SM-5GCPA
Colon - Sigmoid	Colon	GTEX-12ZZW-2426-SM-5DUWE
Colon - Sigmoid	Colon	GTEX-12ZZX-2726-SM-5N9FT
Colon - Sigmoid	Colon	GTEX-12ZZY-2726-SM-5EQ45
Colon - Sigmoid	Colon	GTEX-13111-1926-SM-5GCOL
Colon - Sigmoid	Colon	GTEX-13113-1426-SM-5EGHU
Colon - Sigmoid	Colon	GTEX-131XE-1026-SM-5EGKE
Colon - Sigmoid	Colon	GTEX-131XG-1526-SM-5GCNQ
Colon - Sigmoid	Colon	GTEX-131YS-2926-SM-5EGKI
Colon - Sigmoid	Colon	GTEX-132AR-2826-SM-5IJFV
Colon - Sigmoid	Colon	GTEX-132NY-2726-SM-5PNY2
Colon - Sigmoid	Colon	GTEX-1339X-1726-SM-5P9J9
Colon - Sigmoid	Colon	GTEX-1399R-2126-SM-5K7WZ
Colon - Sigmoid	Colon	GTEX-1399U-2226-SM-5KLZI
Colon - Sigmoid	Colon	GTEX-139D8-2926-SM-5IJBM
Colon - Sigmoid	Colon	GTEX-139T6-1426-SM-5P9J7
Colon - Sigmoid	Colon	GTEX-139TS-2226-SM-5J2N1
Colon - Sigmoid	Colon	GTEX-139UW-2326-SM-5KM1Y
Colon - Sigmoid	Colon	GTEX-13CF2-1826-SM-5N9GK
Colon - Sigmoid	Colon	GTEX-13CF3-1326-SM-5LZXZ
Colon - Sigmoid	Colon	GTEX-13D11-0626-SM-5LZYY
Colon - Sigmoid	Colon	GTEX-13FH7-1626-SM-5J2O9
Colon - Sigmoid	Colon	GTEX-13FHO-2726-SM-5K7VG
Colon - Sigmoid	Colon	GTEX-13FTW-2126-SM-5K7YG
Colon - Sigmoid	Colon	GTEX-13G51-2126-SM-5IJD9
Colon - Sigmoid	Colon	GTEX-13N2G-2226-SM-5IJCU
Colon - Sigmoid	Colon	GTEX-13O1R-2426-SM-5KLZZ
Colon - Sigmoid	Colon	GTEX-13O61-1526-SM-5KM3P
Colon - Sigmoid	Colon	GTEX-13OVI-2126-SM-5KM3U
Colon - Sigmoid	Colon	GTEX-13OVJ-1826-SM-5KM2D
Colon - Sigmoid	Colon	GTEX-13OVK-1026-SM-7KFTC
Colon - Sigmoid	Colon	GTEX-13OW5-2826-SM-5KM1G
Colon - Sigmoid	Colon	GTEX-13OW6-2226-SM-5N9FL
Colon - Sigmoid	Colon	GTEX-13OW8-2726-SM-5L3FZ
Colon - Sigmoid	Colon	GTEX-13PL7-2426-SM-5J2N3
Colon - Sigmoid	Colon	GTEX-13PVQ-2726-SM-5L3H7
Colon - Sigmoid	Colon	GTEX-13PVR-1726-SM-5Q5EC
Colon - Sigmoid	Colon	GTEX-13QBU-2026-SM-5KLZB
Colon - Sigmoid	Colon	GTEX-13RTJ-1926-SM-5YYA2
Colon - Sigmoid	Colon	GTEX-13SLW-2826-SM-5Q5ES
Colon - Sigmoid	Colon	GTEX-13U4I-2626-SM-5SI9C
Colon - Sigmoid	Colon	GTEX-13VXT-2026-SM-5L3EW
Colon - Sigmoid	Colon	GTEX-13X6I-2726-SM-5Q5ET
Colon - Sigmoid	Colon	GTEX-13X6J-2826-SM-7EPGA
Colon - Sigmoid	Colon	GTEX-13X6K-2726-SM-5O9DG
Colon - Sigmoid	Colon	GTEX-144GM-1126-SM-79OJK
Colon - Sigmoid	Colon	GTEX-145LS-2926-SM-5O99G
Colon - Sigmoid	Colon	GTEX-145LT-0826-SM-5S2QL

Colon - Sigmoid	Colon	GTEX-145ME-1126-SM-5SIAT
Colon - Sigmoid	Colon	GTEX-145MH-2726-SM-5QGQ6
Colon - Sigmoid	Colon	GTEX-145MO-2626-SM-5QGPD
Colon - Sigmoid	Colon	GTEX-146FH-2426-SM-5Q5EW
Colon - Sigmoid	Colon	GTEX-1477Z-2126-SM-5Q5CM
Colon - Sigmoid	Colon	GTEX-147GR-2826-SM-5RQK8
Colon - Sigmoid	Colon	GTEX-147JS-2626-SM-5S2UX
Colon - Sigmoid	Colon	GTEX-148VJ-2426-SM-5NQ9Y
Colon - Sigmoid	Colon	GTEX-14A5H-1626-SM-5SIAN
Colon - Sigmoid	Colon	GTEX-14A5I-2426-SM-5Q5CP
Colon - Sigmoid	Colon	GTEX-14AS3-1026-SM-5TDD7
Colon - Sigmoid	Colon	GTEX-14BIN-2426-SM-5TDCF
Colon - Sigmoid	Colon	GTEX-14BMU-1326-SM-5RQIH
Colon - Sigmoid	Colon	GTEX-14C39-1626-SM-5S2P4
Colon - Sigmoid	Colon	GTEX-14C5O-2626-SM-5RQI5
Colon - Sigmoid	Colon	GTEX-14DAQ-2926-SM-664MV
Colon - Sigmoid	Colon	GTEX-14E1K-1626-SM-664MO
Colon - Sigmoid	Colon	GTEX-14E6C-2226-SM-5QGQG
Colon - Sigmoid	Colon	GTEX-14E6E-1726-SM-5S2R6
Colon - Sigmoid	Colon	GTEX-14JG6-1626-SM-5YYBC
Colon - Sigmoid	Colon	GTEX-14PHX-1126-SM-5YYA5
Colon - Sigmoid	Colon	GTEX-14PJ3-1226-SM-5ZZVY
Colon - Sigmoid	Colon	GTEX-14PJ4-1826-SM-5YY97
Colon - Sigmoid	Colon	GTEX-14PK6-1826-SM-69LOM
Colon - Sigmoid	Colon	GTEX-14PKV-1926-SM-686YU
Colon - Sigmoid	Colon	GTEX-14PN3-1926-SM-6EU1Y
Colon - Sigmoid	Colon	GTEX-15CHQ-2626-SM-686YZ
Colon - Sigmoid	Colon	GTEX-15DCZ-2826-SM-6EU2J
Colon - Sigmoid	Colon	GTEX-15DYW-2426-SM-7KUG8
Colon - Sigmoid	Colon	GTEX-15DZA-1026-SM-6PAND
Colon - Sigmoid	Colon	GTEX-15EO6-2326-SM-6LPKK
Colon - Sigmoid	Colon	GTEX-15ER7-2826-SM-6PAN2
Colon - Sigmoid	Colon	GTEX-15ETS-2326-SM-7KUGC
Colon - Sigmoid	Colon	GTEX-15F5U-1426-SM-7KUN2
Colon - Sigmoid	Colon	GTEX-15G19-2726-SM-6LPKG
Colon - Sigmoid	Colon	GTEX-15RIE-2026-SM-7KFRW
Colon - Sigmoid	Colon	GTEX-15RIF-1426-SM-7KUKQ
Colon - Sigmoid	Colon	GTEX-15RJE-2226-SM-6M46A
Colon - Sigmoid	Colon	GTEX-15UF6-2726-SM-7KULY
Colon - Sigmoid	Colon	GTEX-15UF7-1326-SM-7KUKV
Colon - Sigmoid	Colon	GTEX-16BQI-2026-SM-7KULQ
Colon - Sigmoid	Colon	GTEX-16MTA-1626-SM-6M47N
Colon - Sigmoid	Colon	GTEX-16NGA-0926-SM-718AG
Colon - Sigmoid	Colon	GTEX-16XZZ-2726-SM-7EWDO
Colon - Sigmoid	Colon	GTEX-16Z82-2426-SM-7KUMS
Colon - Sigmoid	Colon	GTEX-17EUY-1726-SM-79OK4
Colon - Sigmoid	Colon	GTEX-17F96-2126-SM-7KFSM
Colon - Sigmoid	Colon	GTEX-17F9Y-2626-SM-7IGN9
Colon - Sigmoid	Colon	GTEX-17GQL-1626-SM-7LG69
Colon - Sigmoid	Colon	GTEX-17HG3-1126-SM-7DUFC
Colon - Sigmoid	Colon	GTEX-17HGU-2626-SM-79OKN
Colon - Sigmoid	Colon	GTEX-17HHE-1626-SM-7DHLN
Colon - Sigmoid	Colon	GTEX-17JCI-2826-SM-7IGNA
Colon - Sigmoid	Colon	GTEX-17KNJ-1826-SM-7LT9N

Colon - Sigmoid	Colon	GTEX-17MFQ-1226-SM-7KFSA
Colon - Sigmoid	Colon	GTEX-183FY-1426-SM-7DHLM
Colon - Sigmoid	Colon	GTEX-183WM-2526-SM-731BP
Colon - Sigmoid	Colon	GTEX-18465-2726-SM-7LT9I
Colon - Sigmoid	Colon	GTEX-18A66-2626-SM-72D67
Colon - Sigmoid	Colon	GTEX-18A67-2226-SM-7LT9Z
Colon - Sigmoid	Colon	GTEX-18A6Q-2226-SM-7LG61
Colon - Sigmoid	Colon	GTEX-18D9A-1326-SM-7LT8P
Colon - Sigmoid	Colon	GTEX-18QFQ-1726-SM-731C9
Colon - Sigmoid	Colon	GTEX-1A32A-2626-SM-73KX9
Colon - Sigmoid	Colon	GTEX-1A3MV-1026-SM-72D5U
Colon - Sigmoid	Colon	GTEX-1A3MX-2326-SM-718B4
Colon - Sigmoid	Colon	GTEX-1AMFI-1826-SM-73KWB
Colon - Sigmoid	Colon	GTEX-1AX9J-2526-SM-73KUY
Colon - Sigmoid	Colon	GTEX-1AX9K-1626-SM-73KUS
Colon - Sigmoid	Colon	GTEX-1AYCT-1926-SM-793AE
Colon - Sigmoid	Colon	GTEX-1B8KE-1526-SM-7DUG2
Colon - Sigmoid	Colon	GTEX-1B8L1-1926-SM-7IGND
Colon - Sigmoid	Colon	GTEX-1B932-2126-SM-7IGMU
Colon - Sigmoid	Colon	GTEX-1B97I-1526-SM-73KUK
Colon - Sigmoid	Colon	GTEX-1B996-2726-SM-7DUGG
Colon - Sigmoid	Colon	GTEX-1BAJH-2726-SM-7IGNJ
Colon - Sigmoid	Colon	GTEX-1C2JI-1726-SM-73KUW
Colon - Sigmoid	Colon	GTEX-1C475-1526-SM-7DHM3
Colon - Sigmoid	Colon	GTEX-1C4CL-1626-SM-7DUF3
Colon - Sigmoid	Colon	GTEX-1C6VQ-2626-SM-7IGMQ
Colon - Sigmoid	Colon	GTEX-1CB4F-1926-SM-7MKFN
Colon - Sigmoid	Colon	GTEX-1CB4G-2726-SM-79OO4
Colon - Sigmoid	Colon	GTEX-1E2YA-2526-SM-7IGOU
Colon - Sigmoid	Colon	GTEX-1EMGI-2626-SM-7IGNR
Colon - Sigmoid	Colon	GTEX-V955-1726-SM-4JBHF
Colon - Sigmoid	Colon	GTEX-WFG7-1626-SM-4LVMF
Colon - Sigmoid	Colon	GTEX-WFG8-1726-SM-4LVM6
Colon - Sigmoid	Colon	GTEX-WFON-1326-SM-4LVMN
Colon - Sigmoid	Colon	GTEX-WHPG-1626-SM-4M1ZL
Colon - Sigmoid	Colon	GTEX-WHWD-1526-SM-4OORV
Colon - Sigmoid	Colon	GTEX-WI4N-2226-SM-4OOS9
Colon - Sigmoid	Colon	GTEX-WQUQ-2626-SM-4MVNP
Colon - Sigmoid	Colon	GTEX-WRHK-1126-SM-4MVOJ
Colon - Sigmoid	Colon	GTEX-WXYG-1726-SM-4ONCU
Colon - Sigmoid	Colon	GTEX-WY7C-1926-SM-4ONCI
Colon - Sigmoid	Colon	GTEX-WYVS-1126-SM-4SOJX
Colon - Sigmoid	Colon	GTEX-X15G-1526-SM-4PQZM
Colon - Sigmoid	Colon	GTEX-X4EO-2726-SM-4E3HS
Colon - Sigmoid	Colon	GTEX-XAJ8-0726-SM-47JY5
Colon - Sigmoid	Colon	GTEX-XBED-1726-SM-47JYO
Colon - Sigmoid	Colon	GTEX-XQ8I-2326-SM-4BOQC
Colon - Sigmoid	Colon	GTEX-XUW1-1826-SM-4BOQD
Colon - Sigmoid	Colon	GTEX-XUZC-1526-SM-4BRV4
Colon - Sigmoid	Colon	GTEX-XV7Q-2226-SM-4BRVY
Colon - Sigmoid	Colon	GTEX-XXEK-1826-SM-4BRVC
Colon - Sigmoid	Colon	GTEX-Y114-1626-SM-4TT7I
Colon - Sigmoid	Colon	GTEX-Y3I4-1126-SM-4TT7Y
Colon - Sigmoid	Colon	GTEX-Y5LM-1426-SM-5RQJL

Colon - Sigmoid	Colon	GTEX-Y8LW-1126-SM-4VDS4
Colon - Sigmoid	Colon	GTEX-Y9LG-1326-SM-4VBQB
Colon - Sigmoid	Colon	GTEX-YEC3-2026-SM-4WWFL
Colon - Sigmoid	Colon	GTEX-YECK-2826-SM-5IFHF
Colon - Sigmoid	Colon	GTEX-YF7O-1626-SM-5IFII
Colon - Sigmoid	Colon	GTEX-YFCO-1526-SM-5YYB8
Colon - Sigmoid	Colon	GTEX-YJ8A-1726-SM-5P9IQ
Colon - Sigmoid	Colon	GTEX-YJ8O-1426-SM-5PNV9
Colon - Sigmoid	Colon	GTEX-Z93S-2626-SM-57WBX
Colon - Sigmoid	Colon	GTEX-ZA64-1426-SM-5CVMC
Colon - Sigmoid	Colon	GTEX-ZAB4-2426-SM-5HL8A
Colon - Sigmoid	Colon	GTEX-ZAB5-1426-SM-5HL9D
Colon - Sigmoid	Colon	GTEX-ZAJG-2626-SM-5S2NU
Colon - Sigmoid	Colon	GTEX-ZC5H-2426-SM-4WAZ6
Colon - Sigmoid	Colon	GTEX-ZDTS-2726-SM-5L3E2
Colon - Sigmoid	Colon	GTEX-ZE7O-2626-SM-51MSS
Colon - Sigmoid	Colon	GTEX-ZE9C-2126-SM-4WKGY
Colon - Sigmoid	Colon	GTEX-ZF28-2526-SM-57WFH
Colon - Sigmoid	Colon	GTEX-ZF29-1826-SM-4WKG8
Colon - Sigmoid	Colon	GTEX-ZF2S-1826-SM-4WKFF
Colon - Sigmoid	Colon	GTEX-ZG7Y-1826-SM-4WWD3
Colon - Sigmoid	Colon	GTEX-ZLFU-1426-SM-4WWEU
Colon - Sigmoid	Colon	GTEX-ZLV1-1326-SM-4WWER
Colon - Sigmoid	Colon	GTEX-ZP4G-1526-SM-57WE5
Colon - Sigmoid	Colon	GTEX-ZPCL-1626-SM-57WGC
Colon - Sigmoid	Colon	GTEX-ZPIC-2626-SM-57WE2
Colon - Sigmoid	Colon	GTEX-ZPU1-1726-SM-4WWFV
Colon - Sigmoid	Colon	GTEX-ZQG8-2426-SM-57WEE
Colon - Sigmoid	Colon	GTEX-ZT9W-1726-SM-51MS3
Colon - Sigmoid	Colon	GTEX-ZTSS-1226-SM-51MSZ
Colon - Sigmoid	Colon	GTEX-ZTTD-2626-SM-57WEX
Colon - Sigmoid	Colon	GTEX-ZV6S-1126-SM-57WE9
Colon - Sigmoid	Colon	GTEX-ZVT2-1726-SM-51MRO
Colon - Sigmoid	Colon	GTEX-ZVT3-2826-SM-5GU6A
Colon - Sigmoid	Colon	GTEX-ZVT4-2626-SM-5N9GJ
Colon - Sigmoid	Colon	GTEX-ZVZQ-2626-SM-59HLB
Colon - Sigmoid	Colon	GTEX-ZXES-1726-SM-5E43G
Colon - Sigmoid	Colon	GTEX-ZY6K-1326-SM-5GZWP
Colon - Sigmoid	Colon	GTEX-ZYFD-2226-SM-5E43P
Colon - Sigmoid	Colon	GTEX-ZYFG-1826-SM-5GZWX
Colon - Sigmoid	Colon	GTEX-ZYT6-2826-SM-5GICX
Colon - Sigmoid	Colon	GTEX-ZYY3-2226-SM-5E45A
Colon - Sigmoid	Colon	GTEX-ZZ64-0826-SM-5E449
Colon - Transverse	Colon	GTEX-111CU-1426-SM-5GZYP
Colon - Transverse	Colon	GTEX-111VG-2226-SM-5N9DU
Colon - Transverse	Colon	GTEX-111YS-1626-SM-5GZZ9
Colon - Transverse	Colon	GTEX-1122O-1526-SM-5N9CL
Colon - Transverse	Colon	GTEX-1128S-1626-SM-5H12O
Colon - Transverse	Colon	GTEX-117YW-1826-SM-5PNY5
Colon - Transverse	Colon	GTEX-117YX-0826-SM-5H11J
Colon - Transverse	Colon	GTEX-11DXX-1826-SM-5H126
Colon - Transverse	Colon	GTEX-11DXZ-1726-SM-5H12M
Colon - Transverse	Colon	GTEX-11EM3-1526-SM-5A5KN
Colon - Transverse	Colon	GTEX-11EQ9-1426-SM-5987G

Colon - Transverse	Colon	GTEX-11GSP-1626-SM-5986N
Colon - Transverse	Colon	GTEX-11I78-1226-SM-5A5K6
Colon - Transverse	Colon	GTEX-11LCK-2026-SM-5A5MA
Colon - Transverse	Colon	GTEX-11NSD-1626-SM-5986U
Colon - Transverse	Colon	GTEX-11O72-2226-SM-5PNW2
Colon - Transverse	Colon	GTEX-11ONC-2026-SM-5HL61
Colon - Transverse	Colon	GTEX-11P7K-1526-SM-5EGI7
Colon - Transverse	Colon	GTEX-11P81-1126-SM-5GU5G
Colon - Transverse	Colon	GTEX-11P82-1026-SM-5BC5J
Colon - Transverse	Colon	GTEX-11TT1-1026-SM-5PNW7
Colon - Transverse	Colon	GTEX-11TTK-2426-SM-5PNYO
Colon - Transverse	Colon	GTEX-11TUW-2026-SM-5EQL8
Colon - Transverse	Colon	GTEX-11VI4-1526-SM-5EQKC
Colon - Transverse	Colon	GTEX-11WQK-2126-SM-5EQME
Colon - Transverse	Colon	GTEX-11ZVC-2126-SM-5EGJ1
Colon - Transverse	Colon	GTEX-1211K-1726-SM-5FQUJ
Colon - Transverse	Colon	GTEX-12BJ1-2126-SM-5BC5O
Colon - Transverse	Colon	GTEX-12C56-0626-SM-5EGGC
Colon - Transverse	Colon	GTEX-12KS4-2426-SM-5LU8P
Colon - Transverse	Colon	GTEX-12WSJ-1526-SM-5GCNI
Colon - Transverse	Colon	GTEX-12WSL-1626-SM-5GCMU
Colon - Transverse	Colon	GTEX-12ZZZ-2426-SM-5GCNN
Colon - Transverse	Colon	GTEX-13111-0726-SM-5DUVE
Colon - Transverse	Colon	GTEX-131XE-0826-SM-5HL9W
Colon - Transverse	Colon	GTEX-131XG-1826-SM-5LZV4
Colon - Transverse	Colon	GTEX-132AR-2726-SM-5PNVN
Colon - Transverse	Colon	GTEX-1339X-1626-SM-5IJDN
Colon - Transverse	Colon	GTEX-133LE-1526-SM-5IFEN
Colon - Transverse	Colon	GTEX-1399R-2226-SM-5P9JB
Colon - Transverse	Colon	GTEX-1399S-1926-SM-5J2M5
Colon - Transverse	Colon	GTEX-1399U-1926-SM-5IFH7
Colon - Transverse	Colon	GTEX-139T6-1326-SM-5IFFO
Colon - Transverse	Colon	GTEX-139TS-1926-SM-5IJFO
Colon - Transverse	Colon	GTEX-139YR-2126-SM-5KM11
Colon - Transverse	Colon	GTEX-13CF3-1426-SM-5K7YY
Colon - Transverse	Colon	GTEX-13D11-0826-SM-5KLZJ
Colon - Transverse	Colon	GTEX-13FH7-1526-SM-5J2N6
Colon - Transverse	Colon	GTEX-13FHP-2426-SM-5KLZ5
Colon - Transverse	Colon	GTEX-13FTW-1826-SM-5IJEN
Colon - Transverse	Colon	GTEX-13O21-2426-SM-5MR4A
Colon - Transverse	Colon	GTEX-13O3O-2526-SM-5L3DR
Colon - Transverse	Colon	GTEX-13O3P-2326-SM-5KM55
Colon - Transverse	Colon	GTEX-13O61-1626-SM-5KM3T
Colon - Transverse	Colon	GTEX-13OVG-1426-SM-5K7VP
Colon - Transverse	Colon	GTEX-13OVI-2026-SM-5J1MU
Colon - Transverse	Colon	GTEX-13OVJ-1726-SM-5IJDZ
Colon - Transverse	Colon	GTEX-13OVK-0826-SM-7KULZ
Colon - Transverse	Colon	GTEX-13OW5-2626-SM-5MR3M
Colon - Transverse	Colon	GTEX-13OW6-2126-SM-5N9GL
Colon - Transverse	Colon	GTEX-13PL7-2526-SM-5IFGR
Colon - Transverse	Colon	GTEX-13PVR-1626-SM-73KVF
Colon - Transverse	Colon	GTEX-13QBU-1826-SM-5IJG3
Colon - Transverse	Colon	GTEX-13S86-1626-SM-5Q5C5
Colon - Transverse	Colon	GTEX-13U4I-2426-SM-5LU34

Colon - Transverse	Colon	GTEX-13VXT-2426-SM-5LU46
Colon - Transverse	Colon	GTEX-13W3W-2126-SM-5K7UX
Colon - Transverse	Colon	GTEX-13X6H-1926-SM-7EWD3
Colon - Transverse	Colon	GTEX-144GL-2526-SM-5TDDL
Colon - Transverse	Colon	GTEX-144GM-1226-SM-5LU52
Colon - Transverse	Colon	GTEX-144GN-1126-SM-5LU33
Colon - Transverse	Colon	GTEX-145LS-2726-SM-5Q5D8
Colon - Transverse	Colon	GTEX-145LT-0926-SM-5LU9C
Colon - Transverse	Colon	GTEX-145ME-0826-SM-5O9AF
Colon - Transverse	Colon	GTEX-145MN-1726-SM-5SIA6
Colon - Transverse	Colon	GTEX-145MO-2426-SM-5NQ9W
Colon - Transverse	Colon	GTEX-146FQ-1926-SM-5NQBY
Colon - Transverse	Colon	GTEX-147F3-1926-SM-5SIB9
Colon - Transverse	Colon	GTEX-147GR-2726-SM-664OO
Colon - Transverse	Colon	GTEX-147JS-2526-SM-5S2Q8
Colon - Transverse	Colon	GTEX-148VJ-2626-SM-5QGPI
Colon - Transverse	Colon	GTEX-14AS3-1126-SM-5Q5CR
Colon - Transverse	Colon	GTEX-14BIN-3026-SM-5ZZUS
Colon - Transverse	Colon	GTEX-14BMU-1526-SM-5TDE6
Colon - Transverse	Colon	GTEX-14BMV-2426-SM-5S2PP
Colon - Transverse	Colon	GTEX-14C39-1926-SM-5ZZW5
Colon - Transverse	Colon	GTEX-14C5O-2526-SM-73KU2
Colon - Transverse	Colon	GTEX-14DAQ-2726-SM-5S2QY
Colon - Transverse	Colon	GTEX-14DAR-1526-SM-5RQIX
Colon - Transverse	Colon	GTEX-14E1K-1826-SM-793DT
Colon - Transverse	Colon	GTEX-14E6E-1526-SM-5RQIG
Colon - Transverse	Colon	GTEX-14H4A-1926-SM-5ZZWN
Colon - Transverse	Colon	GTEX-14ICK-1026-SM-6AJA4
Colon - Transverse	Colon	GTEX-14ICL-1226-SM-5S2RD
Colon - Transverse	Colon	GTEX-14JG6-1726-SM-69LPL
Colon - Transverse	Colon	GTEX-14JIY-3326-SM-6871E
Colon - Transverse	Colon	GTEX-14PHX-1526-SM-68717
Colon - Transverse	Colon	GTEX-14PJ3-0926-SM-69LQG
Colon - Transverse	Colon	GTEX-14PJ4-1926-SM-6LLHG
Colon - Transverse	Colon	GTEX-14PJ5-0926-SM-6LLIK
Colon - Transverse	Colon	GTEX-14PJM-2626-SM-686ZD
Colon - Transverse	Colon	GTEX-14PK6-1726-SM-6871J
Colon - Transverse	Colon	GTEX-14PKV-2126-SM-664O3
Colon - Transverse	Colon	GTEX-14XAO-2026-SM-69LOI
Colon - Transverse	Colon	GTEX-15CHC-1626-SM-5ZZWS
Colon - Transverse	Colon	GTEX-15CHQ-2526-SM-6EU24
Colon - Transverse	Colon	GTEX-15CHR-1426-SM-7DUGV
Colon - Transverse	Colon	GTEX-15DCE-2026-SM-7KUFE
Colon - Transverse	Colon	GTEX-15DYW-2326-SM-6PAMU
Colon - Transverse	Colon	GTEX-15DZA-0926-SM-69LQ3
Colon - Transverse	Colon	GTEX-15ER7-2926-SM-7KUFK
Colon - Transverse	Colon	GTEX-15G1A-1826-SM-7KUKO
Colon - Transverse	Colon	GTEX-15RIE-1826-SM-6M48P
Colon - Transverse	Colon	GTEX-15RIF-1326-SM-6LPKO
Colon - Transverse	Colon	GTEX-15SB6-1226-SM-7KUMP
Colon - Transverse	Colon	GTEX-15SHU-2526-SM-6M47D
Colon - Transverse	Colon	GTEX-15UF6-2626-SM-6M466
Colon - Transverse	Colon	GTEX-16AAH-1326-SM-7KFRG
Colon - Transverse	Colon	GTEX-16MT8-2726-SM-7IGLJ

Colon - Transverse	Colon	GTEX-16NGA-1026-SM-7KFS3
Colon - Transverse	Colon	GTEX-16NPV-2626-SM-6M484
Colon - Transverse	Colon	GTEX-16Z82-2226-SM-6LPJ7
Colon - Transverse	Colon	GTEX-178AV-1326-SM-6LPJX
Colon - Transverse	Colon	GTEX-17EUY-1826-SM-7DUEZ
Colon - Transverse	Colon	GTEX-17EVQ-1826-SM-7IGPH
Colon - Transverse	Colon	GTEX-17F96-2026-SM-79ON4
Colon - Transverse	Colon	GTEX-17F98-1026-SM-7IGO8
Colon - Transverse	Colon	GTEX-17F9E-1826-SM-7IGMC
Colon - Transverse	Colon	GTEX-17GQL-1526-SM-7LTAC
Colon - Transverse	Colon	GTEX-17HGU-2426-SM-7EWDP
Colon - Transverse	Colon	GTEX-17HHE-1826-SM-79OKE
Colon - Transverse	Colon	GTEX-17JCI-2626-SM-7EWE6
Colon - Transverse	Colon	GTEX-17KNJ-1726-SM-7LG6A
Colon - Transverse	Colon	GTEX-17MF6-2426-SM-7LTAM
Colon - Transverse	Colon	GTEX-18A66-2426-SM-7KFSQ
Colon - Transverse	Colon	GTEX-18A7A-2226-SM-7LT8K
Colon - Transverse	Colon	GTEX-18A7B-2026-SM-72D71
Colon - Transverse	Colon	GTEX-18D9A-1126-SM-7KFRJ
Colon - Transverse	Colon	GTEX-18D9B-2526-SM-718BN
Colon - Transverse	Colon	GTEX-18D9U-1926-SM-7KFTM
Colon - Transverse	Colon	GTEX-18QFQ-1526-SM-72D57
Colon - Transverse	Colon	GTEX-1A3MV-1126-SM-718BS
Colon - Transverse	Colon	GTEX-1AMEY-1926-SM-73KVT
Colon - Transverse	Colon	GTEX-1AMFI-1726-SM-7189L
Colon - Transverse	Colon	GTEX-1AX9I-2626-SM-73KU5
Colon - Transverse	Colon	GTEX-1AYCT-1826-SM-731EG
Colon - Transverse	Colon	GTEX-1B8KE-1926-SM-731EE
Colon - Transverse	Colon	GTEX-1B8KZ-1326-SM-7DUG6
Colon - Transverse	Colon	GTEX-1B932-2826-SM-73KUD
Colon - Transverse	Colon	GTEX-1B996-2626-SM-73KZ1
Colon - Transverse	Colon	GTEX-1BAJH-2326-SM-7IGMO
Colon - Transverse	Colon	GTEX-1C2JI-1626-SM-7EPHC
Colon - Transverse	Colon	GTEX-1C475-1626-SM-731FP
Colon - Transverse	Colon	GTEX-1C4CL-2126-SM-7IGQC
Colon - Transverse	Colon	GTEX-1C64O-2326-SM-73KU8
Colon - Transverse	Colon	GTEX-1C6VQ-2726-SM-73KUL
Colon - Transverse	Colon	GTEX-1CAMS-2526-SM-7EPH7
Colon - Transverse	Colon	GTEX-1CB4F-2026-SM-79OLU
Colon - Transverse	Colon	GTEX-NFK9-2026-SM-3LK5K
Colon - Transverse	Colon	GTEX-O5YT-1426-SM-3MJHC
Colon - Transverse	Colon	GTEX-O5YW-1426-SM-3MJHF
Colon - Transverse	Colon	GTEX-OHPM-1426-SM-3TW8Y
Colon - Transverse	Colon	GTEX-OIZH-1426-SM-3NB1O
Colon - Transverse	Colon	GTEX-OXRK-1726-SM-3NB16
Colon - Transverse	Colon	GTEX-OXRL-1426-SM-3NM9E
Colon - Transverse	Colon	GTEX-P4PP-1426-SM-3NM9L
Colon - Transverse	Colon	GTEX-P4QT-1426-SM-3NMCX
Colon - Transverse	Colon	GTEX-P78B-1726-SM-3P5ZV
Colon - Transverse	Colon	GTEX-PLZ5-1126-SM-3P613
Colon - Transverse	Colon	GTEX-PLZ6-0926-SM-3P5ZQ
Colon - Transverse	Colon	GTEX-PWCY-1026-SM-48TD4
Colon - Transverse	Colon	GTEX-PWN1-1426-SM-48TDF
Colon - Transverse	Colon	GTEX-PX3G-1426-SM-48U1J

Colon - Transverse	Colon	GTEX-Q2AH-1226-SM-48TZL
Colon - Transverse	Colon	GTEX-Q2AI-0926-SM-48U1F
Colon - Transverse	Colon	GTEX-Q734-1126-SM-48TZY
Colon - Transverse	Colon	GTEX-QCQG-1626-SM-48U26
Colon - Transverse	Colon	GTEX-QDVJ-1326-SM-48U1X
Colon - Transverse	Colon	GTEX-QDVN-1326-SM-48TZ3
Colon - Transverse	Colon	GTEX-QEL4-1926-SM-4R1JI
Colon - Transverse	Colon	GTEX-QLQ7-1126-SM-4R1KN
Colon - Transverse	Colon	GTEX-QLQW-0426-SM-447A7
Colon - Transverse	Colon	GTEX-QMRM-1226-SM-447C6
Colon - Transverse	Colon	GTEX-QV31-0826-SM-4R1KR
Colon - Transverse	Colon	GTEX-R53T-1326-SM-48FCQ
Colon - Transverse	Colon	GTEX-R55C-1126-SM-48FCJ
Colon - Transverse	Colon	GTEX-R55D-1826-SM-48FEF
Colon - Transverse	Colon	GTEX-R55G-1226-SM-48FDC
Colon - Transverse	Colon	GTEX-RM2N-0926-SM-48FD1
Colon - Transverse	Colon	GTEX-RU1J-1326-SM-46MUL
Colon - Transverse	Colon	GTEX-RWS6-1826-SM-47JXX
Colon - Transverse	Colon	GTEX-S341-1426-SM-4AD6U
Colon - Transverse	Colon	GTEX-S3XE-1126-SM-4AD4N
Colon - Transverse	Colon	GTEX-S4P3-1226-SM-4AD4Y
Colon - Transverse	Colon	GTEX-S4Q7-0826-SM-4AD5E
Colon - Transverse	Colon	GTEX-S4UY-0826-SM-4AD4Z
Colon - Transverse	Colon	GTEX-S7SF-1926-SM-4AT5B
Colon - Transverse	Colon	GTEX-SE5C-1526-SM-4BRWU
Colon - Transverse	Colon	GTEX-SNMC-1126-SM-4DM5M
Colon - Transverse	Colon	GTEX-SNOS-1226-SM-4DM5H
Colon - Transverse	Colon	GTEX-T5JW-1126-SM-4DM5V
Colon - Transverse	Colon	GTEX-T6MO-0826-SM-4DM51
Colon - Transverse	Colon	GTEX-TKQ1-0626-SM-4DXTS
Colon - Transverse	Colon	GTEX-TKQ2-1326-SM-4DXT9
Colon - Transverse	Colon	GTEX-TML8-1326-SM-4DXTO
Colon - Transverse	Colon	GTEX-U3ZH-1326-SM-4DXSF
Colon - Transverse	Colon	GTEX-U3ZM-1126-SM-4DXUB
Colon - Transverse	Colon	GTEX-U3ZN-2126-SM-4DXU1
Colon - Transverse	Colon	GTEX-U4B1-1126-SM-4DXV3
Colon - Transverse	Colon	GTEX-UJHI-1126-SM-4IHLN
Colon - Transverse	Colon	GTEX-UJMC-1326-SM-4IHLS
Colon - Transverse	Colon	GTEX-V955-1626-SM-4JBHJ
Colon - Transverse	Colon	GTEX-VJYA-2026-SM-4KL1K
Colon - Transverse	Colon	GTEX-W5WG-2426-SM-4LMI6
Colon - Transverse	Colon	GTEX-WEY5-1426-SM-4LMJ3
Colon - Transverse	Colon	GTEX-WFG7-1526-SM-4LVMG
Colon - Transverse	Colon	GTEX-WFON-1426-SM-4LVMT
Colon - Transverse	Colon	GTEX-WH7G-1326-SM-4LVMS
Colon - Transverse	Colon	GTEX-WHSB-0926-SM-4M1XJ
Colon - Transverse	Colon	GTEX-WHWD-1226-SM-4OOS1
Colon - Transverse	Colon	GTEX-WOFM-2026-SM-4OOSQ
Colon - Transverse	Colon	GTEX-WQUQ-2526-SM-4MVNO
Colon - Transverse	Colon	GTEX-WY7C-1626-SM-4ONC8
Colon - Transverse	Colon	GTEX-WYJK-2226-SM-4ONDJ
Colon - Transverse	Colon	GTEX-WYVS-1826-SM-4OND6
Colon - Transverse	Colon	GTEX-X15G-1326-SM-4PQZJ
Colon - Transverse	Colon	GTEX-X3Y1-1726-SM-4PQZL

Colon - Transverse	Colon	GTEX-X5EB-0926-SM-46MVT
Colon - Transverse	Colon	GTEX-XBED-1526-SM-4AT5W
Colon - Transverse	Colon	GTEX-XBEW-1926-SM-4SOJO
Colon - Transverse	Colon	GTEX-XGQ4-1226-SM-4AT67
Colon - Transverse	Colon	GTEX-XMK1-1726-SM-4B64Z
Colon - Transverse	Colon	GTEX-XPVG-1826-SM-4B64X
Colon - Transverse	Colon	GTEX-XQ8I-2126-SM-4BOOM
Colon - Transverse	Colon	GTEX-XUW1-1926-SM-4BOP1
Colon - Transverse	Colon	GTEX-XUZC-1326-SM-4BRV2
Colon - Transverse	Colon	GTEX-XV7Q-2126-SM-4BRVX
Colon - Transverse	Colon	GTEX-XXEK-1926-SM-4BRVD
Colon - Transverse	Colon	GTEX-XYKS-2226-SM-4E3IU
Colon - Transverse	Colon	GTEX-Y114-1526-SM-4TT79
Colon - Transverse	Colon	GTEX-Y3I4-1426-SM-4TT9C
Colon - Transverse	Colon	GTEX-Y3IK-1826-SM-4WWDS
Colon - Transverse	Colon	GTEX-Y5LM-1526-SM-4VDTA
Colon - Transverse	Colon	GTEX-Y8E4-2426-SM-4WWDN
Colon - Transverse	Colon	GTEX-Y8LW-1226-SM-4WWDP
Colon - Transverse	Colon	GTEX-Y9LG-1526-SM-4WWDR
Colon - Transverse	Colon	GTEX-YB5E-1626-SM-5IFIQ
Colon - Transverse	Colon	GTEX-YEC3-2426-SM-5IFJR
Colon - Transverse	Colon	GTEX-YEC4-1926-SM-5IFHJ
Colon - Transverse	Colon	GTEX-YF7O-1726-SM-5IFJ9
Colon - Transverse	Colon	GTEX-Z93S-2426-SM-5HL8N
Colon - Transverse	Colon	GTEX-ZAB4-2226-SM-5HL97
Colon - Transverse	Colon	GTEX-ZDYS-2326-SM-4WKG6
Colon - Transverse	Colon	GTEX-ZEX8-1926-SM-4WKFR
Colon - Transverse	Colon	GTEX-ZF29-1726-SM-4WKFV
Colon - Transverse	Colon	GTEX-ZF2S-1926-SM-4WWAN
Colon - Transverse	Colon	GTEX-ZGAY-2126-SM-4YCFB
Colon - Transverse	Colon	GTEX-ZLV1-1226-SM-4WWBX
Colon - Transverse	Colon	GTEX-ZP4G-1726-SM-4WWCM
Colon - Transverse	Colon	GTEX-ZPCL-1826-SM-57WF1
Colon - Transverse	Colon	GTEX-ZPIC-2226-SM-57WDX
Colon - Transverse	Colon	GTEX-ZPU1-1926-SM-57WDT
Colon - Transverse	Colon	GTEX-ZT9W-1826-SM-51MQX
Colon - Transverse	Colon	GTEX-ZTSS-1126-SM-5985O
Colon - Transverse	Colon	GTEX-ZV6S-1226-SM-59HLH
Colon - Transverse	Colon	GTEX-ZV7C-1526-SM-5NQ72
Colon - Transverse	Colon	GTEX-ZVP2-1526-SM-5NQ82
Colon - Transverse	Colon	GTEX-ZVT2-1926-SM-5GU5T
Colon - Transverse	Colon	GTEX-ZXES-1626-SM-5NQ7L
Colon - Transverse	Colon	GTEX-ZY6K-1226-SM-5GZYL
Colon - Transverse	Colon	GTEX-ZYFC-2626-SM-5NQ6S
Colon - Transverse	Colon	GTEX-ZYFG-1926-SM-5GID5
Colon - Transverse	Colon	GTEX-ZYVF-2826-SM-5GIDK
Colon - Transverse	Colon	GTEX-ZZ64-0726-SM-5GZX4
Esophagus - Gastroesophageal Junction	Esophagus
GTEX-111CU-0826-SM-5EGIJ
Esophagus - Gastroesophageal Junction	Esophagus
GTEX-111YS-0826-SM-5GZYK
Esophagus - Gastroesophageal Junction	Esophagus
GTEX-1122O-1826-SM-5EGIP
Esophagus - Gastroesophageal Junction	Esophagus

GTEX-113JC-1026-SM-5H117
Esophagus - Gastroesophageal Junction	Esophagus
GTEX-1192X-1826-SM-5GIE2
Esophagus - Gastroesophageal Junction	Esophagus
GTEX-11DXX-1226-SM-5GICD
Esophagus - Gastroesophageal Junction	Esophagus
GTEX-11DXY-1326-SM-5987Z
Esophagus - Gastroesophageal Junction	Esophagus
GTEX-11DXZ-1026-SM-5N9D5
Esophagus - Gastroesophageal Junction	Esophagus
GTEX-11DYG-1926-SM-5H12I
Esophagus - Gastroesophageal Junction	Esophagus
GTEX-11EM3-0926-SM-5A5KK
Esophagus - Gastroesophageal Junction	Esophagus
GTEX-11EQ9-0926-SM-5HL6I
Esophagus - Gastroesophageal Junction	Esophagus
GTEX-11LCK-1526-SM-5HL64
Esophagus - Gastroesophageal Junction	Esophagus
GTEX-11NUK-1726-SM-5A5L2
Esophagus - Gastroesophageal Junction	Esophagus
GTEX-11O72-2026-SM-5N9G8
Esophagus - Gastroesophageal Junction	Esophagus
GTEX-11OF3-2326-SM-5GU73
Esophagus - Gastroesophageal Junction	Esophagus
GTEX-11P7K-1426-SM-5EGKY
Esophagus - Gastroesophageal Junction	Esophagus
GTEX-11P81-1026-SM-5GU54
Esophagus - Gastroesophageal Junction	Esophagus
GTEX-11P82-0426-SM-5HL5D
Esophagus - Gastroesophageal Junction	Esophagus
GTEX-11TT1-0626-SM-5GU4X
Esophagus - Gastroesophageal Junction	Esophagus
GTEX-11UD2-1526-SM-5EQLQ
Esophagus - Gastroesophageal Junction	Esophagus
GTEX-11VI4-0826-SM-5GU5N
Esophagus - Gastroesophageal Junction	Esophagus
GTEX-11WQK-2226-SM-5CVL7
Esophagus - Gastroesophageal Junction	Esophagus
GTEX-11XUK-0926-SM-5EQL3
Esophagus - Gastroesophageal Junction	Esophagus
GTEX-11ZTS-2726-SM-5EQLU
Esophagus - Gastroesophageal Junction	Esophagus
GTEX-1211K-1326-SM-5FQV2
Esophagus - Gastroesophageal Junction	Esophagus
GTEX-12696-1526-SM-5EGL6
Esophagus - Gastroesophageal Junction	Esophagus
GTEX-12BJ1-1526-SM-5LUAQ
Esophagus - Gastroesophageal Junction	Esophagus
GTEX-12C56-0426-SM-5FQU1
Esophagus - Gastroesophageal Junction	Esophagus
GTEX-12WSD-2026-SM-5HL7D
Esophagus - Gastroesophageal Junction	Esophagus
GTEX-12WSJ-0826-SM-5PNWV
Esophagus - Gastroesophageal Junction	Esophagus

GTEX-12WSK-1326-SM-5CVNM
Esophagus - Gastroesophageal Junction Esophagus
GTEX-12WSL-1226-SM-5DUXE
Esophagus - Gastroesophageal Junction Esophagus
GTEX-12WSN-1826-SM-5LZUT
Esophagus - Gastroesophageal Junction Esophagus
GTEX-13111-1126-SM-5GCMZ
Esophagus - Gastroesophageal Junction Esophagus
GTEX-131XE-1526-SM-5KM1A
Esophagus - Gastroesophageal Junction Esophagus
GTEX-131XF-1126-SM-5HL8J
Esophagus - Gastroesophageal Junction Esophagus
GTEX-131XG-1126-SM-5DUWR
Esophagus - Gastroesophageal Junction Esophagus
GTEX-132AR-2526-SM-5PNVM
Esophagus - Gastroesophageal Junction Esophagus
GTEX-132NY-2026-SM-5L3FM
Esophagus - Gastroesophageal Junction Esophagus
GTEX-132QS-1626-SM-5K7YZ
Esophagus - Gastroesophageal Junction Esophagus
GTEX-1339X-1126-SM-5J2OI
Esophagus - Gastroesophageal Junction Esophagus
GTEX-1399R-0926-SM-5J2N8
Esophagus - Gastroesophageal Junction Esophagus
GTEX-1399S-1526-SM-5P9JC
Esophagus - Gastroesophageal Junction Esophagus
GTEX-1399U-1426-SM-5J1N8
Esophagus - Gastroesophageal Junction Esophagus
GTEX-139T6-0826-SM-5J1O2
Esophagus - Gastroesophageal Junction Esophagus
GTEX-139YR-1826-SM-5LZXA
Esophagus - Gastroesophageal Junction Esophagus
GTEX-13CF3-1726-SM-5LU9E
Esophagus - Gastroesophageal Junction Esophagus
GTEX-13D11-2026-SM-5IJBK
Esophagus - Gastroesophageal Junction Esophagus
GTEX-13FH7-0626-SM-5KLZD
Esophagus - Gastroesophageal Junction Esophagus
GTEX-13FLV-1326-SM-5L3EJ
Esophagus - Gastroesophageal Junction Esophagus
GTEX-13FTW-1926-SM-5K7Y4
Esophagus - Gastroesophageal Junction Esophagus
GTEX-13N1W-1926-SM-5IJCH
Esophagus - Gastroesophageal Junction Esophagus
GTEX-13NYB-1326-SM-5K7Y1
Esophagus - Gastroesophageal Junction Esophagus
GTEX-13O21-0726-SM-5L3EG
Esophagus - Gastroesophageal Junction Esophagus
GTEX-13O3P-2726-SM-5KM2R
Esophagus - Gastroesophageal Junction Esophagus
GTEX-13O61-1226-SM-5KM21
Esophagus - Gastroesophageal Junction Esophagus
GTEX-13OVG-1026-SM-5KLZ3
Esophagus - Gastroesophageal Junction Esophagus

GTEX-13OVH-1826-SM-5K7YW
Esophagus - Gastroesophageal Junction Esophagus
GTEX-13OVJ-1426-SM-5K7Z2
Esophagus - Gastroesophageal Junction Esophagus
GTEX-13OVL-2726-SM-5J2MP
Esophagus - Gastroesophageal Junction Esophagus
GTEX-13OW6-1626-SM-5IJD5
Esophagus - Gastroesophageal Junction Esophagus
GTEX-13OW7-1926-SM-5LUB4
Esophagus - Gastroesophageal Junction Esophagus
GTEX-13OW8-2426-SM-5L3ID
Esophagus - Gastroesophageal Junction Esophagus
GTEX-13PL7-0526-SM-5J2MQ
Esophagus - Gastroesophageal Junction Esophagus
GTEX-13PVQ-1926-SM-5L3GN
Esophagus - Gastroesophageal Junction Esophagus
GTEX-13PVR-2026-SM-73KXT
Esophagus - Gastroesophageal Junction Esophagus
GTEX-13QBU-0826-SM-5IJBU
Esophagus - Gastroesophageal Junction Esophagus
GTEX-13QIC-2026-SM-5N9G9
Esophagus - Gastroesophageal Junction Esophagus
GTEX-13QJ3-1926-SM-5QGQM
Esophagus - Gastroesophageal Junction Esophagus
GTEX-13RTK-0426-SM-5RQHT
Esophagus - Gastroesophageal Junction Esophagus
GTEX-13U4I-1326-SM-5KM25
Esophagus - Gastroesophageal Junction Esophagus
GTEX-13VXT-1826-SM-5KM2F
Esophagus - Gastroesophageal Junction Esophagus
GTEX-13VXU-1126-SM-5LU3U
Esophagus - Gastroesophageal Junction Esophagus
GTEX-13W3W-1826-SM-5IJDE
Esophagus - Gastroesophageal Junction Esophagus
GTEX-13X6H-1526-SM-5Q5E2
Esophagus - Gastroesophageal Junction Esophagus
GTEX-13X6J-1726-SM-5TDCS
Esophagus - Gastroesophageal Junction Esophagus
GTEX-13YAN-1826-SM-5LU3Q
Esophagus - Gastroesophageal Junction Esophagus
GTEX-144FL-2626-SM-5TDCW
Esophagus - Gastroesophageal Junction Esophagus
GTEX-144GM-1626-SM-5O9AG
Esophagus - Gastroesophageal Junction Esophagus
GTEX-145LS-2026-SM-5Q5D7
Esophagus - Gastroesophageal Junction Esophagus
GTEX-145LT-1326-SM-5O9AQ
Esophagus - Gastroesophageal Junction Esophagus
GTEX-145MF-1526-SM-5LU95
Esophagus - Gastroesophageal Junction Esophagus
GTEX-146FH-1926-SM-5Q5B2
Esophagus - Gastroesophageal Junction Esophagus
GTEX-146FQ-1226-SM-5QGQ4
Esophagus - Gastroesophageal Junction Esophagus

GTEX-148VI-1226-SM-5RQJR
Esophagus - Gastroesophageal Junction	Esophagus
GTEX-1497J-2126-SM-5NQB8
Esophagus - Gastroesophageal Junction	Esophagus
GTEX-14AS3-0826-SM-5TDD5
Esophagus - Gastroesophageal Junction	Esophagus
GTEX-14B4R-0626-SM-5TDDQ
Esophagus - Gastroesophageal Junction	Esophagus
GTEX-14BIL-1826-SM-7DUEO
Esophagus - Gastroesophageal Junction	Esophagus
GTEX-14BMU-1026-SM-5RQJ5
Esophagus - Gastroesophageal Junction	Esophagus
GTEX-14BMV-1526-SM-793AV
Esophagus - Gastroesophageal Junction	Esophagus
GTEX-14C5O-2226-SM-5ZZW7
Esophagus - Gastroesophageal Junction	Esophagus
GTEX-14DAQ-1626-SM-5S2R1
Esophagus - Gastroesophageal Junction	Esophagus
GTEX-14DAR-0726-SM-5RQIA
Esophagus - Gastroesophageal Junction	Esophagus
GTEX-14E1K-1426-SM-5YY8V
Esophagus - Gastroesophageal Junction	Esophagus
GTEX-14E6C-0726-SM-664NB
Esophagus - Gastroesophageal Junction	Esophagus
GTEX-14E6E-0826-SM-664N7
Esophagus - Gastroesophageal Junction	Esophagus
GTEX-14E7W-1926-SM-5S2R9
Esophagus - Gastroesophageal Junction	Esophagus
GTEX-14ICK-1126-SM-69LOO
Esophagus - Gastroesophageal Junction	Esophagus
GTEX-14JG1-1826-SM-5YY9Y
Esophagus - Gastroesophageal Junction	Esophagus
GTEX-14JG6-1026-SM-6LLIF
Esophagus - Gastroesophageal Junction	Esophagus
GTEX-14PHW-1326-SM-6EU1S
Esophagus - Gastroesophageal Junction	Esophagus
GTEX-14PHX-0926-SM-5ZZWC
Esophagus - Gastroesophageal Junction	Esophagus
GTEX-14PJ2-2826-SM-6EU1N
Esophagus - Gastroesophageal Junction	Esophagus
GTEX-14PJ4-1326-SM-5ZZVM
Esophagus - Gastroesophageal Junction	Esophagus
GTEX-14PJM-1726-SM-6LLHB
Esophagus - Gastroesophageal Junction	Esophagus
GTEX-14PJN-0926-SM-6EU19
Esophagus - Gastroesophageal Junction	Esophagus
GTEX-14PK6-1226-SM-6EU1W
Esophagus - Gastroesophageal Junction	Esophagus
GTEX-14PKU-1026-SM-6EU12
Esophagus - Gastroesophageal Junction	Esophagus
GTEX-14PN3-1226-SM-69LOW
Esophagus - Gastroesophageal Junction	Esophagus
GTEX-14XAO-1326-SM-6EU1A
Esophagus - Gastroesophageal Junction	Esophagus

GTEX-15CHC-1326-SM-6LLJA
Esophagus - Gastroesophageal Junction Esophagus
GTEX-15D1Q-1526-SM-686Z2
Esophagus - Gastroesophageal Junction Esophagus
GTEX-15DCE-1526-SM-7KUFD
Esophagus - Gastroesophageal Junction Esophagus
GTEX-15DYW-1726-SM-7KUM5
Esophagus - Gastroesophageal Junction Esophagus
GTEX-15EO6-0926-SM-6M48N
Esophagus - Gastroesophageal Junction Esophagus
GTEX-15ER7-1926-SM-7KUEW
Esophagus - Gastroesophageal Junction Esophagus
GTEX-15FZZ-1326-SM-6PANN
Esophagus - Gastroesophageal Junction Esophagus
GTEX-15RIE-0526-SM-7KUF9
Esophagus - Gastroesophageal Junction Esophagus
GTEX-15RIF-1026-SM-7KUMZ
Esophagus - Gastroesophageal Junction Esophagus
GTEX-15SB6-0926-SM-7KUFM
Esophagus - Gastroesophageal Junction Esophagus
GTEX-15SHU-1626-SM-6M46B
Esophagus - Gastroesophageal Junction Esophagus
GTEX-15SHV-0926-SM-6M46I
Esophagus - Gastroesophageal Junction Esophagus
GTEX-15UF6-2226-SM-6M471
Esophagus - Gastroesophageal Junction Esophagus
GTEX-16A39-1226-SM-7KUEQ
Esophagus - Gastroesophageal Junction Esophagus
GTEX-16AAH-0826-SM-7LT9V
Esophagus - Gastroesophageal Junction Esophagus
GTEX-16GPK-1426-SM-6M47Y
Esophagus - Gastroesophageal Junction Esophagus
GTEX-16MT8-1226-SM-6M488
Esophagus - Gastroesophageal Junction Esophagus
GTEX-16NGA-1226-SM-7KFSD
Esophagus - Gastroesophageal Junction Esophagus
GTEX-16XZZ-1726-SM-7KULD
Esophagus - Gastroesophageal Junction Esophagus
GTEX-178AV-0826-SM-7KUET
Esophagus - Gastroesophageal Junction Esophagus
GTEX-17EVQ-1726-SM-7LT9R
Esophagus - Gastroesophageal Junction Esophagus
GTEX-17F96-2826-SM-7IGP3
Esophagus - Gastroesophageal Junction Esophagus
GTEX-17F98-1826-SM-7DUFK
Esophagus - Gastroesophageal Junction Esophagus
GTEX-17F9E-1026-SM-7DUF1
Esophagus - Gastroesophageal Junction Esophagus
GTEX-17HHE-0826-SM-793A2
Esophagus - Gastroesophageal Junction Esophagus
GTEX-17MF6-1726-SM-7LTA3
Esophagus - Gastroesophageal Junction Esophagus
GTEX-183FY-1726-SM-7LT9Q
Esophagus - Gastroesophageal Junction Esophagus

GTEX-18465-1326-SM-7LT9K
Esophagus - Gastroesophageal Junction	Esophagus
GTEX-18A66-1626-SM-7LTAF
Esophagus - Gastroesophageal Junction	Esophagus
GTEX-18A67-2526-SM-7LG5Z
Esophagus - Gastroesophageal Junction	Esophagus
GTEX-18A6Q-1126-SM-7LG5A
Esophagus - Gastroesophageal Junction	Esophagus
GTEX-18A7A-2526-SM-7LG6I
Esophagus - Gastroesophageal Junction	Esophagus
GTEX-18D9A-0426-SM-72D55
Esophagus - Gastroesophageal Junction	Esophagus
GTEX-1A32A-2026-SM-718AY
Esophagus - Gastroesophageal Junction	Esophagus
GTEX-1A3MV-1426-SM-72D63
Esophagus - Gastroesophageal Junction	Esophagus
GTEX-1AMFI-0126-SM-73KZ2
Esophagus - Gastroesophageal Junction	Esophagus
GTEX-1AX8Z-1826-SM-73KW6
Esophagus - Gastroesophageal Junction	Esophagus
GTEX-1AX9J-2026-SM-731AV
Esophagus - Gastroesophageal Junction	Esophagus
GTEX-1AX9K-1026-SM-73KU4
Esophagus - Gastroesophageal Junction	Esophagus
GTEX-1AYCT-1426-SM-7DUG1
Esophagus - Gastroesophageal Junction	Esophagus
GTEX-1B8KE-2026-SM-73KYL
Esophagus - Gastroesophageal Junction	Esophagus
GTEX-1B8KZ-0626-SM-73KWE
Esophagus - Gastroesophageal Junction	Esophagus
GTEX-1B8L1-1026-SM-79ONW
Esophagus - Gastroesophageal Junction	Esophagus
GTEX-1B932-1926-SM-79OOA
Esophagus - Gastroesophageal Junction	Esophagus
GTEX-1B97J-1426-SM-79OLR
Esophagus - Gastroesophageal Junction	Esophagus
GTEX-1B996-1726-SM-7IGMW
Esophagus - Gastroesophageal Junction	Esophagus
GTEX-1BAJH-2226-SM-7IGLK
Esophagus - Gastroesophageal Junction	Esophagus
GTEX-1C2JI-1326-SM-7IGMX
Esophagus - Gastroesophageal Junction	Esophagus
GTEX-1C475-0826-SM-793AD
Esophagus - Gastroesophageal Junction	Esophagus
GTEX-1C4CL-1426-SM-793CT
Esophagus - Gastroesophageal Junction	Esophagus
GTEX-1C64O-1726-SM-73KW9
Esophagus - Gastroesophageal Junction	Esophagus
GTEX-1C6VQ-2026-SM-79OOH
Esophagus - Gastroesophageal Junction	Esophagus
GTEX-1CAMQ-2626-SM-7IGNI
Esophagus - Gastroesophageal Junction	Esophagus
GTEX-1CAMR-0626-SM-7DHMI
Esophagus - Gastroesophageal Junction	Esophagus

GTEX-1CAMS-1626-SM-79394
Esophagus - Gastroesophageal Junction	Esophagus
GTEX-1CB4E-1226-SM-7MKFX
Esophagus - Gastroesophageal Junction	Esophagus
GTEX-1CB4F-1326-SM-793CW
Esophagus - Gastroesophageal Junction	Esophagus
GTEX-1CB4I-2026-SM-7DHMG
Esophagus - Gastroesophageal Junction	Esophagus
GTEX-1CB4J-2326-SM-7EPIF
Esophagus - Gastroesophageal Junction	Esophagus	GTEX-
U8XE-1326-SM-4E3JK
Esophagus - Gastroesophageal Junction	Esophagus	GTEX-
V1D1-1226-SM-4JBI5
Esophagus - Gastroesophageal Junction	Esophagus	GTEX-
V955-1226-SM-4JBI9
Esophagus - Gastroesophageal Junction	Esophagus	GTEX-
VUSG-1026-SM-4KKZN
Esophagus - Gastroesophageal Junction	Esophagus	GTEX-
W5WG-2726-SM-4LMIC
Esophagus - Gastroesophageal Junction	Esophagus	GTEX-
WEY5-1126-SM-4LMIE
Esophagus - Gastroesophageal Junction	Esophagus	GTEX-
WFG7-1226-SM-4LMK2
Esophagus - Gastroesophageal Junction	Esophagus	GTEX-
WFG8-1126-SM-4LVN1
Esophagus - Gastroesophageal Junction	Esophagus	GTEX-
WFJO-0426-SM-4LVM7
Esophagus - Gastroesophageal Junction	Esophagus	GTEX-
WFON-1026-SM-4LVMD
Esophagus - Gastroesophageal Junction	Esophagus	GTEX-
WHSE-1426-SM-4M1XG
Esophagus - Gastroesophageal Junction	Esophagus	GTEX-
WQUQ-1826-SM-4OOSD
Esophagus - Gastroesophageal Junction	Esophagus	GTEX-
WVLH-1526-SM-4MVOC
Esophagus - Gastroesophageal Junction	Esophagus	GTEX-
WXYG-1026-SM-4ONCD
Esophagus - Gastroesophageal Junction	Esophagus	GTEX-
WY7C-0826-SM-4ONCG
Esophagus - Gastroesophageal Junction	Esophagus	GTEX-
WYBS-1626-SM-4ONCA
Esophagus - Gastroesophageal Junction	Esophagus	GTEX-
WYJK-2126-SM-4ONDG
Esophagus - Gastroesophageal Junction	Esophagus	GTEX-
WYVS-1026-SM-4SOJW
Esophagus - Gastroesophageal Junction	Esophagus	GTEX-
WZTO-1726-SM-4PQYV
Esophagus - Gastroesophageal Junction	Esophagus	GTEX-
X15G-1026-SM-4PQZF
Esophagus - Gastroesophageal Junction	Esophagus	GTEX-
X3Y1-1126-SM-4PQZ9
Esophagus - Gastroesophageal Junction	Esophagus	GTEX-
X4LF-0826-SM-4QASC
Esophagus - Gastroesophageal Junction	Esophagus	GTEX-

X5EB-1026-SM-46MVU
Esophagus - Gastroesophageal Junction Esophagus GTEX-
X8HC-1326-SM-46MWB
Esophagus - Gastroesophageal Junction Esophagus GTEX-
XBED-1126-SM-48TCF
Esophagus - Gastroesophageal Junction Esophagus GTEX-
XBEW-1526-SM-4AT4K
Esophagus - Gastroesophageal Junction Esophagus GTEX-
XGQ4-1626-SM-4AT6J
Esophagus - Gastroesophageal Junction Esophagus GTEX-
XPVG-1426-SM-4B668
Esophagus - Gastroesophageal Junction Esophagus GTEX-
XQ3S-1026-SM-4BOPJ
Esophagus - Gastroesophageal Junction Esophagus GTEX-
XQ8I-1826-SM-4BOOJ
Esophagus - Gastroesophageal Junction Esophagus GTEX-
XUW1-1326-SM-4BOO1
Esophagus - Gastroesophageal Junction Esophagus GTEX-
XUZC-0526-SM-4BOPF
Esophagus - Gastroesophageal Junction Esophagus GTEX-
XV7Q-1226-SM-4BRVT
Esophagus - Gastroesophageal Junction Esophagus GTEX-
XXEK-0526-SM-4BRWD
Esophagus - Gastroesophageal Junction Esophagus GTEX-
XYKS-1026-SM-4BRVH
Esophagus - Gastroesophageal Junction Esophagus GTEX-
Y111-1826-SM-4SOIR
Esophagus - Gastroesophageal Junction Esophagus GTEX-
Y114-1126-SM-4TT7V
Esophagus - Gastroesophageal Junction Esophagus GTEX-
Y3I4-0926-SM-4TT2B
Esophagus - Gastroesophageal Junction Esophagus GTEX-
Y3IK-1326-SM-4YCD8
Esophagus - Gastroesophageal Junction Esophagus GTEX-
Y5LM-1226-SM-4VDT6
Esophagus - Gastroesophageal Junction Esophagus GTEX-
Y5V5-1526-SM-4VBQ1
Esophagus - Gastroesophageal Junction Esophagus GTEX-
Y8E4-1926-SM-4VBPW
Esophagus - Gastroesophageal Junction Esophagus GTEX-
Y8LW-0526-SM-4VDS7
Esophagus - Gastroesophageal Junction Esophagus GTEX-
Y9LG-0926-SM-4VDS6
Esophagus - Gastroesophageal Junction Esophagus GTEX-
YB5E-1026-SM-5IFI2
Esophagus - Gastroesophageal Junction Esophagus GTEX-
YB5K-0626-SM-5LUB5
Esophagus - Gastroesophageal Junction Esophagus GTEX-
YEC3-2326-SM-5IFJF
Esophagus - Gastroesophageal Junction Esophagus GTEX-
YEC4-1226-SM-5CVLX
Esophagus - Gastroesophageal Junction Esophagus GTEX-
YF7O-1026-SM-5P9IP
Esophagus - Gastroesophageal Junction Esophagus GTEX-

YFCO-0726-SM-5ZZVJ
Esophagus - Gastroesophageal Junction	Esophagus	GTEX-YJ8A-1426-SM-5PNV7
Esophagus - Gastroesophageal Junction	Esophagus	GTEX-YJ8O-0326-SM-5RQJW
Esophagus - Gastroesophageal Junction	Esophagus	GTEX-Z9EW-0326-SM-5CVM8
Esophagus - Gastroesophageal Junction	Esophagus	GTEX-ZAK1-1826-SM-5HL93
Esophagus - Gastroesophageal Junction	Esophagus	GTEX-ZDYS-0726-SM-4WKGN
Esophagus - Gastroesophageal Junction	Esophagus	GTEX-ZEX8-1526-SM-4WWBE
Esophagus - Gastroesophageal Junction	Esophagus	GTEX-ZF2S-1326-SM-4WWAZ
Esophagus - Gastroesophageal Junction	Esophagus	GTEX-ZGAY-1526-SM-4WWEO
Esophagus - Gastroesophageal Junction	Esophagus	GTEX-ZP4G-1126-SM-4WWCI
Esophagus - Gastroesophageal Junction	Esophagus	GTEX-ZPCL-1326-SM-57WFN
Esophagus - Gastroesophageal Junction	Esophagus	GTEX-ZPIC-1926-SM-57WFQ
Esophagus - Gastroesophageal Junction	Esophagus	GTEX-ZQUD-0626-SM-51MR2
Esophagus - Gastroesophageal Junction	Esophagus	GTEX-ZT9W-1426-SM-4YCD2
Esophagus - Gastroesophageal Junction	Esophagus	GTEX-ZTSS-0826-SM-5985L
Esophagus - Gastroesophageal Junction	Esophagus	GTEX-ZTTD-1226-SM-51MS2
Esophagus - Gastroesophageal Junction	Esophagus	GTEX-ZV6S-0726-SM-57WFY
Esophagus - Gastroesophageal Junction	Esophagus	GTEX-ZV7C-1326-SM-5BC7G
Esophagus - Gastroesophageal Junction	Esophagus	GTEX-ZVP2-1426-SM-5NQ7P
Esophagus - Gastroesophageal Junction	Esophagus	GTEX-ZVZP-1626-SM-5GIDJ
Esophagus - Gastroesophageal Junction	Esophagus	GTEX-ZY6K-0826-SM-5A5L7
Esophagus - Gastroesophageal Junction	Esophagus	GTEX-ZYFC-2226-SM-5NQ96
Esophagus - Gastroesophageal Junction	Esophagus	GTEX-ZYFG-1226-SM-5GIC7
Esophagus - Gastroesophageal Junction	Esophagus	GTEX-ZYVF-2526-SM-5E45C
Esophagus - Gastroesophageal Junction	Esophagus	GTEX-ZYW4-1626-SM-5GIE6
Esophagus - Mucosa	Esophagus	GTEX-111CU-0726-SM-5GZYD
Esophagus - Mucosa	Esophagus	GTEX-111YS-0926-SM-5EGGI
Esophagus - Mucosa	Esophagus	GTEX-1122O-1726-SM-5GICM
Esophagus - Mucosa	Esophagus	GTEX-117YW-1926-SM-5H121
Esophagus - Mucosa	Esophagus	GTEX-117YX-1626-SM-5GZZG

Esophagus - Mucosa	Esophagus	GTEX-11DXX-1126-SM-5GZZK
Esophagus - Mucosa	Esophagus	GTEX-11DXY-1426-SM-5N9CN
Esophagus - Mucosa	Esophagus	GTEX-11DXZ-1226-SM-5H11L
Esophagus - Mucosa	Esophagus	GTEX-11DYG-1326-SM-5N9DO
Esophagus - Mucosa	Esophagus	GTEX-11DZ1-2026-SM-5A5KG
Esophagus - Mucosa	Esophagus	GTEX-11EI6-1526-SM-5P9GL
Esophagus - Mucosa	Esophagus	GTEX-11EM3-0826-SM-5N9CC
Esophagus - Mucosa	Esophagus	GTEX-11EMC-2226-SM-5A5JW
Esophagus - Mucosa	Esophagus	GTEX-11EQ9-0726-SM-5A5K2
Esophagus - Mucosa	Esophagus	GTEX-11GSP-1826-SM-5N9DQ
Esophagus - Mucosa	Esophagus	GTEX-11I78-1526-SM-5A5K7
Esophagus - Mucosa	Esophagus	GTEX-11LCK-1726-SM-5S2OQ
Esophagus - Mucosa	Esophagus	GTEX-11NSD-1126-SM-5N9BQ
Esophagus - Mucosa	Esophagus	GTEX-11NUK-1326-SM-5P9GN
Esophagus - Mucosa	Esophagus	GTEX-11ONC-1526-SM-5GU6S
Esophagus - Mucosa	Esophagus	GTEX-11P7K-1326-SM-5BC5G
Esophagus - Mucosa	Esophagus	GTEX-11P81-0826-SM-5P9GR
Esophagus - Mucosa	Esophagus	GTEX-11P82-0526-SM-5986P
Esophagus - Mucosa	Esophagus	GTEX-11TT1-0526-SM-5P9JO
Esophagus - Mucosa	Esophagus	GTEX-11TUW-1326-SM-5EQKV
Esophagus - Mucosa	Esophagus	GTEX-11VI4-0526-SM-5EQ48
Esophagus - Mucosa	Esophagus	GTEX-11XUK-0826-SM-5GU6P
Esophagus - Mucosa	Esophagus	GTEX-11ZTS-2626-SM-5EGIC
Esophagus - Mucosa	Esophagus	GTEX-11ZU8-1526-SM-5EGHX
Esophagus - Mucosa	Esophagus	GTEX-1211K-0926-SM-5FQTL
Esophagus - Mucosa	Esophagus	GTEX-1212Z-2126-SM-5FQTM
Esophagus - Mucosa	Esophagus	GTEX-12696-1326-SM-5FQTJ
Esophagus - Mucosa	Esophagus	GTEX-1269C-1026-SM-5N9EY
Esophagus - Mucosa	Esophagus	GTEX-12BJ1-1626-SM-5HL8Y
Esophagus - Mucosa	Esophagus	GTEX-12C56-1226-SM-5FQSU
Esophagus - Mucosa	Esophagus	GTEX-12KS4-0926-SM-5BC5S
Esophagus - Mucosa	Esophagus	GTEX-12WSA-1426-SM-5EGJU
Esophagus - Mucosa	Esophagus	GTEX-12WSD-1826-SM-5GCOA
Esophagus - Mucosa	Esophagus	GTEX-12WSG-5007-SM-7DUGZ
Esophagus - Mucosa	Esophagus	GTEX-12WSK-1126-SM-5LZV8
Esophagus - Mucosa	Esophagus	GTEX-12WSL-1526-SM-5GCP8
Esophagus - Mucosa	Esophagus	GTEX-12ZZW-1726-SM-5DUVD
Esophagus - Mucosa	Esophagus	GTEX-12ZZX-1326-SM-5DUWI
Esophagus - Mucosa	Esophagus	GTEX-12ZZY-1426-SM-5N9EI
Esophagus - Mucosa	Esophagus	GTEX-13111-0826-SM-5LZV3
Esophagus - Mucosa	Esophagus	GTEX-13113-5007-SM-793CD
Esophagus - Mucosa	Esophagus	GTEX-131XE-1426-SM-5HL7V
Esophagus - Mucosa	Esophagus	GTEX-131XF-0826-SM-5GICG
Esophagus - Mucosa	Esophagus	GTEX-131XG-1026-SM-5GCMX
Esophagus - Mucosa	Esophagus	GTEX-131YS-1426-SM-5IJF7
Esophagus - Mucosa	Esophagus	GTEX-132AR-2226-SM-5J2NT
Esophagus - Mucosa	Esophagus	GTEX-132Q8-1826-SM-5EGIY
Esophagus - Mucosa	Esophagus	GTEX-132QS-1426-SM-5P9GE
Esophagus - Mucosa	Esophagus	GTEX-133LE-0626-SM-5IFFJ
Esophagus - Mucosa	Esophagus	GTEX-1399Q-1126-SM-5P9JA
Esophagus - Mucosa	Esophagus	GTEX-1399R-0826-SM-5IJEL
Esophagus - Mucosa	Esophagus	GTEX-1399S-1326-SM-5P9GH
Esophagus - Mucosa	Esophagus	GTEX-1399U-1026-SM-5J1OC
Esophagus - Mucosa	Esophagus	GTEX-139T6-0626-SM-5IFF9

Esophagus - Mucosa	Esophagus	GTEX-139TU-1226-SM-5J2NL
Esophagus - Mucosa	Esophagus	GTEX-139UW-1126-SM-5IJEJ
Esophagus - Mucosa	Esophagus	GTEX-139YR-1726-SM-5IFFK
Esophagus - Mucosa	Esophagus	GTEX-13CF3-1626-SM-5IFG9
Esophagus - Mucosa	Esophagus	GTEX-13D11-1926-SM-5IFGB
Esophagus - Mucosa	Esophagus	GTEX-13FH7-0526-SM-5IJF8
Esophagus - Mucosa	Esophagus	GTEX-13FHO-1726-SM-5IFGM
Esophagus - Mucosa	Esophagus	GTEX-13FHP-1526-SM-5IJGE
Esophagus - Mucosa	Esophagus	GTEX-13FLV-1126-SM-5J2NO
Esophagus - Mucosa	Esophagus	GTEX-13FTW-1626-SM-5KM4N
Esophagus - Mucosa	Esophagus	GTEX-13FTX-0526-SM-5IFGS
Esophagus - Mucosa	Esophagus	GTEX-13G51-0926-SM-5IFFU
Esophagus - Mucosa	Esophagus	GTEX-13NZB-1226-SM-5J2MW
Esophagus - Mucosa	Esophagus	GTEX-13O1R-1326-SM-5IJF5
Esophagus - Mucosa	Esophagus	GTEX-13O21-0626-SM-5IJDI
Esophagus - Mucosa	Esophagus	GTEX-13O3Q-0826-SM-5IFGF
Esophagus - Mucosa	Esophagus	GTEX-13O61-0926-SM-5IFFE
Esophagus - Mucosa	Esophagus	GTEX-13OVG-0626-SM-5IFEP
Esophagus - Mucosa	Esophagus	GTEX-13OVI-2626-SM-5KM4T
Esophagus - Mucosa	Esophagus	GTEX-13OVJ-1226-SM-5L3HA
Esophagus - Mucosa	Esophagus	GTEX-13OVK-1126-SM-6PAMR
Esophagus - Mucosa	Esophagus	GTEX-13OW8-2326-SM-5L3I9
Esophagus - Mucosa	Esophagus	GTEX-13PL7-0226-SM-731DT
Esophagus - Mucosa	Esophagus	GTEX-13PVQ-1726-SM-5IFF3
Esophagus - Mucosa	Esophagus	GTEX-13PVR-1826-SM-5Q5CC
Esophagus - Mucosa	Esophagus	GTEX-13QBU-1026-SM-5LU3R
Esophagus - Mucosa	Esophagus	GTEX-13QJ3-1726-SM-5RQK9
Esophagus - Mucosa	Esophagus	GTEX-13RTK-0526-SM-5S2QF
Esophagus - Mucosa	Esophagus	GTEX-13S86-0826-SM-5SI6J
Esophagus - Mucosa	Esophagus	GTEX-13SLX-1726-SM-5Q5E5
Esophagus - Mucosa	Esophagus	GTEX-13U4I-1126-SM-5LU3S
Esophagus - Mucosa	Esophagus	GTEX-13W3W-1626-SM-5LU35
Esophagus - Mucosa	Esophagus	GTEX-13W46-1926-SM-5J2MF
Esophagus - Mucosa	Esophagus	GTEX-13X6H-1326-SM-5LU42
Esophagus - Mucosa	Esophagus	GTEX-13X6I-1026-SM-5LU4C
Esophagus - Mucosa	Esophagus	GTEX-13X6K-2126-SM-5O9D4
Esophagus - Mucosa	Esophagus	GTEX-13YAN-1926-SM-7EPH9
Esophagus - Mucosa	Esophagus	GTEX-144FL-2726-SM-5O98Z
Esophagus - Mucosa	Esophagus	GTEX-144GM-1426-SM-5LU31
Esophagus - Mucosa	Esophagus	GTEX-144GN-0626-SM-5O9B2
Esophagus - Mucosa	Esophagus	GTEX-144GO-2726-SM-5O9A2
Esophagus - Mucosa	Esophagus	GTEX-145LS-1826-SM-5LUAZ
Esophagus - Mucosa	Esophagus	GTEX-145LT-1126-SM-5LUA1
Esophagus - Mucosa	Esophagus	GTEX-145LU-0926-SM-79OJL
Esophagus - Mucosa	Esophagus	GTEX-145LV-1326-SM-5S2QI
Esophagus - Mucosa	Esophagus	GTEX-145ME-0526-SM-5QGQV
Esophagus - Mucosa	Esophagus	GTEX-145MF-1426-SM-5O9B6
Esophagus - Mucosa	Esophagus	GTEX-145MN-1026-SM-5NQ9G
Esophagus - Mucosa	Esophagus	GTEX-145MO-1426-SM-5Q5BS
Esophagus - Mucosa	Esophagus	GTEX-146FH-1726-SM-5QGQ2
Esophagus - Mucosa	Esophagus	GTEX-146FQ-1026-SM-5LU8I
Esophagus - Mucosa	Esophagus	GTEX-146FR-0426-SM-5SI97
Esophagus - Mucosa	Esophagus	GTEX-147F3-0826-SM-5NQA7
Esophagus - Mucosa	Esophagus	GTEX-148VI-1026-SM-5TDDJ

Esophagus - Mucosa	Esophagus	GTEX-148VJ-1026-SM-5LU98
Esophagus - Mucosa	Esophagus	GTEX-1497J-2726-SM-5SI9W
Esophagus - Mucosa	Esophagus	GTEX-14A6H-1326-SM-5NQBC
Esophagus - Mucosa	Esophagus	GTEX-14AS3-0626-SM-5QGQD
Esophagus - Mucosa	Esophagus	GTEX-14B4R-0326-SM-5TDDP
Esophagus - Mucosa	Esophagus	GTEX-14BIM-1326-SM-7EWD1
Esophagus - Mucosa	Esophagus	GTEX-14BIN-2726-SM-5RQIE
Esophagus - Mucosa	Esophagus	GTEX-14BMU-0926-SM-5S2QB
Esophagus - Mucosa	Esophagus	GTEX-14C38-2126-SM-5RQJO
Esophagus - Mucosa	Esophagus	GTEX-14C5O-1626-SM-73KYH
Esophagus - Mucosa	Esophagus	GTEX-14DAQ-1426-SM-5RQJD
Esophagus - Mucosa	Esophagus	GTEX-14DAR-0826-SM-73KYJ
Esophagus - Mucosa	Esophagus	GTEX-14E1K-1226-SM-5S2PD
Esophagus - Mucosa	Esophagus	GTEX-14E6E-0726-SM-5S2R3
Esophagus - Mucosa	Esophagus	GTEX-14H4A-1626-SM-5SI69
Esophagus - Mucosa	Esophagus	GTEX-14ICK-2026-SM-6AJB2
Esophagus - Mucosa	Esophagus	GTEX-14ICL-0526-SM-5RQJ6
Esophagus - Mucosa	Esophagus	GTEX-14JG6-0926-SM-6871Y
Esophagus - Mucosa	Esophagus	GTEX-14JIY-0926-SM-6871D
Esophagus - Mucosa	Esophagus	GTEX-14PHY-0626-SM-664NN
Esophagus - Mucosa	Esophagus	GTEX-14PJ3-0626-SM-7KFRZ
Esophagus - Mucosa	Esophagus	GTEX-14PJ4-1526-SM-664OA
Esophagus - Mucosa	Esophagus	GTEX-14PJ5-0526-SM-664OE
Esophagus - Mucosa	Esophagus	GTEX-14PJ6-1026-SM-686ZB
Esophagus - Mucosa	Esophagus	GTEX-14PJM-1526-SM-664NY
Esophagus - Mucosa	Esophagus	GTEX-14PJN-0526-SM-68724
Esophagus - Mucosa	Esophagus	GTEX-14PK6-1326-SM-686ZE
Esophagus - Mucosa	Esophagus	GTEX-14PKV-1726-SM-6871U
Esophagus - Mucosa	Esophagus	GTEX-15DCD-1626-SM-6AJBH
Esophagus - Mucosa	Esophagus	GTEX-15DCE-1326-SM-6LPJL
Esophagus - Mucosa	Esophagus	GTEX-15DCZ-2426-SM-6871N
Esophagus - Mucosa	Esophagus	GTEX-15DYW-1826-SM-6LPIY
Esophagus - Mucosa	Esophagus	GTEX-15EOM-5013-SM-7MKF9
Esophagus - Mucosa	Esophagus	GTEX-15ER7-2126-SM-7939E
Esophagus - Mucosa	Esophagus	GTEX-15ETS-1926-SM-7KUN5
Esophagus - Mucosa	Esophagus	GTEX-15EU6-1626-SM-6LPKF
Esophagus - Mucosa	Esophagus	GTEX-15F5U-0926-SM-6LPKB
Esophagus - Mucosa	Esophagus	GTEX-15RIE-0826-SM-6M47X
Esophagus - Mucosa	Esophagus	GTEX-15RIF-0826-SM-6M47C
Esophagus - Mucosa	Esophagus	GTEX-15RJ7-0926-SM-6LPJO
Esophagus - Mucosa	Esophagus	GTEX-15RJE-0926-SM-6LLHZ
Esophagus - Mucosa	Esophagus	GTEX-15SB6-0726-SM-6M48F
Esophagus - Mucosa	Esophagus	GTEX-15SHU-1426-SM-6LPIN
Esophagus - Mucosa	Esophagus	GTEX-15UF6-2326-SM-6M465
Esophagus - Mucosa	Esophagus	GTEX-15UF7-0926-SM-6M46F
Esophagus - Mucosa	Esophagus	GTEX-169BO-0526-SM-793AK
Esophagus - Mucosa	Esophagus	GTEX-16A39-1026-SM-6LPJU
Esophagus - Mucosa	Esophagus	GTEX-16AAH-0726-SM-793A9
Esophagus - Mucosa	Esophagus	GTEX-16BQI-1526-SM-6LLIE
Esophagus - Mucosa	Esophagus	GTEX-16MT8-1326-SM-6M47R
Esophagus - Mucosa	Esophagus	GTEX-16MTA-0826-SM-6LLIC
Esophagus - Mucosa	Esophagus	GTEX-16NGA-1426-SM-718AC
Esophagus - Mucosa	Esophagus	GTEX-16NPX-1626-SM-6LLI5
Esophagus - Mucosa	Esophagus	GTEX-16XZY-0926-SM-7938M

Esophagus - Mucosa	Esophagus	GTEX-16Z82-1926-SM-6M47U
Esophagus - Mucosa	Esophagus	GTEX-178AV-0526-SM-6LPJH
Esophagus - Mucosa	Esophagus	GTEX-17EUY-1426-SM-7938X
Esophagus - Mucosa	Esophagus	GTEX-17EVQ-1926-SM-7IGOF
Esophagus - Mucosa	Esophagus	GTEX-17F96-2526-SM-7DHL3
Esophagus - Mucosa	Esophagus	GTEX-17F98-1726-SM-7938O
Esophagus - Mucosa	Esophagus	GTEX-17F9E-1926-SM-7IGQF
Esophagus - Mucosa	Esophagus	GTEX-17HGU-1426-SM-7938G
Esophagus - Mucosa	Esophagus	GTEX-17HHE-0926-SM-7938K
Esophagus - Mucosa	Esophagus	GTEX-17JCI-1226-SM-793D9
Esophagus - Mucosa	Esophagus	GTEX-17KNJ-1326-SM-7KFTG
Esophagus - Mucosa	Esophagus	GTEX-17MFQ-0526-SM-7KFTX
Esophagus - Mucosa	Esophagus	GTEX-183FY-1526-SM-7IGOW
Esophagus - Mucosa	Esophagus	GTEX-183WM-1726-SM-7KFT5
Esophagus - Mucosa	Esophagus	GTEX-18465-1126-SM-7LG6E
Esophagus - Mucosa	Esophagus	GTEX-18A66-1426-SM-7KFRT
Esophagus - Mucosa	Esophagus	GTEX-18A67-2426-SM-7LT95
Esophagus - Mucosa	Esophagus	GTEX-18A6Q-1226-SM-7KFS9
Esophagus - Mucosa	Esophagus	GTEX-18A7A-2326-SM-7KFRH
Esophagus - Mucosa	Esophagus	GTEX-18D9A-0526-SM-7KFSW
Esophagus - Mucosa	Esophagus	GTEX-18D9B-2026-SM-7LT96
Esophagus - Mucosa	Esophagus	GTEX-18D9U-2126-SM-7LG4Y
Esophagus - Mucosa	Esophagus	GTEX-1A32A-1826-SM-72D5G
Esophagus - Mucosa	Esophagus	GTEX-1A3MV-1326-SM-7LG6C
Esophagus - Mucosa	Esophagus	GTEX-1AMEY-0226-SM-7189Q
Esophagus - Mucosa	Esophagus	GTEX-1AMFI-0226-SM-731D5
Esophagus - Mucosa	Esophagus	GTEX-1AX9I-1426-SM-7DHM8
Esophagus - Mucosa	Esophagus	GTEX-1AX9J-1826-SM-731FM
Esophagus - Mucosa	Esophagus	GTEX-1AX9K-1126-SM-731DQ
Esophagus - Mucosa	Esophagus	GTEX-1AYD5-0926-SM-793AF
Esophagus - Mucosa	Esophagus	GTEX-1B8KE-1826-SM-731FD
Esophagus - Mucosa	Esophagus	GTEX-1B8KZ-0726-SM-7938W
Esophagus - Mucosa	Esophagus	GTEX-1B933-2026-SM-7EWFC
Esophagus - Mucosa	Esophagus	GTEX-1B97I-0926-SM-731DZ
Esophagus - Mucosa	Esophagus	GTEX-1C2JI-1126-SM-731FH
Esophagus - Mucosa	Esophagus	GTEX-1C475-0926-SM-7MKFI
Esophagus - Mucosa	Esophagus	GTEX-1C64O-1826-SM-7DHM4
Esophagus - Mucosa	Esophagus	GTEX-1C6VQ-1826-SM-7EWEH
Esophagus - Mucosa	Esophagus	GTEX-1CB4E-1026-SM-793AB
Esophagus - Mucosa	Esophagus	GTEX-1CB4F-1526-SM-7MKFM
Esophagus - Mucosa	Esophagus	GTEX-1CB4G-1326-SM-7MKFP
Esophagus - Mucosa	Esophagus	GTEX-1CB4I-1026-SM-7MKFZ
Esophagus - Mucosa	Esophagus	GTEX-1E1VI-1526-SM-7IGQ2
Esophagus - Mucosa	Esophagus	GTEX-1EH9U-2426-SM-7EWFG
Esophagus - Mucosa	Esophagus	GTEX-1EMGI-1926-SM-7938Y
Esophagus - Mucosa	Esophagus	GTEX-1EU9M-2326-SM-7MKGS
Esophagus - Mucosa	Esophagus	GTEX-1EWIQ-1526-SM-79OO7
Esophagus - Mucosa	Esophagus	GTEX-1EX96-2226-SM-7MKGT
Esophagus - Mucosa	Esophagus	GTEX-1F5PK-1526-SM-7MKGV
Esophagus - Mucosa	Esophagus	GTEX-1F75I-1726-SM-7MKGW
Esophagus - Mucosa	Esophagus	GTEX-1F88F-1126-SM-7MKHL
Esophagus - Mucosa	Esophagus	GTEX-1GL5R-1526-SM-7MKHO
Esophagus - Mucosa	Esophagus	GTEX-N7MT-0326-SM-48TDP
Esophagus - Mucosa	Esophagus	GTEX-NFK9-1126-SM-3LK78

Esophagus - Mucosa	Esophagus	GTEX-NPJ8-1926-SM-3MJGB
Esophagus - Mucosa	Esophagus	GTEX-O5YV-1026-SM-3LK72
Esophagus - Mucosa	Esophagus	GTEX-OIZG-1026-SM-3LK5X
Esophagus - Mucosa	Esophagus	GTEX-OIZH-0626-SM-3NB1L
Esophagus - Mucosa	Esophagus	GTEX-OIZI-1426-SM-3NB1T
Esophagus - Mucosa	Esophagus	GTEX-OOBK-0625-SM-3LK5P
Esophagus - Mucosa	Esophagus	GTEX-OXRK-1326-SM-3NB1A
Esophagus - Mucosa	Esophagus	GTEX-OXRL-0626-SM-3NM9C
Esophagus - Mucosa	Esophagus	GTEX-OXRN-1726-SM-3NM9B
Esophagus - Mucosa	Esophagus	GTEX-OXRP-0926-SM-48TC1
Esophagus - Mucosa	Esophagus	GTEX-P4PP-0626-SM-3NM9X
Esophagus - Mucosa	Esophagus	GTEX-P4PQ-0626-SM-3NMCU
Esophagus - Mucosa	Esophagus	GTEX-P4QS-0626-SM-3NMD1
Esophagus - Mucosa	Esophagus	GTEX-P4QT-0626-SM-3NMCP
Esophagus - Mucosa	Esophagus	GTEX-P78B-1026-SM-3NMC4
Esophagus - Mucosa	Esophagus	GTEX-PLZ4-1626-SM-3P618
Esophagus - Mucosa	Esophagus	GTEX-PLZ5-0926-SM-3TW8L
Esophagus - Mucosa	Esophagus	GTEX-PLZ6-0526-SM-3P61C
Esophagus - Mucosa	Esophagus	GTEX-POYW-1326-SM-48TCG
Esophagus - Mucosa	Esophagus	GTEX-PSDG-1326-SM-48TD2
Esophagus - Mucosa	Esophagus	GTEX-PVOW-1626-SM-48TC9
Esophagus - Mucosa	Esophagus	GTEX-PWCY-0726-SM-48TCS
Esophagus - Mucosa	Esophagus	GTEX-PWN1-0626-SM-48TDT
Esophagus - Mucosa	Esophagus	GTEX-Q2AH-0826-SM-48TZN
Esophagus - Mucosa	Esophagus	GTEX-Q2AI-0626-SM-48TZU
Esophagus - Mucosa	Esophagus	GTEX-QCQG-0626-SM-48U21
Esophagus - Mucosa	Esophagus	GTEX-QDVJ-0726-SM-48U1W
Esophagus - Mucosa	Esophagus	GTEX-QDVN-1026-SM-48TZA
Esophagus - Mucosa	Esophagus	GTEX-QEG4-0726-SM-4R1JL
Esophagus - Mucosa	Esophagus	GTEX-QEG5-1426-SM-447AS
Esophagus - Mucosa	Esophagus	GTEX-QEL4-1426-SM-447AC
Esophagus - Mucosa	Esophagus	GTEX-QESD-0826-SM-47J4G
Esophagus - Mucosa	Esophagus	GTEX-QLQ7-0926-SM-447BC
Esophagus - Mucosa	Esophagus	GTEX-QLQW-0526-SM-4R1KS
Esophagus - Mucosa	Esophagus	GTEX-QV44-1126-SM-4R1KO
Esophagus - Mucosa	Esophagus	GTEX-QVUS-0626-SM-447CC
Esophagus - Mucosa	Esophagus	GTEX-R3RS-1326-SM-48FE7
Esophagus - Mucosa	Esophagus	GTEX-R45C-1226-SM-48FEE
Esophagus - Mucosa	Esophagus	GTEX-R53T-1126-SM-48FD4
Esophagus - Mucosa	Esophagus	GTEX-R55C-0826-SM-48FCL
Esophagus - Mucosa	Esophagus	GTEX-R55D-1126-SM-48FEB
Esophagus - Mucosa	Esophagus	GTEX-R55E-1326-SM-48FCR
Esophagus - Mucosa	Esophagus	GTEX-R55G-1026-SM-48FDI
Esophagus - Mucosa	Esophagus	GTEX-REY6-1326-SM-48FDO
Esophagus - Mucosa	Esophagus	GTEX-RM2N-0726-SM-48FD5
Esophagus - Mucosa	Esophagus	GTEX-RN64-1026-SM-48FDX
Esophagus - Mucosa	Esophagus	GTEX-RNOR-1226-SM-48FDQ
Esophagus - Mucosa	Esophagus	GTEX-RTLS-1126-SM-46MUQ
Esophagus - Mucosa	Esophagus	GTEX-RU1J-0326-SM-46MUM
Esophagus - Mucosa	Esophagus	GTEX-RU72-0726-SM-46MUW
Esophagus - Mucosa	Esophagus	GTEX-RUSQ-0826-SM-47JWW
Esophagus - Mucosa	Esophagus	GTEX-RVPV-0526-SM-47JYL
Esophagus - Mucosa	Esophagus	GTEX-RWS6-0826-SM-47JXF
Esophagus - Mucosa	Esophagus	GTEX-S32W-0926-SM-4AD5X

Esophagus - Mucosa	Esophagus	GTEX-S33H-0826-SM-4AD5Y
Esophagus - Mucosa	Esophagus	GTEX-S341-0526-SM-4AD5U
Esophagus - Mucosa	Esophagus	GTEX-S3XE-0926-SM-4AD4S
Esophagus - Mucosa	Esophagus	GTEX-S4P3-0426-SM-4AD56
Esophagus - Mucosa	Esophagus	GTEX-S4Q7-0626-SM-4AD5M
Esophagus - Mucosa	Esophagus	GTEX-S7SF-0926-SM-4AD4M
Esophagus - Mucosa	Esophagus	GTEX-S95S-0426-SM-4B64I
Esophagus - Mucosa	Esophagus	GTEX-SE5C-1226-SM-4BRWV
Esophagus - Mucosa	Esophagus	GTEX-SNMC-0526-SM-4DM69
Esophagus - Mucosa	Esophagus	GTEX-SNOS-0626-SM-4DM5F
Esophagus - Mucosa	Esophagus	GTEX-T2IS-0826-SM-4DM6L
Esophagus - Mucosa	Esophagus	GTEX-T2YK-1326-SM-4DM6S
Esophagus - Mucosa	Esophagus	GTEX-T5JC-1726-SM-4DM6U
Esophagus - Mucosa	Esophagus	GTEX-T5JW-1326-SM-4DM5G
Esophagus - Mucosa	Esophagus	GTEX-T6MO-0526-SM-4DM6R
Esophagus - Mucosa	Esophagus	GTEX-T8EM-0526-SM-4DM64
Esophagus - Mucosa	Esophagus	GTEX-TKQ1-0326-SM-4DXSM
Esophagus - Mucosa	Esophagus	GTEX-TML8-1526-SM-4DXUX
Esophagus - Mucosa	Esophagus	GTEX-TMMY-1126-SM-4DXSX
Esophagus - Mucosa	Esophagus	GTEX-TSE9-1226-SM-4DXTM
Esophagus - Mucosa	Esophagus	GTEX-U3ZH-1026-SM-4DXUZ
Esophagus - Mucosa	Esophagus	GTEX-U3ZM-0526-SM-4DXTB
Esophagus - Mucosa	Esophagus	GTEX-U3ZN-1726-SM-4DXUQ
Esophagus - Mucosa	Esophagus	GTEX-U4B1-0826-SM-4DXTW
Esophagus - Mucosa	Esophagus	GTEX-U8XE-1726-SM-4E3IF
Esophagus - Mucosa	Esophagus	GTEX-UJHI-0826-SM-4IHLM
Esophagus - Mucosa	Esophagus	GTEX-UJMC-0926-SM-4IHLK
Esophagus - Mucosa	Esophagus	GTEX-UPK5-1226-SM-4IHL8
Esophagus - Mucosa	Esophagus	GTEX-V1D1-1026-SM-4JBHE
Esophagus - Mucosa	Esophagus	GTEX-V955-1126-SM-4JBH3
Esophagus - Mucosa	Esophagus	GTEX-VJYA-0726-SM-4KL1T
Esophagus - Mucosa	Esophagus	GTEX-VUSG-1226-SM-4KKZF
Esophagus - Mucosa	Esophagus	GTEX-W5WG-1626-SM-4LMKG
Esophagus - Mucosa	Esophagus	GTEX-WEY5-0926-SM-4LMI4
Esophagus - Mucosa	Esophagus	GTEX-WFG7-1126-SM-4LMK3
Esophagus - Mucosa	Esophagus	GTEX-WFG8-1226-SM-4V6EI
Esophagus - Mucosa	Esophagus	GTEX-WFJO-0826-SM-4LVM5
Esophagus - Mucosa	Esophagus	GTEX-WFON-0826-SM-4LVMI
Esophagus - Mucosa	Esophagus	GTEX-WH7G-0926-SM-4LVMJ
Esophagus - Mucosa	Esophagus	GTEX-WHPG-1126-SM-4M1XT
Esophagus - Mucosa	Esophagus	GTEX-WHSB-0426-SM-4M1XI
Esophagus - Mucosa	Esophagus	GTEX-WHSE-1526-SM-4M1XH
Esophagus - Mucosa	Esophagus	GTEX-WHWD-0926-SM-4OORY
Esophagus - Mucosa	Esophagus	GTEX-WK11-1026-SM-4OOS8
Esophagus - Mucosa	Esophagus	GTEX-WOFM-0626-SM-4OOS3
Esophagus - Mucosa	Esophagus	GTEX-WQUQ-1626-SM-4OOSH
Esophagus - Mucosa	Esophagus	GTEX-WRHK-0526-SM-4MVOF
Esophagus - Mucosa	Esophagus	GTEX-WVLH-1426-SM-4MVNX
Esophagus - Mucosa	Esophagus	GTEX-WXYG-1226-SM-4ONCJ
Esophagus - Mucosa	Esophagus	GTEX-WYJK-1926-SM-4ONDP
Esophagus - Mucosa	Esophagus	GTEX-WYVS-0726-SM-4ONDO
Esophagus - Mucosa	Esophagus	GTEX-X15G-0826-SM-4PQZD
Esophagus - Mucosa	Esophagus	GTEX-X3Y1-1326-SM-4PQZB
Esophagus - Mucosa	Esophagus	GTEX-X4EO-1226-SM-4QARR

Esophagus - Mucosa	Esophagus	GTEX-X4EP-1626-SM-4QAS6
Esophagus - Mucosa	Esophagus	GTEX-X4LF-0626-SM-4QASB
Esophagus - Mucosa	Esophagus	GTEX-X4XY-1726-SM-46MVO
Esophagus - Mucosa	Esophagus	GTEX-X585-1226-SM-46MW7
Esophagus - Mucosa	Esophagus	GTEX-X5EB-1526-SM-4E3JC
Esophagus - Mucosa	Esophagus	GTEX-X62O-2226-SM-46MW3
Esophagus - Mucosa	Esophagus	GTEX-XBED-1026-SM-48TCB
Esophagus - Mucosa	Esophagus	GTEX-XBEW-1326-SM-4AT63
Esophagus - Mucosa	Esophagus	GTEX-XGQ4-1526-SM-4AT6H
Esophagus - Mucosa	Esophagus	GTEX-XK95-1026-SM-4GIDV
Esophagus - Mucosa	Esophagus	GTEX-XLM4-2326-SM-4TT2D
Esophagus - Mucosa	Esophagus	GTEX-XMD2-1326-SM-4YCET
Esophagus - Mucosa	Esophagus	GTEX-XMK1-1326-SM-4B65Q
Esophagus - Mucosa	Esophagus	GTEX-XQ3S-1126-SM-4BOPK
Esophagus - Mucosa	Esophagus	GTEX-XQ8I-1526-SM-4BOOH
Esophagus - Mucosa	Esophagus	GTEX-XUZC-0426-SM-4BOPE
Esophagus - Mucosa	Esophagus	GTEX-XV7Q-1126-SM-4BRVS
Esophagus - Mucosa	Esophagus	GTEX-XXEK-0326-SM-4BRVV
Esophagus - Mucosa	Esophagus	GTEX-XYKS-1526-SM-4BRUP
Esophagus - Mucosa	Esophagus	GTEX-Y111-1626-SM-57WCY
Esophagus - Mucosa	Esophagus	GTEX-Y114-0926-SM-4TT7J
Esophagus - Mucosa	Esophagus	GTEX-Y3I4-1226-SM-4TT8N
Esophagus - Mucosa	Esophagus	GTEX-Y3IK-1126-SM-51MSV
Esophagus - Mucosa	Esophagus	GTEX-Y5LM-1026-SM-4VDSQ
Esophagus - Mucosa	Esophagus	GTEX-Y5V5-1626-SM-4VDSG
Esophagus - Mucosa	Esophagus	GTEX-Y5V6-1026-SM-4VBS2
Esophagus - Mucosa	Esophagus	GTEX-Y8E4-1726-SM-5LU9G
Esophagus - Mucosa	Esophagus	GTEX-Y8LW-0726-SM-5IFI1
Esophagus - Mucosa	Esophagus	GTEX-Y9LG-0626-SM-4WWDH
Esophagus - Mucosa	Esophagus	GTEX-YB5E-0926-SM-4WWDQ
Esophagus - Mucosa	Esophagus	GTEX-YB5K-0826-SM-5YYB7
Esophagus - Mucosa	Esophagus	GTEX-YEC3-2526-SM-4YCDN
Esophagus - Mucosa	Esophagus	GTEX-YEC4-1026-SM-5P9FW
Esophagus - Mucosa	Esophagus	GTEX-YECK-1626-SM-5IFHQ
Esophagus - Mucosa	Esophagus	GTEX-YF7O-0926-SM-5IFIF
Esophagus - Mucosa	Esophagus	GTEX-YFC4-1226-SM-5LU9R
Esophagus - Mucosa	Esophagus	GTEX-YJ89-1526-SM-5P9IR
Esophagus - Mucosa	Esophagus	GTEX-Z9EW-0526-SM-5HL8S
Esophagus - Mucosa	Esophagus	GTEX-ZA64-0626-SM-5HL9O
Esophagus - Mucosa	Esophagus	GTEX-ZAB4-1126-SM-5HL86
Esophagus - Mucosa	Esophagus	GTEX-ZAB5-0926-SM-5CVMV
Esophagus - Mucosa	Esophagus	GTEX-ZAJG-1326-SM-5S2NV
Esophagus - Mucosa	Esophagus	GTEX-ZAK1-1526-SM-5S2OV
Esophagus - Mucosa	Esophagus	GTEX-ZC5H-1326-SM-5HL7E
Esophagus - Mucosa	Esophagus	GTEX-ZDTT-1326-SM-4WKFH
Esophagus - Mucosa	Esophagus	GTEX-ZDYS-0826-SM-4WWCR
Esophagus - Mucosa	Esophagus	GTEX-ZE9C-1126-SM-4WWBD
Esophagus - Mucosa	Esophagus	GTEX-ZEX8-1426-SM-4WKHQ
Esophagus - Mucosa	Esophagus	GTEX-ZF29-1326-SM-4WWB6
Esophagus - Mucosa	Esophagus	GTEX-ZF2S-1226-SM-4WWAX
Esophagus - Mucosa	Esophagus	GTEX-ZF3C-1626-SM-4WWB4
Esophagus - Mucosa	Esophagus	GTEX-ZG7Y-1126-SM-4WWEZ
Esophagus - Mucosa	Esophagus	GTEX-ZGAY-1126-SM-4WWBS
Esophagus - Mucosa	Esophagus	GTEX-ZLV1-0526-SM-4WWD4

Esophagus - Mucosa	Esophagus	GTEX-ZPCL-1426-SM-57WEC
Esophagus - Mucosa	Esophagus	GTEX-ZPU1-1426-SM-4WWFU
Esophagus - Mucosa	Esophagus	GTEX-ZQUD-0426-SM-57WFR
Esophagus - Mucosa	Esophagus	GTEX-ZT9W-1326-SM-51MTB
Esophagus - Mucosa	Esophagus	GTEX-ZT9X-0526-SM-5GCOP
Esophagus - Mucosa	Esophagus	GTEX-ZTPG-1626-SM-5DUX2
Esophagus - Mucosa	Esophagus	GTEX-ZTSS-0626-SM-59867
Esophagus - Mucosa	Esophagus	GTEX-ZTX8-0226-SM-5E44D
Esophagus - Mucosa	Esophagus	GTEX-ZUA1-1226-SM-5NQ6O
Esophagus - Mucosa	Esophagus	GTEX-ZV68-0926-SM-59HK7
Esophagus - Mucosa	Esophagus	GTEX-ZVE2-1326-SM-5NQ94
Esophagus - Mucosa	Esophagus	GTEX-ZVP2-1326-SM-57WCE
Esophagus - Mucosa	Esophagus	GTEX-ZVT2-1326-SM-5E45H
Esophagus - Mucosa	Esophagus	GTEX-ZVT3-1426-SM-59HLP
Esophagus - Mucosa	Esophagus	GTEX-ZXES-1126-SM-5GZXN
Esophagus - Mucosa	Esophagus	GTEX-ZY6K-0626-SM-59HJL
Esophagus - Mucosa	Esophagus	GTEX-ZYFC-1626-SM-5N9EU
Esophagus - Mucosa	Esophagus	GTEX-ZYFG-1126-SM-5GICY
Esophagus - Mucosa	Esophagus	GTEX-ZYT6-1126-SM-5GZYR
Esophagus - Mucosa	Esophagus	GTEX-ZYVF-2226-SM-5GIDD
Esophagus - Mucosa	Esophagus	GTEX-ZYW4-1326-SM-5E45N
Esophagus - Mucosa	Esophagus	GTEX-ZYY3-1526-SM-5SIA9
Esophagus - Mucosa	Esophagus	GTEX-ZZ64-0226-SM-5E44X
Esophagus - Mucosa	Esophagus	GTEX-ZZPT-1426-SM-5N9C5
Esophagus - Muscularis	Esophagus	GTEX-111CU-0626-SM-5EGHL
Esophagus - Muscularis	Esophagus	GTEX-111YS-1026-SM-5GZYE
Esophagus - Muscularis	Esophagus	GTEX-1122O-1626-SM-5N9CX
Esophagus - Muscularis	Esophagus	GTEX-113JC-0926-SM-5H114
Esophagus - Muscularis	Esophagus	GTEX-117YW-2026-SM-5N9BH
Esophagus - Muscularis	Esophagus	GTEX-117YX-1826-SM-5H12K
Esophagus - Muscularis	Esophagus	GTEX-11DXX-1026-SM-5H11E
Esophagus - Muscularis	Esophagus	GTEX-11DXY-1526-SM-5H129
Esophagus - Muscularis	Esophagus	GTEX-11DYG-1426-SM-5A5JR
Esophagus - Muscularis	Esophagus	GTEX-11EM3-0726-SM-5GZZM
Esophagus - Muscularis	Esophagus	GTEX-11EQ9-0826-SM-5986Y
Esophagus - Muscularis	Esophagus	GTEX-11GS4-1426-SM-5N9D2
Esophagus - Muscularis	Esophagus	GTEX-11GSP-1726-SM-5A5LI
Esophagus - Muscularis	Esophagus	GTEX-11I78-1426-SM-5PNVY
Esophagus - Muscularis	Esophagus	GTEX-11LCK-1626-SM-5PNYB
Esophagus - Muscularis	Esophagus	GTEX-11NSD-1226-SM-5986Q
Esophagus - Muscularis	Esophagus	GTEX-11O72-1926-SM-5PNW1
Esophagus - Muscularis	Esophagus	GTEX-11OF3-2226-SM-5986F
Esophagus - Muscularis	Esophagus	GTEX-11ONC-1426-SM-5N9F4
Esophagus - Muscularis	Esophagus	GTEX-11P7K-1226-SM-5HL4X
Esophagus - Muscularis	Esophagus	GTEX-11P81-0926-SM-59874
Esophagus - Muscularis	Esophagus	GTEX-11P82-0626-SM-5PNYK
Esophagus - Muscularis	Esophagus	GTEX-11TT1-0426-SM-5EGI9
Esophagus - Muscularis	Esophagus	GTEX-11TUW-1226-SM-5GU7C
Esophagus - Muscularis	Esophagus	GTEX-11UD2-1226-SM-5EQMI
Esophagus - Muscularis	Esophagus	GTEX-11VI4-0626-SM-5EQLO
Esophagus - Muscularis	Esophagus	GTEX-11XUK-0726-SM-5EQMX
Esophagus - Muscularis	Esophagus	GTEX-11ZTT-0926-SM-5EQMS
Esophagus - Muscularis	Esophagus	GTEX-11ZUS-0926-SM-5FQSY
Esophagus - Muscularis	Esophagus	GTEX-1211K-1026-SM-5EQ4B

Esophagus - Muscularis	Esophagus	GTEX-1212Z-2026-SM-5N9EA
Esophagus - Muscularis	Esophagus	GTEX-12696-1426-SM-5EGJ6
Esophagus - Muscularis	Esophagus	GTEX-12BJ1-1426-SM-5BC5Q
Esophagus - Muscularis	Esophagus	GTEX-12C56-1126-SM-5FQTO
Esophagus - Muscularis	Esophagus	GTEX-12WSD-1926-SM-5GCOV
Esophagus - Muscularis	Esophagus	GTEX-12WSJ-0726-SM-5GCN6
Esophagus - Muscularis	Esophagus	GTEX-12WSL-1326-SM-5LZVW
Esophagus - Muscularis	Esophagus	GTEX-12WSN-5016-SM-7DUGY
Esophagus - Muscularis	Esophagus	GTEX-12ZZY-1526-SM-5LZWF
Esophagus - Muscularis	Esophagus	GTEX-13111-0926-SM-5DUVQ
Esophagus - Muscularis	Esophagus	GTEX-13113-1026-SM-5LZWM
Esophagus - Muscularis	Esophagus	GTEX-131XE-1226-SM-5EGKF
Esophagus - Muscularis	Esophagus	GTEX-131XF-0926-SM-5BC69
Esophagus - Muscularis	Esophagus	GTEX-131XG-0926-SM-5LZW5
Esophagus - Muscularis	Esophagus	GTEX-132AR-2326-SM-5IJDY
Esophagus - Muscularis	Esophagus	GTEX-132QS-1526-SM-5J2MV
Esophagus - Muscularis	Esophagus	GTEX-133LE-0726-SM-5P9IZ
Esophagus - Muscularis	Esophagus	GTEX-1399R-0726-SM-5KM2A
Esophagus - Muscularis	Esophagus	GTEX-1399T-1426-SM-664M8
Esophagus - Muscularis	Esophagus	GTEX-1399U-1226-SM-5K7XT
Esophagus - Muscularis	Esophagus	GTEX-139D8-1426-SM-5IJEX
Esophagus - Muscularis	Esophagus	GTEX-139T6-0726-SM-5PNVK
Esophagus - Muscularis	Esophagus	GTEX-139TU-1326-SM-5LZXW
Esophagus - Muscularis	Esophagus	GTEX-139YR-1626-SM-5IJGK
Esophagus - Muscularis	Esophagus	GTEX-13CF3-1526-SM-5J2NB
Esophagus - Muscularis	Esophagus	GTEX-13D11-1826-SM-5J2NM
Esophagus - Muscularis	Esophagus	GTEX-13FH7-0726-SM-5J2NK
Esophagus - Muscularis	Esophagus	GTEX-13FHO-1826-SM-5K7Y6
Esophagus - Muscularis	Esophagus	GTEX-13FHP-1626-SM-5N9FK
Esophagus - Muscularis	Esophagus	GTEX-13FLV-1226-SM-5IJBZ
Esophagus - Muscularis	Esophagus	GTEX-13FTW-1526-SM-5LZXC
Esophagus - Muscularis	Esophagus	GTEX-13G51-1026-SM-5K7YQ
Esophagus - Muscularis	Esophagus	GTEX-13NYB-0826-SM-5MR4V
Esophagus - Muscularis	Esophagus	GTEX-13O1R-1526-SM-5J1O7
Esophagus - Muscularis	Esophagus	GTEX-13O21-0826-SM-5J2NU
Esophagus - Muscularis	Esophagus	GTEX-13O3P-1326-SM-5K7Y8
Esophagus - Muscularis	Esophagus	GTEX-13O61-1026-SM-5L3F6
Esophagus - Muscularis	Esophagus	GTEX-13OVG-0926-SM-5J2OJ
Esophagus - Muscularis	Esophagus	GTEX-13OVI-2526-SM-5KM4V
Esophagus - Muscularis	Esophagus	GTEX-13OVJ-1326-SM-5K7Z9
Esophagus - Muscularis	Esophagus	GTEX-13OVK-1226-SM-6M473
Esophagus - Muscularis	Esophagus	GTEX-13OVL-1926-SM-5KM14
Esophagus - Muscularis	Esophagus	GTEX-13OW6-1426-SM-5L3EU
Esophagus - Muscularis	Esophagus	GTEX-13OW8-2226-SM-5K7Z4
Esophagus - Muscularis	Esophagus	GTEX-13PL7-0326-SM-5IJEK
Esophagus - Muscularis	Esophagus	GTEX-13PVQ-1826-SM-5L3GU
Esophagus - Muscularis	Esophagus	GTEX-13QBU-0926-SM-5K7WQ
Esophagus - Muscularis	Esophagus	GTEX-13QJ3-1826-SM-5S2PT
Esophagus - Muscularis	Esophagus	GTEX-13RTJ-1426-SM-793B3
Esophagus - Muscularis	Esophagus	GTEX-13RTK-0626-SM-5Q5EK
Esophagus - Muscularis	Esophagus	GTEX-13S86-0726-SM-5Q5DH
Esophagus - Muscularis	Esophagus	GTEX-13SLX-1926-SM-5ZZUR
Esophagus - Muscularis	Esophagus	GTEX-13U4I-1226-SM-5LU5M
Esophagus - Muscularis	Esophagus	GTEX-13W3W-1726-SM-5LU4A

Esophagus - Muscularis	Esophagus	GTEX-13X6H-1426-SM-5SI91
Esophagus - Muscularis	Esophagus	GTEX-13X6J-1626-SM-5Q5CV
Esophagus - Muscularis	Esophagus	GTEX-13X6K-2226-SM-79OMI
Esophagus - Muscularis	Esophagus	GTEX-13YAN-2026-SM-5Q5EY
Esophagus - Muscularis	Esophagus	GTEX-144GL-2226-SM-79OMD
Esophagus - Muscularis	Esophagus	GTEX-144GM-1526-SM-79OJM
Esophagus - Muscularis	Esophagus	GTEX-145LT-1226-SM-5Q5BZ
Esophagus - Muscularis	Esophagus	GTEX-145LV-1426-SM-5Q5BV
Esophagus - Muscularis	Esophagus	GTEX-145ME-5007-SM-7DHN8
Esophagus - Muscularis	Esophagus	GTEX-145MH-1226-SM-5RQJT
Esophagus - Muscularis	Esophagus	GTEX-145MN-1126-SM-5RQJB
Esophagus - Muscularis	Esophagus	GTEX-145MO-1526-SM-5QGQC
Esophagus - Muscularis	Esophagus	GTEX-146FH-1626-SM-5NQ9H
Esophagus - Muscularis	Esophagus	GTEX-146FQ-1126-SM-5NQBM
Esophagus - Muscularis	Esophagus	GTEX-146FR-0526-SM-5Q5EX
Esophagus - Muscularis	Esophagus	GTEX-14753-1926-SM-5NQAA
Esophagus - Muscularis	Esophagus	GTEX-147JS-1826-SM-5ZZWG
Esophagus - Muscularis	Esophagus	GTEX-148VI-1126-SM-5S2QW
Esophagus - Muscularis	Esophagus	GTEX-148VJ-1126-SM-5NQBZ
Esophagus - Muscularis	Esophagus	GTEX-1497J-2526-SM-5TDCE
Esophagus - Muscularis	Esophagus	GTEX-14AS3-0726-SM-5S2NY
Esophagus - Muscularis	Esophagus	GTEX-14B4R-0426-SM-5Q5EI
Esophagus - Muscularis	Esophagus	GTEX-14BIM-1226-SM-5SIBC
Esophagus - Muscularis	Esophagus	GTEX-14BIN-2626-SM-5YY8U
Esophagus - Muscularis	Esophagus	GTEX-14BMU-0826-SM-73KZ8
Esophagus - Muscularis	Esophagus	GTEX-14BMV-1426-SM-5S2WA
Esophagus - Muscularis	Esophagus	GTEX-14C38-2226-SM-793AW
Esophagus - Muscularis	Esophagus	GTEX-14C5O-1726-SM-5RQI9
Esophagus - Muscularis	Esophagus	GTEX-14DAQ-1526-SM-5ZZW8
Esophagus - Muscularis	Esophagus	GTEX-14DAR-0626-SM-664MY
Esophagus - Muscularis	Esophagus	GTEX-14E1K-1126-SM-73KV4
Esophagus - Muscularis	Esophagus	GTEX-14E6C-0926-SM-5S2V9
Esophagus - Muscularis	Esophagus	GTEX-14E6E-0626-SM-73KX6
Esophagus - Muscularis	Esophagus	GTEX-14E7W-1826-SM-69LQ1
Esophagus - Muscularis	Esophagus	GTEX-14H4A-1726-SM-5ZZUV
Esophagus - Muscularis	Esophagus	GTEX-14ICL-0626-SM-5ZZUO
Esophagus - Muscularis	Esophagus	GTEX-14PHX-0826-SM-69LPO
Esophagus - Muscularis	Esophagus	GTEX-14PHY-0726-SM-5YY8X
Esophagus - Muscularis	Esophagus	GTEX-14PJ2-2726-SM-5ZZVZ
Esophagus - Muscularis	Esophagus	GTEX-14PJ3-0826-SM-6AJBR
Esophagus - Muscularis	Esophagus	GTEX-14PJ4-1426-SM-69LO6
Esophagus - Muscularis	Esophagus	GTEX-14PJ5-0626-SM-6LLIJ
Esophagus - Muscularis	Esophagus	GTEX-14PJ6-1126-SM-6AJ9R
Esophagus - Muscularis	Esophagus	GTEX-14PJM-1626-SM-6LLHL
Esophagus - Muscularis	Esophagus	GTEX-14PK6-1126-SM-5YY93
Esophagus - Muscularis	Esophagus	GTEX-14PKU-0926-SM-69LPS
Esophagus - Muscularis	Esophagus	GTEX-14PKV-1526-SM-664O2
Esophagus - Muscularis	Esophagus	GTEX-14PN3-1126-SM-69LOR
Esophagus - Muscularis	Esophagus	GTEX-14XAO-1426-SM-6EU2C
Esophagus - Muscularis	Esophagus	GTEX-15CHC-1426-SM-5ZZVQ
Esophagus - Muscularis	Esophagus	GTEX-15CHQ-2026-SM-6AJAX
Esophagus - Muscularis	Esophagus	GTEX-15CHR-0926-SM-7DUFW
Esophagus - Muscularis	Esophagus	GTEX-15DCD-1726-SM-7KUN1
Esophagus - Muscularis	Esophagus	GTEX-15DCZ-2526-SM-69LOC

Esophagus - Muscularis	Esophagus	GTEX-15DYW-1926-SM-6EU2K
Esophagus - Muscularis	Esophagus	GTEX-15DZA-0526-SM-6LPKE
Esophagus - Muscularis	Esophagus	GTEX-15EOM-1926-SM-69LQ2
Esophagus - Muscularis	Esophagus	GTEX-15ER7-2226-SM-7KFRN
Esophagus - Muscularis	Esophagus	GTEX-15ETS-2026-SM-6AJBC
Esophagus - Muscularis	Esophagus	GTEX-15EU6-1526-SM-7KUGA
Esophagus - Muscularis	Esophagus	GTEX-15FZZ-1126-SM-6AJBG
Esophagus - Muscularis	Esophagus	GTEX-15G1A-0526-SM-7KUKM
Esophagus - Muscularis	Esophagus	GTEX-15RIF-0926-SM-7KUKP
Esophagus - Muscularis	Esophagus	GTEX-15SB6-0826-SM-7KUMK
Esophagus - Muscularis	Esophagus	GTEX-15SDE-0726-SM-6M48Q
Esophagus - Muscularis	Esophagus	GTEX-15SHU-1526-SM-7KUF5
Esophagus - Muscularis	Esophagus	GTEX-15SHV-1126-SM-6PALO
Esophagus - Muscularis	Esophagus	GTEX-15SHW-1926-SM-7KUKU
Esophagus - Muscularis	Esophagus	GTEX-15UF6-2426-SM-7KUME
Esophagus - Muscularis	Esophagus	GTEX-15UKP-2026-SM-6LPIC
Esophagus - Muscularis	Esophagus	GTEX-16AAH-0926-SM-7939B
Esophagus - Muscularis	Esophagus	GTEX-16BQI-1626-SM-7KULR
Esophagus - Muscularis	Esophagus	GTEX-16NGA-1326-SM-718AH
Esophagus - Muscularis	Esophagus	GTEX-16NPV-1526-SM-7KUL6
Esophagus - Muscularis	Esophagus	GTEX-16NPX-1726-SM-7KULP
Esophagus - Muscularis	Esophagus	GTEX-16Z82-2026-SM-6M48B
Esophagus - Muscularis	Esophagus	GTEX-178AV-0926-SM-793BK
Esophagus - Muscularis	Esophagus	GTEX-17EUY-1326-SM-7DHKZ
Esophagus - Muscularis	Esophagus	GTEX-17F97-1426-SM-7LG5C
Esophagus - Muscularis	Esophagus	GTEX-17F98-1926-SM-7DHLW
Esophagus - Muscularis	Esophagus	GTEX-17F9E-2126-SM-7DUFG
Esophagus - Muscularis	Esophagus	GTEX-17F9Y-1826-SM-7IGMI
Esophagus - Muscularis	Esophagus	GTEX-17HGU-1526-SM-7EWE4
Esophagus - Muscularis	Esophagus	GTEX-17HHE-0726-SM-7DHL7
Esophagus - Muscularis	Esophagus	GTEX-17HHY-1726-SM-7EWD7
Esophagus - Muscularis	Esophagus	GTEX-17KNJ-1226-SM-7LT9M
Esophagus - Muscularis	Esophagus	GTEX-17MFQ-0626-SM-793A7
Esophagus - Muscularis	Esophagus	GTEX-183FY-1326-SM-7DHLL
Esophagus - Muscularis	Esophagus	GTEX-18A66-1526-SM-72D6Y
Esophagus - Muscularis	Esophagus	GTEX-18A67-2126-SM-7LT9Y
Esophagus - Muscularis	Esophagus	GTEX-18A6Q-1326-SM-7LG62
Esophagus - Muscularis	Esophagus	GTEX-18D9A-0326-SM-7LT8L
Esophagus - Muscularis	Esophagus	GTEX-18D9B-2126-SM-7LTAQ
Esophagus - Muscularis	Esophagus	GTEX-18D9U-1726-SM-7LTA5
Esophagus - Muscularis	Esophagus	GTEX-18QFQ-1126-SM-72D6R
Esophagus - Muscularis	Esophagus	GTEX-1A3MV-1526-SM-731BO
Esophagus - Muscularis	Esophagus	GTEX-1A8FM-1426-SM-7IGPD
Esophagus - Muscularis	Esophagus	GTEX-1A8G6-1226-SM-73KVH
Esophagus - Muscularis	Esophagus	GTEX-1AMEY-0326-SM-72D5T
Esophagus - Muscularis	Esophagus	GTEX-1AMFI-0326-SM-731CU
Esophagus - Muscularis	Esophagus	GTEX-1AX8Z-1926-SM-72D7B
Esophagus - Muscularis	Esophagus	GTEX-1AX9I-1526-SM-72D53
Esophagus - Muscularis	Esophagus	GTEX-1AX9J-1926-SM-73KUM
Esophagus - Muscularis	Esophagus	GTEX-1AX9K-1226-SM-793CN
Esophagus - Muscularis	Esophagus	GTEX-1AYCT-1526-SM-73KWC
Esophagus - Muscularis	Esophagus	GTEX-1B8KE-1626-SM-793CK
Esophagus - Muscularis	Esophagus	GTEX-1B8KZ-0826-SM-73KYB
Esophagus - Muscularis	Esophagus	GTEX-1B932-2626-SM-73KWR

Esophagus - Muscularis	Esophagus	GTEX-1B933-1926-SM-73KUN
Esophagus - Muscularis	Esophagus	GTEX-1B97I-1026-SM-73KVC
Esophagus - Muscularis	Esophagus	GTEX-1B97J-0926-SM-79ONU
Esophagus - Muscularis	Esophagus	GTEX-1B996-1626-SM-7DHLZ
Esophagus - Muscularis	Esophagus	GTEX-1C2JI-1226-SM-7DHM2
Esophagus - Muscularis	Esophagus	GTEX-1C475-0726-SM-73KVL
Esophagus - Muscularis	Esophagus	GTEX-1C64O-1926-SM-79ONP
Esophagus - Muscularis	Esophagus	GTEX-1CB4E-1126-SM-79OOM
Esophagus - Muscularis	Esophagus	GTEX-1CB4F-1426-SM-793DA
Esophagus - Muscularis	Esophagus	GTEX-1CB4J-2226-SM-7EWF5
Esophagus - Muscularis	Esophagus	GTEX-1E1VI-1626-SM-7EPIL
Esophagus - Muscularis	Esophagus	GTEX-1EH9U-2526-SM-79OO5
Esophagus - Muscularis	Esophagus	GTEX-NFK9-1226-SM-3LK79
Esophagus - Muscularis	Esophagus	GTEX-NPJ8-2026-SM-3TW91
Esophagus - Muscularis	Esophagus	GTEX-O5YT-0726-SM-3MJHA
Esophagus - Muscularis	Esophagus	GTEX-OHPM-0726-SM-3LK7A
Esophagus - Muscularis	Esophagus	GTEX-OIZG-0926-SM-3LK5Y
Esophagus - Muscularis	Esophagus	GTEX-OIZH-0726-SM-3NB1I
Esophagus - Muscularis	Esophagus	GTEX-OIZI-1326-SM-3NB1B
Esophagus - Muscularis	Esophagus	GTEX-OOBK-0726-SM-3LK5Q
Esophagus - Muscularis	Esophagus	GTEX-OXRK-1426-SM-3NB19
Esophagus - Muscularis	Esophagus	GTEX-OXRL-0726-SM-3NM9A
Esophagus - Muscularis	Esophagus	GTEX-P4PP-0726-SM-3NM9S
Esophagus - Muscularis	Esophagus	GTEX-P4PQ-0726-SM-3NMCR
Esophagus - Muscularis	Esophagus	GTEX-P4QS-0726-SM-3NMCZ
Esophagus - Muscularis	Esophagus	GTEX-P4QT-0726-SM-3NMCM
Esophagus - Muscularis	Esophagus	GTEX-P78B-1126-SM-3P615
Esophagus - Muscularis	Esophagus	GTEX-PLZ5-0826-SM-3P5ZW
Esophagus - Muscularis	Esophagus	GTEX-PLZ6-0626-SM-3P61B
Esophagus - Muscularis	Esophagus	GTEX-POMQ-0626-SM-3P61E
Esophagus - Muscularis	Esophagus	GTEX-PSDG-1426-SM-48TD1
Esophagus - Muscularis	Esophagus	GTEX-PWCY-0826-SM-48TCR
Esophagus - Muscularis	Esophagus	GTEX-PWN1-0726-SM-48TDR
Esophagus - Muscularis	Esophagus	GTEX-PX3G-0726-SM-48TZT
Esophagus - Muscularis	Esophagus	GTEX-Q2AG-1226-SM-4GICA
Esophagus - Muscularis	Esophagus	GTEX-Q2AH-1026-SM-48TZI
Esophagus - Muscularis	Esophagus	GTEX-Q734-0826-SM-48U1G
Esophagus - Muscularis	Esophagus	GTEX-QCQG-0726-SM-48U1Z
Esophagus - Muscularis	Esophagus	GTEX-QDT8-1326-SM-48TYY
Esophagus - Muscularis	Esophagus	GTEX-QDVJ-0826-SM-48U1S
Esophagus - Muscularis	Esophagus	GTEX-QDVN-1126-SM-48TZ7
Esophagus - Muscularis	Esophagus	GTEX-QEG5-1326-SM-4R1JS
Esophagus - Muscularis	Esophagus	GTEX-QEL4-1526-SM-447AB
Esophagus - Muscularis	Esophagus	GTEX-QESD-0726-SM-4R1KQ
Esophagus - Muscularis	Esophagus	GTEX-QLQ7-1026-SM-447BB
Esophagus - Muscularis	Esophagus	GTEX-QLQW-0626-SM-447A6
Esophagus - Muscularis	Esophagus	GTEX-QMRM-0926-SM-447BR
Esophagus - Muscularis	Esophagus	GTEX-QV44-1026-SM-447CG
Esophagus - Muscularis	Esophagus	GTEX-R53T-1026-SM-48FCO
Esophagus - Muscularis	Esophagus	GTEX-R55C-0726-SM-48FCN
Esophagus - Muscularis	Esophagus	GTEX-R55D-1226-SM-48FE9
Esophagus - Muscularis	Esophagus	GTEX-R55G-0926-SM-48FDN
Esophagus - Muscularis	Esophagus	GTEX-REY6-1426-SM-48FDK
Esophagus - Muscularis	Esophagus	GTEX-RM2N-0626-SM-48FD6

Esophagus - Muscularis	Esophagus	GTEX-RNOR-1326-SM-48FDE
Esophagus - Muscularis	Esophagus	GTEX-RTLS-1226-SM-46MUP
Esophagus - Muscularis	Esophagus	GTEX-RU1J-0426-SM-46MUK
Esophagus - Muscularis	Esophagus	GTEX-RU72-0826-SM-46MUS
Esophagus - Muscularis	Esophagus	GTEX-RUSQ-0926-SM-47JWU
Esophagus - Muscularis	Esophagus	GTEX-RWS6-0726-SM-47JXI
Esophagus - Muscularis	Esophagus	GTEX-RWSA-1326-SM-47JX9
Esophagus - Muscularis	Esophagus	GTEX-S32W-1026-SM-4AD5W
Esophagus - Muscularis	Esophagus	GTEX-S341-0426-SM-4AD5L
Esophagus - Muscularis	Esophagus	GTEX-S3XE-0826-SM-4AD4U
Esophagus - Muscularis	Esophagus	GTEX-S4P3-0526-SM-4AD58
Esophagus - Muscularis	Esophagus	GTEX-S4Q7-0526-SM-4AD5N
Esophagus - Muscularis	Esophagus	GTEX-S4Z8-0726-SM-4GICB
Esophagus - Muscularis	Esophagus	GTEX-S7SF-1026-SM-4AD4I
Esophagus - Muscularis	Esophagus	GTEX-S95S-0326-SM-4B66U
Esophagus - Muscularis	Esophagus	GTEX-SE5C-1326-SM-4BRUH
Esophagus - Muscularis	Esophagus	GTEX-SNMC-0426-SM-4DM5Y
Esophagus - Muscularis	Esophagus	GTEX-SNOS-0726-SM-4DM5Z
Esophagus - Muscularis	Esophagus	GTEX-T2IS-0926-SM-4DM5B
Esophagus - Muscularis	Esophagus	GTEX-T5JC-1826-SM-4DM6E
Esophagus - Muscularis	Esophagus	GTEX-T5JW-1426-SM-4DM5Q
Esophagus - Muscularis	Esophagus	GTEX-T6MN-1526-SM-4DM5P
Esophagus - Muscularis	Esophagus	GTEX-T6MO-0626-SM-4DM6P
Esophagus - Muscularis	Esophagus	GTEX-T8EM-0626-SM-4DM62
Esophagus - Muscularis	Esophagus	GTEX-TKQ1-0426-SM-4DXT4
Esophagus - Muscularis	Esophagus	GTEX-TKQ2-1226-SM-4DXSV
Esophagus - Muscularis	Esophagus	GTEX-TML8-1426-SM-4DXUT
Esophagus - Muscularis	Esophagus	GTEX-TMMY-1226-SM-4DXT6
Esophagus - Muscularis	Esophagus	GTEX-U3ZH-1126-SM-4DXUG
Esophagus - Muscularis	Esophagus	GTEX-U3ZM-0626-SM-4DXTV
Esophagus - Muscularis	Esophagus	GTEX-U3ZN-1826-SM-4DXUY
Esophagus - Muscularis	Esophagus	GTEX-U4B1-0926-SM-4DXUV
Esophagus - Muscularis	Esophagus	GTEX-U8XE-1826-SM-4E3HV
Esophagus - Muscularis	Esophagus	GTEX-UJHI-0926-SM-4IHKF
Esophagus - Muscularis	Esophagus	GTEX-UJMC-1026-SM-4IHKN
Esophagus - Muscularis	Esophagus	GTEX-UPK5-1326-SM-4IHLE
Esophagus - Muscularis	Esophagus	GTEX-V1D1-1126-SM-4JBHT
Esophagus - Muscularis	Esophagus	GTEX-V955-1026-SM-4JBHO
Esophagus - Muscularis	Esophagus	GTEX-VJYA-0626-SM-4KL1S
Esophagus - Muscularis	Esophagus	GTEX-VUSG-1126-SM-4KKZQ
Esophagus - Muscularis	Esophagus	GTEX-W5WG-1526-SM-4LMIG
Esophagus - Muscularis	Esophagus	GTEX-WEY5-1026-SM-4LMK9
Esophagus - Muscularis	Esophagus	GTEX-WFG7-1026-SM-4LMK8
Esophagus - Muscularis	Esophagus	GTEX-WFG8-1026-SM-4LVMZ
Esophagus - Muscularis	Esophagus	GTEX-WFJO-0726-SM-4LVM8
Esophagus - Muscularis	Esophagus	GTEX-WFON-0926-SM-4LVMK
Esophagus - Muscularis	Esophagus	GTEX-WH7G-1026-SM-4LVML
Esophagus - Muscularis	Esophagus	GTEX-WHPG-1226-SM-4M1ZI
Esophagus - Muscularis	Esophagus	GTEX-WHSB-0526-SM-4M1XM
Esophagus - Muscularis	Esophagus	GTEX-WHSE-1626-SM-4M1XL
Esophagus - Muscularis	Esophagus	GTEX-WHWD-1026-SM-4OORZ
Esophagus - Muscularis	Esophagus	GTEX-WK11-1126-SM-4OOSJ
Esophagus - Muscularis	Esophagus	GTEX-WOFM-0526-SM-4OOS2
Esophagus - Muscularis	Esophagus	GTEX-WRHK-0426-SM-4MVOG

Esophagus - Muscularis	Esophagus	GTEX-WXYG-1126-SM-4ONCH
Esophagus - Muscularis	Esophagus	GTEX-WY7C-1326-SM-4ONCX
Esophagus - Muscularis	Esophagus	GTEX-WYJK-2026-SM-4ONDE
Esophagus - Muscularis	Esophagus	GTEX-WYVS-0826-SM-4RTXB
Esophagus - Muscularis	Esophagus	GTEX-WZTO-1526-SM-4PQYQ
Esophagus - Muscularis	Esophagus	GTEX-X15G-0926-SM-4PQZE
Esophagus - Muscularis	Esophagus	GTEX-X3Y1-1226-SM-4PQZ8
Esophagus - Muscularis	Esophagus	GTEX-X5EB-1426-SM-46MVW
Esophagus - Muscularis	Esophagus	GTEX-X8HC-1526-SM-46MWD
Esophagus - Muscularis	Esophagus	GTEX-XBED-0926-SM-48TCE
Esophagus - Muscularis	Esophagus	GTEX-XBEW-1426-SM-4AT4J
Esophagus - Muscularis	Esophagus	GTEX-XPVG-1326-SM-4B65P
Esophagus - Muscularis	Esophagus	GTEX-XQ3S-1226-SM-4BOPP
Esophagus - Muscularis	Esophagus	GTEX-XQ8I-1626-SM-4BOOI
Esophagus - Muscularis	Esophagus	GTEX-XUW1-1526-SM-4BOOY
Esophagus - Muscularis	Esophagus	GTEX-XUZC-0326-SM-4BOO8
Esophagus - Muscularis	Esophagus	GTEX-XV7Q-1026-SM-4BRVR
Esophagus - Muscularis	Esophagus	GTEX-XXEK-0426-SM-4BRVW
Esophagus - Muscularis	Esophagus	GTEX-XYKS-1126-SM-4BRWP
Esophagus - Muscularis	Esophagus	GTEX-Y111-1726-SM-4SOIQ
Esophagus - Muscularis	Esophagus	GTEX-Y3I4-1026-SM-4TT2C
Esophagus - Muscularis	Esophagus	GTEX-Y3IK-1226-SM-4WWEK
Esophagus - Muscularis	Esophagus	GTEX-Y5LM-1126-SM-4VDT5
Esophagus - Muscularis	Esophagus	GTEX-Y5V6-1126-SM-4VDTD
Esophagus - Muscularis	Esophagus	GTEX-Y8E4-1826-SM-4VBPV
Esophagus - Muscularis	Esophagus	GTEX-Y8LW-0626-SM-4VDS8
Esophagus - Muscularis	Esophagus	GTEX-Y9LG-0826-SM-5ZZVG
Esophagus - Muscularis	Esophagus	GTEX-YB5E-0826-SM-4VDRZ
Esophagus - Muscularis	Esophagus	GTEX-YB5K-0726-SM-5LU8R
Esophagus - Muscularis	Esophagus	GTEX-YEC3-1926-SM-4WWF5
Esophagus - Muscularis	Esophagus	GTEX-YEC4-1126-SM-5CVLW
Esophagus - Muscularis	Esophagus	GTEX-YECK-1726-SM-5IFI3
Esophagus - Muscularis	Esophagus	GTEX-YF7O-0826-SM-4W21F
Esophagus - Muscularis	Esophagus	GTEX-YFCO-0526-SM-4W1ZJ
Esophagus - Muscularis	Esophagus	GTEX-YJ8A-1226-SM-5IFHH
Esophagus - Muscularis	Esophagus	GTEX-Z9EW-0626-SM-5HL95
Esophagus - Muscularis	Esophagus	GTEX-ZA64-0526-SM-5HL9I
Esophagus - Muscularis	Esophagus	GTEX-ZAB4-1226-SM-5CVN5
Esophagus - Muscularis	Esophagus	GTEX-ZAB5-1026-SM-5HL91
Esophagus - Muscularis	Esophagus	GTEX-ZDTT-1226-SM-4WKG9
Esophagus - Muscularis	Esophagus	GTEX-ZDYS-0926-SM-4WWCS
Esophagus - Muscularis	Esophagus	GTEX-ZEX8-1326-SM-4WWEW
Esophagus - Muscularis	Esophagus	GTEX-ZF29-1226-SM-4WKH1
Esophagus - Muscularis	Esophagus	GTEX-ZF2S-1126-SM-4WKHH
Esophagus - Muscularis	Esophagus	GTEX-ZG7Y-1326-SM-4WWBL
Esophagus - Muscularis	Esophagus	GTEX-ZGAY-1226-SM-4YCF4
Esophagus - Muscularis	Esophagus	GTEX-ZLFU-1026-SM-5EQMZ
Esophagus - Muscularis	Esophagus	GTEX-ZLV1-0626-SM-4WWF6
Esophagus - Muscularis	Esophagus	GTEX-ZLWG-1226-SM-4WWFS
Esophagus - Muscularis	Esophagus	GTEX-ZP4G-0926-SM-4WWF4
Esophagus - Muscularis	Esophagus	GTEX-ZPCL-1226-SM-4WWFF
Esophagus - Muscularis	Esophagus	GTEX-ZPIC-1626-SM-57WEB
Esophagus - Muscularis	Esophagus	GTEX-ZPU1-1326-SM-57WE1
Esophagus - Muscularis	Esophagus	GTEX-ZQUD-0326-SM-4YCEN

Esophagus - Muscularis	Esophagus	GTEX-ZT9W-1226-SM-57WE4
Esophagus - Muscularis	Esophagus	GTEX-ZTPG-1526-SM-51MTF
Esophagus - Muscularis	Esophagus	GTEX-ZTSS-0726-SM-51MSN
Esophagus - Muscularis	Esophagus	GTEX-ZTTD-1426-SM-4YCEX
Esophagus - Muscularis	Esophagus	GTEX-ZTX8-0326-SM-59HLA
Esophagus - Muscularis	Esophagus	GTEX-ZVT2-1226-SM-5GU5H
Esophagus - Muscularis	Esophagus	GTEX-ZVZP-1426-SM-5NQ7R
Esophagus - Muscularis	Esophagus	GTEX-ZXES-1226-SM-5NQ8G
Esophagus - Muscularis	Esophagus	GTEX-ZY6K-0526-SM-5GZY9
Esophagus - Muscularis	Esophagus	GTEX-ZYFC-1726-SM-5GZYX
Esophagus - Muscularis	Esophagus	GTEX-ZYT6-1226-SM-5E44Q
Esophagus - Muscularis	Esophagus	GTEX-ZYVF-2326-SM-5E44Z
Esophagus - Muscularis	Esophagus	GTEX-ZYW4-1426-SM-5SIAX
Esophagus - Muscularis	Esophagus	GTEX-ZYY3-1626-SM-5EGJF
Esophagus - Muscularis	Esophagus	GTEX-ZZ64-0326-SM-5E45S
Esophagus - Muscularis	Esophagus	GTEX-ZZPU-1826-SM-5E43L
Fallopian Tube	Fallopian Tube	GTEX-OHPK-2326-SM-3MJH2
Fallopian Tube	Fallopian Tube	GTEX-S32W-1326-SM-4AD5Q
Fallopian Tube	Fallopian Tube	GTEX-S341-0826-SM-4AD73
Fallopian Tube	Fallopian Tube	GTEX-SE5C-0926-SM-4BRUF
Fallopian Tube	Fallopian Tube	GTEX-T5JW-0326-SM-4DM6J
Fallopian Tube	Fallopian Tube	GTEX-T6MO-1026-SM-4DM72
Fallopian Tube	Fallopian Tube	GTEX-U3ZN-1126-SM-4DXUL
Heart - Atrial Appendage	Heart	GTEX-1117F-0726-SM-5GIEN
Heart - Atrial Appendage	Heart	GTEX-111FC-0626-SM-5N9CU
Heart - Atrial Appendage	Heart	GTEX-111VG-0326-SM-5GZX7
Heart - Atrial Appendage	Heart	GTEX-111YS-0326-SM-5GZZ3
Heart - Atrial Appendage	Heart	GTEX-1122O-0626-SM-5N9B9
Heart - Atrial Appendage	Heart	GTEX-117XS-0726-SM-5H131
Heart - Atrial Appendage	Heart	GTEX-117YW-0426-SM-5GZZZ
Heart - Atrial Appendage	Heart	GTEX-117YX-0626-SM-5EGJI
Heart - Atrial Appendage	Heart	GTEX-1192W-0226-SM-5EGGT
Heart - Atrial Appendage	Heart	GTEX-1192X-0726-SM-5987R
Heart - Atrial Appendage	Heart	GTEX-11DXX-0526-SM-5PNVR
Heart - Atrial Appendage	Heart	GTEX-11DXY-1026-SM-5987V
Heart - Atrial Appendage	Heart	GTEX-11DXZ-0326-SM-5EGH1
Heart - Atrial Appendage	Heart	GTEX-11DYG-1026-SM-5A5JQ
Heart - Atrial Appendage	Heart	GTEX-11EM3-0426-SM-5N9BZ
Heart - Atrial Appendage	Heart	GTEX-11EMC-0826-SM-59862
Heart - Atrial Appendage	Heart	GTEX-11GS4-0426-SM-5N9CD
Heart - Atrial Appendage	Heart	GTEX-11GSP-1226-SM-5985M
Heart - Atrial Appendage	Heart	GTEX-11LCK-0826-SM-5PNYD
Heart - Atrial Appendage	Heart	GTEX-11NUK-0926-SM-5HL57
Heart - Atrial Appendage	Heart	GTEX-11NV4-0826-SM-5BC4S
Heart - Atrial Appendage	Heart	GTEX-11O72-1026-SM-5986B
Heart - Atrial Appendage	Heart	GTEX-11ONC-1026-SM-5GU64
Heart - Atrial Appendage	Heart	GTEX-11P81-0526-SM-59873
Heart - Atrial Appendage	Heart	GTEX-11TT1-1426-SM-5EGIA
Heart - Atrial Appendage	Heart	GTEX-11TUW-1126-SM-5EQKJ
Heart - Atrial Appendage	Heart	GTEX-11UD2-0926-SM-5CVL6
Heart - Atrial Appendage	Heart	GTEX-11WQK-0226-SM-5EQLI
Heart - Atrial Appendage	Heart	GTEX-11ZTT-0526-SM-5EQLA
Heart - Atrial Appendage	Heart	GTEX-11ZUS-0326-SM-5EQ4W
Heart - Atrial Appendage	Heart	GTEX-11ZVC-0526-SM-5N9G1

Heart - Atrial Appendage	Heart	GTEX-1211K-0226-SM-59HJY
Heart - Atrial Appendage	Heart	GTEX-1212Z-0726-SM-5EGI5
Heart - Atrial Appendage	Heart	GTEX-12584-1026-SM-59HK3
Heart - Atrial Appendage	Heart	GTEX-12696-1226-SM-5FQSM
Heart - Atrial Appendage	Heart	GTEX-12BJ1-0326-SM-5FQUB
Heart - Atrial Appendage	Heart	GTEX-12WSA-1126-SM-5EGKU
Heart - Atrial Appendage	Heart	GTEX-12WSC-1026-SM-5EQ5Y
Heart - Atrial Appendage	Heart	GTEX-12WSD-1226-SM-5HL9Q
Heart - Atrial Appendage	Heart	GTEX-12WSE-0926-SM-5S2VX
Heart - Atrial Appendage	Heart	GTEX-12WSG-1226-SM-5EQ4C
Heart - Atrial Appendage	Heart	GTEX-12WSI-0426-SM-5EQ5J
Heart - Atrial Appendage	Heart	GTEX-12WSK-0526-SM-5CVNA
Heart - Atrial Appendage	Heart	GTEX-12WSN-0226-SM-5DUXH
Heart - Atrial Appendage	Heart	GTEX-12ZZX-0726-SM-5EGKA
Heart - Atrial Appendage	Heart	GTEX-12ZZY-1226-SM-5GCNK
Heart - Atrial Appendage	Heart	GTEX-13112-0526-SM-5EQ4S
Heart - Atrial Appendage	Heart	GTEX-1313W-1126-SM-5EQ5U
Heart - Atrial Appendage	Heart	GTEX-131XE-0526-SM-5K7YT
Heart - Atrial Appendage	Heart	GTEX-131XF-0426-SM-5HL7U
Heart - Atrial Appendage	Heart	GTEX-131XG-0526-SM-5DUWF
Heart - Atrial Appendage	Heart	GTEX-131YS-0826-SM-5PNYV
Heart - Atrial Appendage	Heart	GTEX-132AR-1926-SM-5EGK5
Heart - Atrial Appendage	Heart	GTEX-132NY-1426-SM-5J1MJ
Heart - Atrial Appendage	Heart	GTEX-132Q8-1126-SM-5K7XS
Heart - Atrial Appendage	Heart	GTEX-132QS-0526-SM-5IJC7
Heart - Atrial Appendage	Heart	GTEX-1339X-0326-SM-5K7Z8
Heart - Atrial Appendage	Heart	GTEX-1399R-1726-SM-5K7YJ
Heart - Atrial Appendage	Heart	GTEX-1399S-0826-SM-5KM23
Heart - Atrial Appendage	Heart	GTEX-1399T-0726-SM-5J1MH
Heart - Atrial Appendage	Heart	GTEX-1399U-0726-SM-5KM1D
Heart - Atrial Appendage	Heart	GTEX-139TU-1926-SM-5J2OC
Heart - Atrial Appendage	Heart	GTEX-139YR-0526-SM-5L3DG
Heart - Atrial Appendage	Heart	GTEX-13CF2-0226-SM-5L3DV
Heart - Atrial Appendage	Heart	GTEX-13D11-1526-SM-5J2NA
Heart - Atrial Appendage	Heart	GTEX-13FH7-0826-SM-5J2NW
Heart - Atrial Appendage	Heart	GTEX-13FHO-1226-SM-5L3EF
Heart - Atrial Appendage	Heart	GTEX-13FHP-0826-SM-5K7V5
Heart - Atrial Appendage	Heart	GTEX-13FTW-0826-SM-5K7XR
Heart - Atrial Appendage	Heart	GTEX-13FTZ-0326-SM-5J1O1
Heart - Atrial Appendage	Heart	GTEX-13FXS-1026-SM-62LFQ
Heart - Atrial Appendage	Heart	GTEX-13JUV-0226-SM-5IJCC
Heart - Atrial Appendage	Heart	GTEX-13JVG-1126-SM-5KM2M
Heart - Atrial Appendage	Heart	GTEX-13N11-0726-SM-5L3DP
Heart - Atrial Appendage	Heart	GTEX-13N1W-0926-SM-5MR36
Heart - Atrial Appendage	Heart	GTEX-13NYB-0326-SM-5IJDP
Heart - Atrial Appendage	Heart	GTEX-13NYS-1926-SM-5IJCB
Heart - Atrial Appendage	Heart	GTEX-13NZ9-0126-SM-5K7XV
Heart - Atrial Appendage	Heart	GTEX-13NZA-0826-SM-5K7WS
Heart - Atrial Appendage	Heart	GTEX-13NZB-0426-SM-5KM26
Heart - Atrial Appendage	Heart	GTEX-13O1R-0926-SM-5L3DS
Heart - Atrial Appendage	Heart	GTEX-13O21-0326-SM-5J1N9
Heart - Atrial Appendage	Heart	GTEX-13O3O-1426-SM-5KM2S
Heart - Atrial Appendage	Heart	GTEX-13O3P-1426-SM-5L3DT
Heart - Atrial Appendage	Heart	GTEX-13O3Q-0326-SM-5L3FE

Heart - Atrial Appendage	Heart	GTEX-13O61-0426-SM-5L3ET
Heart - Atrial Appendage	Heart	GTEX-13OVG-0426-SM-5KM2T
Heart - Atrial Appendage	Heart	GTEX-13OVI-0326-SM-5K7X1
Heart - Atrial Appendage	Heart	GTEX-13OW5-0926-SM-5L3GZ
Heart - Atrial Appendage	Heart	GTEX-13OW6-0926-SM-5L3GM
Heart - Atrial Appendage	Heart	GTEX-13OW7-1626-SM-5IJDH
Heart - Atrial Appendage	Heart	GTEX-13PL6-0826-SM-5IJBI
Heart - Atrial Appendage	Heart	GTEX-13PL7-0726-SM-5KM1S
Heart - Atrial Appendage	Heart	GTEX-13PVQ-1326-SM-5LU4J
Heart - Atrial Appendage	Heart	GTEX-13PVR-0926-SM-5S2RB
Heart - Atrial Appendage	Heart	GTEX-13QBU-0226-SM-5LU48
Heart - Atrial Appendage	Heart	GTEX-13RTJ-0526-SM-62LDP
Heart - Atrial Appendage	Heart	GTEX-13S86-0326-SM-5SI6K
Heart - Atrial Appendage	Heart	GTEX-13SLW-0826-SM-5QGP7
Heart - Atrial Appendage	Heart	GTEX-13SLX-0926-SM-5S2NM
Heart - Atrial Appendage	Heart	GTEX-13U4I-0626-SM-5LU5L
Heart - Atrial Appendage	Heart	GTEX-13VXT-0726-SM-5SIAD
Heart - Atrial Appendage	Heart	GTEX-13W3W-0526-SM-5LU3X
Heart - Atrial Appendage	Heart	GTEX-13X6J-1226-SM-79OMF
Heart - Atrial Appendage	Heart	GTEX-13X6K-1726-SM-7IGLG
Heart - Atrial Appendage	Heart	GTEX-13YAN-1426-SM-7EPG8
Heart - Atrial Appendage	Heart	GTEX-1445S-1226-SM-5O9BF
Heart - Atrial Appendage	Heart	GTEX-144GL-1026-SM-5O99R
Heart - Atrial Appendage	Heart	GTEX-145LS-1326-SM-5Q5EP
Heart - Atrial Appendage	Heart	GTEX-145LV-0226-SM-5S2QG
Heart - Atrial Appendage	Heart	GTEX-145MN-0726-SM-5NQBH
Heart - Atrial Appendage	Heart	GTEX-146FH-1126-SM-5NQAT
Heart - Atrial Appendage	Heart	GTEX-146FR-0926-SM-5QGPE
Heart - Atrial Appendage	Heart	GTEX-1477Z-1126-SM-5P9GK
Heart - Atrial Appendage	Heart	GTEX-147F4-1126-SM-5TDDE
Heart - Atrial Appendage	Heart	GTEX-147JS-1326-SM-5SI6D
Heart - Atrial Appendage	Heart	GTEX-148VJ-1426-SM-5TDE3
Heart - Atrial Appendage	Heart	GTEX-14A5I-1026-SM-5Q5E3
Heart - Atrial Appendage	Heart	GTEX-14A6H-0326-SM-5NQAN
Heart - Atrial Appendage	Heart	GTEX-14ASI-0826-SM-5Q5EB
Heart - Atrial Appendage	Heart	GTEX-14BIL-0926-SM-7DHKN
Heart - Atrial Appendage	Heart	GTEX-14BIM-2726-SM-5Q5EG
Heart - Atrial Appendage	Heart	GTEX-14BIN-2126-SM-793AX
Heart - Atrial Appendage	Heart	GTEX-14BMV-1026-SM-5SI6B
Heart - Atrial Appendage	Heart	GTEX-14C38-1326-SM-62LDK
Heart - Atrial Appendage	Heart	GTEX-14C39-0726-SM-5RQIK
Heart - Atrial Appendage	Heart	GTEX-14C5O-1226-SM-664N3
Heart - Atrial Appendage	Heart	GTEX-14DAQ-1026-SM-793B1
Heart - Atrial Appendage	Heart	GTEX-14E1K-0826-SM-5ZZW9
Heart - Atrial Appendage	Heart	GTEX-14E6C-0326-SM-5Q5EE
Heart - Atrial Appendage	Heart	GTEX-14E7W-1126-SM-5ZZVS
Heart - Atrial Appendage	Heart	GTEX-14H4A-0426-SM-5ZZWO
Heart - Atrial Appendage	Heart	GTEX-14JG1-0826-SM-5ZZWR
Heart - Atrial Appendage	Heart	GTEX-14PHX-0626-SM-5YY8Z
Heart - Atrial Appendage	Heart	GTEX-14PHY-0326-SM-62LE3
Heart - Atrial Appendage	Heart	GTEX-14PII-1026-SM-5ZZVW
Heart - Atrial Appendage	Heart	GTEX-14PJ2-1126-SM-69LOD
Heart - Atrial Appendage	Heart	GTEX-14PJ4-0926-SM-5YY98
Heart - Atrial Appendage	Heart	GTEX-14PJ6-0626-SM-686Z6

Heart - Atrial Appendage	Heart	GTEX-14PJM-0826-SM-6LLIW
Heart - Atrial Appendage	Heart	GTEX-14PJO-0826-SM-6ETZK
Heart - Atrial Appendage	Heart	GTEX-14PK6-0726-SM-6LLIX
Heart - Atrial Appendage	Heart	GTEX-14PN3-0726-SM-6EU14
Heart - Atrial Appendage	Heart	GTEX-14PN4-0826-SM-69LON
Heart - Atrial Appendage	Heart	GTEX-14XAO-0926-SM-69LPZ
Heart - Atrial Appendage	Heart	GTEX-15CHC-0326-SM-5ZZVP
Heart - Atrial Appendage	Heart	GTEX-15CHQ-1326-SM-6871Z
Heart - Atrial Appendage	Heart	GTEX-15CHR-0326-SM-7DHN3
Heart - Atrial Appendage	Heart	GTEX-15D1Q-0426-SM-6AJ9G
Heart - Atrial Appendage	Heart	GTEX-15DCE-0826-SM-7KULW
Heart - Atrial Appendage	Heart	GTEX-15ER7-0826-SM-6PALW
Heart - Atrial Appendage	Heart	GTEX-15EU6-1326-SM-6PANG
Heart - Atrial Appendage	Heart	GTEX-15RIE-1326-SM-6PAMM
Heart - Atrial Appendage	Heart	GTEX-15RJE-0726-SM-7KUEG
Heart - Atrial Appendage	Heart	GTEX-15SDE-1826-SM-7KUFN
Heart - Atrial Appendage	Heart	GTEX-15SHU-0926-SM-6PAMK
Heart - Atrial Appendage	Heart	GTEX-15SHV-0526-SM-7KUEN
Heart - Atrial Appendage	Heart	GTEX-15SKB-0926-SM-7KUFC
Heart - Atrial Appendage	Heart	GTEX-15UF7-1526-SM-6LPI7
Heart - Atrial Appendage	Heart	GTEX-16GPK-1226-SM-7LTAU
Heart - Atrial Appendage	Heart	GTEX-16MT8-0826-SM-7KULO
Heart - Atrial Appendage	Heart	GTEX-16NPX-0826-SM-6M489
Heart - Atrial Appendage	Heart	GTEX-16XZY-0526-SM-7DHKY
Heart - Atrial Appendage	Heart	GTEX-16XZZ-1026-SM-7DHKS
Heart - Atrial Appendage	Heart	GTEX-16YQH-0726-SM-7IGM1
Heart - Atrial Appendage	Heart	GTEX-17EVP-0526-SM-7EPGY
Heart - Atrial Appendage	Heart	GTEX-17F96-0826-SM-7DHLU
Heart - Atrial Appendage	Heart	GTEX-17F9E-0726-SM-793A1
Heart - Atrial Appendage	Heart	GTEX-17F9Y-1226-SM-7EPGE
Heart - Atrial Appendage	Heart	GTEX-17GQL-0626-SM-731CN
Heart - Atrial Appendage	Heart	GTEX-17HGU-1126-SM-793BP
Heart - Atrial Appendage	Heart	GTEX-17JCI-0826-SM-7LT8W
Heart - Atrial Appendage	Heart	GTEX-17KNJ-0626-SM-7LT9L
Heart - Atrial Appendage	Heart	GTEX-17MF6-0926-SM-7LTAL
Heart - Atrial Appendage	Heart	GTEX-183WM-0926-SM-718AN
Heart - Atrial Appendage	Heart	GTEX-18465-0726-SM-7LG4L
Heart - Atrial Appendage	Heart	GTEX-18A66-1126-SM-731C5
Heart - Atrial Appendage	Heart	GTEX-18A67-1026-SM-731CE
Heart - Atrial Appendage	Heart	GTEX-18A6Q-1526-SM-731CO
Heart - Atrial Appendage	Heart	GTEX-18A7A-1226-SM-7LTAJ
Heart - Atrial Appendage	Heart	GTEX-18D9B-1126-SM-7KFSH
Heart - Atrial Appendage	Heart	GTEX-18D9U-1426-SM-718BO
Heart - Atrial Appendage	Heart	GTEX-1A32A-1326-SM-73KWW
Heart - Atrial Appendage	Heart	GTEX-1A3MX-1226-SM-731B3
Heart - Atrial Appendage	Heart	GTEX-1A8G6-1826-SM-73KY8
Heart - Atrial Appendage	Heart	GTEX-1AX8Z-1126-SM-731CS
Heart - Atrial Appendage	Heart	GTEX-1AX9I-1026-SM-73KYF
Heart - Atrial Appendage	Heart	GTEX-1AX9J-0926-SM-73KXA
Heart - Atrial Appendage	Heart	GTEX-1AX9K-0426-SM-73KUG
Heart - Atrial Appendage	Heart	GTEX-1B8KE-0326-SM-73KTY
Heart - Atrial Appendage	Heart	GTEX-1B8SF-1326-SM-7IGQJ
Heart - Atrial Appendage	Heart	GTEX-1B8SG-0726-SM-79ONS
Heart - Atrial Appendage	Heart	GTEX-1B932-1326-SM-79OM4

Heart - Atrial Appendage	Heart	GTEX-1B933-0826-SM-79OMW
Heart - Atrial Appendage	Heart	GTEX-1B97J-0326-SM-7939H
Heart - Atrial Appendage	Heart	GTEX-1B996-1026-SM-793CF
Heart - Atrial Appendage	Heart	GTEX-1BAJH-1026-SM-7EWEG
Heart - Atrial Appendage	Heart	GTEX-1C2JI-0626-SM-7DHM1
Heart - Atrial Appendage	Heart	GTEX-1C64O-0926-SM-79OLN
Heart - Atrial Appendage	Heart	GTEX-1CB4E-0626-SM-7DHMW
Heart - Atrial Appendage	Heart	GTEX-1CB4G-0726-SM-79OOJ
Heart - Atrial Appendage	Heart	GTEX-1CB4H-0626-SM-7IGN3
Heart - Atrial Appendage	Heart	GTEX-1CB4I-1526-SM-7DHMY
Heart - Atrial Appendage	Heart	GTEX-1E1VI-1426-SM-793CY
Heart - Atrial Appendage	Heart	GTEX-1EWIQ-1126-SM-7EPIK
Heart - Atrial Appendage	Heart	GTEX-U8XE-0926-SM-3DB8V
Heart - Atrial Appendage	Heart	GTEX-UPK5-0226-SM-3GAEV
Heart - Atrial Appendage	Heart	GTEX-UTHO-1226-SM-3GAEE
Heart - Atrial Appendage	Heart	GTEX-V1D1-0626-SM-4JBHN
Heart - Atrial Appendage	Heart	GTEX-V955-0826-SM-5SI82
Heart - Atrial Appendage	Heart	GTEX-VJWN-1026-SM-4M1ZN
Heart - Atrial Appendage	Heart	GTEX-VUSG-0726-SM-3GIK1
Heart - Atrial Appendage	Heart	GTEX-W5X1-0826-SM-3GILN
Heart - Atrial Appendage	Heart	GTEX-WEY5-0226-SM-3GIKN
Heart - Atrial Appendage	Heart	GTEX-WFG7-0826-SM-3GIKU
Heart - Atrial Appendage	Heart	GTEX-WFG8-0726-SM-3GILP
Heart - Atrial Appendage	Heart	GTEX-WFON-0226-SM-3GIKR
Heart - Atrial Appendage	Heart	GTEX-WH7G-0326-SM-3NMBH
Heart - Atrial Appendage	Heart	GTEX-WHPG-1026-SM-3NMBA
Heart - Atrial Appendage	Heart	GTEX-WHSE-0826-SM-4SOJU
Heart - Atrial Appendage	Heart	GTEX-WHWD-0326-SM-3LK6L
Heart - Atrial Appendage	Heart	GTEX-WI4N-0726-SM-3TW93
Heart - Atrial Appendage	Heart	GTEX-WK11-0626-SM-3NMAV
Heart - Atrial Appendage	Heart	GTEX-WL46-0726-SM-3LK5Z
Heart - Atrial Appendage	Heart	GTEX-WOFL-1226-SM-4OOST
Heart - Atrial Appendage	Heart	GTEX-WQUQ-1326-SM-3MJFF
Heart - Atrial Appendage	Heart	GTEX-WRHU-1026-SM-4E3ID
Heart - Atrial Appendage	Heart	GTEX-WWYW-1126-SM-3NB2Q
Heart - Atrial Appendage	Heart	GTEX-WY7C-1126-SM-3NB3A
Heart - Atrial Appendage	Heart	GTEX-WYBS-1226-SM-3NM9N
Heart - Atrial Appendage	Heart	GTEX-WYJK-1126-SM-3NM9Z
Heart - Atrial Appendage	Heart	GTEX-WZTO-1226-SM-3NMAZ
Heart - Atrial Appendage	Heart	GTEX-X261-0926-SM-3NMCY
Heart - Atrial Appendage	Heart	GTEX-X3Y1-0226-SM-3P5Z5
Heart - Atrial Appendage	Heart	GTEX-X4EP-0326-SM-3P5Z6
Heart - Atrial Appendage	Heart	GTEX-X4XX-1126-SM-3NMBY
Heart - Atrial Appendage	Heart	GTEX-X5EB-0626-SM-46MVQ
Heart - Atrial Appendage	Heart	GTEX-X62O-0826-SM-46MW8
Heart - Atrial Appendage	Heart	GTEX-X8HC-1026-SM-46MWH
Heart - Atrial Appendage	Heart	GTEX-XBEC-1226-SM-4AT65
Heart - Atrial Appendage	Heart	GTEX-XGQ4-0526-SM-4AT6C
Heart - Atrial Appendage	Heart	GTEX-XLM4-2026-SM-4AT4X
Heart - Atrial Appendage	Heart	GTEX-XOT4-1126-SM-4B66E
Heart - Atrial Appendage	Heart	GTEX-XPT6-0226-SM-4B65L
Heart - Atrial Appendage	Heart	GTEX-XPVG-0726-SM-4B658
Heart - Atrial Appendage	Heart	GTEX-XQ3S-0526-SM-4BOQA
Heart - Atrial Appendage	Heart	GTEX-XQ8I-0226-SM-4BOPM

Heart - Atrial Appendage	Heart	GTEX-XV7Q-0726-SM-4BRV6
Heart - Atrial Appendage	Heart	GTEX-XXEK-2026-SM-4BRVE
Heart - Atrial Appendage	Heart	GTEX-XYKS-0226-SM-4BRW3
Heart - Atrial Appendage	Heart	GTEX-Y114-0126-SM-4TT8K
Heart - Atrial Appendage	Heart	GTEX-Y3I4-0526-SM-4TT6X
Heart - Atrial Appendage	Heart	GTEX-Y3IK-1026-SM-51MR7
Heart - Atrial Appendage	Heart	GTEX-Y5V5-0726-SM-4VBPY
Heart - Atrial Appendage	Heart	GTEX-Y8E4-0426-SM-4V6GB
Heart - Atrial Appendage	Heart	GTEX-YB5K-0226-SM-5IFJE
Heart - Atrial Appendage	Heart	GTEX-YEC3-0426-SM-4YCEP
Heart - Atrial Appendage	Heart	GTEX-YEC4-0226-SM-4W1YG
Heart - Atrial Appendage	Heart	GTEX-YECK-1226-SM-4W21G
Heart - Atrial Appendage	Heart	GTEX-YF7O-0526-SM-5P9IO
Heart - Atrial Appendage	Heart	GTEX-YFC4-0426-SM-4TT3J
Heart - Atrial Appendage	Heart	GTEX-YJ8O-0826-SM-5CVM3
Heart - Atrial Appendage	Heart	GTEX-Z93S-0326-SM-5HL84
Heart - Atrial Appendage	Heart	GTEX-ZAB4-0926-SM-5CVN4
Heart - Atrial Appendage	Heart	GTEX-ZAB5-0126-SM-5CVMT
Heart - Atrial Appendage	Heart	GTEX-ZAJG-0926-SM-5Q5AB
Heart - Atrial Appendage	Heart	GTEX-ZAK1-1126-SM-5PNXU
Heart - Atrial Appendage	Heart	GTEX-ZDTS-1226-SM-4WKGL
Heart - Atrial Appendage	Heart	GTEX-ZDTT-0326-SM-4WKF9
Heart - Atrial Appendage	Heart	GTEX-ZDXO-0426-SM-4WKF6
Heart - Atrial Appendage	Heart	GTEX-ZDYS-0326-SM-5HL4W
Heart - Atrial Appendage	Heart	GTEX-ZE7O-0626-SM-57WCD
Heart - Atrial Appendage	Heart	GTEX-ZEX8-0326-SM-4WKGS
Heart - Atrial Appendage	Heart	GTEX-ZF29-0426-SM-4WKFN
Heart - Atrial Appendage	Heart	GTEX-ZF2S-0826-SM-4WWBI
Heart - Atrial Appendage	Heart	GTEX-ZF3C-1126-SM-57WEG
Heart - Atrial Appendage	Heart	GTEX-ZG7Y-1426-SM-4WWBM
Heart - Atrial Appendage	Heart	GTEX-ZGAY-0226-SM-4WWAL
Heart - Atrial Appendage	Heart	GTEX-ZLFU-0326-SM-4WWBK
Heart - Atrial Appendage	Heart	GTEX-ZPU1-0726-SM-5HL6O
Heart - Atrial Appendage	Heart	GTEX-ZT9W-0526-SM-57WFG
Heart - Atrial Appendage	Heart	GTEX-ZTPG-1326-SM-51MSQ
Heart - Atrial Appendage	Heart	GTEX-ZTTD-0626-SM-4YCCY
Heart - Atrial Appendage	Heart	GTEX-ZUA1-0726-SM-4YCD9
Heart - Atrial Appendage	Heart	GTEX-ZV7C-0526-SM-51MRB
Heart - Atrial Appendage	Heart	GTEX-ZVT2-0926-SM-5GICE
Heart - Atrial Appendage	Heart	GTEX-ZVT3-0826-SM-5GIC8
Heart - Atrial Appendage	Heart	GTEX-ZVT4-1326-SM-5NQ8E
Heart - Atrial Appendage	Heart	GTEX-ZVZQ-0726-SM-51MR3
Heart - Atrial Appendage	Heart	GTEX-ZXG5-0626-SM-5NQ85
Heart - Atrial Appendage	Heart	GTEX-ZYFC-1026-SM-5GZX9
Heart - Atrial Appendage	Heart	GTEX-ZYFD-2026-SM-5E459
Heart - Atrial Appendage	Heart	GTEX-ZYFG-0526-SM-5GZXX
Heart - Atrial Appendage	Heart	GTEX-ZYT6-0926-SM-5GIEM
Heart - Atrial Appendage	Heart	GTEX-ZYVF-1826-SM-5E44F
Heart - Atrial Appendage	Heart	GTEX-ZZPT-0926-SM-5GICZ
Heart - Atrial Appendage	Heart	GTEX-ZZPU-1126-SM-5N9CW
Heart - Left Ventricle	Heart	GTEX-111FC-0826-SM-5GZWO
Heart - Left Ventricle	Heart	GTEX-111YS-0426-SM-5987O
Heart - Left Ventricle	Heart	GTEX-1122O-0826-SM-5GICV
Heart - Left Ventricle	Heart	GTEX-117YW-0326-SM-5N9CY

Heart - Left Ventricle	Heart	GTEX-117YX-1126-SM-5H128
Heart - Left Ventricle	Heart	GTEX-11DXX-0326-SM-5PNWC
Heart - Left Ventricle	Heart	GTEX-11DXY-0826-SM-5EGGR
Heart - Left Ventricle	Heart	GTEX-11DXZ-0626-SM-5GU77
Heart - Left Ventricle	Heart	GTEX-11EM3-0626-SM-5H12Z
Heart - Left Ventricle	Heart	GTEX-11EMC-0726-SM-5EGJO
Heart - Left Ventricle	Heart	GTEX-11GS4-0526-SM-5A5KQ
Heart - Left Ventricle	Heart	GTEX-11GSP-1326-SM-5A5KY
Heart - Left Ventricle	Heart	GTEX-11I78-0826-SM-5A5K4
Heart - Left Ventricle	Heart	GTEX-11LCK-0926-SM-5A5KA
Heart - Left Ventricle	Heart	GTEX-11O72-1126-SM-5N9E2
Heart - Left Ventricle	Heart	GTEX-11ONC-0126-SM-5PNW6
Heart - Left Ventricle	Heart	GTEX-11TT1-1326-SM-5PNYM
Heart - Left Ventricle	Heart	GTEX-11TUW-1026-SM-5GU7D
Heart - Left Ventricle	Heart	GTEX-11ZUS-0226-SM-5FQT8
Heart - Left Ventricle	Heart	GTEX-11ZVC-0426-SM-5CVLD
Heart - Left Ventricle	Heart	GTEX-1211K-0626-SM-5FQUZ
Heart - Left Ventricle	Heart	GTEX-1212Z-0626-SM-5FQTB
Heart - Left Ventricle	Heart	GTEX-12584-0926-SM-5FQTN
Heart - Left Ventricle	Heart	GTEX-12696-1126-SM-5FQTI
Heart - Left Ventricle	Heart	GTEX-1269C-0826-SM-5N9EM
Heart - Left Ventricle	Heart	GTEX-12BJ1-0226-SM-5LUA2
Heart - Left Ventricle	Heart	GTEX-12WSC-1126-SM-5EQ4G
Heart - Left Ventricle	Heart	GTEX-12WSD-1126-SM-5EGJD
Heart - Left Ventricle	Heart	GTEX-12WSG-0526-SM-5FQTH
Heart - Left Ventricle	Heart	GTEX-12WSK-0626-SM-5LZUJ
Heart - Left Ventricle	Heart	GTEX-12WSN-5001-SM-7DHMK
Heart - Left Ventricle	Heart	GTEX-12ZZW-1026-SM-5GCMM
Heart - Left Ventricle	Heart	GTEX-12ZZY-1126-SM-5DUWQ
Heart - Left Ventricle	Heart	GTEX-12ZZZ-1726-SM-59HK5
Heart - Left Ventricle	Heart	GTEX-13112-0426-SM-5PNVC
Heart - Left Ventricle	Heart	GTEX-1313W-1426-SM-5KLZU
Heart - Left Ventricle	Heart	GTEX-1314G-0126-SM-5LZUL
Heart - Left Ventricle	Heart	GTEX-131XE-0626-SM-5HL98
Heart - Left Ventricle	Heart	GTEX-131XF-5001-SM-7DHLY
Heart - Left Ventricle	Heart	GTEX-131XG-0626-SM-5GCMP
Heart - Left Ventricle	Heart	GTEX-131XH-0226-SM-5LZVB
Heart - Left Ventricle	Heart	GTEX-132AR-2026-SM-5IJG4
Heart - Left Ventricle	Heart	GTEX-132NY-1726-SM-5EGKK
Heart - Left Ventricle	Heart	GTEX-132QS-0426-SM-5KLZ6
Heart - Left Ventricle	Heart	GTEX-1339X-0426-SM-5KLZY
Heart - Left Ventricle	Heart	GTEX-1399R-1926-SM-5K7X8
Heart - Left Ventricle	Heart	GTEX-1399S-1026-SM-5KLZ9
Heart - Left Ventricle	Heart	GTEX-1399T-0426-SM-5PNVJ
Heart - Left Ventricle	Heart	GTEX-1399U-0526-SM-5K7YM
Heart - Left Ventricle	Heart	GTEX-139YR-0826-SM-5LZXY
Heart - Left Ventricle	Heart	GTEX-13CF2-0426-SM-5KM1H
Heart - Left Ventricle	Heart	GTEX-13CF3-0726-SM-5J2MY
Heart - Left Ventricle	Heart	GTEX-13D11-1726-SM-5IFGQ
Heart - Left Ventricle	Heart	GTEX-13FH7-0926-SM-5J2MR
Heart - Left Ventricle	Heart	GTEX-13FHP-1026-SM-5K7Y2
Heart - Left Ventricle	Heart	GTEX-13FLV-0926-SM-5L3DZ
Heart - Left Ventricle	Heart	GTEX-13FTW-1026-SM-5L3E3
Heart - Left Ventricle	Heart	GTEX-13FTZ-0226-SM-5K7X6

Heart - Left Ventricle	Heart	GTEX-13JVG-1326-SM-5N9F8
Heart - Left Ventricle	Heart	GTEX-13N11-0426-SM-5KM3O
Heart - Left Ventricle	Heart	GTEX-13N1W-1026-SM-5IJC5
Heart - Left Ventricle	Heart	GTEX-13NYB-0226-SM-5N9G4
Heart - Left Ventricle	Heart	GTEX-13NZB-0226-SM-5KM1T
Heart - Left Ventricle	Heart	GTEX-13O1R-1226-SM-5J1NU
Heart - Left Ventricle	Heart	GTEX-13O21-2326-SM-5MR3X
Heart - Left Ventricle	Heart	GTEX-13O3O-1526-SM-5KM1C
Heart - Left Ventricle	Heart	GTEX-13O3P-1626-SM-5K7X3
Heart - Left Ventricle	Heart	GTEX-13O61-0526-SM-5J2M1
Heart - Left Ventricle	Heart	GTEX-13OVG-0526-SM-5K7YD
Heart - Left Ventricle	Heart	GTEX-13OVL-0526-SM-5KLZQ
Heart - Left Ventricle	Heart	GTEX-13OW5-1126-SM-5J1NR
Heart - Left Ventricle	Heart	GTEX-13OW6-1126-SM-5L3HL
Heart - Left Ventricle	Heart	GTEX-13OW8-2126-SM-5J1OS
Heart - Left Ventricle	Heart	GTEX-13PVQ-1226-SM-5IJDC
Heart - Left Ventricle	Heart	GTEX-13PVR-1026-SM-5QGQW
Heart - Left Ventricle	Heart	GTEX-13QBU-0426-SM-5J2O4
Heart - Left Ventricle	Heart	GTEX-13RTJ-0726-SM-5QGQN
Heart - Left Ventricle	Heart	GTEX-13S86-0226-SM-5S2PJ
Heart - Left Ventricle	Heart	GTEX-13SLX-1026-SM-5ZZUQ
Heart - Left Ventricle	Heart	GTEX-13U4I-0826-SM-5SIBD
Heart - Left Ventricle	Heart	GTEX-13VXT-1126-SM-5LU3A
Heart - Left Ventricle	Heart	GTEX-13W3W-0426-SM-5SI9D
Heart - Left Ventricle	Heart	GTEX-13X6H-0426-SM-5LU4E
Heart - Left Ventricle	Heart	GTEX-13X6I-1426-SM-5SI9Z
Heart - Left Ventricle	Heart	GTEX-13X6K-1826-SM-5O9CR
Heart - Left Ventricle	Heart	GTEX-145LV-0326-SM-5Q5BW
Heart - Left Ventricle	Heart	GTEX-145MN-0626-SM-5QGRH
Heart - Left Ventricle	Heart	GTEX-145MO-1126-SM-5NQBX
Heart - Left Ventricle	Heart	GTEX-146FH-1026-SM-5RQIF
Heart - Left Ventricle	Heart	GTEX-146FR-0626-SM-5RQJ1
Heart - Left Ventricle	Heart	GTEX-147F4-1226-SM-5NQAY
Heart - Left Ventricle	Heart	GTEX-147JS-1526-SM-5ZZWF
Heart - Left Ventricle	Heart	GTEX-148VI-0326-SM-5RQK7
Heart - Left Ventricle	Heart	GTEX-148VJ-1526-SM-5Q5DU
Heart - Left Ventricle	Heart	GTEX-1497J-1226-SM-5Q5BL
Heart - Left Ventricle	Heart	GTEX-14A5I-1226-SM-5NQBW
Heart - Left Ventricle	Heart	GTEX-14A6H-0226-SM-5Q5DX
Heart - Left Ventricle	Heart	GTEX-14BIL-1126-SM-79OMC
Heart - Left Ventricle	Heart	GTEX-14BIN-2226-SM-5YYA1
Heart - Left Ventricle	Heart	GTEX-14C38-1426-SM-5RQHZ
Heart - Left Ventricle	Heart	GTEX-14C39-0926-SM-5TDDV
Heart - Left Ventricle	Heart	GTEX-14C5O-1326-SM-5S2UW
Heart - Left Ventricle	Heart	GTEX-14DAQ-1126-SM-664MT
Heart - Left Ventricle	Heart	GTEX-14E1K-1026-SM-73KUR
Heart - Left Ventricle	Heart	GTEX-14E6C-0426-SM-5YYA8
Heart - Left Ventricle	Heart	GTEX-14E6D-0926-SM-5S2QV
Heart - Left Ventricle	Heart	GTEX-14E7W-1226-SM-5RQIU
Heart - Left Ventricle	Heart	GTEX-14H4A-0626-SM-5ZZUT
Heart - Left Ventricle	Heart	GTEX-14JG1-1026-SM-6ETZY
Heart - Left Ventricle	Heart	GTEX-14JIY-1126-SM-6EU28
Heart - Left Ventricle	Heart	GTEX-14PHX-2126-SM-6EU2U
Heart - Left Ventricle	Heart	GTEX-14PHY-0226-SM-5ZZWM

Heart - Left Ventricle	Heart	GTEX-14PJ2-1026-SM-6EU1M
Heart - Left Ventricle	Heart	GTEX-14PJ4-1126-SM-5YY91
Heart - Left Ventricle	Heart	GTEX-14PJ6-0726-SM-69LOL
Heart - Left Ventricle	Heart	GTEX-14PK6-0826-SM-5ZZVO
Heart - Left Ventricle	Heart	GTEX-14PN3-0526-SM-69LQF
Heart - Left Ventricle	Heart	GTEX-14XAO-1126-SM-6EU11
Heart - Left Ventricle	Heart	GTEX-15CHC-0426-SM-6LLHI
Heart - Left Ventricle	Heart	GTEX-15CHQ-1526-SM-69LPW
Heart - Left Ventricle	Heart	GTEX-15DCZ-1126-SM-6871O
Heart - Left Ventricle	Heart	GTEX-15DDE-1126-SM-6LLHN
Heart - Left Ventricle	Heart	GTEX-15DYW-1126-SM-6LPK5
Heart - Left Ventricle	Heart	GTEX-15EOM-5007-SM-793DG
Heart - Left Ventricle	Heart	GTEX-15ER7-1726-SM-6PAMX
Heart - Left Ventricle	Heart	GTEX-15RJ7-0226-SM-7KUMN
Heart - Left Ventricle	Heart	GTEX-15RJE-0626-SM-7KUKR
Heart - Left Ventricle	Heart	GTEX-15SHU-1126-SM-7KUMB
Heart - Left Ventricle	Heart	GTEX-15SHW-1226-SM-6M46C
Heart - Left Ventricle	Heart	GTEX-16BQI-0926-SM-6M485
Heart - Left Ventricle	Heart	GTEX-16MT8-0926-SM-6M487
Heart - Left Ventricle	Heart	GTEX-16NPX-0926-SM-7KUEZ
Heart - Left Ventricle	Heart	GTEX-16XZY-0426-SM-793BI
Heart - Left Ventricle	Heart	GTEX-16YQH-0926-SM-793BC
Heart - Left Ventricle	Heart	GTEX-17F9E-0926-SM-7IGMG
Heart - Left Ventricle	Heart	GTEX-17GQL-0526-SM-72D5N
Heart - Left Ventricle	Heart	GTEX-17HGU-1026-SM-79OKL
Heart - Left Ventricle	Heart	GTEX-17HHY-1426-SM-7EWDQ
Heart - Left Ventricle	Heart	GTEX-17JCI-1126-SM-7IGMM
Heart - Left Ventricle	Heart	GTEX-17KNJ-0826-SM-793A4
Heart - Left Ventricle	Heart	GTEX-17MF6-0726-SM-7LT8E
Heart - Left Ventricle	Heart	GTEX-18465-0926-SM-731AY
Heart - Left Ventricle	Heart	GTEX-18A66-1226-SM-7LT8G
Heart - Left Ventricle	Heart	GTEX-18A67-1226-SM-7KFRS
Heart - Left Ventricle	Heart	GTEX-18A6Q-1626-SM-7KFTA
Heart - Left Ventricle	Heart	GTEX-18D9B-1226-SM-72D6V
Heart - Left Ventricle	Heart	GTEX-18D9U-1126-SM-72D5Y
Heart - Left Ventricle	Heart	GTEX-1A3MW-1326-SM-7189P
Heart - Left Ventricle	Heart	GTEX-1A3MX-1326-SM-72D58
Heart - Left Ventricle	Heart	GTEX-1AX8Z-1326-SM-7DHMO
Heart - Left Ventricle	Heart	GTEX-1AX9I-1126-SM-7EPHH
Heart - Left Ventricle	Heart	GTEX-1AX9J-1026-SM-72D6E
Heart - Left Ventricle	Heart	GTEX-1AX9K-0526-SM-73KV1
Heart - Left Ventricle	Heart	GTEX-1AYCT-0426-SM-73KVZ
Heart - Left Ventricle	Heart	GTEX-1B8KE-0126-SM-793AM
Heart - Left Ventricle	Heart	GTEX-1B8L1-0526-SM-79ONX
Heart - Left Ventricle	Heart	GTEX-1B8SG-0826-SM-7EWF7
Heart - Left Ventricle	Heart	GTEX-1B932-1026-SM-793CL
Heart - Left Ventricle	Heart	GTEX-1B996-1126-SM-73KWN
Heart - Left Ventricle	Heart	GTEX-1BAJH-1626-SM-73KUV
Heart - Left Ventricle	Heart	GTEX-1C2JI-0526-SM-7EWEQ
Heart - Left Ventricle	Heart	GTEX-1C64O-0826-SM-79OKZ
Heart - Left Ventricle	Heart	GTEX-1C6VQ-1126-SM-7EWET
Heart - Left Ventricle	Heart	GTEX-1CAMS-1326-SM-79OJR
Heart - Left Ventricle	Heart	GTEX-1CB4G-0826-SM-7DUGF
Heart - Left Ventricle	Heart	GTEX-1E1VI-1326-SM-7IGQD

Heart - Left Ventricle	Heart	GTEX-1EH9U-0826-SM-7IGPT
Heart - Left Ventricle	Heart	GTEX-1EMGI-1226-SM-793DD
Heart - Left Ventricle	Heart	GTEX-1EU9M-1826-SM-7DUGU
Heart - Left Ventricle	Heart	GTEX-1EWIQ-1426-SM-7IGOS
Heart - Left Ventricle	Heart	GTEX-N7MS-0826-SM-2HML4
Heart - Left Ventricle	Heart	GTEX-N7MT-1326-SM-2I3FV
Heart - Left Ventricle	Heart	GTEX-NFK9-0926-SM-2HMJU
Heart - Left Ventricle	Heart	GTEX-NPJ8-0426-SM-2HMK6
Heart - Left Ventricle	Heart	GTEX-O5YT-0326-SM-32PKA
Heart - Left Ventricle	Heart	GTEX-O5YV-0326-SM-2I5H2
Heart - Left Ventricle	Heart	GTEX-O5YW-0326-SM-2I5EI
Heart - Left Ventricle	Heart	GTEX-OHPK-0326-SM-2HMJO
Heart - Left Ventricle	Heart	GTEX-OHPL-0326-SM-33HC8
Heart - Left Ventricle	Heart	GTEX-OHPM-0326-SM-33HCA
Heart - Left Ventricle	Heart	GTEX-OIZG-1126-SM-2HMIU
Heart - Left Ventricle	Heart	GTEX-OIZH-0326-SM-2HMKC
Heart - Left Ventricle	Heart	GTEX-OOBJ-0326-SM-33HBO
Heart - Left Ventricle	Heart	GTEX-OXRK-0826-SM-2HMK7
Heart - Left Ventricle	Heart	GTEX-OXRL-0326-SM-2I3F2
Heart - Left Ventricle	Heart	GTEX-OXRO-2026-SM-2YUMZ
Heart - Left Ventricle	Heart	GTEX-P44H-0726-SM-48TBT
Heart - Left Ventricle	Heart	GTEX-P4PP-0326-SM-33HC4
Heart - Left Ventricle	Heart	GTEX-P4PQ-0326-SM-2HMJ8
Heart - Left Ventricle	Heart	GTEX-P4QS-0326-SM-2I3EU
Heart - Left Ventricle	Heart	GTEX-P78B-0426-SM-2I5F5
Heart - Left Ventricle	Heart	GTEX-PLZ5-0626-SM-2I5F8
Heart - Left Ventricle	Heart	GTEX-POMQ-0326-SM-2I5FO
Heart - Left Ventricle	Heart	GTEX-PSDG-0926-SM-2I5FP
Heart - Left Ventricle	Heart	GTEX-PVOW-0426-SM-5MR5R
Heart - Left Ventricle	Heart	GTEX-PWCY-0526-SM-5P9HG
Heart - Left Ventricle	Heart	GTEX-PX3G-0326-SM-2I3EO
Heart - Left Ventricle	Heart	GTEX-Q2AH-0526-SM-2I3ED
Heart - Left Ventricle	Heart	GTEX-QDVJ-0426-SM-2I5FW
Heart - Left Ventricle	Heart	GTEX-QDVN-0326-SM-2I3FS
Heart - Left Ventricle	Heart	GTEX-QEG4-0426-SM-33HC3
Heart - Left Ventricle	Heart	GTEX-QEG5-0926-SM-2TC64
Heart - Left Ventricle	Heart	GTEX-QEL4-0926-SM-3GAD1
Heart - Left Ventricle	Heart	GTEX-QESD-0526-SM-2I5G5
Heart - Left Ventricle	Heart	GTEX-QLQ7-0526-SM-2I5G3
Heart - Left Ventricle	Heart	GTEX-QMRM-0526-SM-2I5GA
Heart - Left Ventricle	Heart	GTEX-QV44-0526-SM-2S1RE
Heart - Left Ventricle	Heart	GTEX-R45C-0926-SM-3GAD4
Heart - Left Ventricle	Heart	GTEX-R53T-0926-SM-3GADH
Heart - Left Ventricle	Heart	GTEX-R55C-0326-SM-3GAF1
Heart - Left Ventricle	Heart	GTEX-R55E-1026-SM-2TC5S
Heart - Left Ventricle	Heart	GTEX-R55G-0526-SM-2TC5O
Heart - Left Ventricle	Heart	GTEX-REY6-1026-SM-2TF4Y
Heart - Left Ventricle	Heart	GTEX-RN64-0826-SM-2TC62
Heart - Left Ventricle	Heart	GTEX-RNOR-0826-SM-2TF5C
Heart - Left Ventricle	Heart	GTEX-RTLS-0826-SM-2TF5Q
Heart - Left Ventricle	Heart	GTEX-RU72-0326-SM-2TF5T
Heart - Left Ventricle	Heart	GTEX-RUSQ-0526-SM-2TF72
Heart - Left Ventricle	Heart	GTEX-RWS6-0326-SM-2XCAP
Heart - Left Ventricle	Heart	GTEX-RWSA-0626-SM-2XCBD

Heart - Left Ventricle	Heart	GTEX-S32W-0626-SM-2XCBG
Heart - Left Ventricle	Heart	GTEX-S33H-0526-SM-5SI83
Heart - Left Ventricle	Heart	GTEX-SE5C-0626-SM-2XCDV
Heart - Left Ventricle	Heart	GTEX-SIU8-0826-SM-2XCDQ
Heart - Left Ventricle	Heart	GTEX-SNMC-0126-SM-2XCFO
Heart - Left Ventricle	Heart	GTEX-T2IS-0426-SM-32QPE
Heart - Left Ventricle	Heart	GTEX-T6MN-0926-SM-32PLX
Heart - Left Ventricle	Heart	GTEX-U3ZH-0326-SM-3DB7A
Heart - Left Ventricle	Heart	GTEX-U3ZN-1426-SM-3DB87
Heart - Left Ventricle	Heart	GTEX-U4B1-0326-SM-3DB8K
Heart - Left Ventricle	Heart	GTEX-U8XE-1126-SM-3DB8W
Heart - Left Ventricle	Heart	GTEX-UJHI-0426-SM-3DB8Y
Heart - Left Ventricle	Heart	GTEX-UJMC-0526-SM-3GAE3
Heart - Left Ventricle	Heart	GTEX-UPK5-0326-SM-5SI81
Heart - Left Ventricle	Heart	GTEX-V1D1-0526-SM-4JBGW
Heart - Left Ventricle	Heart	GTEX-V955-0726-SM-3GAFG
Heart - Left Ventricle	Heart	GTEX-WEY5-0426-SM-3GIKT
Heart - Left Ventricle	Heart	GTEX-WFG7-0726-SM-3GIKO
Heart - Left Ventricle	Heart	GTEX-WFG8-0626-SM-3GILJ
Heart - Left Ventricle	Heart	GTEX-WFON-0326-SM-3GIKX
Heart - Left Ventricle	Heart	GTEX-WH7G-0426-SM-3NMBJ
Heart - Left Ventricle	Heart	GTEX-WHPG-0826-SM-3NMBF
Heart - Left Ventricle	Heart	GTEX-WHSE-0926-SM-3NMBS
Heart - Left Ventricle	Heart	GTEX-WHWD-0426-SM-3LK83
Heart - Left Ventricle	Heart	GTEX-WI4N-0626-SM-3TW8Z
Heart - Left Ventricle	Heart	GTEX-WL46-0926-SM-3LK7T
Heart - Left Ventricle	Heart	GTEX-WQUQ-1426-SM-3MJFD
Heart - Left Ventricle	Heart	GTEX-WRHU-1226-SM-4E3IJ
Heart - Left Ventricle	Heart	GTEX-WWYW-1326-SM-3NB2S
Heart - Left Ventricle	Heart	GTEX-WY7C-0526-SM-3NB3D
Heart - Left Ventricle	Heart	GTEX-WYJK-1026-SM-3NM8W
Heart - Left Ventricle	Heart	GTEX-WZTO-1326-SM-3NM8X
Heart - Left Ventricle	Heart	GTEX-X15G-0426-SM-4PQZ6
Heart - Left Ventricle	Heart	GTEX-X261-1426-SM-4PQZ1
Heart - Left Ventricle	Heart	GTEX-X3Y1-0426-SM-3P5Z4
Heart - Left Ventricle	Heart	GTEX-X8HC-1626-SM-46MWE
Heart - Left Ventricle	Heart	GTEX-XBEC-1326-SM-4AT69
Heart - Left Ventricle	Heart	GTEX-XBED-0526-SM-47JY3
Heart - Left Ventricle	Heart	GTEX-XGQ4-0326-SM-4GIEE
Heart - Left Ventricle	Heart	GTEX-XPT6-0126-SM-4B65S
Heart - Left Ventricle	Heart	GTEX-XPVG-0826-SM-4B654
Heart - Left Ventricle	Heart	GTEX-XQ3S-0626-SM-4BOOB
Heart - Left Ventricle	Heart	GTEX-XQ8I-0126-SM-4BOPL
Heart - Left Ventricle	Heart	GTEX-XV7Q-0826-SM-4BRV7
Heart - Left Ventricle	Heart	GTEX-XXEK-0926-SM-4BRWH
Heart - Left Ventricle	Heart	GTEX-Y114-0226-SM-4TT8W
Heart - Left Ventricle	Heart	GTEX-Y3I4-0626-SM-4TT7A
Heart - Left Ventricle	Heart	GTEX-Y3IK-0826-SM-51MT8
Heart - Left Ventricle	Heart	GTEX-Y5V5-0626-SM-4VBPX
Heart - Left Ventricle	Heart	GTEX-Y5V6-0826-SM-4VBRU
Heart - Left Ventricle	Heart	GTEX-Y8E4-0326-SM-4VBRR
Heart - Left Ventricle	Heart	GTEX-YB5K-0126-SM-5IFJ2
Heart - Left Ventricle	Heart	GTEX-YEC3-0726-SM-4YCD1
Heart - Left Ventricle	Heart	GTEX-YEC4-0326-SM-4W216

Heart - Left Ventricle	Heart	GTEX-YF7O-0426-SM-5P9FS
Heart - Left Ventricle	Heart	GTEX-YFC4-0526-SM-5ZZVI
Heart - Left Ventricle	Heart	GTEX-ZAB4-1026-SM-5HL7T
Heart - Left Ventricle	Heart	GTEX-ZAB5-0226-SM-5CVMH
Heart - Left Ventricle	Heart	GTEX-ZAJG-0826-SM-5PNVA
Heart - Left Ventricle	Heart	GTEX-ZDTS-1326-SM-6LLHE
Heart - Left Ventricle	Heart	GTEX-ZDTT-0726-SM-4WKFK
Heart - Left Ventricle	Heart	GTEX-ZDXO-0726-SM-57WBT
Heart - Left Ventricle	Heart	GTEX-ZDYS-0226-SM-5HL4K
Heart - Left Ventricle	Heart	GTEX-ZEX8-0426-SM-4WWEN
Heart - Left Ventricle	Heart	GTEX-ZF29-0226-SM-4WKHO
Heart - Left Ventricle	Heart	GTEX-ZF2S-1026-SM-4WWB1
Heart - Left Ventricle	Heart	GTEX-ZF3C-1226-SM-4WWCB
Heart - Left Ventricle	Heart	GTEX-ZG7Y-0926-SM-5EQ6O
Heart - Left Ventricle	Heart	GTEX-ZGAY-0326-SM-57WF5
Heart - Left Ventricle	Heart	GTEX-ZLFU-0226-SM-4WWFH
Heart - Left Ventricle	Heart	GTEX-ZPIC-0826-SM-4WWFJ
Heart - Left Ventricle	Heart	GTEX-ZPU1-0626-SM-4YCDM
Heart - Left Ventricle	Heart	GTEX-ZQUD-1526-SM-7IGO2
Heart - Left Ventricle	Heart	GTEX-ZTPG-1226-SM-4YCDO
Heart - Left Ventricle	Heart	GTEX-ZUA1-0826-SM-4YCDL
Heart - Left Ventricle	Heart	GTEX-ZV7C-0326-SM-57WB1
Heart - Left Ventricle	Heart	GTEX-ZVT2-1026-SM-5GU55
Heart - Left Ventricle	Heart	GTEX-ZVZP-0226-SM-5NQ73
Heart - Left Ventricle	Heart	GTEX-ZYFC-1126-SM-5E44W
Heart - Left Ventricle	Heart	GTEX-ZYFG-0426-SM-5E43M
Heart - Left Ventricle	Heart	GTEX-ZYT6-1726-SM-5E44P
Heart - Left Ventricle	Heart	GTEX-ZYW4-0926-SM-59HJS
Heart - Left Ventricle	Heart	GTEX-ZZPU-0926-SM-5GZYT
Kidney - Cortex	Kidney	GTEX-11GS4-2326-SM-5A5KS
Kidney - Cortex	Kidney	GTEX-11OF3-1326-SM-5N9FJ
Kidney - Cortex	Kidney	GTEX-11TTK-1926-SM-5PNW8
Kidney - Cortex	Kidney	GTEX-12696-0926-SM-5FQTV
Kidney - Cortex	Kidney	GTEX-12WSG-0826-SM-5EQ5A
Kidney - Cortex	Kidney	GTEX-13112-2126-SM-5GCO4
Kidney - Cortex	Kidney	GTEX-1399S-0526-SM-5IJG8
Kidney - Cortex	Kidney	GTEX-13NYB-1726-SM-5N9G2
Kidney - Cortex	Kidney	GTEX-13O1R-2526-SM-5N9FW
Kidney - Cortex	Kidney	GTEX-13OVI-1126-SM-5KLZF
Kidney - Cortex	Kidney	GTEX-13OVL-1826-SM-5KLZR
Kidney - Cortex	Kidney	GTEX-13OW6-1826-SM-5N9F9
Kidney - Cortex	Kidney	GTEX-13RTJ-2226-SM-5S2Q1
Kidney - Cortex	Kidney	GTEX-145MN-0326-SM-5QGQI
Kidney - Cortex	Kidney	GTEX-147F4-2626-SM-5Q5CS
Kidney - Cortex	Kidney	GTEX-1497J-0826-SM-5NQAJ
Kidney - Cortex	Kidney	GTEX-14C39-2126-SM-664OH
Kidney - Cortex	Kidney	GTEX-14C5O-2026-SM-5YYB1
Kidney - Cortex	Kidney	GTEX-14E6D-2526-SM-5YYA9
Kidney - Cortex	Kidney	GTEX-15CHQ-2126-SM-6871M
Kidney - Cortex	Kidney	GTEX-15DYW-2026-SM-6AJBD
Kidney - Cortex	Kidney	GTEX-16MT8-1926-SM-7EPGL
Kidney - Cortex	Kidney	GTEX-17F97-1926-SM-7IGM4
Kidney - Cortex	Kidney	GTEX-17HHY-2326-SM-7KFSR
Kidney - Cortex	Kidney	GTEX-1A3MW-2226-SM-73KUX

Kidney - Cortex	Kidney	GTEX-1AX8Z-2326-SM-731CR
Kidney - Cortex	Kidney	GTEX-1BAJH-1826-SM-731DA
Kidney - Cortex	Kidney	GTEX-1C6VQ-2326-SM-7IGN1
Kidney - Cortex	Kidney	GTEX-1EKGG-2226-SM-7IGOA
Kidney - Cortex	Kidney	GTEX-N7MS-1626-SM-3LK5F
Kidney - Cortex	Kidney	GTEX-NPJ8-2226-SM-3TW8D
Kidney - Cortex	Kidney	GTEX-P4QS-1126-SM-3NMD5
Kidney - Cortex	Kidney	GTEX-QDVN-1626-SM-48TZC
Kidney - Cortex	Kidney	GTEX-QLQW-1626-SM-4R1K1
Kidney - Cortex	Kidney	GTEX-RN64-1626-SM-48FD7
Kidney - Cortex	Kidney	GTEX-T5JC-1526-SM-4DM68
Kidney - Cortex	Kidney	GTEX-XPVG-0526-SM-4B65N
Kidney - Cortex	Kidney	GTEX-Y5V6-2026-SM-5IFHO
Kidney - Cortex	Kidney	GTEX-ZC5H-1726-SM-5HL7X
Kidney - Cortex	Kidney	GTEX-ZDXO-0226-SM-4WKH7
Kidney - Cortex	Kidney	GTEX-ZE9C-1426-SM-4WKGM
Kidney - Cortex	Kidney	GTEX-ZLFU-0926-SM-5P9F8
Kidney - Cortex	Kidney	GTEX-ZVZP-0926-SM-5GIDB
Kidney - Cortex	Kidney	GTEX-ZYFG-1626-SM-5GZYY
Kidney - Cortex	Kidney	GTEX-ZYT6-2226-SM-5GIC9
Liver	Liver	GTEX-11DXY-0526-SM-5EGGQ
Liver	Liver	GTEX-11DXZ-0126-SM-5EGGY
Liver	Liver	GTEX-11EQ9-0526-SM-5A5JZ
Liver	Liver	GTEX-11GSP-0626-SM-5986T
Liver	Liver	GTEX-11NUK-1226-SM-5P9GM
Liver	Liver	GTEX-11NV4-1326-SM-5HL6V
Liver	Liver	GTEX-11OF3-0726-SM-5BC4Z
Liver	Liver	GTEX-11TT1-1726-SM-5EQLJ
Liver	Liver	GTEX-11TUW-1726-SM-5BC5C
Liver	Liver	GTEX-11WQC-0726-SM-5EQMR
Liver	Liver	GTEX-11ZTS-1426-SM-5EQMM
Liver	Liver	GTEX-11ZUS-2526-SM-59872
Liver	Liver	GTEX-11ZVC-0726-SM-5FQT9
Liver	Liver	GTEX-1212Z-0226-SM-59HLF
Liver	Liver	GTEX-12696-0826-SM-5EGGE
Liver	Liver	GTEX-1269C-0626-SM-5FQSS
Liver	Liver	GTEX-12KS4-1326-SM-5LUB3
Liver	Liver	GTEX-12WSD-1426-SM-5GCN9
Liver	Liver	GTEX-12WSG-0626-SM-5FQTQ
Liver	Liver	GTEX-12WSI-0226-SM-5GCNA
Liver	Liver	GTEX-12WSL-0226-SM-5CVMJ
Liver	Liver	GTEX-12WSM-0726-SM-5GCOW
Liver	Liver	GTEX-12ZZZ-1326-SM-59HKW
Liver	Liver	GTEX-13112-1426-SM-5EGH8
Liver	Liver	GTEX-13113-1326-SM-5GCOI
Liver	Liver	GTEX-131XE-0326-SM-5LZVO
Liver	Liver	GTEX-131XH-0626-SM-5LZWH
Liver	Liver	GTEX-131YS-1626-SM-5HL6C
Liver	Liver	GTEX-132AR-0426-SM-5IFH8
Liver	Liver	GTEX-132NY-0926-SM-5P9G3
Liver	Liver	GTEX-1399R-1226-SM-5P9GF
Liver	Liver	GTEX-1399T-0826-SM-5IFES
Liver	Liver	GTEX-139TS-1426-SM-5IFJD
Liver	Liver	GTEX-139TU-0826-SM-5IJFG

Liver	Liver	GTEX-139YR-0226-SM-5IFEM
Liver	Liver	GTEX-13FLV-0326-SM-5N9DJ
Liver	Liver	GTEX-13FTW-1126-SM-5J2NV
Liver	Liver	GTEX-13FTZ-0726-SM-5IFFY
Liver	Liver	GTEX-13N11-0926-SM-5IJG2
Liver	Liver	GTEX-13N2G-0926-SM-5IFGJ
Liver	Liver	GTEX-13NYB-1026-SM-5IFH3
Liver	Liver	GTEX-13NZ9-1326-SM-5MR3V
Liver	Liver	GTEX-13NZB-0626-SM-5IFH6
Liver	Liver	GTEX-13O1R-2026-SM-5KM3N
Liver	Liver	GTEX-13O3O-1826-SM-5IFGW
Liver	Liver	GTEX-13OVJ-1026-SM-5IFGI
Liver	Liver	GTEX-13OW6-2626-SM-5IFF2
Liver	Liver	GTEX-13PVQ-1526-SM-5IFEQ
Liver	Liver	GTEX-13PVR-0126-SM-5S2PY
Liver	Liver	GTEX-13QJC-0726-SM-5RQJK
Liver	Liver	GTEX-13SLX-1226-SM-5S2Q6
Liver	Liver	GTEX-13VXU-0926-SM-5IFFH
Liver	Liver	GTEX-13X6J-1826-SM-5TDCT
Liver	Liver	GTEX-144GL-1426-SM-79OMA
Liver	Liver	GTEX-144GM-1326-SM-5LU5E
Liver	Liver	GTEX-145LU-1326-SM-5LU9N
Liver	Liver	GTEX-145MF-0826-SM-5QGQA
Liver	Liver	GTEX-145MO-2326-SM-5NQ9K
Liver	Liver	GTEX-146FH-1526-SM-5NQBU
Liver	Liver	GTEX-14753-1626-SM-5NQ9L
Liver	Liver	GTEX-147F4-1426-SM-5LUA8
Liver	Liver	GTEX-147GR-1326-SM-7IGLB
Liver	Liver	GTEX-147JS-1126-SM-5RQIW
Liver	Liver	GTEX-148VI-0626-SM-5TDDH
Liver	Liver	GTEX-1497J-0726-SM-5Q5D1
Liver	Liver	GTEX-14A5I-1726-SM-5QGQ5
Liver	Liver	GTEX-14AS3-0126-SM-5Q5F4
Liver	Liver	GTEX-14BIL-1326-SM-79OMR
Liver	Liver	GTEX-14C38-1526-SM-5RQJ7
Liver	Liver	GTEX-14DAQ-1726-SM-5S2R2
Liver	Liver	GTEX-14E1K-0326-SM-5S2PE
Liver	Liver	GTEX-14E7W-1526-SM-6871X
Liver	Liver	GTEX-14JG1-1626-SM-664NH
Liver	Liver	GTEX-14PHX-0526-SM-664NW
Liver	Liver	GTEX-14PJO-1726-SM-68719
Liver	Liver	GTEX-14XAO-0226-SM-68728
Liver	Liver	GTEX-15DYW-1326-SM-6LPIV
Liver	Liver	GTEX-15ER7-1826-SM-6LLI7
Liver	Liver	GTEX-15RJ7-2026-SM-6LPJ4
Liver	Liver	GTEX-15RJE-1126-SM-6LPI5
Liver	Liver	GTEX-15SHU-1826-SM-7KUKS
Liver	Liver	GTEX-15SZO-1226-SM-6LPIP
Liver	Liver	GTEX-17EVP-1026-SM-7IGOZ
Liver	Liver	GTEX-17F96-1226-SM-79OK2
Liver	Liver	GTEX-17HGU-1826-SM-7IGQM
Liver	Liver	GTEX-17HHE-0126-SM-79398
Liver	Liver	GTEX-17HII-1026-SM-7IGLY
Liver	Liver	GTEX-17MF6-1126-SM-7DUFS

Liver	Liver	GTEX-18465-1626-SM-7LG6N
Liver	Liver	GTEX-18A66-2026-SM-7189C
Liver	Liver	GTEX-18A6Q-2026-SM-718AQ
Liver	Liver	GTEX-18A7A-1526-SM-72D69
Liver	Liver	GTEX-18D9B-1526-SM-7KFRK
Liver	Liver	GTEX-1A32A-0826-SM-72D5C
Liver	Liver	GTEX-1A8FM-1226-SM-7IGMD
Liver	Liver	GTEX-1A8G7-1126-SM-731ED
Liver	Liver	GTEX-1AMFI-0826-SM-731DV
Liver	Liver	GTEX-1AX8Z-1026-SM-7189R
Liver	Liver	GTEX-1AX9I-1626-SM-72D5D
Liver	Liver	GTEX-1B8SG-1326-SM-79ONT
Liver	Liver	GTEX-1B932-1426-SM-793AN
Liver	Liver	GTEX-1B933-1226-SM-731DW
Liver	Liver	GTEX-1B996-1426-SM-7EPIA
Liver	Liver	GTEX-1C6VQ-1626-SM-79OO1
Liver	Liver	GTEX-1CAMQ-1426-SM-7MKFL
Liver	Liver	GTEX-1CB4J-0726-SM-7IGN8
Liver	Liver	GTEX-1EH9U-1126-SM-7IGLU
Liver	Liver	GTEX-1EKGG-1226-SM-7IGNO
Liver	Liver	GTEX-1EWIQ-0726-SM-7MKFW
Liver	Liver	GTEX-1GF9V-1226-SM-7MKGY
Liver	Liver	GTEX-1GF9W-0826-SM-7MKHI
Liver	Liver	GTEX-1GF9X-0426-SM-7MKHN
Liver	Liver	GTEX-O5YT-0826-SM-3TW8N
Liver	Liver	GTEX-OOBJ-0826-SM-3NB2K
Liver	Liver	GTEX-P78B-1326-SM-3P611
Liver	Liver	GTEX-PX3G-0826-SM-48TZS
Liver	Liver	GTEX-Q2AG-1126-SM-48U1P
Liver	Liver	GTEX-Q734-0326-SM-48U15
Liver	Liver	GTEX-QDVN-0826-SM-48TZ2
Liver	Liver	GTEX-QEG4-1826-SM-4R1JN
Liver	Liver	GTEX-QEL4-1226-SM-447A4
Liver	Liver	GTEX-QESD-2026-SM-447BI
Liver	Liver	GTEX-QV44-0326-SM-4R1KD
Liver	Liver	GTEX-R53T-0326-SM-48FEC
Liver	Liver	GTEX-REY6-1226-SM-48FDR
Liver	Liver	GTEX-RM2N-1926-SM-48FCU
Liver	Liver	GTEX-RN64-1826-SM-48FDV
Liver	Liver	GTEX-RNOR-1426-SM-48FDJ
Liver	Liver	GTEX-RTLS-1326-SM-46MUN
Liver	Liver	GTEX-RU72-1426-SM-46MUF
Liver	Liver	GTEX-RWSA-1426-SM-47JXA
Liver	Liver	GTEX-S32W-1926-SM-4AD63
Liver	Liver	GTEX-S33H-1626-SM-4AD68
Liver	Liver	GTEX-S4Z8-0526-SM-4AD4T
Liver	Liver	GTEX-SJXC-1226-SM-4DM78
Liver	Liver	GTEX-T6MN-1226-SM-3NMA5
Liver	Liver	GTEX-TKQ2-1726-SM-4DXUP
Liver	Liver	GTEX-U3ZN-0226-SM-3DB8D
Liver	Liver	GTEX-U8XE-1526-SM-4E3HT
Liver	Liver	GTEX-UPK5-1426-SM-4JBHH
Liver	Liver	GTEX-UTHO-2426-SM-4JBHD
Liver	Liver	GTEX-VUSG-0126-SM-4KL1X

Liver	Liver	GTEX-WFON-1726-SM-4LVMQ
Liver	Liver	GTEX-WK11-1326-SM-4OOSI
Liver	Liver	GTEX-WQUQ-1926-SM-4OOSA
Liver	Liver	GTEX-WY7C-0726-SM-4ONCB
Liver	Liver	GTEX-WYVS-1926-SM-4PQZ2
Liver	Liver	GTEX-WZTO-0626-SM-4PQYY
Liver	Liver	GTEX-X261-1726-SM-4PQYT
Liver	Liver	GTEX-X3Y1-2726-SM-4PQZH
Liver	Liver	GTEX-X4EO-1126-SM-4QARQ
Liver	Liver	GTEX-X4EP-1026-SM-4QAS5
Liver	Liver	GTEX-X4XY-1626-SM-46MVN
Liver	Liver	GTEX-XBEC-1526-SM-4AT68
Liver	Liver	GTEX-XXEK-1126-SM-4BRUX
Liver	Liver	GTEX-Y5LM-0426-SM-4VBRO
Liver	Liver	GTEX-Y5V5-0926-SM-4VBPZ
Liver	Liver	GTEX-YB5E-0326-SM-5IFHU
Liver	Liver	GTEX-YEC4-0826-SM-5P9FV
Liver	Liver	GTEX-YECK-1926-SM-4W21H
Liver	Liver	GTEX-YFC4-1526-SM-5IFJS
Liver	Liver	GTEX-Z9EW-0426-SM-5CVM9
Liver	Liver	GTEX-ZAB4-0826-SM-5LU9D
Liver	Liver	GTEX-ZAB5-0426-SM-5CVMI
Liver	Liver	GTEX-ZEX8-0826-SM-4WKHK
Liver	Liver	GTEX-ZF29-2026-SM-4WWB7
Liver	Liver	GTEX-ZF2S-3026-SM-4WWCH
Liver	Liver	GTEX-ZPU1-0826-SM-57WG2
Liver	Liver	GTEX-ZTPG-1426-SM-51MT3
Liver	Liver	GTEX-ZVP2-0626-SM-51MSO
Liver	Liver	GTEX-ZVT3-1626-SM-5GU66
Liver	Liver	GTEX-ZVT4-0626-SM-5E45T
Liver	Liver	GTEX-ZYT6-0626-SM-5E45V
Liver	Liver	GTEX-ZYY3-0626-SM-5NQ6W
Liver	Liver	GTEX-ZZPU-0426-SM-5GZYH
Lung	Lung	GTEX-111CU-0326-SM-5GZXO
Lung	Lung	GTEX-111FC-1126-SM-5GZWU
Lung	Lung	GTEX-111VG-0726-SM-5GIDC
Lung	Lung	GTEX-111YS-0626-SM-5GZXV
Lung	Lung	GTEX-1122O-0126-SM-5GICA
Lung	Lung	GTEX-1128S-0726-SM-5N9D6
Lung	Lung	GTEX-117YW-0526-SM-5H11C
Lung	Lung	GTEX-117YX-1326-SM-5H125
Lung	Lung	GTEX-11DXX-0626-SM-5Q5AG
Lung	Lung	GTEX-11DXZ-0726-SM-5N9C4
Lung	Lung	GTEX-11DZ1-0426-SM-5H11A
Lung	Lung	GTEX-11EI6-0826-SM-5985V
Lung	Lung	GTEX-11EMC-0126-SM-5EGKV
Lung	Lung	GTEX-11EQ9-0226-SM-5A5JX
Lung	Lung	GTEX-11GSP-0726-SM-5986L
Lung	Lung	GTEX-11I78-0126-SM-5HL6F
Lung	Lung	GTEX-11LCK-0426-SM-5A5M8
Lung	Lung	GTEX-11NSD-0326-SM-5A5LS
Lung	Lung	GTEX-11NUK-0826-SM-5HL4U
Lung	Lung	GTEX-11NV4-1126-SM-5HL6J
Lung	Lung	GTEX-11O72-1326-SM-5BC5A

Lung	Lung	GTEX-11OF3-1126-SM-5986C
Lung	Lung	GTEX-11P7K-0326-SM-59871
Lung	Lung	GTEX-11P81-0226-SM-5HL5M
Lung	Lung	GTEX-11PRG-0926-SM-5EGI8
Lung	Lung	GTEX-11TT1-1626-SM-5EQL7
Lung	Lung	GTEX-11TUW-0526-SM-5LU9A
Lung	Lung	GTEX-11UD2-0726-SM-5EQ69
Lung	Lung	GTEX-11WQC-0626-SM-5EQMF
Lung	Lung	GTEX-11WQK-1226-SM-5GU5Z
Lung	Lung	GTEX-11ZTS-1226-SM-5EQMQ
Lung	Lung	GTEX-11ZTT-0626-SM-5EQLM
Lung	Lung	GTEX-11ZUS-0126-SM-5EQM5
Lung	Lung	GTEX-11ZVC-0226-SM-731E8
Lung	Lung	GTEX-1211K-0826-SM-5FQUP
Lung	Lung	GTEX-1212Z-1026-SM-5EGJ8
Lung	Lung	GTEX-12584-1426-SM-5EGJ9
Lung	Lung	GTEX-12696-1026-SM-5FQUV
Lung	Lung	GTEX-1269C-0926-SM-5FQSR
Lung	Lung	GTEX-12BJ1-1026-SM-5EGJA
Lung	Lung	GTEX-12KS4-0726-SM-5FQSX
Lung	Lung	GTEX-12WSA-1026-SM-5EGHN
Lung	Lung	GTEX-12WSD-0826-SM-5GCNE
Lung	Lung	GTEX-12WSE-0826-SM-5S2VL
Lung	Lung	GTEX-12WSG-5004-SM-7EPG9
Lung	Lung	GTEX-12WSH-0126-SM-5GCO3
Lung	Lung	GTEX-12WSI-0826-SM-5EGKD
Lung	Lung	GTEX-12WSJ-0226-SM-5GCP7
Lung	Lung	GTEX-12WSK-0826-SM-5CVNP
Lung	Lung	GTEX-12WSL-1026-SM-5CVNJ
Lung	Lung	GTEX-12WSN-5004-SM-793CE
Lung	Lung	GTEX-12ZZW-0926-SM-5LZUD
Lung	Lung	GTEX-12ZZY-0926-SM-5EQ6I
Lung	Lung	GTEX-13111-0426-SM-5DUXR
Lung	Lung	GTEX-13113-5004-SM-79ONL
Lung	Lung	GTEX-1313W-0926-SM-5EQ56
Lung	Lung	GTEX-131XE-0726-SM-5HL9K
Lung	Lung	GTEX-131XF-1026-SM-5BC6A
Lung	Lung	GTEX-131XH-0426-SM-5DUWU
Lung	Lung	GTEX-131XW-1126-SM-5EGK4
Lung	Lung	GTEX-131YS-0926-SM-5IJB9
Lung	Lung	GTEX-132NY-1226-SM-5PNVF
Lung	Lung	GTEX-132QS-0726-SM-5IJE9
Lung	Lung	GTEX-1339X-0626-SM-5IJER
Lung	Lung	GTEX-133LE-0526-SM-5N9EJ
Lung	Lung	GTEX-1399S-1726-SM-5L3DI
Lung	Lung	GTEX-1399U-0826-SM-5KM1P
Lung	Lung	GTEX-139T6-0426-SM-5IJEM
Lung	Lung	GTEX-139TT-0726-SM-5K7XW
Lung	Lung	GTEX-139UW-0226-SM-5K7WU
Lung	Lung	GTEX-139YR-0926-SM-5LZYB
Lung	Lung	GTEX-13CF3-0426-SM-5IJEU
Lung	Lung	GTEX-13D11-0326-SM-5LZXX
Lung	Lung	GTEX-13FH7-1726-SM-5IJE7
Lung	Lung	GTEX-13FHO-1026-SM-5KM1Q

Lung	Lung	GTEX-13FHP-0726-SM-5K7YI
Lung	Lung	GTEX-13FLV-0426-SM-5KLZA
Lung	Lung	GTEX-13FTX-0326-SM-5J2NG
Lung	Lung	GTEX-13FTY-0126-SM-5J2NZ
Lung	Lung	GTEX-13FTZ-0526-SM-5IJCW
Lung	Lung	GTEX-13G51-0426-SM-5K7Z5
Lung	Lung	GTEX-13JUV-0526-SM-5K7XE
Lung	Lung	GTEX-13JVG-1426-SM-5MR4W
Lung	Lung	GTEX-13N11-0326-SM-5LUA3
Lung	Lung	GTEX-13N1W-0726-SM-5MR57
Lung	Lung	GTEX-13N2G-0826-SM-5IJE6
Lung	Lung	GTEX-13NYB-0626-SM-5MR47
Lung	Lung	GTEX-13NYS-1626-SM-5J2MU
Lung	Lung	GTEX-13NZ8-0326-SM-5L3DF
Lung	Lung	GTEX-13NZ9-0926-SM-5KM12
Lung	Lung	GTEX-13NZA-1426-SM-5KM4Y
Lung	Lung	GTEX-13O21-3026-SM-5J2NI
Lung	Lung	GTEX-13O3O-0726-SM-5J1N7
Lung	Lung	GTEX-13O3P-1026-SM-5N9E7
Lung	Lung	GTEX-13O3Q-0526-SM-5KM18
Lung	Lung	GTEX-13O61-0726-SM-5J2MD
Lung	Lung	GTEX-13OVG-0326-SM-5KM57
Lung	Lung	GTEX-13OVH-1026-SM-5J2NP
Lung	Lung	GTEX-13OVJ-0726-SM-5KM1W
Lung	Lung	GTEX-13OVL-0626-SM-5KM13
Lung	Lung	GTEX-13OW5-0726-SM-5KLZK
Lung	Lung	GTEX-13OW6-0826-SM-5L3GA
Lung	Lung	GTEX-13OW7-0926-SM-5L3EX
Lung	Lung	GTEX-13OW8-1726-SM-5L3GO
Lung	Lung	GTEX-13PL7-1726-SM-5J2NX
Lung	Lung	GTEX-13PVQ-0926-SM-5IJFD
Lung	Lung	GTEX-13QBU-0726-SM-5J2OA
Lung	Lung	GTEX-13QJ3-1026-SM-5QGQU
Lung	Lung	GTEX-13QJC-0526-SM-5RQKB
Lung	Lung	GTEX-13RTJ-1126-SM-5S2UJ
Lung	Lung	GTEX-13S7M-2126-SM-5S2QR
Lung	Lung	GTEX-13S86-0626-SM-5Q5E7
Lung	Lung	GTEX-13SLW-1226-SM-5S2Q7
Lung	Lung	GTEX-13U4I-1426-SM-5J2M3
Lung	Lung	GTEX-13VXT-1426-SM-5LU4B
Lung	Lung	GTEX-13VXU-2726-SM-5LU4N
Lung	Lung	GTEX-13W3W-0326-SM-731DS
Lung	Lung	GTEX-13X6K-1626-SM-7EWCX
Lung	Lung	GTEX-13YAN-1026-SM-5O9CF
Lung	Lung	GTEX-144GM-0126-SM-5Q5AX
Lung	Lung	GTEX-144GN-0426-SM-5O9AP
Lung	Lung	GTEX-144GO-0226-SM-5LUB1
Lung	Lung	GTEX-145LS-1226-SM-5Q5D9
Lung	Lung	GTEX-145LT-0326-SM-5LUAD
Lung	Lung	GTEX-145LU-0526-SM-5O9AT
Lung	Lung	GTEX-145ME-0226-SM-5S2QN
Lung	Lung	GTEX-145MF-0726-SM-5Q5BT
Lung	Lung	GTEX-145MH-0626-SM-5NQAK
Lung	Lung	GTEX-145MN-0926-SM-5NQBT

Lung	Lung	GTEX-145MO-1326-SM-5Q5EF
Lung	Lung	GTEX-146FH-1226-SM-5NQB6
Lung	Lung	GTEX-146FQ-0926-SM-5LUAV
Lung	Lung	GTEX-1477Z-0626-SM-5NQB7
Lung	Lung	GTEX-147F3-0726-SM-5NQ9U
Lung	Lung	GTEX-147F4-0926-SM-5Q5EO
Lung	Lung	GTEX-147GR-1226-SM-5TDCL
Lung	Lung	GTEX-147JS-1226-SM-5RQK4
Lung	Lung	GTEX-148VI-0226-SM-5RQKA
Lung	Lung	GTEX-148VJ-0826-SM-5LU8V
Lung	Lung	GTEX-1497J-0326-SM-5Q5CN
Lung	Lung	GTEX-14A6H-0526-SM-5NQAZ
Lung	Lung	GTEX-14ABY-1126-SM-5Q5F8
Lung	Lung	GTEX-14AS3-0926-SM-5TDD6
Lung	Lung	GTEX-14BIL-1226-SM-79OME
Lung	Lung	GTEX-14BMU-0526-SM-73KW4
Lung	Lung	GTEX-14BMV-0826-SM-73KXU
Lung	Lung	GTEX-14C39-0326-SM-5TDDX
Lung	Lung	GTEX-14C5O-1126-SM-5TDEH
Lung	Lung	GTEX-14DAQ-0926-SM-793AZ
Lung	Lung	GTEX-14DAR-0226-SM-5S2PR
Lung	Lung	GTEX-14E1K-0226-SM-62LDT
Lung	Lung	GTEX-14E6C-1426-SM-5ZZWH
Lung	Lung	GTEX-14E6D-1026-SM-5S2RS
Lung	Lung	GTEX-14E6E-0426-SM-73KUE
Lung	Lung	GTEX-14E7W-1326-SM-5RQIV
Lung	Lung	GTEX-14JG1-0926-SM-5YY8W
Lung	Lung	GTEX-14JG6-0326-SM-6AJBT
Lung	Lung	GTEX-14JIY-1326-SM-6AJB3
Lung	Lung	GTEX-14LLW-0726-SM-5ZZVV
Lung	Lung	GTEX-14LZ3-0726-SM-5YYAB
Lung	Lung	GTEX-14PHY-0526-SM-664NM
Lung	Lung	GTEX-14PJ4-0626-SM-6AJBS
Lung	Lung	GTEX-14PJ5-0226-SM-5YY99
Lung	Lung	GTEX-14PJM-0926-SM-6AJ9Y
Lung	Lung	GTEX-14PJO-0926-SM-686YT
Lung	Lung	GTEX-14PK6-0326-SM-6AJ9S
Lung	Lung	GTEX-14PQA-1126-SM-7KUM4
Lung	Lung	GTEX-14XAO-0526-SM-6AJB7
Lung	Lung	GTEX-15CHC-0226-SM-5YYBB
Lung	Lung	GTEX-15CHR-0726-SM-7EPHG
Lung	Lung	GTEX-15D1Q-0526-SM-6AJAY
Lung	Lung	GTEX-15DCZ-0826-SM-6AJBB
Lung	Lung	GTEX-15ER7-0926-SM-7KUMG
Lung	Lung	GTEX-15EU6-1226-SM-6AJBE
Lung	Lung	GTEX-15FZZ-0326-SM-6M48O
Lung	Lung	GTEX-15G1A-0426-SM-6M468
Lung	Lung	GTEX-15RIE-0326-SM-6PAMC
Lung	Lung	GTEX-15RJ7-0626-SM-6M47V
Lung	Lung	GTEX-15RJE-1026-SM-6M46X
Lung	Lung	GTEX-15SB6-0426-SM-6LPJ5
Lung	Lung	GTEX-15SHV-0326-SM-6M475
Lung	Lung	GTEX-15SHW-0926-SM-6LPIO
Lung	Lung	GTEX-15SKB-0826-SM-7KUFB

Lung	Lung	GTEX-15UKP-1926-SM-6LPI9
Lung	Lung	GTEX-169BO-0226-SM-79OL1
Lung	Lung	GTEX-16AAH-0426-SM-7DUFM
Lung	Lung	GTEX-16MT8-1026-SM-6LPK1
Lung	Lung	GTEX-16MTA-1226-SM-7KULL
Lung	Lung	GTEX-16NGA-0226-SM-718AI
Lung	Lung	GTEX-16XZZ-0926-SM-7DHLI
Lung	Lung	GTEX-16Z82-1026-SM-6M48A
Lung	Lung	GTEX-178AV-0326-SM-6LPJF
Lung	Lung	GTEX-17EVP-0726-SM-7EWDX
Lung	Lung	GTEX-17EVQ-1526-SM-79ONG
Lung	Lung	GTEX-17F96-0626-SM-793CC
Lung	Lung	GTEX-17F98-0226-SM-793BV
Lung	Lung	GTEX-17F9Y-1026-SM-7IGO4
Lung	Lung	GTEX-17GQL-0726-SM-731BL
Lung	Lung	GTEX-17HG3-0326-SM-7IGP4
Lung	Lung	GTEX-17HGU-0926-SM-79OKO
Lung	Lung	GTEX-17HHE-0626-SM-7DHL6
Lung	Lung	GTEX-17HII-0926-SM-79ON7
Lung	Lung	GTEX-17JCI-1526-SM-7IGOQ
Lung	Lung	GTEX-17KNJ-0926-SM-7IGP2
Lung	Lung	GTEX-17MF6-0826-SM-7LT8F
Lung	Lung	GTEX-183FY-0726-SM-793C3
Lung	Lung	GTEX-18465-0626-SM-7LT8X
Lung	Lung	GTEX-18A66-0926-SM-718BG
Lung	Lung	GTEX-18A67-1126-SM-7KFSB
Lung	Lung	GTEX-18A6Q-0826-SM-7KFRD
Lung	Lung	GTEX-18A7A-1026-SM-7LT8B
Lung	Lung	GTEX-18D9A-0226-SM-7KFSJ
Lung	Lung	GTEX-18D9B-1026-SM-7KFS8
Lung	Lung	GTEX-18D9U-0526-SM-72D7C
Lung	Lung	GTEX-18QFQ-0926-SM-7LG4V
Lung	Lung	GTEX-1A32A-0726-SM-731D4
Lung	Lung	GTEX-1A3MV-0526-SM-72D5A
Lung	Lung	GTEX-1A8FM-0826-SM-793D5
Lung	Lung	GTEX-1A8G6-0726-SM-73KV5
Lung	Lung	GTEX-1AX8Z-1526-SM-73KW1
Lung	Lung	GTEX-1AX9I-0826-SM-73KUT
Lung	Lung	GTEX-1AX9J-1626-SM-73KUI
Lung	Lung	GTEX-1AX9K-0826-SM-731F9
Lung	Lung	GTEX-1AYCT-0726-SM-7IGNG
Lung	Lung	GTEX-1AYD5-1226-SM-7EWEP
Lung	Lung	GTEX-1B8KZ-0526-SM-73KW2
Lung	Lung	GTEX-1B8L1-0626-SM-7EPHL
Lung	Lung	GTEX-1B8SF-0826-SM-73KW8
Lung	Lung	GTEX-1B932-0726-SM-731EY
Lung	Lung	GTEX-1B97I-0226-SM-73KVO
Lung	Lung	GTEX-1B996-0726-SM-7IGPI
Lung	Lung	GTEX-1C4CL-0826-SM-7EWEZ
Lung	Lung	GTEX-1C64O-1226-SM-79OOC
Lung	Lung	GTEX-1C6VR-0626-SM-79OOY
Lung	Lung	GTEX-1C6VS-1226-SM-79OO2
Lung	Lung	GTEX-1C6WA-0726-SM-7IGQ5
Lung	Lung	GTEX-1CAMQ-1026-SM-7EPIC

Lung	Lung	GTEX-1CB4F-0926-SM-7DHMJ
Lung	Lung	GTEX-1CB4I-2126-SM-793AG
Lung	Lung	GTEX-1CB4J-1726-SM-79OOL
Lung	Lung	GTEX-1EMGI-1426-SM-7IGNQ
Lung	Lung	GTEX-1EN7A-0826-SM-7MKFV
Lung	Lung	GTEX-N7MS-0926-SM-2HMIZ
Lung	Lung	GTEX-N7MT-0126-SM-2D7VT
Lung	Lung	GTEX-NFK9-1026-SM-2HMK1
Lung	Lung	GTEX-NL3G-0526-SM-4RTWW
Lung	Lung	GTEX-NPJ8-0326-SM-2D7VV
Lung	Lung	GTEX-O5YT-0526-SM-32PK8
Lung	Lung	GTEX-O5YV-0526-SM-2I5GE
Lung	Lung	GTEX-O5YW-0526-SM-2YUMX
Lung	Lung	GTEX-OHPL-0526-SM-3NM8U
Lung	Lung	GTEX-OHPM-0526-SM-2YUMJ
Lung	Lung	GTEX-OIZG-0526-SM-2HMLF
Lung	Lung	GTEX-OIZH-0526-SM-2HMKV
Lung	Lung	GTEX-OIZI-1026-SM-3NB1K
Lung	Lung	GTEX-OOBJ-0526-SM-48TDK
Lung	Lung	GTEX-OOBK-0526-SM-2HMJJ
Lung	Lung	GTEX-OXRK-0926-SM-2HMKP
Lung	Lung	GTEX-OXRL-0526-SM-2I3EZ
Lung	Lung	GTEX-OXRN-0526-SM-2I5EN
Lung	Lung	GTEX-OXRO-0326-SM-33HBM
Lung	Lung	GTEX-OXRP-0526-SM-2I3EW
Lung	Lung	GTEX-P44H-1126-SM-48TBU
Lung	Lung	GTEX-P4PP-0526-SM-2HMKE
Lung	Lung	GTEX-P4PQ-0526-SM-2HMKR
Lung	Lung	GTEX-P4QS-0526-SM-2I3ET
Lung	Lung	GTEX-P4QT-0526-SM-2I3EX
Lung	Lung	GTEX-P78B-0926-SM-2I5FA
Lung	Lung	GTEX-PLZ4-0726-SM-2TC6Q
Lung	Lung	GTEX-PLZ5-0726-SM-2I5F9
Lung	Lung	GTEX-PLZ6-0426-SM-5IJDW
Lung	Lung	GTEX-POMQ-0526-SM-3GADD
Lung	Lung	GTEX-POYW-1226-SM-2XCEP
Lung	Lung	GTEX-PSDG-1126-SM-2S1ON
Lung	Lung	GTEX-PVOW-1026-SM-2XCF9
Lung	Lung	GTEX-PW2O-0526-SM-2I3DX
Lung	Lung	GTEX-PX3G-0526-SM-2I3EM
Lung	Lung	GTEX-Q2AG-1026-SM-33HBW
Lung	Lung	GTEX-Q2AH-0426-SM-2I3EP
Lung	Lung	GTEX-Q734-0626-SM-2I3EF
Lung	Lung	GTEX-QCQG-0326-SM-2I3ES
Lung	Lung	GTEX-QDT8-0926-SM-32PL2
Lung	Lung	GTEX-QDVJ-0926-SM-2I5FU
Lung	Lung	GTEX-QDVN-0726-SM-4B64L
Lung	Lung	GTEX-QEG4-0526-SM-48TZD
Lung	Lung	GTEX-QEG5-1126-SM-33HC2
Lung	Lung	GTEX-QEL4-0826-SM-3GAF2
Lung	Lung	GTEX-QESD-0626-SM-2I5G4
Lung	Lung	GTEX-QMR6-1926-SM-32PL9
Lung	Lung	GTEX-QMRM-0826-SM-3NB33
Lung	Lung	GTEX-QV44-0926-SM-2S1RH

Lung	Lung	GTEX-QXCU-0626-SM-2TC69
Lung	Lung	GTEX-R3RS-1026-SM-3GADF
Lung	Lung	GTEX-R55C-0526-SM-3GIKA
Lung	Lung	GTEX-R55D-0926-SM-3GAEU
Lung	Lung	GTEX-R55G-0826-SM-2TC5U
Lung	Lung	GTEX-REY6-0426-SM-2TF5G
Lung	Lung	GTEX-RM2N-0426-SM-2TF4T
Lung	Lung	GTEX-RN64-1226-SM-2TC6E
Lung	Lung	GTEX-RNOR-0726-SM-2TF5I
Lung	Lung	GTEX-RTLS-0926-SM-2TF5X
Lung	Lung	GTEX-RU1J-0126-SM-2TF6Y
Lung	Lung	GTEX-RU72-0526-SM-2TF5Z
Lung	Lung	GTEX-RUSQ-0626-SM-2TF5V
Lung	Lung	GTEX-RVPV-1726-SM-5SI8C
Lung	Lung	GTEX-RWS6-0226-SM-2XCA9
Lung	Lung	GTEX-RWSA-1126-SM-2XCAZ
Lung	Lung	GTEX-S32W-0326-SM-2XCBI
Lung	Lung	GTEX-S33H-0626-SM-2XCBJ
Lung	Lung	GTEX-S341-0326-SM-2XCAU
Lung	Lung	GTEX-S7SE-0926-SM-2XCD6
Lung	Lung	GTEX-SE5C-0526-SM-2XCE1
Lung	Lung	GTEX-SIU8-0926-SM-5SI87
Lung	Lung	GTEX-SN8G-0926-SM-4DM5I
Lung	Lung	GTEX-SNOS-0426-SM-32PMH
Lung	Lung	GTEX-T2IS-0526-SM-32QP9
Lung	Lung	GTEX-T5JC-0826-SM-32PMC
Lung	Lung	GTEX-T6MN-0826-SM-32PM4
Lung	Lung	GTEX-T6MO-0426-SM-32QOI
Lung	Lung	GTEX-T8EM-0326-SM-3DB7F
Lung	Lung	GTEX-TML8-0326-SM-4GICN
Lung	Lung	GTEX-TMMY-0926-SM-4TT1Z
Lung	Lung	GTEX-TSE9-0726-SM-3DB8C
Lung	Lung	GTEX-U3ZH-0526-SM-3DB75
Lung	Lung	GTEX-U3ZM-0426-SM-3DB73
Lung	Lung	GTEX-U3ZN-0626-SM-3DB7U
Lung	Lung	GTEX-U412-0826-SM-3DB9K
Lung	Lung	GTEX-U8T8-2226-SM-3DB95
Lung	Lung	GTEX-U8XE-1426-SM-3DB8Q
Lung	Lung	GTEX-UJHI-0726-SM-3DB92
Lung	Lung	GTEX-UJMC-0726-SM-3GADX
Lung	Lung	GTEX-UPJH-0826-SM-4WKFD
Lung	Lung	GTEX-UPK5-1126-SM-3GAEJ
Lung	Lung	GTEX-V1D1-0826-SM-3P5ZA
Lung	Lung	GTEX-VJYA-0326-SM-3GAEX
Lung	Lung	GTEX-VUSG-0926-SM-3GIK6
Lung	Lung	GTEX-W5X1-0526-SM-3GILH
Lung	Lung	GTEX-WFG7-0526-SM-3GIKI
Lung	Lung	GTEX-WFG8-0926-SM-3GIKJ
Lung	Lung	GTEX-WFJO-0326-SM-3GIL3
Lung	Lung	GTEX-WFON-0426-SM-3GIL4
Lung	Lung	GTEX-WH7G-0726-SM-3NMBM
Lung	Lung	GTEX-WHPG-1426-SM-3NMBB
Lung	Lung	GTEX-WHSB-0326-SM-5FQSD
Lung	Lung	GTEX-WK11-0526-SM-3NB3O

Lung	Lung	GTEX-WOFM-0126-SM-3MJFE
Lung	Lung	GTEX-WRHU-0226-SM-3MJFV
Lung	Lung	GTEX-WVJS-0826-SM-4MVNR
Lung	Lung	GTEX-WWYW-0926-SM-3NB2Z
Lung	Lung	GTEX-WY7C-0426-SM-3NB3C
Lung	Lung	GTEX-WYBS-1126-SM-3NMAM
Lung	Lung	GTEX-WYJK-0826-SM-3NM8Y
Lung	Lung	GTEX-WYVS-0526-SM-3NM9W
Lung	Lung	GTEX-WZTO-0426-SM-3NM99
Lung	Lung	GTEX-X261-1026-SM-3NMDL
Lung	Lung	GTEX-X3Y1-0626-SM-3P5YS
Lung	Lung	GTEX-X4EO-0926-SM-3P5Z2
Lung	Lung	GTEX-X4EP-0526-SM-3P5YW
Lung	Lung	GTEX-X4LF-0526-SM-3NMB6
Lung	Lung	GTEX-X4XX-1026-SM-4QARO
Lung	Lung	GTEX-X4XY-1026-SM-46MVX
Lung	Lung	GTEX-X585-1026-SM-46MW6
Lung	Lung	GTEX-X5EB-0426-SM-46MVY
Lung	Lung	GTEX-XBEC-1026-SM-4QASM
Lung	Lung	GTEX-XBED-0826-SM-47JYC
Lung	Lung	GTEX-XBEW-0226-SM-4AT6A
Lung	Lung	GTEX-XGQ4-0826-SM-4AT4T
Lung	Lung	GTEX-XMD2-1026-SM-4WWE8
Lung	Lung	GTEX-XOT4-1426-SM-4B65T
Lung	Lung	GTEX-XPVG-1026-SM-4B64Y
Lung	Lung	GTEX-XQ3S-0926-SM-4BOPI
Lung	Lung	GTEX-XQ8I-1126-SM-4BOO2
Lung	Lung	GTEX-XV7Q-0426-SM-4BRVN
Lung	Lung	GTEX-XXEK-0626-SM-4BRWE
Lung	Lung	GTEX-XYKS-0526-SM-4BRW2
Lung	Lung	GTEX-Y111-1026-SM-4TT22
Lung	Lung	GTEX-Y3I4-0426-SM-4TT29
Lung	Lung	GTEX-Y3IK-0626-SM-4WWE4
Lung	Lung	GTEX-Y5LM-0726-SM-4VBRP
Lung	Lung	GTEX-Y5V5-0826-SM-4VBQD
Lung	Lung	GTEX-Y5V6-0226-SM-4V6G7
Lung	Lung	GTEX-Y8E4-0526-SM-4V6GC
Lung	Lung	GTEX-Y8LW-0326-SM-4VBQ9
Lung	Lung	GTEX-Y9LG-0526-SM-4VBRY
Lung	Lung	GTEX-YB5E-0726-SM-4VDSH
Lung	Lung	GTEX-YEC3-0226-SM-5IFJO
Lung	Lung	GTEX-YEC4-0526-SM-4W21U
Lung	Lung	GTEX-YECK-0926-SM-4W214
Lung	Lung	GTEX-YF7O-0626-SM-4W21R
Lung	Lung	GTEX-YFC4-1126-SM-5RQJN
Lung	Lung	GTEX-YFCO-0426-SM-4W1Z7
Lung	Lung	GTEX-YJ8O-1826-SM-5HL82
Lung	Lung	GTEX-ZA64-0326-SM-5HL8T
Lung	Lung	GTEX-ZAB4-0626-SM-5CVN3
Lung	Lung	GTEX-ZAB5-0626-SM-5PNVB
Lung	Lung	GTEX-ZC5H-0926-SM-5CVMZ
Lung	Lung	GTEX-ZDTS-1026-SM-4WAXS
Lung	Lung	GTEX-ZDTT-0926-SM-5J2MS
Lung	Lung	GTEX-ZDXO-1326-SM-57WBS

Lung	Lung	GTEX-ZDYS-0426-SM-5IJEQ
Lung	Lung	GTEX-ZE7O-0826-SM-57WCP
Lung	Lung	GTEX-ZEX8-0526-SM-4WKH5
Lung	Lung	GTEX-ZF28-0726-SM-4WKFU
Lung	Lung	GTEX-ZF29-1026-SM-4WKGC
Lung	Lung	GTEX-ZF2S-0626-SM-4WKH2
Lung	Lung	GTEX-ZG7Y-1026-SM-4WWDG
Lung	Lung	GTEX-ZLV1-0426-SM-4WWC2
Lung	Lung	GTEX-ZLWG-0626-SM-4WWFR
Lung	Lung	GTEX-ZPIC-0626-SM-57WDY
Lung	Lung	GTEX-ZPU1-0926-SM-57WDO
Lung	Lung	GTEX-ZQG8-0326-SM-51MSE
Lung	Lung	GTEX-ZT9W-0726-SM-4YCDC
Lung	Lung	GTEX-ZT9X-0326-SM-51MTE
Lung	Lung	GTEX-ZTPG-0926-SM-5O99H
Lung	Lung	GTEX-ZTTD-1126-SM-51MRP
Lung	Lung	GTEX-ZUA1-1026-SM-4YCEA
Lung	Lung	GTEX-ZVT3-0926-SM-5GICK
Lung	Lung	GTEX-ZVZQ-1526-SM-5N9G6
Lung	Lung	GTEX-ZXG5-0826-SM-5GID6
Lung	Lung	GTEX-ZY6K-0326-SM-5SIBB
Lung	Lung	GTEX-ZYFG-0226-SM-5GIDT
Lung	Lung	GTEX-ZYT6-0526-SM-5GIEA
Lung	Lung	GTEX-ZYVF-1726-SM-5E443
Lung	Lung	GTEX-ZYW4-1526-SM-5SIBA
Lung	Lung	GTEX-ZYY3-0926-SM-5E454
Lung	Lung	GTEX-ZZPT-1326-SM-5E43H
Lung	Lung	GTEX-ZZPU-0526-SM-5E44U
Minor Salivary Gland	Salivary Gland	GTEX-1117F-3026-SM-5GZYU
Minor Salivary Gland	Salivary Gland	GTEX-111FC-2926-SM-5GZY7
Minor Salivary Gland	Salivary Gland	GTEX-1192X-2926-SM-5N9BA
Minor Salivary Gland	Salivary Gland	GTEX-11EMC-2526-SM-5PNVU
Minor Salivary Gland	Salivary Gland	GTEX-11EQ9-2026-SM-5P9JI
Minor Salivary Gland	Salivary Gland	GTEX-11NV4-1926-SM-5N9D4
Minor Salivary Gland	Salivary Gland	GTEX-11P81-2026-SM-5P9GT
Minor Salivary Gland	Salivary Gland	GTEX-11P82-0926-SM-5986X
Minor Salivary Gland	Salivary Gland	GTEX-11TUW-2426-SM-5EQKB
Minor Salivary Gland	Salivary Gland	GTEX-11WQK-2926-SM-5EQKT
Minor Salivary Gland	Salivary Gland	GTEX-11ZTS-3026-SM-5EQM7
Minor Salivary Gland	Salivary Gland	GTEX-11ZTT-2226-SM-5CVLP
Minor Salivary Gland	Salivary Gland	GTEX-12BJ1-2226-SM-5BC6G
Minor Salivary Gland	Salivary Gland	GTEX-12WSL-2126-SM-5GCNG
Minor Salivary Gland	Salivary Gland	GTEX-12WSM-1826-SM-5BC64
Minor Salivary Gland	Salivary Gland	GTEX-12WSN-2226-SM-5DUXT
Minor Salivary Gland	Salivary Gland	GTEX-13111-2326-SM-5LZUQ
Minor Salivary Gland	Salivary Gland	GTEX-13113-1526-SM-5EGHV
Minor Salivary Gland	Salivary Gland	GTEX-1314G-2026-SM-5LZUM
Minor Salivary Gland	Salivary Gland	GTEX-131XF-2826-SM-5EGKH
Minor Salivary Gland	Salivary Gland	GTEX-131XH-2626-SM-5GCNP
Minor Salivary Gland	Salivary Gland	GTEX-1339X-2326-SM-5HL5B
Minor Salivary Gland	Salivary Gland	GTEX-13CF2-2426-SM-5LZYL
Minor Salivary Gland	Salivary Gland	GTEX-13G51-2426-SM-5LZY8
Minor Salivary Gland	Salivary Gland	GTEX-13JVG-2926-SM-5J2N9
Minor Salivary Gland	Salivary Gland	GTEX-13NYB-2426-SM-5IFF4

Minor Salivary Gland	Salivary Gland	GTEX-13O21-1926-SM-5MR39
Minor Salivary Gland	Salivary Gland	GTEX-13OVI-2326-SM-5KM47
Minor Salivary Gland	Salivary Gland	GTEX-13OVK-1826-SM-6PALE
Minor Salivary Gland	Salivary Gland	GTEX-13OVL-2926-SM-5IFG3
Minor Salivary Gland	Salivary Gland	GTEX-13QBU-2126-SM-5KLZN
Minor Salivary Gland	Salivary Gland	GTEX-13QJC-2326-SM-5Q5CD
Minor Salivary Gland	Salivary Gland	GTEX-13VXT-1726-SM-5IFF5
Minor Salivary Gland	Salivary Gland	GTEX-1445S-2926-SM-5O993
Minor Salivary Gland	Salivary Gland	GTEX-145ME-1626-SM-5RQI3
Minor Salivary Gland	Salivary Gland	GTEX-146FR-1226-SM-5SIA7
Minor Salivary Gland	Salivary Gland	GTEX-14B4R-1426-SM-5Q5CG
Minor Salivary Gland	Salivary Gland	GTEX-14E1K-2626-SM-664OJ
Minor Salivary Gland	Salivary Gland	GTEX-14JIY-3026-SM-6EU1R
Minor Salivary Gland	Salivary Gland	GTEX-14PJ3-1526-SM-664O9
Minor Salivary Gland	Salivary Gland	GTEX-14PJN-1726-SM-69LO7
Minor Salivary Gland	Salivary Gland	GTEX-14PN4-2526-SM-686ZG
Minor Salivary Gland	Salivary Gland	GTEX-15CHC-1226-SM-686ZH
Minor Salivary Gland	Salivary Gland	GTEX-15EU6-2626-SM-7KUN4
Minor Salivary Gland	Salivary Gland	GTEX-15RJE-2726-SM-6LPJ1
Minor Salivary Gland	Salivary Gland	GTEX-15SHV-2226-SM-6M464
Minor Salivary Gland	Salivary Gland	GTEX-178AV-1426-SM-6LLID
Minor Salivary Gland	Salivary Gland	GTEX-17KNJ-2726-SM-7KFRE
Minor Salivary Gland	Salivary Gland	GTEX-17MFQ-1626-SM-7938Z
Minor Salivary Gland	Salivary Gland	GTEX-18A67-0626-SM-7KFSL
Minor Salivary Gland	Salivary Gland	GTEX-18A7B-2826-SM-7KFTK
Minor Salivary Gland	Salivary Gland	GTEX-18D9U-2226-SM-7KFTN
Minor Salivary Gland	Salivary Gland	GTEX-18QFQ-0626-SM-718AW
Minor Salivary Gland	Salivary Gland	GTEX-1A3MV-1726-SM-72D68
Minor Salivary Gland	Salivary Gland	GTEX-1AMEY-1126-SM-72D79
Minor Salivary Gland	Salivary Gland	GTEX-1AX8Z-0626-SM-731EH
Minor Salivary Gland	Salivary Gland	GTEX-1AX9K-2126-SM-72D7K
Minor Salivary Gland	Salivary Gland	GTEX-1B8KZ-1726-SM-73KV7
Minor Salivary Gland	Salivary Gland	GTEX-1B8SF-1826-SM-731DJ
Minor Salivary Gland	Salivary Gland	GTEX-1B932-0626-SM-731EM
Minor Salivary Gland	Salivary Gland	GTEX-1B97J-2126-SM-79OL6
Minor Salivary Gland	Salivary Gland	GTEX-1C4CL-2026-SM-731EB
Minor Salivary Gland	Salivary Gland	GTEX-1C64N-2926-SM-79OLM
Minor Salivary Gland	Salivary Gland	GTEX-1C64O-0626-SM-7DHL1
Minor Salivary Gland	Salivary Gland	GTEX-1CB4I-2526-SM-7MKG1
Minor Salivary Gland	Salivary Gland	GTEX-1EH9U-2926-SM-7DHLX
Minor Salivary Gland	Salivary Gland	GTEX-1GMR2-2026-SM-7MKHK
Minor Salivary Gland	Salivary Gland	GTEX-1GPI7-0326-SM-7MKH6
Minor Salivary Gland	Salivary Gland	GTEX-WFJO-2026-SM-4LVM3
Minor Salivary Gland	Salivary Gland	GTEX-WH7G-2126-SM-4M1XU
Minor Salivary Gland	Salivary Gland	GTEX-WHSB-2326-SM-4M1XK
Minor Salivary Gland	Salivary Gland	GTEX-WOFM-2126-SM-4OOSR
Minor Salivary Gland	Salivary Gland	GTEX-WY7C-1426-SM-4OND1
Minor Salivary Gland	Salivary Gland	GTEX-WYVS-2026-SM-4RGNS
Minor Salivary Gland	Salivary Gland	GTEX-X15G-2126-SM-4PQZR
Minor Salivary Gland	Salivary Gland	GTEX-X261-2426-SM-4PQZU
Minor Salivary Gland	Salivary Gland	GTEX-X3Y1-1926-SM-4PQZO
Minor Salivary Gland	Salivary Gland	GTEX-X5EB-1726-SM-4E3J7
Minor Salivary Gland	Salivary Gland	GTEX-XGQ4-2626-SM-4AT6B
Minor Salivary Gland	Salivary Gland	GTEX-XMK1-2626-SM-4B65R

Minor Salivary Gland	Salivary Gland	GTEX-XV7Q-2426-SM-4BRV8
Minor Salivary Gland	Salivary Gland	GTEX-Y111-2626-SM-4TT24
Minor Salivary Gland	Salivary Gland	GTEX-Y3I4-1926-SM-4TT8L
Minor Salivary Gland	Salivary Gland	GTEX-Y5LM-1826-SM-4VDT9
Minor Salivary Gland	Salivary Gland	GTEX-Y5V6-2226-SM-4VDTC
Minor Salivary Gland	Salivary Gland	GTEX-YB5K-1726-SM-5IFIZ
Minor Salivary Gland	Salivary Gland	GTEX-YF7O-2226-SM-5CVN8
Minor Salivary Gland	Salivary Gland	GTEX-ZA64-1726-SM-5CVMF
Minor Salivary Gland	Salivary Gland	GTEX-ZC5H-2726-SM-5KLZW
Minor Salivary Gland	Salivary Gland	GTEX-ZE7O-2926-SM-51MSG
Minor Salivary Gland	Salivary Gland	GTEX-ZF29-2126-SM-4WWB8
Minor Salivary Gland	Salivary Gland	GTEX-ZP4G-1926-SM-57WDZ
Minor Salivary Gland	Salivary Gland	GTEX-ZPU1-2326-SM-57WEP
Minor Salivary Gland	Salivary Gland	GTEX-ZQG8-2126-SM-4YCF1
Minor Salivary Gland	Salivary Gland	GTEX-ZQUD-2126-SM-51MSY
Minor Salivary Gland	Salivary Gland	GTEX-ZT9W-2126-SM-51MSF
Minor Salivary Gland	Salivary Gland	GTEX-ZXG5-2826-SM-5NQ8T
Muscle - Skeletal	Muscle	GTEX-1117F-0426-SM-5EGHI
Muscle - Skeletal	Muscle	GTEX-111CU-2026-SM-5GZZC
Muscle - Skeletal	Muscle	GTEX-111FC-0326-SM-5GZZ1
Muscle - Skeletal	Muscle	GTEX-111VG-2626-SM-5GZY2
Muscle - Skeletal	Muscle	GTEX-111YS-2326-SM-5987L
Muscle - Skeletal	Muscle	GTEX-1122O-2426-SM-5GIDN
Muscle - Skeletal	Muscle	GTEX-1128S-2426-SM-5H11B
Muscle - Skeletal	Muscle	GTEX-113JC-2726-SM-5EGIS
Muscle - Skeletal	Muscle	GTEX-117XS-2526-SM-5H11G
Muscle - Skeletal	Muscle	GTEX-117YW-2426-SM-5Q5AE
Muscle - Skeletal	Muscle	GTEX-117YX-2526-SM-5EQ4Q
Muscle - Skeletal	Muscle	GTEX-1192X-0426-SM-5GIEE
Muscle - Skeletal	Muscle	GTEX-11DXW-0726-SM-5H12J
Muscle - Skeletal	Muscle	GTEX-11DXX-2726-SM-5PNXO
Muscle - Skeletal	Muscle	GTEX-11DXY-2726-SM-5GID2
Muscle - Skeletal	Muscle	GTEX-11DXZ-2426-SM-5N9DT
Muscle - Skeletal	Muscle	GTEX-11DZ1-0926-SM-5EQ5R
Muscle - Skeletal	Muscle	GTEX-11EI6-0326-SM-5EQ6G
Muscle - Skeletal	Muscle	GTEX-11EM3-2126-SM-5H11M
Muscle - Skeletal	Muscle	GTEX-11EMC-2626-SM-59864
Muscle - Skeletal	Muscle	GTEX-11EQ8-0526-SM-5N9BC
Muscle - Skeletal	Muscle	GTEX-11EQ9-2126-SM-5PNVW
Muscle - Skeletal	Muscle	GTEX-11GS4-2526-SM-5A5KT
Muscle - Skeletal	Muscle	GTEX-11GSO-2526-SM-5PNVX
Muscle - Skeletal	Muscle	GTEX-11GSP-2726-SM-5A5LJ
Muscle - Skeletal	Muscle	GTEX-11H98-0326-SM-5HL4S
Muscle - Skeletal	Muscle	GTEX-11I78-2426-SM-5A5K9
Muscle - Skeletal	Muscle	GTEX-11LCK-1226-SM-5Q5AM
Muscle - Skeletal	Muscle	GTEX-11NSD-2026-SM-5HL5U
Muscle - Skeletal	Muscle	GTEX-11NUK-0226-SM-5A5L4
Muscle - Skeletal	Muscle	GTEX-11NV4-0326-SM-5HL58
Muscle - Skeletal	Muscle	GTEX-11O72-0326-SM-5986D
Muscle - Skeletal	Muscle	GTEX-11OC5-0326-SM-5PNW5
Muscle - Skeletal	Muscle	GTEX-11OF3-2526-SM-5P9JL
Muscle - Skeletal	Muscle	GTEX-11ONC-2426-SM-5GU7H
Muscle - Skeletal	Muscle	GTEX-11P7K-1926-SM-5GU6R
Muscle - Skeletal	Muscle	GTEX-11P81-2526-SM-5GU6H

Muscle - Skeletal	Muscle	GTEX-11P82-1826-SM-5PNYJ
Muscle - Skeletal	Muscle	GTEX-11PRG-0326-SM-5GU53
Muscle - Skeletal	Muscle	GTEX-11TT1-2326-SM-5GU6N
Muscle - Skeletal	Muscle	GTEX-11TTK-0326-SM-5BC5N
Muscle - Skeletal	Muscle	GTEX-11TUW-2526-SM-5EQKN
Muscle - Skeletal	Muscle	GTEX-11UD1-0326-SM-5EQKX
Muscle - Skeletal	Muscle	GTEX-11UD2-0326-SM-5EQLE
Muscle - Skeletal	Muscle	GTEX-11VI4-1926-SM-5CVLI
Muscle - Skeletal	Muscle	GTEX-11WQC-2626-SM-5CVLF
Muscle - Skeletal	Muscle	GTEX-11WQK-0726-SM-5GU71
Muscle - Skeletal	Muscle	GTEX-11XUK-2226-SM-5CVLL
Muscle - Skeletal	Muscle	GTEX-11ZTS-0526-SM-5CVL9
Muscle - Skeletal	Muscle	GTEX-11ZTT-2626-SM-5EQKK
Muscle - Skeletal	Muscle	GTEX-11ZU8-2326-SM-5EQMJ
Muscle - Skeletal	Muscle	GTEX-11ZUS-1426-SM-5FQSP
Muscle - Skeletal	Muscle	GTEX-11ZVC-2726-SM-5FQSZ
Muscle - Skeletal	Muscle	GTEX-1211K-2126-SM-59HJZ
Muscle - Skeletal	Muscle	GTEX-12126-0526-SM-5PNW4
Muscle - Skeletal	Muscle	GTEX-1212Z-2626-SM-5YY8P
Muscle - Skeletal	Muscle	GTEX-12584-0526-SM-5FQTS
Muscle - Skeletal	Muscle	GTEX-12696-2826-SM-5FQTY
Muscle - Skeletal	Muscle	GTEX-1269C-2626-SM-5EQ5C
Muscle - Skeletal	Muscle	GTEX-12BJ1-2526-SM-5FQUE
Muscle - Skeletal	Muscle	GTEX-12C56-1926-SM-5FQUG
Muscle - Skeletal	Muscle	GTEX-12KS4-0826-SM-5BC5R
Muscle - Skeletal	Muscle	GTEX-12WSA-0726-SM-5FQU2
Muscle - Skeletal	Muscle	GTEX-12WSC-0326-SM-5EQ5E
Muscle - Skeletal	Muscle	GTEX-12WSD-0526-SM-5EQ6F
Muscle - Skeletal	Muscle	GTEX-12WSF-0926-SM-6PALU
Muscle - Skeletal	Muscle	GTEX-12WSG-2526-SM-5EQ4O
Muscle - Skeletal	Muscle	GTEX-12WSH-2526-SM-5CVNH
Muscle - Skeletal	Muscle	GTEX-12WSJ-1726-SM-5LZVV
Muscle - Skeletal	Muscle	GTEX-12WSL-2726-SM-5CVNL
Muscle - Skeletal	Muscle	GTEX-12WSM-0326-SM-5LZVI
Muscle - Skeletal	Muscle	GTEX-12WSN-2526-SM-5LZU8
Muscle - Skeletal	Muscle	GTEX-12ZZX-0326-SM-5DUVH
Muscle - Skeletal	Muscle	GTEX-12ZZY-0626-SM-5EQ5T
Muscle - Skeletal	Muscle	GTEX-12ZZZ-0226-SM-59HKU
Muscle - Skeletal	Muscle	GTEX-13111-2226-SM-5LZUE
Muscle - Skeletal	Muscle	GTEX-13112-2626-SM-5BC66
Muscle - Skeletal	Muscle	GTEX-13113-5019-SM-7EPH2
Muscle - Skeletal	Muscle	GTEX-1313W-0526-SM-5P9FZ
Muscle - Skeletal	Muscle	GTEX-1314G-1726-SM-5LZUX
Muscle - Skeletal	Muscle	GTEX-131XE-2726-SM-5P9G7
Muscle - Skeletal	Muscle	GTEX-131XF-2326-SM-5BC6B
Muscle - Skeletal	Muscle	GTEX-131XG-2326-SM-5HL8F
Muscle - Skeletal	Muscle	GTEX-131XH-2726-SM-5GCO2
Muscle - Skeletal	Muscle	GTEX-131XW-0326-SM-5KM1J
Muscle - Skeletal	Muscle	GTEX-131YS-0526-SM-5EGKJ
Muscle - Skeletal	Muscle	GTEX-132AR-1026-SM-5PNVL
Muscle - Skeletal	Muscle	GTEX-132NY-0726-SM-5KLZM
Muscle - Skeletal	Muscle	GTEX-132Q8-0626-SM-5L3F3
Muscle - Skeletal	Muscle	GTEX-1339X-2426-SM-5KLYU
Muscle - Skeletal	Muscle	GTEX-133LE-2026-SM-5K7WM

Muscle - Skeletal	Muscle	GTEX-1399Q-2426-SM-5J2MO
Muscle - Skeletal	Muscle	GTEX-1399R-2526-SM-5IFG2
Muscle - Skeletal	Muscle	GTEX-1399S-2726-SM-5KM22
Muscle - Skeletal	Muscle	GTEX-1399T-2426-SM-5L3FJ
Muscle - Skeletal	Muscle	GTEX-1399U-2526-SM-5L3DL
Muscle - Skeletal	Muscle	GTEX-139D8-0726-SM-5P9GJ
Muscle - Skeletal	Muscle	GTEX-139T4-0326-SM-5K7XN
Muscle - Skeletal	Muscle	GTEX-139T6-2026-SM-5IFFL
Muscle - Skeletal	Muscle	GTEX-139T8-0526-SM-5KLZL
Muscle - Skeletal	Muscle	GTEX-139TS-2626-SM-5J1MT
Muscle - Skeletal	Muscle	GTEX-139TT-0426-SM-5IJFA
Muscle - Skeletal	Muscle	GTEX-139TU-0526-SM-5J2M8
Muscle - Skeletal	Muscle	GTEX-139UW-2626-SM-5K7W1
Muscle - Skeletal	Muscle	GTEX-139YR-2526-SM-5IJC6
Muscle - Skeletal	Muscle	GTEX-13CF2-2726-SM-5LZWW
Muscle - Skeletal	Muscle	GTEX-13CF3-1826-SM-5J1NK
Muscle - Skeletal	Muscle	GTEX-13D11-2526-SM-5KM28
Muscle - Skeletal	Muscle	GTEX-13FH7-2126-SM-5L3DJ
Muscle - Skeletal	Muscle	GTEX-13FHO-0726-SM-5IJDG
Muscle - Skeletal	Muscle	GTEX-13FHP-0526-SM-5K7UY
Muscle - Skeletal	Muscle	GTEX-13FTW-2326-SM-5K7YS
Muscle - Skeletal	Muscle	GTEX-13FTX-2026-SM-5L3D6
Muscle - Skeletal	Muscle	GTEX-13FTY-0226-SM-5IJBW
Muscle - Skeletal	Muscle	GTEX-13FTZ-2226-SM-5LZXI
Muscle - Skeletal	Muscle	GTEX-13FXS-0326-SM-5K7TV
Muscle - Skeletal	Muscle	GTEX-13G51-2726-SM-5LZZ9
Muscle - Skeletal	Muscle	GTEX-13IVO-0626-SM-5LZYJ
Muscle - Skeletal	Muscle	GTEX-13JUV-2326-SM-5IJCE
Muscle - Skeletal	Muscle	GTEX-13JVG-0126-SM-5L3D3
Muscle - Skeletal	Muscle	GTEX-13N11-2726-SM-5L3ER
Muscle - Skeletal	Muscle	GTEX-13N1W-0526-SM-5MR4I
Muscle - Skeletal	Muscle	GTEX-13N2G-2326-SM-5J1ON
Muscle - Skeletal	Muscle	GTEX-13NYB-2726-SM-5J2MT
Muscle - Skeletal	Muscle	GTEX-13NYC-0726-SM-5J2NQ
Muscle - Skeletal	Muscle	GTEX-13NYS-0326-SM-5MR4L
Muscle - Skeletal	Muscle	GTEX-13NZ8-2126-SM-5L3EO
Muscle - Skeletal	Muscle	GTEX-13NZ9-0626-SM-5IJCO
Muscle - Skeletal	Muscle	GTEX-13NZA-0526-SM-5L3D7
Muscle - Skeletal	Muscle	GTEX-13NZB-2626-SM-5J2M7
Muscle - Skeletal	Muscle	GTEX-13O1R-0526-SM-5K7XU
Muscle - Skeletal	Muscle	GTEX-13O21-1726-SM-5MR5A
Muscle - Skeletal	Muscle	GTEX-13O3O-0526-SM-5J1NL
Muscle - Skeletal	Muscle	GTEX-13O3P-0626-SM-5L3D5
Muscle - Skeletal	Muscle	GTEX-13O3Q-2726-SM-5KM51
Muscle - Skeletal	Muscle	GTEX-13O61-2326-SM-5J1NJ
Muscle - Skeletal	Muscle	GTEX-13OVG-2126-SM-5KM1K
Muscle - Skeletal	Muscle	GTEX-13OVH-0626-SM-5L3EI
Muscle - Skeletal	Muscle	GTEX-13OVI-1726-SM-5L3DW
Muscle - Skeletal	Muscle	GTEX-13OVJ-0226-SM-5L3GK
Muscle - Skeletal	Muscle	GTEX-13OVK-1926-SM-6M48G
Muscle - Skeletal	Muscle	GTEX-13OVL-1426-SM-5IJC1
Muscle - Skeletal	Muscle	GTEX-13OW5-0326-SM-5L3HK
Muscle - Skeletal	Muscle	GTEX-13OW6-0626-SM-5L3IB
Muscle - Skeletal	Muscle	GTEX-13OW7-0326-SM-5J1MP

Muscle - Skeletal	Muscle	GTEX-13OW8-1426-SM-5IJDT
Muscle - Skeletal	Muscle	GTEX-13PDP-0326-SM-5K7XP
Muscle - Skeletal	Muscle	GTEX-13PL6-0326-SM-5IJCP
Muscle - Skeletal	Muscle	GTEX-13PL7-0626-SM-5IJCD
Muscle - Skeletal	Muscle	GTEX-13PLJ-0326-SM-5L3I7
Muscle - Skeletal	Muscle	GTEX-13PVQ-0326-SM-5K7YF
Muscle - Skeletal	Muscle	GTEX-13PVR-2526-SM-5RQIT
Muscle - Skeletal	Muscle	GTEX-13QBU-2426-SM-5LU4L
Muscle - Skeletal	Muscle	GTEX-13QIC-1226-SM-5K7YR
Muscle - Skeletal	Muscle	GTEX-13QJ3-0726-SM-5SI68
Muscle - Skeletal	Muscle	GTEX-13RTJ-2726-SM-73KVG
Muscle - Skeletal	Muscle	GTEX-13RTK-1626-SM-5QGQS
Muscle - Skeletal	Muscle	GTEX-13RTL-0326-SM-7LT89
Muscle - Skeletal	Muscle	GTEX-13S86-2326-SM-5RQK1
Muscle - Skeletal	Muscle	GTEX-13SLW-0326-SM-5RQK5
Muscle - Skeletal	Muscle	GTEX-13SLX-0326-SM-5Q5E4
Muscle - Skeletal	Muscle	GTEX-13U4I-1826-SM-5IJD2
Muscle - Skeletal	Muscle	GTEX-13VXT-0326-SM-5LU4U
Muscle - Skeletal	Muscle	GTEX-13VXU-0326-SM-5LU36
Muscle - Skeletal	Muscle	GTEX-13W3W-2626-SM-5SIA2
Muscle - Skeletal	Muscle	GTEX-13W46-0726-SM-5LU3Z
Muscle - Skeletal	Muscle	GTEX-13X6H-2326-SM-7EWEB
Muscle - Skeletal	Muscle	GTEX-13X6I-0526-SM-5QGP8
Muscle - Skeletal	Muscle	GTEX-13X6J-0326-SM-7EPGD
Muscle - Skeletal	Muscle	GTEX-13X6K-0326-SM-5Q5D6
Muscle - Skeletal	Muscle	GTEX-13YAN-0526-SM-5O9BE
Muscle - Skeletal	Muscle	GTEX-1445S-0626-SM-5LU3C
Muscle - Skeletal	Muscle	GTEX-144FL-0326-SM-5O99O
Muscle - Skeletal	Muscle	GTEX-144GL-0326-SM-5QGOY
Muscle - Skeletal	Muscle	GTEX-144GM-2026-SM-5LU3D
Muscle - Skeletal	Muscle	GTEX-144GN-2426-SM-5LU4R
Muscle - Skeletal	Muscle	GTEX-144GO-1326-SM-5Q5EL
Muscle - Skeletal	Muscle	GTEX-145LS-0526-SM-5LU8Y
Muscle - Skeletal	Muscle	GTEX-145LT-1626-SM-5O992
Muscle - Skeletal	Muscle	GTEX-145LU-2026-SM-5Q5E8
Muscle - Skeletal	Muscle	GTEX-145LV-2326-SM-5LU8Z
Muscle - Skeletal	Muscle	GTEX-145ME-2026-SM-5SIA5
Muscle - Skeletal	Muscle	GTEX-145MG-0226-SM-5TDDB
Muscle - Skeletal	Muscle	GTEX-145MH-0326-SM-5LU8H
Muscle - Skeletal	Muscle	GTEX-145MI-0326-SM-5Q5EV
Muscle - Skeletal	Muscle	GTEX-145MN-2426-SM-5NQA5
Muscle - Skeletal	Muscle	GTEX-145MO-0726-SM-5NQB9
Muscle - Skeletal	Muscle	GTEX-146FH-0526-SM-5QGQ3
Muscle - Skeletal	Muscle	GTEX-146FQ-0326-SM-5NQAX
Muscle - Skeletal	Muscle	GTEX-146FR-1726-SM-5Q5DT
Muscle - Skeletal	Muscle	GTEX-14753-0526-SM-5QGPR
Muscle - Skeletal	Muscle	GTEX-1477Z-1626-SM-5SIA8
Muscle - Skeletal	Muscle	GTEX-147F3-0226-SM-5NQBV
Muscle - Skeletal	Muscle	GTEX-147F4-0326-SM-5TDCK
Muscle - Skeletal	Muscle	GTEX-147GR-0126-SM-62LER
Muscle - Skeletal	Muscle	GTEX-147JS-0526-SM-5TDD3
Muscle - Skeletal	Muscle	GTEX-148VJ-0326-SM-5QGQE
Muscle - Skeletal	Muscle	GTEX-1497J-2626-SM-5SI9K
Muscle - Skeletal	Muscle	GTEX-14A5H-0326-SM-5RQIB

Muscle - Skeletal	Muscle	GTEX-14A5I-0326-SM-5NQ9J
Muscle - Skeletal	Muscle	GTEX-14A6H-5019-SM-793DH
Muscle - Skeletal	Muscle	GTEX-14ABY-0126-SM-62LDI
Muscle - Skeletal	Muscle	GTEX-14AS3-2126-SM-5S2MK
Muscle - Skeletal	Muscle	GTEX-14ASI-0526-SM-5QGQP
Muscle - Skeletal	Muscle	GTEX-14BIL-0326-SM-5SI9Q
Muscle - Skeletal	Muscle	GTEX-14BIM-0326-SM-5SI9B
Muscle - Skeletal	Muscle	GTEX-14BMU-2026-SM-5S2W6
Muscle - Skeletal	Muscle	GTEX-14BMV-0326-SM-5RQHW
Muscle - Skeletal	Muscle	GTEX-14C38-0426-SM-5TDDZ
Muscle - Skeletal	Muscle	GTEX-14C39-2426-SM-5S2U5
Muscle - Skeletal	Muscle	GTEX-14C5O-0626-SM-664N2
Muscle - Skeletal	Muscle	GTEX-14DAQ-0326-SM-664MR
Muscle - Skeletal	Muscle	GTEX-14DAR-1826-SM-664N1
Muscle - Skeletal	Muscle	GTEX-14E1K-2326-SM-664MP
Muscle - Skeletal	Muscle	GTEX-14E6C-1526-SM-664ND
Muscle - Skeletal	Muscle	GTEX-14E6D-0326-SM-62LDX
Muscle - Skeletal	Muscle	GTEX-14E6E-2026-SM-664N5
Muscle - Skeletal	Muscle	GTEX-14H4A-0926-SM-5ZZUU
Muscle - Skeletal	Muscle	GTEX-14ICL-1926-SM-5RQIZ
Muscle - Skeletal	Muscle	GTEX-14JG1-0126-SM-5ZZUP
Muscle - Skeletal	Muscle	GTEX-14JG6-2126-SM-6EU2F
Muscle - Skeletal	Muscle	GTEX-14JIY-0426-SM-62LF7
Muscle - Skeletal	Muscle	GTEX-14LLW-0526-SM-62LFB
Muscle - Skeletal	Muscle	GTEX-14LZ3-0626-SM-5ZZWP
Muscle - Skeletal	Muscle	GTEX-14PII-0626-SM-69LPY
Muscle - Skeletal	Muscle	GTEX-14PJ2-0326-SM-6EU2Q
Muscle - Skeletal	Muscle	GTEX-14PJ3-2026-SM-5ZZVK
Muscle - Skeletal	Muscle	GTEX-14PJ4-2426-SM-6EU1U
Muscle - Skeletal	Muscle	GTEX-14PJ5-2026-SM-6LLIN
Muscle - Skeletal	Muscle	GTEX-14PJ6-2326-SM-6ETZR
Muscle - Skeletal	Muscle	GTEX-14PJM-0426-SM-5ZZVN
Muscle - Skeletal	Muscle	GTEX-14PJN-2126-SM-6AJBM
Muscle - Skeletal	Muscle	GTEX-14PJO-0526-SM-6EU2O
Muscle - Skeletal	Muscle	GTEX-14PK6-2126-SM-6ETZV
Muscle - Skeletal	Muscle	GTEX-14PN4-0326-SM-6EU2N
Muscle - Skeletal	Muscle	GTEX-14PQA-0526-SM-7KUFS
Muscle - Skeletal	Muscle	GTEX-14XAO-2226-SM-6LLHU
Muscle - Skeletal	Muscle	GTEX-15CHC-2426-SM-6EU22
Muscle - Skeletal	Muscle	GTEX-15CHQ-0726-SM-6EU18
Muscle - Skeletal	Muscle	GTEX-15CHR-2426-SM-7DUFX
Muscle - Skeletal	Muscle	GTEX-15D79-0526-SM-6ETZP
Muscle - Skeletal	Muscle	GTEX-15DCD-0626-SM-7KUG7
Muscle - Skeletal	Muscle	GTEX-15DCZ-0526-SM-686Z3
Muscle - Skeletal	Muscle	GTEX-15DDE-0526-SM-6ETZQ
Muscle - Skeletal	Muscle	GTEX-15DZA-2026-SM-6PANE
Muscle - Skeletal	Muscle	GTEX-15EO6-2826-SM-6PANB
Muscle - Skeletal	Muscle	GTEX-15ER7-0226-SM-7KUEU
Muscle - Skeletal	Muscle	GTEX-15ETS-0326-SM-6PANH
Muscle - Skeletal	Muscle	GTEX-15F5U-1826-SM-6LLJF
Muscle - Skeletal	Muscle	GTEX-15G19-0526-SM-6PAMT
Muscle - Skeletal	Muscle	GTEX-15G1A-2026-SM-7KUFG
Muscle - Skeletal	Muscle	GTEX-15RIE-2426-SM-7KUDS
Muscle - Skeletal	Muscle	GTEX-15RIF-2126-SM-7KUM8

Muscle - Skeletal	Muscle	GTEX-15RIG-0426-SM-7KUF4
Muscle - Skeletal	Muscle	GTEX-15RJ7-2426-SM-6M48L
Muscle - Skeletal	Muscle	GTEX-15SB6-1826-SM-6PALN
Muscle - Skeletal	Muscle	GTEX-15SDE-2326-SM-6PAL4
Muscle - Skeletal	Muscle	GTEX-15SHU-0626-SM-7KUFH
Muscle - Skeletal	Muscle	GTEX-15SHV-2526-SM-6PAMG
Muscle - Skeletal	Muscle	GTEX-15SHW-0726-SM-7KUEI
Muscle - Skeletal	Muscle	GTEX-15SKB-2526-SM-6PALP
Muscle - Skeletal	Muscle	GTEX-15UF6-1026-SM-7KUF7
Muscle - Skeletal	Muscle	GTEX-15UF7-2226-SM-6PAMQ
Muscle - Skeletal	Muscle	GTEX-15UKP-0326-SM-7KUFP
Muscle - Skeletal	Muscle	GTEX-16AAH-1826-SM-7939D
Muscle - Skeletal	Muscle	GTEX-16BQI-0326-SM-6PAM4
Muscle - Skeletal	Muscle	GTEX-16GPK-0526-SM-7KUER
Muscle - Skeletal	Muscle	GTEX-16MT8-0326-SM-6PAM5
Muscle - Skeletal	Muscle	GTEX-16MTA-2026-SM-7KUL3
Muscle - Skeletal	Muscle	GTEX-16NGA-1926-SM-7LTA6
Muscle - Skeletal	Muscle	GTEX-16NPV-2426-SM-6M482
Muscle - Skeletal	Muscle	GTEX-16NPX-0626-SM-7KULB
Muscle - Skeletal	Muscle	GTEX-16XZY-2226-SM-7938N
Muscle - Skeletal	Muscle	GTEX-16XZZ-0526-SM-7KUF1
Muscle - Skeletal	Muscle	GTEX-16YQH-0426-SM-7KUL7
Muscle - Skeletal	Muscle	GTEX-16Z82-0126-SM-7DHLF
Muscle - Skeletal	Muscle	GTEX-17EUY-0626-SM-793BE
Muscle - Skeletal	Muscle	GTEX-17EVP-1326-SM-7DHMR
Muscle - Skeletal	Muscle	GTEX-17EVQ-1226-SM-7LG59
Muscle - Skeletal	Muscle	GTEX-17F96-2726-SM-7LT9F
Muscle - Skeletal	Muscle	GTEX-17F97-0526-SM-7DUF8
Muscle - Skeletal	Muscle	GTEX-17F98-1626-SM-7938R
Muscle - Skeletal	Muscle	GTEX-17F9E-0226-SM-79OKK
Muscle - Skeletal	Muscle	GTEX-17GQL-1926-SM-718B9
Muscle - Skeletal	Muscle	GTEX-17HG3-2726-SM-7DHL5
Muscle - Skeletal	Muscle	GTEX-17HGU-0626-SM-7939Y
Muscle - Skeletal	Muscle	GTEX-17HHE-2126-SM-7DHLO
Muscle - Skeletal	Muscle	GTEX-17HHY-0326-SM-7938H
Muscle - Skeletal	Muscle	GTEX-17HII-0426-SM-793A3
Muscle - Skeletal	Muscle	GTEX-17JCI-0326-SM-7LG5H
Muscle - Skeletal	Muscle	GTEX-17KNJ-2426-SM-7DHLR
Muscle - Skeletal	Muscle	GTEX-17MF6-2026-SM-7LG5I
Muscle - Skeletal	Muscle	GTEX-17MFQ-1926-SM-793A6
Muscle - Skeletal	Muscle	GTEX-183FY-0226-SM-7LG54
Muscle - Skeletal	Muscle	GTEX-183WM-0426-SM-731B1
Muscle - Skeletal	Muscle	GTEX-18465-0326-SM-7LTAD
Muscle - Skeletal	Muscle	GTEX-18A66-0126-SM-731CQ
Muscle - Skeletal	Muscle	GTEX-18A67-0326-SM-7LG5X
Muscle - Skeletal	Muscle	GTEX-18A6Q-0326-SM-718AT
Muscle - Skeletal	Muscle	GTEX-18A7A-0626-SM-718BJ
Muscle - Skeletal	Muscle	GTEX-18A7B-0326-SM-7LG65
Muscle - Skeletal	Muscle	GTEX-18D9A-1826-SM-718BK
Muscle - Skeletal	Muscle	GTEX-18D9B-0426-SM-7LTAR
Muscle - Skeletal	Muscle	GTEX-18D9U-0626-SM-7LTA4
Muscle - Skeletal	Muscle	GTEX-18QFQ-0326-SM-731C3
Muscle - Skeletal	Muscle	GTEX-1A32A-0526-SM-731B9
Muscle - Skeletal	Muscle	GTEX-1A3MV-2026-SM-718BU

Muscle - Skeletal	Muscle	GTEX-1A3MW-0526-SM-731BY
Muscle - Skeletal	Muscle	GTEX-1A3MX-0526-SM-718A9
Muscle - Skeletal	Muscle	GTEX-1A8G6-0126-SM-7DUFY
Muscle - Skeletal	Muscle	GTEX-1A8G7-0326-SM-72D5B
Muscle - Skeletal	Muscle	GTEX-1AMEY-1526-SM-73KYW
Muscle - Skeletal	Muscle	GTEX-1AMFI-2226-SM-731C6
Muscle - Skeletal	Muscle	GTEX-1AX8Z-0326-SM-731BU
Muscle - Skeletal	Muscle	GTEX-1AX9I-0326-SM-73KXQ
Muscle - Skeletal	Muscle	GTEX-1AX9J-0126-SM-73KY2
Muscle - Skeletal	Muscle	GTEX-1AX9K-2726-SM-7DHMA
Muscle - Skeletal	Muscle	GTEX-1AYD5-2226-SM-73KX3
Muscle - Skeletal	Muscle	GTEX-1B8KE-2226-SM-7939K
Muscle - Skeletal	Muscle	GTEX-1B8KZ-2126-SM-73KYG
Muscle - Skeletal	Muscle	GTEX-1B8L1-2726-SM-793AL
Muscle - Skeletal	Muscle	GTEX-1B8SF-0426-SM-79OL4
Muscle - Skeletal	Muscle	GTEX-1B8SG-0326-SM-73KXW
Muscle - Skeletal	Muscle	GTEX-1B932-0526-SM-73KU7
Muscle - Skeletal	Muscle	GTEX-1B933-0426-SM-7DHMC
Muscle - Skeletal	Muscle	GTEX-1B97I-2026-SM-7DHN2
Muscle - Skeletal	Muscle	GTEX-1B996-0326-SM-7DHMU
Muscle - Skeletal	Muscle	GTEX-1BAJH-0126-SM-7DHMF
Muscle - Skeletal	Muscle	GTEX-1C2JI-2126-SM-793CG
Muscle - Skeletal	Muscle	GTEX-1C475-1726-SM-73KVX
Muscle - Skeletal	Muscle	GTEX-1C4CL-0326-SM-7DHMH
Muscle - Skeletal	Muscle	GTEX-1C64O-0326-SM-73KVW
Muscle - Skeletal	Muscle	GTEX-1C6VQ-0326-SM-7DHMS
Muscle - Skeletal	Muscle	GTEX-1C6VR-2526-SM-7DHMT
Muscle - Skeletal	Muscle	GTEX-1C6WA-0326-SM-7DUGE
Muscle - Skeletal	Muscle	GTEX-1CAMR-1726-SM-793CU
Muscle - Skeletal	Muscle	GTEX-1CAMS-0126-SM-79396
Muscle - Skeletal	Muscle	GTEX-1CB4E-2126-SM-793AP
Muscle - Skeletal	Muscle	GTEX-1CB4I-0326-SM-7DHMM
Muscle - Skeletal	Muscle	GTEX-1E1VI-0426-SM-7DHN1
Muscle - Skeletal	Muscle	GTEX-N7MS-0426-SM-2YUN6
Muscle - Skeletal	Muscle	GTEX-NFK9-0626-SM-2HMIV
Muscle - Skeletal	Muscle	GTEX-O5YT-1626-SM-32PK6
Muscle - Skeletal	Muscle	GTEX-O5YV-2026-SM-2D7VS
Muscle - Skeletal	Muscle	GTEX-OHPK-1626-SM-2YUN3
Muscle - Skeletal	Muscle	GTEX-OHPL-1626-SM-2HMIR
Muscle - Skeletal	Muscle	GTEX-OHPN-2726-SM-2I5H4
Muscle - Skeletal	Muscle	GTEX-OIZG-1326-SM-2HMIQ
Muscle - Skeletal	Muscle	GTEX-OIZH-1626-SM-2HMKI
Muscle - Skeletal	Muscle	GTEX-OIZI-0626-SM-2XCEH
Muscle - Skeletal	Muscle	GTEX-OOBJ-1626-SM-2I3F7
Muscle - Skeletal	Muscle	GTEX-OOBK-1626-SM-2HMKG
Muscle - Skeletal	Muscle	GTEX-OXRK-1826-SM-2HMJE
Muscle - Skeletal	Muscle	GTEX-OXRL-1626-SM-2YUMU
Muscle - Skeletal	Muscle	GTEX-OXRN-1326-SM-3LK5V
Muscle - Skeletal	Muscle	GTEX-OXRP-2326-SM-2S1NL
Muscle - Skeletal	Muscle	GTEX-P44H-0426-SM-2XCEZ
Muscle - Skeletal	Muscle	GTEX-P4PP-1626-SM-2HMJF
Muscle - Skeletal	Muscle	GTEX-P4PQ-1626-SM-2HMKK
Muscle - Skeletal	Muscle	GTEX-P4QS-1626-SM-2S1NH
Muscle - Skeletal	Muscle	GTEX-P4QT-1626-SM-5S2UR

Muscle - Skeletal	Muscle	GTEX-P78B-1626-SM-5SI85
Muscle - Skeletal	Muscle	GTEX-PLZ4-0926-SM-2S1OI
Muscle - Skeletal	Muscle	GTEX-PLZ5-1726-SM-2I5F6
Muscle - Skeletal	Muscle	GTEX-PLZ6-1526-SM-2S1OC
Muscle - Skeletal	Muscle	GTEX-POMQ-1926-SM-3NB1Y
Muscle - Skeletal	Muscle	GTEX-POYW-0526-SM-2XCEY
Muscle - Skeletal	Muscle	GTEX-PSDG-0426-SM-5SI86
Muscle - Skeletal	Muscle	GTEX-PW2O-1726-SM-2S1OO
Muscle - Skeletal	Muscle	GTEX-PWCY-2026-SM-5SI8R
Muscle - Skeletal	Muscle	GTEX-PWN1-1626-SM-2S1OL
Muscle - Skeletal	Muscle	GTEX-PX3G-1626-SM-2S1PT
Muscle - Skeletal	Muscle	GTEX-Q2AG-0426-SM-2S1PU
Muscle - Skeletal	Muscle	GTEX-Q2AH-1826-SM-2S1Q2
Muscle - Skeletal	Muscle	GTEX-Q2AI-1526-SM-3GIJ3
Muscle - Skeletal	Muscle	GTEX-Q734-2026-SM-3GADA
Muscle - Skeletal	Muscle	GTEX-QCQG-2126-SM-2S1P8
Muscle - Skeletal	Muscle	GTEX-QDT8-0526-SM-3NMD8
Muscle - Skeletal	Muscle	GTEX-QDVJ-1926-SM-2S1PJ
Muscle - Skeletal	Muscle	GTEX-QDVN-2426-SM-2S1Q4
Muscle - Skeletal	Muscle	GTEX-QEG4-0626-SM-2S1OY
Muscle - Skeletal	Muscle	GTEX-QEG5-0426-SM-2I5GJ
Muscle - Skeletal	Muscle	GTEX-QEL4-0626-SM-3GIJM
Muscle - Skeletal	Muscle	GTEX-QESD-1626-SM-2S1RB
Muscle - Skeletal	Muscle	GTEX-QLQ7-1726-SM-2S1QQ
Muscle - Skeletal	Muscle	GTEX-QLQW-1326-SM-2S1QS
Muscle - Skeletal	Muscle	GTEX-QV31-1426-SM-2S1QD
Muscle - Skeletal	Muscle	GTEX-QV44-2026-SM-2S1RD
Muscle - Skeletal	Muscle	GTEX-QVJO-0126-SM-3GIK4
Muscle - Skeletal	Muscle	GTEX-QVUS-0226-SM-3GIJY
Muscle - Skeletal	Muscle	GTEX-QXCU-1726-SM-2TC6L
Muscle - Skeletal	Muscle	GTEX-R3RS-0526-SM-3GADG
Muscle - Skeletal	Muscle	GTEX-R53T-1826-SM-3GIJX
Muscle - Skeletal	Muscle	GTEX-R55C-1726-SM-3GADJ
Muscle - Skeletal	Muscle	GTEX-R55D-0626-SM-3GAD5
Muscle - Skeletal	Muscle	GTEX-R55E-0526-SM-2TC6B
Muscle - Skeletal	Muscle	GTEX-R55G-2326-SM-2TC61
Muscle - Skeletal	Muscle	GTEX-REY6-0826-SM-2TF4S
Muscle - Skeletal	Muscle	GTEX-RM2N-1626-SM-2TF5N
Muscle - Skeletal	Muscle	GTEX-RN64-0326-SM-2TC5J
Muscle - Skeletal	Muscle	GTEX-RNOR-0526-SM-2TF4O
Muscle - Skeletal	Muscle	GTEX-RTLS-0526-SM-2TF64
Muscle - Skeletal	Muscle	GTEX-RU1J-1726-SM-2TF5S
Muscle - Skeletal	Muscle	GTEX-RU72-1326-SM-2TF6T
Muscle - Skeletal	Muscle	GTEX-RWS6-2126-SM-2XCAV
Muscle - Skeletal	Muscle	GTEX-RWSA-0726-SM-2XCBE
Muscle - Skeletal	Muscle	GTEX-S32W-2326-SM-2XCAW
Muscle - Skeletal	Muscle	GTEX-S33H-2226-SM-5S2UF
Muscle - Skeletal	Muscle	GTEX-S7PM-0526-SM-3NM92
Muscle - Skeletal	Muscle	GTEX-S95S-1426-SM-2XCDM
Muscle - Skeletal	Muscle	GTEX-SE5C-1826-SM-2XCE4
Muscle - Skeletal	Muscle	GTEX-SIU8-0526-SM-2XCDP
Muscle - Skeletal	Muscle	GTEX-SJXC-0526-SM-2XCFG
Muscle - Skeletal	Muscle	GTEX-SN8G-0326-SM-32PLG
Muscle - Skeletal	Muscle	GTEX-SNMC-1426-SM-2XCFM

Muscle - Skeletal	Muscle	GTEX-SNOS-1526-SM-32PLW
Muscle - Skeletal	Muscle	GTEX-SSA3-0326-SM-32QPS
Muscle - Skeletal	Muscle	GTEX-T2IS-2626-SM-32QPP
Muscle - Skeletal	Muscle	GTEX-T5JC-0626-SM-3NMA6
Muscle - Skeletal	Muscle	GTEX-T5JW-1826-SM-3GAE1
Muscle - Skeletal	Muscle	GTEX-T6MN-0526-SM-32PMS
Muscle - Skeletal	Muscle	GTEX-T8EM-1326-SM-3DB7G
Muscle - Skeletal	Muscle	GTEX-TKQ1-1426-SM-4GICK
Muscle - Skeletal	Muscle	GTEX-TKQ2-0826-SM-33HB6
Muscle - Skeletal	Muscle	GTEX-TML8-1826-SM-32QOR
Muscle - Skeletal	Muscle	GTEX-TMMY-0426-SM-33HBB
Muscle - Skeletal	Muscle	GTEX-TMZS-0326-SM-3DB9P
Muscle - Skeletal	Muscle	GTEX-TSE9-0526-SM-3DB7Z
Muscle - Skeletal	Muscle	GTEX-U3ZG-0326-SM-47JXN
Muscle - Skeletal	Muscle	GTEX-U3ZH-1926-SM-4DXTR
Muscle - Skeletal	Muscle	GTEX-U3ZM-1226-SM-3DB9G
Muscle - Skeletal	Muscle	GTEX-U3ZN-2226-SM-3DB88
Muscle - Skeletal	Muscle	GTEX-U412-0326-SM-3DB9L
Muscle - Skeletal	Muscle	GTEX-U4B1-1626-SM-3DB8N
Muscle - Skeletal	Muscle	GTEX-U8T8-1426-SM-3DB9H
Muscle - Skeletal	Muscle	GTEX-U8XE-0726-SM-3DB8O
Muscle - Skeletal	Muscle	GTEX-UJHI-1726-SM-3DB9B
Muscle - Skeletal	Muscle	GTEX-UJMC-1826-SM-3GADT
Muscle - Skeletal	Muscle	GTEX-UPJH-0526-SM-4IHK8
Muscle - Skeletal	Muscle	GTEX-UTHO-0726-SM-3GAEN
Muscle - Skeletal	Muscle	GTEX-V1D1-2426-SM-3GAER
Muscle - Skeletal	Muscle	GTEX-V955-2426-SM-3GAEF
Muscle - Skeletal	Muscle	GTEX-VJWN-0426-SM-3GIJI
Muscle - Skeletal	Muscle	GTEX-VJYA-1926-SM-3GIJJ
Muscle - Skeletal	Muscle	GTEX-VUSG-2626-SM-4KKZI
Muscle - Skeletal	Muscle	GTEX-W5WG-1926-SM-4KKZK
Muscle - Skeletal	Muscle	GTEX-WEY5-2026-SM-5SI8D
Muscle - Skeletal	Muscle	GTEX-WFG7-2226-SM-3GIKP
Muscle - Skeletal	Muscle	GTEX-WFG8-2426-SM-3GILL
Muscle - Skeletal	Muscle	GTEX-WFON-2326-SM-3LK7M
Muscle - Skeletal	Muscle	GTEX-WHPG-2226-SM-3NMBO
Muscle - Skeletal	Muscle	GTEX-WHSB-1826-SM-3TW8M
Muscle - Skeletal	Muscle	GTEX-WHSE-0526-SM-5GZZD
Muscle - Skeletal	Muscle	GTEX-WK11-2526-SM-3NM9Y
Muscle - Skeletal	Muscle	GTEX-WL46-0626-SM-3LK7R
Muscle - Skeletal	Muscle	GTEX-WOFL-0626-SM-3MJG3
Muscle - Skeletal	Muscle	GTEX-WOFM-1326-SM-3MJFR
Muscle - Skeletal	Muscle	GTEX-WRHK-1626-SM-3MJFH
Muscle - Skeletal	Muscle	GTEX-WRHU-0826-SM-3MJFN
Muscle - Skeletal	Muscle	GTEX-WWTW-0626-SM-4MVNS
Muscle - Skeletal	Muscle	GTEX-WWYW-0526-SM-3NB2W
Muscle - Skeletal	Muscle	GTEX-WXYG-2526-SM-3NB3F
Muscle - Skeletal	Muscle	GTEX-WY7C-2526-SM-3NB2N
Muscle - Skeletal	Muscle	GTEX-WYJK-1726-SM-3NM9U
Muscle - Skeletal	Muscle	GTEX-WYVS-2326-SM-3NMAI
Muscle - Skeletal	Muscle	GTEX-WZTO-0826-SM-3NM8Q
Muscle - Skeletal	Muscle	GTEX-X261-0326-SM-3NMD4
Muscle - Skeletal	Muscle	GTEX-X4EO-0526-SM-3P5Z3
Muscle - Skeletal	Muscle	GTEX-X4XX-0626-SM-3NMC1

Muscle - Skeletal	Muscle	GTEX-X4XY-0626-SM-4E3IN
Muscle - Skeletal	Muscle	GTEX-X5EB-2326-SM-46MW5
Muscle - Skeletal	Muscle	GTEX-X638-0326-SM-47JY1
Muscle - Skeletal	Muscle	GTEX-X88G-0326-SM-47JZ4
Muscle - Skeletal	Muscle	GTEX-X8HC-0526-SM-4E3JA
Muscle - Skeletal	Muscle	GTEX-XAJ8-1026-SM-47JY9
Muscle - Skeletal	Muscle	GTEX-XBEC-0626-SM-4QARY
Muscle - Skeletal	Muscle	GTEX-XBED-2626-SM-4E3J5
Muscle - Skeletal	Muscle	GTEX-XBEW-1026-SM-4QARV
Muscle - Skeletal	Muscle	GTEX-XGQ4-2326-SM-4AT53
Muscle - Skeletal	Muscle	GTEX-XMD2-0526-SM-4YCDI
Muscle - Skeletal	Muscle	GTEX-XOT4-0526-SM-4B66O
Muscle - Skeletal	Muscle	GTEX-XPT6-2026-SM-4B64V
Muscle - Skeletal	Muscle	GTEX-XPVG-2526-SM-4B66D
Muscle - Skeletal	Muscle	GTEX-XQ3S-0426-SM-4BOOA
Muscle - Skeletal	Muscle	GTEX-XQ8I-0626-SM-4BOPT
Muscle - Skeletal	Muscle	GTEX-XUW1-0826-SM-4BOP6
Muscle - Skeletal	Muscle	GTEX-XUYS-0326-SM-47JX2
Muscle - Skeletal	Muscle	GTEX-XUZC-2126-SM-4BRW8
Muscle - Skeletal	Muscle	GTEX-XV7Q-2926-SM-4BRUL
Muscle - Skeletal	Muscle	GTEX-XYKS-2426-SM-4AT43
Muscle - Skeletal	Muscle	GTEX-Y111-0326-SM-4SOIX
Muscle - Skeletal	Muscle	GTEX-Y114-2526-SM-4TT8M
Muscle - Skeletal	Muscle	GTEX-Y3I4-2326-SM-4TT81
Muscle - Skeletal	Muscle	GTEX-Y3IK-2626-SM-4WWDK
Muscle - Skeletal	Muscle	GTEX-Y5LM-2126-SM-4VDSY
Muscle - Skeletal	Muscle	GTEX-Y5V5-2526-SM-4VBQ4
Muscle - Skeletal	Muscle	GTEX-Y5V6-2626-SM-5IFIX
Muscle - Skeletal	Muscle	GTEX-Y8DK-0326-SM-4TT3P
Muscle - Skeletal	Muscle	GTEX-Y8E4-1026-SM-4VBQ5
Muscle - Skeletal	Muscle	GTEX-Y8E5-0326-SM-57WBM
Muscle - Skeletal	Muscle	GTEX-Y8LW-2026-SM-4V6G8
Muscle - Skeletal	Muscle	GTEX-Y9LG-1926-SM-5IFHY
Muscle - Skeletal	Muscle	GTEX-YB5E-2226-SM-5IFJ5
Muscle - Skeletal	Muscle	GTEX-YB5K-2326-SM-5IFHD
Muscle - Skeletal	Muscle	GTEX-YBZK-0326-SM-59HLN
Muscle - Skeletal	Muscle	GTEX-YEC3-2126-SM-4YCDB
Muscle - Skeletal	Muscle	GTEX-YEC4-2226-SM-5IFJT
Muscle - Skeletal	Muscle	GTEX-YECK-0626-SM-4W1ZE
Muscle - Skeletal	Muscle	GTEX-YF7O-2526-SM-5IFJX
Muscle - Skeletal	Muscle	GTEX-YFC4-1026-SM-5RQJM
Muscle - Skeletal	Muscle	GTEX-YFCO-1926-SM-4W212
Muscle - Skeletal	Muscle	GTEX-YJ8O-2826-SM-5CVM4
Muscle - Skeletal	Muscle	GTEX-Z93S-1326-SM-5CVMR
Muscle - Skeletal	Muscle	GTEX-Z9EW-1726-SM-5CVMO
Muscle - Skeletal	Muscle	GTEX-ZA64-2026-SM-5PNXT
Muscle - Skeletal	Muscle	GTEX-ZAB4-0426-SM-5HL7N
Muscle - Skeletal	Muscle	GTEX-ZAB5-2126-SM-5K7UN
Muscle - Skeletal	Muscle	GTEX-ZAJG-0526-SM-5S2MT
Muscle - Skeletal	Muscle	GTEX-ZAK1-0526-SM-5K7X4
Muscle - Skeletal	Muscle	GTEX-ZAKK-0326-SM-5EGHA
Muscle - Skeletal	Muscle	GTEX-ZC5H-0326-SM-5HL9F
Muscle - Skeletal	Muscle	GTEX-ZDTS-0526-SM-4WKH9
Muscle - Skeletal	Muscle	GTEX-ZDXO-0326-SM-4WKHJ

Muscle - Skeletal	Muscle	GTEX-ZDYS-1726-SM-5HL4Y
Muscle - Skeletal	Muscle	GTEX-ZE7O-0326-SM-51MTH
Muscle - Skeletal	Muscle	GTEX-ZE9C-2326-SM-5J1NT
Muscle - Skeletal	Muscle	GTEX-ZF28-0526-SM-4WKGW
Muscle - Skeletal	Muscle	GTEX-ZF2S-2126-SM-4WWAY
Muscle - Skeletal	Muscle	GTEX-ZF3C-0326-SM-4WWBF
Muscle - Skeletal	Muscle	GTEX-ZLV1-2126-SM-4WWD2
Muscle - Skeletal	Muscle	GTEX-ZP4G-2026-SM-57WEH
Muscle - Skeletal	Muscle	GTEX-ZPCL-2026-SM-57WFD
Muscle - Skeletal	Muscle	GTEX-ZPIC-2526-SM-57WDP
Muscle - Skeletal	Muscle	GTEX-ZQG8-1226-SM-51MRX
Muscle - Skeletal	Muscle	GTEX-ZQUD-1726-SM-51MRQ
Muscle - Skeletal	Muscle	GTEX-ZT9X-1826-SM-57WDU
Muscle - Skeletal	Muscle	GTEX-ZTPG-0126-SM-5O9AL
Muscle - Skeletal	Muscle	GTEX-ZTX8-1626-SM-51MRY
Muscle - Skeletal	Muscle	GTEX-ZUA1-0326-SM-5NQA3
Muscle - Skeletal	Muscle	GTEX-ZV68-0526-SM-59HKI
Muscle - Skeletal	Muscle	GTEX-ZV6S-2126-SM-4YCEM
Muscle - Skeletal	Muscle	GTEX-ZV7C-2426-SM-5NQ7Q
Muscle - Skeletal	Muscle	GTEX-ZVE1-0526-SM-5NQ71
Muscle - Skeletal	Muscle	GTEX-ZVE2-0626-SM-5GU7I
Muscle - Skeletal	Muscle	GTEX-ZVP2-2126-SM-5GU6E
Muscle - Skeletal	Muscle	GTEX-ZVT2-2426-SM-57WCQ
Muscle - Skeletal	Muscle	GTEX-ZVT3-0526-SM-5GIE9
Muscle - Skeletal	Muscle	GTEX-ZVT4-0326-SM-51MS5
Muscle - Skeletal	Muscle	GTEX-ZVTK-0626-SM-5GU6Y
Muscle - Skeletal	Muscle	GTEX-ZVZO-0326-SM-5HL4N
Muscle - Skeletal	Muscle	GTEX-ZVZP-2526-SM-57WBR
Muscle - Skeletal	Muscle	GTEX-ZWKS-0326-SM-5NQ7G
Muscle - Skeletal	Muscle	GTEX-ZXG5-0326-SM-5GICH
Muscle - Skeletal	Muscle	GTEX-ZY6K-2026-SM-5GZXQ
Muscle - Skeletal	Muscle	GTEX-ZYFC-0526-SM-5GIDF
Muscle - Skeletal	Muscle	GTEX-ZYFD-0326-SM-5NQ8I
Muscle - Skeletal	Muscle	GTEX-ZYFG-2426-SM-5GIE8
Muscle - Skeletal	Muscle	GTEX-ZYT6-1626-SM-5E45R
Muscle - Skeletal	Muscle	GTEX-ZYVF-0626-SM-5E43Q
Muscle - Skeletal	Muscle	GTEX-ZYW4-0526-SM-5GZZ5
Muscle - Skeletal	Muscle	GTEX-ZYY3-0526-SM-5E45G
Muscle - Skeletal	Muscle	GTEX-ZZ64-1526-SM-5E43K
Muscle - Skeletal	Muscle	GTEX-ZZPT-0626-SM-5GZXT
Muscle - Skeletal	Muscle	GTEX-ZZPU-2626-SM-5E45Y
Nerve - Tibial	Nerve	GTEX-111CU-2226-SM-5N9G5
Nerve - Tibial	Nerve	GTEX-111FC-0526-SM-5GZZ8
Nerve - Tibial	Nerve	GTEX-111VG-2526-SM-5GZXP
Nerve - Tibial	Nerve	GTEX-111YS-2126-SM-5EGGM
Nerve - Tibial	Nerve	GTEX-113IC-0526-SM-5HL76
Nerve - Tibial	Nerve	GTEX-117YX-2426-SM-5GZZJ
Nerve - Tibial	Nerve	GTEX-1192X-0326-SM-5H11U
Nerve - Tibial	Nerve	GTEX-11DXW-0426-SM-5H12E
Nerve - Tibial	Nerve	GTEX-11DXX-2526-SM-5H119
Nerve - Tibial	Nerve	GTEX-11DXZ-2526-SM-5N9BG
Nerve - Tibial	Nerve	GTEX-11DYG-0426-SM-5987J
Nerve - Tibial	Nerve	GTEX-11EI6-0426-SM-5EQ43
Nerve - Tibial	Nerve	GTEX-11EQ9-2326-SM-5HL5H

Nerve - Tibial	Nerve	GTEX-11GS4-2826-SM-5HL56
Nerve - Tibial	Nerve	GTEX-11GSO-2626-SM-5Q5AK
Nerve - Tibial	Nerve	GTEX-11GSP-2826-SM-5A5LK
Nerve - Tibial	Nerve	GTEX-11H98-0426-SM-5HL55
Nerve - Tibial	Nerve	GTEX-11LCK-1326-SM-5Q5AN
Nerve - Tibial	Nerve	GTEX-11NSD-1826-SM-5N9CF
Nerve - Tibial	Nerve	GTEX-11NUK-0426-SM-5HL5V
Nerve - Tibial	Nerve	GTEX-11O72-0526-SM-5Q5AO
Nerve - Tibial	Nerve	GTEX-11OC5-0526-SM-5N9EE
Nerve - Tibial	Nerve	GTEX-11ONC-2326-SM-5HL6P
Nerve - Tibial	Nerve	GTEX-11P7K-2226-SM-5GU56
Nerve - Tibial	Nerve	GTEX-11P81-2626-SM-5GU6T
Nerve - Tibial	Nerve	GTEX-11P82-1926-SM-5Q5AU
Nerve - Tibial	Nerve	GTEX-11PRG-0126-SM-5BC55
Nerve - Tibial	Nerve	GTEX-11TT1-2626-SM-5EGL2
Nerve - Tibial	Nerve	GTEX-11TTK-0426-SM-5EQLT
Nerve - Tibial	Nerve	GTEX-11UD2-0426-SM-5EQ5W
Nerve - Tibial	Nerve	GTEX-11VI4-2126-SM-5EGI1
Nerve - Tibial	Nerve	GTEX-11WQC-2826-SM-5GU7A
Nerve - Tibial	Nerve	GTEX-11XUK-2326-SM-5CVLM
Nerve - Tibial	Nerve	GTEX-11ZU8-2626-SM-5EQLL
Nerve - Tibial	Nerve	GTEX-11ZUS-1226-SM-5FQU8
Nerve - Tibial	Nerve	GTEX-11ZVC-2826-SM-5FQTC
Nerve - Tibial	Nerve	GTEX-1211K-2326-SM-5FQU7
Nerve - Tibial	Nerve	GTEX-12126-0626-SM-5Q5AQ
Nerve - Tibial	Nerve	GTEX-1212Z-2726-SM-5EQ59
Nerve - Tibial	Nerve	GTEX-12584-0326-SM-59HK2
Nerve - Tibial	Nerve	GTEX-12696-2626-SM-5EQ44
Nerve - Tibial	Nerve	GTEX-12BJ1-2626-SM-5FQUF
Nerve - Tibial	Nerve	GTEX-12C56-1826-SM-5EQ6D
Nerve - Tibial	Nerve	GTEX-12KS4-1126-SM-5FQUI
Nerve - Tibial	Nerve	GTEX-12WSD-0326-SM-59HKP
Nerve - Tibial	Nerve	GTEX-12WSF-0426-SM-7KUMT
Nerve - Tibial	Nerve	GTEX-12WSG-2726-SM-5EQ5M
Nerve - Tibial	Nerve	GTEX-12WSK-2626-SM-5GCOT
Nerve - Tibial	Nerve	GTEX-12WSL-2826-SM-5LZWL
Nerve - Tibial	Nerve	GTEX-12WSM-0426-SM-5LZVU
Nerve - Tibial	Nerve	GTEX-12ZZX-0426-SM-5DUVT
Nerve - Tibial	Nerve	GTEX-12ZZY-0426-SM-5LZW3
Nerve - Tibial	Nerve	GTEX-13111-2026-SM-5GCMN
Nerve - Tibial	Nerve	GTEX-13113-2026-SM-5LZU3
Nerve - Tibial	Nerve	GTEX-1313W-0326-SM-5LZU5
Nerve - Tibial	Nerve	GTEX-1314G-1826-SM-5LZVA
Nerve - Tibial	Nerve	GTEX-131XE-2526-SM-5EQ57
Nerve - Tibial	Nerve	GTEX-131XF-2426-SM-5BC6C
Nerve - Tibial	Nerve	GTEX-131XG-2526-SM-5HL7F
Nerve - Tibial	Nerve	GTEX-131XH-2526-SM-5GCND
Nerve - Tibial	Nerve	GTEX-131XW-0126-SM-5LZWD
Nerve - Tibial	Nerve	GTEX-131YS-0326-SM-5PNYW
Nerve - Tibial	Nerve	GTEX-132NY-0526-SM-5EQ4V
Nerve - Tibial	Nerve	GTEX-132QS-2426-SM-62LFI
Nerve - Tibial	Nerve	GTEX-1339X-2726-SM-5PNYU
Nerve - Tibial	Nerve	GTEX-133LE-2126-SM-5IJBX
Nerve - Tibial	Nerve	GTEX-1399R-2426-SM-5IJFC

Nerve - Tibial	Nerve	GTEX-1399S-2426-SM-5K7U3
Nerve - Tibial	Nerve	GTEX-139T4-0426-SM-5J1OQ
Nerve - Tibial	Nerve	GTEX-139T6-1726-SM-5PNZ3
Nerve - Tibial	Nerve	GTEX-139T8-0326-SM-5IJCM
Nerve - Tibial	Nerve	GTEX-139TT-0326-SM-5IJC3
Nerve - Tibial	Nerve	GTEX-139TU-0326-SM-62LFK
Nerve - Tibial	Nerve	GTEX-139YR-2626-SM-5K7U4
Nerve - Tibial	Nerve	GTEX-13CF2-2826-SM-5LZX9
Nerve - Tibial	Nerve	GTEX-13D11-2626-SM-5IJDB
Nerve - Tibial	Nerve	GTEX-13FH7-2426-SM-5K7US
Nerve - Tibial	Nerve	GTEX-13FHO-0526-SM-5K7VS
Nerve - Tibial	Nerve	GTEX-13FHP-0326-SM-5K7U2
Nerve - Tibial	Nerve	GTEX-13FLV-2326-SM-5K7UK
Nerve - Tibial	Nerve	GTEX-13FTY-0326-SM-5L3DN
Nerve - Tibial	Nerve	GTEX-13G51-2826-SM-5LZWV
Nerve - Tibial	Nerve	GTEX-13IVO-0426-SM-5LZY7
Nerve - Tibial	Nerve	GTEX-13JUV-2526-SM-5J1OD
Nerve - Tibial	Nerve	GTEX-13JVG-0526-SM-5IJBN
Nerve - Tibial	Nerve	GTEX-13N11-2826-SM-5KM4Z
Nerve - Tibial	Nerve	GTEX-13N1W-0426-SM-5MR46
Nerve - Tibial	Nerve	GTEX-13NYC-0626-SM-5K7U8
Nerve - Tibial	Nerve	GTEX-13NYS-0426-SM-5MR4X
Nerve - Tibial	Nerve	GTEX-13NZ9-0726-SM-5J1ME
Nerve - Tibial	Nerve	GTEX-13NZA-0326-SM-5J1MK
Nerve - Tibial	Nerve	GTEX-13O1R-0426-SM-5K7WG
Nerve - Tibial	Nerve	GTEX-13O21-1826-SM-5MR5M
Nerve - Tibial	Nerve	GTEX-13O3O-0326-SM-5K7W2
Nerve - Tibial	Nerve	GTEX-13O3P-0326-SM-5K7VM
Nerve - Tibial	Nerve	GTEX-13O3Q-2626-SM-5KM3S
Nerve - Tibial	Nerve	GTEX-13O61-2626-SM-5KM4U
Nerve - Tibial	Nerve	GTEX-13OVG-2226-SM-5K7V8
Nerve - Tibial	Nerve	GTEX-13OVI-1526-SM-5K7VX
Nerve - Tibial	Nerve	GTEX-13OVJ-0426-SM-5L3GX
Nerve - Tibial	Nerve	GTEX-13OVL-1526-SM-5K7WA
Nerve - Tibial	Nerve	GTEX-13OW6-0226-SM-5J1NB
Nerve - Tibial	Nerve	GTEX-13OW7-0526-SM-5J1N2
Nerve - Tibial	Nerve	GTEX-13OW8-1526-SM-5K7V2
Nerve - Tibial	Nerve	GTEX-13PL7-1926-SM-5J1MM
Nerve - Tibial	Nerve	GTEX-13PVQ-0426-SM-5J1OG
Nerve - Tibial	Nerve	GTEX-13PVR-2626-SM-7DHKQ
Nerve - Tibial	Nerve	GTEX-13QBU-2326-SM-5LU49
Nerve - Tibial	Nerve	GTEX-13QIC-1426-SM-5LU4V
Nerve - Tibial	Nerve	GTEX-13QJ3-0526-SM-5QGQY
Nerve - Tibial	Nerve	GTEX-13RTJ-2826-SM-5QGQH
Nerve - Tibial	Nerve	GTEX-13S7M-0426-SM-5S2US
Nerve - Tibial	Nerve	GTEX-13S86-2426-SM-5S2MX
Nerve - Tibial	Nerve	GTEX-13SLX-0126-SM-5S2MM
Nerve - Tibial	Nerve	GTEX-13VXT-0426-SM-5LU57
Nerve - Tibial	Nerve	GTEX-13VXU-0426-SM-5LU3I
Nerve - Tibial	Nerve	GTEX-13W3W-2726-SM-5SIAE
Nerve - Tibial	Nerve	GTEX-13W46-0526-SM-5IJBD
Nerve - Tibial	Nerve	GTEX-13X6J-0426-SM-5O9D3
Nerve - Tibial	Nerve	GTEX-13X6K-0426-SM-5O9DZ
Nerve - Tibial	Nerve	GTEX-13YAN-0626-SM-5O9BQ

Nerve - Tibial	Nerve	GTEX-144FL-0426-SM-5O9A1
Nerve - Tibial	Nerve	GTEX-144GL-0426-SM-5QGOZ
Nerve - Tibial	Nerve	GTEX-144GM-2126-SM-5LU3P
Nerve - Tibial	Nerve	GTEX-144GN-2526-SM-5LU54
Nerve - Tibial	Nerve	GTEX-145LT-1926-SM-5QGP5
Nerve - Tibial	Nerve	GTEX-145LU-0726-SM-5QGP2
Nerve - Tibial	Nerve	GTEX-145LV-2526-SM-5S2VA
Nerve - Tibial	Nerve	GTEX-145ME-2126-SM-5SIAH
Nerve - Tibial	Nerve	GTEX-145MH-1426-SM-5QGPO
Nerve - Tibial	Nerve	GTEX-145MO-0526-SM-5Q5F7
Nerve - Tibial	Nerve	GTEX-146FH-0326-SM-5QGPT
Nerve - Tibial	Nerve	GTEX-146FQ-0426-SM-5NQBA
Nerve - Tibial	Nerve	GTEX-146FR-1426-SM-5SIAV
Nerve - Tibial	Nerve	GTEX-14753-0126-SM-5S2TX
Nerve - Tibial	Nerve	GTEX-1477Z-1526-SM-5SI9V
Nerve - Tibial	Nerve	GTEX-147JS-0626-SM-5TDD4
Nerve - Tibial	Nerve	GTEX-14A5I-0426-SM-5NQ9V
Nerve - Tibial	Nerve	GTEX-14A6H-2626-SM-5NQBO
Nerve - Tibial	Nerve	GTEX-14AS3-2226-SM-5S2OX
Nerve - Tibial	Nerve	GTEX-14ASI-0326-SM-5S2VH
Nerve - Tibial	Nerve	GTEX-14BIL-0426-SM-5SIA3
Nerve - Tibial	Nerve	GTEX-14BIM-0426-SM-5SI9N
Nerve - Tibial	Nerve	GTEX-14BIN-0826-SM-793DR
Nerve - Tibial	Nerve	GTEX-14BMV-0426-SM-5TDEF
Nerve - Tibial	Nerve	GTEX-14C38-0626-SM-5S2TV
Nerve - Tibial	Nerve	GTEX-14C39-2726-SM-7DUEK
Nerve - Tibial	Nerve	GTEX-14C5O-0426-SM-5ZZUW
Nerve - Tibial	Nerve	GTEX-14DAQ-0426-SM-664MS
Nerve - Tibial	Nerve	GTEX-14DAR-2226-SM-5S2VT
Nerve - Tibial	Nerve	GTEX-14E6C-1026-SM-5SI6P
Nerve - Tibial	Nerve	GTEX-14E6E-2226-SM-5SI6F
Nerve - Tibial	Nerve	GTEX-14E7W-0426-SM-5S2O4
Nerve - Tibial	Nerve	GTEX-14ICK-0526-SM-6EU26
Nerve - Tibial	Nerve	GTEX-14JG6-1926-SM-6LLIG
Nerve - Tibial	Nerve	GTEX-14LLW-0326-SM-5ZZWD
Nerve - Tibial	Nerve	GTEX-14PHW-0526-SM-6LLIP
Nerve - Tibial	Nerve	GTEX-14PHX-2526-SM-62LEO
Nerve - Tibial	Nerve	GTEX-14PHY-2626-SM-62LF8
Nerve - Tibial	Nerve	GTEX-14PII-0426-SM-6LLIR
Nerve - Tibial	Nerve	GTEX-14PJ3-2226-SM-62LFD
Nerve - Tibial	Nerve	GTEX-14PJ4-2526-SM-6LLIT
Nerve - Tibial	Nerve	GTEX-14PJ6-2426-SM-6ETZS
Nerve - Tibial	Nerve	GTEX-14PJO-0326-SM-6LLHT
Nerve - Tibial	Nerve	GTEX-14PN3-2626-SM-6EU17
Nerve - Tibial	Nerve	GTEX-15CHC-2526-SM-686YX
Nerve - Tibial	Nerve	GTEX-15DDE-0326-SM-6LLJ5
Nerve - Tibial	Nerve	GTEX-15DYW-0326-SM-6PAN9
Nerve - Tibial	Nerve	GTEX-15EO6-2626-SM-6PALK
Nerve - Tibial	Nerve	GTEX-15ER7-0126-SM-6PAM2
Nerve - Tibial	Nerve	GTEX-15F5U-1926-SM-6PANL
Nerve - Tibial	Nerve	GTEX-15FZZ-1926-SM-7KFTQ
Nerve - Tibial	Nerve	GTEX-15G19-0326-SM-7KUGF
Nerve - Tibial	Nerve	GTEX-15RIG-0526-SM-7KFTP
Nerve - Tibial	Nerve	GTEX-15SKB-0426-SM-6PALQ

Nerve - Tibial	Nerve	GTEX-15UKP-0226-SM-7KFTD
Nerve - Tibial	Nerve	GTEX-16AAH-2126-SM-7LG4D
Nerve - Tibial	Nerve	GTEX-16BQI-0626-SM-6PAMS
Nerve - Tibial	Nerve	GTEX-16GPK-0326-SM-793BT
Nerve - Tibial	Nerve	GTEX-16MT9-0226-SM-793BN
Nerve - Tibial	Nerve	GTEX-16NGA-2026-SM-7LG4Q
Nerve - Tibial	Nerve	GTEX-16NPX-0426-SM-7EWDJ
Nerve - Tibial	Nerve	GTEX-16XZY-2326-SM-7EWDD
Nerve - Tibial	Nerve	GTEX-16XZZ-0326-SM-7EWD6
Nerve - Tibial	Nerve	GTEX-16YQH-0526-SM-7KUE8
Nerve - Tibial	Nerve	GTEX-178AV-1826-SM-7KUE2
Nerve - Tibial	Nerve	GTEX-17EUY-0226-SM-79OKV
Nerve - Tibial	Nerve	GTEX-17EVP-1126-SM-793C4
Nerve - Tibial	Nerve	GTEX-17EVQ-1026-SM-7IGOG
Nerve - Tibial	Nerve	GTEX-17F96-0326-SM-793BR
Nerve - Tibial	Nerve	GTEX-17F97-0126-SM-7LT8N
Nerve - Tibial	Nerve	GTEX-17F98-0826-SM-7DUF7
Nerve - Tibial	Nerve	GTEX-17F9E-0426-SM-7IGP1
Nerve - Tibial	Nerve	GTEX-17F9Y-0926-SM-79OJU
Nerve - Tibial	Nerve	GTEX-17HG3-2526-SM-7IGOD
Nerve - Tibial	Nerve	GTEX-17HGU-0426-SM-7DUES
Nerve - Tibial	Nerve	GTEX-17HHE-2226-SM-7EPGN
Nerve - Tibial	Nerve	GTEX-17HHY-0626-SM-7EPGF
Nerve - Tibial	Nerve	GTEX-17HII-0526-SM-7DHLP
Nerve - Tibial	Nerve	GTEX-17JCI-0426-SM-7LG4R
Nerve - Tibial	Nerve	GTEX-17MF6-0426-SM-79ONB
Nerve - Tibial	Nerve	GTEX-183FY-0426-SM-7MKGK
Nerve - Tibial	Nerve	GTEX-183WM-0526-SM-731BD
Nerve - Tibial	Nerve	GTEX-18465-0526-SM-7LG4K
Nerve - Tibial	Nerve	GTEX-18A66-0426-SM-718BF
Nerve - Tibial	Nerve	GTEX-18A6Q-0426-SM-731CC
Nerve - Tibial	Nerve	GTEX-18A7A-0426-SM-731B6
Nerve - Tibial	Nerve	GTEX-18A7B-0426-SM-7LG4T
Nerve - Tibial	Nerve	GTEX-18D9A-1726-SM-731BS
Nerve - Tibial	Nerve	GTEX-18D9B-0226-SM-731CV
Nerve - Tibial	Nerve	GTEX-18D9U-0826-SM-7LG5K
Nerve - Tibial	Nerve	GTEX-18QFQ-0426-SM-72D7E
Nerve - Tibial	Nerve	GTEX-1A32A-0326-SM-718B3
Nerve - Tibial	Nerve	GTEX-1A3MV-2326-SM-731CT
Nerve - Tibial	Nerve	GTEX-1A3MW-0326-SM-731EF
Nerve - Tibial	Nerve	GTEX-1A3MX-0326-SM-73KXV
Nerve - Tibial	Nerve	GTEX-1A8G6-0326-SM-72D6F
Nerve - Tibial	Nerve	GTEX-1A8G7-0526-SM-73KYA
Nerve - Tibial	Nerve	GTEX-1AMFI-2326-SM-731CI
Nerve - Tibial	Nerve	GTEX-1AX9I-0426-SM-73KY3
Nerve - Tibial	Nerve	GTEX-1AX9J-0526-SM-72D5O
Nerve - Tibial	Nerve	GTEX-1AX9K-2526-SM-7DUG4
Nerve - Tibial	Nerve	GTEX-1AYCT-2026-SM-793CJ
Nerve - Tibial	Nerve	GTEX-1B8KE-2426-SM-73KYE
Nerve - Tibial	Nerve	GTEX-1B8KZ-2326-SM-7DUG9
Nerve - Tibial	Nerve	GTEX-1B8L1-2926-SM-7DUGA
Nerve - Tibial	Nerve	GTEX-1B8SF-0326-SM-7EWEN
Nerve - Tibial	Nerve	GTEX-1B8SG-0426-SM-73KY9
Nerve - Tibial	Nerve	GTEX-1B932-0326-SM-7IGMV

Nerve - Tibial	Nerve	GTEX-1B97I-1826-SM-7939W
Nerve - Tibial	Nerve	GTEX-1B996-0526-SM-79OOE
Nerve - Tibial	Nerve	GTEX-1BAJH-0326-SM-73KWM
Nerve - Tibial	Nerve	GTEX-1C2JI-2026-SM-7IGML
Nerve - Tibial	Nerve	GTEX-1C475-2026-SM-793CI
Nerve - Tibial	Nerve	GTEX-1C6VQ-0626-SM-7EPHE
Nerve - Tibial	Nerve	GTEX-1C6VR-2726-SM-79OM7
Nerve - Tibial	Nerve	GTEX-1C6WA-0526-SM-7IGNZ
Nerve - Tibial	Nerve	GTEX-1CAMS-0526-SM-7EWEX
Nerve - Tibial	Nerve	GTEX-1CAV2-2526-SM-7IGQ7
Nerve - Tibial	Nerve	GTEX-1CB4I-0626-SM-79OM1
Nerve - Tibial	Nerve	GTEX-1CB4J-0526-SM-7DUGI
Nerve - Tibial	Nerve	GTEX-1E1VI-0526-SM-79OM2
Nerve - Tibial	Nerve	GTEX-1EWIQ-0426-SM-793D4
Nerve - Tibial	Nerve	GTEX-N7MS-0526-SM-4E3JP
Nerve - Tibial	Nerve	GTEX-NFK9-0426-SM-2YUNK
Nerve - Tibial	Nerve	GTEX-NPJ7-1726-SM-2YUNA
Nerve - Tibial	Nerve	GTEX-NPJ8-1726-SM-2YUNB
Nerve - Tibial	Nerve	GTEX-O5YT-1726-SM-3NMD7
Nerve - Tibial	Nerve	GTEX-O5YV-1826-SM-2YUNI
Nerve - Tibial	Nerve	GTEX-OHPK-1726-SM-48TC4
Nerve - Tibial	Nerve	GTEX-OHPN-2926-SM-3LK65
Nerve - Tibial	Nerve	GTEX-OIZI-0426-SM-2XCEF
Nerve - Tibial	Nerve	GTEX-OXRK-0426-SM-3NB2E
Nerve - Tibial	Nerve	GTEX-OXRN-1426-SM-3LK5U
Nerve - Tibial	Nerve	GTEX-OXRP-2526-SM-2S1NO
Nerve - Tibial	Nerve	GTEX-P44H-0526-SM-2XCF1
Nerve - Tibial	Nerve	GTEX-P4PP-1726-SM-2S1NS
Nerve - Tibial	Nerve	GTEX-P4PQ-1726-SM-3NB15
Nerve - Tibial	Nerve	GTEX-P4QS-1726-SM-3NB1V
Nerve - Tibial	Nerve	GTEX-P4QT-1726-SM-2S1NQ
Nerve - Tibial	Nerve	GTEX-P78B-0626-SM-5S2W4
Nerve - Tibial	Nerve	GTEX-PLZ5-1626-SM-3NB21
Nerve - Tibial	Nerve	GTEX-PLZ6-1726-SM-2S1O6
Nerve - Tibial	Nerve	GTEX-POMQ-2026-SM-2S1OD
Nerve - Tibial	Nerve	GTEX-POYW-0426-SM-2XCEV
Nerve - Tibial	Nerve	GTEX-PSDG-0626-SM-2S1OE
Nerve - Tibial	Nerve	GTEX-PVOW-0626-SM-2XCF6
Nerve - Tibial	Nerve	GTEX-PW2O-1826-SM-5SI7R
Nerve - Tibial	Nerve	GTEX-PWCY-2126-SM-48TEC
Nerve - Tibial	Nerve	GTEX-PWN1-1726-SM-2S1O9
Nerve - Tibial	Nerve	GTEX-Q2AG-0626-SM-2S1PV
Nerve - Tibial	Nerve	GTEX-Q2AH-1926-SM-2S1PN
Nerve - Tibial	Nerve	GTEX-QCQG-2026-SM-2S1PH
Nerve - Tibial	Nerve	GTEX-QDT8-0326-SM-32PL1
Nerve - Tibial	Nerve	GTEX-QDVN-2226-SM-2S1PM
Nerve - Tibial	Nerve	GTEX-QEG4-1126-SM-2S1P7
Nerve - Tibial	Nerve	GTEX-QEG5-0526-SM-5S2V3
Nerve - Tibial	Nerve	GTEX-QEL4-0426-SM-3GACZ
Nerve - Tibial	Nerve	GTEX-QESD-1726-SM-2S1R7
Nerve - Tibial	Nerve	GTEX-QLQ7-1826-SM-2S1R5
Nerve - Tibial	Nerve	GTEX-QLQW-1426-SM-2S1QU
Nerve - Tibial	Nerve	GTEX-QVJO-0326-SM-2S1R1
Nerve - Tibial	Nerve	GTEX-QXCU-0126-SM-2TC5Q

Nerve - Tibial	Nerve	GTEX-R55C-1926-SM-2TF4K
Nerve - Tibial	Nerve	GTEX-R55E-0426-SM-2TC65
Nerve - Tibial	Nerve	GTEX-R55G-2126-SM-2TC67
Nerve - Tibial	Nerve	GTEX-REY6-0626-SM-2TF4G
Nerve - Tibial	Nerve	GTEX-RNOR-0326-SM-2TF51
Nerve - Tibial	Nerve	GTEX-RTLS-0326-SM-2TF6A
Nerve - Tibial	Nerve	GTEX-RU72-1126-SM-2TF6H
Nerve - Tibial	Nerve	GTEX-RUSQ-1826-SM-2TF6E
Nerve - Tibial	Nerve	GTEX-RWS6-2426-SM-2XCB9
Nerve - Tibial	Nerve	GTEX-S32W-2526-SM-2XCB8
Nerve - Tibial	Nerve	GTEX-S33H-2326-SM-2XCB3
Nerve - Tibial	Nerve	GTEX-S95S-1526-SM-2XCDH
Nerve - Tibial	Nerve	GTEX-SIU8-0326-SM-2XCDR
Nerve - Tibial	Nerve	GTEX-SJXC-0326-SM-2XCFI
Nerve - Tibial	Nerve	GTEX-SN8G-0426-SM-32PLF
Nerve - Tibial	Nerve	GTEX-SNMC-1626-SM-4DM52
Nerve - Tibial	Nerve	GTEX-SNOS-1626-SM-3NMA4
Nerve - Tibial	Nerve	GTEX-SSA3-0526-SM-32QPL
Nerve - Tibial	Nerve	GTEX-T2IS-1126-SM-4DM6Z
Nerve - Tibial	Nerve	GTEX-T2YK-0326-SM-4DM7D
Nerve - Tibial	Nerve	GTEX-T6MN-0326-SM-32PMK
Nerve - Tibial	Nerve	GTEX-T6MO-2026-SM-33HB4
Nerve - Tibial	Nerve	GTEX-T8EM-1526-SM-3DB7I
Nerve - Tibial	Nerve	GTEX-TKQ1-1326-SM-4DXU7
Nerve - Tibial	Nerve	GTEX-TML8-1626-SM-32QOO
Nerve - Tibial	Nerve	GTEX-TMMY-0526-SM-33HBC
Nerve - Tibial	Nerve	GTEX-TSE9-0326-SM-3DB82
Nerve - Tibial	Nerve	GTEX-U3ZH-1626-SM-3DB74
Nerve - Tibial	Nerve	GTEX-U8T8-0426-SM-3DB8X
Nerve - Tibial	Nerve	GTEX-U8XE-0526-SM-3DB8R
Nerve - Tibial	Nerve	GTEX-UJMC-1926-SM-3GADS
Nerve - Tibial	Nerve	GTEX-UPJH-0626-SM-4IHJN
Nerve - Tibial	Nerve	GTEX-V955-2526-SM-4JBJG
Nerve - Tibial	Nerve	GTEX-VJYA-1726-SM-3NMDQ
Nerve - Tibial	Nerve	GTEX-VUSG-2726-SM-4KKZJ
Nerve - Tibial	Nerve	GTEX-W5WG-2326-SM-3GIJH
Nerve - Tibial	Nerve	GTEX-W5X1-2826-SM-3GILM
Nerve - Tibial	Nerve	GTEX-WEY5-2226-SM-3GILQ
Nerve - Tibial	Nerve	GTEX-WFG7-2426-SM-3GIL2
Nerve - Tibial	Nerve	GTEX-WFG8-2526-SM-3GILR
Nerve - Tibial	Nerve	GTEX-WH7G-2626-SM-3NMBE
Nerve - Tibial	Nerve	GTEX-WHSB-2026-SM-3LK6H
Nerve - Tibial	Nerve	GTEX-WHSE-1026-SM-57WAX
Nerve - Tibial	Nerve	GTEX-WHWD-2326-SM-3LK6Q
Nerve - Tibial	Nerve	GTEX-WL46-0426-SM-3TW8J
Nerve - Tibial	Nerve	GTEX-WOFM-1626-SM-3MJFX
Nerve - Tibial	Nerve	GTEX-WRHU-0626-SM-3MJFI
Nerve - Tibial	Nerve	GTEX-WY7C-2626-SM-3NB2P
Nerve - Tibial	Nerve	GTEX-WYBS-0326-SM-3NM8S
Nerve - Tibial	Nerve	GTEX-WYJK-0326-SM-3NMA8
Nerve - Tibial	Nerve	GTEX-WYVS-2426-SM-3NMA9
Nerve - Tibial	Nerve	GTEX-WZTO-1026-SM-3NM9P
Nerve - Tibial	Nerve	GTEX-X15G-2526-SM-4PQZT
Nerve - Tibial	Nerve	GTEX-X3Y1-2426-SM-3P5Z7

Nerve - Tibial	Nerve	GTEX-X4EO-0126-SM-3P5YN
Nerve - Tibial	Nerve	GTEX-X4EP-0726-SM-3P5YJ
Nerve - Tibial	Nerve	GTEX-X585-0426-SM-4E3JZ
Nerve - Tibial	Nerve	GTEX-X5EB-2526-SM-4E3HY
Nerve - Tibial	Nerve	GTEX-X638-0426-SM-47JY2
Nerve - Tibial	Nerve	GTEX-XAJ8-1126-SM-47JYA
Nerve - Tibial	Nerve	GTEX-XBEC-0426-SM-4QART
Nerve - Tibial	Nerve	GTEX-XBED-2526-SM-47JYD
Nerve - Tibial	Nerve	GTEX-XBEW-0926-SM-4QASR
Nerve - Tibial	Nerve	GTEX-XGQ4-2526-SM-4AT57
Nerve - Tibial	Nerve	GTEX-XMD1-0526-SM-4AT4Z
Nerve - Tibial	Nerve	GTEX-XMD2-0326-SM-4YCD6
Nerve - Tibial	Nerve	GTEX-XMK1-2426-SM-4B66I
Nerve - Tibial	Nerve	GTEX-XOT4-0326-SM-4B66S
Nerve - Tibial	Nerve	GTEX-XPT6-2126-SM-4B66P
Nerve - Tibial	Nerve	GTEX-XPVG-2826-SM-4B66J
Nerve - Tibial	Nerve	GTEX-XQ8I-0726-SM-4BOPU
Nerve - Tibial	Nerve	GTEX-XUW1-0626-SM-4BOP4
Nerve - Tibial	Nerve	GTEX-XXEK-2526-SM-4BRUR
Nerve - Tibial	Nerve	GTEX-XYKS-2526-SM-4BOPX
Nerve - Tibial	Nerve	GTEX-Y111-0526-SM-4TT3M
Nerve - Tibial	Nerve	GTEX-Y114-2626-SM-4TT8Y
Nerve - Tibial	Nerve	GTEX-Y3I4-2426-SM-6LLJK
Nerve - Tibial	Nerve	GTEX-Y3IK-2726-SM-4WWDV
Nerve - Tibial	Nerve	GTEX-Y5LM-2226-SM-4VDT1
Nerve - Tibial	Nerve	GTEX-Y5V5-2626-SM-5IFJM
Nerve - Tibial	Nerve	GTEX-Y5V6-2726-SM-4VDSC
Nerve - Tibial	Nerve	GTEX-Y8DK-0426-SM-4TT3Q
Nerve - Tibial	Nerve	GTEX-Y8E4-0826-SM-4VBRS
Nerve - Tibial	Nerve	GTEX-Y8E5-0526-SM-57WBY
Nerve - Tibial	Nerve	GTEX-Y8LW-2126-SM-4VBRX
Nerve - Tibial	Nerve	GTEX-Y9LG-2226-SM-4VDS9
Nerve - Tibial	Nerve	GTEX-YB5E-2326-SM-5IFIJ
Nerve - Tibial	Nerve	GTEX-YB5K-2426-SM-5IFHP
Nerve - Tibial	Nerve	GTEX-YBZK-0426-SM-59HLM
Nerve - Tibial	Nerve	GTEX-YEC4-2526-SM-4W1Z2
Nerve - Tibial	Nerve	GTEX-YECK-0426-SM-4W1Z5
Nerve - Tibial	Nerve	GTEX-YF7O-2626-SM-5IFHK
Nerve - Tibial	Nerve	GTEX-YFC4-0826-SM-5CVM1
Nerve - Tibial	Nerve	GTEX-YJ8A-0726-SM-5Q5A6
Nerve - Tibial	Nerve	GTEX-YJ8O-2626-SM-5HL8H
Nerve - Tibial	Nerve	GTEX-Z93S-0826-SM-5HL7Y
Nerve - Tibial	Nerve	GTEX-Z9EW-2026-SM-5CVMQ
Nerve - Tibial	Nerve	GTEX-ZA64-2126-SM-5Q5A8
Nerve - Tibial	Nerve	GTEX-ZAB4-1826-SM-5HL8O
Nerve - Tibial	Nerve	GTEX-ZAKK-0526-SM-5EGHC
Nerve - Tibial	Nerve	GTEX-ZC5H-0526-SM-5HL9E
Nerve - Tibial	Nerve	GTEX-ZDTS-0326-SM-5HL83
Nerve - Tibial	Nerve	GTEX-ZDXO-1926-SM-5E45Q
Nerve - Tibial	Nerve	GTEX-ZDYS-1526-SM-4WKFY
Nerve - Tibial	Nerve	GTEX-ZF29-2626-SM-4WWBA
Nerve - Tibial	Nerve	GTEX-ZF2S-2526-SM-5CVNT
Nerve - Tibial	Nerve	GTEX-ZGAY-0826-SM-4WWEP
Nerve - Tibial	Nerve	GTEX-ZLV1-2026-SM-5EQMB

Nerve - Tibial	Nerve	GTEX-ZLWG-2526-SM-57WDS
Nerve - Tibial	Nerve	GTEX-ZP4G-2226-SM-57WFB
Nerve - Tibial	Nerve	GTEX-ZPCL-2426-SM-57WFP
Nerve - Tibial	Nerve	GTEX-ZQG8-1326-SM-51MQW
Nerve - Tibial	Nerve	GTEX-ZQUD-1226-SM-51MSP
Nerve - Tibial	Nerve	GTEX-ZT9X-2126-SM-57WEV
Nerve - Tibial	Nerve	GTEX-ZTPG-0526-SM-5O98W
Nerve - Tibial	Nerve	GTEX-ZTX8-1726-SM-51MSB
Nerve - Tibial	Nerve	GTEX-ZV68-0326-SM-59HJG
Nerve - Tibial	Nerve	GTEX-ZVE2-0426-SM-59HJ7
Nerve - Tibial	Nerve	GTEX-ZVT3-0326-SM-5GU6U
Nerve - Tibial	Nerve	GTEX-ZVTK-0826-SM-5GZXI
Nerve - Tibial	Nerve	GTEX-ZVZO-0526-SM-5A5LB
Nerve - Tibial	Nerve	GTEX-ZVZP-2626-SM-51MR4
Nerve - Tibial	Nerve	GTEX-ZXES-1926-SM-5E445
Nerve - Tibial	Nerve	GTEX-ZXG5-0426-SM-5GICT
Nerve - Tibial	Nerve	GTEX-ZYFC-0626-SM-5GIDR
Nerve - Tibial	Nerve	GTEX-ZYFD-0426-SM-5E45X
Nerve - Tibial	Nerve	GTEX-ZYFG-2526-SM-5GIEK
Nerve - Tibial	Nerve	GTEX-ZYT6-1426-SM-5E43U
Nerve - Tibial	Nerve	GTEX-ZYVF-0726-SM-5GICF
Nerve - Tibial	Nerve	GTEX-ZYW4-0326-SM-5E44Y
Nerve - Tibial	Nerve	GTEX-ZZ64-1426-SM-5NQ8C
Ovary	Ovary	GTEX-1117F-2226-SM-5N9CH
Ovary	Ovary	GTEX-11DXX-1426-SM-5GIDU
Ovary	Ovary	GTEX-11EM3-1726-SM-5N9D1
Ovary	Ovary	GTEX-11EMC-1726-SM-5H11P
Ovary	Ovary	GTEX-11GSP-0226-SM-5A5KV
Ovary	Ovary	GTEX-11I78-1926-SM-59878
Ovary	Ovary	GTEX-11P81-1526-SM-5P9GS
Ovary	Ovary	GTEX-11VI4-1026-SM-5EQM1
Ovary	Ovary	GTEX-11XUK-1626-SM-5GU5O
Ovary	Ovary	GTEX-11ZTS-1926-SM-5CVLA
Ovary	Ovary	GTEX-11ZTT-1826-SM-5CVLN
Ovary	Ovary	GTEX-11ZVC-1426-SM-5EGGA
Ovary	Ovary	GTEX-1269C-1826-SM-5N9E1
Ovary	Ovary	GTEX-12WSD-2726-SM-59HLR
Ovary	Ovary	GTEX-12WSG-1426-SM-5FQUK
Ovary	Ovary	GTEX-12WSJ-1226-SM-5LU91
Ovary	Ovary	GTEX-12WSK-1926-SM-5LZVK
Ovary	Ovary	GTEX-12ZZX-2026-SM-5LZV9
Ovary	Ovary	GTEX-1313W-2726-SM-5P9IX
Ovary	Ovary	GTEX-131XG-1926-SM-5LZVG
Ovary	Ovary	GTEX-131YS-2226-SM-5P9G8
Ovary	Ovary	GTEX-132AR-1426-SM-5IFF1
Ovary	Ovary	GTEX-133LE-1226-SM-5IFGC
Ovary	Ovary	GTEX-1399S-2126-SM-5J2MH
Ovary	Ovary	GTEX-1399U-1726-SM-5P9J4
Ovary	Ovary	GTEX-139D8-2426-SM-5KM3A
Ovary	Ovary	GTEX-13D11-1126-SM-5KLYS
Ovary	Ovary	GTEX-13FTX-0926-SM-5IFF7
Ovary	Ovary	GTEX-13N11-0526-SM-5IJFI
Ovary	Ovary	GTEX-13O3O-2726-SM-5KM35
Ovary	Ovary	GTEX-13OVI-0726-SM-5L3DD

Ovary	Ovary	GTEX-13OVJ-2426-SM-5KM3I
Ovary	Ovary	GTEX-13PL7-2326-SM-5L3FY
Ovary	Ovary	GTEX-13PVR-1226-SM-5RQJ2
Ovary	Ovary	GTEX-13QBU-1526-SM-5LU3W
Ovary	Ovary	GTEX-13QIC-1526-SM-5IJFL
Ovary	Ovary	GTEX-13SLX-2426-SM-664OM
Ovary	Ovary	GTEX-13U4I-2126-SM-5LU55
Ovary	Ovary	GTEX-13VXT-1526-SM-5LU3J
Ovary	Ovary	GTEX-13W3W-1526-SM-5LU5I
Ovary	Ovary	GTEX-13X6H-1026-SM-5SIBE
Ovary	Ovary	GTEX-145LS-2326-SM-5TDCY
Ovary	Ovary	GTEX-145ME-1226-SM-5SIB6
Ovary	Ovary	GTEX-145MI-2626-SM-5QGQ8
Ovary	Ovary	GTEX-146FH-2526-SM-5Q5BP
Ovary	Ovary	GTEX-14AS3-1326-SM-5RQJE
Ovary	Ovary	GTEX-14BIN-1426-SM-664NI
Ovary	Ovary	GTEX-14BMU-1726-SM-5TDE8
Ovary	Ovary	GTEX-14DAQ-2226-SM-5YYAD
Ovary	Ovary	GTEX-14JG6-0626-SM-68716
Ovary	Ovary	GTEX-14PJM-2426-SM-686ZC
Ovary	Ovary	GTEX-14PKU-1226-SM-686ZM
Ovary	Ovary	GTEX-14PKV-1226-SM-6871T
Ovary	Ovary	GTEX-14PN3-1526-SM-6ETZM
Ovary	Ovary	GTEX-15DDE-2326-SM-6AJA8
Ovary	Ovary	GTEX-15DYW-2626-SM-6LPK7
Ovary	Ovary	GTEX-15EOM-1526-SM-68723
Ovary	Ovary	GTEX-15ER7-2326-SM-7KUN3
Ovary	Ovary	GTEX-15FZZ-0826-SM-6LLJO
Ovary	Ovary	GTEX-15SDE-0926-SM-6LPIA
Ovary	Ovary	GTEX-15UF6-2026-SM-6LPI3
Ovary	Ovary	GTEX-16MT8-2226-SM-6LPK2
Ovary	Ovary	GTEX-16NGA-0426-SM-7LG6K
Ovary	Ovary	GTEX-17JCI-2326-SM-7IGPC
Ovary	Ovary	GTEX-183WM-1926-SM-72D5F
Ovary	Ovary	GTEX-18A7A-2626-SM-7LG6L
Ovary	Ovary	GTEX-18D9A-0826-SM-718C1
Ovary	Ovary	GTEX-1A32A-2726-SM-73KVU
Ovary	Ovary	GTEX-1AMEY-0926-SM-72D6G
Ovary	Ovary	GTEX-1AMFI-1426-SM-731EL
Ovary	Ovary	GTEX-1F48J-2326-SM-7MKG3
Ovary	Ovary	GTEX-1F6RS-2726-SM-7MKHA
Ovary	Ovary	GTEX-OHPK-2426-SM-3MJGH
Ovary	Ovary	GTEX-OHPL-2426-SM-48TDN
Ovary	Ovary	GTEX-P4PP-2426-SM-3P61L
Ovary	Ovary	GTEX-P4QT-2426-SM-3NMCL
Ovary	Ovary	GTEX-P78B-1926-SM-3P616
Ovary	Ovary	GTEX-PLZ4-2826-SM-3P617
Ovary	Ovary	GTEX-POMQ-1426-SM-3P61D
Ovary	Ovary	GTEX-PWCY-1326-SM-48TCU
Ovary	Ovary	GTEX-PWN1-2426-SM-48TDD
Ovary	Ovary	GTEX-PX3G-2426-SM-48TZZ
Ovary	Ovary	GTEX-Q734-0726-SM-48TZP
Ovary	Ovary	GTEX-QCQG-1426-SM-48U22
Ovary	Ovary	GTEX-QVJO-3126-SM-4R1KC

Ovary	Ovary	GTEX-R55G-1526-SM-48FER
Ovary	Ovary	GTEX-RTLS-2326-SM-46MUH
Ovary	Ovary	GTEX-RU1J-0826-SM-46MUU
Ovary	Ovary	GTEX-RU72-2226-SM-46MUE
Ovary	Ovary	GTEX-RWS6-1026-SM-47JXD
Ovary	Ovary	GTEX-S32W-1226-SM-4AD67
Ovary	Ovary	GTEX-S341-0726-SM-4AD5R
Ovary	Ovary	GTEX-S4P3-0926-SM-4AD54
Ovary	Ovary	GTEX-S7SF-1326-SM-4AD4P
Ovary	Ovary	GTEX-T2IS-2026-SM-4DM6W
Ovary	Ovary	GTEX-T5JW-0426-SM-4DM7M
Ovary	Ovary	GTEX-T6MO-1126-SM-4DM5D
Ovary	Ovary	GTEX-TML8-0926-SM-4DXSJ
Ovary	Ovary	GTEX-TMMY-1726-SM-4DXTD
Ovary	Ovary	GTEX-TSE9-2226-SM-4DXUR
Ovary	Ovary	GTEX-U3ZN-1026-SM-4DXTC
Ovary	Ovary	GTEX-UJHI-1226-SM-4IHLR
Ovary	Ovary	GTEX-W5WG-0926-SM-4RTX9
Ovary	Ovary	GTEX-WEY5-0626-SM-4LMIA
Ovary	Ovary	GTEX-WI4N-2526-SM-4OOSE
Ovary	Ovary	GTEX-WWYW-2726-SM-4MVOP
Ovary	Ovary	GTEX-WXYG-1426-SM-4ONCK
Ovary	Ovary	GTEX-WYBS-2426-SM-4ONDI
Ovary	Ovary	GTEX-WYVS-1526-SM-4OND4
Ovary	Ovary	GTEX-X15G-1726-SM-4PQZN
Ovary	Ovary	GTEX-X4EO-2426-SM-4QASD
Ovary	Ovary	GTEX-X8HC-2726-SM-46MUA
Ovary	Ovary	GTEX-XMD3-2326-SM-4AT5H
Ovary	Ovary	GTEX-XUW1-0126-SM-4BOOQ
Ovary	Ovary	GTEX-XUZC-1026-SM-4BOPY
Ovary	Ovary	GTEX-XV7Q-1426-SM-4BRWA
Ovary	Ovary	GTEX-XYKS-1726-SM-4E3IO
Ovary	Ovary	GTEX-Y114-1726-SM-4TT7U
Ovary	Ovary	GTEX-Y3IK-2026-SM-4YCDG
Ovary	Ovary	GTEX-YB5K-1426-SM-5IFIB
Ovary	Ovary	GTEX-YFC4-2726-SM-5IFJW
Ovary	Ovary	GTEX-YJ8O-1026-SM-5CVM5
Ovary	Ovary	GTEX-ZAJG-2726-SM-5S2MU
Ovary	Ovary	GTEX-ZC5H-2026-SM-5CVN2
Ovary	Ovary	GTEX-ZLWG-0926-SM-4WWC3
Ovary	Ovary	GTEX-ZP4G-0626-SM-4WWF1
Ovary	Ovary	GTEX-ZPIC-1426-SM-4WWCP
Ovary	Ovary	GTEX-ZV6S-1426-SM-59HKX
Ovary	Ovary	GTEX-ZVT2-0326-SM-5E44G
Ovary	Ovary	GTEX-ZVT3-2626-SM-5GU5L
Ovary	Ovary	GTEX-ZYFG-1726-SM-5GZZB
Ovary	Ovary	GTEX-ZYY3-2726-SM-5EGH4
Ovary	Ovary	GTEX-ZZPU-2126-SM-5EGIU
Pancreas	Pancreas	GTEX-111CU-0526-SM-5EGHK
Pancreas	Pancreas	GTEX-111YS-1226-SM-5EGGJ
Pancreas	Pancreas	GTEX-1122O-0726-SM-5GIEV
Pancreas	Pancreas	GTEX-1128S-0826-SM-5GZZI
Pancreas	Pancreas	GTEX-117YX-0226-SM-5EGH6
Pancreas	Pancreas	GTEX-11DXX-0926-SM-5H112

Pancreas	Pancreas	GTEX-11EQ9-1026-SM-5H134
Pancreas	Pancreas	GTEX-11GSP-0426-SM-5A5KX
Pancreas	Pancreas	GTEX-11I78-0626-SM-5A5LZ
Pancreas	Pancreas	GTEX-11LCK-0226-SM-5A5M6
Pancreas	Pancreas	GTEX-11NSD-0526-SM-5A5LT
Pancreas	Pancreas	GTEX-11ONC-0526-SM-5BC57
Pancreas	Pancreas	GTEX-11P7K-0526-SM-5BC5I
Pancreas	Pancreas	GTEX-11TT1-0326-SM-5LUAY
Pancreas	Pancreas	GTEX-11VI4-0426-SM-5EGHZ
Pancreas	Pancreas	GTEX-11XUK-0626-SM-5N9ES
Pancreas	Pancreas	GTEX-1211K-1126-SM-5EGGB
Pancreas	Pancreas	GTEX-12BJ1-1126-SM-5EGGG
Pancreas	Pancreas	GTEX-12WSD-1626-SM-5GCNR
Pancreas	Pancreas	GTEX-12WSG-1026-SM-5EGII
Pancreas	Pancreas	GTEX-12WSK-0226-SM-5BC62
Pancreas	Pancreas	GTEX-12WSL-0426-SM-5GCNX
Pancreas	Pancreas	GTEX-12WSN-0826-SM-5GCOF
Pancreas	Pancreas	GTEX-131XE-1926-SM-5IFER
Pancreas	Pancreas	GTEX-131XF-1426-SM-5BC68
Pancreas	Pancreas	GTEX-131XG-1426-SM-5GCMO
Pancreas	Pancreas	GTEX-132AR-1826-SM-5EGHR
Pancreas	Pancreas	GTEX-132QS-0926-SM-5P9GB
Pancreas	Pancreas	GTEX-1339X-1026-SM-5IFH5
Pancreas	Pancreas	GTEX-1399R-0426-SM-5IJE3
Pancreas	Pancreas	GTEX-1399S-0326-SM-5IFFS
Pancreas	Pancreas	GTEX-1399U-0926-SM-5IFHA
Pancreas	Pancreas	GTEX-139YR-1526-SM-5IFJ1
Pancreas	Pancreas	GTEX-13CF3-1026-SM-5LZWY
Pancreas	Pancreas	GTEX-13D11-2226-SM-5IFEO
Pancreas	Pancreas	GTEX-13FH7-1426-SM-5IFIC
Pancreas	Pancreas	GTEX-13FLV-0626-SM-5IFEY
Pancreas	Pancreas	GTEX-13FTW-0526-SM-5IFIP
Pancreas	Pancreas	GTEX-13FTX-1226-SM-5IFGN
Pancreas	Pancreas	GTEX-13N11-0226-SM-5KM3C
Pancreas	Pancreas	GTEX-13O1R-1826-SM-5KM3B
Pancreas	Pancreas	GTEX-13O61-2126-SM-5IJEO
Pancreas	Pancreas	GTEX-13OVI-0526-SM-5IFFQ
Pancreas	Pancreas	GTEX-13PL7-2226-SM-5L3IC
Pancreas	Pancreas	GTEX-13PVR-0726-SM-5S2PX
Pancreas	Pancreas	GTEX-13SLX-1326-SM-5S2QS
Pancreas	Pancreas	GTEX-13U4I-1526-SM-5IFFF
Pancreas	Pancreas	GTEX-13VXT-1226-SM-5LU3M
Pancreas	Pancreas	GTEX-13W3W-1026-SM-5IFG4
Pancreas	Pancreas	GTEX-13X6H-0626-SM-5LU53
Pancreas	Pancreas	GTEX-13YAN-2126-SM-5Q5C4
Pancreas	Pancreas	GTEX-144FL-1526-SM-5Q5CA
Pancreas	Pancreas	GTEX-144GM-0726-SM-79OJQ
Pancreas	Pancreas	GTEX-144GN-0826-SM-5LU5G
Pancreas	Pancreas	GTEX-145ME-0326-SM-5S2QO
Pancreas	Pancreas	GTEX-145MN-1426-SM-5SI9H
Pancreas	Pancreas	GTEX-145MO-2126-SM-5Q5CZ
Pancreas	Pancreas	GTEX-146FQ-1526-SM-5Q5CX
Pancreas	Pancreas	GTEX-146FR-0126-SM-5Q5F3
Pancreas	Pancreas	GTEX-148VI-0826-SM-5TDDI

Pancreas	Pancreas	GTEX-1497J-0426-SM-5Q5CO
Pancreas	Pancreas	GTEX-14AS3-0326-SM-5Q5DB
Pancreas	Pancreas	GTEX-14BMU-0726-SM-73KXS
Pancreas	Pancreas	GTEX-14C39-1026-SM-73KWG
Pancreas	Pancreas	GTEX-14DAR-0526-SM-664MX
Pancreas	Pancreas	GTEX-14E6E-1426-SM-5RQI2
Pancreas	Pancreas	GTEX-14ICL-1126-SM-5S2RE
Pancreas	Pancreas	GTEX-14JG6-0426-SM-68715
Pancreas	Pancreas	GTEX-14PHX-2026-SM-6872C
Pancreas	Pancreas	GTEX-14PHY-1126-SM-664OR
Pancreas	Pancreas	GTEX-14PJ2-1526-SM-686Z4
Pancreas	Pancreas	GTEX-14PJ3-0426-SM-6LLHF
Pancreas	Pancreas	GTEX-14PJ4-0726-SM-6872E
Pancreas	Pancreas	GTEX-14PJ6-0926-SM-686ZA
Pancreas	Pancreas	GTEX-14PJM-2126-SM-6AJAS
Pancreas	Pancreas	GTEX-14PJN-1526-SM-6AJBP
Pancreas	Pancreas	GTEX-14PJO-1826-SM-69LPR
Pancreas	Pancreas	GTEX-14PK6-1026-SM-664NZ
Pancreas	Pancreas	GTEX-14PKU-0626-SM-6EU1Q
Pancreas	Pancreas	GTEX-14PKV-0926-SM-6AJB4
Pancreas	Pancreas	GTEX-14PN3-0126-SM-6AJAU
Pancreas	Pancreas	GTEX-14PN4-1626-SM-6AJB5
Pancreas	Pancreas	GTEX-15DYW-1626-SM-6LLI1
Pancreas	Pancreas	GTEX-15ER7-1326-SM-6LPK9
Pancreas	Pancreas	GTEX-15EU6-1126-SM-68721
Pancreas	Pancreas	GTEX-15FZZ-0626-SM-7KFTO
Pancreas	Pancreas	GTEX-15RIF-0626-SM-6LLI3
Pancreas	Pancreas	GTEX-15RJ7-0826-SM-6LLI8
Pancreas	Pancreas	GTEX-15SDE-0826-SM-6M463
Pancreas	Pancreas	GTEX-15SHV-1526-SM-6LLIB
Pancreas	Pancreas	GTEX-15UF6-1826-SM-6LLI9
Pancreas	Pancreas	GTEX-15UF7-0826-SM-6M46E
Pancreas	Pancreas	GTEX-15UKP-1326-SM-6LPI8
Pancreas	Pancreas	GTEX-16MT8-1726-SM-7EWE7
Pancreas	Pancreas	GTEX-178AV-0226-SM-793B7
Pancreas	Pancreas	GTEX-17F98-0426-SM-79ONZ
Pancreas	Pancreas	GTEX-17F9E-1426-SM-793BG
Pancreas	Pancreas	GTEX-17HGU-2026-SM-79OKS
Pancreas	Pancreas	GTEX-17HHE-0526-SM-7DUGR
Pancreas	Pancreas	GTEX-17KNJ-1926-SM-7KFRV
Pancreas	Pancreas	GTEX-17MF6-1426-SM-7IGOX
Pancreas	Pancreas	GTEX-18A66-2126-SM-7189D
Pancreas	Pancreas	GTEX-18A67-1726-SM-7KFT9
Pancreas	Pancreas	GTEX-18A6Q-1726-SM-7LT9A
Pancreas	Pancreas	GTEX-18A7A-1726-SM-7LT93
Pancreas	Pancreas	GTEX-18D9U-1626-SM-7KFTW
Pancreas	Pancreas	GTEX-1A3MW-1926-SM-7IGM8
Pancreas	Pancreas	GTEX-1AMFI-0726-SM-731D9
Pancreas	Pancreas	GTEX-1AX8Z-1726-SM-731DE
Pancreas	Pancreas	GTEX-1AX9J-1726-SM-731FF
Pancreas	Pancreas	GTEX-1AX9K-2026-SM-731D1
Pancreas	Pancreas	GTEX-1AYCT-1226-SM-7EPGH
Pancreas	Pancreas	GTEX-1AYD5-0326-SM-7EWEO
Pancreas	Pancreas	GTEX-1B8KE-0726-SM-72D7G

Pancreas	Pancreas	GTEX-1B8KZ-0926-SM-731D7
Pancreas	Pancreas	GTEX-1B932-1626-SM-731EA
Pancreas	Pancreas	GTEX-1B933-1726-SM-731FC
Pancreas	Pancreas	GTEX-1B97J-1526-SM-79ONI
Pancreas	Pancreas	GTEX-1B996-1326-SM-731EO
Pancreas	Pancreas	GTEX-1C2JI-0926-SM-731F1
Pancreas	Pancreas	GTEX-1C4CL-0926-SM-79OL2
Pancreas	Pancreas	GTEX-1C64O-1426-SM-7EWER
Pancreas	Pancreas	GTEX-1C6VQ-1726-SM-7IGLQ
Pancreas	Pancreas	GTEX-1CAMS-1926-SM-7DUEU
Pancreas	Pancreas	GTEX-1CB4F-1226-SM-7DHKU
Pancreas	Pancreas	GTEX-1CB4I-1326-SM-79OLY
Pancreas	Pancreas	GTEX-1F5PL-0726-SM-7MKH8
Pancreas	Pancreas	GTEX-NFK9-1626-SM-3LK5J
Pancreas	Pancreas	GTEX-NPJ8-2126-SM-3MJGK
Pancreas	Pancreas	GTEX-O5YT-1026-SM-3MJGF
Pancreas	Pancreas	GTEX-OHPL-1026-SM-3MJGI
Pancreas	Pancreas	GTEX-OHPM-1026-SM-3LK74
Pancreas	Pancreas	GTEX-OOBJ-1026-SM-3NB2L
Pancreas	Pancreas	GTEX-OOBK-1026-SM-48TC2
Pancreas	Pancreas	GTEX-P4PP-1026-SM-3NM9O
Pancreas	Pancreas	GTEX-P4PQ-1026-SM-3NMCN
Pancreas	Pancreas	GTEX-P4QS-1026-SM-3NMCW
Pancreas	Pancreas	GTEX-PLZ6-0726-SM-3P619
Pancreas	Pancreas	GTEX-PSDG-1526-SM-48TCY
Pancreas	Pancreas	GTEX-PW2O-0826-SM-48TC5
Pancreas	Pancreas	GTEX-PX3G-1026-SM-48TZW
Pancreas	Pancreas	GTEX-Q2AH-0926-SM-48TZK
Pancreas	Pancreas	GTEX-Q2AI-0426-SM-48U13
Pancreas	Pancreas	GTEX-Q734-0426-SM-48TZX
Pancreas	Pancreas	GTEX-QCQG-0426-SM-48U29
Pancreas	Pancreas	GTEX-QDVJ-1226-SM-48U1V
Pancreas	Pancreas	GTEX-QDVN-0926-SM-2I5GL
Pancreas	Pancreas	GTEX-QEL4-1326-SM-447AD
Pancreas	Pancreas	GTEX-QESD-0226-SM-447BH
Pancreas	Pancreas	GTEX-QLQ7-0626-SM-4R1JT
Pancreas	Pancreas	GTEX-QLQW-0326-SM-447A8
Pancreas	Pancreas	GTEX-QMRM-0326-SM-4R1K5
Pancreas	Pancreas	GTEX-QV31-0226-SM-447BO
Pancreas	Pancreas	GTEX-QV44-0426-SM-4R1KF
Pancreas	Pancreas	GTEX-R53T-0426-SM-48FEM
Pancreas	Pancreas	GTEX-R55D-1426-SM-48FEN
Pancreas	Pancreas	GTEX-R55G-0326-SM-48FDM
Pancreas	Pancreas	GTEX-RM2N-0326-SM-48FD8
Pancreas	Pancreas	GTEX-RWS6-1126-SM-47JXC
Pancreas	Pancreas	GTEX-S32W-0826-SM-4AD5Z
Pancreas	Pancreas	GTEX-S33H-1226-SM-4AD69
Pancreas	Pancreas	GTEX-S3XE-0526-SM-4AD4G
Pancreas	Pancreas	GTEX-S4P3-0626-SM-4AD59
Pancreas	Pancreas	GTEX-S95S-0726-SM-4B64H
Pancreas	Pancreas	GTEX-SE5C-0326-SM-4BRWX
Pancreas	Pancreas	GTEX-SNOS-0926-SM-4DM7A
Pancreas	Pancreas	GTEX-T5JW-0226-SM-4DM7I
Pancreas	Pancreas	GTEX-T8EM-0826-SM-4DM76

Pancreas	Pancreas	GTEX-TKQ2-0426-SM-4DXUO
Pancreas	Pancreas	GTEX-TML8-0526-SM-32QOQ
Pancreas	Pancreas	GTEX-TMMY-1326-SM-4DXU9
Pancreas	Pancreas	GTEX-U4B1-0726-SM-4DXUA
Pancreas	Pancreas	GTEX-U8XE-2026-SM-3DB8S
Pancreas	Pancreas	GTEX-UJHI-0626-SM-3DB8T
Pancreas	Pancreas	GTEX-UJMC-1126-SM-3GADP
Pancreas	Pancreas	GTEX-V1D1-0726-SM-4JBH7
Pancreas	Pancreas	GTEX-V955-0326-SM-4JBGV
Pancreas	Pancreas	GTEX-VJYA-0826-SM-4KL1M
Pancreas	Pancreas	GTEX-VUSG-0326-SM-3GIJ7
Pancreas	Pancreas	GTEX-W5WG-0826-SM-4RGNE
Pancreas	Pancreas	GTEX-WFG7-0426-SM-4LMK5
Pancreas	Pancreas	GTEX-WFG8-0326-SM-4LVN4
Pancreas	Pancreas	GTEX-WFJO-0626-SM-4LVMC
Pancreas	Pancreas	GTEX-WFON-0626-SM-4LVLX
Pancreas	Pancreas	GTEX-WH7G-0826-SM-4LVMR
Pancreas	Pancreas	GTEX-WHPG-0326-SM-4M1XV
Pancreas	Pancreas	GTEX-WHSB-0726-SM-4M1XQ
Pancreas	Pancreas	GTEX-WHWD-0726-SM-4OORX
Pancreas	Pancreas	GTEX-WI4N-1826-SM-4OOSF
Pancreas	Pancreas	GTEX-WQUQ-2126-SM-4OOSO
Pancreas	Pancreas	GTEX-WRHK-0226-SM-4MVOH
Pancreas	Pancreas	GTEX-WXYG-0826-SM-4ONC7
Pancreas	Pancreas	GTEX-WY7C-1026-SM-4OND3
Pancreas	Pancreas	GTEX-WYJK-2426-SM-4ONDQ
Pancreas	Pancreas	GTEX-WYVS-0926-SM-4SOJV
Pancreas	Pancreas	GTEX-X15G-0726-SM-4PQZ5
Pancreas	Pancreas	GTEX-X3Y1-0726-SM-3P5YU
Pancreas	Pancreas	GTEX-X4LF-0326-SM-4QAS9
Pancreas	Pancreas	GTEX-X5EB-0526-SM-46MVP
Pancreas	Pancreas	GTEX-XBED-0226-SM-47JY8
Pancreas	Pancreas	GTEX-XBEW-1626-SM-4SOJP
Pancreas	Pancreas	GTEX-XGQ4-0226-SM-4GIDS
Pancreas	Pancreas	GTEX-XMK1-0326-SM-4B652
Pancreas	Pancreas	GTEX-XPVG-0326-SM-4B653
Pancreas	Pancreas	GTEX-XQ8I-1926-SM-4BOOK
Pancreas	Pancreas	GTEX-XUW1-1726-SM-4BOOZ
Pancreas	Pancreas	GTEX-XV7Q-0926-SM-4BRVQ
Pancreas	Pancreas	GTEX-XXEK-1726-SM-4BRVB
Pancreas	Pancreas	GTEX-XYKS-1226-SM-4BRVI
Pancreas	Pancreas	GTEX-Y114-0826-SM-4TT77
Pancreas	Pancreas	GTEX-Y3I4-0826-SM-4TT2A
Pancreas	Pancreas	GTEX-Y3IK-0426-SM-4WWE2
Pancreas	Pancreas	GTEX-Y5LM-0526-SM-4V6G3
Pancreas	Pancreas	GTEX-Y5V5-1026-SM-5LUAH
Pancreas	Pancreas	GTEX-Y8E4-1326-SM-5IFIY
Pancreas	Pancreas	GTEX-Y8LW-1026-SM-5IFJY
Pancreas	Pancreas	GTEX-Y9LG-0726-SM-4VDS3
Pancreas	Pancreas	GTEX-YB5E-0526-SM-4VDSD
Pancreas	Pancreas	GTEX-YB5K-1826-SM-5IFJC
Pancreas	Pancreas	GTEX-YEC3-0626-SM-5IFHZ
Pancreas	Pancreas	GTEX-YEC4-1326-SM-5IFHG
Pancreas	Pancreas	GTEX-YF7O-1326-SM-4W1ZR

Pancreas	Pancreas	GTEX-YFCO-1026-SM-5LU9S
Pancreas	Pancreas	GTEX-ZAB4-1726-SM-5HL8C
Pancreas	Pancreas	GTEX-ZAB5-0826-SM-5P9FU
Pancreas	Pancreas	GTEX-ZAK1-2326-SM-5CVMY
Pancreas	Pancreas	GTEX-ZC5H-0826-SM-5N9FH
Pancreas	Pancreas	GTEX-ZDTT-1126-SM-4WKFW
Pancreas	Pancreas	GTEX-ZDYS-2526-SM-4WKGU
Pancreas	Pancreas	GTEX-ZEX8-1026-SM-4WKHE
Pancreas	Pancreas	GTEX-ZF29-1126-SM-4WKGO
Pancreas	Pancreas	GTEX-ZF3C-2026-SM-4WWB5
Pancreas	Pancreas	GTEX-ZG7Y-0326-SM-4WWEY
Pancreas	Pancreas	GTEX-ZLFU-0726-SM-57WF6
Pancreas	Pancreas	GTEX-ZLWG-0326-SM-4WWC7
Pancreas	Pancreas	GTEX-ZP4G-0426-SM-4YCER
Pancreas	Pancreas	GTEX-ZPIC-0926-SM-4WWFK
Pancreas	Pancreas	GTEX-ZPU1-0226-SM-4WWC9
Pancreas	Pancreas	GTEX-ZT9W-0926-SM-57WFS
Pancreas	Pancreas	GTEX-ZTPG-1026-SM-5DUWP
Pancreas	Pancreas	GTEX-ZV7C-0726-SM-59HKH
Pancreas	Pancreas	GTEX-ZVP2-0726-SM-59HKY
Pancreas	Pancreas	GTEX-ZVT2-2026-SM-5NQ8Q
Pancreas	Pancreas	GTEX-ZVZP-0626-SM-59HL5
Pancreas	Pancreas	GTEX-ZYFG-0826-SM-5BC5T
Pancreas	Pancreas	GTEX-ZYW4-2126-SM-59HJ9
Pancreas	Pancreas	GTEX-ZYY3-0826-SM-5E44R
Pancreas	Pancreas	GTEX-ZZPU-0726-SM-5N9C8
Pituitary	Pituitary	GTEX-1128S-2626-SM-5H11Z
Pituitary	Pituitary	GTEX-113JC-2826-SM-5EGIT
Pituitary	Pituitary	GTEX-117XS-2926-SM-5N9BX
Pituitary	Pituitary	GTEX-117YW-2826-SM-5H116
Pituitary	Pituitary	GTEX-1192W-3026-SM-5GZZV
Pituitary	Pituitary	GTEX-1192X-3026-SM-5N9BM
Pituitary	Pituitary	GTEX-11DXW-1226-SM-5H133
Pituitary	Pituitary	GTEX-11DXY-3026-SM-5N9CB
Pituitary	Pituitary	GTEX-11DYG-3126-SM-5A5L6
Pituitary	Pituitary	GTEX-11EI6-3126-SM-5H111
Pituitary	Pituitary	GTEX-11EMC-3126-SM-5EGJP
Pituitary	Pituitary	GTEX-11GS4-3026-SM-5A5LG
Pituitary	Pituitary	GTEX-11GSO-2826-SM-5HL6Q
Pituitary	Pituitary	GTEX-11GSP-3026-SM-5N9CE
Pituitary	Pituitary	GTEX-11NUK-3126-SM-5986M
Pituitary	Pituitary	GTEX-11NV4-2326-SM-5BC4X
Pituitary	Pituitary	GTEX-11O72-3026-SM-5986R
Pituitary	Pituitary	GTEX-11OF3-3026-SM-5GU52
Pituitary	Pituitary	GTEX-11ONC-2826-SM-5P9GQ
Pituitary	Pituitary	GTEX-11TUW-3026-SM-5EGKM
Pituitary	Pituitary	GTEX-11WQK-3226-SM-5EGI3
Pituitary	Pituitary	GTEX-11ZTS-3326-SM-5LU9Y
Pituitary	Pituitary	GTEX-11ZUS-3026-SM-5EGI4
Pituitary	Pituitary	GTEX-11ZVC-3226-SM-5FQV1
Pituitary	Pituitary	GTEX-12126-1126-SM-5P9GP
Pituitary	Pituitary	GTEX-1212Z-2926-SM-59HKV
Pituitary	Pituitary	GTEX-12584-3126-SM-5EGKR
Pituitary	Pituitary	GTEX-12696-3126-SM-5FQUX

Pituitary	Pituitary	GTEX-1269C-3226-SM-5EGKT
Pituitary	Pituitary	GTEX-12WSC-3126-SM-5GCNB
Pituitary	Pituitary	GTEX-12WSD-3226-SM-5HL9G
Pituitary	Pituitary	GTEX-12WSE-3126-SM-5YY9F
Pituitary	Pituitary	GTEX-12WSF-3026-SM-6LPIU
Pituitary	Pituitary	GTEX-12WSH-2926-SM-5GCMR
Pituitary	Pituitary	GTEX-12WSM-2226-SM-5DUVS
Pituitary	Pituitary	GTEX-12ZZW-3026-SM-5LZW7
Pituitary	Pituitary	GTEX-12ZZX-3026-SM-5GCP3
Pituitary	Pituitary	GTEX-12ZZY-3126-SM-5DUX3
Pituitary	Pituitary	GTEX-13112-3126-SM-5IFGP
Pituitary	Pituitary	GTEX-1313W-3226-SM-5LZUU
Pituitary	Pituitary	GTEX-131XW-3226-SM-5LZUO
Pituitary	Pituitary	GTEX-131YS-3226-SM-5IFGV
Pituitary	Pituitary	GTEX-1399T-2926-SM-5IFF8
Pituitary	Pituitary	GTEX-139T4-0826-SM-5N9GF
Pituitary	Pituitary	GTEX-139TS-2926-SM-5KM1Z
Pituitary	Pituitary	GTEX-139TT-2726-SM-5IJFM
Pituitary	Pituitary	GTEX-13CF2-3226-SM-5IFF6
Pituitary	Pituitary	GTEX-13FHO-3126-SM-5IJF6
Pituitary	Pituitary	GTEX-13FHP-3126-SM-5KLYX
Pituitary	Pituitary	GTEX-13FTY-2826-SM-5J2ML
Pituitary	Pituitary	GTEX-13G51-3226-SM-5IFG7
Pituitary	Pituitary	GTEX-13IVO-3026-SM-5J2M2
Pituitary	Pituitary	GTEX-13JUV-2626-SM-5N9EB
Pituitary	Pituitary	GTEX-13JVG-3026-SM-5IJEV
Pituitary	Pituitary	GTEX-13N1W-3126-SM-5IFHB
Pituitary	Pituitary	GTEX-13N2G-2926-SM-5IJEE
Pituitary	Pituitary	GTEX-13NYS-2926-SM-5IFGH
Pituitary	Pituitary	GTEX-13NZA-3126-SM-5IJFU
Pituitary	Pituitary	GTEX-13O1R-3226-SM-5KM58
Pituitary	Pituitary	GTEX-13OVH-3126-SM-5MR4Z
Pituitary	Pituitary	GTEX-13OVJ-2626-SM-5IJFT
Pituitary	Pituitary	GTEX-13OVL-3226-SM-5L3H8
Pituitary	Pituitary	GTEX-13OW5-2926-SM-5IJF2
Pituitary	Pituitary	GTEX-13OW6-3126-SM-5L3IA
Pituitary	Pituitary	GTEX-13OW7-3126-SM-5L3HB
Pituitary	Pituitary	GTEX-13OW8-3026-SM-5L3G8
Pituitary	Pituitary	GTEX-13PLJ-1726-SM-5L3FT
Pituitary	Pituitary	GTEX-13QJC-3326-SM-664OS
Pituitary	Pituitary	GTEX-13RTJ-2926-SM-5Q5DE
Pituitary	Pituitary	GTEX-13SLW-2926-SM-5Q5BQ
Pituitary	Pituitary	GTEX-13SLX-3026-SM-5RQJU
Pituitary	Pituitary	GTEX-13VXU-3126-SM-5SIA4
Pituitary	Pituitary	GTEX-13X6J-3126-SM-5Q5EJ
Pituitary	Pituitary	GTEX-13X6K-3026-SM-5QGPA
Pituitary	Pituitary	GTEX-1445S-3226-SM-5TDE1
Pituitary	Pituitary	GTEX-144GL-3126-SM-5TDDM
Pituitary	Pituitary	GTEX-145LS-3226-SM-5TDCA
Pituitary	Pituitary	GTEX-145LU-2826-SM-5TDD1
Pituitary	Pituitary	GTEX-145MF-2626-SM-5O98S
Pituitary	Pituitary	GTEX-145MH-3126-SM-5S2QT
Pituitary	Pituitary	GTEX-145MO-3126-SM-5S2RT
Pituitary	Pituitary	GTEX-14753-2926-SM-5LU9J

Pituitary	Pituitary	GTEX-1477Z-2826-SM-5SI9J
Pituitary	Pituitary	GTEX-147F4-3126-SM-5TDDF
Pituitary	Pituitary	GTEX-147GR-2926-SM-5RQIN
Pituitary	Pituitary	GTEX-148VJ-2926-SM-5Q5DA
Pituitary	Pituitary	GTEX-14A5H-3026-SM-5RQHV
Pituitary	Pituitary	GTEX-14A5I-3026-SM-5TDEC
Pituitary	Pituitary	GTEX-14ABY-3126-SM-664NF
Pituitary	Pituitary	GTEX-14BIL-2926-SM-5SIAF
Pituitary	Pituitary	GTEX-14BIM-3226-SM-5RQIQ
Pituitary	Pituitary	GTEX-14BIN-3126-SM-664NJ
Pituitary	Pituitary	GTEX-14BMV-3126-SM-5S2UV
Pituitary	Pituitary	GTEX-14C39-2926-SM-73KU3
Pituitary	Pituitary	GTEX-14C5O-3126-SM-664NK
Pituitary	Pituitary	GTEX-14DAQ-3226-SM-664OI
Pituitary	Pituitary	GTEX-14E6C-3126-SM-664ON
Pituitary	Pituitary	GTEX-14E7W-3126-SM-68725
Pituitary	Pituitary	GTEX-14JG1-3126-SM-69LOQ
Pituitary	Pituitary	GTEX-14LLW-3026-SM-6EU1O
Pituitary	Pituitary	GTEX-14LZ3-3126-SM-69LPN
Pituitary	Pituitary	GTEX-14PJM-3226-SM-6AJA1
Pituitary	Pituitary	GTEX-14PN4-3226-SM-6AJBO
Pituitary	Pituitary	GTEX-14PQA-3126-SM-6LPKD
Pituitary	Pituitary	GTEX-15CHQ-3126-SM-6872B
Pituitary	Pituitary	GTEX-15D79-3126-SM-6M48C
Pituitary	Pituitary	GTEX-15DCD-3026-SM-6PALL
Pituitary	Pituitary	GTEX-15DDE-3226-SM-6LPK6
Pituitary	Pituitary	GTEX-15EO6-3126-SM-6LPIQ
Pituitary	Pituitary	GTEX-15ER7-3226-SM-6LPIX
Pituitary	Pituitary	GTEX-15G19-3026-SM-6LPJP
Pituitary	Pituitary	GTEX-15UF6-3126-SM-6M46J
Pituitary	Pituitary	GTEX-16GPK-3126-SM-79OOB
Pituitary	Pituitary	GTEX-16XZZ-3126-SM-7EPGJ
Pituitary	Pituitary	GTEX-16YQH-3126-SM-79OJZ
Pituitary	Pituitary	GTEX-16Z82-3126-SM-7EWEC
Pituitary	Pituitary	GTEX-17EVP-3126-SM-79OKW
Pituitary	Pituitary	GTEX-17EVQ-2926-SM-7EWE3
Pituitary	Pituitary	GTEX-17HG3-3026-SM-79ONC
Pituitary	Pituitary	GTEX-17HHY-3126-SM-7KFSG
Pituitary	Pituitary	GTEX-17HII-3126-SM-7KFRX
Pituitary	Pituitary	GTEX-18465-3126-SM-72D75
Pituitary	Pituitary	GTEX-18A6Q-3026-SM-72D6T
Pituitary	Pituitary	GTEX-1A32A-3226-SM-731FA
Pituitary	Pituitary	GTEX-1A3MW-3226-SM-731E1
Pituitary	Pituitary	GTEX-1A3MX-3126-SM-731FR
Pituitary	Pituitary	GTEX-1A8FM-3126-SM-7MKGF
Pituitary	Pituitary	GTEX-1A8G6-3026-SM-7EWEJ
Pituitary	Pituitary	GTEX-1A8G7-2926-SM-79ONA
Pituitary	Pituitary	GTEX-1B8L1-3126-SM-7EPHA
Pituitary	Pituitary	GTEX-1B8SG-1826-SM-731F3
Pituitary	Pituitary	GTEX-1B933-2926-SM-731FO
Pituitary	Pituitary	GTEX-1B996-3026-SM-7MKG8
Pituitary	Pituitary	GTEX-1BAJH-2926-SM-7EPHP
Pituitary	Pituitary	GTEX-1C6VQ-3126-SM-7MKGH
Pituitary	Pituitary	GTEX-1C6VS-2926-SM-7EWEV

Pituitary	Pituitary	GTEX-1C6WA-2926-SM-79OLF
Pituitary	Pituitary	GTEX-1CB4G-3126-SM-7EPHX
Pituitary	Pituitary	GTEX-1CB4J-3126-SM-7MKFS
Pituitary	Pituitary	GTEX-1EH9U-3226-SM-7MKGR
Pituitary	Pituitary	GTEX-1EKGG-3026-SM-7MKFU
Pituitary	Pituitary	GTEX-1EMGI-2926-SM-7DUEJ
Pituitary	Pituitary	GTEX-1F48J-2926-SM-7MKG4
Pituitary	Pituitary	GTEX-1F6I4-3226-SM-7MKGI
Pituitary	Pituitary	GTEX-1F6IF-2926-SM-7MKGB
Pituitary	Pituitary	GTEX-1GF9V-3126-SM-7MKHH
Pituitary	Pituitary	GTEX-1GF9W-2826-SM-7MKGE
Pituitary	Pituitary	GTEX-1GN1U-3226-SM-7MKH5
Pituitary	Pituitary	GTEX-N7MS-2625-SM-3LK77
Pituitary	Pituitary	GTEX-N7MT-1026-SM-3TW8T
Pituitary	Pituitary	GTEX-NPJ7-2926-SM-3MJGQ
Pituitary	Pituitary	GTEX-NPJ8-1426-SM-3MJHR
Pituitary	Pituitary	GTEX-OXRN-2626-SM-48TBX
Pituitary	Pituitary	GTEX-PVOW-2726-SM-48TCA
Pituitary	Pituitary	GTEX-Q2AG-3026-SM-48U1L
Pituitary	Pituitary	GTEX-QVJO-1226-SM-4R1KA
Pituitary	Pituitary	GTEX-R55E-2726-SM-48FCX
Pituitary	Pituitary	GTEX-RNOR-2426-SM-48FDY
Pituitary	Pituitary	GTEX-RU72-3126-SM-46MUB
Pituitary	Pituitary	GTEX-T2IS-3126-SM-32QPK
Pituitary	Pituitary	GTEX-T5JC-2526-SM-4DM6G
Pituitary	Pituitary	GTEX-T6MN-2726-SM-4DM77
Pituitary	Pituitary	GTEX-TSE9-3126-SM-4DXSY
Pituitary	Pituitary	GTEX-UTHO-3126-SM-3P5ZB
Pituitary	Pituitary	GTEX-WHSE-3126-SM-3P5ZI
Pituitary	Pituitary	GTEX-WL46-3026-SM-3LK7Z
Pituitary	Pituitary	GTEX-WVLH-3126-SM-3MJGA
Pituitary	Pituitary	GTEX-WWYW-3226-SM-3NB3B
Pituitary	Pituitary	GTEX-WZTO-3026-SM-3NMA2
Pituitary	Pituitary	GTEX-X261-3226-SM-3NMC3
Pituitary	Pituitary	GTEX-X4EP-3226-SM-3P5YR
Pituitary	Pituitary	GTEX-X585-3126-SM-4QASH
Pituitary	Pituitary	GTEX-XLM4-3126-SM-4AT6M
Pituitary	Pituitary	GTEX-YFC4-3226-SM-5CVM2
Pituitary	Pituitary	GTEX-Z93S-3026-SM-5CVMS
Pituitary	Pituitary	GTEX-ZAB4-3126-SM-57WCJ
Pituitary	Pituitary	GTEX-ZAJG-3226-SM-5HL9V
Pituitary	Pituitary	GTEX-ZAK1-3126-SM-5S2O8
Pituitary	Pituitary	GTEX-ZDXO-1426-SM-4WKHG
Pituitary	Pituitary	GTEX-ZE9C-3126-SM-4WKHN
Pituitary	Pituitary	GTEX-ZF28-3126-SM-4WKFC
Pituitary	Pituitary	GTEX-ZUA1-3126-SM-59HJ8
Pituitary	Pituitary	GTEX-ZYY3-3226-SM-5SI9X
Prostate	Prostate	GTEX-111CU-1526-SM-5N9FS
Prostate	Prostate	GTEX-111FC-2026-SM-5GZYO
Prostate	Prostate	GTEX-111YS-1726-SM-5GIED
Prostate	Prostate	GTEX-117YW-1426-SM-5EGGO
Prostate	Prostate	GTEX-117YX-1526-SM-5H12T
Prostate	Prostate	GTEX-11DXZ-1826-SM-5H12Y
Prostate	Prostate	GTEX-11DYG-2526-SM-5N9BB

Prostate	Prostate	GTEX-11EQ9-1726-SM-5HL6U
Prostate	Prostate	GTEX-11NSD-0826-SM-5986S
Prostate	Prostate	GTEX-11O72-2726-SM-5HL6K
Prostate	Prostate	GTEX-11OF3-1726-SM-5GU5Q
Prostate	Prostate	GTEX-11P7K-0626-SM-5985Z
Prostate	Prostate	GTEX-11P82-1126-SM-5BC5K
Prostate	Prostate	GTEX-11TT1-2026-SM-5EQM8
Prostate	Prostate	GTEX-11TUW-2126-SM-5GU7B
Prostate	Prostate	GTEX-11WQK-2726-SM-5EQMU
Prostate	Prostate	GTEX-11ZUS-2126-SM-5N9E6
Prostate	Prostate	GTEX-12696-2226-SM-5EQ65
Prostate	Prostate	GTEX-12BJ1-1226-SM-5LUAE
Prostate	Prostate	GTEX-12C56-1326-SM-5FQSV
Prostate	Prostate	GTEX-12ZZZ-2026-SM-5LZWJ
Prostate	Prostate	GTEX-13111-1326-SM-5GCNO
Prostate	Prostate	GTEX-131XF-5013-SM-7EWFI
Prostate	Prostate	GTEX-132NY-2826-SM-5P9G2
Prostate	Prostate	GTEX-132QS-1126-SM-5P9GC
Prostate	Prostate	GTEX-1399R-0526-SM-5IJEF
Prostate	Prostate	GTEX-1399T-1226-SM-5P9J5
Prostate	Prostate	GTEX-139T6-1526-SM-5P9G6
Prostate	Prostate	GTEX-13FHO-2826-SM-5K7ZA
Prostate	Prostate	GTEX-13FTW-1226-SM-5LZZ1
Prostate	Prostate	GTEX-13FXS-2826-SM-5LZYC
Prostate	Prostate	GTEX-13G51-2326-SM-5LZXV
Prostate	Prostate	GTEX-13N2G-1826-SM-5KM1I
Prostate	Prostate	GTEX-13NYS-1026-SM-5KLZT
Prostate	Prostate	GTEX-13O21-1126-SM-5N9EV
Prostate	Prostate	GTEX-13O61-1726-SM-5KM46
Prostate	Prostate	GTEX-13OW8-0426-SM-5J2NR
Prostate	Prostate	GTEX-13RTJ-2126-SM-5S2PZ
Prostate	Prostate	GTEX-144GM-0826-SM-5O98R
Prostate	Prostate	GTEX-145LT-0626-SM-5O99E
Prostate	Prostate	GTEX-145LU-2126-SM-5Q5E9
Prostate	Prostate	GTEX-145LV-1226-SM-5S2QH
Prostate	Prostate	GTEX-146FQ-1726-SM-5QGPX
Prostate	Prostate	GTEX-1477Z-2226-SM-5QGPG
Prostate	Prostate	GTEX-147JS-2826-SM-5YYA7
Prostate	Prostate	GTEX-148VJ-1826-SM-5Q5DV
Prostate	Prostate	GTEX-14ABY-0726-SM-5Q5DF
Prostate	Prostate	GTEX-14BIL-2126-SM-73KW3
Prostate	Prostate	GTEX-14BMV-2326-SM-5RQJ4
Prostate	Prostate	GTEX-14C5O-2826-SM-5RQI6
Prostate	Prostate	GTEX-14DAR-1026-SM-73KV3
Prostate	Prostate	GTEX-14E6E-1126-SM-5S2R4
Prostate	Prostate	GTEX-14ICK-1826-SM-69LOP
Prostate	Prostate	GTEX-14PJ2-2126-SM-5YY96
Prostate	Prostate	GTEX-14PJ3-1726-SM-5ZZVH
Prostate	Prostate	GTEX-14PJ4-1226-SM-5YY9U
Prostate	Prostate	GTEX-14PJO-2826-SM-6AJA6
Prostate	Prostate	GTEX-15CHR-1226-SM-79OON
Prostate	Prostate	GTEX-15RIF-1526-SM-6M46K
Prostate	Prostate	GTEX-16GPK-2826-SM-6LPJ6
Prostate	Prostate	GTEX-16MTA-1426-SM-6PALY

Prostate	Prostate	GTEX-16XZZ-2426-SM-7KULN
Prostate	Prostate	GTEX-16YQH-2326-SM-7EWDS
Prostate	Prostate	GTEX-178AV-0426-SM-6LPJG
Prostate	Prostate	GTEX-17EUY-2026-SM-79OMV
Prostate	Prostate	GTEX-17HHE-1126-SM-793BS
Prostate	Prostate	GTEX-17HHY-2526-SM-7IGME
Prostate	Prostate	GTEX-17HII-2526-SM-7LT8C
Prostate	Prostate	GTEX-17MFQ-1326-SM-718BX
Prostate	Prostate	GTEX-18A66-2326-SM-7LT8Q
Prostate	Prostate	GTEX-18QFQ-2026-SM-72D7J
Prostate	Prostate	GTEX-1A3MV-0826-SM-72D6J
Prostate	Prostate	GTEX-1AX8Z-2626-SM-731EP
Prostate	Prostate	GTEX-1AX9J-1426-SM-731BV
Prostate	Prostate	GTEX-1AX9K-1526-SM-73KVP
Prostate	Prostate	GTEX-1B8KE-1026-SM-731EQ
Prostate	Prostate	GTEX-1B8KZ-1626-SM-73KUU
Prostate	Prostate	GTEX-1B97I-0826-SM-731DN
Prostate	Prostate	GTEX-1C64O-2026-SM-7IGPA
Prostate	Prostate	GTEX-1C6VQ-2426-SM-7EWEU
Prostate	Prostate	GTEX-1CAMR-1626-SM-79OLC
Prostate	Prostate	GTEX-1CB4E-1726-SM-7IGMS
Prostate	Prostate	GTEX-1CB4F-2326-SM-7MKFO
Prostate	Prostate	GTEX-1EU9M-1526-SM-79OLI
Prostate	Prostate	GTEX-NFK9-2226-SM-3MJGP
Prostate	Prostate	GTEX-NPJ8-2426-SM-3MJHL
Prostate	Prostate	GTEX-OIZH-2026-SM-3NB1M
Prostate	Prostate	GTEX-OOBJ-2026-SM-3NB1R
Prostate	Prostate	GTEX-OOBK-2025-SM-3LK5S
Prostate	Prostate	GTEX-P4QS-2026-SM-3NMCG
Prostate	Prostate	GTEX-PLZ6-1126-SM-3P5ZR
Prostate	Prostate	GTEX-Q2AI-1126-SM-48U19
Prostate	Prostate	GTEX-QEG4-2226-SM-4R1JM
Prostate	Prostate	GTEX-QLQ7-1326-SM-4R1JY
Prostate	Prostate	GTEX-QMRM-1426-SM-4R1K8
Prostate	Prostate	GTEX-QV31-1026-SM-4R1K3
Prostate	Prostate	GTEX-QV44-1526-SM-4R1KI
Prostate	Prostate	GTEX-REY6-2126-SM-48FD9
Prostate	Prostate	GTEX-RM2N-1126-SM-48FCY
Prostate	Prostate	GTEX-RWSA-2026-SM-47JX8
Prostate	Prostate	GTEX-S33H-1826-SM-4AD65
Prostate	Prostate	GTEX-S3XE-1326-SM-4AD4H
Prostate	Prostate	GTEX-S4Q7-1026-SM-4AD75
Prostate	Prostate	GTEX-S95S-1026-SM-4B64M
Prostate	Prostate	GTEX-SJXC-2026-SM-4DM6N
Prostate	Prostate	GTEX-SNMC-0926-SM-4DM5U
Prostate	Prostate	GTEX-SNOS-1026-SM-4DM4Y
Prostate	Prostate	GTEX-T5JC-1226-SM-4DM7C
Prostate	Prostate	GTEX-T8EM-1826-SM-4DM7F
Prostate	Prostate	GTEX-U3ZH-0926-SM-4DXU4
Prostate	Prostate	GTEX-U3ZM-0926-SM-4DXSW
Prostate	Prostate	GTEX-U412-2026-SM-4DXSI
Prostate	Prostate	GTEX-U4B1-1426-SM-4DXTX
Prostate	Prostate	GTEX-U8XE-2526-SM-4E3IT
Prostate	Prostate	GTEX-UTHO-2726-SM-4JBH9

Prostate Prostate GTEX-V1D1-1926-SM-4JBGX
Prostate Prostate GTEX-V955-1826-SM-4JBIL
Prostate Prostate GTEX-VJYA-1226-SM-3GIJ6
Prostate Prostate GTEX-WFG7-1826-SM-3GIL1
Prostate Prostate GTEX-WFG8-1826-SM-4LVM4
Prostate Prostate GTEX-WFON-1626-SM-4LVMV
Prostate Prostate GTEX-WH7G-1726-SM-4LVKY
Prostate Prostate GTEX-WK11-2626-SM-4OOSK
Prostate Prostate GTEX-WOFM-0326-SM-4OOSL
Prostate Prostate GTEX-WY7C-2026-SM-4ONCM
Prostate Prostate GTEX-WYJK-0626-SM-4ONCZ
Prostate Prostate GTEX-WZTO-0926-SM-4PQZ3
Prostate Prostate GTEX-X5EB-1826-SM-4E3K8
Prostate Prostate GTEX-XAJ8-0626-SM-47JY4
Prostate Prostate GTEX-XGQ4-1826-SM-4AT6F
Prostate Prostate GTEX-XMK1-1026-SM-4B65H
Prostate Prostate GTEX-XPVG-2026-SM-4B65E
Prostate Prostate GTEX-XQ3S-2526-SM-4BOOG
Prostate Prostate GTEX-XXEK-1226-SM-4BRUY
Prostate Prostate GTEX-Y8E4-2126-SM-5RQHO
Prostate Prostate GTEX-Y9LG-1826-SM-4VBQC
Prostate Prostate GTEX-YB5E-1826-SM-5IFI4
Prostate Prostate GTEX-YF7O-1926-SM-4W1YQ
Prostate Prostate GTEX-YFCO-1626-SM-4W1Z3
Prostate Prostate GTEX-YJ8A-1126-SM-5IFJU
Prostate Prostate GTEX-ZDYS-1226-SM-5IJF3
Prostate Prostate GTEX-ZEX8-2026-SM-4WKF3
Prostate Prostate GTEX-ZLFU-1726-SM-4WWBU
Prostate Prostate GTEX-ZPU1-2026-SM-57WFI
Prostate Prostate GTEX-ZT9W-1626-SM-4YCDQ
Prostate Prostate GTEX-ZTTD-2726-SM-57WFA
Prostate Prostate GTEX-ZTX8-1026-SM-5EGHD
Prostate Prostate GTEX-ZUA1-2826-SM-59HLE
Prostate Prostate GTEX-ZVP2-0826-SM-59HJ6
Prostate Prostate GTEX-ZY6K-1526-SM-5GZXE
Prostate Prostate GTEX-ZYFD-2526-SM-5E45L
Prostate Prostate GTEX-ZZ64-0926-SM-5E44L
Skin - Not Sun Exposed (Suprapubic) Skin GTEX-1117F-2926-SM-5GZYI
Skin - Not Sun Exposed (Suprapubic) Skin GTEX-111CU-1126-SM-5EGIM
Skin - Not Sun Exposed (Suprapubic) Skin GTEX-111FC-2526-SM-5GZXU
Skin - Not Sun Exposed (Suprapubic) Skin GTEX-111VG-1626-SM-5EGIO
Skin - Not Sun Exposed (Suprapubic) Skin GTEX-111YS-1526-SM-5GZYW
Skin - Not Sun Exposed (Suprapubic) Skin GTEX-117YX-1926-SM-5GIEC
Skin - Not Sun Exposed (Suprapubic) Skin GTEX-1192X-2726-SM-5N9DN
Skin - Not Sun Exposed (Suprapubic) Skin GTEX-11DXW-0826-SM-5H118
Skin - Not Sun Exposed (Suprapubic) Skin GTEX-11DXX-2126-

SM-5PNYR
Skin - Not Sun Exposed (Suprapubic)	Skin	GTEX-11DXY-0126-SM-5H11Q
Skin - Not Sun Exposed (Suprapubic)	Skin	GTEX-11DXZ-2026-SM-5987S
Skin - Not Sun Exposed (Suprapubic)	Skin	GTEX-11DYG-1526-SM-5A5JS
Skin - Not Sun Exposed (Suprapubic)	Skin	GTEX-11DZ1-2426-SM-5GZZX
Skin - Not Sun Exposed (Suprapubic)	Skin	GTEX-11EI6-2826-SM-5H11D
Skin - Not Sun Exposed (Suprapubic)	Skin	GTEX-11EM3-1226-SM-5N9DD
Skin - Not Sun Exposed (Suprapubic)	Skin	GTEX-11EQ8-0926-SM-5H12V
Skin - Not Sun Exposed (Suprapubic)	Skin	GTEX-11EQ9-1626-SM-5PNY8
Skin - Not Sun Exposed (Suprapubic)	Skin	GTEX-11GS4-0226-SM-5HL77
Skin - Not Sun Exposed (Suprapubic)	Skin	GTEX-11GSP-2326-SM-5HL63
Skin - Not Sun Exposed (Suprapubic)	Skin	GTEX-11LCK-2226-SM-5HL4Q
Skin - Not Sun Exposed (Suprapubic)	Skin	GTEX-11NSD-0626-SM-5A5LU
Skin - Not Sun Exposed (Suprapubic)	Skin	GTEX-11NUK-2726-SM-5A5MC
Skin - Not Sun Exposed (Suprapubic)	Skin	GTEX-11O72-0626-SM-5PNYE
Skin - Not Sun Exposed (Suprapubic)	Skin	GTEX-11OF3-0126-SM-5PNYF
Skin - Not Sun Exposed (Suprapubic)	Skin	GTEX-11P7K-1126-SM-5HL4L
Skin - Not Sun Exposed (Suprapubic)	Skin	GTEX-11P81-2226-SM-5PNYI
Skin - Not Sun Exposed (Suprapubic)	Skin	GTEX-11P82-1426-SM-5BC5L
Skin - Not Sun Exposed (Suprapubic)	Skin	GTEX-11PRG-0726-SM-5EGL1
Skin - Not Sun Exposed (Suprapubic)	Skin	GTEX-11TT1-1826-SM-5EQLV
Skin - Not Sun Exposed (Suprapubic)	Skin	GTEX-11TUW-0626-SM-5LU9M
Skin - Not Sun Exposed (Suprapubic)	Skin	GTEX-11UD2-2526-SM-5CVNU
Skin - Not Sun Exposed (Suprapubic)	Skin	GTEX-11VI4-1126-SM-5EQMD
Skin - Not Sun Exposed (Suprapubic)	Skin	GTEX-11WQC-1826-SM-5GU59
Skin - Not Sun Exposed (Suprapubic)	Skin	GTEX-11WQK-1026-SM-5EQLX
Skin - Not Sun Exposed (Suprapubic)	Skin	GTEX-11ZTT-2126-SM-5CVLO
Skin - Not Sun Exposed (Suprapubic)	Skin	GTEX-11ZU8-0126-

SM-5EQ58
Skin - Not Sun Exposed (Suprapubic)	Skin	GTEX-11ZUS-2626-
SM-5FQUR
Skin - Not Sun Exposed (Suprapubic)	Skin	GTEX-1211K-1526-
SM-5FQU4
Skin - Not Sun Exposed (Suprapubic)	Skin	GTEX-12126-0826-
SM-5FQTZ
Skin - Not Sun Exposed (Suprapubic)	Skin	GTEX-1212Z-0126-
SM-59HK4
Skin - Not Sun Exposed (Suprapubic)	Skin	GTEX-12584-0726-
SM-5FQTK
Skin - Not Sun Exposed (Suprapubic)	Skin	GTEX-12696-0126-
SM-5EQ4L
Skin - Not Sun Exposed (Suprapubic)	Skin	GTEX-1269C-0126-
SM-5EQ5L
Skin - Not Sun Exposed (Suprapubic)	Skin	GTEX-12BJ1-0926-
SM-5EQ62
Skin - Not Sun Exposed (Suprapubic)	Skin	GTEX-12C56-1526-
SM-5FQUQ
Skin - Not Sun Exposed (Suprapubic)	Skin	GTEX-12KS4-2126-
SM-5FQUT
Skin - Not Sun Exposed (Suprapubic)	Skin	GTEX-12WSA-0426-
SM-5EQ5X
Skin - Not Sun Exposed (Suprapubic)	Skin	GTEX-12WSC-2826-
SM-5BC5Y
Skin - Not Sun Exposed (Suprapubic)	Skin	GTEX-12WSD-0626-
SM-5GCMW
Skin - Not Sun Exposed (Suprapubic)	Skin	GTEX-12WSF-1626-
SM-7KUG5
Skin - Not Sun Exposed (Suprapubic)	Skin	GTEX-12WSH-1526-
SM-5BC5W
Skin - Not Sun Exposed (Suprapubic)	Skin	GTEX-12WSJ-1126-
SM-5LZVJ
Skin - Not Sun Exposed (Suprapubic)	Skin	GTEX-12WSK-2126-
SM-5CVNC
Skin - Not Sun Exposed (Suprapubic)	Skin	GTEX-12WSL-2026-
SM-5GCML
Skin - Not Sun Exposed (Suprapubic)	Skin	GTEX-12WSM-1926-
SM-5BC65
Skin - Not Sun Exposed (Suprapubic)	Skin	GTEX-12WSN-1426-
SM-5GCO6
Skin - Not Sun Exposed (Suprapubic)	Skin	GTEX-12ZZX-2426-
SM-5GCOE
Skin - Not Sun Exposed (Suprapubic)	Skin	GTEX-12ZZY-0726-
SM-5EQ66
Skin - Not Sun Exposed (Suprapubic)	Skin	GTEX-12ZZZ-0726-
SM-5GCNZ
Skin - Not Sun Exposed (Suprapubic)	Skin	GTEX-13112-0126-
SM-5P9IU
Skin - Not Sun Exposed (Suprapubic)	Skin	GTEX-13113-1626-
SM-5EQ4I
Skin - Not Sun Exposed (Suprapubic)	Skin	GTEX-1313W-0626-
SM-5EQ4H
Skin - Not Sun Exposed (Suprapubic)	Skin	GTEX-1314G-1326-

SM-5BC6E
Skin - Not Sun Exposed (Suprapubic)	Skin	GTEX-131XE-1626-
SM-5K7VO
Skin - Not Sun Exposed (Suprapubic)	Skin	GTEX-131XF-2626-
SM-5EQ6K
Skin - Not Sun Exposed (Suprapubic)	Skin	GTEX-131XG-0326-
SM-5L3DX
Skin - Not Sun Exposed (Suprapubic)	Skin	GTEX-131XH-0126-
SM-5LZUY
Skin - Not Sun Exposed (Suprapubic)	Skin	GTEX-131XW-0626-
SM-5PNVD
Skin - Not Sun Exposed (Suprapubic)	Skin	GTEX-131YS-2826-
SM-5EQ47
Skin - Not Sun Exposed (Suprapubic)	Skin	GTEX-132AR-0326-
SM-5KM2C
Skin - Not Sun Exposed (Suprapubic)	Skin	GTEX-133LE-2326-
SM-5K7W3
Skin - Not Sun Exposed (Suprapubic)	Skin	GTEX-1399Q-2326-
SM-5KM2X
Skin - Not Sun Exposed (Suprapubic)	Skin	GTEX-1399R-1326-
SM-5PNYS
Skin - Not Sun Exposed (Suprapubic)	Skin	GTEX-1399S-0126-
SM-5IJCF
Skin - Not Sun Exposed (Suprapubic)	Skin	GTEX-1399U-1126-
SM-5L3DB
Skin - Not Sun Exposed (Suprapubic)	Skin	GTEX-139D8-1126-
SM-5LU8W
Skin - Not Sun Exposed (Suprapubic)	Skin	GTEX-139T8-0626-
SM-5KLZX
Skin - Not Sun Exposed (Suprapubic)	Skin	GTEX-139TS-2126-
SM-5K7XC
Skin - Not Sun Exposed (Suprapubic)	Skin	GTEX-139TT-2126-
SM-5LZWC
Skin - Not Sun Exposed (Suprapubic)	Skin	GTEX-139TU-0726-
SM-5L3EK
Skin - Not Sun Exposed (Suprapubic)	Skin	GTEX-139UW-0326-
SM-5J1ML
Skin - Not Sun Exposed (Suprapubic)	Skin	GTEX-139YR-1026-
SM-5LZYN
Skin - Not Sun Exposed (Suprapubic)	Skin	GTEX-13D11-0926-
SM-5J2NC
Skin - Not Sun Exposed (Suprapubic)	Skin	GTEX-13FH7-1126-
SM-5K7UG
Skin - Not Sun Exposed (Suprapubic)	Skin	GTEX-13FHO-0426-
SM-5L3F5
Skin - Not Sun Exposed (Suprapubic)	Skin	GTEX-13FLV-0126-
SM-5K7WT
Skin - Not Sun Exposed (Suprapubic)	Skin	GTEX-13FTW-0326-
SM-5K7UW
Skin - Not Sun Exposed (Suprapubic)	Skin	GTEX-13FTX-1526-
SM-5K7VJ
Skin - Not Sun Exposed (Suprapubic)	Skin	GTEX-13FTY-2126-
SM-5IFFB
Skin - Not Sun Exposed (Suprapubic)	Skin	GTEX-13G51-1826-

SM-5KLZH
Skin - Not Sun Exposed (Suprapubic)	Skin	GTEX-13IVO-0726-
SM-5LZYV
Skin - Not Sun Exposed (Suprapubic)	Skin	GTEX-13JUV-2026-
SM-5K7UA
Skin - Not Sun Exposed (Suprapubic)	Skin	GTEX-13JVG-0726-
SM-5KM16
Skin - Not Sun Exposed (Suprapubic)	Skin	GTEX-13N11-2326-
SM-5IJBF
Skin - Not Sun Exposed (Suprapubic)	Skin	GTEX-13N1W-2426-
SM-5IJD1
Skin - Not Sun Exposed (Suprapubic)	Skin	GTEX-13N2G-0226-
SM-5K7VF
Skin - Not Sun Exposed (Suprapubic)	Skin	GTEX-13NYB-0126-
SM-5J1MD
Skin - Not Sun Exposed (Suprapubic)	Skin	GTEX-13NYC-0126-
SM-5L3F4
Skin - Not Sun Exposed (Suprapubic)	Skin	GTEX-13NYS-0626-
SM-5L3F1
Skin - Not Sun Exposed (Suprapubic)	Skin	GTEX-13NZ8-1226-
SM-5KM4A
Skin - Not Sun Exposed (Suprapubic)	Skin	GTEX-13NZ9-2626-
SM-5L3FK
Skin - Not Sun Exposed (Suprapubic)	Skin	GTEX-13O3O-2326-
SM-5IJD6
Skin - Not Sun Exposed (Suprapubic)	Skin	GTEX-13O3Q-0126-
SM-5KM34
Skin - Not Sun Exposed (Suprapubic)	Skin	GTEX-13O61-1426-
SM-5KM3D
Skin - Not Sun Exposed (Suprapubic)	Skin	GTEX-13OVG-1626-
SM-5J1NF
Skin - Not Sun Exposed (Suprapubic)	Skin	GTEX-13OVH-0526-
SM-5L3E6
Skin - Not Sun Exposed (Suprapubic)	Skin	GTEX-13OVJ-0826-
SM-5J1OK
Skin - Not Sun Exposed (Suprapubic)	Skin	GTEX-13OVK-1426-
SM-6PAN7
Skin - Not Sun Exposed (Suprapubic)	Skin	GTEX-13OVL-0126-
SM-5L3GJ
Skin - Not Sun Exposed (Suprapubic)	Skin	GTEX-13OW5-2326-
SM-5L3HO
Skin - Not Sun Exposed (Suprapubic)	Skin	GTEX-13OW6-2726-
SM-5J2OE
Skin - Not Sun Exposed (Suprapubic)	Skin	GTEX-13OW7-0426-
SM-5K7VK
Skin - Not Sun Exposed (Suprapubic)	Skin	GTEX-13OW8-1026-
SM-5J1NQ
Skin - Not Sun Exposed (Suprapubic)	Skin	GTEX-13PDP-0726-
SM-5L3HP
Skin - Not Sun Exposed (Suprapubic)	Skin	GTEX-13PL6-1126-
SM-5L3IE
Skin - Not Sun Exposed (Suprapubic)	Skin	GTEX-13PVQ-0626-
SM-5LU47
Skin - Not Sun Exposed (Suprapubic)	Skin	GTEX-13PVR-0426-

SM-5RQK2
Skin - Not Sun Exposed (Suprapubic)	Skin	GTEX-13QBU-1226-SM-5LU4G
Skin - Not Sun Exposed (Suprapubic)	Skin	GTEX-13QJC-0126-SM-5ZZWJ
Skin - Not Sun Exposed (Suprapubic)	Skin	GTEX-13RTJ-0126-SM-5S2NN
Skin - Not Sun Exposed (Suprapubic)	Skin	GTEX-13SLW-0626-SM-5Q5ER
Skin - Not Sun Exposed (Suprapubic)	Skin	GTEX-13SLX-2226-SM-62LEP
Skin - Not Sun Exposed (Suprapubic)	Skin	GTEX-13U4I-2226-SM-5LU5H
Skin - Not Sun Exposed (Suprapubic)	Skin	GTEX-13VXT-1926-SM-5K7UO
Skin - Not Sun Exposed (Suprapubic)	Skin	GTEX-13VXU-0626-SM-5L3F9
Skin - Not Sun Exposed (Suprapubic)	Skin	GTEX-13W3W-0726-SM-5IJDQ
Skin - Not Sun Exposed (Suprapubic)	Skin	GTEX-13X6H-0926-SM-5SIB2
Skin - Not Sun Exposed (Suprapubic)	Skin	GTEX-13X6I-1826-SM-5SIAO
Skin - Not Sun Exposed (Suprapubic)	Skin	GTEX-13X6J-0626-SM-5LU5F
Skin - Not Sun Exposed (Suprapubic)	Skin	GTEX-13X6K-0626-SM-5LU5D
Skin - Not Sun Exposed (Suprapubic)	Skin	GTEX-13YAN-0226-SM-5TDC6
Skin - Not Sun Exposed (Suprapubic)	Skin	GTEX-144FL-0626-SM-5LU43
Skin - Not Sun Exposed (Suprapubic)	Skin	GTEX-144GL-0626-SM-5LU4D
Skin - Not Sun Exposed (Suprapubic)	Skin	GTEX-144GM-0526-SM-79OJO
Skin - Not Sun Exposed (Suprapubic)	Skin	GTEX-144GO-2026-SM-5O99P
Skin - Not Sun Exposed (Suprapubic)	Skin	GTEX-145LS-0626-SM-7EPG7
Skin - Not Sun Exposed (Suprapubic)	Skin	GTEX-145LT-0526-SM-5LUB2
Skin - Not Sun Exposed (Suprapubic)	Skin	GTEX-145LU-1926-SM-79OJN
Skin - Not Sun Exposed (Suprapubic)	Skin	GTEX-145ME-1726-SM-5Q5EU
Skin - Not Sun Exposed (Suprapubic)	Skin	GTEX-145MF-0926-SM-5Q5EA
Skin - Not Sun Exposed (Suprapubic)	Skin	GTEX-145MG-1526-SM-5Q5EM
Skin - Not Sun Exposed (Suprapubic)	Skin	GTEX-145MH-2826-SM-5RQJF
Skin - Not Sun Exposed (Suprapubic)	Skin	GTEX-145MI-0626-SM-5SI8S
Skin - Not Sun Exposed (Suprapubic)	Skin	GTEX-145MN-1526-

SM-5SI9T
Skin - Not Sun Exposed (Suprapubic)	Skin	GTEX-145MO-2826-
SM-5LU96
Skin - Not Sun Exposed (Suprapubic)	Skin	GTEX-146FQ-2126-
SM-5RQHU
Skin - Not Sun Exposed (Suprapubic)	Skin	GTEX-14753-0726-
SM-5QGQO
Skin - Not Sun Exposed (Suprapubic)	Skin	GTEX-1477Z-0326-
SM-7IGLA
Skin - Not Sun Exposed (Suprapubic)	Skin	GTEX-147F3-1726-
SM-5SIAW
Skin - Not Sun Exposed (Suprapubic)	Skin	GTEX-147F4-0726-
SM-5TDDD
Skin - Not Sun Exposed (Suprapubic)	Skin	GTEX-147GR-2426-
SM-5ZZWQ
Skin - Not Sun Exposed (Suprapubic)	Skin	GTEX-147JS-0226-
SM-5S2U9
Skin - Not Sun Exposed (Suprapubic)	Skin	GTEX-148VJ-0626-
SM-5LUAW
Skin - Not Sun Exposed (Suprapubic)	Skin	GTEX-1497J-0226-
SM-5QGPU
Skin - Not Sun Exposed (Suprapubic)	Skin	GTEX-14A5H-0826-
SM-5QGPJ
Skin - Not Sun Exposed (Suprapubic)	Skin	GTEX-14A6H-2026-
SM-5Q5DD
Skin - Not Sun Exposed (Suprapubic)	Skin	GTEX-14ABY-2026-
SM-62LDL
Skin - Not Sun Exposed (Suprapubic)	Skin	GTEX-14AS3-1526-
SM-5Q5ED
Skin - Not Sun Exposed (Suprapubic)	Skin	GTEX-14B4R-1326-
SM-5Q5EZ
Skin - Not Sun Exposed (Suprapubic)	Skin	GTEX-14BIN-1126-
SM-5RQJZ
Skin - Not Sun Exposed (Suprapubic)	Skin	GTEX-14BMU-1226-
SM-5S2OR
Skin - Not Sun Exposed (Suprapubic)	Skin	GTEX-14BMV-2826-
SM-5TDED
Skin - Not Sun Exposed (Suprapubic)	Skin	GTEX-14C38-0726-
SM-5SI6M
Skin - Not Sun Exposed (Suprapubic)	Skin	GTEX-14C39-1726-
SM-5RQIL
Skin - Not Sun Exposed (Suprapubic)	Skin	GTEX-14C5O-0326-
SM-5ZZUX
Skin - Not Sun Exposed (Suprapubic)	Skin	GTEX-14DAQ-0626-
SM-793DS
Skin - Not Sun Exposed (Suprapubic)	Skin	GTEX-14DAR-1226-
SM-73KYV
Skin - Not Sun Exposed (Suprapubic)	Skin	GTEX-14E1K-2026-
SM-7DUEI
Skin - Not Sun Exposed (Suprapubic)	Skin	GTEX-14E6C-2426-
SM-5S2NF
Skin - Not Sun Exposed (Suprapubic)	Skin	GTEX-14E6E-0526-
SM-793DN
Skin - Not Sun Exposed (Suprapubic)	Skin	GTEX-14ICL-1626-

SM-5YYAO
Skin - Not Sun Exposed (Suprapubic)	Skin	GTEX-14JG1-1526-SM-5YY9X
Skin - Not Sun Exposed (Suprapubic)	Skin	GTEX-14LLW-2626-SM-62LDZ
Skin - Not Sun Exposed (Suprapubic)	Skin	GTEX-14LZ3-0126-SM-69LPM
Skin - Not Sun Exposed (Suprapubic)	Skin	GTEX-14PHX-2226-SM-6EU2P
Skin - Not Sun Exposed (Suprapubic)	Skin	GTEX-14PHY-1226-SM-5ZZWK
Skin - Not Sun Exposed (Suprapubic)	Skin	GTEX-14PII-0726-SM-6EU2B
Skin - Not Sun Exposed (Suprapubic)	Skin	GTEX-14PJ2-0626-SM-6LLII
Skin - Not Sun Exposed (Suprapubic)	Skin	GTEX-14PJ3-1126-SM-5ZZVX
Skin - Not Sun Exposed (Suprapubic)	Skin	GTEX-14PJ4-0226-SM-5YY92
Skin - Not Sun Exposed (Suprapubic)	Skin	GTEX-14PJ5-1526-SM-6LLIL
Skin - Not Sun Exposed (Suprapubic)	Skin	GTEX-14PJ6-2626-SM-6EU2T
Skin - Not Sun Exposed (Suprapubic)	Skin	GTEX-14PJN-1826-SM-6AJ98
Skin - Not Sun Exposed (Suprapubic)	Skin	GTEX-14PJO-2526-SM-6AJBQ
Skin - Not Sun Exposed (Suprapubic)	Skin	GTEX-14PK6-0226-SM-62LF5
Skin - Not Sun Exposed (Suprapubic)	Skin	GTEX-14PKU-0126-SM-6LLHV
Skin - Not Sun Exposed (Suprapubic)	Skin	GTEX-14PKV-1126-SM-6LLJ1
Skin - Not Sun Exposed (Suprapubic)	Skin	GTEX-14XAO-1226-SM-6EU1C
Skin - Not Sun Exposed (Suprapubic)	Skin	GTEX-15CHC-1126-SM-6LLHQ
Skin - Not Sun Exposed (Suprapubic)	Skin	GTEX-15CHQ-0326-SM-69LQD
Skin - Not Sun Exposed (Suprapubic)	Skin	GTEX-15CHR-1126-SM-7EWF4
Skin - Not Sun Exposed (Suprapubic)	Skin	GTEX-15D1Q-2826-SM-6LLH7
Skin - Not Sun Exposed (Suprapubic)	Skin	GTEX-15DCZ-0626-SM-6AJ9I
Skin - Not Sun Exposed (Suprapubic)	Skin	GTEX-15DDE-2926-SM-6LLJ3
Skin - Not Sun Exposed (Suprapubic)	Skin	GTEX-15DYW-2726-SM-6EU2I
Skin - Not Sun Exposed (Suprapubic)	Skin	GTEX-15EO6-2426-SM-6PALZ
Skin - Not Sun Exposed (Suprapubic)	Skin	GTEX-15ER7-0626-SM-6PANF
Skin - Not Sun Exposed (Suprapubic)	Skin	GTEX-15EU6-2226-

SM-6EU2L
Skin - Not Sun Exposed (Suprapubic)	Skin	GTEX-15FZZ-0526-
SM-7KUGD
Skin - Not Sun Exposed (Suprapubic)	Skin	GTEX-15G19-2426-
SM-7KUMR
Skin - Not Sun Exposed (Suprapubic)	Skin	GTEX-15G1A-1626-
SM-7KUKN
Skin - Not Sun Exposed (Suprapubic)	Skin	GTEX-15RIE-1926-
SM-7KUKJ
Skin - Not Sun Exposed (Suprapubic)	Skin	GTEX-15RIG-0726-
SM-7KUM7
Skin - Not Sun Exposed (Suprapubic)	Skin	GTEX-15RJ7-2126-
SM-6PAMD
Skin - Not Sun Exposed (Suprapubic)	Skin	GTEX-15RJE-2826-
SM-7KUM9
Skin - Not Sun Exposed (Suprapubic)	Skin	GTEX-15SHW-0226-
SM-7LTAT
Skin - Not Sun Exposed (Suprapubic)	Skin	GTEX-15SKB-2826-
SM-6PAMH
Skin - Not Sun Exposed (Suprapubic)	Skin	GTEX-15UF6-1926-
SM-7KUDV
Skin - Not Sun Exposed (Suprapubic)	Skin	GTEX-16AAH-0626-
SM-793C9
Skin - Not Sun Exposed (Suprapubic)	Skin	GTEX-16BQI-1326-
SM-6M486
Skin - Not Sun Exposed (Suprapubic)	Skin	GTEX-16GPK-0626-
SM-7KFSF
Skin - Not Sun Exposed (Suprapubic)	Skin	GTEX-16MT8-2926-
SM-7DUFO
Skin - Not Sun Exposed (Suprapubic)	Skin	GTEX-16MT9-0126-
SM-7EPI8
Skin - Not Sun Exposed (Suprapubic)	Skin	GTEX-16MTA-0926-
SM-6M47Z
Skin - Not Sun Exposed (Suprapubic)	Skin	GTEX-16NGA-0726-
SM-731AO
Skin - Not Sun Exposed (Suprapubic)	Skin	GTEX-16NPX-0726-
SM-7KULC
Skin - Not Sun Exposed (Suprapubic)	Skin	GTEX-16XZZ-0726-
SM-7KUF2
Skin - Not Sun Exposed (Suprapubic)	Skin	GTEX-16YQH-2226-
SM-7IGM2
Skin - Not Sun Exposed (Suprapubic)	Skin	GTEX-16Z82-2626-
SM-7KULU
Skin - Not Sun Exposed (Suprapubic)	Skin	GTEX-178AV-1726-
SM-7KUL8
Skin - Not Sun Exposed (Suprapubic)	Skin	GTEX-17EVP-2626-
SM-7IGMA
Skin - Not Sun Exposed (Suprapubic)	Skin	GTEX-17EVQ-0126-
SM-7IGLR
Skin - Not Sun Exposed (Suprapubic)	Skin	GTEX-17F96-0126-
SM-7EWDU
Skin - Not Sun Exposed (Suprapubic)	Skin	GTEX-17F97-2626-
SM-7IGM5
Skin - Not Sun Exposed (Suprapubic)	Skin	GTEX-17F98-1326-

SM-7DUEP
Skin - Not Sun Exposed (Suprapubic)	Skin	GTEX-17F9E-2326-
SM-79OKD
Skin - Not Sun Exposed (Suprapubic)	Skin	GTEX-17F9Y-0126-
SM-79OKX
Skin - Not Sun Exposed (Suprapubic)	Skin	GTEX-17HG3-2626-
SM-7DUF2
Skin - Not Sun Exposed (Suprapubic)	Skin	GTEX-17HGU-2326-
SM-7DUFE
Skin - Not Sun Exposed (Suprapubic)	Skin	GTEX-17HHE-1326-
SM-7EWDZ
Skin - Not Sun Exposed (Suprapubic)	Skin	GTEX-17HHY-0426-
SM-7DHLC
Skin - Not Sun Exposed (Suprapubic)	Skin	GTEX-17HII-1426-
SM-7EPGG
Skin - Not Sun Exposed (Suprapubic)	Skin	GTEX-17JCI-2926-
SM-7DUFF
Skin - Not Sun Exposed (Suprapubic)	Skin	GTEX-17KNJ-2126-
SM-718AM
Skin - Not Sun Exposed (Suprapubic)	Skin	GTEX-17MF6-2626-
SM-79OKQ
Skin - Not Sun Exposed (Suprapubic)	Skin	GTEX-17MFQ-1526-
SM-7KFRR
Skin - Not Sun Exposed (Suprapubic)	Skin	GTEX-183FY-1026-
SM-7939A
Skin - Not Sun Exposed (Suprapubic)	Skin	GTEX-183WM-0326-
SM-72D5V
Skin - Not Sun Exposed (Suprapubic)	Skin	GTEX-18465-2826-
SM-7LT9J
Skin - Not Sun Exposed (Suprapubic)	Skin	GTEX-18A66-0626-
SM-7LT8Z
Skin - Not Sun Exposed (Suprapubic)	Skin	GTEX-18A67-1626-
SM-7LT9T
Skin - Not Sun Exposed (Suprapubic)	Skin	GTEX-18A6Q-0626-
SM-7KFRP
Skin - Not Sun Exposed (Suprapubic)	Skin	GTEX-18A7A-0126-
SM-731AH
Skin - Not Sun Exposed (Suprapubic)	Skin	GTEX-18A7B-2726-
SM-7LG56
Skin - Not Sun Exposed (Suprapubic)	Skin	GTEX-18D9B-0926-
SM-7LG4U
Skin - Not Sun Exposed (Suprapubic)	Skin	GTEX-18D9U-0126-
SM-731BM
Skin - Not Sun Exposed (Suprapubic)	Skin	GTEX-18QFQ-1826-
SM-72D52
Skin - Not Sun Exposed (Suprapubic)	Skin	GTEX-1A32A-0626-
SM-731AQ
Skin - Not Sun Exposed (Suprapubic)	Skin	GTEX-1A3MV-1826-
SM-731CD
Skin - Not Sun Exposed (Suprapubic)	Skin	GTEX-1A3MW-0626-
SM-731CB
Skin - Not Sun Exposed (Suprapubic)	Skin	GTEX-1A8G6-2726-
SM-731AR
Skin - Not Sun Exposed (Suprapubic)	Skin	GTEX-1A8G7-1326-

SM-73KVM
Skin - Not Sun Exposed (Suprapubic)	Skin	GTEX-1AMEY-0826-
SM-72D65
Skin - Not Sun Exposed (Suprapubic)	Skin	GTEX-1AMFI-1326-
SM-7939R
Skin - Not Sun Exposed (Suprapubic)	Skin	GTEX-1AX8Z-0726-
SM-73KTS
Skin - Not Sun Exposed (Suprapubic)	Skin	GTEX-1AX9I-2226-
SM-73KUO
Skin - Not Sun Exposed (Suprapubic)	Skin	GTEX-1AX9J-1326-
SM-731BJ
Skin - Not Sun Exposed (Suprapubic)	Skin	GTEX-1B8KE-1126-
SM-7EPHI
Skin - Not Sun Exposed (Suprapubic)	Skin	GTEX-1B8KZ-0326-
SM-73KY4
Skin - Not Sun Exposed (Suprapubic)	Skin	GTEX-1B8SF-0126-
SM-7DUG3
Skin - Not Sun Exposed (Suprapubic)	Skin	GTEX-1B932-2326-
SM-79OL5
Skin - Not Sun Exposed (Suprapubic)	Skin	GTEX-1B933-2426-
SM-7DHMD
Skin - Not Sun Exposed (Suprapubic)	Skin	GTEX-1B97I-0626-
SM-73KUZ
Skin - Not Sun Exposed (Suprapubic)	Skin	GTEX-1B996-0426-
SM-7EWF8
Skin - Not Sun Exposed (Suprapubic)	Skin	GTEX-1BAJH-2426-
SM-7IGP8
Skin - Not Sun Exposed (Suprapubic)	Skin	GTEX-1C2JI-1026-
SM-73KY1
Skin - Not Sun Exposed (Suprapubic)	Skin	GTEX-1C6VQ-1226-
SM-73KYD
Skin - Not Sun Exposed (Suprapubic)	Skin	GTEX-1C6WA-2626-
SM-7MKGM
Skin - Not Sun Exposed (Suprapubic)	Skin	GTEX-1CAV2-0126-
SM-7IGN7
Skin - Not Sun Exposed (Suprapubic)	Skin	GTEX-1CB4E-0926-
SM-7EPHT
Skin - Not Sun Exposed (Suprapubic)	Skin	GTEX-1CB4F-1026-
SM-7DUH1
Skin - Not Sun Exposed (Suprapubic)	Skin	GTEX-1CB4H-1126-
SM-7MKGL
Skin - Not Sun Exposed (Suprapubic)	Skin	GTEX-1E1VI-0326-
SM-793D1
Skin - Not Sun Exposed (Suprapubic)	Skin	GTEX-1EH9U-2826-
SM-7DUGT
Skin - Not Sun Exposed (Suprapubic)	Skin	GTEX-1EKGG-0626-
SM-7DUGJ
Skin - Not Sun Exposed (Suprapubic)	Skin	GTEX-1EN7A-2426-
SM-793DB
Skin - Not Sun Exposed (Suprapubic)	Skin	GTEX-1EU9M-1426-
SM-793AI
Skin - Not Sun Exposed (Suprapubic)	Skin	GTEX-1EWIQ-0326-
SM-7DHMQ
Skin - Not Sun Exposed (Suprapubic)	Skin	GTEX-1EX96-1026-

SM-7MKG2
Skin - Not Sun Exposed (Suprapubic)	Skin	GTEX-1F52S-2926-SM-7MKGA
Skin - Not Sun Exposed (Suprapubic)	Skin	GTEX-U8XE-0226-SM-4E3J3
Skin - Not Sun Exposed (Suprapubic)	Skin	GTEX-UPJH-0226-SM-3GADV
Skin - Not Sun Exposed (Suprapubic)	Skin	GTEX-UPK5-0426-SM-3GAEK
Skin - Not Sun Exposed (Suprapubic)	Skin	GTEX-V1D1-2026-SM-3GAF4
Skin - Not Sun Exposed (Suprapubic)	Skin	GTEX-VJYA-1526-SM-3GIJV
Skin - Not Sun Exposed (Suprapubic)	Skin	GTEX-VUSG-2326-SM-4KL1U
Skin - Not Sun Exposed (Suprapubic)	Skin	GTEX-W5X1-2526-SM-3GILC
Skin - Not Sun Exposed (Suprapubic)	Skin	GTEX-WEY5-2326-SM-3GIKK
Skin - Not Sun Exposed (Suprapubic)	Skin	GTEX-WFG7-2026-SM-5SI7P
Skin - Not Sun Exposed (Suprapubic)	Skin	GTEX-WFG8-2126-SM-3GIKQ
Skin - Not Sun Exposed (Suprapubic)	Skin	GTEX-WFON-1926-SM-3LK7L
Skin - Not Sun Exposed (Suprapubic)	Skin	GTEX-WH7G-2026-SM-3NMBL
Skin - Not Sun Exposed (Suprapubic)	Skin	GTEX-WHPG-2626-SM-3NMBR
Skin - Not Sun Exposed (Suprapubic)	Skin	GTEX-WHSB-1326-SM-3LK6W
Skin - Not Sun Exposed (Suprapubic)	Skin	GTEX-WHSE-0126-SM-3NMBT
Skin - Not Sun Exposed (Suprapubic)	Skin	GTEX-WHWD-1826-SM-3LK6I
Skin - Not Sun Exposed (Suprapubic)	Skin	GTEX-WI4N-2726-SM-3LK7Y
Skin - Not Sun Exposed (Suprapubic)	Skin	GTEX-WK11-2726-SM-3NMAQ
Skin - Not Sun Exposed (Suprapubic)	Skin	GTEX-WRHU-2826-SM-3MJG8
Skin - Not Sun Exposed (Suprapubic)	Skin	GTEX-WXYG-2026-SM-4E3IY
Skin - Not Sun Exposed (Suprapubic)	Skin	GTEX-WY7C-2126-SM-3NB2R
Skin - Not Sun Exposed (Suprapubic)	Skin	GTEX-WYBS-0626-SM-3NMAS
Skin - Not Sun Exposed (Suprapubic)	Skin	GTEX-WYJK-0526-SM-3NM8Z
Skin - Not Sun Exposed (Suprapubic)	Skin	GTEX-WYVS-1626-SM-3NM9R
Skin - Not Sun Exposed (Suprapubic)	Skin	GTEX-X3Y1-2026-SM-3P5YM
Skin - Not Sun Exposed (Suprapubic)	Skin	GTEX-X4EO-2926-

SM-4E3JH
Skin - Not Sun Exposed (Suprapubic)   Skin   GTEX-X4EP-0626-
SM-3P621
Skin - Not Sun Exposed (Suprapubic)   Skin   GTEX-X585-2426-
SM-46MW2
Skin - Not Sun Exposed (Suprapubic)   Skin   GTEX-X5EB-1926-
SM-4E3IW
Skin - Not Sun Exposed (Suprapubic)   Skin   GTEX-X8HC-0626-
SM-4E3HQ
Skin - Not Sun Exposed (Suprapubic)   Skin   GTEX-XAJ8-1426-
SM-47JYM
Skin - Not Sun Exposed (Suprapubic)   Skin   GTEX-XBEC-0726-
SM-4QARZ
Skin - Not Sun Exposed (Suprapubic)   Skin   GTEX-XBED-1926-
SM-47JYP
Skin - Not Sun Exposed (Suprapubic)   Skin   GTEX-XBEW-1126-
SM-4QARW
Skin - Not Sun Exposed (Suprapubic)   Skin   GTEX-XK95-0226-
SM-4AT58
Skin - Not Sun Exposed (Suprapubic)   Skin   GTEX-XMD2-2126-
SM-4YCF8
Skin - Not Sun Exposed (Suprapubic)   Skin   GTEX-XOT4-0626-
SM-4B66L
Skin - Not Sun Exposed (Suprapubic)   Skin   GTEX-XPVG-2126-
SM-4B667
Skin - Not Sun Exposed (Suprapubic)   Skin   GTEX-XQ3S-1426-
SM-4BOPR
Skin - Not Sun Exposed (Suprapubic)   Skin   GTEX-XQ8I-0926-
SM-4BOOF
Skin - Not Sun Exposed (Suprapubic)   Skin   GTEX-XUW1-0926-
SM-4BONX
Skin - Not Sun Exposed (Suprapubic)   Skin   GTEX-XV7Q-1826-
SM-4BRUV
Skin - Not Sun Exposed (Suprapubic)   Skin   GTEX-XXEK-1626-
SM-4BRUZ
Skin - Not Sun Exposed (Suprapubic)   Skin   GTEX-XYKS-1426-
SM-4BRUO
Skin - Not Sun Exposed (Suprapubic)   Skin   GTEX-Y111-0626-
SM-4SOIT
Skin - Not Sun Exposed (Suprapubic)   Skin   GTEX-Y114-2126-
SM-4TT8B
Skin - Not Sun Exposed (Suprapubic)   Skin   GTEX-Y3I4-1726-
SM-4TT89
Skin - Not Sun Exposed (Suprapubic)   Skin   GTEX-Y3IK-1626-
SM-4YCF5
Skin - Not Sun Exposed (Suprapubic)   Skin   GTEX-Y5LM-0926-
SM-6LLJG
Skin - Not Sun Exposed (Suprapubic)   Skin   GTEX-Y5V5-0126-
SM-5S2NW
Skin - Not Sun Exposed (Suprapubic)   Skin   GTEX-Y5V6-2326-
SM-4VDSA
Skin - Not Sun Exposed (Suprapubic)   Skin   GTEX-Y8E4-2026-
SM-5IFJB
Skin - Not Sun Exposed (Suprapubic)   Skin   GTEX-Y8LW-1526-

SM-5IFHL
Skin - Not Sun Exposed (Suprapubic)	Skin	GTEX-Y9LG-1126-SM-4VBQ7
Skin - Not Sun Exposed (Suprapubic)	Skin	GTEX-YB5K-2026-SM-4VDT3
Skin - Not Sun Exposed (Suprapubic)	Skin	GTEX-YEC3-1326-SM-4WWEL
Skin - Not Sun Exposed (Suprapubic)	Skin	GTEX-YEC4-1826-SM-4W1YP
Skin - Not Sun Exposed (Suprapubic)	Skin	GTEX-YECK-0326-SM-4W1YS
Skin - Not Sun Exposed (Suprapubic)	Skin	GTEX-YF7O-1526-SM-5IFI6
Skin - Not Sun Exposed (Suprapubic)	Skin	GTEX-YFCO-1226-SM-5LUAT
Skin - Not Sun Exposed (Suprapubic)	Skin	GTEX-YJ8O-2526-SM-5HL85
Skin - Not Sun Exposed (Suprapubic)	Skin	GTEX-Z93S-1626-SM-5CVMB
Skin - Not Sun Exposed (Suprapubic)	Skin	GTEX-ZA64-1026-SM-5HL8R
Skin - Not Sun Exposed (Suprapubic)	Skin	GTEX-ZAB4-2126-SM-5HL8U
Skin - Not Sun Exposed (Suprapubic)	Skin	GTEX-ZAJG-1526-SM-5CVML
Skin - Not Sun Exposed (Suprapubic)	Skin	GTEX-ZAK1-0626-SM-5HL8E
Skin - Not Sun Exposed (Suprapubic)	Skin	GTEX-ZC5H-1526-SM-5HL9Y
Skin - Not Sun Exposed (Suprapubic)	Skin	GTEX-ZDTS-0826-SM-4WKHL
Skin - Not Sun Exposed (Suprapubic)	Skin	GTEX-ZDTT-0426-SM-4WKFL
Skin - Not Sun Exposed (Suprapubic)	Skin	GTEX-ZDXO-3126-SM-5IJDF
Skin - Not Sun Exposed (Suprapubic)	Skin	GTEX-ZDYS-2426-SM-4WKGI
Skin - Not Sun Exposed (Suprapubic)	Skin	GTEX-ZE7O-2726-SM-51MT5
Skin - Not Sun Exposed (Suprapubic)	Skin	GTEX-ZE9C-2526-SM-5IJDR
Skin - Not Sun Exposed (Suprapubic)	Skin	GTEX-ZF29-0926-SM-4WKFZ
Skin - Not Sun Exposed (Suprapubic)	Skin	GTEX-ZF2S-1626-SM-57WES
Skin - Not Sun Exposed (Suprapubic)	Skin	GTEX-ZLFU-1826-SM-4WWBV
Skin - Not Sun Exposed (Suprapubic)	Skin	GTEX-ZLV1-1526-SM-4WWBC
Skin - Not Sun Exposed (Suprapubic)	Skin	GTEX-ZLWG-0826-SM-59888
Skin - Not Sun Exposed (Suprapubic)	Skin	GTEX-ZP4G-1426-SM-4WWCL
Skin - Not Sun Exposed (Suprapubic)	Skin	GTEX-ZPCL-1126-

SM-4WWFE
Skin - Not Sun Exposed (Suprapubic)   Skin   GTEX-ZPU1-1126-
SM-4YCF3
Skin - Not Sun Exposed (Suprapubic)   Skin   GTEX-ZT9W-1126-
SM-57WDR
Skin - Not Sun Exposed (Suprapubic)   Skin   GTEX-ZT9X-1526-
SM-5DUXD
Skin - Not Sun Exposed (Suprapubic)   Skin   GTEX-ZTSS-1426-
SM-5985P
Skin - Not Sun Exposed (Suprapubic)   Skin   GTEX-ZTTD-2826-
SM-4YCFA
Skin - Not Sun Exposed (Suprapubic)   Skin   GTEX-ZTX8-1326-
SM-4YCEL
Skin - Not Sun Exposed (Suprapubic)   Skin   GTEX-ZU9S-1826-
SM-5NQ9E
Skin - Not Sun Exposed (Suprapubic)   Skin   GTEX-ZUA1-2626-
SM-5NQ92
Skin - Not Sun Exposed (Suprapubic)   Skin   GTEX-ZVE1-0726-
SM-51MRN
Skin - Not Sun Exposed (Suprapubic)   Skin   GTEX-ZVE2-2826-
SM-5GU51
Skin - Not Sun Exposed (Suprapubic)   Skin   GTEX-ZVP2-0326-
SM-57WBP
Skin - Not Sun Exposed (Suprapubic)   Skin   GTEX-ZVT2-0126-
SM-5GIDQ
Skin - Not Sun Exposed (Suprapubic)   Skin   GTEX-ZVT4-2426-
SM-5GIEI
Skin - Not Sun Exposed (Suprapubic)   Skin   GTEX-ZVTK-0226-
SM-51MRG
Skin - Not Sun Exposed (Suprapubic)   Skin   GTEX-ZVZP-1226-
SM-5GID7
Skin - Not Sun Exposed (Suprapubic)   Skin   GTEX-ZWKS-0626-
SM-5GIDV
Skin - Not Sun Exposed (Suprapubic)   Skin   GTEX-ZXG5-2126-
SM-59HKD
Skin - Not Sun Exposed (Suprapubic)   Skin   GTEX-ZY6K-1426-
SM-5GZX2
Skin - Not Sun Exposed (Suprapubic)   Skin   GTEX-ZYFC-0426-
SM-5GICQ
Skin - Not Sun Exposed (Suprapubic)   Skin   GTEX-ZYFD-0626-
SM-5E44E
Skin - Not Sun Exposed (Suprapubic)   Skin   GTEX-ZYFG-1526-
SM-5GZYM
Skin - Not Sun Exposed (Suprapubic)   Skin   GTEX-ZYT6-2326-
SM-5GZY3
Skin - Not Sun Exposed (Suprapubic)   Skin   GTEX-ZYVF-0926-
SM-5E44J
Skin - Not Sun Exposed (Suprapubic)   Skin   GTEX-ZYW4-0626-
SM-59HJR
Skin - Not Sun Exposed (Suprapubic)   Skin   GTEX-ZYY3-2926-
SM-5GIEB
Skin - Not Sun Exposed (Suprapubic)   Skin   GTEX-ZZ64-1026-
SM-5GZXG
Skin - Not Sun Exposed (Suprapubic)   Skin   GTEX-ZZPU-0826-

SM-5GZX5

Skin - Sun Exposed (Lower leg)	Skin	GTEX-111FC-0126-SM-5N9DL
Skin - Sun Exposed (Lower leg)	Skin	GTEX-111VG-2426-SM-5GZXD
Skin - Sun Exposed (Lower leg)	Skin	GTEX-1122O-2126-SM-5EGIR
Skin - Sun Exposed (Lower leg)	Skin	GTEX-1128S-2326-SM-5GZZY
Skin - Sun Exposed (Lower leg)	Skin	GTEX-113IC-0126-SM-5HL6T
Skin - Sun Exposed (Lower leg)	Skin	GTEX-113JC-2326-SM-5EQ4E
Skin - Sun Exposed (Lower leg)	Skin	GTEX-117XS-2726-SM-5N9BL
Skin - Sun Exposed (Lower leg)	Skin	GTEX-117YW-2626-SM-5GZZH
Skin - Sun Exposed (Lower leg)	Skin	GTEX-117YX-2326-SM-5H12W
Skin - Sun Exposed (Lower leg)	Skin	GTEX-1192W-2626-SM-5Q5AF
Skin - Sun Exposed (Lower leg)	Skin	GTEX-1192X-0226-SM-5H12D
Skin - Sun Exposed (Lower leg)	Skin	GTEX-11DXW-0226-SM-5H122
Skin - Sun Exposed (Lower leg)	Skin	GTEX-11DXX-2426-SM-5GZZW
Skin - Sun Exposed (Lower leg)	Skin	GTEX-11DXY-2626-SM-5GIE7
Skin - Sun Exposed (Lower leg)	Skin	GTEX-11DXZ-2326-SM-5EGGV
Skin - Sun Exposed (Lower leg)	Skin	GTEX-11DYG-0126-SM-59883
Skin - Sun Exposed (Lower leg)	Skin	GTEX-11DZ1-0126-SM-5985Q
Skin - Sun Exposed (Lower leg)	Skin	GTEX-11EI6-0126-SM-5985R
Skin - Sun Exposed (Lower leg)	Skin	GTEX-11EM3-2426-SM-59861
Skin - Sun Exposed (Lower leg)	Skin	GTEX-11EMC-2926-SM-5EQ4F
Skin - Sun Exposed (Lower leg)	Skin	GTEX-11EQ9-2426-SM-5HL5T
Skin - Sun Exposed (Lower leg)	Skin	GTEX-11GS4-2726-SM-5A5LE
Skin - Sun Exposed (Lower leg)	Skin	GTEX-11GSO-2426-SM-5A5LY
Skin - Sun Exposed (Lower leg)	Skin	GTEX-11GSP-2526-SM-5N9BP
Skin - Sun Exposed (Lower leg)	Skin	GTEX-11H98-0126-SM-5EGHG
Skin - Sun Exposed (Lower leg)	Skin	GTEX-11LCK-1026-SM-5A5KB
Skin - Sun Exposed (Lower leg)	Skin	GTEX-11NSD-2226-SM-5986V
Skin - Sun Exposed (Lower leg)	Skin	GTEX-11NUK-0126-SM-5A5L3
Skin - Sun Exposed (Lower leg)	Skin	GTEX-11O72-0126-SM-59887
Skin - Sun Exposed (Lower leg)	Skin	GTEX-11OC5-0126-SM-5HL6A
Skin - Sun Exposed (Lower leg)	Skin	GTEX-11OF3-2626-SM-5GU7F
Skin - Sun Exposed (Lower leg)	Skin	GTEX-11P7K-2126-SM-5GU7G
Skin - Sun Exposed (Lower leg)	Skin	GTEX-11P81-2326-SM-59875
Skin - Sun Exposed (Lower leg)	Skin	GTEX-11P82-1626-SM-59879
Skin - Sun Exposed (Lower leg)	Skin	GTEX-11TT1-2526-SM-5EGIB
Skin - Sun Exposed (Lower leg)	Skin	GTEX-11TTK-0126-SM-5987B
Skin - Sun Exposed (Lower leg)	Skin	GTEX-11TUW-2726-SM-5EQLC
Skin - Sun Exposed (Lower leg)	Skin	GTEX-11UD1-0126-SM-5PNYG
Skin - Sun Exposed (Lower leg)	Skin	GTEX-11UD2-0226-SM-5EQKY
Skin - Sun Exposed (Lower leg)	Skin	GTEX-11VI4-1726-SM-5CVLH
Skin - Sun Exposed (Lower leg)	Skin	GTEX-11WQC-2526-SM-5CVLE
Skin - Sun Exposed (Lower leg)	Skin	GTEX-11WQK-0526-SM-5EQLD
Skin - Sun Exposed (Lower leg)	Skin	GTEX-11XUK-2026-SM-5EQLF
Skin - Sun Exposed (Lower leg)	Skin	GTEX-11ZTS-0126-SM-5EQ6M
Skin - Sun Exposed (Lower leg)	Skin	GTEX-11ZTT-2426-SM-5EQLS
Skin - Sun Exposed (Lower leg)	Skin	GTEX-11ZU8-2526-SM-5EQKI
Skin - Sun Exposed (Lower leg)	Skin	GTEX-11ZVC-2526-SM-5FQTE
Skin - Sun Exposed (Lower leg)	Skin	GTEX-1211K-2026-SM-5EQ4Z
Skin - Sun Exposed (Lower leg)	Skin	GTEX-12126-0326-SM-5PNW3
Skin - Sun Exposed (Lower leg)	Skin	GTEX-1212Z-2526-SM-5EGKP
Skin - Sun Exposed (Lower leg)	Skin	GTEX-12584-0126-SM-5EGKQ
Skin - Sun Exposed (Lower leg)	Skin	GTEX-12696-2426-SM-5EQ6H
Skin - Sun Exposed (Lower leg)	Skin	GTEX-1269C-2526-SM-5EGJ3

Skin - Sun Exposed (Lower leg)	Skin	GTEX-12C56-1726-SM-5EQ61
Skin - Sun Exposed (Lower leg)	Skin	GTEX-12KS4-0326-SM-5EQ41
Skin - Sun Exposed (Lower leg)	Skin	GTEX-12WSA-0226-SM-5BC5V
Skin - Sun Exposed (Lower leg)	Skin	GTEX-12WSC-0126-SM-5EQ4P
Skin - Sun Exposed (Lower leg)	Skin	GTEX-12WSD-0126-SM-59HKN
Skin - Sun Exposed (Lower leg)	Skin	GTEX-12WSE-0526-SM-793B2
Skin - Sun Exposed (Lower leg)	Skin	GTEX-12WSG-2826-SM-5FQUS
Skin - Sun Exposed (Lower leg)	Skin	GTEX-12WSJ-1826-SM-5GCNY
Skin - Sun Exposed (Lower leg)	Skin	GTEX-12WSK-2326-SM-5GCOH
Skin - Sun Exposed (Lower leg)	Skin	GTEX-12WSL-2526-SM-5GCNJ
Skin - Sun Exposed (Lower leg)	Skin	GTEX-12WSN-5019-SM-79OM6
Skin - Sun Exposed (Lower leg)	Skin	GTEX-12ZZX-0126-SM-5EGK9
Skin - Sun Exposed (Lower leg)	Skin	GTEX-12ZZY-0226-SM-5LZVE
Skin - Sun Exposed (Lower leg)	Skin	GTEX-12ZZZ-0626-SM-5DUXI
Skin - Sun Exposed (Lower leg)	Skin	GTEX-13111-1626-SM-5EGJY
Skin - Sun Exposed (Lower leg)	Skin	GTEX-13112-2826-SM-5DUWH
Skin - Sun Exposed (Lower leg)	Skin	GTEX-13113-5016-SM-7EPIP
Skin - Sun Exposed (Lower leg)	Skin	GTEX-1313W-0126-SM-5LZUN
Skin - Sun Exposed (Lower leg)	Skin	GTEX-1314G-1526-SM-5EGK2
Skin - Sun Exposed (Lower leg)	Skin	GTEX-131XE-2326-SM-5PNZ2
Skin - Sun Exposed (Lower leg)	Skin	GTEX-131XF-2126-SM-5DUWS
Skin - Sun Exposed (Lower leg)	Skin	GTEX-131XG-2626-SM-5KM17
Skin - Sun Exposed (Lower leg)	Skin	GTEX-131XH-2226-SM-5DUXJ
Skin - Sun Exposed (Lower leg)	Skin	GTEX-131XW-0426-SM-5LZWP
Skin - Sun Exposed (Lower leg)	Skin	GTEX-131YS-0126-SM-5KM1M
Skin - Sun Exposed (Lower leg)	Skin	GTEX-132NY-0326-SM-5IJBL
Skin - Sun Exposed (Lower leg)	Skin	GTEX-133LE-1826-SM-5J1MV
Skin - Sun Exposed (Lower leg)	Skin	GTEX-1399R-2726-SM-5IJBA
Skin - Sun Exposed (Lower leg)	Skin	GTEX-1399T-2826-SM-5KLYZ
Skin - Sun Exposed (Lower leg)	Skin	GTEX-1399U-2326-SM-5K7WY
Skin - Sun Exposed (Lower leg)	Skin	GTEX-139D8-0226-SM-5KLZO
Skin - Sun Exposed (Lower leg)	Skin	GTEX-139T4-0226-SM-5HL5S
Skin - Sun Exposed (Lower leg)	Skin	GTEX-139T8-0126-SM-5K7XZ
Skin - Sun Exposed (Lower leg)	Skin	GTEX-139TS-2826-SM-5J1OO
Skin - Sun Exposed (Lower leg)	Skin	GTEX-139TT-0126-SM-5K7Y5
Skin - Sun Exposed (Lower leg)	Skin	GTEX-139TU-0126-SM-5K7WH
Skin - Sun Exposed (Lower leg)	Skin	GTEX-139UW-2826-SM-5L3E7
Skin - Sun Exposed (Lower leg)	Skin	GTEX-13CF2-2526-SM-5LZYX
Skin - Sun Exposed (Lower leg)	Skin	GTEX-13CF3-2426-SM-5IFGX
Skin - Sun Exposed (Lower leg)	Skin	GTEX-13D11-2426-SM-5KM1V
Skin - Sun Exposed (Lower leg)	Skin	GTEX-13FH7-2026-SM-5IJCR
Skin - Sun Exposed (Lower leg)	Skin	GTEX-13FHO-0226-SM-5IFGY
Skin - Sun Exposed (Lower leg)	Skin	GTEX-13FHP-0126-SM-5IJBG
Skin - Sun Exposed (Lower leg)	Skin	GTEX-13FTW-2626-SM-5K7WL
Skin - Sun Exposed (Lower leg)	Skin	GTEX-13FTX-1826-SM-5J1NX
Skin - Sun Exposed (Lower leg)	Skin	GTEX-13FTY-0526-SM-5L3EV
Skin - Sun Exposed (Lower leg)	Skin	GTEX-13FXS-0226-SM-5IJCK
Skin - Sun Exposed (Lower leg)	Skin	GTEX-13G51-2526-SM-5LZYK
Skin - Sun Exposed (Lower leg)	Skin	GTEX-13IVO-0126-SM-5J1NG
Skin - Sun Exposed (Lower leg)	Skin	GTEX-13JUV-2726-SM-5LZZ8
Skin - Sun Exposed (Lower leg)	Skin	GTEX-13N11-2526-SM-5K7UE
Skin - Sun Exposed (Lower leg)	Skin	GTEX-13N1W-0126-SM-5K7VT
Skin - Sun Exposed (Lower leg)	Skin	GTEX-13N2G-2626-SM-5J1OT
Skin - Sun Exposed (Lower leg)	Skin	GTEX-13NYB-2526-SM-5K7Z6

Skin - Sun Exposed (Lower leg)	Skin	GTEX-13NYC-0326-SM-5K7WP
Skin - Sun Exposed (Lower leg)	Skin	GTEX-13NYS-0126-SM-5MR3W
Skin - Sun Exposed (Lower leg)	Skin	GTEX-13NZ8-0826-SM-5MR3I
Skin - Sun Exposed (Lower leg)	Skin	GTEX-13NZA-0226-SM-5K7Z7
Skin - Sun Exposed (Lower leg)	Skin	GTEX-13NZB-2526-SM-5J1OM
Skin - Sun Exposed (Lower leg)	Skin	GTEX-13O1R-0126-SM-5L3EH
Skin - Sun Exposed (Lower leg)	Skin	GTEX-13O21-1426-SM-5K7XI
Skin - Sun Exposed (Lower leg)	Skin	GTEX-13O3O-0126-SM-5KM4E
Skin - Sun Exposed (Lower leg)	Skin	GTEX-13O3P-0126-SM-5KM4S
Skin - Sun Exposed (Lower leg)	Skin	GTEX-13O3Q-2426-SM-5IJBR
Skin - Sun Exposed (Lower leg)	Skin	GTEX-13OVG-1926-SM-5L3DK
Skin - Sun Exposed (Lower leg)	Skin	GTEX-13OVH-0126-SM-5MR4B
Skin - Sun Exposed (Lower leg)	Skin	GTEX-13OVI-1426-SM-5L3EY
Skin - Sun Exposed (Lower leg)	Skin	GTEX-13OVJ-0126-SM-5KM36
Skin - Sun Exposed (Lower leg)	Skin	GTEX-13OVK-2026-SM-7KUEO
Skin - Sun Exposed (Lower leg)	Skin	GTEX-13OVL-0226-SM-5L3E9
Skin - Sun Exposed (Lower leg)	Skin	GTEX-13OW5-0126-SM-5MR3A
Skin - Sun Exposed (Lower leg)	Skin	GTEX-13OW6-0426-SM-5L3HM
Skin - Sun Exposed (Lower leg)	Skin	GTEX-13OW7-0126-SM-5MR3B
Skin - Sun Exposed (Lower leg)	Skin	GTEX-13OW8-1326-SM-5J1OW
Skin - Sun Exposed (Lower leg)	Skin	GTEX-13PDP-0126-SM-5L3HD
Skin - Sun Exposed (Lower leg)	Skin	GTEX-13PVQ-0126-SM-5SIB4
Skin - Sun Exposed (Lower leg)	Skin	GTEX-13PVR-2326-SM-5QGR1
Skin - Sun Exposed (Lower leg)	Skin	GTEX-13QJ3-0326-SM-5S2RA
Skin - Sun Exposed (Lower leg)	Skin	GTEX-13RTJ-2426-SM-5S2Q2
Skin - Sun Exposed (Lower leg)	Skin	GTEX-13RTK-1426-SM-5QGQJ
Skin - Sun Exposed (Lower leg)	Skin	GTEX-13RTL-0126-SM-7LG5S
Skin - Sun Exposed (Lower leg)	Skin	GTEX-13S7M-0126-SM-5SI6A
Skin - Sun Exposed (Lower leg)	Skin	GTEX-13SLW-0126-SM-5SI6C
Skin - Sun Exposed (Lower leg)	Skin	GTEX-13SLX-0526-SM-5S2ON
Skin - Sun Exposed (Lower leg)	Skin	GTEX-13U4I-0126-SM-5LU38
Skin - Sun Exposed (Lower leg)	Skin	GTEX-13VXT-0126-SM-5LU4I
Skin - Sun Exposed (Lower leg)	Skin	GTEX-13VXU-0126-SM-5SI9F
Skin - Sun Exposed (Lower leg)	Skin	GTEX-13W3W-2426-SM-5LU5B
Skin - Sun Exposed (Lower leg)	Skin	GTEX-13W46-0226-SM-5SIAG
Skin - Sun Exposed (Lower leg)	Skin	GTEX-13X6H-2126-SM-7EWD5
Skin - Sun Exposed (Lower leg)	Skin	GTEX-13X6I-0626-SM-5QGP9
Skin - Sun Exposed (Lower leg)	Skin	GTEX-13X6J-0126-SM-5QGOU
Skin - Sun Exposed (Lower leg)	Skin	GTEX-13YAN-0326-SM-5O9DF
Skin - Sun Exposed (Lower leg)	Skin	GTEX-144FL-0126-SM-5TDC8
Skin - Sun Exposed (Lower leg)	Skin	GTEX-144GL-0126-SM-5TDE2
Skin - Sun Exposed (Lower leg)	Skin	GTEX-144GN-2326-SM-5LU4F
Skin - Sun Exposed (Lower leg)	Skin	GTEX-145LS-0126-SM-5QGP1
Skin - Sun Exposed (Lower leg)	Skin	GTEX-145LT-1726-SM-5QGP3
Skin - Sun Exposed (Lower leg)	Skin	GTEX-145LU-0126-SM-5TDCB
Skin - Sun Exposed (Lower leg)	Skin	GTEX-145LV-2126-SM-5LU8N
Skin - Sun Exposed (Lower leg)	Skin	GTEX-145ME-1826-SM-5SI9G
Skin - Sun Exposed (Lower leg)	Skin	GTEX-145MF-0626-SM-5LUAI
Skin - Sun Exposed (Lower leg)	Skin	GTEX-145MI-0526-SM-5O9A8
Skin - Sun Exposed (Lower leg)	Skin	GTEX-145MN-2226-SM-5SIAU
Skin - Sun Exposed (Lower leg)	Skin	GTEX-145MO-0326-SM-5Q5B3
Skin - Sun Exposed (Lower leg)	Skin	GTEX-146FH-0126-SM-5QGPP
Skin - Sun Exposed (Lower leg)	Skin	GTEX-146FQ-0126-SM-5NQA9
Skin - Sun Exposed (Lower leg)	Skin	GTEX-146FR-1326-SM-5SIAJ

Skin - Sun Exposed (Lower leg)	Skin	GTEX-14753-0426-SM-5NQAM
Skin - Sun Exposed (Lower leg)	Skin	GTEX-147F3-0526-SM-5SI98
Skin - Sun Exposed (Lower leg)	Skin	GTEX-147F4-0226-SM-5QGPW
Skin - Sun Exposed (Lower leg)	Skin	GTEX-147GR-0526-SM-5S2MY
Skin - Sun Exposed (Lower leg)	Skin	GTEX-148VI-1626-SM-5SI6E
Skin - Sun Exposed (Lower leg)	Skin	GTEX-148VJ-0126-SM-5TDDN
Skin - Sun Exposed (Lower leg)	Skin	GTEX-1497J-2426-SM-7IGNX
Skin - Sun Exposed (Lower leg)	Skin	GTEX-14A5H-0426-SM-5QGQF
Skin - Sun Exposed (Lower leg)	Skin	GTEX-14A5I-0126-SM-5TDEA
Skin - Sun Exposed (Lower leg)	Skin	GTEX-14A6H-0126-SM-5QGPK
Skin - Sun Exposed (Lower leg)	Skin	GTEX-14ABY-0226-SM-5TDCQ
Skin - Sun Exposed (Lower leg)	Skin	GTEX-14BIL-0126-SM-5SI92
Skin - Sun Exposed (Lower leg)	Skin	GTEX-14BIM-0226-SM-7IGLD
Skin - Sun Exposed (Lower leg)	Skin	GTEX-14BIN-0626-SM-793DP
Skin - Sun Exposed (Lower leg)	Skin	GTEX-14BMU-2226-SM-5TDE9
Skin - Sun Exposed (Lower leg)	Skin	GTEX-14BMV-0126-SM-5S2PO
Skin - Sun Exposed (Lower leg)	Skin	GTEX-14C5O-0126-SM-5SI6N
Skin - Sun Exposed (Lower leg)	Skin	GTEX-14DAQ-0126-SM-5S2MQ
Skin - Sun Exposed (Lower leg)	Skin	GTEX-14DAR-2026-SM-5S2O3
Skin - Sun Exposed (Lower leg)	Skin	GTEX-14E6C-0126-SM-5RQIP
Skin - Sun Exposed (Lower leg)	Skin	GTEX-14E6D-0126-SM-62LDV
Skin - Sun Exposed (Lower leg)	Skin	GTEX-14E6E-1826-SM-5S2R7
Skin - Sun Exposed (Lower leg)	Skin	GTEX-14E7W-0126-SM-6LLJ9
Skin - Sun Exposed (Lower leg)	Skin	GTEX-14ICK-0226-SM-6EU25
Skin - Sun Exposed (Lower leg)	Skin	GTEX-14JG1-0426-SM-6ETZZ
Skin - Sun Exposed (Lower leg)	Skin	GTEX-14JG6-2226-SM-6EU2G
Skin - Sun Exposed (Lower leg)	Skin	GTEX-14LLW-0126-SM-6LLIO
Skin - Sun Exposed (Lower leg)	Skin	GTEX-14LZ3-0226-SM-62LET
Skin - Sun Exposed (Lower leg)	Skin	GTEX-14PHW-0126-SM-6LLJI
Skin - Sun Exposed (Lower leg)	Skin	GTEX-14PHX-2326-SM-62LEM
Skin - Sun Exposed (Lower leg)	Skin	GTEX-14PII-0226-SM-6EU1T
Skin - Sun Exposed (Lower leg)	Skin	GTEX-14PJ3-1826-SM-62LEX
Skin - Sun Exposed (Lower leg)	Skin	GTEX-14PJ6-2126-SM-6EU1V
Skin - Sun Exposed (Lower leg)	Skin	GTEX-14PJM-0226-SM-62LF1
Skin - Sun Exposed (Lower leg)	Skin	GTEX-14PK6-1926-SM-6ETZU
Skin - Sun Exposed (Lower leg)	Skin	GTEX-14PN3-2426-SM-6LLHS
Skin - Sun Exposed (Lower leg)	Skin	GTEX-14PN4-0126-SM-6EU2V
Skin - Sun Exposed (Lower leg)	Skin	GTEX-14XAO-2326-SM-6EU1B
Skin - Sun Exposed (Lower leg)	Skin	GTEX-15CHC-2126-SM-6EU21
Skin - Sun Exposed (Lower leg)	Skin	GTEX-15D1Q-0126-SM-7KUG6
Skin - Sun Exposed (Lower leg)	Skin	GTEX-15DCD-0126-SM-7KUEK
Skin - Sun Exposed (Lower leg)	Skin	GTEX-15DDE-0126-SM-6LPKH
Skin - Sun Exposed (Lower leg)	Skin	GTEX-15DYW-0126-SM-7KULE
Skin - Sun Exposed (Lower leg)	Skin	GTEX-15EOM-0126-SM-7KUGG
Skin - Sun Exposed (Lower leg)	Skin	GTEX-15ER7-0326-SM-7KUEV
Skin - Sun Exposed (Lower leg)	Skin	GTEX-15ETS-0126-SM-7KUMW
Skin - Sun Exposed (Lower leg)	Skin	GTEX-15EU6-0426-SM-6PAN5
Skin - Sun Exposed (Lower leg)	Skin	GTEX-15F5U-1526-SM-6PANO
Skin - Sun Exposed (Lower leg)	Skin	GTEX-15G19-0126-SM-6PANQ
Skin - Sun Exposed (Lower leg)	Skin	GTEX-15RIF-1926-SM-7KUDW
Skin - Sun Exposed (Lower leg)	Skin	GTEX-15RIG-0226-SM-7KFTL
Skin - Sun Exposed (Lower leg)	Skin	GTEX-15SB6-1726-SM-6PAME
Skin - Sun Exposed (Lower leg)	Skin	GTEX-15SDE-2226-SM-7IGLO
Skin - Sun Exposed (Lower leg)	Skin	GTEX-15SHU-0126-SM-7KUEH

Skin - Sun Exposed (Lower leg)	Skin	GTEX-15SHV-2426-SM-6PAMF
Skin - Sun Exposed (Lower leg)	Skin	GTEX-15SHW-0326-SM-6PAML
Skin - Sun Exposed (Lower leg)	Skin	GTEX-15UF6-0826-SM-6PALJ
Skin - Sun Exposed (Lower leg)	Skin	GTEX-15UKP-0426-SM-7KUFQ
Skin - Sun Exposed (Lower leg)	Skin	GTEX-16AAH-1726-SM-7939C
Skin - Sun Exposed (Lower leg)	Skin	GTEX-16BQI-0226-SM-7KUEX
Skin - Sun Exposed (Lower leg)	Skin	GTEX-16GPK-0126-SM-7DHLE
Skin - Sun Exposed (Lower leg)	Skin	GTEX-16MT8-0426-SM-7KUEY
Skin - Sun Exposed (Lower leg)	Skin	GTEX-16NGA-1726-SM-731B4
Skin - Sun Exposed (Lower leg)	Skin	GTEX-16NPX-0226-SM-79OMT
Skin - Sun Exposed (Lower leg)	Skin	GTEX-16XZY-2126-SM-79OK1
Skin - Sun Exposed (Lower leg)	Skin	GTEX-16XZZ-0126-SM-793DV
Skin - Sun Exposed (Lower leg)	Skin	GTEX-16YQH-0126-SM-7DUFJ
Skin - Sun Exposed (Lower leg)	Skin	GTEX-17EUY-0426-SM-793BD
Skin - Sun Exposed (Lower leg)	Skin	GTEX-17EVP-0326-SM-7DHLG
Skin - Sun Exposed (Lower leg)	Skin	GTEX-17EVQ-0226-SM-7LG4P
Skin - Sun Exposed (Lower leg)	Skin	GTEX-17F96-1626-SM-7DUFL
Skin - Sun Exposed (Lower leg)	Skin	GTEX-17F97-0326-SM-7LG5B
Skin - Sun Exposed (Lower leg)	Skin	GTEX-17F98-0626-SM-79OMX
Skin - Sun Exposed (Lower leg)	Skin	GTEX-17GQL-2026-SM-718BA
Skin - Sun Exposed (Lower leg)	Skin	GTEX-17HG3-2226-SM-7938L
Skin - Sun Exposed (Lower leg)	Skin	GTEX-17HGU-0226-SM-7DHKV
Skin - Sun Exposed (Lower leg)	Skin	GTEX-17HHE-2026-SM-79OKF
Skin - Sun Exposed (Lower leg)	Skin	GTEX-17HHY-0126-SM-7LG5D
Skin - Sun Exposed (Lower leg)	Skin	GTEX-17HII-0226-SM-7LG4E
Skin - Sun Exposed (Lower leg)	Skin	GTEX-17JCI-0126-SM-7LG5G
Skin - Sun Exposed (Lower leg)	Skin	GTEX-17KNJ-2326-SM-7DHLQ
Skin - Sun Exposed (Lower leg)	Skin	GTEX-17MF6-0126-SM-7LG4F
Skin - Sun Exposed (Lower leg)	Skin	GTEX-17MFQ-1726-SM-793C7
Skin - Sun Exposed (Lower leg)	Skin	GTEX-183FY-0126-SM-7LT9P
Skin - Sun Exposed (Lower leg)	Skin	GTEX-183WM-0126-SM-7LTAB
Skin - Sun Exposed (Lower leg)	Skin	GTEX-18465-0126-SM-731BN
Skin - Sun Exposed (Lower leg)	Skin	GTEX-18A66-0226-SM-718BD
Skin - Sun Exposed (Lower leg)	Skin	GTEX-18A67-0126-SM-7LG5P
Skin - Sun Exposed (Lower leg)	Skin	GTEX-18A6Q-0126-SM-731BZ
Skin - Sun Exposed (Lower leg)	Skin	GTEX-18A7A-0226-SM-718BH
Skin - Sun Exposed (Lower leg)	Skin	GTEX-18A7B-0126-SM-7LG64
Skin - Sun Exposed (Lower leg)	Skin	GTEX-18D9A-1926-SM-7LG6P
Skin - Sun Exposed (Lower leg)	Skin	GTEX-18D9B-0526-SM-7LTAS
Skin - Sun Exposed (Lower leg)	Skin	GTEX-18D9U-0226-SM-718BP
Skin - Sun Exposed (Lower leg)	Skin	GTEX-18QFQ-0126-SM-731BE
Skin - Sun Exposed (Lower leg)	Skin	GTEX-1A32A-0126-SM-718B1
Skin - Sun Exposed (Lower leg)	Skin	GTEX-1A3MV-1926-SM-718BT
Skin - Sun Exposed (Lower leg)	Skin	GTEX-1A3MW-0126-SM-73KXX
Skin - Sun Exposed (Lower leg)	Skin	GTEX-1A3MX-0126-SM-731BR
Skin - Sun Exposed (Lower leg)	Skin	GTEX-1AMEY-1326-SM-73KYK
Skin - Sun Exposed (Lower leg)	Skin	GTEX-1AMFI-2026-SM-731BH
Skin - Sun Exposed (Lower leg)	Skin	GTEX-1AX8Z-0126-SM-7DHM6
Skin - Sun Exposed (Lower leg)	Skin	GTEX-1AX9J-0226-SM-73KXM
Skin - Sun Exposed (Lower leg)	Skin	GTEX-1AX9K-2326-SM-73KWU
Skin - Sun Exposed (Lower leg)	Skin	GTEX-1AYCT-2426-SM-7IGQ3
Skin - Sun Exposed (Lower leg)	Skin	GTEX-1AYD5-2426-SM-73KXR
Skin - Sun Exposed (Lower leg)	Skin	GTEX-1B8KE-2126-SM-73KYX
Skin - Sun Exposed (Lower leg)	Skin	GTEX-1B8KZ-2026-SM-7DHMB

Skin - Sun Exposed (Lower leg)	Skin	GTEX-1B8SF-1426-SM-7DUGL
Skin - Sun Exposed (Lower leg)	Skin	GTEX-1B8SG-0126-SM-73KZ3
Skin - Sun Exposed (Lower leg)	Skin	GTEX-1B97I-1626-SM-73KYO
Skin - Sun Exposed (Lower leg)	Skin	GTEX-1BAJH-0526-SM-793CQ
Skin - Sun Exposed (Lower leg)	Skin	GTEX-1C2JI-1826-SM-73KXC
Skin - Sun Exposed (Lower leg)	Skin	GTEX-1C475-1826-SM-73KWA
Skin - Sun Exposed (Lower leg)	Skin	GTEX-1C4CL-0526-SM-73KVK
Skin - Sun Exposed (Lower leg)	Skin	GTEX-1C64N-0126-SM-793D7
Skin - Sun Exposed (Lower leg)	Skin	GTEX-1C6VR-2426-SM-79OLD
Skin - Sun Exposed (Lower leg)	Skin	GTEX-1C6VS-0526-SM-7DUEN
Skin - Sun Exposed (Lower leg)	Skin	GTEX-1CAMQ-0226-SM-7IGPK
Skin - Sun Exposed (Lower leg)	Skin	GTEX-1CAMR-1926-SM-7EPI2
Skin - Sun Exposed (Lower leg)	Skin	GTEX-1CB4E-2226-SM-793DC
Skin - Sun Exposed (Lower leg)	Skin	GTEX-1CB4I-0226-SM-79OLH
Skin - Sun Exposed (Lower leg)	Skin	GTEX-1CB4J-0626-SM-7DHMP
Skin - Sun Exposed (Lower leg)	Skin	GTEX-1E1VI-0126-SM-7DHMZ
Skin - Sun Exposed (Lower leg)	Skin	GTEX-NFK9-0226-SM-2HMKQ
Skin - Sun Exposed (Lower leg)	Skin	GTEX-NPJ8-0126-SM-2YUNR
Skin - Sun Exposed (Lower leg)	Skin	GTEX-O5YT-0126-SM-48TBW
Skin - Sun Exposed (Lower leg)	Skin	GTEX-O5YW-0126-SM-3LK6D
Skin - Sun Exposed (Lower leg)	Skin	GTEX-OHPL-0126-SM-2HMJ7
Skin - Sun Exposed (Lower leg)	Skin	GTEX-OHPM-0126-SM-2YUN9
Skin - Sun Exposed (Lower leg)	Skin	GTEX-OIZG-0726-SM-33HBL
Skin - Sun Exposed (Lower leg)	Skin	GTEX-OIZH-0126-SM-2HMIS
Skin - Sun Exposed (Lower leg)	Skin	GTEX-OOBK-0126-SM-2YUND
Skin - Sun Exposed (Lower leg)	Skin	GTEX-OXRK-0226-SM-3NB2G
Skin - Sun Exposed (Lower leg)	Skin	GTEX-OXRL-0126-SM-2YUMP
Skin - Sun Exposed (Lower leg)	Skin	GTEX-OXRN-0126-SM-48TDM
Skin - Sun Exposed (Lower leg)	Skin	GTEX-OXRO-0126-SM-2YUN4
Skin - Sun Exposed (Lower leg)	Skin	GTEX-OXRP-0126-SM-3NB32
Skin - Sun Exposed (Lower leg)	Skin	GTEX-P44H-0226-SM-2XCEU
Skin - Sun Exposed (Lower leg)	Skin	GTEX-P4PP-0126-SM-3LK69
Skin - Sun Exposed (Lower leg)	Skin	GTEX-P4PQ-0126-SM-2S1NM
Skin - Sun Exposed (Lower leg)	Skin	GTEX-P4QT-0126-SM-2I3FL
Skin - Sun Exposed (Lower leg)	Skin	GTEX-PLZ4-0126-SM-5SI8O
Skin - Sun Exposed (Lower leg)	Skin	GTEX-PLZ5-2026-SM-2S1O4
Skin - Sun Exposed (Lower leg)	Skin	GTEX-PLZ6-1426-SM-5S2TR
Skin - Sun Exposed (Lower leg)	Skin	GTEX-POMQ-2226-SM-2I5FK
Skin - Sun Exposed (Lower leg)	Skin	GTEX-PSDG-0226-SM-33HC1
Skin - Sun Exposed (Lower leg)	Skin	GTEX-PVOW-0126-SM-2XCFA
Skin - Sun Exposed (Lower leg)	Skin	GTEX-PWCY-1826-SM-5SI7U
Skin - Sun Exposed (Lower leg)	Skin	GTEX-PWO3-1526-SM-48TCM
Skin - Sun Exposed (Lower leg)	Skin	GTEX-PX3G-0126-SM-2I3EN
Skin - Sun Exposed (Lower leg)	Skin	GTEX-Q2AG-0126-SM-33HBV
Skin - Sun Exposed (Lower leg)	Skin	GTEX-Q2AH-1626-SM-3GAF8
Skin - Sun Exposed (Lower leg)	Skin	GTEX-Q2AI-1326-SM-2S1PL
Skin - Sun Exposed (Lower leg)	Skin	GTEX-Q734-1926-SM-2I5EV
Skin - Sun Exposed (Lower leg)	Skin	GTEX-QCQG-1726-SM-3GIJ9
Skin - Sun Exposed (Lower leg)	Skin	GTEX-QDT8-0126-SM-48TZ1
Skin - Sun Exposed (Lower leg)	Skin	GTEX-QDVJ-1726-SM-2I5FX
Skin - Sun Exposed (Lower leg)	Skin	GTEX-QDVN-2026-SM-3GAEP
Skin - Sun Exposed (Lower leg)	Skin	GTEX-QEG4-0226-SM-2S1PY
Skin - Sun Exposed (Lower leg)	Skin	GTEX-QESD-1426-SM-2S1R9
Skin - Sun Exposed (Lower leg)	Skin	GTEX-QLQ7-1626-SM-2S1R8

Skin - Sun Exposed (Lower leg)	Skin	GTEX-QLQW-1126-SM-2S1Q8
Skin - Sun Exposed (Lower leg)	Skin	GTEX-QV44-1926-SM-2S1RF
Skin - Sun Exposed (Lower leg)	Skin	GTEX-QVJO-1626-SM-2S1QW
Skin - Sun Exposed (Lower leg)	Skin	GTEX-R3RS-0126-SM-3GIJL
Skin - Sun Exposed (Lower leg)	Skin	GTEX-R55E-0126-SM-2TC5Y
Skin - Sun Exposed (Lower leg)	Skin	GTEX-R55G-2526-SM-2TC6D
Skin - Sun Exposed (Lower leg)	Skin	GTEX-RM2N-1426-SM-2TF4H
Skin - Sun Exposed (Lower leg)	Skin	GTEX-RN64-0126-SM-2TC68
Skin - Sun Exposed (Lower leg)	Skin	GTEX-RNOR-0126-SM-2TF57
Skin - Sun Exposed (Lower leg)	Skin	GTEX-RTLS-0126-SM-447CA
Skin - Sun Exposed (Lower leg)	Skin	GTEX-RU72-0926-SM-2TF6B
Skin - Sun Exposed (Lower leg)	Skin	GTEX-RUSQ-1526-SM-2TF62
Skin - Sun Exposed (Lower leg)	Skin	GTEX-RWS6-2026-SM-2XCB5
Skin - Sun Exposed (Lower leg)	Skin	GTEX-RWSA-0126-SM-2XCBB
Skin - Sun Exposed (Lower leg)	Skin	GTEX-S32W-2126-SM-2XCB1
Skin - Sun Exposed (Lower leg)	Skin	GTEX-S7PM-0126-SM-4AD6S
Skin - Sun Exposed (Lower leg)	Skin	GTEX-S7SE-0126-SM-2XCD5
Skin - Sun Exposed (Lower leg)	Skin	GTEX-S95S-1226-SM-4GICG
Skin - Sun Exposed (Lower leg)	Skin	GTEX-SIU8-0126-SM-2XCDT
Skin - Sun Exposed (Lower leg)	Skin	GTEX-SJXC-0126-SM-2XCFF
Skin - Sun Exposed (Lower leg)	Skin	GTEX-SN8G-0126-SM-32PLI
Skin - Sun Exposed (Lower leg)	Skin	GTEX-SNMC-1226-SM-2XCFP
Skin - Sun Exposed (Lower leg)	Skin	GTEX-SSA3-0126-SM-32QPU
Skin - Sun Exposed (Lower leg)	Skin	GTEX-T2IS-0126-SM-4DM6O
Skin - Sun Exposed (Lower leg)	Skin	GTEX-T2YK-0526-SM-32QPJ
Skin - Sun Exposed (Lower leg)	Skin	GTEX-T5JC-0426-SM-32PLO
Skin - Sun Exposed (Lower leg)	Skin	GTEX-T5JW-1626-SM-3GADZ
Skin - Sun Exposed (Lower leg)	Skin	GTEX-T6MN-0126-SM-32PLP
Skin - Sun Exposed (Lower leg)	Skin	GTEX-T6MO-1626-SM-32QOM
Skin - Sun Exposed (Lower leg)	Skin	GTEX-T8EM-1026-SM-3DB7M
Skin - Sun Exposed (Lower leg)	Skin	GTEX-TKQ1-1026-SM-4GICL
Skin - Sun Exposed (Lower leg)	Skin	GTEX-TML8-1926-SM-32QOS
Skin - Sun Exposed (Lower leg)	Skin	GTEX-TMMY-0226-SM-33HBA
Skin - Sun Exposed (Lower leg)	Skin	GTEX-TMZS-0126-SM-3DB9Q
Skin - Sun Exposed (Lower leg)	Skin	GTEX-TSE9-0126-SM-3DB83
Skin - Sun Exposed (Lower leg)	Skin	GTEX-U3ZH-2026-SM-3DB78
Skin - Sun Exposed (Lower leg)	Skin	GTEX-U3ZM-1526-SM-3DB9D
Skin - Sun Exposed (Lower leg)	Skin	GTEX-U3ZN-2326-SM-3DB7W
Skin - Sun Exposed (Lower leg)	Skin	GTEX-U412-0426-SM-3DB9O
Skin - Sun Exposed (Lower leg)	Skin	GTEX-U8T8-0126-SM-3DB94
Skin - Sun Exposed (Lower leg)	Skin	GTEX-U8XE-0326-SM-3DB8P
Skin - Sun Exposed (Lower leg)	Skin	GTEX-UJHI-1526-SM-3DB99
Skin - Sun Exposed (Lower leg)	Skin	GTEX-UPJH-0326-SM-3GADU
Skin - Sun Exposed (Lower leg)	Skin	GTEX-V1D1-2226-SM-3NMAX
Skin - Sun Exposed (Lower leg)	Skin	GTEX-VJWN-0126-SM-3GIK7
Skin - Sun Exposed (Lower leg)	Skin	GTEX-VJYA-1126-SM-3GIJU
Skin - Sun Exposed (Lower leg)	Skin	GTEX-VUSG-2526-SM-4KL1V
Skin - Sun Exposed (Lower leg)	Skin	GTEX-W5WG-1826-SM-4KL2Y
Skin - Sun Exposed (Lower leg)	Skin	GTEX-WFG8-2226-SM-3GIL9
Skin - Sun Exposed (Lower leg)	Skin	GTEX-WFON-2126-SM-3LK7O
Skin - Sun Exposed (Lower leg)	Skin	GTEX-WH7G-2326-SM-3NMBC
Skin - Sun Exposed (Lower leg)	Skin	GTEX-WI4N-1026-SM-3LK7N
Skin - Sun Exposed (Lower leg)	Skin	GTEX-WOFL-0126-SM-3MJG2
Skin - Sun Exposed (Lower leg)	Skin	GTEX-WQUQ-0426-SM-3MJFU

Skin - Sun Exposed (Lower leg)   Skin   GTEX-WRHK-1426-SM-3MJF9
Skin - Sun Exposed (Lower leg)   Skin   GTEX-WVJS-0126-SM-4MVOT
Skin - Sun Exposed (Lower leg)   Skin   GTEX-WXYG-2326-SM-4E3I6
Skin - Sun Exposed (Lower leg)   Skin   GTEX-WY7C-2326-SM-3NB2U
Skin - Sun Exposed (Lower leg)   Skin   GTEX-WYJK-0126-SM-3NMAB
Skin - Sun Exposed (Lower leg)   Skin   GTEX-WYVS-2126-SM-3NMA3
Skin - Sun Exposed (Lower leg)   Skin   GTEX-WZTO-0126-SM-3NM95
Skin - Sun Exposed (Lower leg)   Skin   GTEX-X261-0126-SM-3NMD6
Skin - Sun Exposed (Lower leg)   Skin   GTEX-X4EO-0326-SM-3P5YO
Skin - Sun Exposed (Lower leg)   Skin   GTEX-X4EP-0126-SM-3P5YV
Skin - Sun Exposed (Lower leg)   Skin   GTEX-X4XX-0126-SM-3NMC2
Skin - Sun Exposed (Lower leg)   Skin   GTEX-X4XY-0226-SM-4E3IZ
Skin - Sun Exposed (Lower leg)   Skin   GTEX-X585-0226-SM-4QAS2
Skin - Sun Exposed (Lower leg)   Skin   GTEX-X5EB-2226-SM-46MW4
Skin - Sun Exposed (Lower leg)   Skin   GTEX-X62O-0126-SM-4E3JN
Skin - Sun Exposed (Lower leg)   Skin   GTEX-X638-0126-SM-47JZ8
Skin - Sun Exposed (Lower leg)   Skin   GTEX-X88G-0126-SM-47JZ3
Skin - Sun Exposed (Lower leg)   Skin   GTEX-X8HC-0126-SM-4E3JW
Skin - Sun Exposed (Lower leg)   Skin   GTEX-XAJ8-0826-SM-47JY6
Skin - Sun Exposed (Lower leg)   Skin   GTEX-XBED-2226-SM-47JYQ
Skin - Sun Exposed (Lower leg)   Skin   GTEX-XBEW-0626-SM-4QASP
Skin - Sun Exposed (Lower leg)   Skin   GTEX-XMD2-0126-SM-4YCDU
Skin - Sun Exposed (Lower leg)   Skin   GTEX-XPVG-2626-SM-4B669
Skin - Sun Exposed (Lower leg)   Skin   GTEX-XQ3S-1526-SM-4BOOC
Skin - Sun Exposed (Lower leg)   Skin   GTEX-XQ8I-0426-SM-4BOPO
Skin - Sun Exposed (Lower leg)   Skin   GTEX-XUW1-0426-SM-4BOOT
Skin - Sun Exposed (Lower leg)   Skin   GTEX-XUYS-0126-SM-47JWZ
Skin - Sun Exposed (Lower leg)   Skin   GTEX-XUZC-1726-SM-4BRWS
Skin - Sun Exposed (Lower leg)   Skin   GTEX-XV7Q-2526-SM-4BRV9
Skin - Sun Exposed (Lower leg)   Skin   GTEX-XXEK-2226-SM-4BRUM
Skin - Sun Exposed (Lower leg)   Skin   GTEX-XYKS-2126-SM-4E3IB
Skin - Sun Exposed (Lower leg)   Skin   GTEX-Y111-0126-SM-4SOIV
Skin - Sun Exposed (Lower leg)   Skin   GTEX-Y114-2426-SM-4TT8A
Skin - Sun Exposed (Lower leg)   Skin   GTEX-Y3I4-2126-SM-4TT7C
Skin - Sun Exposed (Lower leg)   Skin   GTEX-Y3IK-2426-SM-4WWDU
Skin - Sun Exposed (Lower leg)   Skin   GTEX-Y5LM-1926-SM-5RQJH
Skin - Sun Exposed (Lower leg)   Skin   GTEX-Y5V5-2326-SM-4V6GA
Skin - Sun Exposed (Lower leg)   Skin   GTEX-Y5V6-2426-SM-4VDSB
Skin - Sun Exposed (Lower leg)   Skin   GTEX-Y8DK-0126-SM-4TT3L
Skin - Sun Exposed (Lower leg)   Skin   GTEX-Y8E4-0626-SM-4WWDL
Skin - Sun Exposed (Lower leg)   Skin   GTEX-Y8LW-1826-SM-5S2MV
Skin - Sun Exposed (Lower leg)   Skin   GTEX-YB5E-2026-SM-5IFIS
Skin - Sun Exposed (Lower leg)   Skin   GTEX-YB5K-2126-SM-4WWDJ
Skin - Sun Exposed (Lower leg)   Skin   GTEX-YEC4-2126-SM-5IFJH
Skin - Sun Exposed (Lower leg)   Skin   GTEX-YF7O-2326-101833-
SM-5CVN9
Skin - Sun Exposed (Lower leg)   Skin   GTEX-YFC4-0126-SM-5CVLY
Skin - Sun Exposed (Lower leg)   Skin   GTEX-YJ89-0126-SM-4TT3X
Skin - Sun Exposed (Lower leg)   Skin   GTEX-YJ8A-0426-SM-5IFID
Skin - Sun Exposed (Lower leg)   Skin   GTEX-Z93S-0126-SM-5HL7M
Skin - Sun Exposed (Lower leg)   Skin   GTEX-Z93T-0126-SM-5HL5N
Skin - Sun Exposed (Lower leg)   Skin   GTEX-Z9EW-1626-SM-5CVMN
Skin - Sun Exposed (Lower leg)   Skin   GTEX-ZAB4-0226-SM-5N9F5
Skin - Sun Exposed (Lower leg)   Skin   GTEX-ZAB5-1826-SM-5HL7C

Skin - Sun Exposed (Lower leg)   Skin   GTEX-ZAJG-0126-SM-5HL94
Skin - Sun Exposed (Lower leg)   Skin   GTEX-ZAK1-0126-SM-5IJD3
Skin - Sun Exposed (Lower leg)   Skin   GTEX-ZC5H-0226-SM-4WAY9
Skin - Sun Exposed (Lower leg)   Skin   GTEX-ZDTS-0126-SM-4WAY5
Skin - Sun Exposed (Lower leg)   Skin   GTEX-ZDTT-2726-SM-4WKF8
Skin - Sun Exposed (Lower leg)   Skin   GTEX-ZDXO-3026-SM-5J1N5
Skin - Sun Exposed (Lower leg)   Skin   GTEX-ZDYS-2026-SM-5HL5L
Skin - Sun Exposed (Lower leg)   Skin   GTEX-ZE7O-0126-SM-57WC1
Skin - Sun Exposed (Lower leg)   Skin   GTEX-ZE9C-2726-SM-57WB5
Skin - Sun Exposed (Lower leg)   Skin   GTEX-ZEX8-2426-SM-4WKG4
Skin - Sun Exposed (Lower leg)   Skin   GTEX-ZF28-0126-SM-4WKGK
Skin - Sun Exposed (Lower leg)   Skin   GTEX-ZF2S-2326-SM-4WWCE
Skin - Sun Exposed (Lower leg)   Skin   GTEX-ZGAY-0526-SM-4WWBB
Skin - Sun Exposed (Lower leg)   Skin   GTEX-ZP4G-2426-SM-57WEY
Skin - Sun Exposed (Lower leg)   Skin   GTEX-ZPIC-1726-SM-57WF2
Skin - Sun Exposed (Lower leg)   Skin   GTEX-ZQG8-1626-SM-5HL6H
Skin - Sun Exposed (Lower leg)   Skin   GTEX-ZQUD-1426-SM-57WER
Skin - Sun Exposed (Lower leg)   Skin   GTEX-ZTPG-0326-SM-5O9AX
Skin - Sun Exposed (Lower leg)   Skin   GTEX-ZTSS-1926-SM-57WEW
Skin - Sun Exposed (Lower leg)   Skin   GTEX-ZTX8-1426-SM-5DUVO
Skin - Sun Exposed (Lower leg)   Skin   GTEX-ZU9S-0226-SM-5E441
Skin - Sun Exposed (Lower leg)   Skin   GTEX-ZUA1-0126-SM-5GU76
Skin - Sun Exposed (Lower leg)   Skin   GTEX-ZV68-0126-SM-59HKS
Skin - Sun Exposed (Lower leg)   Skin   GTEX-ZV6S-2326-SM-4YCEY
Skin - Sun Exposed (Lower leg)   Skin   GTEX-ZVE2-0226-SM-59HJB
Skin - Sun Exposed (Lower leg)   Skin   GTEX-ZVT2-2526-SM-51MT1
Skin - Sun Exposed (Lower leg)   Skin   GTEX-ZVT3-0126-SM-5GU6I
Skin - Sun Exposed (Lower leg)   Skin   GTEX-ZVT4-0126-SM-51MS1
Skin - Sun Exposed (Lower leg)   Skin   GTEX-ZVTK-0426-SM-51MRS
Skin - Sun Exposed (Lower leg)   Skin   GTEX-ZVZO-0126-SM-5A5L9
Skin - Sun Exposed (Lower leg)   Skin   GTEX-ZVZP-2426-SM-59HKJ
Skin - Sun Exposed (Lower leg)   Skin   GTEX-ZWKS-0126-SM-5SIAA
Skin - Sun Exposed (Lower leg)   Skin   GTEX-ZXES-1826-SM-5E43S
Skin - Sun Exposed (Lower leg)   Skin   GTEX-ZXG5-0126-SM-5GIEU
Skin - Sun Exposed (Lower leg)   Skin   GTEX-ZY6K-1826-SM-5GZXK
Skin - Sun Exposed (Lower leg)   Skin   GTEX-ZYFC-0226-SM-5NQ75
Skin - Sun Exposed (Lower leg)   Skin   GTEX-ZYFD-0126-SM-5GIDL
Skin - Sun Exposed (Lower leg)   Skin   GTEX-ZYFG-2326-SM-5E44B
Skin - Sun Exposed (Lower leg)   Skin   GTEX-ZYT6-0226-SM-5NQ6T
Skin - Sun Exposed (Lower leg)   Skin   GTEX-ZYW4-0126-SM-5E44A
Skin - Sun Exposed (Lower leg)   Skin   GTEX-ZYY3-0126-SM-5GZY5
Skin - Sun Exposed (Lower leg)   Skin   GTEX-ZZ64-1726-SM-5GZYB
Skin - Sun Exposed (Lower leg)   Skin   GTEX-ZZPT-0226-SM-5E43X
Small Intestine - Terminal Ileum Small Intestine GTEX-111CU-1326-SM-5NQ8L
Small Intestine - Terminal Ileum Small Intestine GTEX-111YS-1426-SM-5GID8
Small Intestine - Terminal Ileum Small Intestine GTEX-1122O-1326-SM-5H11F
Small Intestine - Terminal Ileum Small Intestine GTEX-117YX-0326-SM-5GICL
Small Intestine - Terminal Ileum Small Intestine GTEX-1192X-2526-SM-59868
Small Intestine - Terminal Ileum Small Intestine GTEX-11DXX-1626-

SM-5H11H
Small Intestine - Terminal Ileum Small Intestine GTEX-11DXZ-1326-
SM-5H11X
Small Intestine - Terminal Ileum Small Intestine GTEX-11EQ9-1326-
SM-5985X
Small Intestine - Terminal Ileum Small Intestine GTEX-11I78-1126-
SM-5A5K5
Small Intestine - Terminal Ileum Small Intestine GTEX-11LCK-1926-
SM-5A5KE
Small Intestine - Terminal Ileum Small Intestine GTEX-11NSD-1726-
SM-5N9C3
Small Intestine - Terminal Ileum Small Intestine GTEX-11P7K-1626-
SM-5GU63
Small Intestine - Terminal Ileum Small Intestine GTEX-11P82-0826-
SM-5P9GU
Small Intestine - Terminal Ileum Small Intestine GTEX-11VI4-1626-
SM-5EQKO
Small Intestine - Terminal Ileum Small Intestine GTEX-11XUK-1426-
SM-5EQMG
Small Intestine - Terminal Ileum Small Intestine GTEX-12BJ1-1926-
SM-5HL9Z
Small Intestine - Terminal Ileum Small Intestine GTEX-12WSK-1526-
SM-5CVNO
Small Intestine - Terminal Ileum Small Intestine GTEX-13111-1426-
SM-5DUW3
Small Intestine - Terminal Ileum Small Intestine GTEX-13113-1226-
SM-5EGHT
Small Intestine - Terminal Ileum Small Intestine GTEX-131XE-0926-
SM-5HL7J
Small Intestine - Terminal Ileum Small Intestine GTEX-131XF-1326-
SM-5GCMQ
Small Intestine - Terminal Ileum Small Intestine GTEX-131XG-1726-
SM-5LZUR
Small Intestine - Terminal Ileum Small Intestine GTEX-133LE-1426-
SM-5IFH1
Small Intestine - Terminal Ileum Small Intestine GTEX-1399R-2026-
SM-5K7WN
Small Intestine - Terminal Ileum Small Intestine GTEX-1399S-2026-
SM-5KM4B
Small Intestine - Terminal Ileum Small Intestine GTEX-1399U-2126-
SM-5IFEZ
Small Intestine - Terminal Ileum Small Intestine GTEX-139T6-1026-
SM-5IJGC
Small Intestine - Terminal Ileum Small Intestine GTEX-139YR-2226-
SM-5IFFW
Small Intestine - Terminal Ileum Small Intestine GTEX-13CF3-1226-
SM-5LZXN
Small Intestine - Terminal Ileum Small Intestine GTEX-13D11-0726-
SM-5LZZB
Small Intestine - Terminal Ileum Small Intestine GTEX-13FH7-1226-
SM-5IJFK
Small Intestine - Terminal Ileum Small Intestine GTEX-13FTW-2026-
SM-5IJE2
Small Intestine - Terminal Ileum Small Intestine GTEX-13NYB-1826-

SM-5J1N3
Small Intestine - Terminal Ileum Small Intestine GTEX-13O21-1026-SM-5IJDU
Small Intestine - Terminal Ileum Small Intestine GTEX-13O61-1326-SM-5KM31
Small Intestine - Terminal Ileum Small Intestine GTEX-13OVI-1926-SM-5IJCG
Small Intestine - Terminal Ileum Small Intestine GTEX-13PL7-2626-SM-5IFH4
Small Intestine - Terminal Ileum Small Intestine GTEX-13QBU-1926-SM-5IJEW
Small Intestine - Terminal Ileum Small Intestine GTEX-13RTK-0826-SM-5Q5C6
Small Intestine - Terminal Ileum Small Intestine GTEX-13W3W-2026-SM-5K7UL
Small Intestine - Terminal Ileum Small Intestine GTEX-13X6H-1826-SM-7EWCZ
Small Intestine - Terminal Ileum Small Intestine GTEX-144GM-1026-SM-79OKU
Small Intestine - Terminal Ileum Small Intestine GTEX-145LT-1026-SM-5LU9O
Small Intestine - Terminal Ileum Small Intestine GTEX-145ME-0926-SM-5O9AR
Small Intestine - Terminal Ileum Small Intestine GTEX-148VJ-2226-SM-5NQ9M
Small Intestine - Terminal Ileum Small Intestine GTEX-1497J-1926-SM-5TDCC
Small Intestine - Terminal Ileum Small Intestine GTEX-14BMU-1426-SM-5TDE5
Small Intestine - Terminal Ileum Small Intestine GTEX-14C39-1826-SM-5ZZW4
Small Intestine - Terminal Ileum Small Intestine GTEX-14DAR-1426-SM-5RQIR
Small Intestine - Terminal Ileum Small Intestine GTEX-14E6E-1626-SM-5RQI7
Small Intestine - Terminal Ileum Small Intestine GTEX-14PJ3-1326-SM-664O7
Small Intestine - Terminal Ileum Small Intestine GTEX-14PJ6-1726-SM-6AJBN
Small Intestine - Terminal Ileum Small Intestine GTEX-14PJN-0826-SM-686ZL
Small Intestine - Terminal Ileum Small Intestine GTEX-14PK6-1626-SM-6871I
Small Intestine - Terminal Ileum Small Intestine GTEX-14PKU-1626-SM-6871C
Small Intestine - Terminal Ileum Small Intestine GTEX-14PKV-2026-SM-6AJ9A
Small Intestine - Terminal Ileum Small Intestine GTEX-14PN3-2026-SM-68729
Small Intestine - Terminal Ileum Small Intestine GTEX-14XAO-1926-SM-6ETZO
Small Intestine - Terminal Ileum Small Intestine GTEX-15DZA-0826-SM-6AJBF
Small Intestine - Terminal Ileum Small Intestine GTEX-15G1A-1726-

SM-6LPI4
Small Intestine - Terminal Ileum Small Intestine GTEX-16A39-1426-
SM-6LLI6
Small Intestine - Terminal Ileum Small Intestine GTEX-16AAH-1426-
SM-7KFTR
Small Intestine - Terminal Ileum Small Intestine GTEX-16MTA-1526-
SM-6LPJB
Small Intestine - Terminal Ileum Small Intestine GTEX-16NGA-1126-
SM-7KFSY
Small Intestine - Terminal Ileum Small Intestine GTEX-17HHE-1726-
SM-79OKT
Small Intestine - Terminal Ileum Small Intestine GTEX-17KNJ-1626-
SM-718AL
Small Intestine - Terminal Ileum Small Intestine GTEX-17MFQ-1026-
SM-7KFSZ
Small Intestine - Terminal Ileum Small Intestine GTEX-183FY-1626-
SM-793D8
Small Intestine - Terminal Ileum Small Intestine GTEX-18A66-2526-
SM-7KFTZ
Small Intestine - Terminal Ileum Small Intestine GTEX-18QFQ-1626-
SM-72D7H
Small Intestine - Terminal Ileum Small Intestine GTEX-1AMFI-1926-
SM-731F2
Small Intestine - Terminal Ileum Small Intestine GTEX-1AX8Z-2526-
SM-731E3
Small Intestine - Terminal Ileum Small Intestine GTEX-1B8KE-1726-
SM-7IGMN
Small Intestine - Terminal Ileum Small Intestine GTEX-1B8KZ-1126-
SM-7IGLX
Small Intestine - Terminal Ileum Small Intestine GTEX-1BAJH-2526-
SM-7IGOT
Small Intestine - Terminal Ileum Small Intestine GTEX-1C475-1426-
SM-793CH
Small Intestine - Terminal Ileum Small Intestine GTEX-1C64O-2526-
SM-79ONO
Small Intestine - Terminal Ileum Small Intestine GTEX-1CAMR-0826-
SM-7MKFK
Small Intestine - Terminal Ileum Small Intestine GTEX-1EMGI-2826-
SM-7IGMR
Small Intestine - Terminal Ileum Small Intestine GTEX-1GL5R-2026-
SM-7MKHP
Small Intestine - Terminal Ileum Small Intestine GTEX-1GMR2-1026-
SM-7MKHJ
Small Intestine - Terminal Ileum Small Intestine GTEX-1GMR3-2726-
SM-7MKFB
Small Intestine - Terminal Ileum Small Intestine GTEX-UPK5-2026-
SM-4JBIM
Small Intestine - Terminal Ileum Small Intestine GTEX-VJYA-0926-
SM-4KL1N
Small Intestine - Terminal Ileum Small Intestine GTEX-VUSG-1726-
SM-4KKZL
Small Intestine - Terminal Ileum Small Intestine GTEX-WEY5-1526-
SM-4LMJF
Small Intestine - Terminal Ileum Small Intestine GTEX-WFG7-1726-

SM-4LVME
Small Intestine - Terminal Ileum Small Intestine GTEX-WFJO-0926-SM-4LVM2
Small Intestine - Terminal Ileum Small Intestine GTEX-WFON-1526-SM-4LVMP
Small Intestine - Terminal Ileum Small Intestine GTEX-WH7G-1626-SM-4LVMY
Small Intestine - Terminal Ileum Small Intestine GTEX-WHSB-1026-SM-4M1XN
Small Intestine - Terminal Ileum Small Intestine GTEX-WQUQ-2426-SM-4MVNW
Small Intestine - Terminal Ileum Small Intestine GTEX-WRHK-1026-SM-4MVOD
Small Intestine - Terminal Ileum Small Intestine GTEX-WY7C-1826-SM-4ONCE
Small Intestine - Terminal Ileum Small Intestine GTEX-WYJK-1526-SM-4ONCW
Small Intestine - Terminal Ileum Small Intestine GTEX-X15G-1426-SM-4PQZK
Small Intestine - Terminal Ileum Small Intestine GTEX-X5EB-1626-SM-4E3IV
Small Intestine - Terminal Ileum Small Intestine GTEX-XAJ8-0526-SM-47JYK
Small Intestine - Terminal Ileum Small Intestine GTEX-XBED-1426-SM-4AT4G
Small Intestine - Terminal Ileum Small Intestine GTEX-XGQ4-1326-SM-4GIDU
Small Intestine - Terminal Ileum Small Intestine GTEX-XMK1-1826-SM-4B66F
Small Intestine - Terminal Ileum Small Intestine GTEX-XPVG-1726-SM-4B65W
Small Intestine - Terminal Ileum Small Intestine GTEX-XUZC-1426-SM-4BRV3
Small Intestine - Terminal Ileum Small Intestine GTEX-XXEK-1026-SM-4BRUW
Small Intestine - Terminal Ileum Small Intestine GTEX-Y111-2226-SM-4SOJB
Small Intestine - Terminal Ileum Small Intestine GTEX-Y114-1426-SM-4TT6W
Small Intestine - Terminal Ileum Small Intestine GTEX-Y3I4-1326-SM-4TT8Z
Small Intestine - Terminal Ileum Small Intestine GTEX-Y3IK-1726-SM-4YCCR
Small Intestine - Terminal Ileum Small Intestine GTEX-Y5V5-1826-SM-4VDS5
Small Intestine - Terminal Ileum Small Intestine GTEX-Y9LG-1626-SM-5IFHM
Small Intestine - Terminal Ileum Small Intestine GTEX-YB5E-1526-SM-5IFIE
Small Intestine - Terminal Ileum Small Intestine GTEX-YEC4-1726-SM-4W21E
Small Intestine - Terminal Ileum Small Intestine GTEX-YF7O-1826-SM-5IFIU
Small Intestine - Terminal Ileum Small Intestine GTEX-YFCO-1326-

SM-664NV
Small Intestine - Terminal Ileum Small Intestine GTEX-Z9EW-0926-
SM-5CVMM
Small Intestine - Terminal Ileum Small Intestine GTEX-ZA64-1226-
SM-5HL7B
Small Intestine - Terminal Ileum Small Intestine GTEX-ZAB5-1526-
SM-5HL9P
Small Intestine - Terminal Ileum Small Intestine GTEX-ZC5H-2326-
SM-4WAYT
Small Intestine - Terminal Ileum Small Intestine GTEX-ZEX8-1826-
SM-57WBH
Small Intestine - Terminal Ileum Small Intestine GTEX-ZF29-1626-
SM-4WKFJ
Small Intestine - Terminal Ileum Small Intestine GTEX-ZF2S-1726-
SM-57WFT
Small Intestine - Terminal Ileum Small Intestine GTEX-ZLFU-1526-
SM-4WWBT
Small Intestine - Terminal Ileum Small Intestine GTEX-ZLV1-1126-
SM-4WWBW
Small Intestine - Terminal Ileum Small Intestine GTEX-ZP4G-1626-
SM-57WGD
Small Intestine - Terminal Ileum Small Intestine GTEX-ZPCL-1726-
SM-57WEO
Small Intestine - Terminal Ileum Small Intestine GTEX-ZPIC-2326-
SM-57WDW
Small Intestine - Terminal Ileum Small Intestine GTEX-ZQG8-2526-
SM-57WEQ
Small Intestine - Terminal Ileum Small Intestine GTEX-ZQUD-0926-
SM-57WE3
Small Intestine - Terminal Ileum Small Intestine GTEX-ZTPG-2626-
SM-57WFX
Small Intestine - Terminal Ileum Small Intestine GTEX-ZTSS-0926-
SM-59865
Small Intestine - Terminal Ileum Small Intestine GTEX-ZTX8-0526-
SM-59HLD
Small Intestine - Terminal Ileum Small Intestine GTEX-ZVP2-1726-
SM-5GU5P
Small Intestine - Terminal Ileum Small Intestine GTEX-ZVT2-2126-
SM-5NQ93
Small Intestine - Terminal Ileum Small Intestine GTEX-ZVZP-1826-
SM-5GZXB
Small Intestine - Terminal Ileum Small Intestine GTEX-ZXES-1526-
SM-5NQ95
Small Intestine - Terminal Ileum Small Intestine GTEX-ZYFG-2026-
SM-5E43Y
Small Intestine - Terminal Ileum Small Intestine GTEX-ZZ64-0526-
SM-5GZXM
Spleen Spleen GTEX-111CU-0426-SM-5GZY1
Spleen Spleen GTEX-111FC-1326-SM-5N9D9
Spleen Spleen GTEX-1122O-0526-SM-5N9DM
Spleen Spleen GTEX-117YX-2126-SM-5GIEL
Spleen Spleen GTEX-11DXX-0726-SM-5H12X
Spleen Spleen GTEX-11EQ9-0426-SM-5A5JY
Spleen Spleen GTEX-11LCK-0126-SM-5A5M5

Spleen	Spleen	GTEX-11NSD-0426-SM-5N9CR
Spleen	Spleen	GTEX-11P7K-0426-SM-5BC5H
Spleen	Spleen	GTEX-11P81-1326-SM-5GU5S
Spleen	Spleen	GTEX-11VI4-0126-SM-5GU6Z
Spleen	Spleen	GTEX-11XUK-0426-SM-5EQML
Spleen	Spleen	GTEX-1211K-0426-SM-5FQTP
Spleen	Spleen	GTEX-12BJ1-0726-SM-5EGGF
Spleen	Spleen	GTEX-12WSD-1726-SM-5GCN4
Spleen	Spleen	GTEX-12WSG-0926-SM-5EGIH
Spleen	Spleen	GTEX-12WSJ-0526-SM-5GCOZ
Spleen	Spleen	GTEX-12WSK-0126-SM-5GCO7
Spleen	Spleen	GTEX-12WSN-0126-SM-5DUX5
Spleen	Spleen	GTEX-13111-0126-SM-5EGHO
Spleen	Spleen	GTEX-131XE-1126-SM-5EGHW
Spleen	Spleen	GTEX-131XG-0426-SM-5IFGD
Spleen	Spleen	GTEX-132QS-0226-SM-5IFFZ
Spleen	Spleen	GTEX-1399R-0226-SM-5IJGG
Spleen	Spleen	GTEX-1399S-0226-SM-5IFFG
Spleen	Spleen	GTEX-139T6-0226-SM-5IFGZ
Spleen	Spleen	GTEX-139YR-0426-SM-5IFFV
Spleen	Spleen	GTEX-13CF3-0226-SM-5J1NW
Spleen	Spleen	GTEX-13FH7-1326-SM-5IFG8
Spleen	Spleen	GTEX-13N11-0126-SM-5KM2Z
Spleen	Spleen	GTEX-13OVK-0426-SM-7KFT8
Spleen	Spleen	GTEX-13PL7-1126-SM-5MR5O
Spleen	Spleen	GTEX-13PVR-0326-SM-5RQJY
Spleen	Spleen	GTEX-13S86-0126-SM-5S2PI
Spleen	Spleen	GTEX-13U4I-1626-SM-5IFFR
Spleen	Spleen	GTEX-13VXT-1326-SM-5LU3Y
Spleen	Spleen	GTEX-13X6H-0726-SM-5Q5BX
Spleen	Spleen	GTEX-144GN-2126-SM-5O99D
Spleen	Spleen	GTEX-145MN-0126-SM-5RQHY
Spleen	Spleen	GTEX-146FQ-0626-SM-5LU9U
Spleen	Spleen	GTEX-146FR-1926-SM-5NQA6
Spleen	Spleen	GTEX-147F3-0126-SM-5NQBJ
Spleen	Spleen	GTEX-14BMU-0426-SM-73KYT
Spleen	Spleen	GTEX-14DAR-0326-SM-5S2PS
Spleen	Spleen	GTEX-14E1K-0126-SM-664NL
Spleen	Spleen	GTEX-14E6E-1226-SM-5S2R5
Spleen	Spleen	GTEX-14ICL-0126-SM-664NG
Spleen	Spleen	GTEX-14JG6-0226-SM-6872A
Spleen	Spleen	GTEX-14PJ4-0526-SM-6871G
Spleen	Spleen	GTEX-14PJ5-0326-SM-664OD
Spleen	Spleen	GTEX-14PJ6-0226-SM-6AJ9K
Spleen	Spleen	GTEX-14PK6-0526-SM-69LOF
Spleen	Spleen	GTEX-14PKU-0526-SM-6871A
Spleen	Spleen	GTEX-14PN3-0226-SM-6871K
Spleen	Spleen	GTEX-15CHC-0726-SM-6AJ9D
Spleen	Spleen	GTEX-15CHQ-1226-SM-6ETZL
Spleen	Spleen	GTEX-15DYW-1426-SM-6LPIW
Spleen	Spleen	GTEX-15DZA-0126-SM-6PAMV
Spleen	Spleen	GTEX-15ER7-1526-SM-6LLHO
Spleen	Spleen	GTEX-15ETS-3126-SM-6LPKN
Spleen	Spleen	GTEX-15F5U-0126-SM-6LPKA

Spleen	Spleen	GTEX-15RIF-0526-SM-6LLI2
Spleen	Spleen	GTEX-15RIG-0126-SM-6PAN4
Spleen	Spleen	GTEX-15RJ7-0126-SM-6LPJN
Spleen	Spleen	GTEX-15SB6-0126-SM-6LPJR
Spleen	Spleen	GTEX-15SDE-0126-SM-793BA
Spleen	Spleen	GTEX-15SHV-0126-SM-6LPIS
Spleen	Spleen	GTEX-15UF7-1926-SM-6LPJT
Spleen	Spleen	GTEX-169BO-0126-SM-79OLS
Spleen	Spleen	GTEX-16MT8-1826-SM-7EWE8
Spleen	Spleen	GTEX-16XZY-0126-SM-7IGQ9
Spleen	Spleen	GTEX-17F96-1426-SM-7IGQA
Spleen	Spleen	GTEX-17F9E-1126-SM-79OMO
Spleen	Spleen	GTEX-17GQL-0126-SM-72D5Q
Spleen	Spleen	GTEX-17HHE-0326-SM-793BH
Spleen	Spleen	GTEX-18A66-1826-SM-7KFSU
Spleen	Spleen	GTEX-18A67-1526-SM-7LG6F
Spleen	Spleen	GTEX-1A3MV-0126-SM-731FQ
Spleen	Spleen	GTEX-1AMFI-0926-SM-731E4
Spleen	Spleen	GTEX-1AX9K-0226-SM-793CM
Spleen	Spleen	GTEX-1AYD5-0226-SM-79ONV
Spleen	Spleen	GTEX-1B8KZ-0226-SM-7EWF6
Spleen	Spleen	GTEX-1B97I-0126-SM-731DH
Spleen	Spleen	GTEX-1B97J-1926-SM-7IGQB
Spleen	Spleen	GTEX-1C4CL-0626-SM-731DM
Spleen	Spleen	GTEX-1C64O-1626-SM-731EN
Spleen	Spleen	GTEX-1C6VQ-2126-SM-7IGNL
Spleen	Spleen	GTEX-1CB4E-0126-SM-7DHMX
Spleen	Spleen	GTEX-1CB4F-0226-SM-7EPHS
Spleen	Spleen	GTEX-1CB4I-1426-SM-79OLZ
Spleen	Spleen	GTEX-1FIGZ-0126-SM-7MKGZ
Spleen	Spleen	GTEX-O5YT-0926-SM-48TDG
Spleen	Spleen	GTEX-OIZH-0926-SM-48TBR
Spleen	Spleen	GTEX-OOBJ-0926-SM-48TDO
Spleen	Spleen	GTEX-P4PP-0926-SM-48TBZ
Spleen	Spleen	GTEX-P4QS-0926-SM-48TBS
Spleen	Spleen	GTEX-PLZ6-0126-SM-48TC6
Spleen	Spleen	GTEX-POMQ-0126-SM-48TD6
Spleen	Spleen	GTEX-PW2O-0126-SM-48TC8
Spleen	Spleen	GTEX-PX3G-0926-SM-48U12
Spleen	Spleen	GTEX-Q2AH-0226-SM-48U1I
Spleen	Spleen	GTEX-Q734-0226-SM-48U1A
Spleen	Spleen	GTEX-QCQG-0226-SM-48U28
Spleen	Spleen	GTEX-QDVN-0526-SM-48TZ4
Spleen	Spleen	GTEX-QLQW-0126-SM-447BK
Spleen	Spleen	GTEX-QMRM-0226-SM-4R1K7
Spleen	Spleen	GTEX-QV31-0126-SM-447BP
Spleen	Spleen	GTEX-QV44-0126-SM-4R1KH
Spleen	Spleen	GTEX-R55G-0226-SM-48FEI
Spleen	Spleen	GTEX-RUSQ-0126-SM-47JWV
Spleen	Spleen	GTEX-S3XE-0626-SM-4AD6B
Spleen	Spleen	GTEX-S4Z8-0226-SM-4AD5K
Spleen	Spleen	GTEX-T5JW-0126-SM-4DM6K
Spleen	Spleen	GTEX-U3ZH-0126-SM-4DXUK
Spleen	Spleen	GTEX-U3ZN-0126-SM-4DXUM

Spleen	Spleen	GTEX-U4B1-0526-SM-4DXTK
Spleen	Spleen	GTEX-UJHI-0126-SM-4IHLP
Spleen	Spleen	GTEX-UPK5-1626-SM-4JBHI
Spleen	Spleen	GTEX-VJYA-0126-SM-4KL1P
Spleen	Spleen	GTEX-W5WG-0626-SM-4SOID
Spleen	Spleen	GTEX-WFON-0126-SM-4LVM9
Spleen	Spleen	GTEX-WHWD-0126-SM-4OORS
Spleen	Spleen	GTEX-WXYG-0126-SM-4OND7
Spleen	Spleen	GTEX-WY7C-0126-SM-4ONCO
Spleen	Spleen	GTEX-WYJK-0726-SM-4ONC9
Spleen	Spleen	GTEX-WYVS-0126-SM-4ONDH
Spleen	Spleen	GTEX-X3Y1-0126-SM-4PQZA
Spleen	Spleen	GTEX-X4LF-0226-SM-4QAS8
Spleen	Spleen	GTEX-X585-1826-SM-4QAS3
Spleen	Spleen	GTEX-XAJ8-0126-SM-47JYG
Spleen	Spleen	GTEX-XGQ4-0126-SM-4AT4H
Spleen	Spleen	GTEX-XMK1-0126-SM-4B65F
Spleen	Spleen	GTEX-XPVG-0626-SM-4B65B
Spleen	Spleen	GTEX-XQ8I-1726-SM-4BOQB
Spleen	Spleen	GTEX-XV7Q-0126-SM-4BRVK
Spleen	Spleen	GTEX-XXEK-0126-SM-4BRVU
Spleen	Spleen	GTEX-Y114-0726-SM-4TT6U
Spleen	Spleen	GTEX-Y3I4-0126-SM-4TT26
Spleen	Spleen	GTEX-Y3IK-0126-SM-4WWE9
Spleen	Spleen	GTEX-Y5LM-0326-SM-4VBRN
Spleen	Spleen	GTEX-Y8LW-0126-SM-4VBS1
Spleen	Spleen	GTEX-YB5E-0426-SM-5IFHR
Spleen	Spleen	GTEX-YB5K-1926-SM-4VDT4
Spleen	Spleen	GTEX-YEC4-0126-SM-4W21T
Spleen	Spleen	GTEX-YFCO-0926-SM-5LUA5
Spleen	Spleen	GTEX-Z9EW-0726-SM-5HL9H
Spleen	Spleen	GTEX-ZAB5-0526-SM-5CVMU
Spleen	Spleen	GTEX-ZDTT-1026-SM-5KM19
Spleen	Spleen	GTEX-ZDYS-1026-SM-4WKHC
Spleen	Spleen	GTEX-ZEX8-0726-SM-4WKH8
Spleen	Spleen	GTEX-ZF29-0626-SM-4WKHD
Spleen	Spleen	GTEX-ZF2S-0326-SM-4WKGD
Spleen	Spleen	GTEX-ZLFU-0526-SM-4WWBN
Spleen	Spleen	GTEX-ZLWG-0426-SM-4WWFA
Spleen	Spleen	GTEX-ZP4G-0326-SM-4YCEF
Spleen	Spleen	GTEX-ZPU1-1026-SM-4YCEQ
Spleen	Spleen	GTEX-ZTTD-1726-SM-57WEL
Spleen	Spleen	GTEX-ZV6S-0826-SM-5NQ6Z
Spleen	Spleen	GTEX-ZVP2-0526-SM-51MSC
Spleen	Spleen	GTEX-ZVZP-0726-SM-59HKA
Spleen	Spleen	GTEX-ZYFG-0726-SM-5GIDX
Spleen	Spleen	GTEX-ZZPU-0126-SM-5E446
Stomach	Stomach	GTEX-111CU-0926-SM-5EGIK
Stomach	Stomach	GTEX-111YS-1126-SM-5GZYQ
Stomach	Stomach	GTEX-1122O-1926-SM-5EGIQ
Stomach	Stomach	GTEX-117YW-2226-SM-5N9DB
Stomach	Stomach	GTEX-117YX-1026-SM-5H11V
Stomach	Stomach	GTEX-11DXX-1326-SM-5GIDZ
Stomach	Stomach	GTEX-11EI6-2426-SM-5PNVS

Stomach	Stomach	GTEX-11EM3-1026-SM-5A5KL
Stomach	Stomach	GTEX-11EQ9-1226-SM-5987E
Stomach	Stomach	GTEX-11GSP-2126-SM-5HL5E
Stomach	Stomach	GTEX-11I78-1726-SM-5A5M3
Stomach	Stomach	GTEX-11NSD-1426-SM-5HL67
Stomach	Stomach	GTEX-11NUK-2426-SM-5BC4U
Stomach	Stomach	GTEX-11P7K-1726-SM-5GU6F
Stomach	Stomach	GTEX-11P82-0726-SM-5PNYL
Stomach	Stomach	GTEX-11TT1-0726-SM-5GU5A
Stomach	Stomach	GTEX-11VI4-0326-SM-5EQ6L
Stomach	Stomach	GTEX-11WQK-2626-SM-5EQ4K
Stomach	Stomach	GTEX-1211K-1426-SM-5FQTF
Stomach	Stomach	GTEX-12696-1726-SM-5EQLH
Stomach	Stomach	GTEX-12BJ1-1726-SM-5HL9B
Stomach	Stomach	GTEX-12C56-0526-SM-5FQST
Stomach	Stomach	GTEX-12WSD-2326-SM-59HKQ
Stomach	Stomach	GTEX-12WSG-2026-SM-5FQUU
Stomach	Stomach	GTEX-12WSJ-0926-SM-5P9JD
Stomach	Stomach	GTEX-12WSK-1426-SM-5CVNN
Stomach	Stomach	GTEX-13111-1226-SM-5GCNC
Stomach	Stomach	GTEX-13113-0726-SM-5LZUF
Stomach	Stomach	GTEX-131XE-2226-SM-5PNYX
Stomach	Stomach	GTEX-131XF-1226-SM-5HL8V
Stomach	Stomach	GTEX-131XG-1226-SM-5EGH9
Stomach	Stomach	GTEX-132AR-2426-SM-5IFFD
Stomach	Stomach	GTEX-132QS-1826-SM-5IFFN
Stomach	Stomach	GTEX-1339X-1426-SM-5K7YO
Stomach	Stomach	GTEX-133LE-1326-SM-5IFGO
Stomach	Stomach	GTEX-1399R-1126-SM-5IFIO
Stomach	Stomach	GTEX-1399S-1626-SM-5P9GI
Stomach	Stomach	GTEX-1399U-1626-SM-5P9J3
Stomach	Stomach	GTEX-139D8-2326-SM-5IFGE
Stomach	Stomach	GTEX-139YR-1926-SM-5LZXM
Stomach	Stomach	GTEX-13CF3-1926-SM-5K7WF
Stomach	Stomach	GTEX-13D11-2126-SM-5IFH2
Stomach	Stomach	GTEX-13FH7-1026-SM-5IJGF
Stomach	Stomach	GTEX-13FTW-1726-SM-5KM2B
Stomach	Stomach	GTEX-13FTX-0726-SM-5N9BI
Stomach	Stomach	GTEX-13N1W-2026-SM-5K7YU
Stomach	Stomach	GTEX-13O21-0926-SM-5IFGT
Stomach	Stomach	GTEX-13O3Q-2126-SM-5KM4C
Stomach	Stomach	GTEX-13O61-1126-SM-5L3FI
Stomach	Stomach	GTEX-13OVI-2426-SM-5KM4J
Stomach	Stomach	GTEX-13OVK-0926-SM-7KUFX
Stomach	Stomach	GTEX-13OW6-2526-SM-5IJEC
Stomach	Stomach	GTEX-13PVQ-2126-SM-5L3FW
Stomach	Stomach	GTEX-13PVR-2126-SM-73KVR
Stomach	Stomach	GTEX-13QBU-1126-SM-5LU44
Stomach	Stomach	GTEX-13QJ3-2726-SM-5SI6L
Stomach	Stomach	GTEX-13RTK-0226-SM-5RQHR
Stomach	Stomach	GTEX-13S7M-1826-SM-5RQK6
Stomach	Stomach	GTEX-13U4I-2526-SM-5SI8Z
Stomach	Stomach	GTEX-13X6H-1626-SM-5Q5CT
Stomach	Stomach	GTEX-13X6J-2126-SM-5TDCV

Stomach	Stomach	GTEX-144GM-1726-SM-5O9AS
Stomach	Stomach	GTEX-144GN-1226-SM-5O991
Stomach	Stomach	GTEX-145LT-1426-SM-5O9B3
Stomach	Stomach	GTEX-145ME-1026-SM-5O9B4
Stomach	Stomach	GTEX-145MN-1326-SM-5NQ9S
Stomach	Stomach	GTEX-145MO-2226-SM-5Q5BN
Stomach	Stomach	GTEX-146FH-2126-SM-5SI9U
Stomach	Stomach	GTEX-146FR-2026-SM-5NQAI
Stomach	Stomach	GTEX-14B4R-0726-SM-5TDDR
Stomach	Stomach	GTEX-14BIL-1926-SM-73KYI
Stomach	Stomach	GTEX-14BMU-1126-SM-5RQJ8
Stomach	Stomach	GTEX-14BMV-2226-SM-5RQHX
Stomach	Stomach	GTEX-14DAQ-2826-SM-664MU
Stomach	Stomach	GTEX-14DAR-0926-SM-73KUQ
Stomach	Stomach	GTEX-14E1K-1526-SM-664MN
Stomach	Stomach	GTEX-14E6E-0926-SM-664N8
Stomach	Stomach	GTEX-14ICL-0826-SM-5YY9W
Stomach	Stomach	GTEX-14JG6-1226-SM-664O6
Stomach	Stomach	GTEX-14PHX-1026-SM-664OL
Stomach	Stomach	GTEX-14PHY-0926-SM-5ZZWL
Stomach	Stomach	GTEX-14PJ3-1426-SM-664O8
Stomach	Stomach	GTEX-14PJ4-1626-SM-664OB
Stomach	Stomach	GTEX-14PJ5-0826-SM-6AJAR
Stomach	Stomach	GTEX-14PJ6-0826-SM-686Z9
Stomach	Stomach	GTEX-14PKU-1126-SM-69LOU
Stomach	Stomach	GTEX-14PKV-1826-SM-69LO9
Stomach	Stomach	GTEX-14PN3-1326-SM-6AJ9U
Stomach	Stomach	GTEX-15CHR-1326-SM-79OLT
Stomach	Stomach	GTEX-15DCE-1626-SM-6LPJM
Stomach	Stomach	GTEX-15DZA-1126-SM-6LPK8
Stomach	Stomach	GTEX-15EU6-2526-SM-7KUGB
Stomach	Stomach	GTEX-15G19-1526-SM-6LPKP
Stomach	Stomach	GTEX-15G1A-1226-SM-7KUF3
Stomach	Stomach	GTEX-15RIE-1026-SM-6LPJQ
Stomach	Stomach	GTEX-15RIF-1126-SM-7KUG2
Stomach	Stomach	GTEX-15SHW-2126-SM-6M46Y
Stomach	Stomach	GTEX-15UF7-1226-SM-6M46G
Stomach	Stomach	GTEX-16AAH-1226-SM-79OO9
Stomach	Stomach	GTEX-16NGA-1526-SM-72D56
Stomach	Stomach	GTEX-17EUY-1526-SM-793CA
Stomach	Stomach	GTEX-17F98-1526-SM-7EWD8
Stomach	Stomach	GTEX-17F9E-2226-SM-7IGNC
Stomach	Stomach	GTEX-17GQL-1226-SM-7KFTS
Stomach	Stomach	GTEX-17HGU-2126-SM-7IGOV
Stomach	Stomach	GTEX-17HHE-1026-SM-79ON6
Stomach	Stomach	GTEX-17HII-2426-SM-7KFTT
Stomach	Stomach	GTEX-17JCI-2226-SM-7LTA1
Stomach	Stomach	GTEX-17KNJ-1526-SM-7LTA9
Stomach	Stomach	GTEX-17MF6-2126-SM-7KFS7
Stomach	Stomach	GTEX-18A66-2226-SM-7189E
Stomach	Stomach	GTEX-18A67-2626-SM-718AD
Stomach	Stomach	GTEX-18A7A-2726-SM-7LG6U
Stomach	Stomach	GTEX-18D9A-0626-SM-7KFTY
Stomach	Stomach	GTEX-18D9B-2226-SM-718BM

Stomach	Stomach	GTEX-18QFQ-1226-SM-718AE
Stomach	Stomach	GTEX-1A3MV-1226-SM-718BW
Stomach	Stomach	GTEX-1A3MX-1926-SM-72D7F
Stomach	Stomach	GTEX-1A8G6-1426-SM-731EZ
Stomach	Stomach	GTEX-1AMEY-1226-SM-731DU
Stomach	Stomach	GTEX-1AMFI-1226-SM-731CZ
Stomach	Stomach	GTEX-1AX9J-2626-SM-731EK
Stomach	Stomach	GTEX-1AYCT-1326-SM-7MKHB
Stomach	Stomach	GTEX-1B8KE-1326-SM-73KVI
Stomach	Stomach	GTEX-1B8KZ-1226-SM-793CP
Stomach	Stomach	GTEX-1B8L1-1526-SM-79OLK
Stomach	Stomach	GTEX-1B97J-1826-SM-7IGPP
Stomach	Stomach	GTEX-1C475-1026-SM-7MKFJ
Stomach	Stomach	GTEX-1C64O-2226-SM-73KTV
Stomach	Stomach	GTEX-1CAMR-0726-SM-7EPIE
Stomach	Stomach	GTEX-1CB4E-1326-SM-7DUGD
Stomach	Stomach	GTEX-1E2YA-2426-SM-7EPIG
Stomach	Stomach	GTEX-1F5PL-1426-SM-7MKFH
Stomach	Stomach	GTEX-1F75I-1826-SM-7MKGX
Stomach	Stomach	GTEX-NFK9-1526-SM-3LK7B
Stomach	Stomach	GTEX-O5YW-1526-SM-3MJGL
Stomach	Stomach	GTEX-OHPK-1526-SM-3MJGM
Stomach	Stomach	GTEX-OIZH-1526-SM-3NB1J
Stomach	Stomach	GTEX-OOBJ-1526-SM-3NB1Q
Stomach	Stomach	GTEX-OXRK-1626-SM-3NB17
Stomach	Stomach	GTEX-P4PP-1526-SM-3P61M
Stomach	Stomach	GTEX-P4PQ-1526-SM-3NMCK
Stomach	Stomach	GTEX-P4QT-1526-SM-3NMCT
Stomach	Stomach	GTEX-P78B-1826-SM-3P5YX
Stomach	Stomach	GTEX-PLZ6-0826-SM-3P61K
Stomach	Stomach	GTEX-POMQ-0826-SM-3P61H
Stomach	Stomach	GTEX-PW2O-1226-SM-48TCH
Stomach	Stomach	GTEX-PWCY-0926-SM-48TD7
Stomach	Stomach	GTEX-PWN1-1526-SM-48TDA
Stomach	Stomach	GTEX-PX3G-1526-SM-48U11
Stomach	Stomach	GTEX-Q2AH-1126-SM-48TZM
Stomach	Stomach	GTEX-Q2AI-0826-SM-48TZO
Stomach	Stomach	GTEX-Q734-1026-SM-48U16
Stomach	Stomach	GTEX-QCQG-0526-SM-48U2A
Stomach	Stomach	GTEX-QDVJ-1426-SM-48U1Y
Stomach	Stomach	GTEX-QDVN-1226-SM-48TZ5
Stomach	Stomach	GTEX-QLQ7-0826-SM-447B3
Stomach	Stomach	GTEX-QLQW-0726-SM-447AA
Stomach	Stomach	GTEX-QMRM-1126-SM-447BN
Stomach	Stomach	GTEX-QV31-0626-SM-447C5
Stomach	Stomach	GTEX-QV44-1226-SM-4R1KE
Stomach	Stomach	GTEX-QXCU-1926-SM-48FE4
Stomach	Stomach	GTEX-R53T-1226-SM-48FCT
Stomach	Stomach	GTEX-R55C-1026-SM-48FCM
Stomach	Stomach	GTEX-R55D-1526-SM-48FEJ
Stomach	Stomach	GTEX-R55G-1126-SM-48FDG
Stomach	Stomach	GTEX-RM2N-0826-SM-48FD3
Stomach	Stomach	GTEX-RTLS-2626-SM-46MUJ
Stomach	Stomach	GTEX-RU1J-0526-SM-46MUT

Stomach	Stomach	GTEX-RWS6-0926-SM-47JXE
Stomach	Stomach	GTEX-S341-0626-SM-4AD5T
Stomach	Stomach	GTEX-S3XE-1026-SM-4AD4O
Stomach	Stomach	GTEX-S4P3-0726-SM-4AD57
Stomach	Stomach	GTEX-S4Q7-0726-SM-4AD5F
Stomach	Stomach	GTEX-S4UY-1626-SM-4AD55
Stomach	Stomach	GTEX-S4Z8-1226-SM-4AD6W
Stomach	Stomach	GTEX-S7SF-0626-SM-4AD4V
Stomach	Stomach	GTEX-S95S-0826-SM-4B64N
Stomach	Stomach	GTEX-SNMC-0626-SM-4DM6H
Stomach	Stomach	GTEX-SNOS-0826-SM-4DM5N
Stomach	Stomach	GTEX-T5JC-1926-SM-4DM6Q
Stomach	Stomach	GTEX-T5JW-0926-SM-4DM5K
Stomach	Stomach	GTEX-T6MO-0726-SM-4DM58
Stomach	Stomach	GTEX-T8EM-1226-SM-4DM5J
Stomach	Stomach	GTEX-TKQ1-0526-SM-4DXTG
Stomach	Stomach	GTEX-TKQ2-0926-SM-4DXU5
Stomach	Stomach	GTEX-TMMY-1626-SM-4DXTY
Stomach	Stomach	GTEX-U3ZN-2026-SM-4DXUC
Stomach	Stomach	GTEX-U4B1-1026-SM-4DXT1
Stomach	Stomach	GTEX-U8T8-1226-SM-4E3IH
Stomach	Stomach	GTEX-UJHI-1026-SM-4IHJP
Stomach	Stomach	GTEX-UJMC-1226-SM-4IHLI
Stomach	Stomach	GTEX-UPK5-2126-SM-4JBJK
Stomach	Stomach	GTEX-V1D1-1726-SM-4JBHB
Stomach	Stomach	GTEX-V955-1326-SM-4JBHR
Stomach	Stomach	GTEX-VJYA-1026-SM-4KL21
Stomach	Stomach	GTEX-W5WG-1726-SM-4LMI5
Stomach	Stomach	GTEX-WEY5-1226-SM-4LMIQ
Stomach	Stomach	GTEX-WFG7-1326-SM-4LMK1
Stomach	Stomach	GTEX-WFG8-1326-SM-4LVN3
Stomach	Stomach	GTEX-WFJO-1126-SM-4LVLZ
Stomach	Stomach	GTEX-WFON-1126-SM-4LVMA
Stomach	Stomach	GTEX-WH7G-1526-SM-4LVMX
Stomach	Stomach	GTEX-WHPG-0426-SM-4M1XW
Stomach	Stomach	GTEX-WHSB-1226-SM-4M1XR
Stomach	Stomach	GTEX-WHWD-1426-SM-4OORU
Stomach	Stomach	GTEX-WRHK-0626-SM-4MVOE
Stomach	Stomach	GTEX-WXYG-1626-SM-4ONCR
Stomach	Stomach	GTEX-WY7C-1726-SM-4ONCC
Stomach	Stomach	GTEX-WYJK-2526-SM-4ONDF
Stomach	Stomach	GTEX-WYVS-1326-SM-4ONCQ
Stomach	Stomach	GTEX-WZTO-2126-SM-4PQYW
Stomach	Stomach	GTEX-X15G-1126-SM-4PQZG
Stomach	Stomach	GTEX-XAJ8-0326-SM-47JYI
Stomach	Stomach	GTEX-XBED-1226-SM-4AT5V
Stomach	Stomach	GTEX-XBEW-1826-SM-4RTWX
Stomach	Stomach	GTEX-XPVG-1526-SM-4B66C
Stomach	Stomach	GTEX-XQ8I-2026-SM-4BOOL
Stomach	Stomach	GTEX-XUZC-0726-SM-4BOPH
Stomach	Stomach	GTEX-XV7Q-1326-SM-4BRWM
Stomach	Stomach	GTEX-XXEK-0826-SM-4BRWG
Stomach	Stomach	GTEX-Y114-1226-SM-4TT88
Stomach	Stomach	GTEX-Y3I4-1626-SM-4TT7W

Stomach	Stomach	GTEX-Y3IK-1426-SM-4YCES
Stomach	Stomach	GTEX-Y5LM-1326-SM-5RQIS
Stomach	Stomach	GTEX-Y5V6-1326-SM-4VDTF
Stomach	Stomach	GTEX-Y8E4-1526-SM-4WWDI
Stomach	Stomach	GTEX-Y8LW-0826-SM-4WWDO
Stomach	Stomach	GTEX-Y9LG-1026-SM-5IFJN
Stomach	Stomach	GTEX-YB5E-1126-SM-664NU
Stomach	Stomach	GTEX-YEC3-1426-101806-SM-5PNXX
Stomach	Stomach	GTEX-YEC4-1426-SM-5IFHS
Stomach	Stomach	GTEX-YFCO-0826-SM-5LUAG
Stomach	Stomach	GTEX-YJ8A-1526-SM-5P9FT
Stomach	Stomach	GTEX-ZA64-0826-SM-5HL9U
Stomach	Stomach	GTEX-ZAB4-1526-SM-5CVN7
Stomach	Stomach	GTEX-ZDYS-1926-SM-5HL59
Stomach	Stomach	GTEX-ZE7O-3026-SM-51MS4
Stomach	Stomach	GTEX-ZEX8-1626-SM-4WKG7
Stomach	Stomach	GTEX-ZF29-1526-SM-4WKF7
Stomach	Stomach	GTEX-ZF2S-1426-SM-57WET
Stomach	Stomach	GTEX-ZLFU-1326-SM-4WWET
Stomach	Stomach	GTEX-ZLV1-0826-SM-4WWEQ
Stomach	Stomach	GTEX-ZP4G-1226-SM-4WWCJ
Stomach	Stomach	GTEX-ZPIC-2026-SM-57WG3
Stomach	Stomach	GTEX-ZPU1-1626-SM-4WWB2
Stomach	Stomach	GTEX-ZQUD-0826-SM-57WDQ
Stomach	Stomach	GTEX-ZT9W-1526-SM-4YCDE
Stomach	Stomach	GTEX-ZTX8-0426-SM-59HLG
Stomach	Stomach	GTEX-ZV6S-0926-SM-57WGB
Stomach	Stomach	GTEX-ZV7C-1626-SM-5NQ7E
Stomach	Stomach	GTEX-ZVP2-1626-SM-5GU5D
Stomach	Stomach	GTEX-ZVT2-1626-SM-51MRC
Stomach	Stomach	GTEX-ZVT3-2126-SM-59HL2
Stomach	Stomach	GTEX-ZVZP-1726-SM-5GZWY
Stomach	Stomach	GTEX-ZXES-1426-SM-5NQ8S
Stomach	Stomach	GTEX-ZY6K-0726-SM-5A5L8
Stomach	Stomach	GTEX-ZYFG-1326-SM-5GICJ
Stomach	Stomach	GTEX-ZYVF-2726-SM-5GID4
Stomach	Stomach	GTEX-ZYY3-1726-SM-5EGH3
Stomach	Stomach	GTEX-ZZ64-0426-SM-5E43F
Stomach	Stomach	GTEX-ZZPU-1426-SM-5GZZ6
Testis	Testis	GTEX-111CU-1726-SM-5EGHM
Testis	Testis	GTEX-111FC-1926-SM-5GZYC
Testis	Testis	GTEX-111VG-1926-SM-5GIDO
Testis	Testis	GTEX-111YS-2026-SM-5EGGL
Testis	Testis	GTEX-117XS-2026-SM-5GID1
Testis	Testis	GTEX-117YW-1526-SM-5EGGP
Testis	Testis	GTEX-117YX-2026-SM-5GIEF
Testis	Testis	GTEX-11DXY-0226-SM-5H123
Testis	Testis	GTEX-11DXZ-2126-SM-59881
Testis	Testis	GTEX-11EI6-2226-SM-5EGJM
Testis	Testis	GTEX-11EQ8-1426-SM-5EGJR
Testis	Testis	GTEX-11EQ9-1926-SM-5PNVV
Testis	Testis	GTEX-11GS4-2026-SM-5N9CP
Testis	Testis	GTEX-11LCK-2326-SM-5HL53
Testis	Testis	GTEX-11NSD-1026-SM-5N9BE

Testis	Testis	GTEX-11NUK-2626-SM-5A5MB
Testis	Testis	GTEX-11NV4-1726-SM-5N9FC
Testis	Testis	GTEX-11O72-0726-SM-5P9GO
Testis	Testis	GTEX-11OF3-1826-SM-5987N
Testis	Testis	GTEX-11ONC-2226-SM-5HL6D
Testis	Testis	GTEX-11P7K-1026-SM-5HL6Y
Testis	Testis	GTEX-11P82-1526-SM-5BC5M
Testis	Testis	GTEX-11TT1-2226-SM-5GU6B
Testis	Testis	GTEX-11TUW-2226-SM-5EQL9
Testis	Testis	GTEX-11WQC-2326-SM-5EQKE
Testis	Testis	GTEX-11ZUS-2726-SM-5FQUA
Testis	Testis	GTEX-1212Z-0326-SM-5FQSJ
Testis	Testis	GTEX-12696-0226-SM-5EGL3
Testis	Testis	GTEX-12BJ1-1326-SM-5BC5P
Testis	Testis	GTEX-12C56-1426-SM-5FQSW
Testis	Testis	GTEX-12WSH-0326-SM-5GCNH
Testis	Testis	GTEX-12WSI-2126-SM-5GCMV
Testis	Testis	GTEX-12WSL-2326-SM-5DUXQ
Testis	Testis	GTEX-12WSM-1326-SM-5GCP9
Testis	Testis	GTEX-12ZZY-0126-SM-5LZV2
Testis	Testis	GTEX-13111-1526-SM-5EGJX
Testis	Testis	GTEX-13112-0226-SM-5P9IV
Testis	Testis	GTEX-131XE-0426-SM-5IJF4
Testis	Testis	GTEX-132QS-1226-SM-5P9GD
Testis	Testis	GTEX-1339X-1926-SM-5PNVP
Testis	Testis	GTEX-1399R-1626-SM-5P9GG
Testis	Testis	GTEX-1399T-1526-SM-5P9J6
Testis	Testis	GTEX-139T6-1226-SM-5IFFC
Testis	Testis	GTEX-139TS-1726-SM-5IJG5
Testis	Testis	GTEX-139TT-2226-SM-5LZWO
Testis	Testis	GTEX-13FHP-2826-SM-5IJFW
Testis	Testis	GTEX-13FLW-2126-SM-5N9FD
Testis	Testis	GTEX-13FTW-1326-SM-5LZZD
Testis	Testis	GTEX-13N1W-2626-SM-5IJEP
Testis	Testis	GTEX-13N2G-0126-SM-5N9DV
Testis	Testis	GTEX-13NYB-2226-SM-5MR58
Testis	Testis	GTEX-13NZA-2526-SM-5IJFX
Testis	Testis	GTEX-13NZB-2026-SM-5MR4M
Testis	Testis	GTEX-13O1R-0726-SM-5IJEI
Testis	Testis	GTEX-13O21-1226-SM-5J2MK
Testis	Testis	GTEX-13O61-2026-SM-5J2M6
Testis	Testis	GTEX-13OVH-0726-SM-5N9BU
Testis	Testis	GTEX-13OVK-2226-SM-6LPJY
Testis	Testis	GTEX-13OVL-0426-SM-5IFG6
Testis	Testis	GTEX-13OW5-2526-SM-5L3I1
Testis	Testis	GTEX-13OW6-0126-SM-5IJGM
Testis	Testis	GTEX-13OW8-0526-SM-5KM24
Testis	Testis	GTEX-13QJ3-0226-SM-5S2PU
Testis	Testis	GTEX-13VXU-0726-SM-5J2O7
Testis	Testis	GTEX-144GL-0726-SM-5LU4P
Testis	Testis	GTEX-144GM-0426-SM-5Q5C8
Testis	Testis	GTEX-144GN-1626-SM-5Q5BU
Testis	Testis	GTEX-145LT-0426-SM-5LUAP
Testis	Testis	GTEX-145MF-1726-SM-5LU9H

Testis	Testis	GTEX-145MH-2326-SM-5O9AW
Testis	Testis	GTEX-145MO-0126-SM-5S2QU
Testis	Testis	GTEX-14753-0626-SM-5Q5CY
Testis	Testis	GTEX-147F4-0626-SM-5LUAK
Testis	Testis	GTEX-147GR-0626-SM-5S2PK
Testis	Testis	GTEX-147JS-0126-SM-5S2TW
Testis	Testis	GTEX-148VJ-2526-SM-5TDEI
Testis	Testis	GTEX-14A5H-0626-SM-5TDCO
Testis	Testis	GTEX-14A6H-2326-SM-5Q5B5
Testis	Testis	GTEX-14ABY-0626-SM-5Q5C9
Testis	Testis	GTEX-14B4R-1026-SM-5TDDS
Testis	Testis	GTEX-14BIL-2226-SM-73KWF
Testis	Testis	GTEX-14C38-0126-SM-5YY9V
Testis	Testis	GTEX-14C39-0526-SM-664OF
Testis	Testis	GTEX-14C5O-2326-SM-73KYU
Testis	Testis	GTEX-14DAR-1126-SM-793AT
Testis	Testis	GTEX-14E1K-1926-SM-73KWS
Testis	Testis	GTEX-14E6E-1026-SM-664N9
Testis	Testis	GTEX-14E7W-0726-SM-664OK
Testis	Testis	GTEX-14PHX-1426-SM-69LPP
Testis	Testis	GTEX-14PJ2-2026-SM-6AJAQ
Testis	Testis	GTEX-14PJ3-1626-SM-664O5
Testis	Testis	GTEX-14PJ4-1726-SM-664OC
Testis	Testis	GTEX-14PJN-1626-SM-68727
Testis	Testis	GTEX-15G19-1326-SM-6LPIR
Testis	Testis	GTEX-15RIE-0626-SM-6M47G
Testis	Testis	GTEX-15RIF-1826-SM-6M469
Testis	Testis	GTEX-15RJ7-1826-SM-7KUMJ
Testis	Testis	GTEX-15SHV-1926-SM-6LPI1
Testis	Testis	GTEX-15SKB-0326-SM-6M477
Testis	Testis	GTEX-16AAH-1626-SM-7EWE2
Testis	Testis	GTEX-16MTA-1126-SM-6LPJA
Testis	Testis	GTEX-16XZZ-0626-SM-6M47T
Testis	Testis	GTEX-16YQH-1826-SM-6LPJW
Testis	Testis	GTEX-16Z82-2726-SM-7KULV
Testis	Testis	GTEX-17F97-2726-SM-7IGQ4
Testis	Testis	GTEX-17F9E-1626-SM-7EWDB
Testis	Testis	GTEX-17GQL-1726-SM-718B8
Testis	Testis	GTEX-17HGU-0726-SM-7DUFN
Testis	Testis	GTEX-17HHE-1226-SM-793C6
Testis	Testis	GTEX-17HHY-0726-SM-7IGM6
Testis	Testis	GTEX-17HII-0126-SM-7KFSS
Testis	Testis	GTEX-17KNJ-1126-SM-7KFT6
Testis	Testis	GTEX-17MF6-2726-SM-7IGMZ
Testis	Testis	GTEX-17MFQ-0826-SM-793C8
Testis	Testis	GTEX-183FY-1926-SM-7KFRI
Testis	Testis	GTEX-18465-1526-SM-7KFTV
Testis	Testis	GTEX-18A66-0726-SM-72D77
Testis	Testis	GTEX-18A67-1826-SM-7KFT7
Testis	Testis	GTEX-18A6Q-2626-SM-7KFT3
Testis	Testis	GTEX-18A7B-1526-SM-7KFTH
Testis	Testis	GTEX-18D9B-2726-SM-72D73
Testis	Testis	GTEX-18QFQ-1926-SM-72D5H
Testis	Testis	GTEX-1A3MV-0726-SM-72D5M

Testis	Testis	GTEX-1A3MX-1026-SM-731F8
Testis	Testis	GTEX-1AX8Z-2826-SM-73KTZ
Testis	Testis	GTEX-1AX9I-1826-SM-72D5I
Testis	Testis	GTEX-1AX9J-1226-SM-72D6P
Testis	Testis	GTEX-1AX9K-1826-SM-731CY
Testis	Testis	GTEX-1AYCT-1026-SM-79ONR
Testis	Testis	GTEX-1B8KE-1426-SM-7EWEL
Testis	Testis	GTEX-1B8KZ-1826-SM-73KVJ
Testis	Testis	GTEX-1B8SF-2926-SM-731DL
Testis	Testis	GTEX-1B996-2326-SM-731EC
Testis	Testis	GTEX-1BAJH-0726-SM-7IGMF
Testis	Testis	GTEX-1C4CL-1926-SM-731DY
Testis	Testis	GTEX-1C64N-2826-SM-7IGP5
Testis	Testis	GTEX-1C64O-2126-SM-7IGPB
Testis	Testis	GTEX-1C6VQ-1426-SM-73KTW
Testis	Testis	GTEX-1C6VR-1526-SM-7MKG9
Testis	Testis	GTEX-1C6VS-1126-SM-7EWEW
Testis	Testis	GTEX-1CAMR-1526-SM-79OLP
Testis	Testis	GTEX-1CB4G-2426-SM-7DUGM
Testis	Testis	GTEX-1CB4I-0926-SM-7MKFY
Testis	Testis	GTEX-1CB4J-1626-SM-79OOK
Testis	Testis	GTEX-1E1VI-1026-SM-7MKGQ
Testis	Testis	GTEX-1E2YA-2826-SM-7EPIN
Testis	Testis	GTEX-1EKGG-2726-SM-79OOD
Testis	Testis	GTEX-1EU9M-1626-SM-7EWF2
Testis	Testis	GTEX-1F6IF-0526-SM-7MKHD
Testis	Testis	GTEX-1GMRU-0626-SM-7MKH2
Testis	Testis	GTEX-N7MS-0126-SM-3TW8O
Testis	Testis	GTEX-NFK9-0126-SM-3LK5H
Testis	Testis	GTEX-NPJ8-1226-SM-3MJHM
Testis	Testis	GTEX-O5YT-2126-SM-3MJGD
Testis	Testis	GTEX-OHPM-2126-SM-3LK75
Testis	Testis	GTEX-OIZH-2126-SM-3NB1P
Testis	Testis	GTEX-OIZI-0126-SM-3NB13
Testis	Testis	GTEX-OOBJ-2126-SM-3NB1N
Testis	Testis	GTEX-OOBK-2126-SM-3LK5T
Testis	Testis	GTEX-OXRL-2126-SM-3NM98
Testis	Testis	GTEX-P4QS-2126-SM-3NMCF
Testis	Testis	GTEX-PLZ5-1526-SM-3P5ZX
Testis	Testis	GTEX-PLZ6-1226-SM-3P5ZS
Testis	Testis	GTEX-PW2O-1426-SM-48TCD
Testis	Testis	GTEX-Q2AH-1526-SM-48TZG
Testis	Testis	GTEX-Q2AI-1226-SM-48U14
Testis	Testis	GTEX-QEG4-0126-SM-48TZE
Testis	Testis	GTEX-QEG5-0126-SM-4R1JR
Testis	Testis	GTEX-QLQ7-1426-SM-4R1JX
Testis	Testis	GTEX-QLQW-1026-SM-447A9
Testis	Testis	GTEX-QMRM-1526-SM-4R1K6
Testis	Testis	GTEX-QV31-1126-SM-4R1K4
Testis	Testis	GTEX-QV44-1726-SM-4R1KG
Testis	Testis	GTEX-R55C-1426-SM-48FED
Testis	Testis	GTEX-R55D-0126-SM-48FEL
Testis	Testis	GTEX-R55E-0726-SM-48FCZ
Testis	Testis	GTEX-REY6-0126-SM-48FDT

Testis	Testis	GTEX-RM2N-1326-SM-48FCW
Testis	Testis	GTEX-RN64-2326-SM-48FDW
Testis	Testis	GTEX-RUSQ-2126-SM-47JXK
Testis	Testis	GTEX-RWSA-2426-SM-47JXR
Testis	Testis	GTEX-S33H-0126-SM-4AD62
Testis	Testis	GTEX-S3XE-1526-SM-4AD5A
Testis	Testis	GTEX-S4Q7-1226-SM-4AD5I
Testis	Testis	GTEX-S4Z8-2126-SM-4AD5H
Testis	Testis	GTEX-S7PM-0626-SM-4AD4Q
Testis	Testis	GTEX-S7SE-0326-SM-4AT5Q
Testis	Testis	GTEX-S95S-1126-SM-4B64E
Testis	Testis	GTEX-SNMC-1026-SM-4DM7K
Testis	Testis	GTEX-SNOS-1126-SM-4DM67
Testis	Testis	GTEX-T5JC-0726-SM-4DM55
Testis	Testis	GTEX-T6MN-2026-SM-4DM7L
Testis	Testis	GTEX-T8EM-0126-SM-4DM5R
Testis	Testis	GTEX-TKQ1-0926-SM-4DXU2
Testis	Testis	GTEX-TKQ2-1526-SM-4DXUN
Testis	Testis	GTEX-U3ZH-1526-SM-4DXV1
Testis	Testis	GTEX-U3ZM-1626-SM-4DXSK
Testis	Testis	GTEX-U4B1-1526-SM-4DXSL
Testis	Testis	GTEX-U8T8-1126-SM-4DXUE
Testis	Testis	GTEX-U8XE-0126-SM-4E3I3
Testis	Testis	GTEX-UPJH-0126-SM-4IHLL
Testis	Testis	GTEX-V1D1-2126-SM-4JBH4
Testis	Testis	GTEX-V955-1926-SM-4KL1L
Testis	Testis	GTEX-VJYA-1426-SM-4KL1Y
Testis	Testis	GTEX-WFG8-1926-SM-4LVM1
Testis	Testis	GTEX-WFON-2026-SM-4LVMW
Testis	Testis	GTEX-WH7G-1926-SM-4LVMM
Testis	Testis	GTEX-WHSB-2126-SM-4M1XF
Testis	Testis	GTEX-WHSE-0426-SM-4M1XO
Testis	Testis	GTEX-WK11-0326-SM-4OOS6
Testis	Testis	GTEX-WOFM-1126-SM-4OOSB
Testis	Testis	GTEX-WVJS-2726-SM-4MVNU
Testis	Testis	GTEX-WVLH-2626-SM-4MVNV
Testis	Testis	GTEX-WY7C-2226-SM-4ONCS
Testis	Testis	GTEX-WYJK-1826-SM-4ONDM
Testis	Testis	GTEX-WZTO-0326-SM-4PQYZ
Testis	Testis	GTEX-X261-2326-SM-4PQYU
Testis	Testis	GTEX-X3Y1-2626-SM-4PQZI
Testis	Testis	GTEX-X5EB-2026-SM-4E3KA
Testis	Testis	GTEX-XAJ8-1326-SM-47JYT
Testis	Testis	GTEX-XBEC-0126-SM-4GIDT
Testis	Testis	GTEX-XBED-2026-SM-4AT5D
Testis	Testis	GTEX-XGQ4-2026-SM-4AT6G
Testis	Testis	GTEX-XLM4-1526-SM-4AT6D
Testis	Testis	GTEX-XMK1-2026-SM-4B65K
Testis	Testis	GTEX-XPT6-1626-SM-4B655
Testis	Testis	GTEX-XPVG-2226-SM-4B65U
Testis	Testis	GTEX-XQ3S-2726-SM-4BOP2
Testis	Testis	GTEX-Y111-2426-SM-4TT23
Testis	Testis	GTEX-Y3I4-2026-SM-4TT6Z
Testis	Testis	GTEX-Y5V6-1726-SM-4VDSZ

Testis	Testis	GTEX-Y8E4-2226-SM-5LU94
Testis	Testis	GTEX-Y9LG-1726-SM-4VBQE
Testis	Testis	GTEX-YB5E-1926-SM-5IFIG
Testis	Testis	GTEX-YEC3-1726-SM-5IFIK
Testis	Testis	GTEX-YEC4-1526-SM-4W1YU
Testis	Testis	GTEX-YF7O-2026-SM-4W1YE
Testis	Testis	GTEX-YFCO-1726-SM-4W21S
Testis	Testis	GTEX-YJ89-0626-SM-4TT3Z
Testis	Testis	GTEX-Z93S-1726-SM-5HL8G
Testis	Testis	GTEX-ZA64-1626-SM-5CVME
Testis	Testis	GTEX-ZAB4-0126-SM-5CVMG
Testis	Testis	GTEX-ZAB5-2426-SM-5CVMW
Testis	Testis	GTEX-ZDTT-2026-SM-5K7TY
Testis	Testis	GTEX-ZDYS-1326-SM-5IJFF
Testis	Testis	GTEX-ZLFU-2026-SM-4WWG2
Testis	Testis	GTEX-ZPU1-2126-SM-57WED
Testis	Testis	GTEX-ZQUD-2026-SM-51MSM
Testis	Testis	GTEX-ZT9W-2226-SM-57WFU
Testis	Testis	GTEX-ZT9X-1426-SM-5DUX1
Testis	Testis	GTEX-ZTSS-1526-SM-51MTC
Testis	Testis	GTEX-ZTX8-1126-SM-51MRM
Testis	Testis	GTEX-ZUA1-2726-SM-59HLJ
Testis	Testis	GTEX-ZV7C-2026-SM-5NQ8F
Testis	Testis	GTEX-ZVTK-0126-SM-57WDG
Testis	Testis	GTEX-ZVZP-2226-SM-57WBF
Testis	Testis	GTEX-ZYFC-0126-SM-5GIEH
Testis	Testis	GTEX-ZYT6-2726-SM-5GICP
Testis	Testis	GTEX-ZZ64-1126-SM-5GZXY
Thyroid	Thyroid	GTEX-111CU-0226-SM-5GZXC
Thyroid	Thyroid	GTEX-111FC-1026-SM-5GZX1
Thyroid	Thyroid	GTEX-111VG-0526-SM-5N9BW
Thyroid	Thyroid	GTEX-111YS-0726-SM-5GZY8
Thyroid	Thyroid	GTEX-1122O-0226-SM-5N9DA
Thyroid	Thyroid	GTEX-1128S-0126-SM-5H12S
Thyroid	Thyroid	GTEX-113JC-0126-SM-5EGJW
Thyroid	Thyroid	GTEX-117XS-0526-SM-5987Q
Thyroid	Thyroid	GTEX-117YW-0126-SM-5EGGN
Thyroid	Thyroid	GTEX-117YX-1226-SM-5H11S
Thyroid	Thyroid	GTEX-1192W-0126-SM-5EGGS
Thyroid	Thyroid	GTEX-1192X-1126-SM-5EGGU
Thyroid	Thyroid	GTEX-11DXX-0226-SM-5P9HL
Thyroid	Thyroid	GTEX-11DXY-0426-SM-5H12R
Thyroid	Thyroid	GTEX-11DXZ-0926-SM-5N9CG
Thyroid	Thyroid	GTEX-11DYG-0826-SM-5N9GH
Thyroid	Thyroid	GTEX-11DZ1-2726-SM-5A5KH
Thyroid	Thyroid	GTEX-11EI6-0726-SM-59866
Thyroid	Thyroid	GTEX-11EM3-0126-SM-5985K
Thyroid	Thyroid	GTEX-11EMC-0226-SM-5EGLP
Thyroid	Thyroid	GTEX-11EQ8-0826-SM-5N9FG
Thyroid	Thyroid	GTEX-11EQ9-0626-SM-5A5K1
Thyroid	Thyroid	GTEX-11GS4-0826-SM-5986J
Thyroid	Thyroid	GTEX-11GSO-0626-SM-5A5LW
Thyroid	Thyroid	GTEX-11I78-0526-SM-5986A
Thyroid	Thyroid	GTEX-11LCK-0526-SM-5A5M9

Thyroid	Thyroid	GTEX-11NSD-0126-SM-5987F
Thyroid	Thyroid	GTEX-11NUK-1026-SM-5HL5J
Thyroid	Thyroid	GTEX-11NV4-0626-SM-5N9BR
Thyroid	Thyroid	GTEX-11O72-2326-SM-5BC7H
Thyroid	Thyroid	GTEX-11OF3-0626-SM-5BC4Y
Thyroid	Thyroid	GTEX-11P7K-0226-SM-5986Z
Thyroid	Thyroid	GTEX-11P81-0126-SM-5HL5Y
Thyroid	Thyroid	GTEX-11P82-0226-SM-5HL4O
Thyroid	Thyroid	GTEX-11TT1-1126-SM-5P9GV
Thyroid	Thyroid	GTEX-11TTK-0826-SM-5N9EG
Thyroid	Thyroid	GTEX-11TUW-0226-SM-5LU8X
Thyroid	Thyroid	GTEX-11UD2-0626-SM-5GU6L
Thyroid	Thyroid	GTEX-11VI4-0226-SM-5GU6C
Thyroid	Thyroid	GTEX-11XUK-0226-SM-5EQLW
Thyroid	Thyroid	GTEX-11ZTS-1126-SM-5LU9X
Thyroid	Thyroid	GTEX-11ZTT-1026-SM-5EQKF
Thyroid	Thyroid	GTEX-11ZVC-0126-SM-5986G
Thyroid	Thyroid	GTEX-1211K-0726-SM-5FQUW
Thyroid	Thyroid	GTEX-1212Z-0426-SM-5FQT6
Thyroid	Thyroid	GTEX-12584-0826-SM-5FQSK
Thyroid	Thyroid	GTEX-12696-0326-SM-5EGL4
Thyroid	Thyroid	GTEX-1269C-0226-SM-5EGKS
Thyroid	Thyroid	GTEX-12BJ1-0426-SM-5FQSO
Thyroid	Thyroid	GTEX-12WSC-0826-SM-5EQ5Q
Thyroid	Thyroid	GTEX-12WSD-0926-SM-5GCNL
Thyroid	Thyroid	GTEX-12WSE-1226-SM-73KUF
Thyroid	Thyroid	GTEX-12WSG-0226-SM-5EGIF
Thyroid	Thyroid	GTEX-12WSH-0226-SM-5GCOG
Thyroid	Thyroid	GTEX-12WSJ-0326-SM-5GCMT
Thyroid	Thyroid	GTEX-12WSK-0926-SM-5CVNQ
Thyroid	Thyroid	GTEX-12WSL-0626-SM-5GCOY
Thyroid	Thyroid	GTEX-12WSN-0726-SM-5GCMS
Thyroid	Thyroid	GTEX-12ZZX-1226-SM-5EGHS
Thyroid	Thyroid	GTEX-12ZZY-0826-SM-5EQMT
Thyroid	Thyroid	GTEX-12ZZZ-1226-SM-59HK1
Thyroid	Thyroid	GTEX-13111-0226-SM-5EQ55
Thyroid	Thyroid	GTEX-13112-0326-SM-5P9IW
Thyroid	Thyroid	GTEX-13113-0126-SM-5LZVX
Thyroid	Thyroid	GTEX-1313W-0726-SM-5EGK1
Thyroid	Thyroid	GTEX-131XE-0126-SM-5LZVC
Thyroid	Thyroid	GTEX-131XF-1826-SM-5EGKG
Thyroid	Thyroid	GTEX-131XG-0226-SM-5IFG1
Thyroid	Thyroid	GTEX-131XH-0526-SM-5DUX7
Thyroid	Thyroid	GTEX-131YS-0726-SM-5P9G9
Thyroid	Thyroid	GTEX-132AR-1126-SM-5P9GA
Thyroid	Thyroid	GTEX-132NY-1026-SM-5P9IY
Thyroid	Thyroid	GTEX-132QS-0326-SM-5IJFN
Thyroid	Thyroid	GTEX-133LE-0326-SM-5P9G4
Thyroid	Thyroid	GTEX-1399R-0126-SM-5IFEV
Thyroid	Thyroid	GTEX-1399T-0126-SM-5KM15
Thyroid	Thyroid	GTEX-1399U-0326-SM-5P9G5
Thyroid	Thyroid	GTEX-139T6-0326-SM-5J2LY
Thyroid	Thyroid	GTEX-139TS-0126-SM-5K7XJ
Thyroid	Thyroid	GTEX-139UW-0126-SM-5KM1B

Thyroid	Thyroid	GTEX-139YR-1226-SM-5IFEU
Thyroid	Thyroid	GTEX-13CF3-0926-SM-5LZZC
Thyroid	Thyroid	GTEX-13D11-0226-SM-5LZXL
Thyroid	Thyroid	GTEX-13FH7-0126-SM-5KLZ1
Thyroid	Thyroid	GTEX-13FHO-0926-SM-5N9EW
Thyroid	Thyroid	GTEX-13FHP-0926-SM-5L3EC
Thyroid	Thyroid	GTEX-13FLV-0226-SM-5J2OF
Thyroid	Thyroid	GTEX-13FLW-0326-SM-5J2M4
Thyroid	Thyroid	GTEX-13FTW-0626-SM-5IFEX
Thyroid	Thyroid	GTEX-13FTY-0726-SM-5J2OH
Thyroid	Thyroid	GTEX-13FXS-0726-SM-5LZXJ
Thyroid	Thyroid	GTEX-13G51-1226-SM-5K7Z3
Thyroid	Thyroid	GTEX-13IVO-0926-SM-5KLZP
Thyroid	Thyroid	GTEX-13JVG-0926-SM-5IJE1
Thyroid	Thyroid	GTEX-13N11-1026-SM-5K7XQ
Thyroid	Thyroid	GTEX-13N1W-0826-SM-5MR5J
Thyroid	Thyroid	GTEX-13N2G-0726-SM-5MR38
Thyroid	Thyroid	GTEX-13NYB-0726-SM-5MR4J
Thyroid	Thyroid	GTEX-13NYC-2426-SM-5MR3K
Thyroid	Thyroid	GTEX-13NZ8-0226-SM-5J2OK
Thyroid	Thyroid	GTEX-13NZ9-1126-SM-5MR37
Thyroid	Thyroid	GTEX-13NZA-1026-SM-5MR48
Thyroid	Thyroid	GTEX-13O1R-0826-SM-5J2MB
Thyroid	Thyroid	GTEX-13O21-2226-SM-5MR3L
Thyroid	Thyroid	GTEX-13O3O-0926-SM-5KM1F
Thyroid	Thyroid	GTEX-13O3P-0726-SM-5J2OM
Thyroid	Thyroid	GTEX-13O3Q-0626-SM-5IJG1
Thyroid	Thyroid	GTEX-13O61-0226-SM-5KM52
Thyroid	Thyroid	GTEX-13OVG-0226-SM-5LU93
Thyroid	Thyroid	GTEX-13OVI-0826-SM-5KLZ8
Thyroid	Thyroid	GTEX-13OVJ-0626-SM-5J2O2
Thyroid	Thyroid	GTEX-13OVK-0226-SM-6M472
Thyroid	Thyroid	GTEX-13OW5-0626-SM-5J2N2
Thyroid	Thyroid	GTEX-13OW6-0726-SM-5L3FX
Thyroid	Thyroid	GTEX-13OW7-0826-SM-5L3EL
Thyroid	Thyroid	GTEX-13OW8-0126-SM-5IJE5
Thyroid	Thyroid	GTEX-13PDP-1026-SM-5L3FA
Thyroid	Thyroid	GTEX-13PL6-1026-SM-5L3E5
Thyroid	Thyroid	GTEX-13PVQ-0726-SM-5L3GI
Thyroid	Thyroid	GTEX-13PVR-0626-SM-5S2RC
Thyroid	Thyroid	GTEX-13QBU-0626-SM-5J2OG
Thyroid	Thyroid	GTEX-13QJ3-0926-SM-73KX5
Thyroid	Thyroid	GTEX-13QJC-0826-SM-5RQKC
Thyroid	Thyroid	GTEX-13RTJ-0326-SM-5YYAE
Thyroid	Thyroid	GTEX-13RTK-0326-SM-5RQHS
Thyroid	Thyroid	GTEX-13S86-1126-SM-5RQJX
Thyroid	Thyroid	GTEX-13U4I-0526-SM-5LU59
Thyroid	Thyroid	GTEX-13VXT-0626-SM-5SIA1
Thyroid	Thyroid	GTEX-13VXU-0826-SM-5KLZ2
Thyroid	Thyroid	GTEX-13W46-0926-SM-5LU3T
Thyroid	Thyroid	GTEX-13X6H-0526-SM-5LU4Q
Thyroid	Thyroid	GTEX-13X6J-0826-SM-5LU32
Thyroid	Thyroid	GTEX-13YAN-0926-SM-5O9C3
Thyroid	Thyroid	GTEX-144GL-1226-SM-5O9A4

Thyroid	Thyroid	GTEX-144GM-0226-SM-5Q5CB
Thyroid	Thyroid	GTEX-144GO-0126-SM-5LUAO
Thyroid	Thyroid	GTEX-145LT-0226-SM-5S2QK
Thyroid	Thyroid	GTEX-145LU-0426-SM-5O9AH
Thyroid	Thyroid	GTEX-145ME-0126-SM-5S2QM
Thyroid	Thyroid	GTEX-145MG-0826-SM-5Q5C2
Thyroid	Thyroid	GTEX-145MH-0426-SM-5LU8T
Thyroid	Thyroid	GTEX-145MI-1126-SM-5O9AK
Thyroid	Thyroid	GTEX-146FQ-0726-SM-5LUA7
Thyroid	Thyroid	GTEX-146FR-0326-SM-5SI8U
Thyroid	Thyroid	GTEX-14753-0926-SM-5Q5BI
Thyroid	Thyroid	GTEX-1477Z-0226-SM-5TDCI
Thyroid	Thyroid	GTEX-147F4-0826-SM-5QGRB
Thyroid	Thyroid	GTEX-147GR-0726-SM-5S2PL
Thyroid	Thyroid	GTEX-148VI-0526-SM-5TDDG
Thyroid	Thyroid	GTEX-148VJ-0726-SM-5LU8J
Thyroid	Thyroid	GTEX-1497J-0126-SM-5Q5BK
Thyroid	Thyroid	GTEX-14A5H-0726-SM-5Q5DW
Thyroid	Thyroid	GTEX-14A6H-2426-SM-5Q5BO
Thyroid	Thyroid	GTEX-14ABY-0926-SM-5Q5DY
Thyroid	Thyroid	GTEX-14AS3-0226-SM-5Q5B6
Thyroid	Thyroid	GTEX-14ASI-0726-SM-5Q5DC
Thyroid	Thyroid	GTEX-14B4R-0126-SM-5TDE4
Thyroid	Thyroid	GTEX-14BIN-0126-SM-5TDCG
Thyroid	Thyroid	GTEX-14BMU-0226-SM-5S2QA
Thyroid	Thyroid	GTEX-14BMV-0726-SM-73KVE
Thyroid	Thyroid	GTEX-14C38-0826-SM-5S2U8
Thyroid	Thyroid	GTEX-14C39-0226-SM-5TDDW
Thyroid	Thyroid	GTEX-14C5O-0826-SM-5TDEG
Thyroid	Thyroid	GTEX-14DAQ-0826-SM-73KWT
Thyroid	Thyroid	GTEX-14E6C-2626-SM-5RQJP
Thyroid	Thyroid	GTEX-14E6E-0326-SM-73KY6
Thyroid	Thyroid	GTEX-14E7W-0926-SM-5YYA4
Thyroid	Thyroid	GTEX-14ICK-1626-SM-6ETZX
Thyroid	Thyroid	GTEX-14ICL-0426-SM-5RQJ3
Thyroid	Thyroid	GTEX-14JIY-1226-SM-6871R
Thyroid	Thyroid	GTEX-14PHW-2926-SM-6AJBA
Thyroid	Thyroid	GTEX-14PII-0826-SM-6871S
Thyroid	Thyroid	GTEX-14PJ3-0126-SM-69LQP
Thyroid	Thyroid	GTEX-14PJ4-0326-SM-664OT
Thyroid	Thyroid	GTEX-14PJ6-0326-SM-6871H
Thyroid	Thyroid	GTEX-14PJM-1326-SM-664NX
Thyroid	Thyroid	GTEX-14PJO-0626-SM-6LLHH
Thyroid	Thyroid	GTEX-14PK6-0426-SM-6EU1J
Thyroid	Thyroid	GTEX-14PKU-0326-SM-6AJA7
Thyroid	Thyroid	GTEX-14PKV-0626-SM-6AJA2
Thyroid	Thyroid	GTEX-14PN3-0826-SM-69LOS
Thyroid	Thyroid	GTEX-14PN4-1526-SM-6871V
Thyroid	Thyroid	GTEX-14PQA-1226-SM-6M47A
Thyroid	Thyroid	GTEX-14XAO-0426-SM-6AJB6
Thyroid	Thyroid	GTEX-15CHC-0126-SM-5YYBA
Thyroid	Thyroid	GTEX-15CHQ-0826-SM-69LOT
Thyroid	Thyroid	GTEX-15CHR-1726-SM-7DUGW
Thyroid	Thyroid	GTEX-15D1Q-0626-SM-6AJAZ

Thyroid	Thyroid	GTEX-15DCZ-1226-SM-6871P
Thyroid	Thyroid	GTEX-15DDE-0626-SM-69LOK
Thyroid	Thyroid	GTEX-15DZA-0226-SM-7KFS6
Thyroid	Thyroid	GTEX-15EO6-0126-SM-6LPKJ
Thyroid	Thyroid	GTEX-15ER7-0726-SM-7KUMF
Thyroid	Thyroid	GTEX-15ETS-0526-SM-6PAN3
Thyroid	Thyroid	GTEX-15EU6-1426-SM-6M48E
Thyroid	Thyroid	GTEX-15FZZ-0226-SM-6LLI4
Thyroid	Thyroid	GTEX-15G19-0626-SM-6M474
Thyroid	Thyroid	GTEX-15G1A-0326-SM-6M467
Thyroid	Thyroid	GTEX-15RIE-0426-SM-7KUMH
Thyroid	Thyroid	GTEX-15RJ7-0326-SM-6M47H
Thyroid	Thyroid	GTEX-15RJE-1326-SM-6LPI6
Thyroid	Thyroid	GTEX-15SB6-1526-SM-7KUMQ
Thyroid	Thyroid	GTEX-15SHU-0726-SM-7KUFI
Thyroid	Thyroid	GTEX-15SHV-0426-SM-6M476
Thyroid	Thyroid	GTEX-15UF6-1126-SM-6LPJ3
Thyroid	Thyroid	GTEX-169BO-0326-SM-7EPIM
Thyroid	Thyroid	GTEX-16AAH-0326-SM-7DHML
Thyroid	Thyroid	GTEX-16BQI-0726-SM-6LPJZ
Thyroid	Thyroid	GTEX-16GPK-0926-SM-6LPJ9
Thyroid	Thyroid	GTEX-16MT8-0626-SM-6M47Q
Thyroid	Thyroid	GTEX-16MTA-0726-SM-7KUL4
Thyroid	Thyroid	GTEX-16NGA-0326-SM-718AJ
Thyroid	Thyroid	GTEX-16NPX-1426-SM-6LPK3
Thyroid	Thyroid	GTEX-16XZY-0726-SM-79OMU
Thyroid	Thyroid	GTEX-16XZZ-0826-SM-7IGM3
Thyroid	Thyroid	GTEX-16YQH-0326-SM-6LPJV
Thyroid	Thyroid	GTEX-16Z82-0426-SM-7EPGX
Thyroid	Thyroid	GTEX-178AV-0726-SM-6LPJI
Thyroid	Thyroid	GTEX-17EVP-0126-SM-7EPHW
Thyroid	Thyroid	GTEX-17EVQ-0526-SM-7KFSK
Thyroid	Thyroid	GTEX-17F96-0526-SM-79OLE
Thyroid	Thyroid	GTEX-17F97-0626-SM-7IGOH
Thyroid	Thyroid	GTEX-17F9E-0626-SM-79ON5
Thyroid	Thyroid	GTEX-17F9Y-0526-SM-7EWDC
Thyroid	Thyroid	GTEX-17HG3-0226-SM-7EWEA
Thyroid	Thyroid	GTEX-17HGU-0826-SM-7EWE5
Thyroid	Thyroid	GTEX-17HHE-0426-SM-79OK3
Thyroid	Thyroid	GTEX-17HHY-0826-SM-7EPID
Thyroid	Thyroid	GTEX-17HII-1926-SM-79OLB
Thyroid	Thyroid	GTEX-17JCI-0626-SM-7IGM7
Thyroid	Thyroid	GTEX-17KNJ-1026-SM-79ONK
Thyroid	Thyroid	GTEX-17MF6-0626-SM-7LT8D
Thyroid	Thyroid	GTEX-183FY-0626-SM-79OKR
Thyroid	Thyroid	GTEX-183WM-2626-SM-7KFRY
Thyroid	Thyroid	GTEX-18465-1426-SM-7KFTF
Thyroid	Thyroid	GTEX-18A66-0826-SM-72D5Z
Thyroid	Thyroid	GTEX-18A67-0826-SM-7KFTI
Thyroid	Thyroid	GTEX-18A6Q-0726-SM-7LT8Y
Thyroid	Thyroid	GTEX-18A7A-0826-SM-7KFTJ
Thyroid	Thyroid	GTEX-18D9A-0126-SM-7KFSI
Thyroid	Thyroid	GTEX-18D9B-0726-SM-72D6K
Thyroid	Thyroid	GTEX-18D9U-1026-SM-72D5R

Thyroid	Thyroid	GTEX-18QFQ-0726-SM-7LG6D
Thyroid	Thyroid	GTEX-1A3MV-0326-SM-73KW7
Thyroid	Thyroid	GTEX-1A8FM-0726-SM-7DUGK
Thyroid	Thyroid	GTEX-1A8G6-0626-SM-7IGNB
Thyroid	Thyroid	GTEX-1A8G7-1026-SM-73KVA
Thyroid	Thyroid	GTEX-1AMEY-0126-SM-73KTX
Thyroid	Thyroid	GTEX-1AMFI-0526-SM-7189M
Thyroid	Thyroid	GTEX-1AX8Z-0826-SM-7DUFZ
Thyroid	Thyroid	GTEX-1AX9I-0626-SM-72D54
Thyroid	Thyroid	GTEX-1AX9J-2126-SM-731DD
Thyroid	Thyroid	GTEX-1AX9K-0626-SM-73KVD
Thyroid	Thyroid	GTEX-1AYCT-0226-SM-73KVB
Thyroid	Thyroid	GTEX-1B8KE-0626-SM-7189H
Thyroid	Thyroid	GTEX-1B8KZ-0426-SM-731DP
Thyroid	Thyroid	GTEX-1B8L1-1626-SM-7IGMH
Thyroid	Thyroid	GTEX-1B8SF-0626-SM-73KVV
Thyroid	Thyroid	GTEX-1B8SG-1126-SM-7IGMT
Thyroid	Thyroid	GTEX-1B932-0926-SM-73KUP
Thyroid	Thyroid	GTEX-1B97I-0326-SM-7DUGB
Thyroid	Thyroid	GTEX-1BAJH-0926-SM-79OO6
Thyroid	Thyroid	GTEX-1C2JI-0326-SM-7EWFD
Thyroid	Thyroid	GTEX-1C4CL-0726-SM-7IGP9
Thyroid	Thyroid	GTEX-1C64N-1026-SM-79ONM
Thyroid	Thyroid	GTEX-1C6VR-0426-SM-7IGN6
Thyroid	Thyroid	GTEX-1C6VS-0826-SM-7EWEI
Thyroid	Thyroid	GTEX-1CAMQ-1126-SM-7EWFE
Thyroid	Thyroid	GTEX-1CAMR-0226-SM-7DUGO
Thyroid	Thyroid	GTEX-1CB4F-0826-SM-793CV
Thyroid	Thyroid	GTEX-1CB4H-0126-SM-7IGN2
Thyroid	Thyroid	GTEX-1CB4I-0726-SM-7DUGS
Thyroid	Thyroid	GTEX-1CB4J-1426-SM-7MKFR
Thyroid	Thyroid	GTEX-1EH9U-0926-SM-7EWF1
Thyroid	Thyroid	GTEX-1EKGG-0726-SM-7IGPX
Thyroid	Thyroid	GTEX-1EMGI-0826-SM-7EPHY
Thyroid	Thyroid	GTEX-1EN7A-1026-SM-7IGPZ
Thyroid	Thyroid	GTEX-N7MS-2326-SM-2HMLD
Thyroid	Thyroid	GTEX-NFK9-0726-SM-2HMJW
Thyroid	Thyroid	GTEX-OHPK-2626-SM-2HMK9
Thyroid	Thyroid	GTEX-OHPM-2626-SM-33HC5
Thyroid	Thyroid	GTEX-OIZG-0226-SM-2TC5L
Thyroid	Thyroid	GTEX-OIZI-0726-SM-2XCEI
Thyroid	Thyroid	GTEX-OOBJ-2626-SM-2I3F6
Thyroid	Thyroid	GTEX-OXRK-0626-SM-2HMJ5
Thyroid	Thyroid	GTEX-OXRL-2626-SM-2I3F1
Thyroid	Thyroid	GTEX-OXRO-1226-SM-48TDL
Thyroid	Thyroid	GTEX-OXRP-0326-SM-33HBJ
Thyroid	Thyroid	GTEX-P4PQ-2626-SM-33HC9
Thyroid	Thyroid	GTEX-P4QS-2626-SM-2I3EV
Thyroid	Thyroid	GTEX-P4QT-2626-SM-2I3FM
Thyroid	Thyroid	GTEX-P78B-0526-SM-2I5F7
Thyroid	Thyroid	GTEX-PLZ4-1226-SM-2I5FE
Thyroid	Thyroid	GTEX-POYW-0826-SM-2XCEM
Thyroid	Thyroid	GTEX-PWCY-2326-SM-2I3EQ
Thyroid	Thyroid	GTEX-PWN1-2626-SM-2I3FH

Thyroid	Thyroid	GTEX-PX3G-2626-SM-2I3EG
Thyroid	Thyroid	GTEX-Q2AG-0826-SM-2HMKF
Thyroid	Thyroid	GTEX-Q2AH-0726-SM-2I3EA
Thyroid	Thyroid	GTEX-Q2AI-0326-SM-2I3EK
Thyroid	Thyroid	GTEX-Q734-0526-SM-2I3EH
Thyroid	Thyroid	GTEX-QDVJ-0226-SM-2I5FV
Thyroid	Thyroid	GTEX-QDVN-0626-SM-2I3FP
Thyroid	Thyroid	GTEX-QEG5-0826-SM-2I5GF
Thyroid	Thyroid	GTEX-QEL4-0726-SM-3GIJ5
Thyroid	Thyroid	GTEX-QLQ7-0726-SM-2I5G2
Thyroid	Thyroid	GTEX-QV31-0726-SM-3GAEG
Thyroid	Thyroid	GTEX-QV44-0826-SM-2S1RG
Thyroid	Thyroid	GTEX-QXCU-0326-SM-2TC63
Thyroid	Thyroid	GTEX-R3RS-0726-SM-3GIJR
Thyroid	Thyroid	GTEX-R53T-0526-SM-3GADL
Thyroid	Thyroid	GTEX-R55C-0626-SM-2TF4Q
Thyroid	Thyroid	GTEX-R55E-0826-SM-2TC5M
Thyroid	Thyroid	GTEX-R55G-0726-SM-2TC6J
Thyroid	Thyroid	GTEX-REY6-0526-SM-2TF5M
Thyroid	Thyroid	GTEX-RM2N-0526-SM-2TF4N
Thyroid	Thyroid	GTEX-RN64-0626-SM-2TC5V
Thyroid	Thyroid	GTEX-RNOR-0926-SM-2TF56
Thyroid	Thyroid	GTEX-RTLS-0626-SM-5SI7Z
Thyroid	Thyroid	GTEX-RU1J-0226-SM-2TF5Y
Thyroid	Thyroid	GTEX-RU72-0126-SM-2TF6Z
Thyroid	Thyroid	GTEX-RUSQ-1026-SM-2TF6V
Thyroid	Thyroid	GTEX-RVPV-1226-SM-2TF73
Thyroid	Thyroid	GTEX-RWS6-0626-SM-2XCAS
Thyroid	Thyroid	GTEX-RWSA-0826-SM-2XCBF
Thyroid	Thyroid	GTEX-S32W-0726-SM-2XCBL
Thyroid	Thyroid	GTEX-S341-0226-SM-5S2VG
Thyroid	Thyroid	GTEX-S7SE-0726-SM-2XCD7
Thyroid	Thyroid	GTEX-S7SF-0226-SM-5SI7H
Thyroid	Thyroid	GTEX-SE5C-0726-SM-4BRWY
Thyroid	Thyroid	GTEX-SIU8-0626-SM-2XCDN
Thyroid	Thyroid	GTEX-SJXC-0726-SM-2XCFJ
Thyroid	Thyroid	GTEX-SN8G-1526-SM-4DM79
Thyroid	Thyroid	GTEX-SNOS-0226-SM-32PLR
Thyroid	Thyroid	GTEX-T2IS-0626-SM-32QP6
Thyroid	Thyroid	GTEX-T5JW-1226-SM-3GACY
Thyroid	Thyroid	GTEX-T6MN-0626-SM-32PM9
Thyroid	Thyroid	GTEX-T6MO-0226-SM-32QOL
Thyroid	Thyroid	GTEX-T8EM-0226-SM-3DB7C
Thyroid	Thyroid	GTEX-TKQ1-0126-SM-33HB3
Thyroid	Thyroid	GTEX-TMMY-0826-SM-33HB9
Thyroid	Thyroid	GTEX-TSE9-0626-SM-3DB8B
Thyroid	Thyroid	GTEX-U3ZM-0126-SM-3DB8M
Thyroid	Thyroid	GTEX-U3ZN-0326-SM-3DB86
Thyroid	Thyroid	GTEX-U4B1-0626-SM-3DB8L
Thyroid	Thyroid	GTEX-U8T8-2326-SM-3DB96
Thyroid	Thyroid	GTEX-UJMC-0326-SM-3GAE2
Thyroid	Thyroid	GTEX-V1D1-0926-SM-4JBHQ
Thyroid	Thyroid	GTEX-V955-0426-SM-3GAEL
Thyroid	Thyroid	GTEX-VJYA-0426-SM-3GIJK

Thyroid	Thyroid	GTEX-VUSG-0426-SM-3GIKD
Thyroid	Thyroid	GTEX-W5WG-1426-SM-4KKZP
Thyroid	Thyroid	GTEX-W5X1-0426-SM-3GILB
Thyroid	Thyroid	GTEX-WEY5-0526-SM-3GIKZ
Thyroid	Thyroid	GTEX-WFG7-0326-SM-5SI7L
Thyroid	Thyroid	GTEX-WFG8-0426-SM-3GILD
Thyroid	Thyroid	GTEX-WFJO-0226-SM-3GIKW
Thyroid	Thyroid	GTEX-WH7G-0526-SM-3NMBI
Thyroid	Thyroid	GTEX-WHPG-0226-SM-3NMB9
Thyroid	Thyroid	GTEX-WHSB-1626-SM-3LK6J
Thyroid	Thyroid	GTEX-WHSE-0626-SM-4RGNF
Thyroid	Thyroid	GTEX-WK11-0926-SM-3NMAU
Thyroid	Thyroid	GTEX-WL46-0126-SM-3TW8I
Thyroid	Thyroid	GTEX-WOFL-0726-SM-3MJG4
Thyroid	Thyroid	GTEX-WRHU-0926-SM-4E3IG
Thyroid	Thyroid	GTEX-WVLH-0626-SM-3MJG7
Thyroid	Thyroid	GTEX-WWYW-0826-SM-3NB2X
Thyroid	Thyroid	GTEX-WXYG-0226-SM-3NB2Y
Thyroid	Thyroid	GTEX-WY7C-0226-SM-3NB37
Thyroid	Thyroid	GTEX-WYBS-1926-SM-3NM8N
Thyroid	Thyroid	GTEX-WYJK-1626-SM-3NM9J
Thyroid	Thyroid	GTEX-WYVS-0326-SM-3NM9V
Thyroid	Thyroid	GTEX-X15G-0526-SM-3NMB7
Thyroid	Thyroid	GTEX-X4LF-0426-SM-3NMB5
Thyroid	Thyroid	GTEX-X4XX-0926-SM-46MV7
Thyroid	Thyroid	GTEX-X4XY-0826-SM-4E3JM
Thyroid	Thyroid	GTEX-X5EB-0726-SM-46MVR
Thyroid	Thyroid	GTEX-X8HC-0726-SM-46MWG
Thyroid	Thyroid	GTEX-XBED-0126-SM-47JY7
Thyroid	Thyroid	GTEX-XBEW-0126-SM-4AT66
Thyroid	Thyroid	GTEX-XGQ4-0426-SM-4AT4I
Thyroid	Thyroid	GTEX-XLM4-0726-SM-4AT64
Thyroid	Thyroid	GTEX-XMK1-0626-SM-4B65A
Thyroid	Thyroid	GTEX-XUW1-1026-SM-4BONY
Thyroid	Thyroid	GTEX-XUZC-0126-SM-4BOO6
Thyroid	Thyroid	GTEX-XV7Q-0326-SM-4BRVM
Thyroid	Thyroid	GTEX-XXEK-1326-SM-4BRV1
Thyroid	Thyroid	GTEX-XYKS-0826-SM-4BRVF
Thyroid	Thyroid	GTEX-Y111-1926-SM-4SOIS
Thyroid	Thyroid	GTEX-Y114-0626-SM-4TT98
Thyroid	Thyroid	GTEX-Y3I4-0226-SM-4TT27
Thyroid	Thyroid	GTEX-Y3IK-0526-SM-4WWE3
Thyroid	Thyroid	GTEX-Y5LM-0626-SM-4V6G4
Thyroid	Thyroid	GTEX-Y5V5-0326-SM-5RQJG
Thyroid	Thyroid	GTEX-Y5V6-0526-SM-4VBRV
Thyroid	Thyroid	GTEX-Y8E4-0126-SM-4VBQ2
Thyroid	Thyroid	GTEX-Y9LG-0426-SM-4VBRT
Thyroid	Thyroid	GTEX-YB5E-0626-SM-4VDSE
Thyroid	Thyroid	GTEX-YB5K-0526-SM-5LUAS
Thyroid	Thyroid	GTEX-YEC3-0826-SM-4WWFP
Thyroid	Thyroid	GTEX-YEC4-0626-SM-5CVLU
Thyroid	Thyroid	GTEX-YF7O-0726-SM-4W213
Thyroid	Thyroid	GTEX-YFC4-2626-SM-5P9FQ
Thyroid	Thyroid	GTEX-YFCO-0326-SM-4W1ZP

Thyroid	Thyroid	GTEX-YJ89-0726-SM-5P9F7
Thyroid	Thyroid	GTEX-Z9EW-0226-SM-5CVM7
Thyroid	Thyroid	GTEX-ZA64-0426-SM-5HL96
Thyroid	Thyroid	GTEX-ZAB5-0726-SM-5P9JG
Thyroid	Thyroid	GTEX-ZAJG-0726-SM-5HL9A
Thyroid	Thyroid	GTEX-ZAK1-0726-SM-5HL8Q
Thyroid	Thyroid	GTEX-ZC5H-0626-SM-5LU9K
Thyroid	Thyroid	GTEX-ZDTS-0926-SM-5YY9D
Thyroid	Thyroid	GTEX-ZDYS-0626-SM-5J2N5
Thyroid	Thyroid	GTEX-ZE7O-1126-SM-57WC8
Thyroid	Thyroid	GTEX-ZF28-0826-SM-4WKGJ
Thyroid	Thyroid	GTEX-ZGAY-1026-SM-4WWBR
Thyroid	Thyroid	GTEX-ZLFU-0626-SM-4WWBO
Thyroid	Thyroid	GTEX-ZLV1-0126-SM-4WWBZ
Thyroid	Thyroid	GTEX-ZLWG-0526-SM-4WWFB
Thyroid	Thyroid	GTEX-ZPCL-0126-SM-4WWC8
Thyroid	Thyroid	GTEX-ZPU1-0426-SM-4WWCA
Thyroid	Thyroid	GTEX-ZQG8-0926-SM-57WFF
Thyroid	Thyroid	GTEX-ZQUD-0126-SM-7EPIS
Thyroid	Thyroid	GTEX-ZT9W-0226-SM-4YCCZ
Thyroid	Thyroid	GTEX-ZT9X-0226-SM-51MT2
Thyroid	Thyroid	GTEX-ZTPG-0826-SM-5DUVC
Thyroid	Thyroid	GTEX-ZTSS-0226-SM-59877
Thyroid	Thyroid	GTEX-ZTX8-0626-SM-59HKC
Thyroid	Thyroid	GTEX-ZUA1-0926-SM-4YCDX
Thyroid	Thyroid	GTEX-ZV6S-0226-SM-59HJT
Thyroid	Thyroid	GTEX-ZV7C-0126-SM-57WDE
Thyroid	Thyroid	GTEX-ZVP2-0426-SM-57WC2
Thyroid	Thyroid	GTEX-ZVT3-0726-SM-5GICN
Thyroid	Thyroid	GTEX-ZVZP-1026-SM-5GICI
Thyroid	Thyroid	GTEX-ZVZQ-0626-SM-59HJU
Thyroid	Thyroid	GTEX-ZXG5-0926-SM-5NQ8H
Thyroid	Thyroid	GTEX-ZY6K-0226-SM-5SIAY
Thyroid	Thyroid	GTEX-ZYFC-0926-SM-5GZWW
Thyroid	Thyroid	GTEX-ZYFD-0826-SM-5NQ9A
Thyroid	Thyroid	GTEX-ZYFG-0626-SM-5GZYA
Thyroid	Thyroid	GTEX-ZYT6-0426-SM-5GID3
Thyroid	Thyroid	GTEX-ZYVF-1126-SM-5E458
Thyroid	Thyroid	GTEX-ZYW4-1126-SM-5SI99
Thyroid	Thyroid	GTEX-ZYY3-1926-SM-5GZXS
Thyroid	Thyroid	GTEX-ZZ64-0126-SM-5GZXA
Thyroid	Thyroid	GTEX-ZZPU-1326-SM-5GZWS
Uterus	Uterus	GTEX-1117F-2426-SM-5EGGH
Uterus	Uterus	GTEX-113JC-2226-SM-5EGJG
Uterus	Uterus	GTEX-11DXX-1526-SM-5H115
Uterus	Uterus	GTEX-11EM3-1926-SM-5987U
Uterus	Uterus	GTEX-11EMC-1826-SM-5A5JT
Uterus	Uterus	GTEX-11GSP-2426-SM-5N9BD
Uterus	Uterus	GTEX-11I78-2126-SM-5A5K8
Uterus	Uterus	GTEX-11P81-1626-SM-5BC52
Uterus	Uterus	GTEX-11ZTS-2326-SM-5EQMY
Uterus	Uterus	GTEX-11ZTT-1726-SM-5EQL4
Uterus	Uterus	GTEX-12WSD-2826-SM-59HKT
Uterus	Uterus	GTEX-12WSG-2126-SM-5EGJ7

Uterus	Uterus	GTEX-12WSK-2026-SM-5CVNB
Uterus	Uterus	GTEX-12ZZX-2126-SM-5LZVL
Uterus	Uterus	GTEX-1313W-2826-SM-5P9G1
Uterus	Uterus	GTEX-131XG-2026-SM-5GCN5
Uterus	Uterus	GTEX-131YS-2326-SM-5IJFJ
Uterus	Uterus	GTEX-132AR-1526-SM-5KM1L
Uterus	Uterus	GTEX-1399S-2226-SM-5IFEW
Uterus	Uterus	GTEX-1399U-1326-SM-5IJET
Uterus	Uterus	GTEX-139D8-2526-SM-5N9G3
Uterus	Uterus	GTEX-13D11-1226-SM-5IFGA
Uterus	Uterus	GTEX-13FTX-1026-SM-5J2O5
Uterus	Uterus	GTEX-13N11-1126-SM-5KM41
Uterus	Uterus	GTEX-13OVI-1026-SM-5L3EM
Uterus	Uterus	GTEX-13OVJ-2326-SM-5IJGA
Uterus	Uterus	GTEX-13PL7-2026-SM-5IFGK
Uterus	Uterus	GTEX-13QBU-1626-SM-5LU4S
Uterus	Uterus	GTEX-13S7M-1726-SM-5RQK3
Uterus	Uterus	GTEX-13U4I-1926-SM-5LU39
Uterus	Uterus	GTEX-13VXT-1626-SM-5IJES
Uterus	Uterus	GTEX-13W3W-1426-SM-5LU56
Uterus	Uterus	GTEX-145LS-2426-SM-5TDCZ
Uterus	Uterus	GTEX-145ME-1326-SM-5O98Q
Uterus	Uterus	GTEX-14AS3-1826-SM-5TDD8
Uterus	Uterus	GTEX-14BIM-2226-SM-5SI8Y
Uterus	Uterus	GTEX-14BIN-1526-SM-73KZ7
Uterus	Uterus	GTEX-14BMU-1826-SM-5RQII
Uterus	Uterus	GTEX-14JG6-0826-SM-6AJB9
Uterus	Uterus	GTEX-14PHW-2426-SM-69LPU
Uterus	Uterus	GTEX-14PJ6-1526-SM-6AJ9M
Uterus	Uterus	GTEX-14PJM-2826-SM-69LPV
Uterus	Uterus	GTEX-14PKV-1426-SM-5YYB9
Uterus	Uterus	GTEX-15CHC-2026-SM-6EU1Z
Uterus	Uterus	GTEX-15DCZ-2226-SM-69LOB
Uterus	Uterus	GTEX-15DZA-1326-SM-68722
Uterus	Uterus	GTEX-15ER7-2426-SM-793B9
Uterus	Uterus	GTEX-15ETS-1526-SM-6LPKM
Uterus	Uterus	GTEX-15FZZ-1026-SM-6AJBI
Uterus	Uterus	GTEX-16A39-0726-SM-7KUEB
Uterus	Uterus	GTEX-16NGA-0626-SM-72D72
Uterus	Uterus	GTEX-1A32A-2826-SM-72D5S
Uterus	Uterus	GTEX-1A8FM-2326-SM-7MKGC
Uterus	Uterus	GTEX-1AMEY-0626-SM-72D6B
Uterus	Uterus	GTEX-1B932-2726-SM-731ER
Uterus	Uterus	GTEX-1B97J-0226-SM-79392
Uterus	Uterus	GTEX-1C475-0626-SM-73KV9
Uterus	Uterus	GTEX-1CAMS-2026-SM-79395
Uterus	Uterus	GTEX-1CB4H-2726-SM-7EWFF
Uterus	Uterus	GTEX-1GMR8-2426-SM-7MKH3
Uterus	Uterus	GTEX-1GN1U-2526-SM-7MKH4
Uterus	Uterus	GTEX-N7MT-0726-SM-3TW8S
Uterus	Uterus	GTEX-OHPK-2026-SM-3MJH7
Uterus	Uterus	GTEX-OHPL-2026-SM-3TW8R
Uterus	Uterus	GTEX-P4PP-2026-SM-3P61N
Uterus	Uterus	GTEX-P4QT-2026-SM-3NMCJ

Uterus	Uterus	GTEX-P78B-2526-SM-3P5ZY
Uterus	Uterus	GTEX-POMQ-1226-SM-3P61F
Uterus	Uterus	GTEX-PWCY-1426-SM-48TCT
Uterus	Uterus	GTEX-PWN1-2026-SM-48TD9
Uterus	Uterus	GTEX-PX3G-2026-SM-48U1H
Uterus	Uterus	GTEX-Q734-1626-SM-48U1B
Uterus	Uterus	GTEX-QCQG-1326-SM-48U24
Uterus	Uterus	GTEX-R55G-1626-SM-48FF4
Uterus	Uterus	GTEX-RTLS-2426-SM-46MUO
Uterus	Uterus	GTEX-RU1J-1026-SM-46MUR
Uterus	Uterus	GTEX-RU72-2626-SM-4TT75
Uterus	Uterus	GTEX-RWS6-1326-SM-47JXB
Uterus	Uterus	GTEX-S32W-1426-SM-4AD66
Uterus	Uterus	GTEX-S341-1026-SM-4AD71
Uterus	Uterus	GTEX-S4UY-1226-SM-4AD51
Uterus	Uterus	GTEX-S7SF-0826-SM-4AD4W
Uterus	Uterus	GTEX-T2IS-2226-SM-4DM65
Uterus	Uterus	GTEX-T5JW-1526-SM-4DM5E
Uterus	Uterus	GTEX-T6MO-1526-SM-4DM57
Uterus	Uterus	GTEX-TMMY-2226-SM-4DXTN
Uterus	Uterus	GTEX-TSE9-2626-SM-4DXV2
Uterus	Uterus	GTEX-U3ZN-0726-SM-4DXT5
Uterus	Uterus	GTEX-W5WG-1326-SM-4LMI9
Uterus	Uterus	GTEX-WEY5-0726-SM-4LMID
Uterus	Uterus	GTEX-WI4N-2426-SM-4OOSC
Uterus	Uterus	GTEX-WWYW-2826-SM-4MVOQ
Uterus	Uterus	GTEX-WXYG-1326-SM-4ONCN
Uterus	Uterus	GTEX-WYVS-1226-SM-4ONCL
Uterus	Uterus	GTEX-XUW1-0226-SM-4BOOS
Uterus	Uterus	GTEX-XUZC-0926-SM-4BOQF
Uterus	Uterus	GTEX-XV7Q-1526-SM-4BRWB
Uterus	Uterus	GTEX-XYKS-1626-SM-4BRUQ
Uterus	Uterus	GTEX-Y114-1826-SM-4TT87
Uterus	Uterus	GTEX-Y3IK-2126-SM-4YCDS
Uterus	Uterus	GTEX-YFC4-2826-SM-5P9FR
Uterus	Uterus	GTEX-YJ8O-1126-SM-5P9IS
Uterus	Uterus	GTEX-ZAJG-2826-SM-5HL8D
Uterus	Uterus	GTEX-ZAK1-2526-SM-5S2N7
Uterus	Uterus	GTEX-ZLWG-1026-SM-4WWC4
Uterus	Uterus	GTEX-ZP4G-0726-SM-4WWF2
Uterus	Uterus	GTEX-ZTPG-2026-SM-5O999
Uterus	Uterus	GTEX-ZV6S-1526-SM-5NQ7O
Uterus	Uterus	GTEX-ZVT2-0426-SM-5E44S
Uterus	Uterus	GTEX-ZXES-0726-SM-5E451
Uterus	Uterus	GTEX-ZYVF-0526-SM-5E43E
Vagina	Vagina	GTEX-1117F-2526-SM-5GZY6
Vagina	Vagina	GTEX-113JC-1926-SM-5N9GE
Vagina	Vagina	GTEX-11DXX-1726-SM-5H11T
Vagina	Vagina	GTEX-11EM3-1826-SM-5A5KO
Vagina	Vagina	GTEX-11EMC-1926-SM-5A5JU
Vagina	Vagina	GTEX-11GSP-2226-SM-5HL5Q
Vagina	Vagina	GTEX-11I78-2026-SM-5987C
Vagina	Vagina	GTEX-11P81-2126-SM-5HL6N
Vagina	Vagina	GTEX-11TTK-2726-SM-5GU58

Vagina	Vagina	GTEX-11VI4-0726-SM-5GU5B
Vagina	Vagina	GTEX-11XUK-1326-SM-5EQM4
Vagina	Vagina	GTEX-12WSD-2926-SM-5LZUH
Vagina	Vagina	GTEX-12WSJ-1326-SM-5GCOB
Vagina	Vagina	GTEX-12ZZX-1926-SM-5EGKC
Vagina	Vagina	GTEX-131XG-2126-SM-5DUXG
Vagina	Vagina	GTEX-131YS-2426-SM-5J2OD
Vagina	Vagina	GTEX-1399S-2326-SM-5K7YV
Vagina	Vagina	GTEX-1399U-1526-SM-5J2N7
Vagina	Vagina	GTEX-13D11-1326-SM-5IJFY
Vagina	Vagina	GTEX-13N11-1526-SM-5L3FL
Vagina	Vagina	GTEX-13OVI-0926-SM-5J1MI
Vagina	Vagina	GTEX-13PL7-2726-SM-5L3GB
Vagina	Vagina	GTEX-13PVR-1426-SM-5S2PW
Vagina	Vagina	GTEX-13QBU-1726-SM-5IJFQ
Vagina	Vagina	GTEX-13QIC-1826-SM-5J1MY
Vagina	Vagina	GTEX-13U4I-2026-SM-5LU3L
Vagina	Vagina	GTEX-13W3W-2326-SM-5LU4Y
Vagina	Vagina	GTEX-145LS-2526-SM-5TDC9
Vagina	Vagina	GTEX-145ME-1426-SM-5RQJS
Vagina	Vagina	GTEX-146FH-2626-SM-5Q5E1
Vagina	Vagina	GTEX-146FR-2426-SM-5NQAU
Vagina	Vagina	GTEX-147F3-1526-SM-5SIAK
Vagina	Vagina	GTEX-14A5I-2726-SM-5SIB3
Vagina	Vagina	GTEX-14BMU-1926-SM-5S2QC
Vagina	Vagina	GTEX-14DAQ-2326-SM-5S2QX
Vagina	Vagina	GTEX-14H4A-2426-SM-664OQ
Vagina	Vagina	GTEX-14JG6-0726-SM-69LQH
Vagina	Vagina	GTEX-14PJ6-1626-SM-6872D
Vagina	Vagina	GTEX-14PJM-2926-SM-69LOE
Vagina	Vagina	GTEX-14PKU-1326-SM-6871B
Vagina	Vagina	GTEX-14PN3-1626-SM-6AJAV
Vagina	Vagina	GTEX-15DCZ-2326-SM-6AJB1
Vagina	Vagina	GTEX-15DYW-2926-SM-6PANA
Vagina	Vagina	GTEX-15ER7-2526-SM-7KUFW
Vagina	Vagina	GTEX-15SB6-1426-SM-7KUEM
Vagina	Vagina	GTEX-15SDE-1726-SM-6LPJS
Vagina	Vagina	GTEX-15SHU-2926-SM-7KUKT
Vagina	Vagina	GTEX-15UF6-2126-SM-6M46Z
Vagina	Vagina	GTEX-16NGA-1626-SM-72D6M
Vagina	Vagina	GTEX-16NPV-2526-SM-6M483
Vagina	Vagina	GTEX-17F9Y-2326-SM-7DUFP
Vagina	Vagina	GTEX-17JCI-2526-SM-7DHLB
Vagina	Vagina	GTEX-183WM-2126-SM-7LG4I
Vagina	Vagina	GTEX-18A7A-2826-SM-7LT8J
Vagina	Vagina	GTEX-18D9A-1026-SM-7KFRU
Vagina	Vagina	GTEX-1A8FM-2426-SM-7IGPE
Vagina	Vagina	GTEX-1AMFI-1526-SM-73KVY
Vagina	Vagina	GTEX-1C475-0526-SM-793AC
Vagina	Vagina	GTEX-1CAMS-2326-SM-793CR
Vagina	Vagina	GTEX-1EWIQ-2326-SM-7IGNF
Vagina	Vagina	GTEX-N7MT-1926-SM-3LK5N
Vagina	Vagina	GTEX-NL3G-2526-SM-4SOIE
Vagina	Vagina	GTEX-OHPK-2526-SM-3MJH9

Vagina	Vagina	GTEX-OHPL-2526-SM-3MJGT
Vagina	Vagina	GTEX-P4PP-2526-SM-3P61P
Vagina	Vagina	GTEX-P78B-2226-SM-3P5ZZ
Vagina	Vagina	GTEX-PLZ4-2726-SM-3P61A
Vagina	Vagina	GTEX-PWCY-1726-SM-48TD3
Vagina	Vagina	GTEX-PWN1-2526-SM-48TDS
Vagina	Vagina	GTEX-PX3G-2526-SM-48TZV
Vagina	Vagina	GTEX-Q734-1426-SM-48TZQ
Vagina	Vagina	GTEX-QCQG-1226-SM-48U23
Vagina	Vagina	GTEX-QDT8-2526-SM-48TYX
Vagina	Vagina	GTEX-QVJO-2526-SM-4R1KB
Vagina	Vagina	GTEX-RU1J-1426-SM-46MUV
Vagina	Vagina	GTEX-RWS6-1726-SM-47JXP
Vagina	Vagina	GTEX-S341-1226-SM-4AD5S
Vagina	Vagina	GTEX-S4P3-1126-SM-4AD52
Vagina	Vagina	GTEX-S4UY-1326-SM-4AD4X
Vagina	Vagina	GTEX-S7SF-1426-SM-4AT5A
Vagina	Vagina	GTEX-SE5C-1126-SM-4BRWZ
Vagina	Vagina	GTEX-T2IS-1926-SM-4DM74
Vagina	Vagina	GTEX-T6MO-1226-SM-4DM5S
Vagina	Vagina	GTEX-TML8-1126-SM-4DXSS
Vagina	Vagina	GTEX-TMMY-1926-SM-4DXUU
Vagina	Vagina	GTEX-TSE9-2526-SM-4DXUS
Vagina	Vagina	GTEX-U3ZN-0926-SM-4DXTU
Vagina	Vagina	GTEX-UJHI-1326-SM-4IHJO
Vagina	Vagina	GTEX-W5WG-1026-SM-4LMIF
Vagina	Vagina	GTEX-WEY5-0826-SM-4LMIH
Vagina	Vagina	GTEX-WRHU-2926-SM-4MVNQ
Vagina	Vagina	GTEX-WYVS-1426-SM-4ONCV
Vagina	Vagina	GTEX-X15G-1926-SM-4PQZQ
Vagina	Vagina	GTEX-X8HC-2826-SM-46MWJ
Vagina	Vagina	GTEX-XUW1-0326-SM-4BOOR
Vagina	Vagina	GTEX-XV7Q-1626-SM-4BRWC
Vagina	Vagina	GTEX-Y114-1926-SM-4TT8J
Vagina	Vagina	GTEX-Y3IK-2226-SM-4YCD4
Vagina	Vagina	GTEX-Y8LW-1426-SM-5RQJJ
Vagina	Vagina	GTEX-YFC4-2926-SM-5IFJ8
Vagina	Vagina	GTEX-YJ8O-1226-SM-5P9JE
Vagina	Vagina	GTEX-ZC5H-2226-SM-4WAWS
Vagina	Vagina	GTEX-ZE7O-2426-SM-57WD7
Vagina	Vagina	GTEX-ZGAY-2226-SM-4YCCX
Vagina	Vagina	GTEX-ZLWG-1126-SM-4WWFQ
Vagina	Vagina	GTEX-ZP4G-0826-SM-4WWF3
Vagina	Vagina	GTEX-ZQG8-1126-SM-51MRL
Vagina	Vagina	GTEX-ZTPG-2226-SM-57WF9
Vagina	Vagina	GTEX-ZV6S-1326-SM-59HJA
Vagina	Vagina	GTEX-ZVT2-0626-SM-5GICR
Vagina	Vagina	GTEX-ZVT3-2726-SM-5GU5X
Vagina	Vagina	GTEX-ZXES-0626-SM-5E45P
Vagina	Vagina	GTEX-ZYVF-0326-SM-5GIES
Vagina	Vagina	GTEX-ZYY3-2626-SM-5N9DH
Vagina	Vagina	GTEX-ZZPU-2226-SM-5EGIV
Whole Blood	Blood	GTEX-111YS-0006-SM-5NQBE
Whole Blood	Blood	GTEX-1122O-0005-SM-5O99J

Whole Blood	Blood	GTEX-1128S-0005-SM-5P9HI
Whole Blood	Blood	GTEX-113IC-0006-SM-5NQ9C
Whole Blood	Blood	GTEX-113JC-0006-SM-5O997
Whole Blood	Blood	GTEX-117XS-0005-SM-5PNU6
Whole Blood	Blood	GTEX-117YW-0005-SM-5NQ8Z
Whole Blood	Blood	GTEX-1192W-0005-SM-5NQBQ
Whole Blood	Blood	GTEX-1192X-0005-SM-5NQC3
Whole Blood	Blood	GTEX-11DXW-0006-SM-5NQ7Y
Whole Blood	Blood	GTEX-11DXX-0005-SM-5NQ8B
Whole Blood	Blood	GTEX-11DXY-0006-SM-5NQ8N
Whole Blood	Blood	GTEX-11DXZ-0006-SM-5LZZH
Whole Blood	Blood	GTEX-11DYG-0006-SM-5NQB2
Whole Blood	Blood	GTEX-11EI6-0005-SM-5N9GN
Whole Blood	Blood	GTEX-11EM3-0005-SM-5N9DK
Whole Blood	Blood	GTEX-11EMC-0006-SM-5O9DN
Whole Blood	Blood	GTEX-11EQ8-0006-SM-5P9HJ
Whole Blood	Blood	GTEX-11EQ9-0006-SM-5LUB6
Whole Blood	Blood	GTEX-11GS4-0006-SM-5SI9M
Whole Blood	Blood	GTEX-11GSO-0005-SM-5O9CA
Whole Blood	Blood	GTEX-11GSP-0006-SM-5N9EL
Whole Blood	Blood	GTEX-11I78-0005-SM-5N9GB
Whole Blood	Blood	GTEX-11LCK-0005-SM-5O98U
Whole Blood	Blood	GTEX-11NSD-0005-SM-5LZZ5
Whole Blood	Blood	GTEX-11NUK-0005-SM-5MR5F
Whole Blood	Blood	GTEX-11NV4-0005-SM-5N9EX
Whole Blood	Blood	GTEX-11O72-0006-SM-5O9DB
Whole Blood	Blood	GTEX-11OC5-0006-SM-5N9FA
Whole Blood	Blood	GTEX-11OF3-0006-SM-5O9CM
Whole Blood	Blood	GTEX-11ONC-0005-SM-5O9CY
Whole Blood	Blood	GTEX-11P7K-0006-SM-5N9FM
Whole Blood	Blood	GTEX-11P81-0006-SM-5NQ7A
Whole Blood	Blood	GTEX-11P82-0006-SM-5N9FY
Whole Blood	Blood	GTEX-11PRG-0005-SM-5NQ6X
Whole Blood	Blood	GTEX-11TT1-0005-SM-5NQ8Y
Whole Blood	Blood	GTEX-11TTK-0005-SM-5O9BX
Whole Blood	Blood	GTEX-11TUW-0006-SM-5LZW2
Whole Blood	Blood	GTEX-11UD2-0005-SM-5NQ9B
Whole Blood	Blood	GTEX-11VI4-0006-SM-5N9D8
Whole Blood	Blood	GTEX-11WQC-0006-SM-5LZVP
Whole Blood	Blood	GTEX-11WQK-0005-SM-5O9AV
Whole Blood	Blood	GTEX-11XUK-0005-SM-5LU9F
Whole Blood	Blood	GTEX-11ZTS-0005-SM-5NQ8A
Whole Blood	Blood	GTEX-11ZTT-0006-SM-5N9FX
Whole Blood	Blood	GTEX-11ZU8-0005-SM-5PNU5
Whole Blood	Blood	GTEX-11ZUS-0006-SM-5O9AJ
Whole Blood	Blood	GTEX-11ZVC-0006-SM-5N9GA
Whole Blood	Blood	GTEX-1211K-0006-SM-5MR53
Whole Blood	Blood	GTEX-12126-0006-SM-5O9B8
Whole Blood	Blood	GTEX-1212Z-0006-SM-5NQ8M
Whole Blood	Blood	GTEX-12584-0005-SM-5N9GM
Whole Blood	Blood	GTEX-12696-0005-SM-5LZYS
Whole Blood	Blood	GTEX-1269C-0005-SM-5N9CJ
Whole Blood	Blood	GTEX-12BJ1-0006-SM-5SIB5
Whole Blood	Blood	GTEX-12C56-0006-SM-5N9E9

Whole Blood	Blood	GTEX-12KS4-0005-SM-5SI94
Whole Blood	Blood	GTEX-12WSA-0005-SM-5O9BM
Whole Blood	Blood	GTEX-12WSC-0005-SM-5MR3H
Whole Blood	Blood	GTEX-12WSD-0005-SM-5N9FZ
Whole Blood	Blood	GTEX-12WSE-0005-SM-5NQAE
Whole Blood	Blood	GTEX-12WSF-0005-SM-5NQAQ
Whole Blood	Blood	GTEX-12WSG-0005-SM-5NQA2
Whole Blood	Blood	GTEX-12WSH-0005-SM-5NQ9P
Whole Blood	Blood	GTEX-12WSI-0005-SM-5O99K
Whole Blood	Blood	GTEX-12WSJ-0005-SM-5LU8S
Whole Blood	Blood	GTEX-12WSK-0006-SM-5NQA1
Whole Blood	Blood	GTEX-12WSL-0005-SM-5NQAD
Whole Blood	Blood	GTEX-12WSM-0005-SM-5NQB3
Whole Blood	Blood	GTEX-12WSN-0006-SM-5NQAP
Whole Blood	Blood	GTEX-12ZZW-0005-SM-5O9BY
Whole Blood	Blood	GTEX-12ZZX-0005-SM-5O9A9
Whole Blood	Blood	GTEX-13111-0005-SM-5NQ7Z
Whole Blood	Blood	GTEX-13113-0006-SM-5NQ7X
Whole Blood	Blood	GTEX-1313W-0006-SM-5O9B1
Whole Blood	Blood	GTEX-1314G-0005-SM-5NQ9O
Whole Blood	Blood	GTEX-131XE-0006-SM-5P9F9
Whole Blood	Blood	GTEX-131XF-0006-SM-5P9HK
Whole Blood	Blood	GTEX-131XG-0006-SM-5O9CE
Whole Blood	Blood	GTEX-131XH-0006-SM-5O98V
Whole Blood	Blood	GTEX-131XW-0006-SM-5O9AO
Whole Blood	Blood	GTEX-131YS-0005-SM-5NQAS
Whole Blood	Blood	GTEX-132AR-0006-SM-5NQ7N
Whole Blood	Blood	GTEX-132NY-0005-SM-5O9AC
Whole Blood	Blood	GTEX-132Q8-0006-SM-5NQA4
Whole Blood	Blood	GTEX-132QS-0005-SM-5LZXK
Whole Blood	Blood	GTEX-1339X-0005-SM-5O9CQ
Whole Blood	Blood	GTEX-1399R-0006-SM-5N9FR
Whole Blood	Blood	GTEX-1399S-0006-SM-5NQ7U
Whole Blood	Blood	GTEX-1399U-0005-SM-5NQ8K
Whole Blood	Blood	GTEX-139D8-0006-SM-5LZX8
Whole Blood	Blood	GTEX-139T6-0006-SM-5LU92
Whole Blood	Blood	GTEX-139T8-0006-SM-5NQ6V
Whole Blood	Blood	GTEX-139TS-0005-SM-5NQC1
Whole Blood	Blood	GTEX-139TT-0006-SM-5O9CG
Whole Blood	Blood	GTEX-139TU-0006-SM-5LZVN
Whole Blood	Blood	GTEX-139UW-0005-SM-5NQ8U
Whole Blood	Blood	GTEX-13CF2-0006-SM-5O99L
Whole Blood	Blood	GTEX-13CF3-0006-SM-5N9ED
Whole Blood	Blood	GTEX-13CIG-0006-SM-5P9HF
Whole Blood	Blood	GTEX-13CZU-0005-SM-5LU8K
Whole Blood	Blood	GTEX-13CZV-0006-SM-5P9IN
Whole Blood	Blood	GTEX-13FTW-0005-SM-5LZVZ
Whole Blood	Blood	GTEX-13FTX-0005-SM-5N9F6
Whole Blood	Blood	GTEX-13FTY-0005-SM-5NQ78
Whole Blood	Blood	GTEX-13FTZ-0005-SM-5NQ9N
Whole Blood	Blood	GTEX-13FXS-0006-SM-5O99X
Whole Blood	Blood	GTEX-13G51-0005-SM-5N9EP
Whole Blood	Blood	GTEX-13JUV-0006-SM-5NQ97
Whole Blood	Blood	GTEX-13O61-0005-SM-5O9AU

Whole Blood	Blood	GTEX-13OVG-0005-SM-5P9HA
Whole Blood	Blood	GTEX-13OVH-0005-SM-5P9HB
Whole Blood	Blood	GTEX-13OVI-0001-SM-5O9BL
Whole Blood	Blood	GTEX-13OVJ-0006-SM-5O98T
Whole Blood	Blood	GTEX-13OVK-0006-SM-5O9B7
Whole Blood	Blood	GTEX-13OVL-0006-SM-5O996
Whole Blood	Blood	GTEX-13OW6-0005-SM-5NQ9Z
Whole Blood	Blood	GTEX-13OW8-0005-SM-5NQAC
Whole Blood	Blood	GTEX-13PL7-0005-SM-5N9ET
Whole Blood	Blood	GTEX-13PLJ-0005-SM-5NQ99
Whole Blood	Blood	GTEX-13S7M-0005-SM-5NQ76
Whole Blood	Blood	GTEX-13S86-0005-SM-5NQ7I
Whole Blood	Blood	GTEX-13U4I-0006-SM-5N9CI
Whole Blood	Blood	GTEX-13VXT-0005-SM-5N9F3
Whole Blood	Blood	GTEX-13VXU-0006-SM-5N9FF
Whole Blood	Blood	GTEX-13W3W-0005-SM-5SI9Y
Whole Blood	Blood	GTEX-1477Z-0005-SM-5PNWD
Whole Blood	Blood	GTEX-147F3-0005-SM-5N9FI
Whole Blood	Blood	GTEX-147F4-0006-SM-5O99T
Whole Blood	Blood	GTEX-147GR-0005-SM-5NQAO
Whole Blood	Blood	GTEX-147JS-0006-SM-5NQ7K
Whole Blood	Blood	GTEX-148VI-0006-SM-5O9A6
Whole Blood	Blood	GTEX-148VJ-0006-SM-5NQB1
Whole Blood	Blood	GTEX-1497J-0005-SM-5NQBD
Whole Blood	Blood	GTEX-14A5H-0006-SM-5O9AI
Whole Blood	Blood	GTEX-14A5I-0005-SM-5N9FU
Whole Blood	Blood	GTEX-14A6H-0006-SM-5NQ7W
Whole Blood	Blood	GTEX-14ABY-0005-SM-5O99U
Whole Blood	Blood	GTEX-14AS3-0006-SM-5NQC2
Whole Blood	Blood	GTEX-14B4R-0006-SM-5O9A7
Whole Blood	Blood	GTEX-14BIL-0006-SM-5N9F2
Whole Blood	Blood	GTEX-14BIM-0006-SM-5O9AA
Whole Blood	Blood	GTEX-14BIN-0005-SM-5N9FE
Whole Blood	Blood	GTEX-14BMU-0006-SM-5MR3T
Whole Blood	Blood	GTEX-14BMV-0005-SM-5NQ6Y
Whole Blood	Blood	GTEX-14C38-0006-SM-5NQBF
Whole Blood	Blood	GTEX-14C39-0005-SM-5NQBR
Whole Blood	Blood	GTEX-14C5O-0005-SM-5P9ER
Whole Blood	Blood	GTEX-14DAQ-0005-SM-5NQ7B
Whole Blood	Blood	GTEX-14DAR-0006-SM-5N9GC
Whole Blood	Blood	GTEX-14E1K-0006-SM-5N9DY
Whole Blood	Blood	GTEX-14E6C-0005-SM-5O98N
Whole Blood	Blood	GTEX-14E6D-0005-SM-5N9D7
Whole Blood	Blood	GTEX-14E6E-0006-SM-5MR5N
Whole Blood	Blood	GTEX-14E7W-0006-SM-5N9GG
Whole Blood	Blood	GTEX-14H4A-0006-SM-5N9E3
Whole Blood	Blood	GTEX-14ICK-0006-SM-5NQB5
Whole Blood	Blood	GTEX-14ICL-0006-SM-5SIAB
Whole Blood	Blood	GTEX-15RIE-0006-SM-7MKF8
Whole Blood	Blood	GTEX-16NPX-0005-SM-7MKGG
Whole Blood	Blood	GTEX-16XZZ-0006-SM-7MKF5
Whole Blood	Blood	GTEX-17GQL-0005-SM-7MKF4
Whole Blood	Blood	GTEX-17HG3-0006-SM-7MKFE
Whole Blood	Blood	GTEX-17HII-0005-SM-7MKF6

Whole Blood	Blood	GTEX-1A3MW-0006-SM-7MKGU
Whole Blood	Blood	GTEX-1B8SG-0006-SM-7MKFA
Whole Blood	Blood	GTEX-1C2JI-0006-SM-7MKFD
Whole Blood	Blood	GTEX-1C4CL-0005-SM-7MKF3
Whole Blood	Blood	GTEX-1C6VR-0005-SM-7MKF7
Whole Blood	Blood	GTEX-N7MS-0007-SM-2D7W1
Whole Blood	Blood	GTEX-N7MT-0007-SM-3GACQ
Whole Blood	Blood	GTEX-NFK9-0006-SM-3GACS
Whole Blood	Blood	GTEX-NL3G-0007-SM-4SOIF
Whole Blood	Blood	GTEX-NPJ7-0006-SM-3GACR
Whole Blood	Blood	GTEX-NPJ8-0007-SM-2D7VX
Whole Blood	Blood	GTEX-O5YT-0007-SM-32PK7
Whole Blood	Blood	GTEX-O5YW-0006-SM-3LK6E
Whole Blood	Blood	GTEX-OHPK-0006-SM-2HMKH
Whole Blood	Blood	GTEX-OHPL-0006-SM-3MJHB
Whole Blood	Blood	GTEX-OHPM-0006-SM-2HMKU
Whole Blood	Blood	GTEX-OHPN-0005-SM-2YUML
Whole Blood	Blood	GTEX-OIZF-0006-SM-2I5GQ
Whole Blood	Blood	GTEX-OIZH-0005-SM-2HMJN
Whole Blood	Blood	GTEX-OIZI-0005-SM-2XCED
Whole Blood	Blood	GTEX-OOBJ-0006-SM-2I3F4
Whole Blood	Blood	GTEX-OOBK-0005-SM-2YUMG
Whole Blood	Blood	GTEX-OXRL-0005-SM-3LK6A
Whole Blood	Blood	GTEX-OXRO-0006-SM-2I5EM
Whole Blood	Blood	GTEX-OXRP-0006-SM-2I3FN
Whole Blood	Blood	GTEX-P44H-0006-SM-2XCFB
Whole Blood	Blood	GTEX-P4PP-0005-SM-2HMKX
Whole Blood	Blood	GTEX-P4PQ-0005-SM-2HMKJ
Whole Blood	Blood	GTEX-P4QS-0005-SM-2I3EY
Whole Blood	Blood	GTEX-P78B-0005-SM-2I5GM
Whole Blood	Blood	GTEX-PLZ4-0006-SM-5SI8L
Whole Blood	Blood	GTEX-PLZ5-0006-SM-5S2W5
Whole Blood	Blood	GTEX-PLZ6-0006-SM-33HBZ
Whole Blood	Blood	GTEX-POMQ-0006-SM-5SI7D
Whole Blood	Blood	GTEX-PSDG-0005-SM-3GADC
Whole Blood	Blood	GTEX-PVOW-0006-SM-3NMB8
Whole Blood	Blood	GTEX-PW2O-0006-SM-2I3DV
Whole Blood	Blood	GTEX-PWCY-0005-SM-33HBP
Whole Blood	Blood	GTEX-PWN1-0006-SM-5SI7T
Whole Blood	Blood	GTEX-PX3G-0006-SM-5SI7E
Whole Blood	Blood	GTEX-Q2AG-0005-SM-5SI7F
Whole Blood	Blood	GTEX-Q2AH-0005-SM-33HBR
Whole Blood	Blood	GTEX-Q2AI-0006-SM-2I3FG
Whole Blood	Blood	GTEX-Q734-0006-SM-2I3FJ
Whole Blood	Blood	GTEX-QCQG-0006-SM-5SI8M
Whole Blood	Blood	GTEX-QDT8-0006-SM-5SI8N
Whole Blood	Blood	GTEX-QDVJ-0005-SM-2TC5X
Whole Blood	Blood	GTEX-QDVN-0006-SM-48U1R
Whole Blood	Blood	GTEX-QEG4-0006-SM-2I5FY
Whole Blood	Blood	GTEX-QEG5-0006-SM-2I5FZ
Whole Blood	Blood	GTEX-QESD-0006-SM-2I5G6
Whole Blood	Blood	GTEX-QLQ7-0005-SM-2S1QP
Whole Blood	Blood	GTEX-QLQW-0005-SM-2S1RA
Whole Blood	Blood	GTEX-QMRM-0005-SM-3NB2A

Whole Blood	Blood	GTEX-QV31-0006-SM-793B5
Whole Blood	Blood	GTEX-QVJO-0006-SM-2S1RC
Whole Blood	Blood	GTEX-QVUS-0006-SM-3GAE8
Whole Blood	Blood	GTEX-QXCU-0006-SM-2TC5K
Whole Blood	Blood	GTEX-R3RS-0005-SM-3GAEH
Whole Blood	Blood	GTEX-R45C-0006-SM-3GAD6
Whole Blood	Blood	GTEX-R53T-0005-SM-3GADK
Whole Blood	Blood	GTEX-R55C-0005-SM-3GAE9
Whole Blood	Blood	GTEX-R55D-0006-SM-3GIJS
Whole Blood	Blood	GTEX-R55E-0006-SM-2TC5G
Whole Blood	Blood	GTEX-R55G-0006-SM-2TC6O
Whole Blood	Blood	GTEX-REY6-0005-SM-2TF54
Whole Blood	Blood	GTEX-RM2N-0006-SM-2TF5H
Whole Blood	Blood	GTEX-RN64-0005-SM-793B4
Whole Blood	Blood	GTEX-RNOR-0005-SM-2TF4Z
Whole Blood	Blood	GTEX-RTLS-0006-SM-2TF58
Whole Blood	Blood	GTEX-RU1J-0006-SM-2TF6M
Whole Blood	Blood	GTEX-RU72-0006-SM-2TF65
Whole Blood	Blood	GTEX-RUSQ-0006-SM-2TF6P
Whole Blood	Blood	GTEX-RVPV-0006-SM-2TF6Q
Whole Blood	Blood	GTEX-RWS6-0005-SM-2XCAN
Whole Blood	Blood	GTEX-RWSA-0005-SM-2XCAO
Whole Blood	Blood	GTEX-S32W-0006-SM-2XCAK
Whole Blood	Blood	GTEX-S33H-0005-SM-2XCAL
Whole Blood	Blood	GTEX-S341-0006-SM-3NM8D
Whole Blood	Blood	GTEX-S7PM-0006-SM-3NM8C
Whole Blood	Blood	GTEX-S7SE-0005-SM-2XCEA
Whole Blood	Blood	GTEX-S95S-0005-SM-2XCEC
Whole Blood	Blood	GTEX-SE5C-0006-SM-4BRW5
Whole Blood	Blood	GTEX-SIU8-0006-SM-2XCE5
Whole Blood	Blood	GTEX-SJXC-0005-SM-2XCE7
Whole Blood	Blood	GTEX-SN8G-0006-SM-32PLD
Whole Blood	Blood	GTEX-SNMC-0006-SM-2XCFE
Whole Blood	Blood	GTEX-SNOS-0006-SM-32PLH
Whole Blood	Blood	GTEX-SSA3-0005-SM-32QOT
Whole Blood	Blood	GTEX-T2YK-0005-SM-32QOV
Whole Blood	Blood	GTEX-T5JC-0005-SM-4DM7B
Whole Blood	Blood	GTEX-T5JW-0005-SM-3GADE
Whole Blood	Blood	GTEX-T6MN-0005-SM-32PLJ
Whole Blood	Blood	GTEX-T6MO-0006-SM-32QOU
Whole Blood	Blood	GTEX-T8EM-0006-SM-3DB71
Whole Blood	Blood	GTEX-TKQ1-0006-SM-33HBI
Whole Blood	Blood	GTEX-TKQ2-0006-SM-33HBH
Whole Blood	Blood	GTEX-TML8-0005-SM-32QPA
Whole Blood	Blood	GTEX-TMMY-0005-SM-33HBN
Whole Blood	Blood	GTEX-TMZS-0006-SM-3DB8G
Whole Blood	Blood	GTEX-TSE9-0005-SM-4DXUF
Whole Blood	Blood	GTEX-U3ZG-0006-SM-47JWX
Whole Blood	Blood	GTEX-U3ZH-0005-SM-3DB72
Whole Blood	Blood	GTEX-U3ZN-0006-SM-3DB7Y
Whole Blood	Blood	GTEX-U412-0006-SM-3DB8J
Whole Blood	Blood	GTEX-U4B1-0006-SM-3DB8E
Whole Blood	Blood	GTEX-U8T8-0005-SM-3DB8F
Whole Blood	Blood	GTEX-U8XE-0005-SM-3DB8I

Whole Blood	Blood	GTEX-UJHI-0006-SM-3DB8H
Whole Blood	Blood	GTEX-UJMC-0005-SM-3GACU
Whole Blood	Blood	GTEX-UPJH-0006-SM-3GACW
Whole Blood	Blood	GTEX-UPK5-0006-SM-3GAD8
Whole Blood	Blood	GTEX-UTHO-0006-SM-3NMCC
Whole Blood	Blood	GTEX-V1D1-0006-SM-3NMCE
Whole Blood	Blood	GTEX-V955-0005-SM-3P5ZC
Whole Blood	Blood	GTEX-VJWN-0005-SM-3GIKF
Whole Blood	Blood	GTEX-VJYA-0005-SM-3P5ZD
Whole Blood	Blood	GTEX-VUSG-0006-SM-3GIK9
Whole Blood	Blood	GTEX-VUSH-0005-SM-3NB2H
Whole Blood	Blood	GTEX-W5WG-0006-SM-3GIJT
Whole Blood	Blood	GTEX-W5X1-0006-SM-3GIJZ
Whole Blood	Blood	GTEX-WCDI-0005-SM-3NB2M
Whole Blood	Blood	GTEX-WEY5-0006-SM-3GIKG
Whole Blood	Blood	GTEX-WFG7-0005-SM-3GIKM
Whole Blood	Blood	GTEX-WFG8-0006-SM-3GIKS
Whole Blood	Blood	GTEX-WFJO-0005-SM-3GIKY
Whole Blood	Blood	GTEX-WFON-0005-SM-3NMC9
Whole Blood	Blood	GTEX-WH7G-0005-SM-3NMBX
Whole Blood	Blood	GTEX-WHPG-0006-SM-3NMBV
Whole Blood	Blood	GTEX-WHSB-0005-SM-3LK7C
Whole Blood	Blood	GTEX-WHSE-0006-SM-3NMBW
Whole Blood	Blood	GTEX-WHWD-0005-SM-3LK7D
Whole Blood	Blood	GTEX-WK11-0006-SM-3NB3J
Whole Blood	Blood	GTEX-WL46-0006-SM-4OOS5
Whole Blood	Blood	GTEX-WOFL-0006-SM-3TW8K
Whole Blood	Blood	GTEX-WOFM-0005-SM-3MJF3
Whole Blood	Blood	GTEX-WQUQ-0006-SM-3MJF4
Whole Blood	Blood	GTEX-WRHK-0005-SM-3MJF5
Whole Blood	Blood	GTEX-WRHU-0006-SM-3MJF6
Whole Blood	Blood	GTEX-WVLH-0006-SM-3MJF7
Whole Blood	Blood	GTEX-WWYW-0005-SM-3NB3K
Whole Blood	Blood	GTEX-WXYG-0005-SM-3NB3M
Whole Blood	Blood	GTEX-WY7C-0006-SM-3NB3L
Whole Blood	Blood	GTEX-WYBS-0005-SM-4ONDR
Whole Blood	Blood	GTEX-WYJK-0005-SM-3NMA1
Whole Blood	Blood	GTEX-WYVS-0006-SM-3NMA7
Whole Blood	Blood	GTEX-WZTO-0006-SM-3NM9T
Whole Blood	Blood	GTEX-X15G-0005-SM-3NMDA
Whole Blood	Blood	GTEX-X261-0005-SM-4PQYX
Whole Blood	Blood	GTEX-X3Y1-0006-SM-3P5ZG
Whole Blood	Blood	GTEX-X4EO-0006-SM-3P5ZF
Whole Blood	Blood	GTEX-X4EP-0005-SM-3P5ZE
Whole Blood	Blood	GTEX-X4XX-0005-SM-3NMCS
Whole Blood	Blood	GTEX-X4XY-0006-SM-46MV2
Whole Blood	Blood	GTEX-X585-0005-SM-46MV3
Whole Blood	Blood	GTEX-X5EB-0006-SM-46MV5
Whole Blood	Blood	GTEX-X62O-0005-SM-46MV1
Whole Blood	Blood	GTEX-X638-0005-SM-47JX6
Whole Blood	Blood	GTEX-X88G-0006-SM-47JX5
Whole Blood	Blood	GTEX-X8HC-0006-SM-46MV6
Whole Blood	Blood	GTEX-XAJ8-0006-SM-46MVM
Whole Blood	Blood	GTEX-XBEC-0006-SM-4AT5T

Whole Blood	Blood	GTEX-XBED-0006-SM-47JXO
Whole Blood	Blood	GTEX-XBEW-0006-SM-4AT4E
Whole Blood	Blood	GTEX-XGQ4-0005-SM-4AT5U
Whole Blood	Blood	GTEX-XK95-0005-SM-4AT4S
Whole Blood	Blood	GTEX-XLM4-0005-SM-4AT4P
Whole Blood	Blood	GTEX-XMD1-0006-SM-4AT4Q
Whole Blood	Blood	GTEX-XMD2-0006-SM-4WWEG
Whole Blood	Blood	GTEX-XMD3-0006-SM-4AT5X
Whole Blood	Blood	GTEX-XMK1-0005-SM-4B665
Whole Blood	Blood	GTEX-XOT4-0005-SM-4B64S
Whole Blood	Blood	GTEX-XPT6-0006-SM-4B66Q
Whole Blood	Blood	GTEX-XPVG-0006-SM-4B65Z
Whole Blood	Blood	GTEX-XQ3S-0006-SM-4BOQ4
Whole Blood	Blood	GTEX-XQ8I-0006-SM-4BOQ5
Whole Blood	Blood	GTEX-XUW1-0005-SM-4BOQ7
Whole Blood	Blood	GTEX-XUYS-0005-SM-47JZ2
Whole Blood	Blood	GTEX-XUZC-0005-SM-4BOQ8
Whole Blood	Blood	GTEX-XXEK-0005-SM-4BRWJ
Whole Blood	Blood	GTEX-XYKS-0005-SM-4BRUD
Whole Blood	Blood	GTEX-Y111-0006-SM-4SOIO
Whole Blood	Blood	GTEX-Y114-0006-SM-4TT76
Whole Blood	Blood	GTEX-Y3I4-0006-SM-4TT1E
Whole Blood	Blood	GTEX-Y3IK-0005-SM-4WWDE
Whole Blood	Blood	GTEX-Y5LM-0005-SM-4V6EJ
Whole Blood	Blood	GTEX-Y5V5-0006-SM-4V6FE
Whole Blood	Blood	GTEX-Y5V6-0005-SM-4V6FD
Whole Blood	Blood	GTEX-Y8DK-0005-SM-4RGNR
Whole Blood	Blood	GTEX-Y8E4-0006-SM-4V6EW
Whole Blood	Blood	GTEX-Y8E5-0006-SM-47JWQ
Whole Blood	Blood	GTEX-Y8LW-0005-SM-4V6EV
Whole Blood	Blood	GTEX-Y9LG-0006-SM-4VBRK
Whole Blood	Blood	GTEX-YB5E-0005-SM-4VDSO
Whole Blood	Blood	GTEX-YB5K-0005-SM-4VDSP
Whole Blood	Blood	GTEX-YBZK-0005-SM-59HKG
Whole Blood	Blood	GTEX-YEC3-0005-SM-4W21J
Whole Blood	Blood	GTEX-YEC4-0001-SM-4W1Y6
Whole Blood	Blood	GTEX-YECK-0005-SM-4W217
Whole Blood	Blood	GTEX-YF7O-0005-SM-4W1ZU
Whole Blood	Blood	GTEX-YFC4-0006-SM-4RGLV
Whole Blood	Blood	GTEX-YFCO-0005-SM-4W1ZI
Whole Blood	Blood	GTEX-Z93S-0005-SM-4RGLW
Whole Blood	Blood	GTEX-ZAB4-0005-SM-4RGM8
Whole Blood	Blood	GTEX-ZC5H-0005-SM-4WAXM
Whole Blood	Blood	GTEX-ZDTS-0006-SM-4WAYZ
Whole Blood	Blood	GTEX-ZDTT-0006-SM-4WKFP
Whole Blood	Blood	GTEX-ZDXO-0006-SM-4WKGF
Whole Blood	Blood	GTEX-ZDYS-0002-SM-4WKGR
Whole Blood	Blood	GTEX-ZE7O-0006-SM-51MTA
Whole Blood	Blood	GTEX-ZE9C-0006-SM-4WKG2
Whole Blood	Blood	GTEX-ZEX8-0005-SM-4WKGE
Whole Blood	Blood	GTEX-ZF28-0005-SM-4WKH3
Whole Blood	Blood	GTEX-ZF29-0006-SM-4WKGQ
Whole Blood	Blood	GTEX-ZF2S-0006-SM-4WKHF
Whole Blood	Blood	GTEX-ZF3C-0005-SM-4WWAR

Whole Blood	Blood	GTEX-ZG7Y-0006-SM-4WWEA
Whole Blood	Blood	GTEX-ZGAY-0006-SM-4WWAQ
Whole Blood	Blood	GTEX-ZLV1-0005-SM-4WWAP
Whole Blood	Blood	GTEX-ZP4G-0006-SM-4WWE6
Whole Blood	Blood	GTEX-ZPCL-0006-SM-4WWAS
Whole Blood	Blood	GTEX-ZPIC-0005-SM-4WWEB
Whole Blood	Blood	GTEX-ZPU1-0006-SM-4WWAT
Whole Blood	Blood	GTEX-ZQG8-0005-SM-4YCEH
Whole Blood	Blood	GTEX-ZQUD-0005-SM-4YCE5
Whole Blood	Blood	GTEX-ZT9W-0005-SM-4YCEG
Whole Blood	Blood	GTEX-ZTPG-0006-SM-4YCFG
Whole Blood	Blood	GTEX-ZTSS-0005-SM-4YCDR
Whole Blood	Blood	GTEX-ZTTD-0006-SM-51MSJ
Whole Blood	Blood	GTEX-ZTX8-0006-SM-4YCE4
Whole Blood	Blood	GTEX-ZUA1-0005-SM-4YCEV
Whole Blood	Blood	GTEX-ZV68-0006-SM-4YCEJ
Whole Blood	Blood	GTEX-ZV7C-0005-SM-57WDL
Whole Blood	Blood	GTEX-ZVE2-0006-SM-51MRW
Whole Blood	Blood	GTEX-ZVP2-0005-SM-51MRK
Whole Blood	Blood	GTEX-ZVT2-0005-SM-57WBW
Whole Blood	Blood	GTEX-ZVT3-0006-SM-51MT9
Whole Blood	Blood	GTEX-ZVT4-0006-SM-57WB8
Whole Blood	Blood	GTEX-ZVTK-0006-SM-57WBK
Whole Blood	Blood	GTEX-ZVZP-0006-SM-51MSW
Whole Blood	Blood	GTEX-ZVZQ-0006-SM-51MR8
Whole Blood	Blood	GTEX-ZXES-0005-SM-57WCB
Whole Blood	Blood	GTEX-ZXG5-0005-SM-57WCN
