# Supplementary material for: Analysis of splice variants of the human protein disulfide isomerase (P4HB) gene
Source: BMC Genomics. 2020 Nov 4;21:766. doi: 10.1186/s12864-020-07164-y (PMC7640458; doi:10.1186/s12864-020-07164-y)
Supplement: Supplementary file 4 — Additional file 4: Figure S1. Top CAGE peaks in TPM (tags per million) for FANTOM CAGE samples (A) and ENCODE CAGE samples. These graphs represent the samples with highest TPM. [file 12864_2020_7164_MOESM4_ESM.docx]

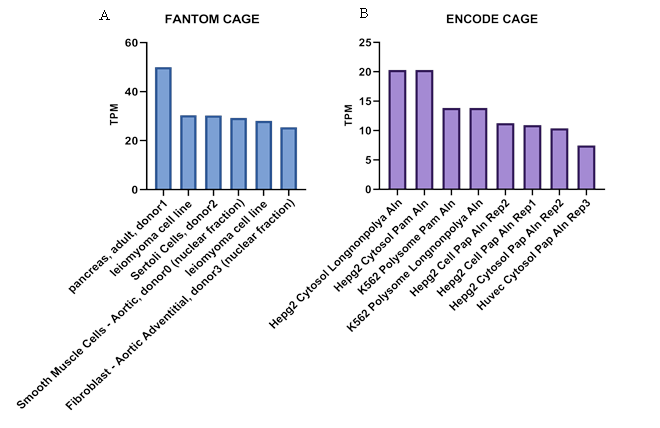


**Figure S1.** Top CAGE peaks in TPM (tags per million) for FANTOM CAGE samples (A) and ENCODE CAGE samples. These graphs represent the samples with highest TPM.
