# Supplementary material for: Analysis of splice variants of the human protein disulfide isomerase (P4HB) gene
Source: BMC Genomics. 2020 Nov 4;21:766. doi: 10.1186/s12864-020-07164-y (PMC7640458; doi:10.1186/s12864-020-07164-y)
Supplement: Supplementary file 5 — Additional file 5: Figure S2. Representative sashimi plots of 10 FANTOM5 samples showing 4 different cell lines for the region chr17: 79796651–79,822,949 obtained using sashimi-plot utility in IGV program. (A) The plot presents the entire P4HB gene with 11 exons in the bottom (blue). The lines indicate exon 3, with the specific junction for P4HB-027. The black arrow indicates the exon 3 and the splice junction of this isoform. [file 12864_2020_7164_MOESM5_ESM.docx]

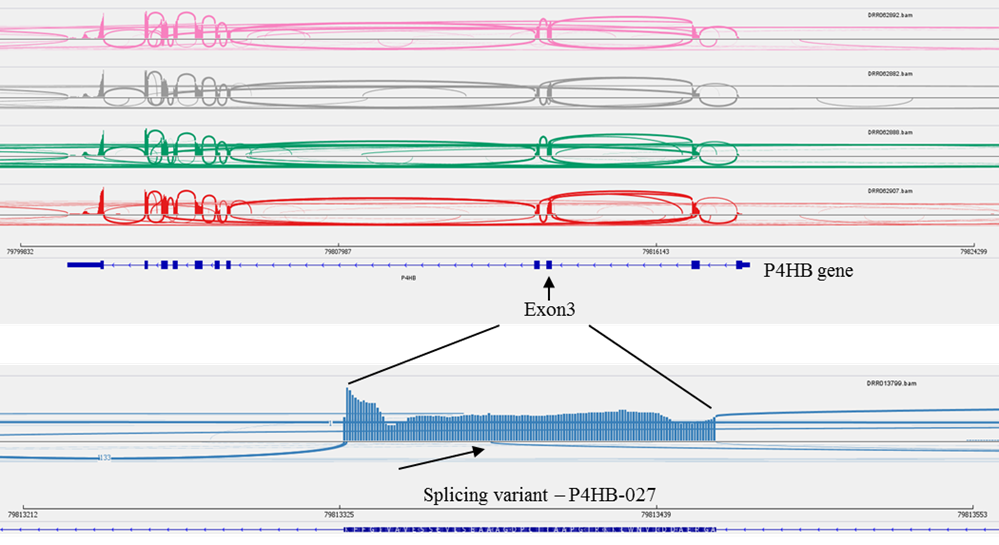


**FIGURE S2.** Representative sashimi plots of 10 FANTOM5 samples showing 4 different cell lines for the region chr17: 79796651-79822949 obtained using sashimi-plot utility in IGV program. (A) The plot presents the entire *P4HB* gene with 11 exons in the bottom (blue). The lines indicate exon 3, with the specific junction for *P4HB*-027. The black arrow indicates the exon 3 and the splice junction of this isoform.
