# Supplementary material for: Analysis of splice variants of the human protein disulfide isomerase (P4HB) gene
Source: BMC Genomics. 2020 Nov 4;21:766. doi: 10.1186/s12864-020-07164-y (PMC7640458; doi:10.1186/s12864-020-07164-y)

**FIGURE S3.** Quantification of *P4HB* splice variants to detect the fraction of isoform abundance normalized by *P4HB* gene. (A) Fraction of *P4HB*-02, *P4HB*-021 and *P4HB*-027 in blood vessels of three subtypes: aorta (n=299), coronary artery (n=172) and tibial artery (n=400) (B) Fraction of *P4HB* splice variants in heart with two sub-regions: atrial appendage (n=300) and left ventricle (n=300).


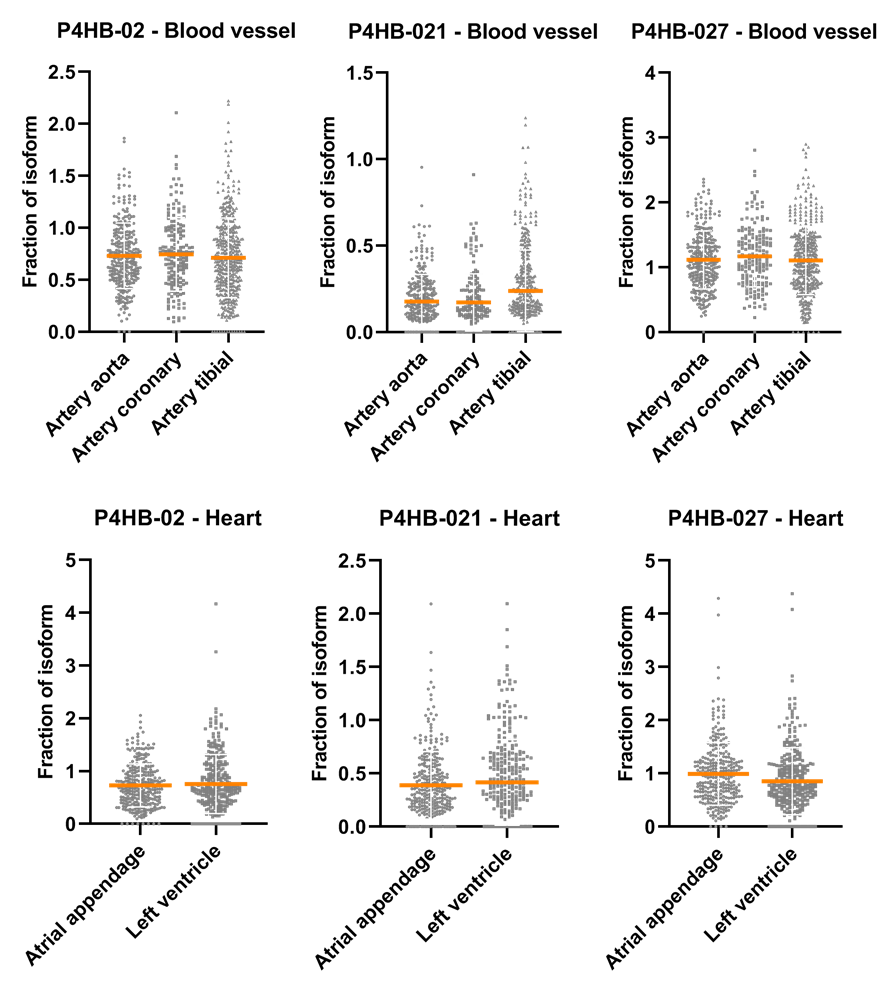

Supplement: Supplementary file 6 — Additional file 6: Figure S3. Quantification of P4HB splice variants to detect the fraction of isoform abundance normalized by P4HB gene. (A) Fraction of P4HB-02, P4HB-021 and P4HB-027 in blood vessels of three subtypes: aorta (n = 299), coronary artery (n = 172) and tibial artery (n = 400) (B) Fraction of P4HB splice variants in heart with two sub-regions: atrial appendage (n = 300) and left ventricle (n = 300). [file 12864_2020_7164_MOESM6_ESM.docx]
