# Supplementary material for: Analysis of splice variants of the human protein disulfide isomerase (P4HB) gene
Source: BMC Genomics. 2020 Nov 4;21:766. doi: 10.1186/s12864-020-07164-y (PMC7640458; doi:10.1186/s12864-020-07164-y)
Supplement: Supplementary file 7 — Additional file 7: Figure S4. Expression of P4HB gene and variants P4HB-02, P4HB-021 and P4HB-027. (A) P4HB and splice variant expression after exposure to tunicamycin for 16 h and (B) 40 h in HEK-293 cell line treated with tunicamycin (0.5 μg/mL, 1 μg/mL and 2 μg/mL). Results shown as fold change versus control sample (without tunicamycin treatment). Total RNA was used to perform RT-qPCR. [file 12864_2020_7164_MOESM7_ESM.docx]

**FIGURE S4.** Expression of *P4HB* gene and variants *P4HB-*02, *P4HB-*021 and *P4HB-*027. (A) *P4HB* and splice variant expression after exposure to tunicamycin for 16h and (B) 40h in HEK-293 cell line treated with tunicamycin (0.5µg/mL, 1µg/mL and 2µg/mL). Results shown as fold change versus control sample (without tunicamycin treatment). Total RNA was used to perform RT-qPCR.


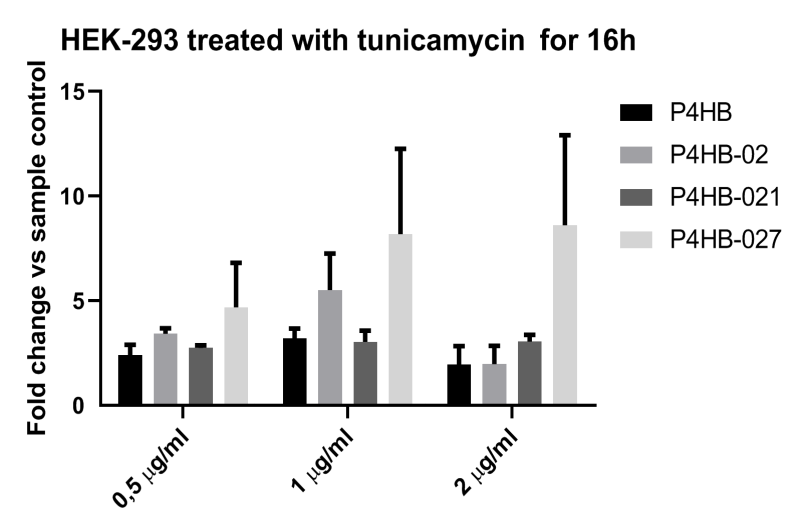

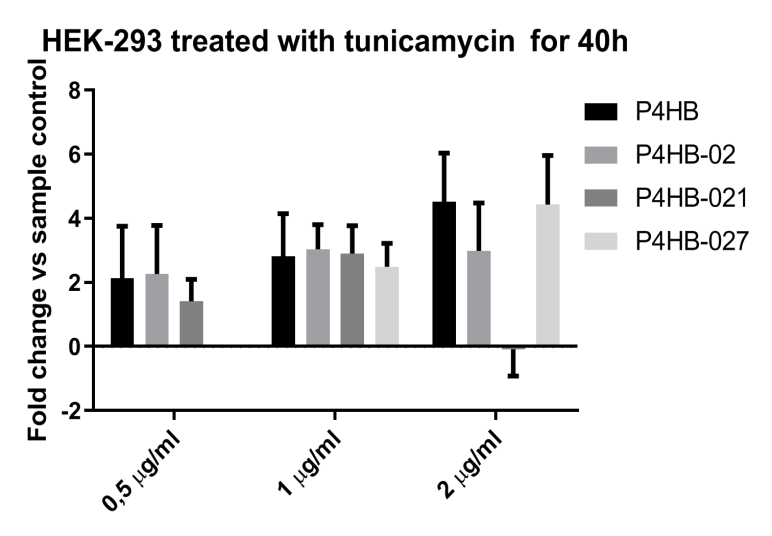


B

A
