# Supplementary material for: Analysis of splice variants of the human protein disulfide isomerase (P4HB) gene
Source: BMC Genomics. 2020 Nov 4;21:766. doi: 10.1186/s12864-020-07164-y (PMC7640458; doi:10.1186/s12864-020-07164-y)
Supplement: Supplementary file 8 — Additional file 8: Figure S5. Real-time PCR expression profiling of 8 genes and 3 P4HB splice variants in HEK-293 cells (A) exposed to tunicamycin (0.5 μg/mL, 1.0 μg/mL and 2.0 μg/mL) or (B) to CoCl2 for 10 h and 24 h. The heatmap was generated by a log transformation of the real-time PCR data presented as ∆Ct (CT gene of interest – CT reference gene). [file 12864_2020_7164_MOESM8_ESM.docx]

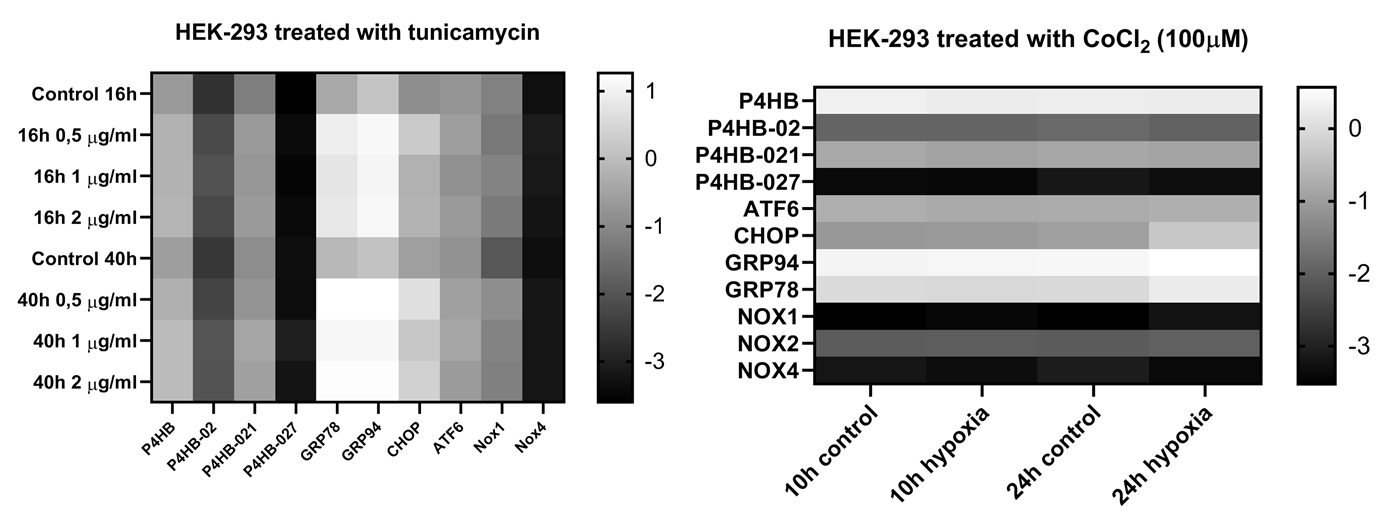


A

B

**FIGURE S5.** Real-time PCR expression profiling of 8 genes and 3 *P4HB* splice variants in HEK-293 cells (A) exposed to tunicamycin (0.5µg/mL, 1.0 µg/mL and 2.0 µg/mL) or (B) to CoCl_2_ for 10h and 24h. The heatmap was generated by a log transformation of the real-time PCR data presented as 🛆Ct (C_T_ gene of interest – C_T_ reference gene).
